# Combined Effects of Aircraft, Rail, and Road Traffic Noise on Total Noise Annoyance—A Cross-Sectional Study in Innsbruck

**DOI:** 10.3390/ijerph16183504

**Published:** 2019-09-19

**Authors:** Christoph Lechner, David Schnaiter, Stephan Bose-O’Reilly

**Affiliations:** 1 Department of Public Health, Health Services Research and Health Technology Assessment, UMIT—University for Health Sciences, Medical Informatics and Technology, 6060 Hall in Tirol, Austria; stephan.boeseoreilly@umit.at; 2 Office of the Tyrolean Regional Government, Department for Emission, Safety and Sites, 6020 Innsbruck, Austria; 3 Independent Researcher, 6020 Innsbruck, Austria; 4 Institute and Clinic for Occupational, Social and Environmental Medicine, University Hospital, LMU Munich, 80336 Munich, Germany

**Keywords:** combined noise effects, noise annoyance, traffic noise, exposure-response relationship, Total Noise Investigation Innsbruck

## Abstract

Noise legislation in Austria does not provide an assessment of the cumulative effect of noise from different sources. The desire of citizens for a total noise assessment is getting stronger. Within the pilot project “Gesamtlärmbetrachtung” (Total Noise Investigation) Innsbruck, data from 1031 face-to-face interviews were correlated with exposure data from road, rail and air traffic noise. The interviews were selected in clusters according to the exposure combinations of these three sources. In addition to exposure-response relationships, it has also been found that the annoyance response to air and rail traffic noise is independent of the background noise from road traffic. The total noise annoyance response shows a cumulative effect in each source considered. From the source specific exposure-response relationships, a total noise assessment model based on the annoyance equivalents model was developed. This model is more suitable than the dominant source model and thus also considerable for legal application.

## 1. Introduction

Noise pollution represents a major health problem in modern society [1] leading to multiple adverse health effects if not properly monitored and assessed. Among them: sleep disorders [2], learning impairment [3,4,5], ischemic heart disease [6,7], and especially annoyance reactions [8,9,10,11,12]. In order to attenuate these effects, studies and mitigating measures such as action plans [13] have been implemented worldwide in the last decades for the main transportation sources of noise affecting modern human lifestyle. A quantification of healthy life years lost in Europe through these health effects was estimated by WHO in 2011 [14]. For each outcome, based on exposure–response relationships, exposure distributions, background prevalence of disease and disability weights, the burden of disease in terms of disability-adjusted life-years (DALYs) can be calculated. Applying conservative assumptions to the calculation methods the WHO estimates DALYs lost from environmental noise by 61,000 years for ischemic heart disease, 45,000 years for cognitive impairment of children, 903,000 years for sleep disturbances, 22,000 years for tinnitus, and 654,000 years for annoyance in the European Union Member States and other western European countries.

Noise has been creating more and more challenges for decision-makers, especially in alpine urban areas. The aim of the pilot project “Gesamtlärmbetrachtung Innsbruck” (Total Noise Investigation Innsbruck), Ref. [15] was to create a representative, comprehensive dataset for assessing the overall noise burden and annoyance for the city population of Innsbruck for the first time and additional support data to create an overall noise assessment model. Overall noise assessment is the consideration of the cumulative effects of sound from different sources. This term is mainly used in the assessment of noise from traffic sources. In effect, differing combinations of road, rail, and aircraft noise are commonly shaping the soundscape of our living surroundings.

Many studies describe relationships between exposure and effects of the most important single noise sources like road traffic [10,16], railway traffic [17,18], airport and air traffic [19,20,21], and harbors [22,23]. In a broader sense, total noise is the sum of the effects of all sound, be it from traffic, industrial plants, passerby, neighboring, construction sites, or even natural sounds such as those caused by wind or water. In this paper, the term is used only for the combination of traffic noise sources. On the one hand, an overall noise assessment of affected citizens is repeatedly demanded; on the other hand, the involved experts criticize the lack of evidence from field studies. According to a decision of the Federal Administrative Court it seems clear that a total noise assessment does not correspond to the state of the art in health sciences and other relevant sciences [24] in the Austrian jurisdiction.

A recent publication from R. Guski and colleagues on noise annoyance with a meta-analysis of 57 studies included five studies on noise source combinations published in four papers. All studies include road traffic noise; two of the studies combine road and railway noise, two combine road and aircraft noise, and one combines road and industrial noise. The total dataset includes 1949 participants. After performing the analyses, however, it became apparent that it was unwise to integrate different noise source combinations into one single analysis. Unfortunately, there were not enough studies available even for the meta-analysis of combinations of two sources. With respect to the weights given for the separate noise levels in future combination studies, the results point to the importance of a dominant source model where annoyance is assessed according to the strongest component [25] in terms of annoyance and underline the existing need for further research on total noise assessment for all three traffic noise sources: road, rail and air traffic [26].

The 2015 NORAH study [27], which was conducted in the vicinity of Frankfurt Airport, dealt with the combined effect of aircraft and rail traffic noise, as well as aircraft and road traffic noise, and showed a preference for a nuisance model for the unweighted 24-h continuous sound level according to the loudest source (dominant-source model) [28]. The peculiarity of this study also lies in the significantly different exposure-effect relationship of aircraft noise compared to the other two sources, road and rail traffic. According to the authors, this was based on the special political significance of Frankfurt Airport at the time of the survey. The authors stated that the transferability of their results is therefore limited.

Several other recent studies have dealt with the combined effect of two traffic noise sources. In a French study with a total number of 1010 of respondents in 8 cities, models for the combined annoyance from road and rail traffic noise were tested [29]. In these studies the Miedema annoyance equivalents model [30] for all three sources was applied for the highly annoyed, annoyed, and (at least) a little annoyed groups according to annoyance reactions identified and based on interview surveys. The determinations of the exposures were grouped. A thesis-testing was not carried out in this study, but the survey results corresponded with the theoretical ones [31].

In Montreal, a study was conducted into the common effects of traffic noise sources, namely road, rail and air traffic noise, as well as other noise sources [32]. However, the study design and results are not easily transferable as the authors themselves already emphasized that the reactions of their respondents were very different from those in Europe. The determination of noise effects could only be grouped or merely estimated. A total noise model describing the common effect based on noise levels or noise indicators was not included.

Specifically, there is a research gap consisting of the lack of field studies in which the overall noise effects regarding annoyance as a result of the effects of road, rail and aircraft noise are directly and for this purpose investigated. In addition, there is a need for more evidence for the basic assumption of the annoyance equivalents model with respect to the independence of variables [30].

The research questions were therefore formulated as follows:

(1) Is there a relevant influence of road traffic noise on the annoyance response to rail traffic noise?
(2) Is there a relevant influence of road traffic noise on the annoyance response to air traffic noise?
(3) Does the influence of road traffic noise in combination with rail traffic noise and air traffic noise lead to an increased overall noise annoyance (total noise annoyance)?

An overall sleep disturbance model due to different noise sources is not in the content of this paper.

## 2. Materials and Methods

### 2.1. Design and Sample

As a member state of the European Union, Austria is obliged to carry out strategic noise mapping every 5 years. Moreover, all sources must be mapped because Innsbruck is an agglomeration in the sense of the Environmental Noise Directive (END) [33]. Innsbruck is the capital of the State of Tyrol in the western part of Austria. It has approximately 130,000 inhabitants. The traffic infrastructure encloses a motorway in the south, a major railway that crosses the town from north to south with a junction to the west and a typical provincial airport with over 35,000 flight movements a year. A tramway connects the inner-city regions. The town is embedded in a mountainous surrounding; the river Inn—The origin for the name of the city—Divides the town into a southern and a northern part. While the city center shows typical urban building structure and population density, the outskirts have a more rural character. According to Statistik Austria’s (Austria’s National Office of Statistics) micro-census, Innsbruck is the most suitable place to study the combined effects of the three major traffic noise sources in Austria because all three noise sources are annoying to a considerable extent [34]. To get additional benefits of the noise mapping, the resulting exposure data were used to carry out a combined noise analysis. A cross-sectional study design was implemented in 2017 with a sample number of at least 1000 personally interviewed persons from all over Innsbruck [15]. The sampling was executed according to three basic principles:

- Demographic representativeness;
- Representative image of the 9 districts of Innsbruck;
- Classification according to given noise corridors corresponding to the noise pollution originating from main road traffic, railroad and air transport.

A random sample selection within defined clusters by given age, gender, district inhabitants and noise exposure groups was analyzed. The noise exposure groups were ranked as little affected address points with L_den_ lower than 45 dB, medium affected with L_den_ between 45 and 55 dB and strongly affected with L_den_ exceeding 55 dB. Between 16 May and 8 July 2017, a total of 1031 face-to-face interviews were completed by 23 interviewers, which were thematically specially trained, in the urban area of Innsbruck, carried out by the Institut für Marktforschung und Datenanalyse (IMAD, Institute for Market Research and Data Analysis). The completed questionnaires were digitized following the highest quality assurance procedures and data protection requirements [35], correlated with the address and noise data and prepared for the continuing processed statistical evaluation.

The real distribution of female and male inhabitants in Innsbruck is 51.3%/48.7% [36]. In the survey a distribution of 52.5/47.5 was achieved, constituting a deviation of 1.2%. The age requirements were categorized into 3 age groups (18–40, 41–60, >60) and deviated to their largest extent by less than 1% in the surveys from the real distribution of Innsbruck’s total population.

The sample also followed the different exposure groups of road, rail and air traffic noise. In each of the 9 noise corridors at least 111 complete interviews had to be carried out (see Table 1). This was achieved successfully.

### 2.2. Means and Instruments

#### 2.2.1. Exposure

The exposure of noise was predicted according to the Environmental Noise Directive with the Austrian legal implementation [37]. We created a three-dimensional model of the entire city. The terrain model is based on a 3D point cloud resulting from the laser scan localization, which has been prepared according to defined requirements for the present project area. The laser scanning was carried out by the State of Tyrol in the years 2012 to 2015 [38]. The maximum vertical distance between two height points was chosen to be 0.1 m. In addition, a maximum horizontal distance of 10 m was set.

In the evaluation of the buildings, the building polygon determined by the laser scan positioning was reduced in circumference by one meter to eliminate any bottom stitch of the laser scan location as this would be significant in the calculation of the building height impact. To obtain the height of the building we calculated the arithmetic mean of minimum and maximum value within the reduced polygon, complying with the requirements of the Austrian guidance [39].

Both the spatial information about the road network of the project area and relevant information for the traffic on it were provided by the city of Innsbruck. The traffic flow is derived from a traffic model calibrated via permanent counting stations and additional manual sampling counts. Traffic flow on the motorway was supplied by the highway company and derived from permanent counting stations.

The spatial information for railway network and the project area as well as the number of trains classified according to the individual rail types was given by the Austrian railway company and derived from the operating program in 2015. Speed was modeled as the minimum from admissible maximum speed in the respective route section and maximum speed for each type of train used. Own validation measurements were not carried out for rail traffic noise, as the uncertainties of the Austrian rail traffic prediction model is well known with ±2 dB and has been recently validated in the framework of the lower Inn valley [40].

We used radar data of each aircraft movement on its specific flight path and converted the data to emission lines. These radar data contain information of flight altitude, aircraft type and speed. As a special characteristic of Innsbruck airport half of the flight movements over the year take place between December and March (winter charter), i.e., the L_den_ over these 4 months is 1.5 dB higher than the yearly average L_den_.

The calculation considered reflections of the sound path on objects of 1st order. In addition to the calculation, we performed a long-term measurement to demonstrate that the calculation and adding of different sound levels work appropriately. The differences between calculation and measurement under reproducible conditions were in the well-known range of ±2 dB.

To predict the noise exposure in the reference year 2015, Austrian interim methods for road traffic noise [41] and rail traffic noise [42] were used. Air traffic noise was predicted according to the common noise assessment method [43]. 

Receiver points were assigned to the facades of buildings in a height of 4 m in 2 m distances. As a result, the loudest facade levels for road, rail and air traffic noise were associated to each address. These levels were used to classify the inhabitants and dwellings. The grid width for the noise maps was chosen as 10 m × 10 m. The input data was taken from the 2015 collection for strategic noise mapping. The velocities of each vehicle category were modelled according to the legal speed limits.

#### 2.2.2. Questionnaire

Quantitative data on noise sensitivity as well as annoyance reactions of the population of Innsbruck are not yet sufficiently available. In order to obtain valid data, we developed a questionnaire based partly on validated and proven items and scales and partly on self-developed and validated questions. The focus was set on the perception of traffic noise and the resulting annoyance reactions of the population of Innsbruck.

The questions about exposure and annoyance from traffic noise, as well as the corresponding scales (mostly 11 point) are based on the recommendations of the ICBEN (International Commission on Biological Effects of Noise) [44,45], the EU-SILC 2015 [46], the LEF-K for noise sensitivity [47], the 5-item scale for sleep disturbance and the Austrian Micro-census “Environmental Conditions” [34]. In addition we linked validated items from comparable studies like NORAH [27] and preliminary surveys in the Tyrolean area [40]. The standardized socio-demographic questions could be used for the most part.

The section in the questionnaire dealing with annoyance in total and annoyance from single sources contained the following wording (translation from German):

“Thinking about noise the last 12 months, when you are here at home, how much does noise disturb or annoy you on a scale from 0 to 10. If you are not at all annoyed choose 0; if you are extremely annoyed choose 10; if you are somewhere in between, choose a number between 0 and 10.

- from noise in total
- from motorway noise
- from road traffic noise
- from rail traffic noise
- from air traffic noise
- from noise from craft and industry
- from noise from passers-by and pubs
- from construction noise
- from neighborhood noise
- from vibration in total”

In many parts, the items of the questionnaire were developed by the authors. After consolidation and harmonization of the questionnaire, statistical reliability tests and a series of test interviews for validation were conducted as shown in Table 2 below.

The core areas in the questionnaire were:

- socio-demographics (age, gender, profession, level of education etc.)
- housing situation (question of residence, type of house, household size, floor, noise protection windows, barriers, patios etc.)
- noise perception (exposure, perception, wind and weather influences)
- living conditions and quality of life (quality of living conditions, transport links, safety, landscape and nature, neighborhood relationships, conditions for children, connectedness to the city, satisfaction with the housing situation)
- subjective assessment of personal quality of life
- subjective assessment of personal sensitivity to noise
- subjective assessment of personal health
- annoyance/disturbance through noise
- sleep disturbance through noise
- noise coping (closed windows, changed use of living space, anger, helplessness, conversation with neighbors, talks with authorities, changes in noise pollution in recent years etc.)
- mobility (use of public transport, car, train, aircraft, bike, footpaths, satisfaction with public transport, commuter question)

Descriptive statistics are published [15], available as File S1 in the Supplementary Materials and summarized in Table 3.

Each participant signed a consent form. The study was reported to the university research committee for scientific and ethical questions (RCSEQ) at UMIT. The RCSEQ replied that since no personal sensitive data is processed in this research project (retrospective analysis of anonymized data), a RCSEQ vote is not required. The scientific work has been documented as an RCSEQ report. Data protection is given by the Government of the State of Tyrol, fulfilling all legal requirements. 

#### 2.2.3. Response Rate

The response rate is considered as the ratio of successful (complete) interviews to the encounter of potential interview persons fitting in the cluster sample.

As a result of several efforts like selection and training of the interviewers, intense field preparation throughout different media, and consideration of people’s life behavior for interview times, a response rate of 47.8% was achieved (see Table 4).

### 2.3. Statistical Analyses

The evaluation was carried out by descriptive statistics and univariate evaluations as to whether significant differences in the distribution of characteristics exist. This was done by group comparisons. Non-parametric methods such as Mann-Whitney U test and Kruskal-Wallis test were used as test methods after analyzing the distribution form. Furthermore, correlations of covariates were examined with each other in order to decide which features are included in the overall noise model. The covariates for logistic regression were determined by applying univariate analysis. The purposeful selection process begins with univariate analysis of each variable. Any variable having a significant univariate test at some arbitrary level was selected as a candidate for the multivariate analysis. We based this on the Wald test from logistic regression and a p-value cut-off point of 0.2. Variables that are significant at the 0.1 level were put in the model, and the model was iteratively reduced as before but only for the variables that were additionally added [48].

Exposure-response curves were calculated for the noise indicators, L_den_. Source-specific exposure-response curves on the percentage of highly annoyed people were calculated by means of binary logistic regression analysis in a generalized linear model (GLM).

Linear regression analyses were calculated to quantify the impact on the specific noise annoyance of the single noise sources.

The hypotheses in Table 5 were answered by the generalized equation:

log odds (A_dich_) = *β*_0_ + *β*_1_ L_road_ + *β*_2_ L_rail_ + *β*_3_ L_air_ + T_effect_, (1)

where T_effect_ = *β*_4_ L_road_ L_rail_ + *β*_5_ L_road_ L_air_ + *β*_6_ L_rail_ L_air_ + *β*_7_ L_road_ L_rail_ L_air_ (2)

In addition to the research question regarding interaction of road noise with noise from other sources, it was assessed if there was an interaction between rail and aircraft noise, where H1 was defined as *β*_6_ ≠ 0.

A_dich_ dichotomous annoyance response for highly annoyed (8 to 10) and annoyed (5 to 10):
L_road_ L_den_ caused by road traffic noise
L_rail_ L_den_ caused by rail traffic noise
L_air_ L_den_ caused by air traffic noise
T_effect_ effect modifier due to the influence of the simultaneous prevalence of different traffic noise sources.

For the development of a total noise assessment model we investigated the curve fits between L_den_ and the response on the 11-point annoyance scale. This was done for a linear fit like in Miedemas model [30] with and without a constant term. Furthermore, curve fits were calculated with a quadratic function, once with and once without a constant term. A third-order function control was performed to quantify the increase of correlation. Using these curves the annoyance equivalents model was constructed with equally annoying road traffic levels for air traffic and for rail traffic as introduced by Miedema [30] and used in a German guideline [49].

## 3. Results

### 3.1. Descriptive Analysis of Combined Noise Data

**Figure 1:**
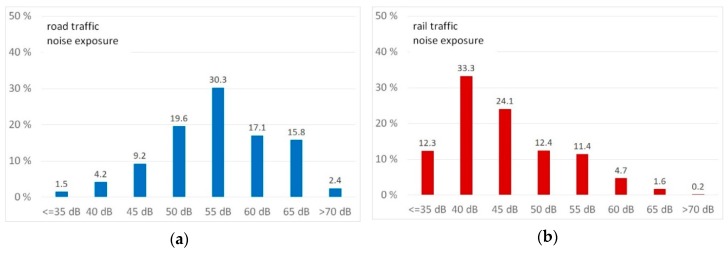

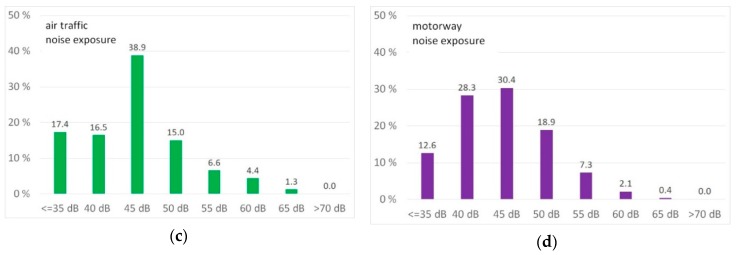
Distribution of participants by noise exposure from road traffic noise (**a**) rail traffic noise (**b**), air traffic noise (**c**), and motorway noise (**d**) in 5 dB L_den_ bands.

Although the random selection of the participants was equally distributed within little, medium, and strongly affected noise clusters of each traffic noise source, the distribution within 5 dB noise bands differs due to the characteristics of the different sources. Figure 1 shows histograms according to the percentage of the 1031 participants in each noise band.

**Figure 2:**
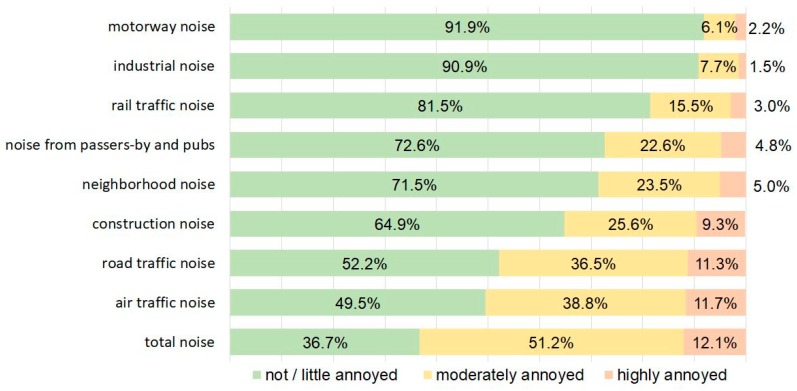
Distribution of noise annoyance from different sources according to annoyance groups.

Noise maps indicating the L_den_ for road traffic noise (Figure S1), rail traffic noise (Figure S2), air traffic noise (Figure S3) and motorway noise (Figure S4) are in the Supplementary Material. The distribution of noise annoyance according to different sources is shown in Figure 2 grouped in not or little annoyed (answer score lower than 5), moderately annoyed (answer score 5 to 7) and highly annoyed (answer score 8 to 10). For example, for the most prevalent noise source, road traffic, 52.2% of the recipients are not or little annoyed, 36.5% are moderately annoyed, and 11.3% are highly annoyed.

### 3.2. Purposeful Selection of Covariates in the Logistic Regression

The influence of the given variables on the response on total noise annoyance expressed as highly annoyed and annoyed is shown in Table 6.

In the iterative process of variable selection, covariates were removed from the model if they were non-significant. Significance was evaluated at the 0.1 alpha level and confounding as a change in any remaining parameter estimate greater than approximately 15 to 20% as compared to the full model. A change in a parameter estimate above the specified level indicates that the excluded variable was important in the sense of providing a needed adjustment for one or more of the variables remaining in the model. At the end of this iterative process of deleting, refitting, and verifying, the model only contained significant covariates. At this point any variables not selected for the original multivariate model were added back one at a time, with significant covariates retained earlier.

After these steps, the final model included self-reported noise sensitivity, access to a quiet facade and the installation of noise protection windows within the last 10 years. The duration of living within the same residence is eliminated because it shows up only within the highly annoyed and not for the annoyed group. The typical epidemiological parameters gender and age as confounders do not meet the inclusion criteria.

### 3.3. Source-Specific Exposure—Response Relationship for Traffic Noise Annoyance

A logistic regression analysis was conducted to assess the exposure–response relationship of noise annoyance and single, source-specific traffic noise by aircraft, road traffic and railway noise. The exposure to aircraft noise ranged from 31 to 66 dB L_den_ (M = 44.60, Std = 6.971). The exposure to road traffic noise ranged from 25 to 72 dB L_den_ (M = 55.12, Std = 7.446). The range of exposure to railway noise covered 21 to 69 dB L_den_ (M = 44.78, Std = 7.628).

**Figure 3:**
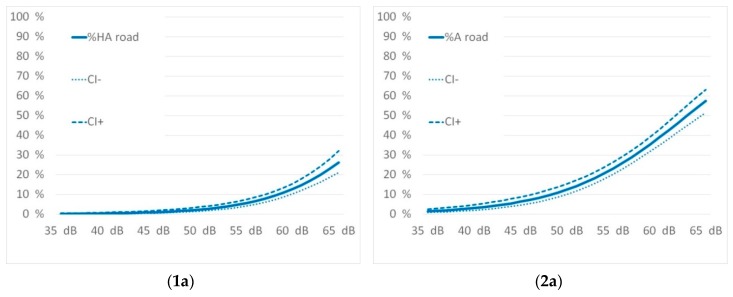

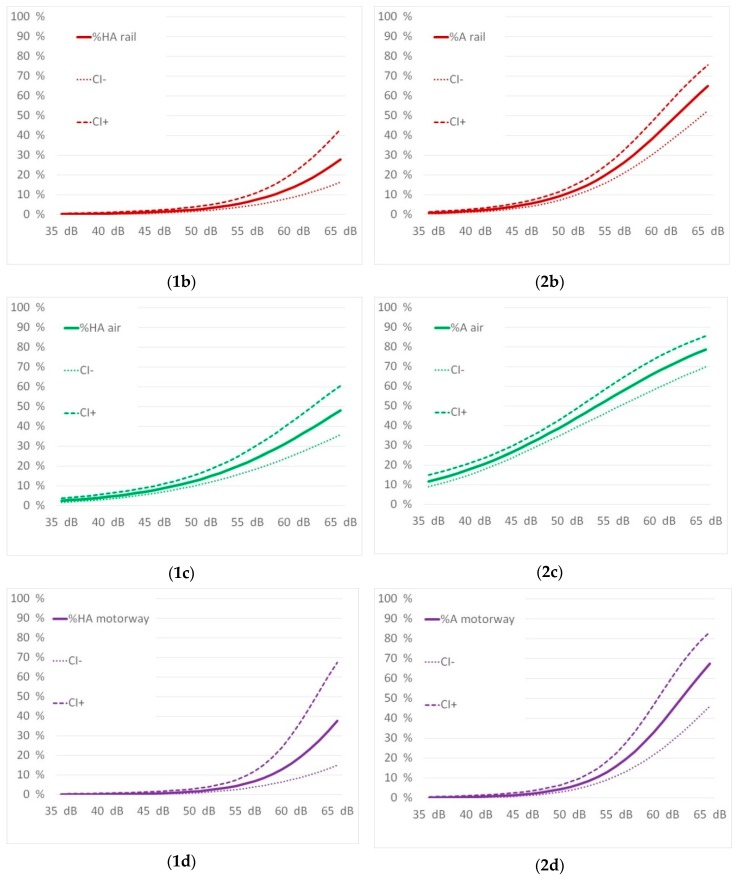
Exposure response relationship for percentage highly annoyed (**1**) and percentage annoyed (**2**) with their confidence intervals for road (**a**), rail (**b**), and air traffic noise (**c**) and for the subgroup motorway noise (**d**).

Source-specific exposure-response relationship for traffic noise annoyance for “highly annoyed” and “annoyed” are shown in Figure 3 below:

### 3.4. Influence of Road Traffic Noise Sources on Annoyance to Rail and Air Traffic Noise

Binary logistic regressions were used to analyze if there is an influence of background noise caused by road traffic in the annoyance response to rail and air traffic noise. According to the results of the bivariate screening, self-reported noise sensitivity was included in these analyses (see Table 7).

The results in Table 7 below show no significant influence of road traffic noise as background sound on noise annoyance caused by specific sources neither in aircraft nor in rail traffic noise. The binary logistic regression for the generalized model with all interaction terms for the three noise sources are summarized in Table 8.

The analysis of contributions resulting from the interaction term for all background noise combinations did not show any significant effect neither for the dichotomized groups for highly annoyed nor for annoyed. All interaction terms are non-significant.

In the next step, it was examined how the overall noise annoyance depends on the specific annoyance scores of single noise producers. This was done by applying a linear regression. As covariate, all annoyance scores for noise sources as well as age, gender, educational level and subjective assessment of noise sensitivity were included. The results are shown in Table 9 below.

**Figure 4:**
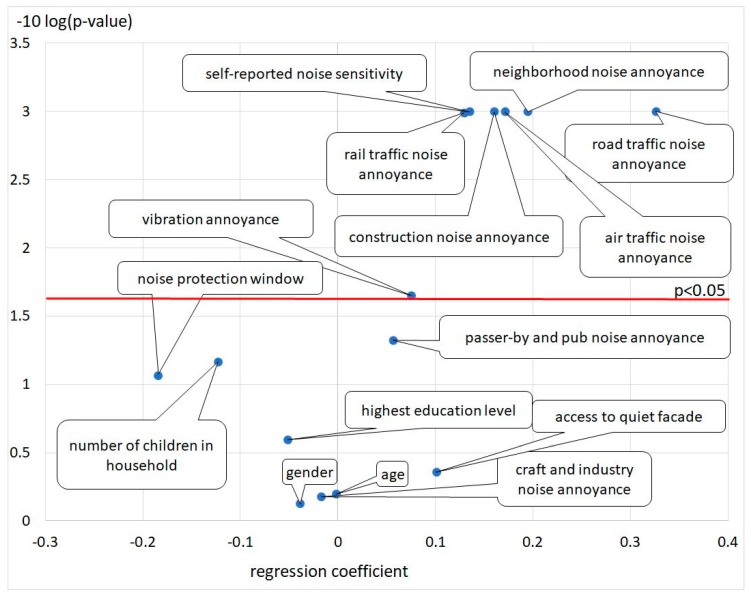
Significance and effect size of the variables in the model and level of significance *p* = 0.05 (red line), *p*-values < 0.001 are shown by a −10 log (*p*-value) of 3.

Figure 4 gives an overview of the significance and effect sizes of the variables in this model.

### 3.5. Total Annoyance Assessment Model forTraffic Noise

The exposure response curves were presented as a logit function. We investigated which curves were the most suitable for the annoyance equivalents model. In a first step, a curve fit was tried as in Miedema [30] as a linear function with and without a constant term. In the second step, a curve fit was calculated by a quadratic function, once with and once without a constant term. Significantly better values for R^2^ were shown, especially for rail traffic noise. These are even higher without a constant term. A third-order function check showed that the quadratic function suited best, since it was better suited for further calculations and the values for R^2^ were no longer significantly increased.

Table 10 shows the value R^2^ for the different curve fit models as follows.

For the purpose of an adjusted regression, a GLM was conducted. According to the analysis for the exposure response relationships, the final models were adjusted for self-reported noise sensitivity, access to a quiet facade and installation of noise protection windows within the last 10 years. The results of the regression analyses by GLM for the quadratic model are given below (see Table 11).

**Figure 5:**
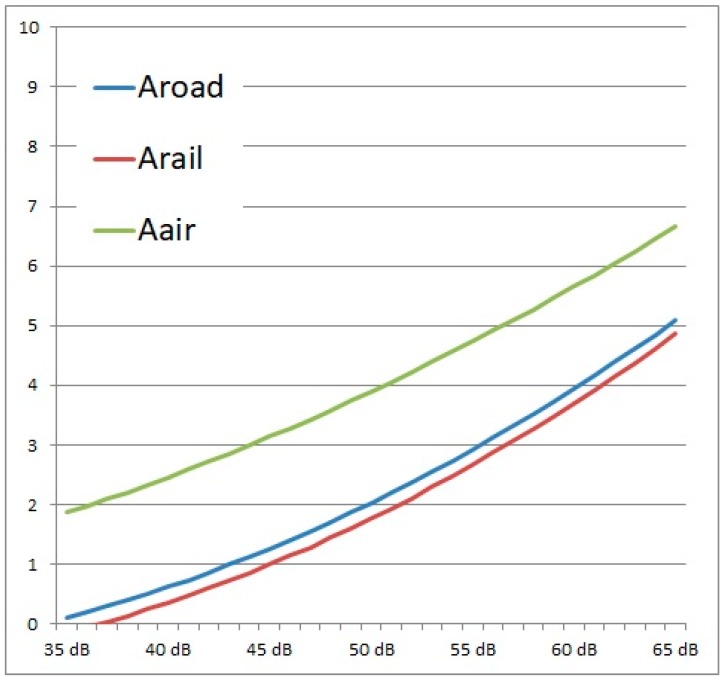
Fitting curves for the individual data points of the 11-point scale for annoyance by the three traffic noise sources.

Figure 5 shows the fitting curves for the individual data points of the 11-point scale for annoyance of all 1031 participants by road, rail and air traffic noise.

The chosen curve fits as a quadratic function providing a much better fit for the overall model compared to the linear approximation. In this point, the approach of Miedema’s model [30] deviates.

According to the exposure annoyance function for road, rail and air traffic noise, the expected annoyance scores were calculated by the following formulas

(3) $$A_{\mathrm{road}}^{\prime }=\;-0.08498\times L_{\mathrm{road}}+0.00251\times L_{\mathrm{road}}^{2}$$

(4) $$A_{\mathrm{rail}}^{\prime }=\;-0.09641\times L_{\mathrm{rail}}+0.00263\times L_{\mathrm{rail}}^{2}$$

(5) $$A_{\mathrm{air}}^{\prime }=\;-0.00424\times L_{\mathrm{air}}+0.00164\times L_{\mathrm{air}}^{2}$$

Annoyance results below 0 are set to 0. The results are checked against the given annoyance scores of the survey. The predicted annoyances scores A’_rail_ and A’_air_ were subsequently converted to L_road_, by solving the quadratic equation for A’_road_, the substitution levels L’_rail_ and L’_air_ are obtained.

(6) $$L_{\mathrm{rail}}^{\prime }=\frac{0.08498+\sqrt{0.08498^{2}+4\times 0.00251\times A_{\mathrm{rail}}^{\prime }}}{2*0.00251}$$

(7) $$L_{\mathrm{air}}^{\prime }=\frac{0.08498+\sqrt{0.08498^{2}+4\times 0.00251\times A_{\mathrm{air}}^{\prime }}}{2\times 0.00251}$$

The substitution levels for rail traffic noise L’_rail_ and air traffic noise L’_air_ are added up with the level of road traffic noise L_road_ to receive the substitution level for the total noise L’_total_ by the following formula:

(8) Ltotal′=10×log(10Lroad10+10Lrail′10+10Lair′10)

Starting from L’_total_ the value for A’_total_ is obtained by solving the quadratic equation for A_road_. From this substitution level L’_total_, the score for the total noise annoyance can be estimated according to the quadratic formula for road traffic noise.

(9) $$A_{\mathrm{total}}^{\prime }=\;-0.08498\times L_{\mathrm{total}}^{\prime }+0.00251\times L_{\mathrm{total}}^{\prime 2}$$

This annoyance score A’_total_ calculated from the substitution level of all noise sources was compared with the survey result A’_total_. This correlation was also done for each single source, road, and rail and air traffic by checking the observed annoyance scores and the predicted scores. Table 12 shows the results of the nonparametric tests as follows.

While the dominant source model [25] scores a correlation coefficient of 0.112 the annoyance equivalents model scores 0.298. For single source the correlation coefficient is considerably higher: 0.463 for road traffic noise, 0.499 for rail traffic noise and 0.398 for air traffic noise.

## 4. Discussion

### 4.1. Covariates in the Model

Purposeful selection of covariates [48] in the logistic regression was helpful in identifying variables that, by themselves, were not significantly related to the outcome but made an important contribution in the presence of other variables. The univariate correlations for total noise annoyance, as given in Table 5, showed different results for total noise annoyance for highly annoyed and annoyed. While the dichotomized results for highly annoyed showed significant influence for the noise index for road and air, but not for rail traffic noise, the results for annoyed showed significance for all three noise sources. The reason could be the small number of highly annoyed from rail traffic noise. The significant results for annoyed participants influenced by all three traffic noise sources is an indicator that an annoyance equivalents model is appropriate to describe total noise annoyance by multiple sources. The typical epidemiological covariates, such as age (age squared) and gender, were not significant. In both categories, highly annoyed and annoyed, the self-reported estimation of noise sensitivity, access to a quiet facade and installation of special noise insulation (noise protection windows) were significant. While the self-reported noise sensitivity but also the access to a quiet facade had an increasing effect on noise annoyance, the sponsored installation of noise protection windows decreased the noise annoyance. The influence of self-reported health status, education, the fact of owning a residence and the type of dwelling were not significant. Only in the category annoyance could a significant correlation between numbers of children in the household be shown.

As shown in Table 8, all traffic noise sources positively and significantly increased the overall noise annoyance score. This was also the case for all other annoyances caused by not modelled sources, except annoyance from craft and industry noise and noise caused by passers-by and pubs. This can be explained by the special local character of these sources. Another explanation could be the character of industrial sounds which contain tonal or impulsive components or are dominated by low frequencies. Noise from passengers and pubs, as well from the neighborhood characterized by informative sound, also significantly contribute to the total noise annoyance score. Access to a quiet facade and noise protection windows installed within the last 10 years were not significant variables. Clearly, age, gender and educational level were not significant.

As shown in Table 6, no significant influence of road traffic background noise was found according to the annoyance of rail and air traffic noise. In both categories of highly annoyed and annoyed, the self-reported noise sensitivity had a significant influence.

The binary logistic regression with the included interaction terms did not show usable results. Except for the subjectively reported noise sensitivity with a strengthening effect on annoyance, there were no significant contributions to either the annoyed category or the highly annoyed category. This was found at the level of the individual sources with the interaction terms for all source combinations. One reason may be an oversaturated model. In accordance to this finding, the annoyance equivalents model was set up not by the dichotomous annoyance reaction annoyed and highly annoyed, but by the annoyance response of the 11-point scale itself. A linear regression of all contributing single annoyance reactions showed significant effects for each source, except noise from craft and industry, passers-by, and pubs. This finding was used as the basis for the total annoyance model for traffic noise.

### 4.2. Comparison of the Exposure Response Relations with Other Study Results

**Figure 6:**
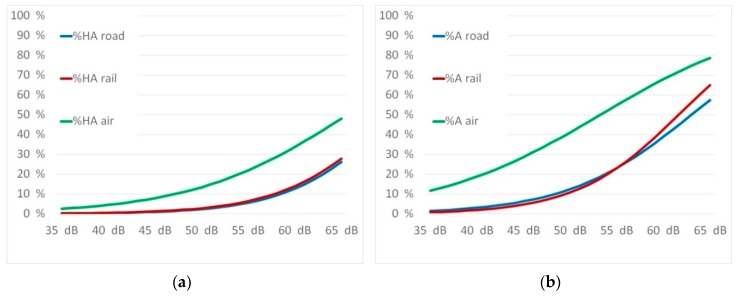
Comparison of exposure response relationship for percentage highly annoyed (**a**) and percentage annoyed (**b**).

A comparison overview of the exposure response curves for the percentage of annoyed and highly annoyed for all three traffic noise sources is given in Figure 6.

The curves of both highly annoyed as well as annoyed show a similar picture. While annoyance from road and rail traffic is approximately the same, air traffic causes significantly higher reactions. This is completely in line with the results of recent studies as discussed below.

**Figure 7:**
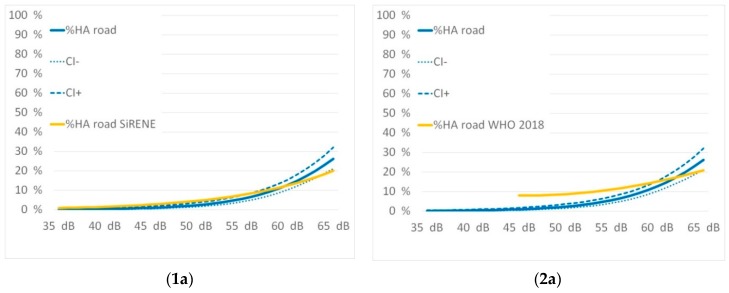

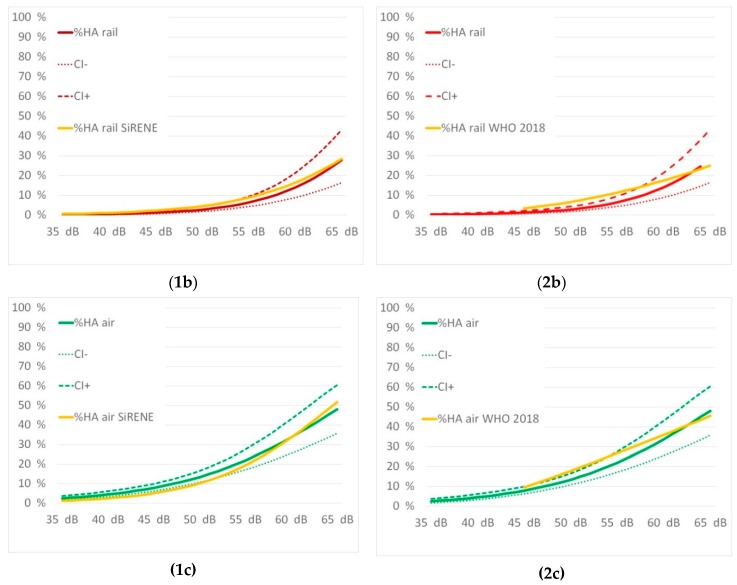
Comparison of the exposure-response curves with SiRENE 2019 for road (**1a**), rail (**1b**) and air traffic noise (**1c**) and WHO 2018 for road (**2a**), rail (**2b**), and air traffic noise (**2c**).

The exposure response relations were assessed versus the recently published results of the SiRENE study from Switzerland [12] and the WHO recommended curves adapted from Guski´s and colleagues metaanalysis [26] as published in the 2018 guidelines [50]. Both studies considered recent data and had similar methods describing the exposure. Figure 7 compares their results with the ones from Innsbruck:

Figure 6 shows that there is a rather good fit of the exposure-response curves for the percentage highly annoyed (%HA) for each of the traffic noise sources. The SiRENE curves are within the confidence intervals of the Innsbruck curves in almost the entire level range. Thus, the slope of road traffic noise annoyance in Innsbruck is steeper indicating a percentage of highly annoyed of 26% at 65 dB L_den_ while SiRENE indicates 20%. This could be a result of the different settings. While SiRENE has pooled data from urban and rural areas, the Innsbruck study relies only on an urban data set. The rail and air traffic noise response curves fit very well together.

The WHO-curves do not fit our data as well as the data of SiRENE do. The slopes of the WHO curves are obviously flatter, and this causes an important effect: The WHO derives its recommendations on thresholds for adverse health effects from an absolute risk for highly annoyed of 10%. This occurs for road traffic noise at L_den_ of 53 dB according to the WHO curves, but not before 59 dB according to the Innsbruck curves. The curves obtained provide the opportunity to set appropriate thresholds or limits and to take greater account of local specificities. The results are also in contradiction to former studies in the alpine space [11] where much stronger noise annoyance reactions versus road and rail traffic noise were observed. It seems that the recent results, which are in line with the SiRENE results, bring new valid information for alpine space too.

**Figure 8:**
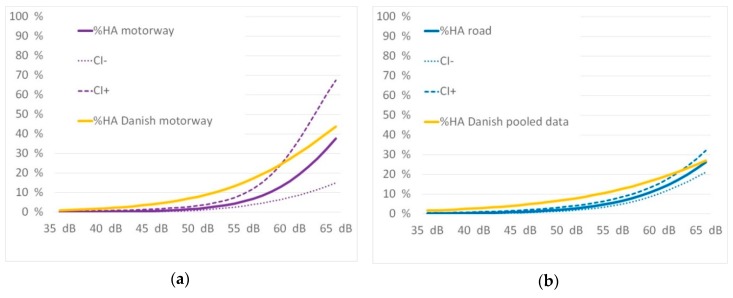
Comparison of the exposure-response curves with Danish road study [10] for motorway (**a**) and pooled road data (**b**).

The Danish road study [10] also elaborated exposure response relations according to annoyance and different road types. Motorway noise was compared to urban road noise. The authors were contacted to share their parameters for the pooled data for motorway and urban roads. The exposure response relations were assessed versus the results of the Danish road study for the motorway and the urban road situations. This is shown in Figure 8 as follows.

The curves from the Danish road study are a better fit to the WHO [50] curves. The percentage of highly annoyed in Innsbruck is much lower as shown in the lower exposure levels. At higher levels of approximately 65 dB the curves meet. In both situations it is shown that motorway noise causes a higher percentage of annoyance than the pooled data.

The European Commission recommended the use of the exposure- response curves of Miedema and Oudshoorn [8] to be used for noise assessments and the formulation of limit values in the member states of the EC. 

**Figure 9:**
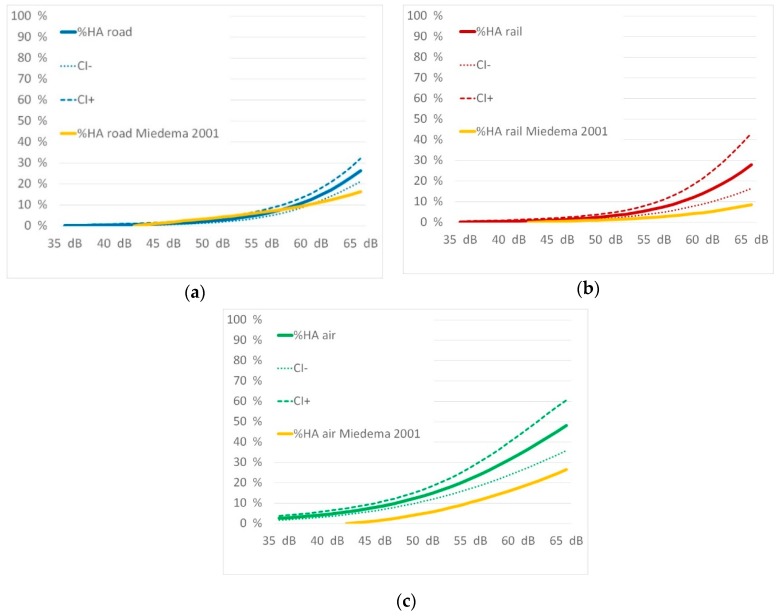
Comparison of the exposure-response curves from Innsbruck with the Miedema curves [8] for road (**a**), rail (**b**), and air traffic noise (**c**).

Figure 9 shows exposure–response curves from Innsbruck versus the Miedema curves [8].

The comparisons of rail and air traffic noise show that Miedema´s curves [8] are underestimating the annoyance effects in Innsbruck. The comparison of road traffic noise evidences a point of intersection between 58 to 59 dB L_den_. This is corresponding to a percentage of 10% highly annoyed. Up to these noise levels the former derivation for limiting values for road traffic noise is still appropriate, for rail and air traffic noise an amendment seems to be necessary.

### 4.3. Total Annoyance Assessment Model for Traffic Noise

A Total Annoyance Assessment Model for legal application must be suitable for all types of source-combinations but also for single sources. The target is to define a single value that can be checked against one common limiting value. This limiting value should express the same probability of annoyance independently of the source or source-combination. This means that if only one source has a relevant impact on a receiver the annoyance should be appropriately described by the used noise index. As shown in Figure 5 there is a difference of 2 points in the 11-point annoyance scale between air traffic noise and road traffic noise respectively rail traffic noise. Obviously, a singular physically model where the noise levels are summed up logarithmically does not meet the requirements for every single source assessment. As the physical noise level gathered for aircraft noise would lead to the same annoyance score than for the two other sources the approach of physical summation was not further investigated. There are different approaches and findings in the published literature regarding the assessment of which model best fits to the annoyance response to noise impact of multiple sources. While Miedema introduced the annoyance equivalents model [30], other researchers [28,29] had better fits for the dominant source model. In a French study [29] the combined effect analyses started at 55 dB L_den_ because this was the lowest exposition value for rail traffic. In Innsbruck we rated inhabitants with such an exposition already as strongly affected. Within the overlap between 55 and 65 dB the results are comparable. As a result, Gille and colleagues stated that in general perceptual models facilitate a better calculation of total annoyance than psychophysical models. This result is approved by the study in Innsbruck.

Wothge and colleagues found a plausible reason for this by explaining the response on air traffic noise was so high due to political circumstances. Their study took place in the context of an environmental impact assessment for a major change in Frankfurt Airport. Between 2011 and 2013, residents around Frankfurt airport were surveyed three times in a panel-study. The main focus of the panel-study was to investigate the impact of aircraft noise on the noise annoyance and quality of life of residents before (2011) and after (2012, 2013) the opening of a new landing runway at Frankfurt airport (called “North West”) in October 2011.

Our study in Innsbruck had no such background for any of the noise sources. There were no running or planned projects in any of the modes of transport which could have had an influence on the judgment of the 1031 participants. In addition, the clusters were selected by the three zones of highly-, medium-, and little-affected areas for the three sources. It seems that this method produces valid results. Spearman´s Rho for the annoyance equivalents model was predicted with 0.298 (*p* value 0.000 is significant at the 0.01 level 2-tailed). In comparison to this, the dominant source model shows a much lower correlation with 0.112. As was shown, there are more sources than only the traffic noise sources that cause the total annoyance. Construction noise, neighborhood noise and noise from passers-by and pubs in open spaces are the most relevant of them. Therefore, it is understandable, that the correlation is not as high compared to the annoyance reaction to a single noise source. However, with Sperman´s Rho of 0.298, it is still within a well-known range of correlation in noise impact research [51]. This model can therefore be recommended for useage in overall noise assessment and is especially to be considered in regulatory approval procedures where an overall noise assessment is required.

### 4.4. Strengths and Limitations

A limiting factor, as with many similar studies, is the determination of the exposure according to the level of the most exposed facade. Thus, the real impact situation is not representative for people who have their living and/or bedrooms towards quiet facades. The study shows the exposure and response situation in an urban area. A transfer of the results into the setting of rural areas should only be performed with care.

One strength of this study was the initial design to evaluate the combined effects of multiple sources. The groups of exposed people to road, rail and air traffic noise and the combinations of it are nearly equally distributed. Another strength is the use of personal interviews as the survey instrument; and the overall response rate of 47.8%, which is to be considered as very high. These facts increase the representativeness and validity of our results.

Other strengths include the rating of the exposure by state-of-the-art sound propagation methods, taking all flight movements, all streets down to access roads, and each train passing by into consideration and the modelling of noise exposure on each building. These were performed prior to the survey. Applying these noise clusters, the participants for the survey were selected. The sound propagation methods are valid in Austria for environmental impact assessment and represent the state-of-the art engineering technology. No accuracy was lost by classification to noise bands.

Due to the high response rate and the stratified selection of 1031 participants, the selection bias is rated to be low. 

A limitation of this study is the annual and daily noise conditions at the airport. The main air traffic takes place in wintertime, caused by winter tourism in the State of Tyrol. This circumstance was taken into account by setting the survey period outside the main traffic period. The survey took place in May and June when the winter charter air traffic was finished but was still remembered by the participants. Brink and colleagues [52] evidenced that questionnaires filled out in autumn were associated with a significantly higher annoyance rating than in springtime. Average annoyance responses in their autumnal assessment exalted by the same amount as a 1.7 dB increase in L_dn_. Compared to the difference in the average of winter charter exposure and the yearly average of aircraft noise consisting in an L_den_ of 1.5 dB this could be compensated in Innsbruck. By choosing the survey season in early summer/late spring a valid mean score could be achieved.

## 5. Conclusions

In Austria the general approach for rating noise is the assessment of annoyance after the change of actual local conditions. These local conditions are usually described by road traffic noise. The higher the noise level of a road, the higher is the allowable additional level of a specific noise source. As shown in Section 3.4, no significant influence of road traffic noise on the annoyance to rail and air traffic noise was found. No protective effect by road traffic background noise could be shown for rail and air traffic noise. The recent noise regulations for rail and air traffic noise do not take background noise of road traffic in account. For other noise sources, especially industrial noise, the traditional Austrian legal approach is the assessment of specific noise by background noise. Further research is required to provide evidence on effectiveness of this approach.

An assessment method separating noise source by noise source to arbitrate annoyance is not valid enough. As shown in this study with 1031 participants in Innsbruck each additional noise source causes an increased effect on the total noise annoyance. For overall noise protection, a comprehensive approach is needed. The study evaluated an annoyance equivalents model [30] based on the elaborated exposure response function in the city of Innsbruck.

A benefit of a total noise assessment is the possibility to combine all known sources by exposure response curves. By adjusting a single source, for which road traffic noise is strongly recommended, all sources and source combinations can be rated by a single assessment method. Threshold values can be set for all single sources using a substitution level which results in the same annoyance score as road traffic noise and combinations of source combined by energetic addition as already introduced for wind turbine noise [53]. Further noise sources in addition to traffic noise sources need to be investigated.

## Figures and Tables

**Table 1:** Distribution of the exposure classes and the address points within.

		Air Traffic Noise			Rail Traffic Noise			Road Traffic Noise		
	L_den_ (dB)	>55	45–55	<45	>55	45–55	<45	>55	45–55	<45
Air traffic noise	>55	643	0	0	19	90	534	281	362	0
	45–55	0	4012	0	367	1213	2432	2139	1768	105
	<45	0	0	5056	470	1769	2817	2353	2018	685
Rail traffic noise	>55	19	367	470	856	0	0	653	203	0
	45–55	90	1213	1769	0	3072	0	1898	1118	56
	<45	534	2432	2817	0	0	5783	2222	2827	734
Road traffic noise	>55	281	2139	2353	653	1898	2222	4773	0	0
	45–55	362	1768	2018	203	1118	2827	0	4148	0
	<45	0	105	685	0	56	734	0	0	790

**Table 2:** Reliability test results on items.

Reliability Statistics	No. of Items	Cronbachs Alpha	Rating
Exposure to noise	10	0.758	very good
Annoyance	10	0.756	very good
Sleep disturbance	10	0.777	very good
Noise mitigation measures	9	0.882	excellent

**Table 3:** Overview of the descriptive statistics for the main parameters of the survey.

		Frequency	Percent	Valid Percent
Gender	female	541	52.5	52.5
	male	490	47.5	47.5
	total	1031	100.0	100.0
Age group	18 to 40 years	440	42.7	42.7
	41 to 60 years	316	30.6	30.6
	over 60 yeas	275	26.7	26.7
	total	1031	100.0	100.0
Type of housing	solitary house	142	13.8	13.8
	semi-detached house	48	4.7	4.7
	terraced house	58	5.6	5.6
	housing complex < 10 flats	412	40.0	40.0
	housing complex 10+ flats	363	35.2	35.2
	others	8	0.8	0.8
	total	1031	100.0	100.0
Protection against traffic noise given by screening	not at all	469	45.5	45.5
	yes, a little	297	28.8	28.8
	yes, the bigger part	209	20.3	20.3
	yes totally	27	2.6	2.6
	n.a.	29	2.8	2.8
	total	1031	100.0	100.0
Access to a quiet facade	yes	701	68.0	69.5
	no	308	29.9	30.5
	total	1009	97.9	100.0
Missing	n.a.	22	2.1	
Total	total	1031	100.0	
Noise protection windows implemented within the last 10 years	yes	187	18.1	18.1
	no	672	65.2	65.2
	n.a.	172	16.7	16.7
	total	1031	100.0	100.0

**Table 4:** Response rates in the noise clusters of strong, medium, and little effected address points.

Noise Cluster	Interviews Completed	Not Encountered	No Interest	Abandoned	Sum of Attempts	Sum of Encountered Contacts	Response of Encountered Contacts in %
Air strong	111	963	184	4	1262	299	37%
Air medium	111	491	118	2	722	231	48%
Air little	118	528	131	2	779	251	47%
Rail strong	111	737	121	2	971	234	47%
Rail medium	114	512	96	3	725	213	54%
Rail little	118	843	126	2	1089	246	48%
Road strong	118	743	138	1	1000	257	46%
Road medium	119	692	135	5	951	259	46%
Road little	111	375	52	4	542	167	66%
Total	1031	5884	1101	25	8041	2157	47.8%

Note: strong = L_den_ > 55 dB; medium = L_den_ 45–55 dB, little = L_den_ < 45 dB.

**Table 5:** Research question translated into hypothesis.

Research Question	(1)	(2)	(3)
H_1_	*β*_4_ ≠ 0	*β*_5_ ≠ 0	T_effect_ > 0
H_0_	*β*_4_ = 0	*β*_5_ = 0	T_effect_ ≤ 0

**Table 6:** Univariate correlation to total noise annoyance for annoyed and highly annoyed.

	Range		A_dich,HA_		A_dich,A_	
Covariate			Chi-Q.	*p*	Chi-Q.	*p*
Number	1	1031				
L_road_	24.9	72.0	37.157	0.000	80.247	0.000
L_rail_	20.7	69.0	2.562	0.109	14.692	0.000
L_air_	30.5	66.4	5.923	0.015	22.750	0.000
Gender	0	1	1.068	0.301	2.398	0.122
Age	18	97	2.183	0.140	0.146	0.703
Age^2^	-	-	1.231	0.267	0.034	0.853
Noise sensitivity	0	10	50.414	0.000	64.185	0.000
Personal health status	0	10	0.005	0.943	0.080	0.777
Educational level	1	5	0.007	0.933	0.049	0.824
Living here since (years)	1	86	7.646	0.006	3.058	0.080
Principal residence	0	1	0.000	-	0.000	-
Ownership	1	3	1.630	0.202	0.595	0.440
Access to quiet facade	1	2	4.593	0.032	5.624	0.018
Noise protection given	1	3	18.105	0.000	19.567	0.000
Type of dwelling	1	6	0.106	0.745	1.775	0.183
Number of children in the household	1	5	1.971	0.160	4.605	0.032

Note: A_dich,HA_ = percentage of highly annoyed by noise of multiple sources; A_dich,A_ = percentage of annoyed by noise of multiple sources; Chi-Q. = Chi-square; *p* = significance level.

**Table 7:** Binary logistic regression due to the influence of road traffic background noise.

		B	SE	Wald	df	*p*	Exp (B)
A_dich,HA,rail_	L_rail_	0.151	0.051	8.777	1	0.003	1.163
	L_rail_ by L_road_	0.000	0.001	0.395	1	0.530	1.000
	Sensitivity	0.151	0.072	4.385	1	0.036	1.163
	Access to quiet facade	0.900	0.410	4.828	1	0.028	2.459
	Noise protection given	0.082	0.308	0.072	1	0.789	1.086
	Living here since	−0.033	0.016	4.476	1	0.034	0.968
	Constant	−13.779	1.896	52.791	1	0.000	0.000
A_dich,A,rail_	L_rail_	0.152	0.032	22.451	1	0.000	1.164
	L_rail_ by L_road_	0.000	0.000	1.320	1	0.251	1.000
	Sensitivity	0.162	0.044	13.352	1	0.000	1.176
	Access to quiet facade	0.692	0.261	7.024	1	0.008	1.998
	Noise protection given	−0.464	0.205	5.105	1	0.024	0.629
	Living here since	−0.014	0.008	3.417	1	0.065	0.986
	Constant	−11.482	1.139	101.609	1	0.000	0.000
A_dich,HA,air_	L_air_	0.093	0.025	13.309	1	0.000	1.097
	L_air_ by L_road_	0.000	0.000	1.830	1	0.176	1.000
	Sensitivity	0.229	0.041	30.875	1	0.000	1.257
	Access to quiet facade	0.870	0.221	15.486	1	0.000	2.387
	Noise protection given	−0.231	0.191	1.460	1	0.227	0.794
	Living here since	0.011	0.006	3.861	1	0.049	1.011
	Constant	−9.774	1.012	93.205	1	0.000	.000
A_dich,A,air_	L_air_	0.087	0.020	19.601	1	0.000	1.090
	L_air_ by L_road_	0.000	0.000	2.489	1	0.115	1.000
	Sensitivity	0.185	0.028	43.042	1	0.000	1.203
	Access to quiet facade	0.296	0.164	3.255	1	0.071	1.344
	Noise protection given	−0.222	0.134	2.753	1	0.097	0.801
	Living here since	0.009	0.004	4.735	1	0.030	1.009
	Constant	−6.738	0.700	92.616	1	0.000	0.001

Note: B = regression coefficient; SE = standard error; Wald = Wald Chi-square; df = degrees of freedom; *p* = significance level; A_dich,HA,rail_ = percentage of highly annoyed by rail traffic noise; A_dich,A,rail_ = percentage of annoyed by rail traffic noise; A_dich,HA,air_ = percentage of highly annoyed by air traffic noise; A_dich,A,air_ = percentage of annoyed by air traffic noise.

**Table 8:** Binary logistic regression for the generalized model with all interaction terms for the three noise sources.

		B	SE	Wald	df	*p*	Exp (B)
A_dich,HA_	L_road_	0.857	0.845	1.030	1	0.310	2.357
	L_rail_	0.639	1.112	0.330	1	0.566	1.894
	L_air_	0.399	1.085	0.135	1	0.713	1.490
	L_rail_ by L_road_	−0.014	0.019	0.501	1	0.479	0.986
	L_rail_ by L_air_	−0.004	0.025	0.028	1	0.867	0.996
	L_air_ by L_road_	−0.009	0.019	0.245	1	0.620	0.991
	L_air_ by L_rail_ by L_road_	0.000	0.000	0.089	1	0.765	1.000
	Sensitivity	0.278	0.040	47.366	1	0.000	1.321
	Access to quiet facade	0.583	0.219	7.110	1	0.008	1.792
	Noise protection given	−0.392	0.183	4.605	1	0.032	0.676
	Living here since	0.010	0.005	3.359	1	0.067	1.010
	Constant	−46.579	48.465	0.924	1	0.337	0.000
A_dich,A,_	L_road_	0.857	0.845	1.030	1	0.310	2.357
	L_rail_	0.639	1.112	0.330	1	0.566	1.894
	L_air_	0.399	1.085	0.135	1	0.713	1.490
	L_rail_ by L_road_	−0.014	0.019	0.501	1	0.479	0.986
	L_rail_ by L_air_	−0.004	0.025	0.028	1	0.867	0.996
	L_air_ by L_road_	−0.009	0.019	0.245	1	0.620	0.991
	L_air_ by L_rail_ by L_road_	0.000	0.000	0.089	1	0.765	1.000
	Sensitivity	0.278	0.040	47.366	1	0.000	1.321
	Access to quiet facade	0.583	0.219	7.110	1	0.008	1.792
	Noise protection given	−0.392	0.183	4.605	1	0.032	.676
	Living here since	0.010	0.005	3.359	1	0.067	1.010
	Constant	−46.579	48.465	0.924	1	0.337	0.000

Note: B = regression coefficient; SE = standard error; Wald = Wald Chi-square; df = degrees of freedom; *p* = significance level; A_dich,HA_ = percentage of highly annoyed by noise of multiple sources; A_dich,A_ = percentage of annoyed by noise of multiple sources.

**Table 9:** Results of the linear regression analyses referred to the score of the total noise annoyance.

Dependent Variable: Total Noise Annoyance	Unstandardized Coefficients		Stand.	T	*p*	CI	
						Lower	Upper
	B	SE	B			Bound	Bound
Constant	1.311	0.459		2.859	0.004	0.411	2.211
Road traffic noise annoyance	0.326	0.025	0.341	12.959	0.000	0.276	0.375
Rail traffic noise annoyance	0.129	0.030	0.101	4.325	0.000	0.071	0.188
Air traffic noise annoyance	0.171	0.023	0.177	7.456	0.000	0.126	0.217
Craft and industry noise annoyance	−0.017	0.039	−0.010	−0.435	0.664	−0.095	0.060
Passer-by and pub noise annoyance	0.057	0.028	0.051	1.993	0.047	0.001	0.112
Construction noise annoyance	0.160	0.023	0.174	7.104	0.000	0.116	0.204
Neighborhood noise annoyance	0.194	0.027	0.174	7.194	0.000	0.141	0.247
Vibration annoyance	0.075	0.033	0.059	2.303	0.022	0.011	0.140
Self-reported noise sensitivity	0.135	0.023	0.132	5.845	0.000	0.090	0.180
Access to quiet facade	0.101	0.130	0.017	0.776	0.438	−0.154	0.356
Noise protection window	−0.184	0.107	−0.039	−1.725	0.085	−0.394	0.025
Number of children in household	−0.123	0.067	−0.040	−1.831	0.067	−0.254	0.009
Highest educational level	−0.051	0.045	−0.025	−1.143	0.253	−0.140	0.037
Age	−0.002	0.003	−0.011	−0.476	0.634	−0.009	0.005
Gender	−0.039	0.119	−0.007	−0.325	0.746	−0.273	0.195

Note: B = regression coefficient; stand. = standardized coefficient; SE = standard error; T = *T*-test; *p* = significance level; CI = 95% confidence interval.

**Table 10:** Results expressed as R^2^ for different curve fit models.

Model	Road	Rail	Air
A = *β*_1_ × L	0.767	0.532	0.773
A = β_0_ + *β*_1_ × L	0.461	0.505	0.394
A = *β*_1_ × L + *β*_2_ × L^2^	0.797	0.656	0.785
A = *β*_0_ + *β*_1_ × L + *β*_2_ × L^2^	0.465	0.527	0.396
A = *β*_1_ × L + *β*_2_ × L^2^ + *β*_3_ × L^3^	0.797	0.657	0.788

**Table 11:** Regression coefficients for the quadratic curve fit model.

	*β*_1_	Wald-Chi-Quadrat	*p*	*β*_2_	Wald-Chi-Quadrat	*p*
Road	−0.08498	56.800	0.000	0.00251	148.719	0.000
Rail	−0.09641	122.113	0.000	0.00263	170.951	0.000
Air	−0.00424	0.117	0.732	0.00164	36.223	0.000

**Table 12:** Correlation coefficients for different models and single sources.

Model	Spearman-Rho
dominant source model	0.112
annoyance equivalents model	0.298
single source road	0.463
single source rail	0.499
single source air	0.398

Note: All *p*-values < 0.001.

## Supplementary Materials

The following are available online at https://www.mdpi.com/1660-4601/16/18/3504/s1, Figure S1: Noise map L_den_ for road traffic noise, Figure S2: Noise map L_den_ for rail traffic noise, Figure S3: Noise map L_den_ for air traffic noise, Figure S4: Noise map L_den_ for motorway noise. File S1: Total noise investigation Innsbruck full report.

## References

[1] Berglund B., Lindvall T., Schwela D.H., WHO (1999). Guidelines for Community Noise.

[2] Muzet A. (2007). Environmental noise, sleep and health. Sleep Med. Rev..

[3] Zacarías F.F., Molina R.H., Ancela J.L.C., López S.L., Ojembarrena A.A. (2013). Noise exposure in preterm infants treated with respiratory support using neonatal helmets. Acta Acust. united with Acust..

[4] Chetoni M., Ascari E., Bianco F., Fredianelli L., Licitra G., Cori L. (2016). Global noise score indicator for classroom evaluation of acoustic performances in LIFE GIOCONDA project. Noise Mapp..

[5] Minichilli F., Gorini F., Ascari E., Bianchi F., Coi A., Fredianelli L., Licitra G., Manzoli F., Mezzasalma L., Cori L. (2018). Annoyance judgment and measurements of environmental noise: A focus on italian secondary schools. Int. J. Environ. Res. Public Health.

[6] Dratva J., Phuleria H.C., Foraster M., Gaspoz J.M., Keidel D., Künzli N., Sally Liu L.J., Pons M., Zemp E., Gerbase M.W. (2012). Transportation noise and blood pressure in a population-based sample of adults. Environ. Health Perspect..

[7] Babisch W., Beule B., Schust M., Kersten N., Ising H. (2005). Traffic noise and risk of myocardial infarction. Epidemiology.

[8] Miedema H.M.E., Oudshoorn C.G.M. (2001). Annoyance from transportation noise: Relationships with exposure metrics DNL and DENL and their confidence intervals. Environ. Health Perspect..

[9] Guski R., Schreckenberg D. (2015). Verkehrslärmwirkungen im Flughafenumfeld, Endbericht, Band 3: Wirkungen von Verkehrslärm auf die Belästigung und Lebensqualität.

[10] Fryd J., Pedersen T.H. Noise annoyance from urban roads and motorways. Proceedings of the Internoise 2016.

[11] Lercher P., de Greve B., Botteldooren D., Rüdisser J. A comparison of regional noise-annoyance-curves in alpine areas with the European standard curves. Proceedings of the 9th Congress of the International Commission on the Biological Effects of Noise (ICBEN 2008).

[12] Brink M., Schäffer B., Vienneau D., Foraster M., Pieren R., Eze I.C., Cajochen C., Probst-Hensch N., Röösli M., Wunderli J.M. (2019). A survey on exposure-response relationships for road, rail, and aircraft noise annoyance: Differences between continuous and intermittent noise. Environ. Int..

[13] Licitra G., Ascari E., Fredianelli L. (2017). Prioritizing Process in Action Plans: A Review of Approaches. Curr. Pollut. Rep..

[14] WHO (2011). Burden of Disease from Environmental Noise: Quantification of Healthy Life Years Lost in Europe.

[15] Lechner C., Schnaiter D. (2018). Gesamtlärmbetrachtung [Total Noise Investigation] Innsbruck 2017.

[16] Ruiz-Padillo A., Ruiz D., Torija A., Ramos-Ridao Á. (2016). Selection of suitable alternatives to reduce the environmental impact of road traffic noise using a fuzzy multi-criteria decision model. Environ. Impact Assess. Rev..

[17] Licitra G., Fredianelli L., Petri D., Vigotti M.A. (2016). Annoyance evaluation due to overall railway noise and vibration in Pisa urban areas. Sci. Total Environ..

[18] Bunn F., Zannin P. (2016). Assessment of railway noise in an urban setting. Appl. Acoust..

[19] Licitra G., Gagliardi P., Fredianelli L., Simonetti D. (2014). Noise mitigation action plan of Pisa civil and military airport and its effects on people exposure. Appl. Acoust..

[20] Flores R., Gagliardi P., Asensio C., Licitra G. (2017). A Case Study of the Influence of Urban Morphology on Aircraft Noise. Acoust. Aust..

[21] Iglesias-Merchan C., Diaz-Balteiro L., Soliño M. (2015). Transportation planning and quiet natural areas preservation: Aircraft overflights noise assessment in a National Park. Transp. Res. Part D Transp. Environ..

[22] Bernardini M., Fredianelli L., Fidecaro F., Gagliardi P., Nastasi M., Licitra G. (2019). Noise Assessment of Small Vessels for Action Planning in Canal Cities. Environments.

[23] Casazza M., Boggia F., Serafino G., Severino V., Lega M. (2018). Environmental impact assessment of an urban port: Noise pollution survey in the port area of Napoli (S Italy). J. Environ. Account. Manag..

[24] BVwG (2014). Urteil [Judgment] Götzendorf GZ W 102 2000176-1/23E.

[25] Berglund B., Berglund U., Goldstein M., Lindvall T. (1981). Loudness (or annoyance) summation of combined community noises. J. Acoust. Soc. Am..

[26] Guski R., Schreckenberg D., Schuemer R. (2017). WHO Environmental Noise Guidelines for the European Region: A Systematic Review on Environmental Noise and Annoyance. Int. J. Environ. Res. Public Health.

[27] Guski R., Schreckenberg D. (2015). Verkehrslärmwirkungen im Flughafenumfeld (Bd. 7) [traffic noise effects in the airport environment Vol. 7]. Norah.

[28] Wothge J., Belke C., Möhler U., Guski R., Schreckenberg D. (2017). The combined effects of aircraft and road traffic noise and aircraft and railway noise on noise annoyance—an analysis in the context of the joint research initiative NORAH. Int. J. Environ. Res. Public Health.

[29] Gille L.-A., Cedex P., Marquis-favre C., Morel J. Annoyance due to combined railway and road traffic noise exposure: Testing of total annoyance models and dose-effect relationships for noise in isolation. Proceedings of the INTER-NOISE 2015-International Conference on Noise Control Engineering.

[30] Miedema H.M.E. (2004). Relationship between exposure to multiple noise sources and noise annoyance. J. Acoust. Soc. Am..

[31] Gille L.-A., Marquis-Favre C., Morel J. (2016). Testing of the European Union exposure-response relationships and annoyance equivalents model for annoyance due to transportation noises: The need of revised exposure-response relationships and annoyance equivalents model. Environ. Int..

[32] Ragettli M.S., Goudreau S., Plante C., Perron S., Fournier M., Smargiassi A. (2016). Annoyance from Road Traffic, Trains, Airplanes and from Total Environmental Noise Levels. Int. J. Environ. Res. Public Health.

[33] (2002). The European Parliament and the Council of the EU Directive 2002/49/EC of the European Parliament and of the Council relating to the assessment and management of environmental noise. Off. J. Eur. Communities.

[34] Baud S. (2017). Umweltbedingungen, Umweltverhalten 2015 [Environmental conditions, environmental behavior 2015].

[35] (2016). The European Parliament and the Council of the EU General Data Protection Regulation 2016. Off. J. Eur. Union.

[36] Ammon E., Behmann M. (2017). Demografische Entwicklung 2017 [Demografic Developement 2017].

[37] Bundes-LärmV (2006). Bundes-Umgebungslärmschutzverordnung BGBl. II 144/2006 [Federal Environmental Noise Ordinance BGBl. II 144/2006].

[38] Land Tirol Laserscan Tirol. https://www.tirol.gv.at/sicherheit/geoinformation/geodaten/laserscandaten/.

[39] Lechner C. (2010). ÖAL-Richtlinie Nr. 36 Blatt 2, Erstellung von Lärmkarten und Konfliktzonenplänen und Planung von Lärmminderungsmaßnahmen Anforderungen im Anwendungsbereich der Umgebungslärmrichtlinie 2002 / 49 / EG [Noise mapping and conflict zone plans].

[40] Schnaiter D. Evaluierungserhebung Neue Unterinntalbahn [Evaluation Survey New Lower Inn Valley Railway], ÖBB Infrastruktur AG, Executive Summary. https://www.brennernordzulauf.eu/infomaterial.html?file=files/mediathek/informationsmaterial/vertiefende_infos/Neue-Unterinntalbahn-Evaluierung.pdf.

[41] RVS 04.02.11 (2008). Lärm und Luftschadstoffe - Lärmschutz [Environmental Protection Noise and Airpollution Noise Control] RVS 04.02.11.

[42] ONR 305011 (2009). Berechnung der Schallimmission durch Schienenverkehr—Zugverkehr, Verschub- und Umschlagbetrieb [Determination of noise immission caused by rail traffic—Railway traffic, shunting and cargo handling operations, ONR 305011.

[43] ECAC (1997). ECAC No 29: Report on Standard Method of Computing Noise Contours around Civil Airports.

[44] Fields J.M., De Jong R.G., Gjestland T., Flindell I.H., Job R.F.S., Kurra S., Lercher P., Vallet M., Yano T., Guski R. (2001). Standardized general-purpose noise reaction questions for community noise surveys: Research and a recommendation. J. Sound Vib..

[45] ISO (2003). ISO/TS 15666 Acoustics—Assessment of Noise Annoyance by Means of Social and Socio-Acoustic Surveys.

[46] (2015). Statistik Austria Datenerhebung [data collection] EU-SILC. https://circabc.europa.eu/faces/jsp/extension/wai/navigation/container.jsp.

[47] Zimmer K., Ellermeier W. (1999). Psychometric properties of four measures of noise sensitivity: A comparison. J. Environ. Psychol..

[48] Bursac Z., Gauss C.H., Williams D.K., Hosmer D.W. (2008). Purposeful selection of variables in logistic regression. Source Code Biol. Med..

[49] VDI (2013). Verein Deutscher Ingenieure-Wirkung von Verkehrsgeräuschen - Kenngrößen beim Einwirken mehrerer Quellenarten [Effects of traffic noise Characteristic quantities in case of impact of multiple sources] VDI 3722 Blatt 2.

[50] World Health Organization (2018). Environmental Noise Guidelines for the European Region.

[51] Brink M. A review of explained variance in exposure-annoyance relationships in noise annoyance surveys. Proceedings of the 11th International Congress on Noise as a Public Health Problem (ICBEN).

[52] Brink M., Schreckenberg D., Vienneau D., Cajochen C., Wunderli J.M., Probst-Hensch N., Röösli M. (2016). Effects of scale, question location, order of response alternatives, and season on self-reported noise annoyance using icben scales: A field experiment. Int. J. Environ. Res. Public Health.

[53] Fredianelli L., Carpita S., Licitra G. (2019). A procedure for deriving wind turbine noise limits by taking into account annoyance. Sci. Total Environ..

