# Supplementary material for: Combined Effects of Aircraft, Rail, and Road Traffic Noise on Total Noise Annoyance—A Cross-Sectional Study in Innsbruck
Source: Int J Environ Res Public Health. 2019 Sep 19;16(18):3504. doi: 10.3390/ijerph16183504 (PMC6766025; doi:10.3390/ijerph16183504)
Supplement: Supplementary file 1 [file ijerph-16-03504-s001.zip › Gesamtlaermbetrachtung_Innsbruck_2017.pdf]

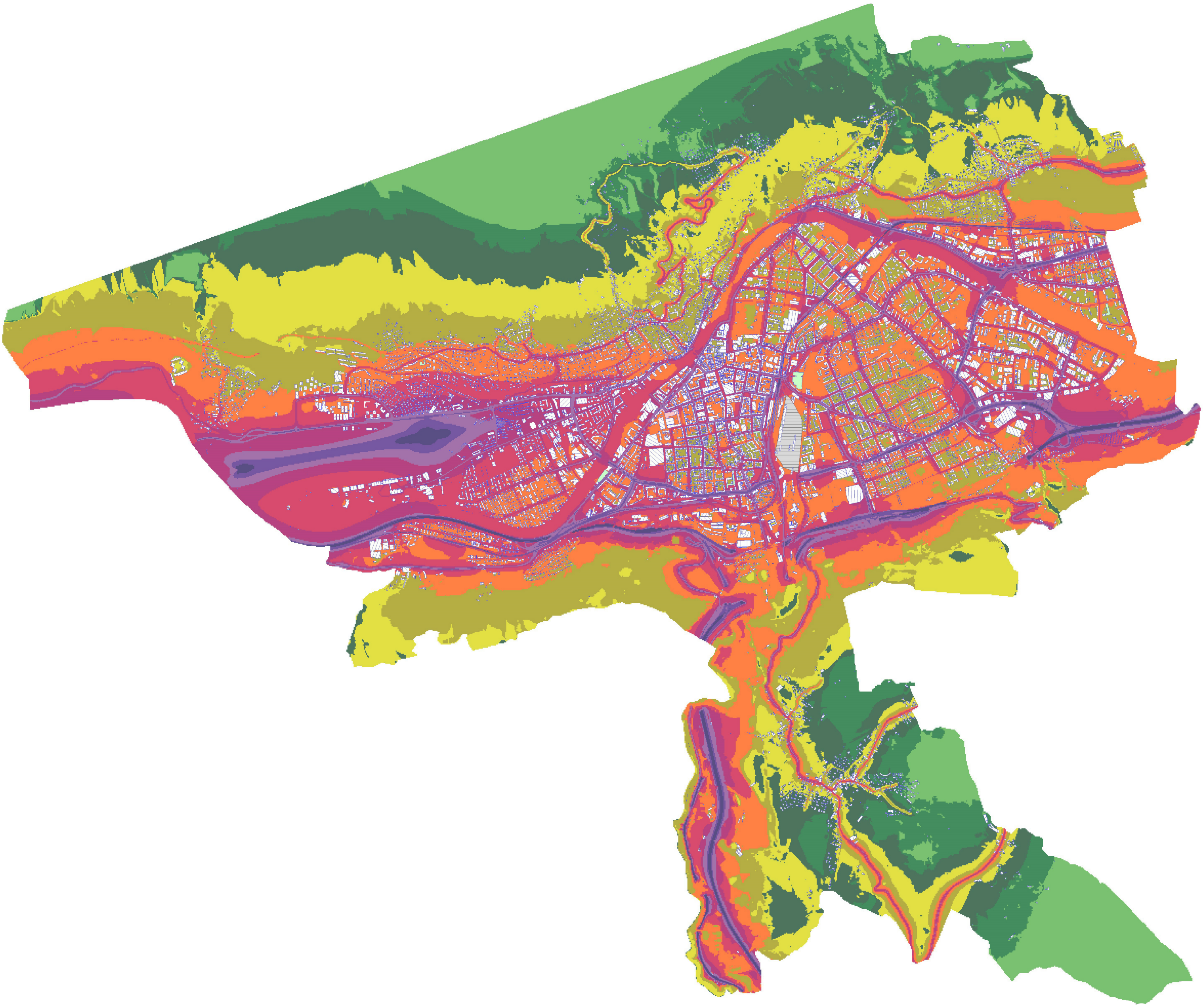

Gesamtlärmbetrachtung Innsbruck 2017

Pilotprojekt

tirol
Unser Land

Christoph Lechner / David Schnaiter

DIPL.-HTL-ING. MAG. CHRISTOPH LECHNER DR. DAVID SCHNAITER

Pilotprojekt

GESAMTLÄRMBETRACHTUNG INNSBRUCK

Gesamtbericht

Innsbruck, März 2018

Ein Projekt des **Landes Tirol** in Zusammenarbeit mit:
Stadt Innsbruck – Asfinag Alpenstrassen GmbH – Innsbruck Airport
Österreichsicher Arbeitsring für Lärmbekämpfung
Österreichische Bundesbahnen

Vorwort der Autoren

Der Durchführung des Pilotprojektes Gesamtlärmbetrachtung Innsbruck ging eine umfassende Literaturrecherche voraus, welche auf die kombinierten Effekte unterschiedlicher Schallquellen, insbesondere auf Verkehrsschallquellen, fokussierte. Zudem wurden von den Autoren rezente Studien über Belästigung (und subjektive Schlafstörung) durch Lärm berücksichtigt.

Das Studiendesign für das vorliegende Pilotprojekt wurde sorgfältig, nach guter wissenschaftlicher Praxis ausgewählt. Verzerrungen (sogenannte Bias) durch ungenaue Bestimmung der Exposition (measurement bias) sollten bestmöglich vermieden werden. Daher wurde nicht wie häufig beobachtet in Expositionsgruppen (z. B. von 50 – 55 dB), sondern punktgenau mit Dezibel-Auflösung gearbeitet, dies für jede einzelne betrachtete Quelle.

Mit dem Instrument der persönlichen Interviewbefragung wurde das in der sozialempirischen Forschung absolut valideste Werkzeug gewählt. Die geschichtete Clusterauswahl, der große Stichprobenumfang (N=1031) und die ausgezeichnete erreichte Rücklaufquote (47,8%) stellen sicher, dass nur minimalste Verzerrungen durch die Auswahl der Befragten (selection bias) auftreten. Die vorliegende Studie zeigt sich in der Aneinanderreihung der Methoden auch im internationalen Vergleich in ihrer Qualität einzigartig und ist damit auch für die anschließende Forschung und Beantwortung offener Fragen bestens geeignet.

Die Autoren danken allen Projektpartnern und ihren Vertreterinnen und Vertretern, den politisch Verantwortlichen, dem Befragungsinstitut IMAD und besonders der Innsbrucker Bevölkerung, die mit ihrer Bereitschaft zur Teilnahme zum Erkenntnisgewinn und Gelingen des Projektes beigetragen hat.

Dipl.-HTL-Ing. Mag. Christoph Lechner und Mag. Dr. David Schnaiter

Innsbruck, März 2018

Zentrale Inhalte und Ergebnisse

1. Einleitungsteil

Im **Einleitungsteil** dieses Gesamtberichts werden die Projektpartner (S. 34) vorgestellt und ein Überblick über die (sozio-)demographische Ausgangslage (S. 36) der Stadt Innsbruck mit ihren 9 Katastralgemeinden (S. 35) präsentiert.

2. Lärmdaten

Der zweite Teil **Lärmdaten** (S. 38) umfasst Erläuterungen zu den Grundbegriffen der methodischen Herangehensweise an die Lärmausbreitungsberechnungen (S. 38), sowie detaillierte Informationen zu den verwendeten Datengrundlagen (S. 40) und zum angewandten Verfahren einer Gesamtlärmberechnung (S. 47 und S. 66). Auch auf die Unterschiede zwischen Messung und Berechnung von Lärmdaten wird eingegangen (S. 40 und 45).

3. Lärm in Innsbruck

Die Beschreibung der Ist-Situation der Verkehrslärmbelastung in Innsbruck findet sich im dritten Teil **Lärm in Innsbruck** (S. 48):

3.1. Belastungszonen

- Der Straßenverkehr verursacht den mit Abstand größten Anteil an stark lärmbelasteten Wohnungen und Gebäuden in Innsbruck. **52% der Gebäude und 63% der Wohnungen in Innsbruck sind durch den 24h-Straßenverkehrslärm L_{den} *stark belastet***, vergleichsweise sind hier der Fluglärm mit 7% und der Schienenverkehrslärm mit 11% Anteil *stark belasteter* Gebäude und Wohnungen nur in wesentlich untergeordnetem Ausmaß beteiligt. Autobahn- und Fluglärm zeigen ein sehr ähnliches Bild was die Belastung betrifft (S. 48).
- Die Verkehrslärmbelastungen werden zunächst als **Rasterlärmkarten L_{den}** getrennt nach Quellen unter Berücksichtigung der drei Belastungskategorien „*gering (<45dB)*“, „*mittel (45-55dB)*“ und „*stark (>55dB)*“ einzeln dargestellt:
  - **Fluglärm:** Während sich der Bereich der starken Belastung auf das unmittelbare Flughafenumfeld beschränkt, erstreckt sich das Gebiet der mittleren Belastung in West-Ostrichtung über einen weiten Bereich des Stadtgebietes (S. 50).
  - **Schienenverkehrslärm:** Der Schienenkorridor von Ost nach West im Inntal mit der Abzweigung der Brennerbahn in Richtung Süden bildet sich in den Lärmeinträgen deutlich ab. Entlang dieses Korridors ergibt sich eine starke Belastung, der Abzweig der Karwendelbahn in Richtung Nordwest ist noch erkennbar, die Straßenbahn tritt kaum in Erscheinung (S. 51).

- **<u>Straßenverkehrslärm:</u>** Der Straßenverkehrslärm belastet Innsbruck sehr großräumig. Ausgenommen sind lediglich die südlichen Randgemeinden Igls und Vill, wie auch Teile von Mühlau und Arzl. Die Katastralgemeinden im Stadtkern weisen ein durchgehend hohes Belastungsbild auf. Die im Süden Innsbrucks vorbeiführende Autobahn A12, sowie auch der Abzweig der Brennerautobahn A13 belasten vor allem die Stadtteile Amras, Pradl und Wilten, ebenso wie einen Teil von Hötting. Lärm von der Brennerautobahn wirkt sich vor allem auf unbesiedeltes Gebiet in der Katastralgemeinde Vill aus (S. 52).

3.2. Verkehrslärmbelastung

– Die **Straßenverkehrslärmbelastung**, welche im Mittel mit rund **55dB Dauerschallbelastung** (L_{den}) auf die Gesamtinnsbrucker Wohngebiete einwirkt, ist die dominierende Lärmquelle in Innsbruck. Mit deutlichem Abstand (mehr als 10dB und damit in ihrer Lautheit rund halb so laut) folgen die weiteren Verkehrslärmquellen **Flug-, Schienenverkehrs- und Autobahnlärm**, die in ihrem mittleren Immissionspegeln **zwischen 44 und 44,6dB** L_{den} fast gleichauf liegen (S. 54).

– Während über die Hälfte der Innsbruckerinnen und Innsbrucker vom Flug-, Schienen- und Autobahnlärm nur gering (<45dB) belastet sind, fallen **über 90%** der Einwohnerinnen und Einwohner beim **Straßenverkehrslärm** in die Kategorien der **mittelgradig bzw. stark Belasteten** (S. 55).

– Der **Straßenverkehrslärm** ist besonders in den Katastralgemeinden **Wilten, Innsbruck, Pradl und Amras** sehr ausgeprägt und liegt in diesen Stadtteilen über dem Schwellenwert von 55dB Dauerschallbelastung. Der **Fluglärm** belastet besonders **Hötting** und reicht dort im Mittel knapp an die 50dB-Schwelle. Der **Schienenverkehrslärm** sticht in den Katastralgemeinden **Innsbruck, Mühlau und Wilten** hervor, wo ebenfalls knapp eine 50dB-Dauerbelastung der Wohngebäude erreicht wird. **Igls und Vill sind die ruhigsten Katastralgemeinden** der Stadt Innsbruck (S. 55).

– Über 80% aller Adresspunkte in **Wilten** sind stark vom **Straßenverkehrslärm** belastet, in den Katastralgemeinden **Innsbruck, Pradl und Amras** sind es über 60%, in Igls und Vill hingegen nur 14%. Vom **Fluglärm** starkbelastete Wohngebäude sind ausschließlich in **Hötting** vorhanden (20,2% aller bewohnten Adresspunkte). Der **Schienenverkehrslärm** belastet in **Innsbruck und Wilten** rund ein Viertel aller Gebäude stark. Die **Autobahn** wirkt sich vor allem in **Wilten** (24%) und **Amras** (17,3%) stark lärmbelastend aus (S. 57).

– Die Lärmbelastung nach Quelle verändert sich im **Nachtzeitraum** (22h bis 6h) deutlich. Der **Straßenverkehrslärm** bleibt die mit Abstand **dominierende Lärmquelle**, nimmt aber im Vergleich zu den 24h-Pegeln um rund 10dB ab und ist damit **in seiner Lautheit halbiert**. Gleiches gilt für den Autobahnlärm. Der Schienenverkehrslärm verringert sich im Nachtzeitraum im Mittel um rund 7dB. Am stärksten nimmt in den Nachtstunden die Verkehrslärmbelastung durch den **Flugverkehr** ab. Diese **verringert sich um knapp 18dB** gegenüber dem 24h-Pegel und trägt damit im Vergleich der einzelnen Verkehrslärmquellen mit Abstand am wenigsten zur Gesamtlärmbelastung Innsbrucks in der Nacht bei (S. 59).

– **Über 50%** der Innsbruckerinnen und Innsbrucker sind **auch in der Nacht mittelgradig bzw. stark vom Straßenverkehrslärm belastet**. Beim **Schienenverkehrslärm** reduziert sich der Anteil der mittelgradig bzw. stark Belasteten in der Nacht auf **unter 14%**, bei den **Autobahnlärmbelasteten** auf **unter 8%**. **Stark fluglärmbelastete Adresspunkte gibt es im Nachtzeitraum in Innsbruck nicht**, deutlich weniger als 1% sind als mittelgradig belastet einzustufen (S. 61).

– Die **nächtliche Straßenverkehrslärmbelastung** liegt im Mittelwert aller bewohnten Innsbrucker Adresspunkte **in Wilten am höchsten** und dort auch über dem Schwellenwert von 50dB. Der **Flugverkehrslärm** in der Nacht sinkt auch in der im 24h-Zeitraum am stärksten belasteten Katastralgemeinde Hötting **unter** die **30dB-Pegelgrenze**. **Igls und Vill** sind auch **im Nachtzeitraum** – sowohl nach einzelnen Verkehrsträgern, als auch insgesamt – **die ruhigsten Katastralgemeinden** der Stadt Innsbruck (S. 61).

– Die **Katastralgemeinde Innsbruck** weist den höchsten Anteil an stark vom Straßenverkehrslärm in der Nacht belasteten Wohngebäuden auf (Schallpegel L_n >55dB). Auch in **Wilten** ist eine erhebliche Anzahl von Adresspunkten (238 Wohngebäude entspricht 19%) in der Nacht starken Lärmpegeln aus dem **Straßenverkehr** ausgesetzt. Weiters sind Innsbruck und Wilten zum Teil (rund 7%) in der Nacht stark **schienenverkehrslärmbelastet**. Stark vom **Autobahnlärm** in der Nacht belastete Wohngebäude treten **nur vereinzelt** auf in Amras (9 Wohngebäude), Wilten (4 Wohngebäude) und Pradl (1 Wohngebäude) auf. Stark vom Flugverkehrslärm belastete Gebäude gibt es im Nachtzeitraum in ganz Innsbruck nicht. In Igls ist unabhängig von der Lärmquelle kein einziger Adresspunkt von Nachtlärmpegeln über 55dB betroffen (S. 63).

3.3. Gesamtlärmbetrachtung Innsbruck

– Die gemeinsame Darstellung von Schallimmissionen aus unterschiedlichen Schallquellen wird zunächst erläutert und für die einzelnen Verkehrslärmquellen in Form von **wirkungs- und effektbezogenen Rasterlärmkarten** dargestellt:

  - **Autobahnlärm** (S. 67)
  - **Straßenverkehrslärm** (S. 68)
  - **Fluglärm** (S. 70 und 71)
  - **Schienenverkehrslärm** (S. 72 und 73)

  Die **Belastungszonen beim Fluglärm weiten sich** in der Darstellung der effektbezogener Substitutionspegel deutlich **aus** und die Katastralgemeinden Innsbruck, aber auch Reichenau und Pradl zeigen sich deutlicher betroffen. Im Gegensatz dazu werden die Belastungszonen ausgedrückt als effektbezogener Substitutionspegel **beim Schienenverkehr deutlich kleiner**, die Dominanz in einigen Bereichen geht daher deutlich zurück.

– Aus den drei Einzelkarten für Straßen-, Flug- und Schienenverkehr wird durch Pegeladdition das Herzstück – die ***Gesamtlärmbelastung Innsbrucks*** ermittelt und dargestellt (S. 75).

– Über ***Hotspotanalysen*** können **Priorisierungsmöglichkeiten für Maßnahmen** visualisiert werden, indem Bereiche, in denen vergleichsweise viele Menschen vergleichsweise hohen Gesamtlärmpegeln ausgesetzt sind, dargestellt werden. Daher wurde für jede Verkehrsquelle an jedem Gebäude in Innsbruck die nach **Exposition-Wirkung-Beziehungen** ermittelte Wahrscheinlichkeit einer Belästigung berechnet, zu einem **Gesamteinwirkungsindex** der unterschiedlichen Lärmquellen zusammengeführt und mittels Rasterzellen die Verteilungssituation dargestellt (S. 76).

  4 besondere Hotspots zeichnen sich ab:

  - **Bereich Haydnplatz** (Katastralgemeinde Innsbruck): Hier ist nicht die Lärmbelastung allein ausschlaggebend, sondern auch die hohe Dichte der Bewohnerinnen und Bewohner.

- **Bereich General-Eccher-Straße** (Pradl): Kumulierender Effekt durch Überlagerung von Schienen- und Straßenverkehrslärm (Westbahn und B 171) in Kombination mit der hohen Anzahl der Hauptwohnsitzgemeldeten.
- **Bereich Dr.-Ferdinand-Kogler-Straße** (Amras): Sich überlagernde Belastungen durch den Erschließungsverkehr des Einkaufszentrums und der Autobahnzubringer, ebenso wie durch die mehrseitigen Belastungen durch das Gemeindestraßennetz.
- **Bereich Kranebitter Allee** (Hötting): Kumulierung der verschiedenen Geräuschquellen - besonders der B 171 und der Schallimmissionen des Flugverkehrs – in seinem Ausmaß ist dieser Hotspot doppelt so groß wie die drei anderen.

– Zur Auffindung **schützenswerter, *ruhiger Bereiche*** im Stadtgebiet von Innsbruck mit einem effektbezogener Substitutionspegel $L_{A,ES}$ < 55dB wurde eine detaillierte ***Summenlärmkarte*** erstellt, die neben Parks und Grünflächen und den vielen ruhigen Gebieten außerhalb der Kernbebauung vor allem die **wertvollen kleinräumigen Strukturen** in Innsbruck aufzeigt. Dabei fällt die klassische **Blockrandbebauung** einzelner Stadtteile Innsbrucks (etwa im Saggen und in Pradl) auf, die als **beruhigte Lärmoasen** eine sehr hohe Bedeutung für die Belästigungsreaktion haben. Insgesamt gibt es vor allem **im innerstädtischen Bereich** jedoch **nur** sehr **wenige ruhigere Bereiche** und **im Westen Innsbrucks** bestehen **durch die Fluglärmeinwirkung keine lärmberuhigten Innenhofsituationen** mehr (S. 78).

4. Durchführungsparameter der Befragungen

Der Berichtsteil **Durchführungsparameter der Befragungen** leitet von den Lärm- zu den Befragungsdaten über und enthält detaillierte Darstellungen zum Setting und Untersuchungsumfang (S. 80), zum Fragebogenaufbau (S. 81), zu Sampling und Stichprobe (S. 83), Rücklaufquote (S. 87), Befragungsdurchführung und Datenkontrolle (S. 89), zum Datenschutz (S. 90) und zu den angewandten statistischen Methoden (S. 91).

– Insgesamt **1031 Personen** wurden im Stadtgebiet von Innsbruck mittels einer repräsentativen Vorortbefragung durch geschulte Interviewerinnen und Interviewer befragt.

– Die erreichte **Rücklaufquote von 47,8%** (S. 88) gewährleistet eine sehr hohe Repräsentativität der Ergebnisse, minimiert systemimmanente Bias (Selbstselektion, Stichprobenfehler, mangelnde Repräsentativität usw.) und ist für eine Vor-Ort-Befragung in einem urbanen Raum als **außergewöhnlich hoch** einzustufen.

– Ein geschichtetes Adress-Cluster-Sample auf Basis der Daten des *Zentralen Melderegisters* nach Lärmkorridoren (Belastungsgruppen) unter Einbeziehung der einzelnen Verkehrsträger (S. 86), der demographischen Parameter (S. 83) und nach Katastralgemeinden (S. 84) wurde gezogen.

– Alle bewohnten Adresspunkte im Stadtgebiet von Innsbruck wurden mit **punktgenauen Fassadenlärmpegeln** für den Tag- und den Nachtzeitraum aus dem entwickelten Gesamtlärmmodell (Bezugsjahr 2015) für sämtliche Verkehrsträger verschnitten.

5. Auswertung der Befragungen

Die **Auswertungen der Befragungen** werden ab S. 53 dargestellt und umfassen die abgefragten Themenbereiche Demographie (S. 93), Wohnen (S. 98), Mobilität (S. 103), Lebensbedingungen in Innsbruck (S. 113), Zufriedenheit mit der eigenen Wohnsituation (S. 134), Gesundheitszustand (S. 145), Lebensqualität (S. 150), Lärmempfindlichkeit (S. 156), Wind- und Wettereinflüsse auf die Lärmbelastung (S. 163), Nachtschlaf bei geöffnetem Fenster (S. 166), Lärmexpositionsempfinden (S. 170), Belästigung / Störung durch Lärm (S. 204), Belästigung / Störung durch Lärm im Schlaf (S. 245), Lärmcoping (S. 287), Empfundene Zu- oder Abnahme des Lärms in Innsbruck (S. 298), Einschätzung der Auswirkungen von Lärm auf die Gesundheit (S. 302), Bekanntheit der Umgebungslärmkarten (S. 305), Einschätzung des Lärmniveaus im Städtevergleich (S. 306), Einschätzung des eigenen Lärmbeitrags (S. 310), Einschätzung der störendsten Lärmquelle in Innsbruck (S. 313) und Sinnhaftigkeit von Maßnahmen zur Lärmreduktion in Innsbruck (S. 319).

5.1. Mobilität

- **Nur 9%** aller Befragten **pendeln** zur Arbeits- oder Ausbildungszwecken aus der Tiroler Landeshauptstadt in andere Umlandgemeinden (S. 103), bezogen allein auf die berufstätigen bzw. sich in Ausbildung befindlichen befragten Innsbruckerinnen und Innsbrucker (ohne Nichtzutreffende, die sich in Pension befinden, nicht berufstätig sind usw.) sind es 14,6%.
- **87,5%** aller Befragten **gehen regelmäßig** (mehrmals pro Woche bis fast täglich) länger als 10 Minuten **zu Fuß** (S. 110), knapp **50% nutzen regelmäßig ein Auto** als Fahrerin / Fahrer (S. 104), **über 45%** - über alle Altersklassen hinweg - ein **Fahrrad** (S. 109) und **37,5% öffentliche Verkehrsmittel** in der Stadt (S. 106).
- Die Nutzung von **öffentlichen Verkehrsmitteln** (Eisenbahn, Autobus) **außerhalb der Stadt** Innsbruck beschränkt sich bei den Befragten größtenteils auf **Gelegenheitsnutzungen**, was direkt mit dem geringen Auspendleranteil aus der Landeshauptstadt assoziiert ist (S. 107).
- **Ein Drittel** aller befragten Innsbruckerinnen und Innsbrucker **nutzt mehrmals pro Jahr ein Flugzeug** (umfasst auch einen einmal im Jahr stattfindenden Urlaub, der mit dem Flugzeug als Transportmittel erreicht wird und bei dem im Regelfall zumindest zwei Flüge – ein Hin- und ein Rückflug – absolviert werden), mehr als die Hälfte nur alle paar Jahre (S. 111).
- Das **Mobilitätsverhalten insgesamt hängt stark mit der Lage der eigenen Wohnung / des eigenen Hauses zusammen**. Bewohnerinnen und Bewohner des Zentrums von Innsbruck (am stärksten ausgeprägt in der Katastralgemeinde Innsbruck, aber auch in Hötting, Pradl und Wilten ist dieser Effekt nachzuvollziehen) fahren wesentlich weniger häufig mit dem Auto, dafür häufiger mit öffentlichen Verkehrsmitteln und vor allem dem Fahrrad bzw. gehen zu Fuß, als dies in Arzl, Mühlau und besonders in Igls und Vill der Fall ist.

5.2 Lebens- und Wohnbedingungen in Innsbruck

- Die **Güte der Lebens- und Wohnbedingungen** in Innsbruck wird über alle 8 Fragen hinweg in allen Teilbereichen überwiegend **sehr hoch** eingeschätzt (S. 113). Am höchsten werden die **öffentlichen Verkehrsmittel** (S. 117) , die eigene **Verbundenheit zur Stadt Innsbruck** (S. 119) und die Frage nach der **Güte von Landschaft, Natur, öffentlichen Parks und Grünflächen**

(S. 116) beurteilt. Vergleichsweise deutlich niederer die **Wohnbedingungen** (S. 114), die **Sicherheit** (S. 115) und die **Luftqualität** (S. 117). Die **Nachbarschaftsbeziehungen** (S. 114) und **die Bedingungen für das Aufwachsen von Kindern** (S. 118) liegen dazwischen.

– Die Beurteilung der Lebens- und Wohnbedingungen unterscheidet sich zwischen den einzelnen Katastralgemeinden von Innsbruck (S. 120). Im **Summenscore** über alle 8 Fragen hinweg, schätzen die Befragten in **Mühlau** die Wohn- und Lebensbedingungen **am höchsten** und die befragten Einwohnerinnen und Einwohner von **Amras am niedrigsten** ein. Insgesamt kann festgestellt werden, dass die die Lebensbedingungen in Innsbruck **in den äußeren Katastralgemeinden höher** eingeschätzt werden **als in den innerstädtischeren Gemeinden**.

– **Je stärker** eine befragte Person in ihrem Wohnumfeld vom **Straßenverkehrslärm** belastet ist (S. 125), sich selbst als **lärmempfindlich** (S. 123) einstuft, sich **Lärm ausgesetzt** (S. 126) oder von diesem **belästigt** (S. 129) fühlt, desto geringer bewertet sie über alle Fragen hinweg die **Güte der Lebensbedingungen** in Innsbruck – mit 2 Ausnahmen: Die Verbundenheit zur Stadt Innsbruck und die Güte des öffentlichen Verkehrs stehen in keinem oder deutlich geringerem Zusammenhang zu diesen Parametern.

– Ausgeprägte **Beziehungen** der Einschätzung der Lebens- und Wohnbedingungen in Innsbruck zur gegebenen Schienen- (S. 125) und Flugverkehrslärmbelastung (S. 126) finden sich im Unterschied **zur Straßenverkehrslärmbelastung** (S. 125) nicht. Die persönliche Einschätzung der eigenen Ausgesetztheit (S: 126) und Belästigung durch Lärm (S. 129) zeigt hingegen für sämtliche Verkehrsträger Unterschiede in der Bewertung der Lebensbedingungen in Innsbruck auf.

– **Weitere Variablen** wie Alter (S. 122), höchster erreichter Schulabschluss (S. 122), Einschätzung der eigenen Lebensqualität (S. 124), Nachtschlaf bei offenem Fenster (S. 133) u.a.m. zeigen ebenfalls **deutliche Bezüge** zur Beurteilung der Lebens- und Wohnbedingungen.

5.3. Zufriedenheit mit der eigenen Wohnsituation

– **Über 70%** aller Befragten sind mit ihrer eigenen Wohnsituation **sehr zufrieden** und antworten in den drei höchsten von 11 möglichen Antwortkategorien, 2,7% sind nicht zufrieden und antworten in den drei Untersten (S. 134).

– Die Zufriedenheit mit der eigenen Wohnsituation ist **in den äußeren Katastralgemeinden höher** als in den inneren Stadtteilen (S. 135).

– Das Ausmaß des gegebenen **Straßenverkehrslärms zeigt deutliche Bezüge** zur Zufriedenheit mit der eigenen Wohnsituation (S. 137), für den Flugverkehrslärm gilt dies eingeschränkt (S. 139), für den Schienenverkehrslärm sind hingegen keine eindeutigen statistischen Zusammenhänge nachweisbar (S. 141).

– Aus den Daten ist ein **Schwellenwert bei etwa 40dB L_n nächtlicher Straßenverkehrslärm** ersichtlich, ab welchem die Zufriedenheit mit der eigenen Wohnsituation abnimmt (S. 140).

– Befragte, die sich als **stark verkehrslärmexponiert** oder vom Verkehrslärm **stark belästigt** ansehen, sind **weniger zufrieden** mit ihrer persönlichen Wohnsituation als sich weniger dem Verkehrslärm ausgesetzt (S. 141) oder von diesem belästigt (S. 143) sehende Probandinnen und Probanden. Die deutlichsten Zusammenhänge zeigen sich in Bezug zum Straßenverkehrslärm, gering ausgeprägte Beziehungen bestehen zum Expositions- und Belästigungsempfinden gegenüber dem Schienen-, Autobahn- und Flugverkehrslärm.

- Sich stark **nicht verkehrsverursachtem Lärm** ausgesetzt oder von diesem belästigt empfindende Befragte schätzen ihre Zufriedenheit mit der eigenen Wohnsituation deutlich schlechter ein, als dies sich weniger ausgesetzt / belästigt fühlende Befragte tun. Die **Unterschiede** treten **stärker als beim verkehrsverursachten Lärm** zutage und besonders befragte Innsbruckerinnen und Innsbrucker, die sich dem **Nachbarschaftslärm**, dem **Lärm von Passanten und Gaststätten** oder aber **Erschütterungen** stark ausgesetzt (S. 142) oder von diesem belästigt (S. 143) sehen, beurteilen ihre eigene Wohnsituation insgesamt um mehr als 2 Antwortkategorien schlechter als gering Exponierte / Belästigte angeben. In Zusammenschau mit den Belästigungsreaktionen im Kapitel *Belästigung / Störung durch Lärm* (ab S. 204) wird ersichtlich, dass **Akutbelastungseffekte durch nicht verkehrsverursachtem Lärm** eine **deutliche negative Beziehung** zu einer Reihe von subjektiven Einschätzungsparametern aufweisen, die in ihrer Ausprägung stärker als beim verkehrsverursachten Lärm zutage treten.

5.4. Gesundheitszustand

- **Knapp drei Viertel** aller Befragten schätzen ihren eigenen Gesundheitszustand als ***gut* bzw. *sehr gut*** ein, 8,6% als *weniger gut* bzw. *schlecht* (S. 145).
- Den größten **Zusammenhang** zur Einschätzung des eigenen Gesundheitszustandes zeigt erwartungsgemäß das **Alter** der Befragten. **Männer** schätzen ihren eigenen Gesundheitszustand geringfügig besser ein als **Frauen**. Befragte mit schlechtem selbstreferenzierten Gesundheitszustand bewerten ihr **Lebensqualität** schlechter als Befragte mit gutem Gesundheitszustand und vice versa (S. 146).
- Auffällig ist die Zusammenschau Gesundheitszustand und **höchster erreichter Bildungsabschluss**: Befragte mit Pflichtschulabschluss gehen von einem deutlich schlechteren eigenen Gesundheitszustand aus als Befragte mit sekundärem Bildungsabschluss und diese unterscheiden sich wiederum ebenso deutlich von befragten Akademikerinnen und Akademikern. Diese **Unterschiede bleiben auch altersbereinigt erhalten** und sind besonders in den beiden höheren Altersklassen sehr ausgeprägt (S. 146).
- **Bezüge zur** vorhanden **Lärmbelastung** sind **in nur geringem Ausmaß vorhanden** (S. 147) und auch die Selbsteinschätzung der eigenen Lärmempfindlichkeit (S. 146), Ausgesetztheit (S. 147) und Belästigung (S. 148) stehen nur geringfügig in Beziehung zur Einschätzung des eigenen Gesundheitszustandes.

5.5. Lebensqualität

- Genau 1% aller Befragten beurteilt die eigene Lebensqualität in den drei untersten Antwortkategorien und damit als schlecht, **über 70%** sind hingegen der Meinung, dass ihre **persönliche Lebensqualität sehr hoch** ist und antworten in den drei obersten Antwortkategorien (S. 150).
- **Frauen** bewerten ihre persönliche Lebensqualität etwas **höher als Männer** (S. 151).
- **Knapp 80%** der Befragten **mit tertiärem höchsten erreichten Schulabschluss schätzen ihre eigene Lebensqualität als *sehr gut* ein**, bei den Probandinnen und Probanden aus der sekundären Bildungsschicht sinkt dieser Anteil auf knapp über zwei Drittel und bei Befragten mit Pflicht-

schulabschluss beurteilen nur mehr 56% ihre persönliche Lebensqualität als *sehr gut* (S. 151). Auch altersbereinigt bleiben diese Unterschiede annähernd bestehen.

- Die Einschätzung der Güte der eigenen Lebensqualität zeigt **nur geringfügige Beziehungen zur tatsächlich vorhandenen Verkehrslärmbelastung** im Wohnumfeld der Befragten (S. 152). Eine (statistisch nur gering ausgeprägte) Beziehung besteht zwischen der Einschätzung der eigenen Lebensqualität und der nächtlichen Straßenlärmbelastung. Mit steigenden nächtlichen Straßenlärmpegeln sinkt die Einschätzung der eigenen Lebensqualität (S. 152).
- Ausgeprägter als zur tatsächlich gegebenen Verkehrslärmbelastung zeigen sich Zusammenhänge zur empfundenen Ausgesetztheit und Belästigung durch Lärm. **Je stärker sich Befragte lärmexponiert** (S. 153) **oder belästigt** (S. 154) **fühlen, desto geringer schätzen sie ihre persönliche Lebensqualität ein.** Vor allem in der Zusammenschau der Einschätzung der eigenen Lebensqualität mit dem Expositions- (S. 153) und Belästigungsempfinden (S. 155) gegenüber Erschütterungen, dem Lärm insgesamt, dem Straßenverkehrs- und dem Nachbarschaftslärm zeigen sich Bezüge.
- In der Zusammenschau der Beurteilung der eigenen Lebensqualität und der Lärmbelästigung fällt auf, dass besonders im Falle der Belästigung durch Schienenverkehrs- und Autobahnlärm die beiden Extremgruppen der Gering- und der Starkbelästigten in der Beurteilung ihrer persönlichen Lebensqualität nicht sehr weit auseinanderliegen. Sehr deutlich unterscheiden sich diese beiden **Extremgruppen** hingegen bei der Belästigung durch Straßenlärm, Nachbarschaftslärm, Lärm insgesamt und bei der Belästigung durch Erschütterungen (S. 155).
- **Im Nachtzeitraum tritt die nicht verkehrsverursachte Lärmbelästigung wesentlich deutlicher in den Vordergrund als im 24h-Zeitraum** (S. 155).

5.6. Lärmempfindlichkeit

- Etwas mehr als ein Viertel aller Befragten schätzt sich selbst als ***überhaupt nicht* bis kaum lärmempfindlich** ein (unterste 3 Antwortkategorien), knapp 17% hingegen als **stark bis *außerordentlich lärmempfindlich*** (oberste 3 Antwortkategorien). Die Mehrheit der Befragten siedelt das Ausmaß der eigenen Lärmempfindlichkeit in den mittleren 5 Antwortkategorien an (S. 156).
- Das Ausmaß der **Lärmempfindlichkeit** der Befragten zeigt **keine deutliche Beziehung zur tatsächlich vorhandenen Verkehrslärmbelastung im Wohnumfeld** der Befragten - im 24h-Zeitraum ebenso wenig wie im Nachtzeitraum (S. 158).
- **Stark lärmempfindliche Befragte fühlen sich häufiger und stärker vom Lärm belästigt** und sich stark vom Lärm gestört empfindende Befragte schätzen sich selbst als lärmempfindlicher ein (S. 160). Die Unterschiede im Antwortverhalten nach Lärmquelle sind besonders im Vergleich der Extremgruppen beim Straßenverkehrs- und Fluglärm, sowie dem Lärm insgesamt sehr stark ausgeprägt, während beim nicht verkehrsverursachten Lärm, sowie dem Schienenverkehrslärm die Extremgruppen geringer voneinander abweichen (S. 161).
- Bezüge zu weiteren ausgewählten Variablen (S. 157):
  - **Frauen** schätzen sich selbst häufiger und auch im Ausmaß **stärker** als **lärmempfindlich** ein als Männer.
  - Die Altersgruppe über 60 Jahren hat den größten Anteil an nicht bis kaum lärmempfindlichen Befragten. Insgesamt empfinden sich jedoch die Befragten der Altersgruppe der 18 bis 40-

Jährigen am wenigsten lärmempfindlich, während in der **Gruppe der 40 bis 60-Jährigen die meisten** und am stärksten **Lärmempfindlichen** enthalten sind. Insgesamt ist eine Zunahme der Häufigkeit und des Ausmaßes der Lärmempfindlichkeit mit ansteigendem Alter bis zur Schwelle des 65. bis 70. Lebensjahres festzustellen.

- Sehr deutlich unterscheiden sich (auch altersbereinigt) erneut die Bildungsschichten: **Akademikerinnen und Akademiker** stufen sich im Vergleich mit Pflichtschulabgängerinnen und -Abgängern sowohl im Ausmaß als auch in der Häufigkeit in etwa **doppelt so** stark und häufig als sehr **lärmempfindlich** ein und umgekehrt.
- Lärmempfindlichere Befragte schätzen die negativen Auswirkungen von Lärm auf die Gesundheit deutlich stärker ein als weniger Lärmempfindliche.
- Probandinnen und Probanden mit selbstreferenziertem schlechteren Gesundheitszustand sind häufiger sehr lärmempfindlich als Befragte mit gutem Gesundheitszustand.
- **Befragte** Innsbruckerinnen und Innsbrucker **mit hoher Lebensqualität** sehen sich **weniger** häufig und in geringerem Ausmaß als **lärmempfindlich** an als Personen mit geringerer Lebensqualität.
- Diejenigen Befragten, die davon ausgehen, dass es in Innsbruck im Vergleich zu anderen Städten Österreichs ruhiger ist, sind deutlich weniger häufig und weniger stark lärmempfindlich als Befragte, welche glauben, dass Innsbruck gleich laut oder lauter als andere vergleichbare Städte ist.

5.7. Nachtschlaf bei geöffnetem / gekipptem Fenster

- **Fast 50%** aller befragten Innsbruckerinnen und Innsbrucker **schlafen nachts *häufig bis immer* bei geöffnetem oder gekippten Fenster**, weitere **30%** tun dies zumindest ***gelegentlich***. Nur 0,7% der Befragten verfügen über eine *Wohnraumlüftung*, 6,6% möchten nicht bei geöffnetem oder gekipptem Fenster schlafen und für 12,3% wäre dies *zu laut* (S. 166).
- Wie zu erwarten schlafen Befragte, die über einen lärmgeschützten Innenhof, eine lärmabgewandte Seite oder gute bauliche Abschirmungen gegen den Verkehrslärm verfügen, häufiger bei geöffnetem oder gekipptem Fenster als Befragte, die über keinerlei oder wenig baulichen Lärmschutz verfügen. Die Unterschiede sind allerdings kleiner als erwartet und **der reine bauliche Lärmschutz an sich** scheint **nicht das ausschlaggebende Element** für die Entscheidung der Befragten, ob sie bei geöffnetem oder geschlossenem Fenster schlafen, zu sein (S. 167).
- Befragte, die sich als stark lärmempfindlich einstufen, dem Lärm stark ausgesetzt sehen, ihre Lebens- und Wohnbedingungen schlechter einstufen, sich vom Lärm stark belästigt fühlen, oder der Meinung sind, es sei in Innsbruck lauter als in anderen vergleichbaren österreichischen Städten schlafen deutlich häufiger bei geschlossenen Fenstern, als jene die sich weniger ausgesetzt und/oder gestört fühlen bzw. weniger lärmempfindlich sind etc. Jedoch schlafen auch in diesen Extremgruppen rund zwei Drittel häufig bei geöffneten / gekippten Fenstern (S. 167).
- Deutlich zeigt sich ein **Bezug zur gegebenen nächtlichen Straßenverkehrslärmbelastung** (S. 168), **nicht jedoch zur Schienen- und zur** (im Nachtzeitraum vergleichsweise geringen) **Flugverkehrslärmbelastung** (S. 168).

5.8. Baulicher Lärmschutz

- **Drei Viertel** aller Befragten **sehen die Wohnung / das Haus in dem sie wohnen als *gar nicht* oder nur *sehr geringfügig* durch bauliche Abschirmungen wie Gebäude, Mauern etc. vor Verkehrslärm geschützt**. Knapp ein Viertel der befragten Innsbruckerinnen und Innsbrucker gibt hingegen an, *zum Großteil* oder *vollständig* vom Verkehrslärm abgeschirmt zu wohnen (S. 101).
- Über **zwei Drittel** der befragten Innsbruckerinnen und Innsbrucker geben an, ihre Wohnung bzw. das Haus, in dem sie wohnen, hätte einen **vor Verkehrslärm geschützten Innenhof bzw. eine lärmabgewandte Seite** (S. 101), was zu **signifikant geringeren Expositions- und Belästigungsreaktionen in dieser Gruppe** führt.
- Dezidiert über **Lärmschutzfenster** verfügen **18%** der Befragten, knapp 17% wissen darüber nicht Bescheid und rund zwei Drittel haben keine Lärmschutzfenster verbaut (S. 102). Vorrangig sollen die verbauten Lärmschutzfenster vor dem Straßenverkehrslärm schützen (S. 102).

5.9. Lärmexpositionsempfinden

- **13,6%** aller befragten Innsbruckerinnen und Innsbrucker schätzen ihre Wohnung bzw. das Haus in dem sie leben, dem Lärm insgesamt als **stark ausgesetzt** ein, 61% antworten in den mittleren Kategorien und etwas **mehr als ein Viertel** aller Probandinnen und Probanden empfindet das eigene Wohnumfeld als **gering** dem Lärm insgesamt **ausgesetzt** (S. 173).
- Die eigene **Exposition dem Flugverkehrslärm gegenüber wird im Mittel am höchsten eingeschätzt**, gefolgt von der Ausgesetztheit gegenüber **Straßenverkehrslärm**. Erst mit deutlichem Abstand folgen **Bau- und Nachbarschaftslärm**, sowie die weiteren erfassten Lärmquellen und die **Erschütterungsexposition**. Die Ausgesetztheit der eigenen Wohnung / des eigenen Hauses gegenüber **Schienenverkehrs- und Autobahnlärm**, sowie dem **Lärm von Gewerbe und Industrie** ist für die befragten Innsbruckerinnen und Innsbrucker im Durchschnitt am geringsten ausgeprägt (S. 170).
- Befragte Innsbruckerinnen und Innsbrucker,
  - deren Wohnung durch bauliche Abschirmungen (Gebäude, Mauern etc.) vor Verkehrslärm weitestgehend geschützt sind,
  - die häufig bei geöffnetem oder gekipptem Fenster schlafen,
  - die über keine Lärmschutzfenster verfügen,

  empfinden sich dem Lärm insgesamt weniger ausgesetzt. Auch bei den **subjektiven Parametern** wird der deutliche Einfluss der **Disposition** deutlich, die auch nach Adjustierung mit den gegebenen Verkehrslärmpegeln erhalten bleibt: Befragte, welche

  - ihre Lebensbedingungen und ihre Lebensqualität besser einschätzen,
  - zufriedener mit ihrer Wohnsituation insgesamt sind,
  - von keiner Zunahme der Lärmbelastungen in den vergangenen Jahren ausgehen,
  - Innsbruck im Vergleich zu anderen Städten Österreichs für ruhiger halten,
  - keine Notwendigkeit für (weitere) Maßnahmen zur Lärmreduktion sehen,
  - sich selbst als weniger lärmempfindlich einstufen,

  fühlen sich weniger häufig und in geringerem Ausmaß lärmexponiert (S. 175).

- Im Mittel die **größte Lärmexposition insgesamt** wird **in Wilten** empfunden, gefolgt von den Katastralgemeinden Innsbruck, Pradl und Hötting. Die **geringste Ausgesetztheit** gegenüber Lärm insgesamt im eigenen Wohnumfeld empfinden die Befragten **in Mühlau, Igls und Vill** (S. 174).
- Das **Expositionsempfinden** gegenüber Lärm beginnt **im** sensibleren **Nachtzeitraum** im Vergleich zu den 24h-Pegeln bereits bei niedereren Belastungspegeln stärker zu steigen (S. 172).
- **Knapp 39%** aller Befragten sehen sich dem **Flugverkehrslärm nicht stark ausgesetzt**. Das Empfinden, wie sehr das persönliche Wohnumfeld der Befragten dem Flugverkehrslärm exponiert ist, übertrifft allerdings in der **Extremgruppe der sich stark ausgesetzt Empfindenden** in seiner Häufigkeit (**16%**) die Spitzengruppe bei der Frage nach der Lärmexposition insgesamt und liegt auch **vor dem Expositionsempfinden durch Straßenverkehrslärm** (S. 177). Entsprechend der gegebenen Fluglärmbelastung zeigt sich das mit Abstand **stärkste Fluglärmexpositionsempfinden in Hötting**. Die **geringste Ausgesetztheit gegenüber Fluglärm** im eigenen Wohnumfeld empfinden die Befragten **in Igls** (S. 178).
- Das Ausmaß des **Expositionsempfindens gegenüber Straßenverkehrslärm** entspricht in seinen Dimensionen größtenteils dem Empfinden der Ausgesetztheit des eigenen Wohnumfeldes gegenüber Flugverkehrslärm und liegt **nur leicht darunter** (S. 180). Das **größte Ausmaß** an empfundener Straßenlärmexposition wird mit deutlichem Abstand **in Wilten** empfunden, **gefolgt von Pradl, Innsbruck und Amras**. Die geringste Ausgesetztheit gegenüber Straßenverkehrslärm im eigenen Wohnumfeld empfinden die Befragten **in Igls, Mühlau und Vill** (S. 182).
- Die empfundene **Ausgesetztheit gegenüber Straßen- und Flugverkehrslärm korrespondiert sehr ausgeprägt mit der tatsächlich gegebenen Lärmbelastung und der folgenden Lärmbelästigung** (vgl. S. 178 und S. 181). Im Unterschied zum Expositionsempfinden beim Fluglärm steigt das Empfinden einer starken **Straßenverkehrslärmexposition erst bei höheren gegebenen Lärmpegeln** deutlich an und ist insgesamt geringer ausgeprägt (S. 172).
- Das Empfinden der **Exposition durch den Lärm von Baustellen** - der stark aus **Akutbelastungen** resultiert - steht in seinem Ausmaß **an dritter Stelle** hinter dem Flug- und dem Straßenverkehrslärm, unterscheidet sich aber in seinen Ausprägungen sehr deutlich von diesen beiden: Mit **über 44%** ist der Anteil an Befragten, die sich ***überhaupt nicht*** **dem Baustellenlärm exponiert** fühlen mehr als dreimal höher als beim Flug- und Straßenverkehrslärm. **Knapp zwei Drittel** aller Probandinnen und Probanden empfinden sich in ihrem Wohnumfeld als **gering** dem Lärm von Baustellen **ausgesetzt**, etwas **mehr als 10%** als **stark ausgesetzt** (S. 184). Die Befragten in **Pradl** geben gemittelt **das größte Ausmaß** an empfundener Baulärmexposition an, die befragten Innsbruckerinnen und Innsbrucker **in Vill das mit Abstand niedrigste** (S. 185).
- Das **Lärmexpositionsempfinden** gegenüber der gegebenen **Schienenverkehrslärmbelastung** liegt im Vergleich der Verkehrsquellen **deutlich unterhalb der Werte für den Straßen- und Flugverkehrslärm** und beginnt erst bei höheren Lärmpegeln stärker anzusteigen (S. 173). Im Vergleich zum Flugverkehrslärm und etwas eingeschränkter auch zum Straßenverkehrslärm ist von einer **größeren Toleranz von Dauerschallpegeln unter 50dB** ausgehend vom Schienenverkehr auszugehen, bis Befragte für sich selbst eine mittelgradige oder darüber hinausgehende Lärmexposition aus dem Schienenverkehr annehmen (S. 197). Entsprechend der gegebenen Schienenverkehrslärmbelastung zeigt sich das mit Abstand **stärkste Schienenlärmexpositionsempfinden in Wilten**. Die **geringste Ausgesetztheit** gegenüber Schienenlärm im eigenen Wohnumfeld empfinden die Befragten **in Igls, Vill und Amras** (S. 197).

- Etwas **weniger als 2%** aller befragten Innsbruckerinnen und Innsbrucker sehen sich in ihrem Wohnumfeld **dem Autobahnlärm stark exponiert**, **über 90% nur gering**, davon **knapp 80% gar nicht** ausgesetzt (S. 199). Das im Mittel stärkste Autobahnlärmexpositionsempfinden zeigt sich **in Wilten, gefolgt von Vill**. Die **geringste Ausgesetztheit** gegenüber Autobahnlärm im eigenen Wohnumfeld empfinden die Befragten in der Innenstadt (Katastralgemeinde Innsbruck), **sowie in Igls, Pradl und Mühlau** (S. 200).

5.10. Belästigung / Störung durch Lärm

- Die **Belästigung / Störung durch den Flugverkehrslärm** wird von den Befragten **im Mittel am höchsten** eingeschätzt, **gefolgt von der Belästigung durch den Straßenverkehrslärm**. Die Belästigungswahrnehmung von **Baustellen**, die Störung durch **Nachbarschaftslärm** und der Lärm verursacht durch **Passanten und Gaststätten** folgen mit deutlichem Abstand. Wiederum in ihrer Ausprägung deutlich dahinter schließen sich **Schienenverkehrslärm** und die Störung durch **Erschütterungen** an. Die Belästigungsreaktion auf **Lärm aus Gewerbe und Industrie** und durch **Autobahnlärm** fällt im Vergleich noch einmal deutlich geringer aus (S. 204).
- **12,1%** aller befragten Innsbruckerinnen und Innsbrucker schätzen sich selbst als **vom Lärm insgesamt stark belästigt** ein, etwas mehr als die Hälfte (51,2%) antworten in den mittleren Kategorien und **36,7%** aller Probandinnen und Probanden sehen sich selbst als **nur gering lärmbelästigt** an (S. 210).
- Insgesamt **am stärksten vom Lärm belästigt / gestört** zeigen sich die befragten Innsbruckerinnen und Innsbrucker in **Wilten**, **gefolgt von Innsbruck, Pradl und Hötting**. In der Summe aller Lärmquellen am geringsten belästigt empfinden sich die Bewohnerinnen und Bewohner von **Igls, Vill und Mühlau** (S. 207).
- **Frauen** empfinden sich **stärker** vom Lärm **belästigt** als Männer, **ebenso die mittlere Altersgruppe der 41- bis 60-Jährigen** im Vergleich zu den Jüngeren und Älteren. Befragte, in deren Haushalt **minderjährige Kinder** leben, die zumeist bei **geöffnetem oder gekippten Fenster** schlafen, die in ihrem Wohnumfeld über eine **lärmabgewandte Seite** oder einen **Innenhof** verfügen, empfinden sich **weniger vom Lärm gestört**. Deutlich zeigen sich auch Beziehungen zwischen dem Ausmaß der Belästigung durch Lärm insgesamt und der Einschätzung und Zufriedenheit mit den eigenen Lebens- und Wohnbedingungen, den Bedingungen für das Aufwachsen von Kindern, oder auch der Verbundenheit zur Stadt Innsbruck. **Je geringer das persönliche Ausmaß der Belästigung durch Lärm bewertet wird, desto höher werden die eigenen Lebens- und Wohnbedingungen eingeschätzt und vice versa** (S. 212). Dasselbe gilt auch für die Beziehungen zur Einschätzung der eigenen **Lebensqualität** und **Lärmempfindlichkeit** und einer ganzen Reihe anderer Parameter: Befragte Innsbruckerinnen und Innsbrucker, die sich **stärker lärmbelästigt** als andere fühlen (S. 213),
    - beurteilen das **Lärmniveau in Innsbruck** im Vergleich zu anderen österr. Städten schlechter,
    - gehen häufiger davon aus, dass der **Lärm** in den letzten Jahren **zugenommen** hat,
    - schätzen die **negativen Wirkungen von Lärm auf die Gesundheit stärker** ein,
    - wenden **häufiger Lärmadaptationsmechanismen** an,
    - sind wesentlich **häufiger für (weitere) Maßnahmen zur Lärmreduktion**,
    - zeigen insgesamt in Bezug zu Beurteilungen der eigenen Wohn- und Lebensbedingungen eine **deutlich negativere Grundhaltung**.

Der starke Bezug zum Verkehrslärm zeigt sich auch in Zusammenschau mit der Frage nach baulichem Schutz vor demselben: **Stärker geschützt Befragte empfinden sich deutlich weniger lärmbelästigt** (S. 211).

- Der **Fluglärm** löst im befragten Kollektiv knapp vor dem Straßenverkehrslärm die **stärkste Belästigungsreaktion** aus. **11,7%** ordnen sich der Gruppe der **Starkbelästigten** zu, 39% antworten in den mittleren Belästigungskategorien, **knapp die Hälfte** aller Befragten schätzen sich als **gering vom Fluglärm belästigt** ein (S. 215). Entsprechend der gegebenen Fluglärmbelastung zeigt sich das mit Abstand **stärkste Fluglärmbelästigungsempfinden** in **Hötting**. Die **geringste Störung** durch Fluglärm nehmen die Befragten **in Igls** wahr (S. 216).
- Die **Belästigung durch Straßenverkehrslärm** fällt in ihrem Ausmaß sehr **ähnlich** derjenigen auf **Fluglärm** aus und fällt in ihren Ausprägungen, auch in Belastungsgruppen kategorisiert, nur sehr geringfügig hinter den Flugverkehrslärm zurück. Die Anzahl an Personen, die in der höchstmöglichen Antwortkategorie 10 (=*belästigt / stört besonders stark*) antwortet, ist mit 3,5% sogar etwas höher als beim Fluglärm. **Mehr als die Hälfte** aller Befragten schätzt sich selbst als nur **gering** vom Straßenverkehrslärm **gestört** ein. Ähnlich wie beim Flugverkehrslärm fühlt sich **jede(r) 9. Befragte stark vom Straßenverkehrslärm belästigt** (S. 219). Im Unterschied zur Belästigung durch Fluglärm steigt das Belästigungsausmaß in Bezug zum Straßenverkehrslärm erst bei höheren gegebenen Lärmpegeln über 50dB L_{den} stark an (S. 220). Das **größte Ausmaß** an Belästigung durch Straßenverkehrslärm wird in Wilten empfunden, gefolgt von Pradl, Innsbruck und Amras. Die **geringste Störung** durch Straßenverkehrslärm empfinden die Befragten **in Igls, Mühlau und Vill** (S. 220).
- Das Ausmaß der Belästigung durch Verkehrslärm zeigt einen deutlichen Zusammenhang zur gegebenen Lärmbelastung: **Je höher die Verkehrslärmbelastung, desto höher die Belästigung**. Allerdings unterscheiden sich die Belästigungsreaktionen in ihrer Ausprägung je nach Verkehrslärmquelle stark voneinander. **Flugverkehrslärm** wird in allen Belastungskategorien als **störender** eingestuft **als** der **Straßenverkehrslärm und** dieser wiederum als deutlich belästigender als der **Schienenverkehrslärm** (S. 206). Wird das gemittelte Belästigungsausmaß nach vorhandener Lärmbelastung in 10dB-Schritten (24-Pegel L_{den}) nach Verkehrslärmquelle verglichen, wird deutlich, dass das Belästigungsausmaß beim **Straßen- und Schienenverkehr** erst bei Dauerschallpegeln über 50dB stärker zu steigen beginnt, bis zu diesen Pegeln jedoch von den Befragten relativ niedrig eingeschätzt wird. Ab diesen **„Schwellenwerten“** steigt das Belästigungsausmaß stark an (S. 206). **Für den Flugverkehrslärm lässt sich diese Schwelle nicht festmachen.** Das Belästigungsausmaß liegt bereits bei niedrigen Dauerlärmpegeln höher als beim Straßen- und Schienenverkehrslärm und steigt linearer an (S. 206).
- Wie schon bei der Einschätzung der Lärmexposition der eigenen Wohnung / des eigenen Hauses fällt die Belästigungsreaktion durch Baustellenlärm und die folgenden Belästigungsreaktionen deutlich anders aus, als die Störungswahrnehmung durch Flug- und Straßenverkehrslärm. **Fast die Hälfte** aller befragten Innsbruckerinnen und Innsbrucker ***stört* der Baustellenlärm** in der Stadt ***überhaupt nicht*** und fast zwei Drittel (65%) aller Probandinnen und Probanden sehen sich selbst als nur gering vom Baustellenlärm belästigt an. **Über 9%** sehen sich hingegen als **stark belästigt** und rund ein Viertel der Befragten (25,6%) antwortet in den mittleren Kategorien (S. 224). Die Befragten **in Pradl** geben **gemittelt das größte Ausmaß an Belästigung** durch Baustellenlärm an, die befragten Innsbruckerinnen und Innsbrucker in **Vill** das mit Abstand **niedrigste** (S. 224). Die Belästigung ausgehend von Baustellenlärm ist zum einen häufig ein zeitlich begrenztes Ärgernis und zum anderen zumindest teilweise unabhängig von anderen Wohn- und Lebens-

bedingungen. Das spiegelt sich auch in den Beantwortungen der einzelnen Befragten wieder. Die Unterschiede zwischen einzelnen Subgruppen fallen vergleichsweise kleiner aus und streuen stärker als bei den rein verkehrsinduzierten Lärmquellen (S. 225).

- **Über 70%** aller Befragten fühlen sich **durch Nachbarschaftslärm gar nicht oder nur geringfügig gestört**, **5%** hingegen **stark belästigt** (S. 226). In **Innsbruck, Wilten und Pradl** fühlen sich die Befragten **am stärksten** vom Nachbarschaftslärm belästigt, in **Igls, Vill und Mühlau am wenigsten** (S. 227). Aus den Auswertungen ist ersichtlich, dass Nachbarschaftslärm und die daraus resultierende Belästigung, sofern diese als stark wahrgenommen wird, sich ganz erheblich auf eine ganze Reihe von Einschätzungen zu den persönlichen **Wohn- und Lebensbedingungen** auswirkt. Diese werden **von den stärker vom Nachbarschaftslärm belästigten** befragten Innsbruckerinnen und Innsbruckern **deutlich schlechter** eingestuft, als von sich nur geringfügig lärmgestört Empfindenden. Gleiches gilt, jeweils wechselseitig, auch für die Einschätzung der eigenen Lärmempfindlichkeit und Lebensqualität (S. 228).

- Die **Belästigungsreaktion auf den Lärm von Passanten und Gaststätten** ist nahezu deckungsgleich mit derjenigen auf Nachbarschaftslärm. **4,8%** fühlen sich **ganz erheblich gestört**, **72,6%** hingegen **nur sehr geringfügig** (davon knapp 53% *überhaupt nicht*) (S. 229). Das Empfinden der eigenen Belästigung durch den Lärm von Passanten und Gaststätten **unterscheidet sich** am deutlichsten aller Belästigungsfragen **zwischen** den einzelnen **Katastralgemeinden** und ist in Abhängigkeit von der Anzahl der vorhandenen Lokale, Sehenswürdigkeiten, der Bevölkerungsdichte etc. zu sehen, wodurch sich die inneren Stadtbezirke von den äußeren abheben (S. 230).

- Deutlich **über zwei Drittel** aller befragten Innsbruckerinnen und Innsbrucker ***stört* der Schienenverkehrslärm in ihrem Wohnumfeld *überhaupt nicht*** und **über 80%** aller Probandinnen und Probanden sehen sich selbst als **nur gering** vom Schienenverkehrslärm **belästigt**. **Rund 3%** sehen sich hingegen als **starkbelästigt** und 15,6% der Befragten antworten in den mittleren Kategorien (S. 232). **Im Vergleich mit der Belästigung durch den Straßenverkehrslärm und insbesondere mit dem Flugverkehrslärm ist das deutlich geringere Belästigungsausmaß durch den Schienenverkehrslärm auffällig.** Diese geringere Störung der befragten Innsbruckerinnen und Innsbrucker durch den Schienenverkehrslärm spiegelt sich auch in der Zusammenschau der Belästigung durch den Schienenverkehrslärm mit der gegebenen Schienenlärmbelastung. Das **Belästigungsausmaß** im niederen Belastungsbereich fällt sehr gering aus und **steigt erst bei höheren gegebenen Lärmpegeln über 50dB L_{den} stärker an**. Das Ausmaß der empfundenen Störung durch den Schienenverkehrslärm deckt sich dabei nahezu 1:1 mit der empfundenen Ausgesetztheit gegenüber demselben. Lediglich in der höchsten Belastungskategorie über 60dB-Dauerbelastung (24h-L_{den}) wird – wie schon beim Flug- und beim Straßenverkehrslärm – die Ausgesetztheit von den Befragten höher angesetzt als die Belästigung (S. 233). Entsprechend der gegebenen Schienenverkehrslärmbelastung zeigt sich die **stärkste Schienenlärmbelästigung in Wilten und Innsbruck**. Die **geringste Störung** durch Schienenlärm im eigenen Wohnumfeld empfinden die Befragten **in Igls, Vill und Amras** (S. 233).

- Die **Belästigungsreaktion auf Erschütterungen** ist in ihren Dimensionen der auf Schienenverkehrslärm sehr ähnlich. Über 70% werden von Erschütterungen *überhaupt nicht gestört*, **83,6%** des befragten Kollektivs sind der Kategorie der **Geringbelästigten** zuzuordnen, wohingegen **3,3%** sich selbst als **stark belästigt** einschätzen (S. 236). Das **größte Ausmaß an Belästigung** durch Erschütterungen wird **in Wilten und Innsbruck** empfunden, **das geringste in Vill und Mühlau** (S. 236).

- **1,5%** aller Befragten fühlen sich durch den L**ärm verursacht von Gewerbe und Industrie stark belästigt**, **über 90%** hingegen **nur geringfügig gestört**, unter diesen über 80% *überhaupt nicht* (S. 238). Die Störung durch Lärm von Gewerbe und Industrie ist **in Mühlau und Vill am geringsten** und **in Innsbruck, Wilten und Arzl am stärksten** ausgeprägt (S. 239).
- Die **Belästigung durch Autobahnlärm** im urbanen Stadtgebiet von Innsbruck fällt im befragten Kollektiv **vergleichsweise gering** aus und nimmt unter allen 10 abgefragten Lärmquellen die geringsten Ausprägungen an. 2,1% empfinden sich als stark belästigt, 91,9% hingegen **als *überhaupt nicht* bis gering vom Autobahnlärm gestört** (S. 241). Sehr ähnlich dem Bezug zum Straßenverkehrslärm ist das Belästigungsausmaß im niederen Belastungsbereich sehr gering und steigt bei höheren gegebenen Lärmpegeln über 50dB L_{den} stark an (S. 242). Die im Mittel **stärkste Belästigung** durch Autobahnlärm zeigt sich **in Wilten, gefolgt von Vill**. Die **geringste Störung** durch Autobahnlärm im eigenen Wohnumfeld empfinden die Befragten **in der Innenstadt** (Katastralgemeinde Innsbruck), **sowie in Igls, Pradl und Mühlau** (S. 243).

5.11. Belästigung / Störung durch Lärm im Schlaf

- Im Mittelwertvergleich der einzelnen Lärmquellen verändert sich das Bild der Belästigungsreaktion auf Lärm im Schlaf im Vergleich zur Belästigungsausprägung auf Lärm im 24h-Zeitraum: Zunächst ist festzuhalten, dass sich das **Ausmaß der Belästigung im Nachtzeitraum in etwa halbiert**. Des Weiteren rückt der **Straßenverkehrslärm an** die **erste Stelle als störendste Lärmquelle**, **gefolgt vom verursachten Lärm durch Passanten und Gaststätten und dem Nachbarschaftslärm**. Der **Flugverkehrslärm fällt an die vierte Stelle, der Baustellenlärm an die fünfte Stelle zurück**. Erschütterungen, Lärm von Gewerbe und Industrie und Autobahnlärm verbleiben in dieser Reihenfolge auf den Plätzen, der am wenigsten belästigendsten Lärmquellen (S. 245).
- **7,8%** aller befragten Innsbruckerinnen und Innsbrucker schätzen sich selbst als vom Lärm insgesamt im Schlaf stark belästigt ein, knapp ein Viertel antwortet in den mittleren Kategorien und **über zwei Drittel** aller Probandinnen und Probanden sehen sich selbst als **nur gering im Schlaf lärmbelästigt** an, **davon über 45% *überhaupt nicht belästigt*** (S. 251).
- Das Ausmaß der Belästigung durch Verkehrslärm zeigt auch für den Nachtzeitraum (22h bis 6h) einen starken Zusammenhang zur gegebenen Lärmbelastung: **Je höher die Verkehrslärmbelastung, desto höher die Belästigung.** Die Unterschiede zwischen den drei Verkehrslärmquellen Straße – Schiene – Flugverkehr nivellieren sich jedoch. Aufgrund der geringeren Flugverkehrslärmbelastung der Innsbruckerinnen und Innsbrucker in der Nacht ist diese im gruppierten Vergleich nur für die Geringbelasteten <45dB sinnvoll heranzuziehen, nimmt in dieser Belastungsgruppe aber auch in den Nachtstunden den Platz als störendste Lärmquelle ein. **Die im Schlaf störendste Verkehrslärmquelle insgesamt ist der Straßenverkehr. Der für den 24h-Zeitraum deutliche Abstand zum Belästigungsausmaß des Schienenverkehrs verringert sich** (S. 247).
- Im Vergleich des gemittelten Belästigungsausmaßes im Schlaf nach vorhandener Lärmbelastung in 10dB-Schritten (Nachtlärmpegel L_n) nach Verkehrslärmquelle zeigt sich erneut das höhere Belästigungsausmaß des Flugverkehrslärms gegenüber dem Straßen- und Schienenverkehrslärm. Auch ist erkennbar, dass die **Belästigungsreaktion** auf Verkehrslärm sich unabhängig von der Quelle bereits **bei deutlich niedrigeren Lärmpegeln** zu verstärken beginnt und kontinuierlicher ansteigt als in Zusammenschau mit den 24h-Pegeln. Ein **„Schwellenwert“** ab dem eine gesteigerte Belästigung / Störung durch den Verkehrslärm eintritt, ist für die Belästigung im Schlaf **nicht** auf den ersten Blick **auszumachen** (S. 247).

- Insgesamt **am stärksten im Schlaf vom Lärm belästigt / gestört** zeigen sich die Befragten in **Wilten und Innsbruck** mit deutlichem Abstand gefolgt von Pradl und Hötting. In der Summe aller Lärmquellen **am geringsten belästigt** empfinden sich die Bewohnerinnen und Bewohner von **Igls, Vill und Mühlau** (S. 248).
- **Frauen** empfinden sich insgesamt **deutlich stärker vom Lärm im Schlaf belästigt als Männer**, insbesonders in der Extremgruppe der sich stark im Schlaf gestört Empfindenden (S. 252). Ähnliches gilt für die **mittlere Altersgruppe** der 41- bis 60-Jährigen im Vergleich zu jüngeren und älteren Befragten. Der höchste erreichte Schulabschluss korrespondiert ebenfalls mit dem Belästigungsausmaß durch Lärm insgesamt im Schlaf: **Akademikerinnen und Akademiker empfinden sich überdurchschnittlich, Pflichtschulabgängerinnen und –Abgänger hingegen unterdurchschnittlich stark belästigt.** Befragte, in deren Haushalt minderjährige Kinder leben, die zumeist bei geöffnetem oder gekippten Fenster schlafen (S. 254), die in ihrem Wohnumfeld über eine lärmabgewandte Seite, einen Innenhof oder baulichen Lärmschutz verfügen, empfinden sich auch im Schlaf weniger vom Lärm gestört. Erkennbar sich auch **Beziehungen** zwischen dem Ausmaß der Belästigung im Schlaf durch Lärm insgesamt und der **Einschätzung und Zufriedenheit mit den eigenen Lebens- und Wohnbedingungen** (S. 253), den Bedingungen für das Aufwachsen von Kindern etc. Die Bezüge sind allerdings geringer ausgeprägt als für die Belästigung durch Lärm im 24h-Zeitraum. Dasselbe gilt auch für die Beziehungen zur Einschätzung der eigenen Lebensqualität (S. 254) und Lärmempfindlichkeit und einer ganzen **Reihe anderer Parameter** (S. 254): Befragte Innsbruckerinnen und Innsbrucker, die sich im Schlaf lärmbelästigt fühlen,
  - beurteilen das Lärmniveau in Innsbruck im Vergleich zu anderen österr. Städten schlechter,
  - gehen häufiger davon aus, dass der Lärm in den letzten Jahren zugenommen hat,
  - schätzen die negativen Wirkungen von Lärm auf die Gesundheit stärker ein,
  - wenden häufiger Lärmadaptationsmechanismen an,
  - sind wesentlich häufiger für (weitere) Maßnahmen zur Lärmreduktion,
  - zeigen insgesamt in Bezug zu Beurteilungen der eigenen Wohn- und Lebensbedingungen eine deutlich negativere Grundhaltung.
- Durch den **Straßenverkehrslärm** im Schlaf **stark belästigt** empfinden sich **5,4%** aller Befragten, mehr als die Hälfte aller Befragten nimmt keine Belästigung im Schlaf wahr und insgesamt ordnen sich **über drei Viertel** aller Befragten der Gruppe der **gering im Schlaf Lärmbelästigten** zu (S. 256). In der Zusammenschau der Belästigung durch Straßenverkehrslärm im Schlaf mit der gegebenen nächtlichen Straßenverkehrslärmbelastung zeigt sich ein dem 24h-Zeitraum sehr vergleichbares, etwas kontinuierlicheres Ansteigen des Belästigungsausmaßes. Ein „Schwellenwert" ab welchem ein stärkeres Ansteigen der Belästigungsreaktion auftritt, ist im Gegensatz zum 24h-Zeitraum nicht eindeutig festzumachen (S. 257). Das **größte Ausmaß an Belästigung** durch Straßenverkehrslärm im Schlaf wird mit einigem Abstand **in Wilten** empfunden, gefolgt von Innsbruck, Pradl und wiederum mit einigem Abstand Hötting. Die **geringste Störung** durch Straßenverkehrslärm im Schlaf im eigenen Wohnumfeld empfinden die Befragten **in Vill und Igls** (S. 257). Spannend sind Bezüge einzelner Fragestellungen zur Belästigung im Schlaf durch Straßenverkehrslärm (wie auch für andere Lärmquellen) in Abhängigkeit von der vorhandenen nächtlichen Straßenverkehrslärmbelastung. So wird etwa die (beabsichtigt) diffus gestellte Frage nach der Einschätzung der Güte der Sicherheit in Innsbruck von stärker belasteten und belästigten Befragten deutlich schlechter eingestuft, als von weniger Lärmbelasteten und -Belästigten (S. 259).
- Die wahrgenommene **Belästigung im Schlaf durch den Lärm verursacht von Passanten und Gaststätten** ist in ihren Ausprägungen **nahezu deckungsgleich** mit jener durch den Straßenver-

kehrslärm. Im Mittel liegen die Antworten der befragten Innsbruckerinnen und Innsbrucker jedoch unterhalb der Werte vom Straßenverkehrslärm (S. 261). Die Belästigung im Schlaf durch den Lärm von Passanten und Gaststätten ist **in den inneren Stadtbezirken naturgemäß größer** und in Vill, Igls und Mühlau de facto nicht existent (S. 261).

– Die **Belästigung im Schlaf durch Nachbarschaftslärm** steht a**uf Platz drei der am meisten störenden Lärmquellen im Nachtzeitraum**. **4,3%** aller Probandinnen und Probanden schätzen sich als **stark** vom Nachbarschaftslärm im Schlaf **belästigt** ein, **über 80%** als **gering belästigt**, davon knapp zwei Drittel *überhaupt nicht gestört* (S. 264). Die Belästigung durch Nachbarschaftslärm im Schlaf nach Katastralgemeinden unterscheidet sich vom 24h-Zeitraum: Liegen Innsbruck, Wilten und Pradl im Antwortverhalten der Befragten im 24h-Zeitraum nahezu gleichauf, so sehen sich die befragten **Wiltenerinnen und Wiltener im Nachtzeitraum stärker** vom Nachbarschaftslärm **im Schlaf gestört** als die Einwohnerinnen und Einwohner der Katastralgemeinde Innsbruck und diese wiederum stärker als die Pradlerinnen und Pradler. **Am wenigsten** vom Nachbarschaftslärm **im Schlaf gestört** empfinden sich die Befragten in **Vill und Igls** (S. 265). Die Störung durch Nachbarschaftslärm in der Nacht zeigt vergleichsweise **stark ausgeprägte Beziehungen** zu den Fragen **zu den eigenen Wohn- und Lebensbedingungen** (S. 265).

– Die **Belästigung im Schlaf durch** den nächtlichen **Flugverkehrslärm** ist **in den einzelnen – vergleichsweise niederen – Lärmbelastungsklassen** im Unterschied zur empfundenen Störung im Schlaf durch den nächtlichen Straßen- und Schienenverkehrslärm **in etwa gleich hoch wie im 24h-Zeitraum und steigt sehr linear mit zunehmender Belastung an** (S. 268). Im Nachtzeitraum sind **keine Befragten mit Dauerschallpegeln über 50dB L_n aus dem Flugverkehr belastet**, weshalb das **Belästigungsempfinden** durch Flugverkehrslärm **in der Nacht insgesamt vergleichsweise gering** ausgeprägt ist: **Knapp 4%** aller befragten Innsbruckerinnen und Innsbrucker fühlen sich im Schlaf durch Flugverkehrslärm **stark gestört**, **knapp 85%** aller Probandinnen und Probanden sehen sich selbst als **nur gering im Schlaf fluglärmbelästigt** an, davon über 70% *überhaupt nicht belästigt* (S. 267). Entsprechend der gegebenen Fluglärmbelastung zeigt sich auch in der Nacht das **stärkste Fluglärmbelästigungsempfinden in Hötting**. Die **geringste Störung** durch Fluglärm im Schlaf nehmen die Befragten **in Igls, Mühlau und Vill** wahr (S. 268).

– Für die Nachtstunden ist nicht von ausgeprägten Baustellentätigkeiten auszugehen, was sich auch in der Beantwortung durch die Befragten nach der Belästigung im Schlaf durch Baustellenlärm widerspiegelt: **Knapp 90%** aller Befragten sehen sich ***überhaupt nicht* bis gering von Baustellenlärm im Schlaf gestört**, **2,1%** gehören hingegen den im Schlaf **stark Lärmbelästigten** an (S. 271).

– **Keine Belästigung durch Schienenverkehrslärm im Schlaf** nehmen **über 83%** der Befragten wahr, insgesamt sind über 90% *überhaupt nicht* bis gering lärmbelästigt. **1,5%** sehen sich als **stark** vom Schienenverkehrslärm **im Schlaf gestört** an (S. 273). Eine **Belästigung durch den Schienenverkehrslärm im Schlaf** ist bis zu Dauerbelastungen von 40dB im Wohnumfeld der Befragten kaum vorhanden und fällt auch bei nächtlichen Dauerlärmpegeln über 50dB **vergleichsweise gering** aus. Eine **steigende Belästigungsreaktion (auf niedrigem Niveau) beginnt bei etwa 10dB niedrigeren Lärmpegeln als im 24h-Zeitraum einzusetzen** (S. 274). Entsprechend der gegebenen Schienenverkehrslärmbelastung zeigt sich auch für den Nachtzeitraum die **stärkste Schienenlärmbelästigung in Innsbruck und Wilten**. Die **geringste Störung** durch Schienenlärm **im Schlaf** im eigenen Wohnumfeld empfinden die Befragten **in Igls und Vill**, wo - wie schon für den 24h-Zeitraum - fast keine Belästigung durch den Schienenverkehrslärm von den Befragten festgestellt wird (S. 275).

- Die **Belästigungsausprägungen durch Erschütterungen im Schlaf** sind in ihren Dimensionen **nahezu deckungsgleich jenen des Schienenverkehrslärms** und fallen gering aus (S. 277).

- Eine **Belästigung in den Nachtstunden durch Lärm aus Gewerbe und Industrie** ist **für die überwiegende Mehrheit** der Befragten in Innsbruck **nicht vorhanden**. Mehr als 90% sehen sich überhaupt nicht gestört, rund ein Prozent (10 von 1031 befragten Personen) fällt in die Gruppe der stark vom Lärm aus Gewerbe und Industrie im Schlaf Belästigten (S. 280).

- Autobahnlärm spielt wie schon bei der Frage nach der Belästigung allgemein auch im Nachtzeitraum für nur sehr wenige befragte Innsbruckerinnen und Innsbrucker eine größere Rolle. Nur **rund 1%** der 1031 Befragten **stört der Autobahnlärm im Schlaf stark**, knapp 96% aller Befragten sehen sich selbst als nur gering im Schlaf autobahnlärmbelästigt an, davon **fast 92% *überhaupt nicht belästigt*** (S. 283). Das Belästigungsausmaß im niederen Belastungsbereich ist sehr gering ausgeprägt, beginnt aber im Vergleich zum 24h-Zeitraum bei rund 10dB niedrigeren Dauerlärmpegeln, ab Belastungen von etwa 40dB L_n, auf niedrigem Niveau anzusteigen (S. 283). Die im Mittel **stärkste Belästigung im Schlaf** durch Autobahnlärm zeigt sich **in Wilten und Vill**. Die **geringste Störung** durch Autobahnlärm in der Nacht im eigenen Wohnumfeld empfinden die Befragten **in Igls, Mühlau und Pradl, sowie in der Innenstadt** (S. 284).

5.12. Lärmcoping

- Lärmcopingmechanismen als **Reaktionen auf und zur Bewältigung von Lärmbelastungen** wurden in Ansätzen erhoben (aktives und passives instrumentelles, emotionales und politisches Copingverhalten). Über alle 7 Fragen hinweg (Summenscore) zeigen **23,5%** der Befragten ein **ausgeprägtes Lärmadaptationsverhalten, rund drei Viertel nicht** (S. 288).

- Die befragten Innsbruckerinnen und Innsbrucker haben wegen der Belästigung durch Lärm schon zumindest einmal (S. 287):
  - **Sich geärgert:** **über 55%**
  - **Die Fenster auch im Sommer nachts geschlossen:** **knapp 46%**
  - **Gespräche mit Nachbarn geführt:** **über 34%**
  - **Sich hilflos gefühlt:** **knapp 24%**
  - **Den Garten oder Balkon nicht genutzt:** **über 21%**
  - **Ihre Wohnräume anders als sonst genutzt:** **15,4%**
  - **Gespräche mit Behörden geführt:** **13%**

- Eine Reihe von Faktoren zeigt **Zusammenhänge** zum Lärmcoping. Unter anderem die vorhandene **Lärmbelastung**, die **Lärmempfindlichkeit** und das **Gefühl der Ausgesetztheit** gegenüber Verkehrslärm, sowie das **Belästigungsempfinden**. Sehr deutlich treten die Unterschiede im Verhalten der befragten Innsbruckerinnen und Innsbrucker auch in Abhängigkeit von **baulichem Lärmschutz** in deren Wohnumfeld zutage (vgl. z. Bsp. S. 289).

- **Höhere gegebene Verkehrslärmbelastungen führen zu deutlich steigendem Lärmcopingverhalten** (S. 294). In der Zusammenschau mit den gegebenen Verkehrslärmpegeln werden sehr deutliche **Unterschiede zwischen den einzelnen Belastungsgruppen** sichtbar: Knapp ein Drittel der vom 24h-Straßenlärm stark Belasteten hat sich schon zumindest einmal hilflos gegenüber dem Verkehrslärm gefühlt. In der Gruppe der vom Straßenlärm gering belasteten Befragten sind es etwas weniger als 9%. Beim Flug- und Schienenverkehrslärm fällt auf, dass die Gruppe der gering Belasteten deutlich stärker reagiert, als auf die Lärmbelastung durch den Straßenverkehr und

beim Fluglärm die mittelgradig belasteten Befragten sogar häufiger emotionales Coping zeigen, als die Starkbelasteten. Die Unterschiede zwischen den einzelnen Belastungsgruppen fallen beim Flug- und Schienenverkehrslärm insgesamt geringer aus (S. 290).

- **Weibliche Befragte reagieren geringfügig häufiger** auf Lärm als Männer. Die jüngsten Befragten zeigen das geringste Lärmadaptationsverhalten, die **mittlere Altersklasse** der 41- bis 60-Jährigen das am deutlichsten ausgeprägte. **Je höher der erreichte höchste Schulabschluss, desto häufiger geben Befragte an, Lärmbewältigungsmechanismen einzusetzen**. Befragte, welche durch bauliche Abschirmungen (Gebäude, Mauern, Innenhöfe …) zumindest zum Teil vor Verkehrslärm geschützt wohnen, haben weniger **Lärmcopingbedarf**. Befragte, welche der Meinung sind, in Innsbruck sei es lauter oder gleich laut wie in anderen vergleichbaren Städten Österreichs, zeigen ein stärkeres Lärmadaptationsverhalten als Befragte, die glauben, dass es in Innsbruck ruhiger als in anderen Städten sei (S. 294).
- **Noch stärker als in Zusammenschau mit der gegebenen Lärmbelastung steigt die Häufigkeit der eingesetzten Lärmadaptationsmechanismen mit zunehmender empfundener Lärmbelästigung** (S. 295). Die einzelnen Quellen der Verkehrslärmbelästigung (Straßen-, Flug- oder Schienenverkehrslärm) zeigen im Summenscore dabei keine ausgeprägten Abweichungen voneinander. Ähnliches gilt für die nicht verkehrsverursachte Lärmbelästigung: Im Vergleich der Extremgruppen fällt bei der Belästigung durch Erschütterungen, Nachbarschaftslärm und Lärm aus Gewerbe und Industrie ein stärker ausgeprägtes Lärmcopingverhalten als bei den anderen Belästigungslärmquellen auf (S. 296).
- **Fühlen sich Befragte durch Lärm in der Nacht im Schlaf gestört, fallen die Lärmbewältigungsmechanismen stärker aus** und werden bereits bei niedrigeren Belästigungsausmaßen gesetzt als in der Zusammenschau mit der Belästigung durch den Lärm insgesamt (S. 297).

5.13. Empfundene Zu- oder Abnahme des Lärmbelastungen in Innsbruck

- Eine **deutliche Mehrheit** der befragten Innsbruckerinnen und Innsbrucker ist **der Meinung, dass die Lärmbelastungen** in der Stadt in den letzten Jahren **nicht zugenommen haben (57,7%)**, über 42% hingegen gehen hingegen davon aus, dass die Lärmbelastungen zugenommen haben. Mit **knapp 95%** relativ einig sind sich die Befragten dahingehend, dass die **Lärmbelastungen** in den letzten Jahren **nicht abgenommen** haben (S. 298).
- Die **gegebene Lärmbelastung** durch die Verkehrsträger **zeigt keine ausgeprägten Beziehungen** zur Einschätzung der Befragten, ob sich die Lärmbelastung in Innsbruck in den vergangenen Jahren verändert hat. Deutlich stärker korrespondieren Faktoren wie die Einschätzung der eigenen **Lebensqualität**, die **Lebens- und Wohnbedingungen**, die eigene **Lärmempfindlichkeit** und das Gefühl der **Ausgesetztheit** gegenüber Lärm (S. 298).
- Die empfundene Störung durch Lärm zeigt mit steigendem Ausmaß unabhängig von der Quelle der Lärmbelästigung in Zusammenschau mit der Einschätzung der Lärmbelastungsveränderung in Innsbruck sehr einheitlich eine deutlich steigende Tendenz der Befragten, von einer gefühlten Zunahme der Lärmbelastung auszugehen. **Rund 70% der Starkbelästigten gehen relativ unabhängig von der Quelle der Belästigung** (Ausnahme Schienenverkehrslärmbelästigung) **von Lärmzunahmen aus**, während es bei den geringbelästigten Befragten im Mittel nur ein Drittel ist (S. 300).

5.14. Einschätzung der Negativwirkung von Lärm auf die Gesundheit

- Mehr als **drei Viertel** (75,8%) der befragten Innsbruckerinnen und Innsbrucker **gehen davon aus, dass sich Lärm *eher stark* bis *sehr stark* negativ auf die Gesundheit auswirkt**, 21% nehmen an, dass Lärmbelastungen nur geringe Gesundheitswirkungen aufweisen (S. 302).
- **Männer gehen** von einer deutlich **geringeren negativen Wirkung** von Lärm auf die Gesundheit **aus** als Frauen, während das **Alter** der Befragten **kaum Einflüsse** zeigt. Die vorhandene Lärmbelastung zeigt nur beim Straßenverkehrslärm Beziehungen zur Einschätzung der negativen Wirkung von Lärm auf die Gesundheit auf, wobei diese vergleichsweise gering ausgeprägt sind. Deutlich stärker fallen die Korrelationen zum Ausmaß der **Lärmempfindlichkeit**, dem Gefühl der **Ausgesetztheit** gegenüber Lärm, sowie den **Belästigungsauswertungen** aus (S. 303).
- Stark unterschiedlich antworten Befragte in Abhängigkeit von ihrem höchsten bisher erreichten Schulabschluss: **Von den Akademikerinnen und Akademikern gehen über 86% davon aus, dass Lärm starke negative Auswirkungen auf die Gesundheit hat, während bei den Pflichtschulabgängerinnen und –Abgängern etwas mehr als 61% dieser Meinung sind** (S. 303).
- Befragte, welche davon ausgehen, dass die negativen Wirkungen von Lärm auf die Gesundheit stark ausgeprägt sind, sind auch wesentlich häufiger der Ansicht, dass **(weitere) Maßnahmen zur Lärmreduktion in Innsbruck** getroffen werden sollten und umgekehrt (S. 304).

5.15. Einfluss von Wind und Wetter auf die Lärmbelastung

- **Zwei Drittel** aller befragten Innsbruckerinnen und Innsbrucker **glauben, dass die Wind- und Wetterverhältnisse sich g*ar nicht* oder *eher wenig* auf die Lärmbelastung auswirken**. 38 Befragte geben an, dass sie diese Frage nicht beurteilen können und **30%** der Probandinnen und Probanden sind der Meinung, dass sich die Wind- und Wetterverhältnisse ***eher stark* bis *sehr stark*** auf die Lärmbelastung insgesamt auswirken (S. 163).

5.16. Bekanntheit der Umgebungslärmkarten

- Etwas mehr als **21%** aller Befragten haben **schon einmal von Umgebungslärmkarten gehört**, oder sich diese sogar angesehen. **Mehr als drei Viertel** der Probandinnen und Probanden sind diese **unbekannt** (S. 305).
- Die Bekanntheit der Umgebungslärmkarten nimmt mit zunehmendem **Alter** leicht ab. **Männer** geben doppelt so häufig wie Frauen an, dass ihnen Umgebungslärmkarten bekannt sind. Je höher der höchste bisher erreichte **Schulabschluss**, desto eher haben Befragte schon von Umgebungslärmkarten gehört oder sich diese angesehen. **Befragte, die angeben, schon zumindest einmal mit Behörden über die Lärmbelastung gesprochen zu haben, kennen die Umgebungslärmkarten nicht häufiger, als jene, die das noch nie getan haben.** Befragte, die (weitere) Maßnahmen zur Lärmreduktion in Innsbruck für sinnvoll halten, geben deutlich häufiger an, von Umgebungslärmkarten schon gehört oder sich diese angesehen zu haben (S. 305).

5.17. Einschätzung des Lärmniveaus von Innsbruck im Städtevergleich

– Bei der Frage, wie laut es in Innsbruck im Vergleich zu anderen Städten in Österreich ist, gehen die Meinungen der Befragten auseinander. Während etwas mehr als **28% der Meinung** sind, **es wäre in Innsbruck *ruhiger* bis *viel ruhiger***, gehen **knapp 17%** davon aus, dass es ***lauter* bis *viel lauter*** sei. Etwas mehr als die **Hälfte** der Befragten **glaubt**, dass das **Lärmniveau in Innsbruck** in etwa dem anderer österreichischer Städte **entspricht** (S. 306). **Im Mittel gehen die Befragten davon aus, dass es Innsbruck in etwa *gleich ruhig oder laut* im Vergleich zu anderen österreichischen Städten ist, mit leichter Tendenz, dass es etwas ruhiger sei** (S. 306). Einzelne bivariate Auswertungen finden sich auf S. 308.

5.18. Einschätzung des eigenen Lärmbeitrags zur Gesamtlärmbelastung

– **Der eigene Beitrag zur Gesamtlärmbelastung in Innsbruck wird von den Befragten größtenteils als *sehr gering* bis *gering* eingeschätzt (75,2%). Nur 2,8% sind der Meinung, Sie selber tragen *stark* oder *sehr stark* zur Lärmbelastung bei.** 22% gehen davon aus, ihr eigener Lärmbeitrag sei *durchschnittlich*. Im Mittel gehen die befragten Innsbruckerinnen und Innsbrucker davon aus, ihr eigener Beitrag zum Lärm in Innsbruck sei *gering* (S. 310).

– Wird das **Mobilitätsverhalten** der Befragten nach der Häufigkeit der Nutzung einzelner Verkehrsmittel aufgeschlüsselt, können die Unterschiede in der Empfindung des eigenen Lärmbeitrages zur Gesamtlärmsituation in Innsbruck verfeinert werden: Diejenigen Befragten, die sich regelmäßig mit **öffentlichen Verkehrsmitteln** bewegen, glauben (ohne Adjustierung anderer Prädiktoren und Kovariablen) den **geringsten Beitrag** zum Gesamtlärm beizusteuern, (fast) tägliche **Autofahrerinnen und Autofahrer den größten**. Die Unterschiede zwischen diesen beiden Extremgruppen fallen sehr deutlich aus. Zwischen regelmäßigen Fußgängerinnen und Fußgängern und den (fast) täglichen Nutzerinnen und Nutzern von Fahrädern bestehen kaum Unterschiede. Auch die gelegentlichen Nutzerinnen und Nutzer von Flugzeugen erkennen für sich keinen per se größeren Lärmbeitrag (S. 312).

5.19. Einschätzung der störendsten Lärmquelle in Innsbruck

– **Fast 40%** im befragten Kollektiv nehmen an, dass der **Straßenverkehrslärm** die Innsbruckerinnen und Innsbrucker insgesamt am meisten belästigt oder stört. **Mit über 15 Prozentpunkten Abstand folgt der Flugverkehrslärm an zweiter Stelle, dicht gefolgt vom Baustellenlärm**. Die Lärmwirkungen ausgehend von Passanten und Gaststätten, Nachbarschaft, Schienenverkehr und Gewerbe und Industrie folgen mit sehr großem Abstand und werden nur von vergleichsweise wenigen bis sehr wenigen Befragten als störendste Lärmquelle in Innsbruck angenommen (S. 313).

– Von den einzelnen Lärmquellen geringbelastete Befragte glauben durchgehend am häufigsten, dass der Straßenverkehr die störendste Lärmquelle in Innsbruck ist. Stärker lärmbelastete Personen nähern sich den Mittelwerten an – mit einer Ausnahme: **Stark fluglärmbelastete Innsbruckerinnen und Innsbrucker antworten entgegen dem Trend und sehen mit großem Abstand den Fluglärm als störendste Lärmquelle für Innsbruck insgesamt an** (S. 316).

– Aus den Auswertungen ist ersichtlich, dass von einer bestimmten Lärmquelle stark belästigte Befragte dazu neigen, dieses Gefühl der persönlichen Störung auf die Gesamtheit Innsbrucks zu übertragen. Eine Abstraktion von der persönlichen Belästigung auf die insgesamt störendste

Lärmquelle gelingt nicht (gänzlich), oder wird bewusst nicht angestrebt. Die **Extremgruppen der Starkbelästigten schätzen die sie selbst stark belästigende Quelle überproportional häufig als störendste Lärmquelle für Gesamtinnsbruck ein.** Das gilt nicht nur für die Verkehrslärmquellen, sondern besonders ausgeprägt auch für die nicht verkehrsinduzierten Quellen (S. 317).

5.20. Sinnhaftigkeit (weiterer) Maßnahmen zur Lärmreduktion

– Mehr als **zwei Drittel** aller Befragten **sind der Meinung, in Innsbruck sollten (weitere) Maßnahmen zur Lärmreduktion getroffen werden**. Über 14% machen keine Angaben und **knapp 19% halten (weitere) Maßnahmen zur Lärmreduktion nicht für zielführend** (S. 319). Werden nur diejenigen Befragten herangezogen, die diese Frage mit *Ja* oder *Nein* beantwortet haben, sprechen sich 78% für (weitere) Lärmreduktionsmaßnahmen aus, knapp 22% dagegen (S. 319).

– **Frauen** halten (weitere) Maßnahmen eher für sinnvoll als Männer. Die jüngste **Altersgruppe** der Befragten sieht eine geringere Notwendigkeit für Lärmreduktionsmaßnahmen als die beiden anderen Altersgruppen. Je höher der höchste erreichte **Bildungsabschluss**, desto eher werden Maßnahmen zur Lärmreduktion von den befragten Innsbruckerinnen und Innsbruckern befürwortet. Die Unterschiede im Antwortverhalten sind wiederum vor allem zwischen Personen mit Pflichtschulabschluss und Akademikerinnen und Akademikern sehr ausgeprägt. Probandinnen und Probanden, die Innsbruck im Vergleich mit anderen österreichischen Städten für lauter halten, sprechen sich häufiger für (weitere) Maßnahmen zur Lärmbekämpfung aus als Befragte, die Innsbruck insgesamt für ruhiger als andere vergleichbare Städte ansehen (S. 321). Die **gegebene Lärmbelastung** im Wohnumfeld der Befragten ist **nicht bzw. nur in sehr geringfügigem Ausmaß ausschlaggebend** für den Grad der Zustimmung zu (weiteren) Lärmreduktionsmaßnahmen (S. 321).

– Die **Befürwortung (weiterer) Maßnahmen** zur Lärmreduktion in Innsbruck **unterscheidet sich** deutlich je **nach Belästigungsgrad** der Befragten. Die vom Lärm insgesamt nur gering belästigten Befragten, erachten zu 65% weitere Maßnahmen für notwendig, dieser **Grad der Zustimmung steigt bei den Starkbelästigten auf 90%.** Dabei zeigt sich auch erneut eine **erhöhte Sensibilität auf Lärmbelästigung im Schlaf** (S. 322).

– Die 691 befragten Innsbruckerinnen und Innsbrucker (67% des befragten Kollektivs), welche (weitere) Maßnahmen zur Lärmreduktion in der Stadt für sinnvoll halten, wurden weitergehend nach ihrer Meinung zu einzelnen Maßnahmenbereichen befragt. Die ***Schaffung, der Schutz und der Erhalt lärmarmer Bereiche in der Stadt Innsbruck*** wird von den Befragten **am häufigsten als sinnvolle Maßnahme befürwortet. Generell werden vor allem Förderungsmaßnahmen für sinnvoll erachtet (*Förderung lärmarmer Verkehrsmittel, Maschinen usw., Förderung von Lärmschutzmaßnahmen*), die von deutlich über 80% derjenigen Befragten, die Maßnahmen zur Lärmreduktion für zweckmäßig halten, angeregt werden** (S. 324). Die ***Bewusstseinsbildung zur Lärmvermeidung*** wird ebenfalls von über 80% aus dieser Subgruppe für wichtig erachtet, erhält aber geringere Zustimmungswerte als die Förderungsmaßnahmen auf den ersten drei Plätzen. ***Strengere Kontrollen der Einhaltung von Lärmgrenzwerten***, sowie ***strengere Vorschriften und Auflagen für Lärmverursacherinnen und –Verursacher*** werden **von rund drei Vierteln gutgeheißen**. ***Finanzielle Auflagen für Lärmverursacherinnen und –Verursacher***, sowie ***Verkehrsbeschränkungen*** hält knapp **mehr als die Hälfte** der Maßnahmenbefürworterinnen und –Befürworter für sinnvoll. **Bezogen auf das Gesamtkollektiv aller 1031 befragten Innsbruckerinnen und Innsbrucker befürwortet über ein Drittel auch Verkehrsbeschränkungen und finanzielle Auflagen** (S. 325).

Ausblick

Der vorliegende Bericht umfasst eine Basisdarstellung der Lärmsituation in Innsbruck und der Ergebnisse aus den Befragungen, die erste Einblicke vermitteln. Das umfangreiche Datenmaterial mit seiner breit gefächerten Schwerpunktsetzung lässt jedoch eine **Vielzahl weiterer**, wesentlich spezifischerer **Nutzungen, Aussagen und Rückschlüsse** zu, die jederzeit erarbeitet werden können:

- **Lebenswerte Stadt:** Schaffung eines Nachhaltigkeitsinstrumentariums für die zukünftige Stadtentwicklung – Innsbruck als Modellfall für die integrative Beurteilung von Gesundheit, Lebensqualität und Lebenszufriedenheit in Bezug auf Lärm und darüber hinausgehend:
    - Identifizierung lärmarmer, schützenswerter (auch kleinräumiger) Bereiche der Stadt
    - Identifizierung von (auch kleinräumigen) Problemzonen mit aktuellem und zukünftigem Konfliktpotential in Bezug auf Lärm.
    - Gezielte Ausrichtung von Aktionsplänen und Leitbildformulierungen unter Berücksichtigung aller Parameter u.v.a.m.
- **Implementierung** der aktualisierten Lärmdaten inkl. Gesamtlärmbelastung in die **Lärmkataster** und **Umgebungslärmkarten**.
- Beurteilung und Umsetzung konkreter öffentlicher Anliegen – auch und besonders mit Bürgerbeteiligung – auf **Basis solider, interessensunabhängiger Daten** und Grundlagen aus einer Hand.
- **GIS-Implementierungen der Belästigungsreaktion** und anderer Umfragedaten als Beurteilungskriterien für die Maßnahmenbeurteilung und –Planung, sowie als Informationsquelle für Raumordnung, Siedlungsplanung, Verkehrskonzepte, Betriebsansiedlungen, Lärmminderungsplanung, Aktionspläne, Mobilitätsstrategien, Städteplanung, Erholungskonzepte, Umweltverträglichkeitsverfahren u.v.a.m.
- **Modularer Aufbau:** Auf das Pilotprojekt aufbauend können Ausbaustufen in jede Richtung einfach aufgesetzt und zusammengeführt werden: Luftgütekataster, Lebensqualitäts- und Lebenszufriedenheitsindizes allgemein und auf spezielle Aspekte bezogen, Ökologie und Umweltschutz, spezifische Lärmforschungsansätze u.v.a.m.
- **Wissenschaftliche Nutzung:**
    - Erstellung von Dosis-Wirkungs-Kurven
    - Sonderauswertungen für spezifische Belastungen / Items
    - Longitudinale oder qualitative Folgestudien
    - (Internationale) Vergleichsstudien und wissenschaftliche Vernetzung der Daten
    - Publikationen und tiefergehende Ausarbeitung einzelner Ergebnisse
- **Innovationsmotor Land Tirol und Stadt Innsbruck:** Der gewählte Ansatz und der Umfang des *Pilotprojekts Gesamtlärmbetrachtung Innsbruck* stellen bis dato ein Alleinstellungsmerkmal dar und werden dazu beitragen, bestehende Dichotomien aufzulösen: Das reine Ausmaß des Schalldrucks, sprich: die gegebene Belastung durch Lärm entspricht nicht allein der Wahrnehmung und Bewertung durch die Betroffenen. Belästigung ist von der Quellenart, der Geräuschcharakteristik und der subjektiven Beurteilung abhängig.

Inhaltsverzeichnis

Vorwort der Autoren **1**

Zentrale Inhalte und Ergebnisse **2**
1. Einleitungsteil 2
2. Lärmdaten 2
3. Lärm in Innsbruck 2
3.1. Belastungszonen 2
3.2. Verkehrslärmbelastung 3
3.3. Gesamtlärmbetrachtung Innsbruck 4
4. Durchführungsparameter der Befragungen 5
5. Auswertung der Befragungen 6
5.1. Mobilität 6
5.2 Lebens- und Wohnbedingungen in Innsbruck 6
5.3. Zufriedenheit mit der eigenen Wohnsituation 7
5.4. Gesundheitszustand 8
5.5. Lebensqualität 8
5.6. Lärmempfindlichkeit 9
5.7. Nachtschlaf bei geöffnetem / gekipptem Fenster 10
5.8. Baulicher Lärmschutz 11
5.9. Lärmexpositionsempfinden 11
5.10. Belästigung / Störung durch Lärm 13
5.11. Belästigung / Störung durch Lärm im Schlaf 16
5.12. Lärmcoping 19
5.13. Empfundene Zu- oder Abnahme des Lärmbelastungen in Innsbruck 20
5.14. Einschätzung der Negativwirkung von Lärm auf die Gesundheit 21
5.15. Einfluss von Wind und Wetter auf die Lärmbelastung 21
5.16. Bekanntheit der Umgebungslärmkarten 21
5.17. Einschätzung des Lärmniveaus von Innsbruck im Städtevergleich 22
5.18. Einschätzung des eigenen Lärmbeitrags zur Gesamtlärmbelastung 22
5.19. Einschätzung der störendsten Lärmquelle in Innsbruck 22
5.20. Sinnhaftigkeit (weiterer) Maßnahmen zur Lärmreduktion 23

Ausblick **24**

Inhaltsverzeichnis **25**

EINLEITUNG **33**

Lärm als Herausforderung **33**

Projektpartner **34**
1. Leitung 34
2. Partner (alphabetisch) 34

Die Stadt Innsbruck **35**
1. Stadtgebiet und Katastralgemeinden 35
2. Demographie 36

LÄRMDATEN **38**

Grundbegriffe Lärm **38**
1. Schalltechnische Grundbegriffe 38
2. Ausbreitungsberechnungen 38
3. GIS-Darstellung 39

Verwendete Lärmdaten **40**
1. Die Ausgangslage 40
2. Berechnung als geeignetes Verfahren 40
3. Umgebungslärmkarten 41
3.1. Umfang 41
3.2. Angaben zur eingesetzten Software 41
3.3. Angaben zur Modellbildung 42
3.3.1. Beschreibung der Eingangsdaten Gelände 42
3.3.2. Beschreibung der Eingangsdaten Gebäude 42
3.3.3. Beschreibung der Eingangsdaten Lärmschutz 42
3.3.4. Beschreibung der Eingangsdaten Bodendämpfung 42
3.3.5. Anzahl der berücksichtigten Reflexionen und Rasterweite 43
3.3.6. Angaben zur grenzüberschreitenden Betrachtung von Lärmquellen 43
4. Verkehrslärm 43
4.1. Straßenverkehrslärm 43
4.1.1. Angaben zur Erhebung der Emissionsquellen 43
4.1.2. Angaben zu den verwendeten Geschwindigkeiten 43
4.1.3. Beschreibung der Eingangsdaten Straßenemissionen 43
4.2. Schienenverkehrslärm 44
4.2.1. Angaben zur Erhebung der Emissionsquellen 44
4.2.2. Angaben zu den verwendeten Geschwindigkeiten 44
4.2.3. Beschreibung der Eingangsdaten Schienenemissionen 44
4.3. Fluglärm 44
4.3.1. Angaben zur Erhebung der Emissionsquellen 44
4.3.2. Beschreibung der Eingangsdaten Flugemissionen 44
4.4. Betroffenenauswertung - Bestimmung der betroffenen Einwohner 45
5. Nicht verkehrsinduzierter Lärm 45
6. Messung in Kranebitten 45
6.1. Lärmmessung 45
6.2. Vergleich Messung – Berechnung Fluglärm 46

Gesamtlärmberechnung **47**

LÄRM IN INNSBRUCK **48**

Die Ist-Situation **48**
1. Belastungszonen 48

Verkehrslärmbelastung **53**
1. 24h-Verkehrslärmbelastung 53
1.1. Vergleich Innsbruck gesamt – befragtes Kollektiv 53
1.2. 24h-Verkehrslärmbelastung nach Katastralgemeinden 55
2. Verkehrslärmbelastung in der Nacht 59
2.1. Vergleich Innsbruck gesamt – befragtes Kollektiv 59
2.2. Verkehrslärmbelastung in der Nacht nach Katastralgemeinden 61

Gesamtlärmbetrachtung Innsbruck **66**
1. Prinzip und Limitationen 66
2. Zusammenführen von Schallbelastungen 66
2. Wirkungsbezogene Darstellung 69
3. Hotspots 76
4. Ruhige Gebiete 77

DURCHFÜHRUNGSPARAMETER DER BEFRAGUNGEN **80**

Setting und Untersuchungsumfang der Befragungen **80**

Der Fragebogen **81**
1. Zusammenstellung 81
2. Aufbau und Inhaltliche Schwerpunkte 81

Die Stichprobe **83**
1. Demographische Repräsentativität 83
1.1. Alter 83
1.2. Geschlechterverteilung 83
1.3. Stichprobengröße 84
2. Repräsentative Abbildung der 9 Katastralgemeinden 84
3. Lärmkorridore 85

Rücklaufquote **87**

Durchführung der Befragungen und Datenkontrolle **89**

Datenschutz **90**

Statistische Methoden **91**
1. Deskriptive Analyse 91
2. Extremgruppen 91
3. Weiterführende Auswertungen und Inferenzstatistik 92

AUSWERTUNG DER BEFRAGUNGEN **93**

Demographie **93**
1. Alter und Geschlecht 93
2. Bildung 94
3. Berufliche Stellung 95

Wohnen **98**
1. Wohndauer im selben Haus / derselben Wohnung 98
2. Art des Wohnhauses und Eigentumsverhältnisse 99
3. Haushaltsgröße 99
4. Stockwerk des Schlafzimmers 100
5. Vorhandene Abschirmungen 101
5.1. Bauliche Abschirmungen 101
5.2. Innenhof / Lärmabgewandte Seite 101
6. Lärmschutzfenster 102

Mobilität **103**
1. Pendlerinnen und Pendler 103
2. Mobilität im Alltag 104
2.1. Kraftfahrzeugnutzung als Fahrerin / Fahrer 104
2.2. Nutzung öffentlicher Verkehrsmittel in der Stadt 106
2.3. Nutzung öffentlicher Verkehrsmittel außerhalb der Stadt 107
2.4. Nutzung von Fahrrädern 108
2.5. Fußläufige Wege 109
2.6. Nutzung von Flugzeugen 111

Lebensbedingungen in Innsbruck **113**
1. Überblick Lebensbedingungen in Innsbruck 113
2. Wohnbedingungen in Innsbruck 113
3. Nachbarschaftsbeziehungen in Innsbruck 114
4. Sicherheit in Innsbruck 115
5. Landschaft, Natur, Parks und Grünflächen in Innsbruck 116
6. Luftqualität in Innsbruck 116
7. Öffentliche Verkehrsmittel in Innsbruck 117
8. Bedingungen für das Aufwachsen von Kinder in Innsbruck 118

9. Verbundenheit zur Stadt Innsbruck 119
10. Lebensbedingungen und ausgewählte Parameter 119
10.1. Lebensbedingungen in Innsbruck und Katastralgemeinde 119
10.2. Lebensbedingungen in Innsbruck und Geschlecht 121
10.3. Lebensbedingungen in Innsbruck und Alter 121
10.4. Lebensbedingungen in Innsbruck und Bildung 122
10.5. Lebensbedingungen in Innsbruck und Gesundheitszustand 123
10.6. Lebensbedingungen in Innsbruck und Lärmempfindlichkeit 123
10.7. Lebensbedingungen in Innsbruck und Lebensqualität 124
10.8. Lebensbedingungen in Innsbruck und Verkehrslärmbelastung 124
10.8.1. Straßenverkehrslärmbelastung 124
10.8.2. Schienenverkehrslärmbelastung 125
10.8.3. Flugverkehrslärmbelastung 126
10.9. Lebensbedingungen in Innsbruck und Lärmexpositionsempfinden 126
10.9.1. Lärmexpositionsempfinden insgesamt 126
10.9.2. Straßenlärmexpositionsempfinden 127
10.9.3. Fluglärmexpositionsempfinden 127
10.9.4. Schienenverkehrslärmexpositionsempfinden 128
10.10. Lebensbedingungen in Innsbruck und Lärmbelästigung 128
10.10.1. Lärmbelästigung insgesamt 128
10.10.2. Straßenverkehrslärmbelästigung 129
10.10.3. Flugverkehrslärmbelästigung 130
10.10.4. Schienenverkehrslärmbelästigung 130
10.10.5. Nicht verkehrsverursachte Lärmbelästigung 131
10.11. Lebensbedingungen in Innsbruck und weitere Variablen 133

Zufriedenheit mit der eigenen Wohnsituation 134
1. Wohnsituation und Katastralgemeinden 134
2. Wohnsituation und ausgewählte Variablen 135
3. Wohnsituation und Lärmbelastung 137
3.1. Straßenverkehrslärmbelastung 137
3.2. Flugverkehrslärmbelastung 138
3.3. Schienenverkehrslärmbelastung 139
4. Wohnsituation und nächtliche Lärmbelastung 140
4.1. Straßenverkehrslärmbelastung 140
4.2. Flugverkehrslärmbelastung 140
4.3. Schienenverkehrslärmbelastung 141
5. Wohnsituation und Lärmexpositionsempfinden 141
5.1. Verkehrsverursachtes Lärmexpositionsempfinden 141
5.2. Nichtverkehrsverursachtes Lärmexpositionsempfinden 142
6. Wohnsituation und Lärmbelästigung 142
7. Wohnsituation und Lärmbelästigung im Schlaf 144

Gesundheitszustand 145
1. Gesundheitszustand im Überblick 145
2. Gesundheitszustand und ausgewählte Parameter 145
3. Gesundheitszustand und Lärmbelastung 147
4. Gesundheitszustand und Lärmexpositionsempfinden 147
5. Gesundheitszustand und Lärmbelästigung 148
6. Gesundheitszustand und Belästigung durch Lärm im Schlaf 149

Lebensqualität 150
1. Lebensqualität im Überblick 150
2. Lebensqualität und ausgewählte Parameter 150
3. Lebensqualität und Lärmbelastung 152
4. Lebensqualität und Lärmexpositionsempfinden 153
5. Lebensqualität und Lärmbelästigung 154
6. Lebensqualität und Belästigung durch Lärm im Schlaf 155

Lärmempfindlichkeit 156
1. Lärmempfindlichkeit im Überblick 156

2. Lärmempfindlichkeit und ausgewählte Parameter ... 156
3. Lärmempfindlichkeit und Lärmbelastung ... 158
4. Lärmempfindlichkeit und Lärmexpositionsempfinden ... 159
5. Lärmempfindlichkeit und Lärmbelästigung ... 160
6. Lärmempfindlichkeit und Belästigung durch Lärm im Schlaf ... 162

Wind- und Wettereinflüsse auf die Lärmbelastung ... 163
1. Wind- und Wettereinflüsse auf den Lärm im Überblick ... 163
2. Wind- und Wettereinflüsse und ausgewählte Parameter ... 163

Nachtschlaf bei geöffnetem Fenster ... 166
1. Nachtschlaf bei geöffnetem Fenster im Überblick ... 166
2. Nachtschlaf und ausgewählte Parameter ... 166

Lärmexpositionsempfinden ... 170
1. Überblick empfundene Lärmexposition ... 170
1.1. Lärmexposition nach Lärmquelle ... 170
1.2. Verkehrslärmexposition und – Belastung ... 172
2. Lärmexposition insgesamt ... 173
2.1. Lärmexposition insgesamt und Katastralgemeinden ... 174
2.2. Lärmexposition insgesamt und ausgewählte Parameter ... 174
3. Flugverkehrslärmexposition ... 177
3.1. Fluglärmexposition und Fluglärmbelastung ... 177
3.2. Fluglärmexposition und Katastralgemeinden ... 178
3.3. Fluglärmexposition und ausgewählte Parameter ... 179
4. Straßenverkehrslärmexposition ... 180
4.1. Straßenlärmexposition und Straßenlärmbelastung ... 181
4.2. Straßenlärmexposition und Katastralgemeinden ... 182
4.3. Straßenlärmexposition und ausgewählte Parameter ... 182
5. Baustellenlärmexposition ... 184
5.1. Baustellenlärmexposition und Katastralgemeinden ... 185
5.2. Baustellenlärmexposition und ausgewählte Parameter ... 185
6. Nachbarschaftslärmexposition ... 187
6.1. Nachbarschaftslärmexposition und Katastralgemeinden ... 188
6.2. Nachbarschaftslärmexposition und ausgewählte Parameter ... 188
7. Lärmexposition durch Passanten und Gaststätten ... 190
7.1. Lärmexposition Passanten / Gaststätten und Katastralgemeinden ... 191
7.2. Lärmexposition Passanten / Gaststätten und einzelne Parameter ... 191
8. Exposition gegenüber Erschütterungen ... 193
8.1. Erschütterungsexposition und Katastralgemeinden ... 193
8.2. Erschütterungsexposition und ausgewählte Parameter ... 194
9. Schienenverkehrslärmexposition ... 195
9.1. Schienenlärmexposition und Schienenlärmbelastung ... 196
9.2. Schienenlärmexposition und Katastralgemeinden ... 197
9.3. Schienenlärmexposition und ausgewählte Parameter ... 198
10. Autobahnlärmexposition ... 199
10.1. Autobahnlärmexposition und Katastralgemeinden ... 200
10.2. Autobahnlärmexposition und ausgewählte Parameter ... 200
11. Lärmexposition durch Gewerbe und Industrie ... 201
11.1. Lärmexposition Gewerbe und Industrie und Katastralgemeinden ... 202
11.2. Lärmexposition Gewerbe und Industrie und einzelne Parameter ... 202

Belästigung / Störung durch Lärm ... 204
1. Überblick Belästigung / Störung durch Lärm ... 204
1.1. Belästigung nach Lärmquelle ... 204
1.2. Belästigung und Verkehrslärmbelastung ... 206
1.3. Belästigung und Katastralgemeinden ... 207
1.3.1. Belästigung nach Quelle und Katastralgemeinden ... 207
1.3.2. Verkehrslärmbelästigung und Katastralgemeinden ... 209
1.3.3. Nicht verkehrsverursachte Belästigung und Katastralgemeinden ... 209
2. Belästigung durch Lärm insgesamt ... 210

2.1. Belästigung durch Lärm insgesamt und Katastralgemeinden ... 210
2.2. Belästigung durch Lärm insgesamt und ausgewählte Parameter ... 211
3. Belästigung durch Flugverkehrslärm ... 215
3.1. Fluglärmbelästigung, -Belastung und -Expositionsempfinden ... 215
3.2. Fluglärmbelästigung und Katastralgemeinden ... 216
3.3. Fluglärmbelästigung und ausgewählte Parameter ... 216
4. Belästigung durch Straßenverkehrslärm ... 219
4.1. Straßenlärmbelästigung, -Belastung und -Expositionsempfinden ... 220
4.2. Straßenlärmbelästigung und Katastralgemeinden ... 220
4.3. Straßenverkehrslärmbelästigung und ausgewählte Parameter ... 221
5. Belästigung durch Baustellenlärm ... 223
5.1. Baulärmbelästigung und Katastralgemeinden ... 224
5.2. Baulärmbelästigung und ausgewählte Parameter ... 225
6. Belästigung durch Nachbarschaftslärm ... 226
6.1. Nachbarschaftslärmbelästigung und Katastralgemeinden ... 227
6.2. Nachbarschaftslärmbelästigung und ausgewählte Parameter ... 227
7. Belästigung durch Lärm von Passanten und Gaststätten ... 229
7.1. Lärmbelästigung Passanten / Gaststätten und Katastralgemeinden ... 230
7.2. Lärmbelästigung Passanten / Gaststätten und einzelne Parameter ... 230
8. Belästigung durch Schienenverkehrslärm ... 232
8.1. Schienenlärmbelästigung, -Belastung und -Expositionsempfinden ... 232
8.2. Schienenlärmbelästigung und Katastralgemeinden ... 233
8.3. Schienenlärmbelästigung und ausgewählte Parameter ... 234
9. Belästigung durch Erschütterungen ... 236
9.1. Belästigung durch Erschütterungen und Katastralgemeinden ... 236
9.2. Belästigung durch Erschütterungen und ausgewählte Parameter ... 237
10. Belästigung durch Lärm aus Gewerbe und Industrie ... 238
10.1. Lärmbelästigung Gewerbe und Industrie und Katastralgemeinden ... 239
10.2. Lärmbelästigung Gewerbe und Industrie und einzelne Parameter ... 239
11. Belästigung durch Autobahnlärm ... 241
11.1. Autobahnlärmbelästigung, -Belastung und -Expositionsempfinden ... 242
11.2. Belästigung durch Autobahnlärm und Katastralgemeinden ... 242
11.3. Belästigung durch Autobahnlärm und ausgewählte Parameter ... 243

Belästigung / Störung durch Lärm im Schlaf ... 245
1. Überblick Belästigung / Störung durch Lärm im Schlaf ... 245
1.1. Belästigung im Schlaf nach Lärmquelle ... 245
1.2. Belästigung im Schlaf und Verkehrslärmbelastung ... 247
1.3. Belästigung im Schlaf und Katastralgemeinden ... 248
1.3.1. Belästigung im Schlaf nach Quelle und Katastralgemeinden ... 248
1.3.2. Verkehrslärmbelästigung im Schlaf und Katastralgemeinden ... 249
1.3.3. Nicht verkehrsverursachte Lärmbelästigung im Schlaf und Katastralgemeinden ... 250
2. Belästigung im Schlaf durch Lärm insgesamt ... 250
2.1. Belästigung im Schlaf durch Lärm insgesamt u. Katastralgemeinden ... 251
2.2. Belästigung im Schlaf durch Lärm insgesamt und ausgewählte Parameter ... 252
3. Belästigung im Schlaf durch Straßenverkehrslärm ... 256
3.1. Straßenlärmbelästigung im Schlaf und Straßenlärmbelastung ... 256
3.2. Straßenlärmbelästigung im Schlaf und Katastralgemeinden ... 257
3.3. Straßenverkehrslärmbelästigung im Schlaf und andere Parameter ... 257
4. Belästigung im Schlaf durch Lärm von Passanten und Gaststätten ... 260
4.1. Lärmbelästigung im Schlaf durch Passanten und Gaststätten und Katastralgemeinden ... 261
4.2. Lärmbelästigung im Schlaf durch Passanten und Gaststätten und ausgewählte Parameter ... 262
5. Belästigung im Schlaf durch Nachbarschaftslärm ... 264
5.1. Nachbarschaftslärmbelästigung im Schlaf und Katastralgemeinden ... 264
5.2. Nachbarschaftslärmbelästigung im Schlaf und einzelne Parameter ... 265
6. Belästigung im Schlaf durch Flugverkehrslärm ... 267
6.1. Fluglärmbelästigung im Schlaf und Fluglärmbelastung ... 267
6.2. Fluglärmbelästigung im Schlaf und Katastralgemeinden ... 268

6.3. Fluglärmbelästigung im Schlaf und ausgewählte Parameter 268
7. Belästigung im Schlaf durch Baustellenlärm 270
7.1. Baulärmbelästigung im Schlaf und Katastralgemeinden................................. 271
7.2. Baulärmbelästigung im Schlaf und ausgewählte Parameter............................ 272
8. Belästigung im Schlaf durch Schienenverkehrslärm 273
8.1. Schienenlärmbelästigung im Schlaf und Schienenlärmbelastung.................... 274
8.2. Schienenlärmbelästigung im Schlaf und Katastralgemeinden 274
8.3. Schienenlärmbelästigung im Schlaf und ausgewählte Parameter 275
9. Belästigung im Schlaf durch Erschütterungen 277
9.1. Belästigung im Schlaf durch Erschütterungen und Katastralgemeinden 277
9.2. Belästigung im Schlaf durch Erschütterungen und ausgewählte Parameter 278
10. Belästigung im Schlaf durch Lärm aus Gewerbe und Industrie 279
10.1. Belästigung im Schlaf durch Lärm von Gewerbe und Industrie und Katastralgemeinden 280
10.2. Belästigung im Schlaf durch Lärm von Gewerbe und Industrie und ausgewählte Parameter 281
11. Belästigung durch Autobahnlärm im Schlaf 282
11.1. Belästigung im Schlaf durch Autobahnlärm und Autobahnlärmbelastung 283
11.2. Belästigung im Schlaf durch Autobahnlärm und Katastralgemeinden.............. 284
11.3. Belästigung im Schlaf durch Autobahnlärm und einzelne Parameter............... 284

Lärmcoping 287
1. Überblick und Summenscore 287
2. Instrumentelles Lärmcoping 288
2.1. Instrumentelles Lärmcoping und ausgewählte Parameter 288
3. Emotionales Lärmcoping 289
3.1. Emotionales Lärmcoping und Lärmbelastung 289
3.2. Emotionales Lärmcoping und ausgewählte Parameter 291
4. Politisches Lärmcoping 292
4.1. Politisches Lärmcoping und ausgewählte Parameter 292
5. Summenscore Lärmcoping und ausgewählte Parameter 293
6. Summenscore Lärmcoping und Lärmbelästigung 295

Empfundene Zu- oder Abnahme des Lärms in Innsbruck 298
1. Lärmbelastungsveränderung im Überblick 298
2. Lärmbelastungsveränderung und ausgewählte Parameter 298
3. Lärmbelastungsveränderung und Lärmbelästigung 300

Einschätzung der Auswirkungen von Lärm auf die Gesundheit 302
1. Auswirkungen von Lärm im Überblick 302
2. Auswirkungen von Lärm und ausgewählte Parameter 303

Bekanntheit der Umgebungslärmkarten 305

Einschätzung des Lärmniveaus im Städtevergleich 306
1. Lärmniveau im Städtevergleich im Überblick 306
2. Lärmniveau im Städtevergleich und einzelne Parameter 307

Einschätzung des eigenen Lärmbeitrags 310
1. Eigener Lärmbeitrag im Überblick 310
2. Eigener Lärmbeitrag und demographische Parameter 311
3. Eigener Lärmbeitrag und Mobilitätsverhalten 311

Einschätzung der störendsten Lärmquelle in Innsbruck 313
1. Störendste Lärmquelle im Überblick 313
2. Störendste Lärmquelle und demographische Parameter 313
3. Störendste Lärmquelle und ausgewählte Parameter 314
4. Störendste Lärmquelle und Lärmbelastung 315
5. Störendste Lärmquelle und Lärmbelästigung 317

Sinnhaftigkeit von Maßnahmen zur Lärmreduktion in Innsbruck 319

1. Überblick Sinnhaftigkeit von Lärmreduktionsmaßnahmen....................................... 319
2. Sinnhaftigkeit von Lärmreduktionsmaßnahmen und ausgewählte Parameter.......... 319
3. Sinnhaftigkeit von Lärmreduktionsmaßnahmen und Lärmbelastung 321
4. Sinnhaftigkeit von Lärmreduktionsmaßnahmen und Lärmbelästigung..................... 322
5. Einzelmaßnahmen zur Lärmreduktion in Innsbruck 324

VERZEICHNISSE.. 326

Abkürzungsverzeichnis.. 326

Tabellenverzeichnis... 329

Abbildungsverzeichnis... 334

ANHANG.. 342

Anhang 1: Deskriptive Statistik der Befragungen 342

Anhang 2: Häufigkeitsstatistik der Befragungen 345

Impressum 368

EINLEITUNG

Lärm als Herausforderung

Die Belastung/Belästigung durch Lärm ist die am häufigsten wahrgenommene Form der Umweltbelastung und gleichzeitig eines der meistverdrängten Umweltprobleme überhaupt.

Speziell im alpinen urbanen Raum stellt Lärm die Entscheidungsträger vor immer größere Herausforderungen. Dabei fehlt es nicht primär an wissenschaftlichen Grundlagen, sondern schlicht an umfassenden, nachvollziehbaren, stabilen Daten.

Das Pilotprojekt Gesamtlärmbetrachtung Innsbruck hat sich zum Ziel gesetzt, erstmals eine repräsentative, nachvollziehbare Datengrundlage für die Beurteilung der Gesamtbelastung und –Belästigung der Innsbrucker Stadtbevölkerung zu schaffen.

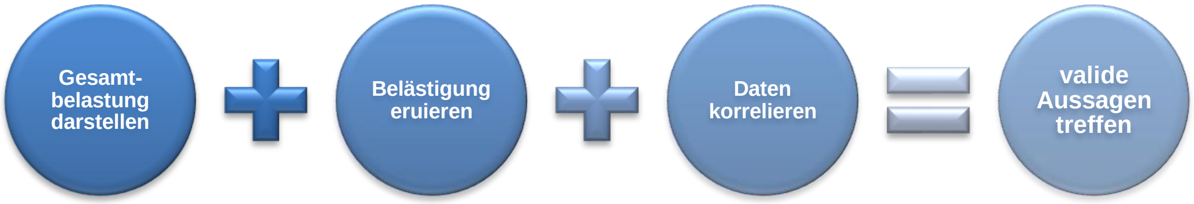

Projektpartner

1. Leitung

**Projektleitung / Gesamtlärmdaten:**	DIPL.-HTL-ING. MAG. CHRISTOPH LECHNER
**Befragungen / Auswertungen:**	MAG. DR. DAVID SCHNAITER

2. Partner (alphabetisch)

**Amt der Tiroler Landesregierung:**	
– Emissionen Sicherheitstechnik Anlagen:	ING. ROLAND GOGL
– Landessanitätsdirektion:	DR. MED. KARL HEINZ FISCHER
– Raumordnung:	DI ROBERT ORTNER
– Verkehr und Straße:	DI DR. CHRISTIAN MOLZER
– Verkehrsplanung:	DI DR. ALFRED LINTNER
**Anrainerschutzgemeinschaft Innsbruck Airport:**	DI MANFRED RONER
**Asfinag Alpenstrassen:**	DI (FH) KLAUS GSPAN
**Innsbruck Airport:**	DI MARCO PERNETTA
**Österr. Arbeitsring für Lärmbekämpfung**	DI WOLFGANG GRUBER
**Österr. Bundesbahnen:**	ING. CHRISTOPH SCHMID
**Stadtmagistrat Innsbruck:**	DI DR. REINHARD FISCHER

Die genannten Infrastrukturträger (in alphabetischer Reihenfolge) *Autobahnen- und Schnellstraßen-Finanzierung-Aktiengesellschaft, Österreichische Bundesbahnen Holding AG, Tiroler Flughafenbetriebsgesellschaft m.b.H.* haben neben der Bereitstellung von Verkehrsdaten ebenso wie das *Land Tirol*, die *Stadt Innsbruck* und der *Österreichische Arbeitsring für Lärmbekämpfung* auch finanzielle Beiträge zur Realisierung des Pilotprojektes Gesamtlärmbetrachtung Innsbruck geleistet und damit dieses Projekt überhaupt erst ermöglicht.

Die Konzeptionierung und Erstellung des Studiendesigns, die Lärmdatenausbreitungsberechnungen, die Entwicklung des Fragebogens, die Befragungsdurchführung und die zusammenführende Berichtlegung etc. wurden jedoch ohne jegliche Einflussnahme der genannten Infrastrukturträger ausgearbeitet und durchgeführt. Die Projektpartner wurden von der Projektleitung über die geplanten Inhalte in wiederkehrenden gemeinsamen Besprechungen informiert, es kam allerdings zu keinem Zeitpunkt und in keinster Weise zu einer inhaltlichen Einflussnahme der Projektpartner, denen auch kein Mitspracherecht in der Planung, Ausführung und Berichtlegung eingeräumt wurde. Durch dieses Vorgehen konnte von Seiten der Projektleitung eine vollkommen unabhängige Projektabwicklung sichergestellt und jeglicher Interessenskonflikt ausgeschlossen werden.

Die Stadt Innsbruck

1. Stadtgebiet und Katastralgemeinden

Das Stadtgebiet von Innsbruck umfasst 9 Katastralgemeinden: Innsbruck, Wilten, Pradl, Hötting, Mühlau, Amras, Arzl, Vill und Igls, die sich wiederum in Stadtteile und Bezirke gliedern.

Abbildung 1: Katastralgemeinden Innsbruck

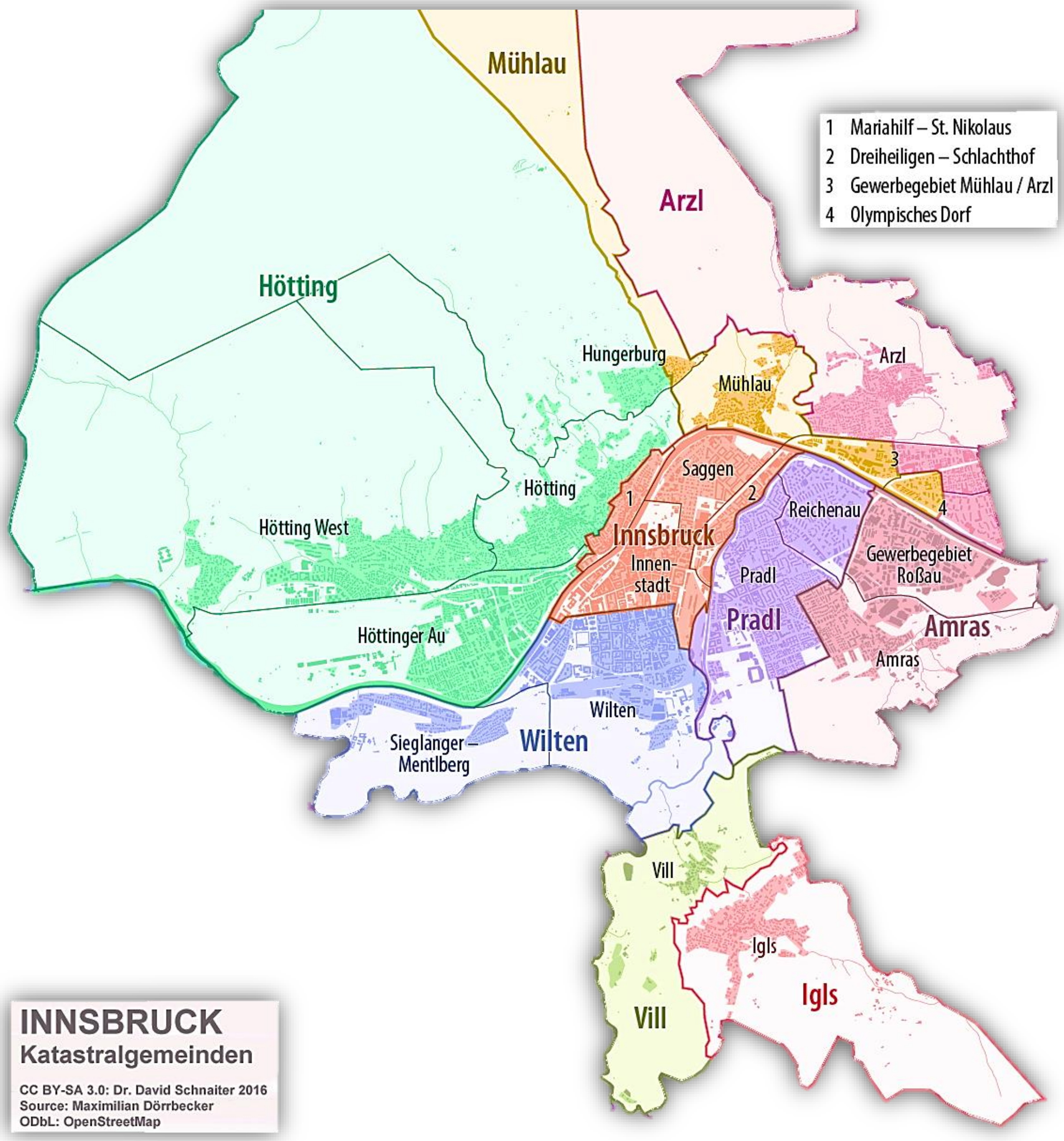

2. Demographie

Um eine repräsentative und homogene Verteilung der zu befragenden Personen zu erreichen, wurde in einem ersten Schritt eine soziodemographische Populationsanalyse auf Basis der Datensätze des *Zentralen Melderegisters*, des *Stadtmagistrats Innsbruck (Abtlg. Statistik und Berichtwesen),* der *Statistik Austria* und den statistischen Publikationen des *Amtes der Tiroler Landesregierung* vorgenommen.

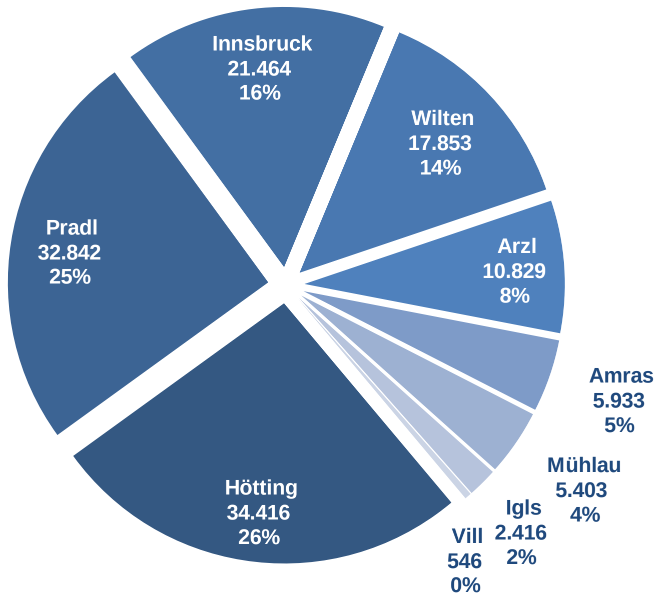

Abbildung 2: Einwohnerschaft Innsbrucks mit Hauptwohnsitz nach Katastralgemeinden (2016)

Mit Stichtag 01.01.2016 lebten im Stadtgebiet von Innsbruck 131.702 Einwohnerinnen und Einwohner mit Hauptwohnsitz (Nebenwohnsitzquote 14,7%), die sich auf die 9 Katastralgemeinden verteilen.

Auf einen Blick lassen sich die wichtigsten demographischen Parameter der Landeshauptstadt Innsbruck wie folgt darstellen:

Abbildung 3: Demographische Strukturen in Innsbruck auf einen Blick

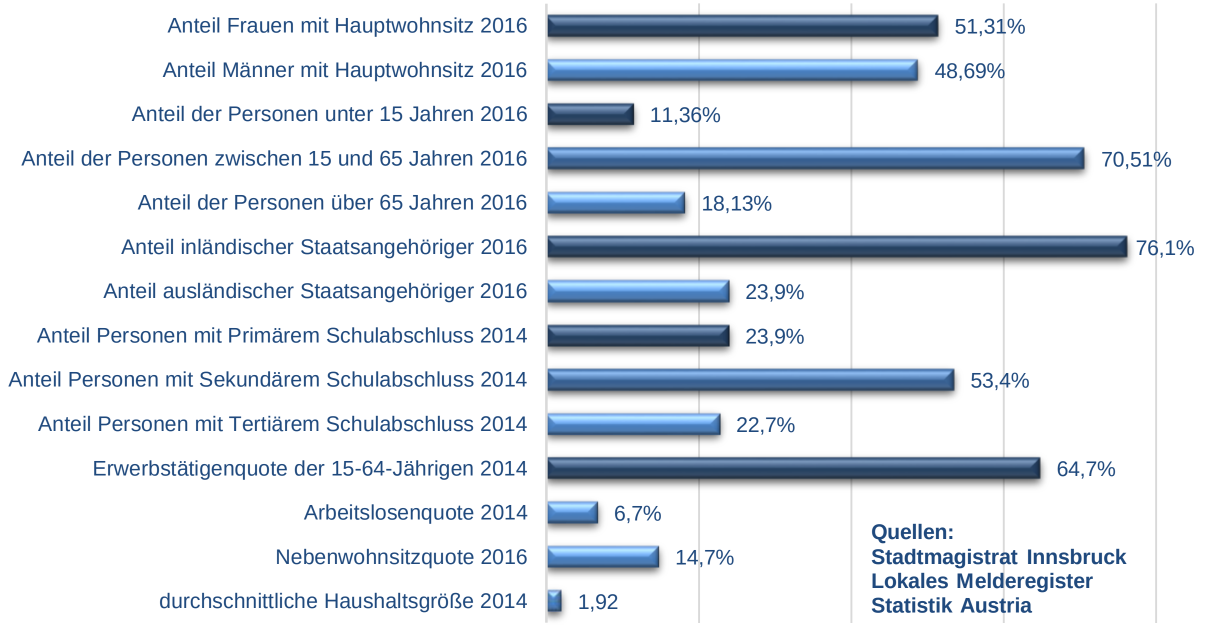

Für die demographische Repräsentativität von Befragungen sind vor allem die beiden Parameter Alter und Geschlecht von besonderem Interesse, aber auch die durchschnittliche Haushaltsgröße, die Er-

werbstätigenquote[1] und die Verteilung auf die Bildungsschichten, sind relevante Informationen für die Auswertung und Interpretation der Befragungsdaten.

Abbildung 4: Alterspyramide der Bevölkerung von Innsbruck nach Geschlecht 2016

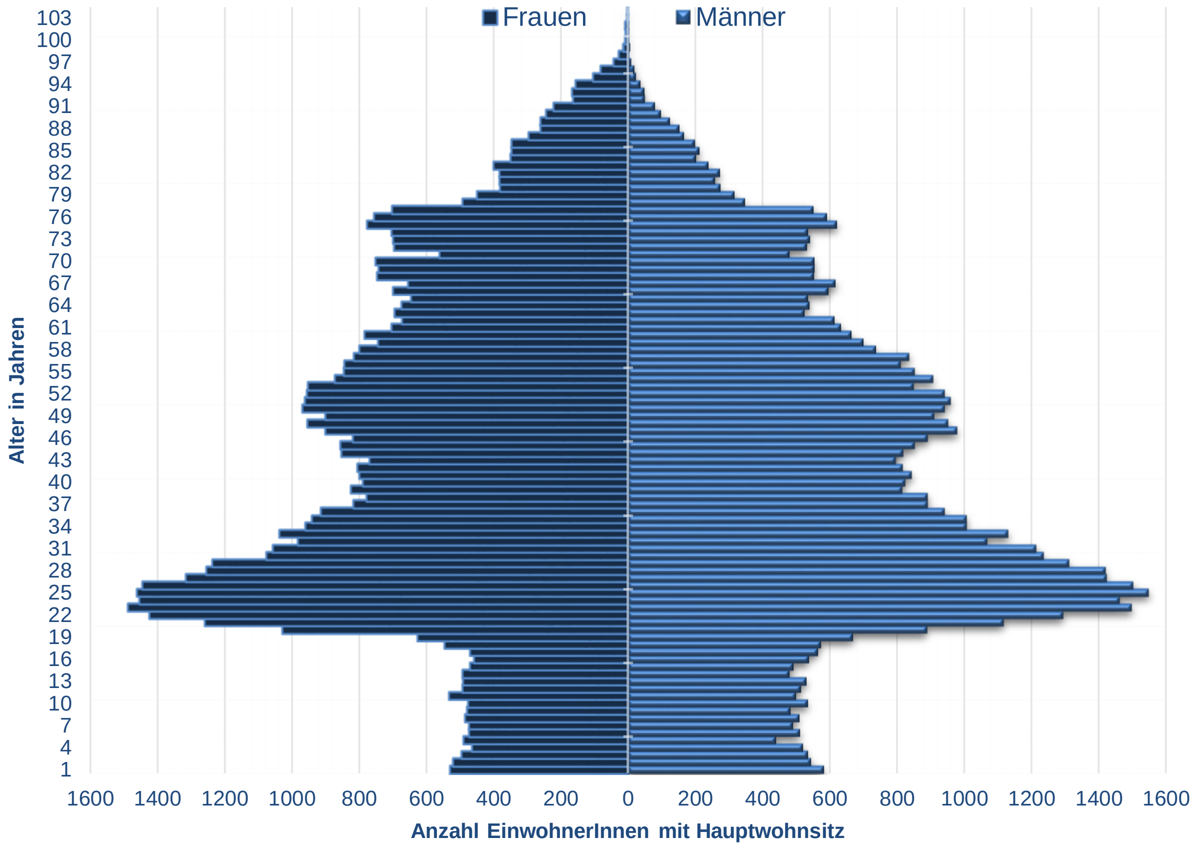

Abbildung 5: Bevölkerung von Innsbruck nach höchstem erreichten Schulabschluss (2014)

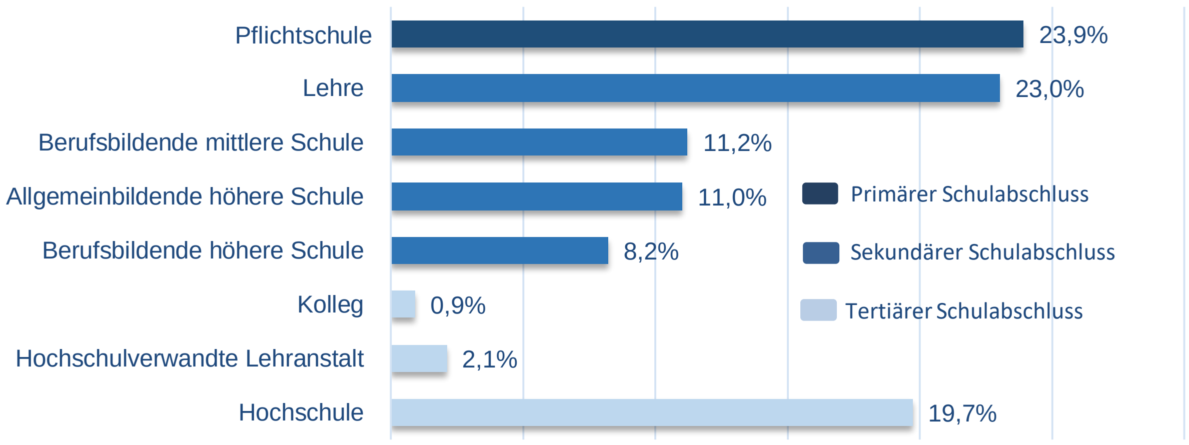

Im Kapitel *Die Stichprobe* werden die einzelnen Parameter detailliert dargestellt und das verwendete Adress-Cluster-Sampling näher erläutert.

[1] Erwerbstätigenquote: Der Erwerbsstatus basiert auf der ILO-Klassifizierung (International Labour Organisation) und untergliedert die Wohnbevölkerung in Erwerbspersonen (erwerbstätig, arbeitslos) und Nicht-Erwerbspersonen (Personen unter 15 Jahren; Personen mit Pensionsbezug; Schülerinnen und Schüler, sowie Studierende 15 Jahre und älter; sonstige Nicht-Erwerbspersonen).

LÄRMDATEN

Grundbegriffe Lärm

1. Schalltechnische Grundbegriffe

Eine Auflistung samt Kurzerläuterungen für die schalltechnischen Grundbegriffe findet sich im Abkürzungsverzeichnis.

2. Ausbreitungsberechnungen

Schallausbreitungsberechnungen sind eine Methode zur Ermittlung von vergangenen, bestehenden und zukünftigen Schalleinwirkungen auf rechnerischer Basis. Auf der Ebene großräumiger Belastungen werden dabei Ingenieurverfahren angewandt, welche auch im Verwaltungsbereich verbindlich sind.

Die Berechnung erfolgt dabei grundsätzlich für Dauerschallpegel und den daraus abgeleiteten Indizes wie L_{den} und L_{night}. Grundsätzlich besteht sie aus zwei Teilen, der Ermittlung der Emission und die Transmission. Bei der Emission werden Durchschnittswerte einzelner Quellen aus einer Vielzahl von Messungen eingesetzt und mit den für die Höhe maßgebenden Eigenschaften belegt. Diese sind bei Straßenfahrzeugen die Art des Fahrzeuges, die Geschwindigkeit, der Fahrbahnbelag und die Steigung der Straße. Durch die Mittelung des Berechnungsergebnisses heben sich auch die Schwankungen der Einzelereignisse auf. Je länger der Mittelungszeitraum und je höher die Anzahl der Vorbeifahrten, desto geringer sind die Abweichungen zwischen einer zeitgleichen Messung und Berechnung. Dass Einzelereignisse vom Mittelwert immer abweichen können, in positiver wie in negativer Richtung entspricht der allgemeinen Lebenserfahrung.

Bei der Transmission wird ein physikalisches Modell verwendet, dessen primäre Lösung darauf beruht, dass die Intensität des Schalls mit zunehmendem Abstand definiert sinkt. Weiters werden Reflexionen, Abminderungen durch Dämpfungen und Abschirmungen, Absorption in der Atmosphäre und meteorologische Effekte berücksichtigt. Letztere werden erst in größeren, 100 m übersteigenden Distanzen wirksam. Im städtischen Bereich werden diese durch die Nähe der Schallquellen kaum schlagend. Die Besonderheit der Ausbreitungsberechnung in Österreich ist die Annahme ausbreitungsgünstiger Meteorologie d. h. tendenziell höherer Immissionspegel. Im städtischen Bereich sind diese aber im Vergleich zum meteorologischen Durchschnitt im Zehntel-Dezibel-Bereich.

3. GIS-Darstellung

Die Darstellung von Schallausbreitungsberechnungen kann durch zwei Methoden erfolgen:

Rasterlärmkarte: flächenhafte Darstellung der Schallimmission, gegeben durch den energieäquivalenten Dauerschallpegel in dB, in einem Plan.

Gebäudelärmkarte: Abbildung der Pegel an den Fassaden eines Gebäudes in einer (zumeist 4m) oder mehreren Ebenen eines Gebäudes und Abwicklung von Einzelpunkten entlang der Fassadenelemente. In Anhang VI der Richtlinie 2002/49/EG ist festgelegt, dass die Anzahl der Personen, die in Gebäuden wohnen, so festzustellen ist, dass sie dem lauteste Fassadenpunkt in Bezug auf die Werte der Lärmindizes L_{den} und L_{night} zuzurechnen sind. Dieser Vorgang dient auch dem Vergleich mit Dosis-Wirkungskurven, die unter denselben Annahmen getroffen werden.

Verwendete Lärmdaten

1. Die Ausgangslage

Die Erstellung von Lärmkarten (Raster- und Gebäudelärmkarten) stellt einen erheblichen personellen und Ressourceneinsatz dar. Das Amt der Tiroler Landesregierung, Abteilung ESA, ist für die Erstellung von strategischen Lärmkarten zuständig. In periodischen Abständen von fünf Jahren sind Umgebungslärmkarten zu überprüfen und erforderlichenfalls neu zu erstellen. Die ständige Entwicklung der Verkehre aber auch der Bebauung sowie das stete Bestreben, die Genauigkeit der Lärmkarten zu verbessern macht de facto die Neuberechnung alle fünf Jahre notwendig. Dabei werden alle Landesstraßen mit einem Verkehrsaufkommen über 3 Mio KFZ/a kartiert, zusätzlich der gesamte Straßenverkehr im Ballungsraum Innsbruck (zu dem auch die Gemeinden Völs und Rum zählen). Über gesonderte Beauftragung des Bundes erfolgen weiters Berechnungen des Schienenverkehrs im Ballungsraum und kombinierte Straßenlärmarten mit dem ASFINAG Straßennetz A und S für das gesamte Landesgebiet. Die verpflichtende periodische Berechnung war 2017 wieder fällig, die Datengrundlage dazu wurde bundeseinheitlich mit dem Jahr 2015 festgelegt.

Für die Gesamtlärmbetrachtung Innsbruck ergeben sich durch diesen Umstand erhebliche Synergien. Die gesamte Datengewinnung und –Aufbereitung konnte in aktueller Form genutzt werden. Dies betraf die gesamte Modellbildung des Stadtgeländes, der Bebauung, der statistischen Bevölkerungsdaten und der Schallschutzmaßnahmen. Es bedurfte daher nur der zusätzlichen Bearbeitungsschritte mit Modellierung und Schallausbreitungsberechnung der ÖBB-Strecken in Innsbruck sowie des Flugverkehrs, welcher neben der Gesamtlärmbetrachtung auch zur Evaluierung der Fluglärmmessstellen und der Radardatenerfassung diente.

2. Berechnung als geeignetes Verfahren

Der Bestimmung der Exposition kommt in dieser wie auch in vergleichbaren Studien eine besondere Bedeutung zu. Dabei kommt es darauf an, die gewählten Kennzahlen für die Exposition bestmöglich im Sinne von „mit den kleinsten Unsicherheiten behaftet“ abzubilden. Für diese Studie wurde entschieden, die Exposition mittels der europäisch festgelegten und international anerkannten Lärmindizes für die allgemeine Lärmbelastung L_{den} und für die Schlafstörung L_{night} abzubilden.

Grundsätzlich stehen für die Ermittlung der Schalleinwirkungen 3 Möglichkeiten zur Verfügung: Messen, Rechnen und Schätzen. Letzteres Verfahren wird zumeist eher belächelt, findet aber gerade in sehr weitläufigen Studien und als Post hoc-Analysemethode immer noch Anwendung. Im allgemeinen genießt die Messung in der Bevölkerung ein wesentlich größeres Vertrauen, dieses äußert sich auch in der jahrelangen Judikatur, welche postuliert, dass grundsätzlich der Messung der Vorzug zu geben ist. Bei näherer Betrachtung erweist sich die Methode der Messung jedoch bei weitem nicht als universell anwendbare bessere Ermittlungsmethode. Zur Bestimmung der europäischen Lärmindizes sind Durchschnittsbetrachtungen über 1 Jahr notwendig. Diese gelten für den lautesten Punkt der Fassade eines jeden Gebäudes. Die Exposition ist notwendiger Weise für alle Quellen des Verkehrslärms getrennt zu bestimmen. Die Messung schöpft vergleichsweise genaue Ergebnisse, deren Gültigkeit sich aber auf den Ort und den Zeitraum der Erfassung beschränkt. Aufgezeichnet werden hier

alle einzelnen Geräusche zusammen, selbst wenn diese als Störgeräusche zu qualifizieren wären und dadurch das Messergebnis verfälscht wird.

Die Berechnung stützt sich auf eine Vielzahl von Emissionsdatenmessungen, welche den Durchschnitt von einzelnen Fahrzeugen, sei es auf der Schiene, der Straße oder in der Luft sehr gut annähern. Die Transmissionseigenschaft ist Physik und die mathematische Umsetzung der Gesetzmäßigkeiten. Diesbezüglich ist kein Grund für Unzulänglichkeiten der Berechnung angebracht, vorausgesetzt das Modell wurde mit Sorgfalt, akustischer Expertise und realitätsnah erstellt. Einen unschlagbaren Vorteil zeigt die Berechnung gegenüber der Messung auf jeden Fall: Im Zuge der vorliegenden Untersuchung wurde an den Fassadenpunkten über 280.000-mal der Jahresdurchschnitt für die unterschiedlichen Quellen berechnet. Eine Anzahl, die einer messtechnischen Verfassung nicht zugänglich ist.

All diese Auswahlgründe und Argumente sprechen für die Berechnung, und zwar ausschließlichen Berechnung der Schallexposition an den betroffenen Fassaden, da Messungen weder die separierte Erfassung der Lärmquellen leisten können noch in Summe für die expositionsrichtige Darstellung leistbar im Sinne des zeitlichen und finanziellen Aufwandes sind.

3. Umgebungslärmkarten

3.1. Umfang

Die Bearbeitung zum Projekt *Gesamtlärmbetrachtung Innsbruck* umfasst die Berechnung und Darstellung von strategischen Umgebungslärmkarten, sowie die Ermittlung von betroffenen Einwohnerinnen und Einwohnern für die Lärmquellen Straße, Schiene und Flug im Stadtgebiet Innsbruck in einer Immissionshöhe von 4 m über Gelände.

Die Berechnung erfolgte unter Anwendung einer Software für die Schallausbreitung. Hierbei wurde ein Berechnungsmodell gebildet, in das alle für die weitere Berechnung relevanten Daten einflossen. Die größten Datenblöcke bilden das Gelände, sämtliche Gebäude (Immissionsorte) und die jeweiligen Emissionen.

3.2. Angaben zur eingesetzten Software

Programm: SoundPLAN

Version: 7.4

Hersteller: Braunstein + Berndt GmbH

D-71522 Backnang

Deutschland

3.3. Angaben zur Modellbildung

3.3.1. Beschreibung der Eingangsdaten Gelände

Das Geländemodell gründet auf einer aus der Laser-Scan-Verortung resultierenden 3D-Punktwolke, welche nach definierten Anforderungen für das vorliegende Projektgebiet aufbereitet wurde. Diese Laser-Scan-Verortung wurde im Auftrag des Landes in den Jahren 2012 bis 2015 durchgeführt.

Als maximaler Vertikalabstand zwischen zwei Höhenpunkten wurde ein Abstand von 0,1 m gewählt. Zudem wurde ein maximaler Horizontalabstand von 10 m berücksichtigt, um bei ausgedehnten ebenen Flächen ebenfalls ausreichend Höhenpunkte zu erhalten.

3.3.2. Beschreibung der Eingangsdaten Gebäude

Bei der Auswertung der Gebäude wurde das durch die Laser-Scan-Verortung festgestellte Gebäudepolygon in seinem Umfang um einen Meter deshalb reduziert, damit ein etwaiger Bodenstich der Laser-Scan-Verortung eliminiert wird. Ein solcher würde sich maßgeblich auf die Berechnung der Gebäudehöhe auswirken.

Um die Höhe des Gebäudes zu erhalten, wurde der arithmetische Mittelwert aus Höhenminimum und Höhenmaximum der Stiche innerhalb des reduzierten Gebäudepolygons gebildet.

In weiterer Folge wurde jedes Gebäudepolygon mit einer eindeutigen Adresse behaftet. Bei Gebäuden mit mehreren Adressen (z.B. Wohnanlagen) ergaben sich gemäß der Anzahl der Adresscodes zusätzliche Gebäudepolygone, welche geometrisch vollständig ident sind.

Die Bestimmung der betroffenen Einwohner erfolgte mittels des Verschnitts der Adresse mit den seitens des Umweltbundesamtes zur Verfügung gestellten AGRWII-Daten. So wurde jedem Gebäudepolygon im Vorfeld der Modellierung als zusätzliches Attribut die Einwohner- sowie Wohnungsanzahl zugeordnet.

Alle Gebäude im Modell weisen somit ihre vollständige, tatsächliche Adresse samt der Anzahl an Wohnungen und Bewohnerinnen und Bewohnern auf.

3.3.3. Beschreibung der Eingangsdaten Lärmschutz

Die Lärmschutzwände im Bereich der Landes- und Gemeindestraßen wurden im Zeitraum 2014 bis 2015 vor Ort erhoben. Lärmschutzwände die in den Zuständigkeitsbereich der ASFINAG bzw. den ÖBB fallen, wurden von diesen im Zuge des Datenaustausches im Jahr 2016 zur Verfügung gestellt.

Den Nachweis der Absorptionswerte der Lärmschutzwände lieferten die Prüfzeugnisse der jeweiligen Hersteller.

3.3.4. Beschreibung der Eingangsdaten Bodendämpfung

Die generelle Bodendämpfung wurde mit G = 0,8 angenommen. Befestigte Flächen wie z.B. Fahrwege oder Gewässer wurden mit G = 0,0 modelliert.

3.3.5. Anzahl der berücksichtigten Reflexionen und Rasterweite

Bei der Berechnung wurden Reflexionen 1. Ordnung berücksichtigt. Die Rasterweite betrug 10 m x 10 m.

3.3.6. Angaben zur grenzüberschreitenden Betrachtung von Lärmquellen

Eine grenzüberschreitende Betrachtung von Lärmquellen wurde durch eine aus schalltechnischer Sicht ausreichend über das Stadtgebiet hinweglaufende Modellierung der jeweiligen Lärmquelle gewährleistet.

4. Verkehrslärm

4.1. Straßenverkehrslärm

4.1.1. Angaben zur Erhebung der Emissionsquellen

Sowohl das Straßennetz im Projektgebiet als auch die Verkehrszahlen wurden seitens der Stadt Innsbruck zur Verfügung gestellt und gehen auf das Jahr 2015 zurück.

Bei den beiden Autobahnen wurden seitens der ASFINAG im Jahr 2016 im Zuge eines Datenaustausches die Emissionsschallpegel $L_{A,eq}$ übermittelt. Dies sind bereits die auf den Verkehrsdaten der Autobahn basierenden schalltechnischen Kenngrößen.

4.1.2. Angaben zu den verwendeten Geschwindigkeiten

Als Geschwindigkeiten wurden entsprechend der ÖAL-Richtlinie 36 Blatt 2 die jeweils erlaubten Höchstgeschwindigkeiten modelliert.

4.1.3. Beschreibung der Eingangsdaten Straßenemissionen

Die einzelnen Straßenzüge wurden auf das Geländemodell aufgesetzt und in weiterer Folge in dieses eingerechnet. Die Verkehrsstärken, Geschwindigkeiten und Fahrbahnoberflächen wurden als Parameter hinterlegt. Ebenso wurden sämtliche Brücken sowie die Tunnel im Bereich der Autobahnen berücksichtigt.

Als Berechnungsvorschrift für die Emission diente die RVS 04.02.11.

4.2. Schienenverkehrslärm

4.2.1. Angaben zur Erhebung der Emissionsquellen

Sowohl das Schienennetz im Projektgebiet als auch die Verkehrszahlen klassifiziert nach den einzelnen Zugarten wurden seitens der ÖBB im Jahr 2016 zur Verfügung gestellt.

4.2.2. Angaben zu den verwendeten Geschwindigkeiten

Als Geschwindigkeit wurde das Minimum aus zulässiger Höchstgeschwindigkeit im jeweiligen Streckenabschnitt und maximaler Höchstgeschwindigkeit der jeweiligen Zugart herangezogen.

4.2.3. Beschreibung der Eingangsdaten Schienenemissionen

Die einzelnen Schienenstrecken wurden auf das Geländemodell aufgesetzt und in weiterer Folge in dieses eingerechnet. Die Verkehrsstärken und Geschwindigkeiten wurden als Parameter hinterlegt. Ebenso wurden sämtliche Brücken sowie die Tunnels berücksichtigt. Als Berechnungsvorschrift für die Emission diente die ON-Regel 305011.

4.3. Fluglärm

4.3.1. Angaben zur Erhebung der Emissionsquellen

Durch die radarunterstützte Flugüberwachung der Austro Control GmbH werden alle Flüge vom und zum Flughafen Innsbruck mittels einer Flugspur beschrieben. Diese Flugspur beschreibt die x- und y-Position des Fluges, aber auch die Flughöhe und Geschwindigkeit des Flugzeuges. Abseits dieser Radardaten sind jedem Flug weitere Informationsdaten wie z.B. die Flugzeugtype hinterlegt. Sowohl Radardaten als auch Fluginformationsdaten werden von der Austro Control GmbH laufend im Rahmen der Fluglärmüberwachung dem Land Tirol übermittelt. Für die Gesamtlärmbetrachtung Innsbruck wurden alle Flüge des Jahres 2015 herangezogen.

4.3.2. Beschreibung der Eingangsdaten Flugemissionen

Die Flugbewegungen wurden entsprechend ihren Flugspuren im Modell abgebildet. Flüge, die nicht dem Flughafen zuzuordnen sind (Überflüge, Hubschrauberflüge) wurden vorab herausgefiltert. Anhand der Flugzeugtype und des Steigprofils wurden die Emissionen nach der Richtlinie ECAC Doc 29 3rd Edition berechnet. Nicht modelliert werden konnten somit die Flugbewegungen der Rettungshubschrauber vom und zum Landeskrankenhaus Innsbruck. Diese stellen besonders in den Wintermonaten im Tageszeitraum eine erhebliche Anzahl an Flügen und verursachen relevante Lärmimmissionen im Stadtgebiet von Innsbruck. Allerdings sind bis dato keine exakten Flugbewegungs- respektive Lärmdaten verfügbar, um diese in die Flugverkehrslärmausbreitungsberechnungen aufnehmen zu können.

4.4. Betroffenenauswertung - Bestimmung der betroffenen Einwohner

Die im weiteren Verlauf berechneten Gebäudelärmkarten lieferten die Ausweisung der betroffenen Einwohnerinnen und Einwohner, sowie der Wohnungen. Bei der Berechnung der Gebäudelärmkarten wurde an jeder Fassade mit Länge >1,5 m in deren Mitte ein Immissionspunkt auf Höhe 4 m über Gelände gesetzt. Es erfolgten getrennte Rechenläufe für die drei unterschiedlichen Emissionsarten.

Als Ergebnis wurde für jede einzelne Adresse der lauteste Fassadenpegel für Straße, Schiene und Flug ausgewiesen. Die Zuordnung der Einwohnerinnen und Einwohner und Wohnungen erfolgte zu jeweils diesem Pegel.

5. Nicht verkehrsinduzierter Lärm

Gesamtlärm im weiteren Sinn inkludiert nicht nur Geräusche von Verkehrsträgern sondern auch aus anderen Quellen. Es ist dies im Speziellen Lärm aus Gewerbe und Industrie, aus Gaststätten und von Passanten, von Baustellen und aus der Nachbarschaft. Die flächendeckende Bestimmung von Schallquellen erfordert das Vorhandensein von entsprechenden Eingangsdaten, welche in vielen Fällen nicht oder nur mit einem hohen Aufwande erhoben werden können. Eine Bestimmung der Exposition in Form der in Dezibel ausgedrückten Schallbelastung erfolgte nicht. Im Fragebogen werden diese Quellen dennoch in zweierlei Hinsicht berücksichtig. Einerseits wird die Ausgesetztheit zu diesen Quellen analog zum Verkehrslärm befragt, andererseits erfolgt ebenso die Abfrage der Belästigung und subjektiven Schlafstörung. Damit ist eine Auswertungsmöglichkeit auch für nichtverkehrsinduzierten Lärm möglich.

6. Messung in Kranebitten

6.1. Lärmmessung

Wie weiter oben ausgeführt, genießt die Messung allgemein ein höheres Vertrauen als die Berechnung. Auf ausdrücklichen Wunsch eines Projektpartners wurde an einem ausgewählten Messpunkt in Kranebitten eine temporäre Dauermessstelle installiert, um einen Vergleich gerade in Bezug auf Fluglärm durchführen zu können. Der Messpunkt befand sich auf Gst.Nr. 2541/3, KG Hötting, ca. 25m östlich des Gebäudes Harterhofweg 99 und ca. 20m südlich der Weggabelung. Die Mikrofonhöhe betrug 4m über Gelände. Die Ergebnisse der Lärmmessung repräsentieren einen Durchschnittstag im Zeitraum von Montag, den 23.01.2017 10:42 Uhr bis Sonntag, den 29.01.2017 24:00 Uhr.

Die Messergebnisse wurden maßgeblich vom Flugverkehrsaufkommen am Flughafen Innsbruck, aber auch dem Straßenverkehr entlang der A12 Inntalautobahn und der B171 Tiroler Straße beeinflusst.

Tabelle 1: Messergebnisse temporäre Dauermessstelle Kranebitten (alle Quellen)

Tag (06:00 – 19:00 Uhr):	60,5 dB
Abend (19:00 – 22:00 Uhr):	57,6 dB
Nacht (22:00 – 06:00 Uhr):	47,8 dB

Aus diesen Messergebnissen wurden die Flugbewegungen extrahiert und ausschließlich die Schallimmissionen des Flugverkehrs ermittelt, damit ein Vergleich mit den Berechnungsergebnissen genau für diesen Zeitabschnitt durchgeführt werden konnte. Die Teilpegel der einzelnen Quellen sind in folgender Tabelle aufgelistet:

Tabelle 2: Teillärmpegel temporäre Dauermessstelle Kranebitten

	Tag	Abend	Nacht 8h
Straße	58,6 dB	55,6 dB	49,7 dB
Schiene	39,7 dB	40,4 dB	40,0 dB
Flug	54,4 dB	47,2 dB	32,8 dB
**Summe Berechnung**	**60,0 dB**	**56,3 dB**	**50,2 dB**
**Summe Messung**	**60,5 dB**	**57,6 dB**	**47,8 dB**
**Differenz Ber. – Mess.**	**-0,5 dB**	**-1,3 dB**	**2,4**

Abweichungen im oben beschriebenen Umfang von -0,5 bis 2,4 dB liegen in einem sehr guten Genauigkeitsbereich.

6.2. Vergleich Messung – Berechnung Fluglärm

Bei den folgenden Ergebnissen wurde sowohl bei der Lärmmessung als auch bei der Berechnung ausschließlich das Flugverkehrsaufkommen am Flughafen Innsbruck berücksichtigt. Der Zeitraum beschränkt sich auf die Woche der Lärmmessung von Montag 23.01.2017 bis Sonntag 29.01.2017:

Tabelle 3: Vergleich der Mess- mit den Berechnungsergebnissen

	Tag	Abend	Nacht
Messung	59,0 dB	56,0 dB	33,8 dB
Berechnung	58,1 dB	52,0 dB	30,4 dB
**Differenz**	**0,9 dB**	**4,0 dB**	**3,4 dB**

Die Unterschiede zwischen Messung und Berechnung im Abend- und Nachtzeitraum lassen sich auf Unschärfen in der Auswertung des Pegel-Zeit-Verlaufes der Messung (händische Zuordnung) bzw. auch auf Ungenauigkeiten der Radarspurkoordinaten zurückführen. Durch das deutlich geringere Flugverkehrsaufkommen im Abend- und Nachtzeitraum schlagen einzelne Ungenauigkeiten, das sind Abweichungen auch der Emissionsdaten der Flugzeuge nach der Berechnungsvorschrift zum tatsächlichen Emissions- und Flugverhalten wesentlich stärker durch.

Dass die Messergebnisse höher als die Berechnungsergebnisse sind, erscheint aber plausibel, da die Berechnung ausschließlich das spezifische Geräusch berücksichtigt, während bei einer Lärmmessung sämtliche Umgebungseinflüsse miterfasst werden und die Trennschärfe zwischen Fluglärmerfassung und Hintergrundgeräuschen nicht exakt ist.

In Summe gibt die Berechnung in Zusammenschau mit den Dauermessstellen, welche beide radardatenbasiert die Flugereignisse korrelieren ein sehr gutes reales Belastungsbild in der Fläche, das sich auch durch weitere Messstellen nicht entscheidend verbessern lässt.

Gesamtlärmberechnung

Die Frage nach Möglichkeiten die Methoden und Evidenz von Gesamtlärmberechnungen ist aktuell in der Lärmwirkungsforschung sehr umstritten. Mit der vorliegenden Gesamtlärmbetrachtung in Innsbruck sollen auch Daten gewonnen werden, welche für die Erhöhung der Evidenz und die Generierung eines verbesserten Gesamtlärmmodels geeignet sind und so unterstützend wirken können. Im Rahmen des vorliegenden Projektberichtes wird aber kein neues Verfahren abgeleitet, sondern ein bestehendes Verfahren zur Anwendung gebracht.

Im *Good practice guide on noise exposure and potential health effects* der Europäischen Umweltagentur (eea technical report No 11/2010) ist ein Verfahren, basierend auf Veröffentlichungen von Henk Miedema enthalten (*Relationship between exposure to multiple noise sources and noise annoyance* J. Acoust. Soc. Am. Volume 116, Issue 2, pp. 949-957 - 2004). Auch in Deutschland wurde ein darauf aufbauendes und durchaus vergleichbares Verfahren entwickelt, welches in VDI 3722-2 beschrieben ist. Die Unterschiede dieser Verfahren untereinander sind marginal und führt die Anwendung des einen oder anderen Verfahrens nicht zu relevant unterschiedlichen Aussagen. Hervorzuheben ist, dass diese Verfahren zur Anwendung in der strategische Planung und Hotspot-Analyse gedacht sind, nicht bzw. noch nicht für die Bewertung individueller Lärmstörungen. Untersuchungen und Studien über die Beeinträchtigung durch mehrere Quellen, die darauf fokussieren, wie die betrachteten Geräuschquellenarten bewertet wurden und welche Geräuschquelle die vorherrschende innerhalb der Gesamtgeräuschsituation ist, sind schwieriger. Der Stand der Forschung ist laut VDI 3722-2 und der darin zitierten Literaturrecherche von B. Schulte-Fortkamp eindeutig: es gibt hierzu keine generelle Antwort.

Das Verfahren der VDI 3722-2 dient der Ermittlung von Kenngrößen zur Bewertung beim Einwirken unterschiedlicher Geräuschquellenarten hinsichtlich Belästigung und selbstberichteter Schlafstörungen. Das Verfahren selbst ist ein Substitutionsverfahren, das die Festsetzung einer Vergleichsgröße erfordert. Für die Wirkung kombinierter Verkehrsgeräuschquellenarten wird der Straßenverkehr herangezogen, dieser dient quasi als Referenzgeräusch zur Bewertung der relativen Belästigungs- und subjektiven Schlafstörungswirkungen. Letztendlich wird das Model der VDI 3722-2 auch deshalb zur Anwendung gebracht, da diese den in deutschsprachigem Raum gegebenen Stand der Technik darstellt. Der Stand des Wissens ist erst weiter zu verfeinern und zu vertiefen *„es wird sich daher erst in Zukunft zeigen, welche Modelle zur Vorhersage einer Gesamtlärmbeeinträchtigung durch mehrere Quellenarten am besten geeignet sind“* (VDI3722-2). Dieser Stand der Technik ist auch in der zur Anwendung gelangenden Software SoundPlan 7.4 realisiert. Aus diesem weiteren Grund war dies das Mittel der Wahl, die gesamte Lärmbelastung vor allem zum Zwecke der Verteilung und verbesserten Analyse der Betroffenheiten anzuwenden.

LÄRM IN INNSBRUCK

Die Ist-Situation

1. Belastungszonen

Wie im Bereich der statistischen Aufarbeitungen noch weiter erläutert, erfolgt die Zuordnung zu Belastungszonen über das Ausmaß der Betroffenheit durch Lärm aus einzelnen Quellen. Dabei wird in die drei Kategorien „gering“; „mittel“ und „stark“ unterschieden. Die folgenden Tabellen und Grafiken geben einen Überblick über diese Belastungen getrennt nach Straßen- (hier nochmals unterteilt in Autobahn und Gesamtstraßenverkehr), Schienen- und Flugverkehr. Die Verkehrslärmbelastung der Einwohnerinnen und Einwohner von Innsbruck wird im nachfolgenden Kapitel *Verkehrslärmbelastung* beschrieben. In der nachstehender Tabelle ist die Anzahl der belasteten Gebäude und Wohnungen nach Quellen enthalten:

Tabelle 4: Anzahl der belasteten Gebäude, Einwohner und Wohnungen nach Quelle

		Gering	Mittel	Stark	Gesamt
**Flug**	Gebäude	6257	4886	807	11950
	Wohnungen	34127	34881	4303	73311
**Schiene**	Gebäude	6968	3709	1273	11950
	Wohnungen	39153	25901	8257	73311
**Straße**	Gebäude	967	4804	6179	11950
	Wohnungen	2198	25186	45927	73311
**Autobahn**	Gebäude	6334	4868	748	11950
	Wohnungen	36915	32842	3554	73311

Abbildung 6: Überblick der Verkehrslärmbelastung nach Quelle

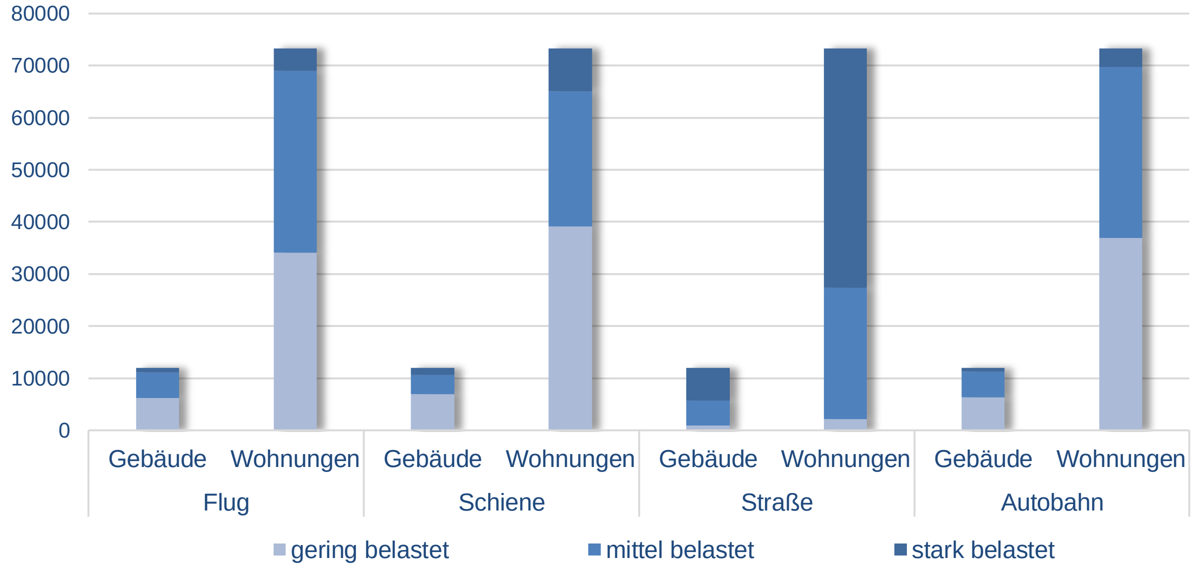

In dieser Graphik wird deutlich, dass der größte Anteil der stark belasteten Wohnungen durch den Straßenverkehr (gesamt) entsteht. Diese Aussage gilt gleichermaßen für Gebäude wie Wohnungen. Mehr als 50 % der Gebäude und 60% der Wohnungen in Innsbruck sind durch Straßenverkehr stark belastet, vergleichsweise sind hier Flug mit 7%, Schiene mit 11% Anteil der stark belasteten Gebäude und Wohnungen nur im wesentlich untergeordneten Ausmaß beteiligt. Dies ist auch aus folgender Tabelle ersichtlich.

Tabelle 5: Anteile der belasteten Gebäude, Einwohnerinnen und Einwohner und Wohnungen bezogen auf ganz Innsbruck nach Quelle in Prozent

		**Gering**	**Mittel**	**Stark**
**Flug**	Gebäude	52%	41%	7%
	Wohnungen	47%	48%	6%
**Schiene**	Gebäude	58%	31%	11%
	Wohnungen	53%	35%	11%
**Straße**	Gebäude	8%	40%	52%
	Wohnungen	3%	34%	63%
**Autobahn**	Gebäude	53%	41%	6%
	Wohnungen	50%	45%	5%

Nachstehende Abbildung zeigt die prozentuelle Verteilung der Belastung auf die Gebäude in Innsbruck nach Quellen:

Abbildung 7: Prozentuelle Verteilung der Belastung der Gebäude und Wohnungen in Innsbruck nach Quelle

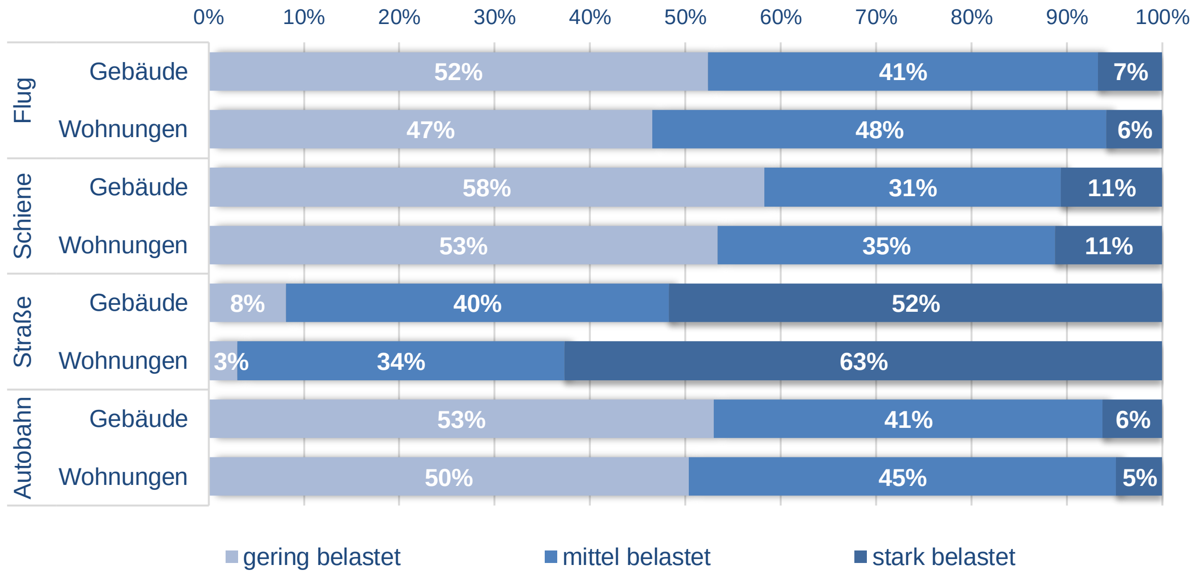

Im Überblick zeigt sich, dass hinsichtlich der Schallbelastung ausgedrückt als L_{den} der Straßenverkehr insgesamt mit 62% den mit großem Abstand bedeutsamsten Teil ausmacht. Autobahn und Flug zeigen ein sehr ähnliches Bild was die Belastung betrifft. Diesbezüglich weicht auch die Schiene nur unerheblich ab.

Die folgenden Abbildungen zeigen die Rasterlärmkarten für den L_{den} getrennt nach Quellen unter Berücksichtigung der drei Kategorien für die Belastung „gering“, „mittel“ und „stark“:

Abbildung 8: Rasterlärmkarte 24h-Fluglärm L_{den} in Innsbruck gruppiert nach Belastungskategorien

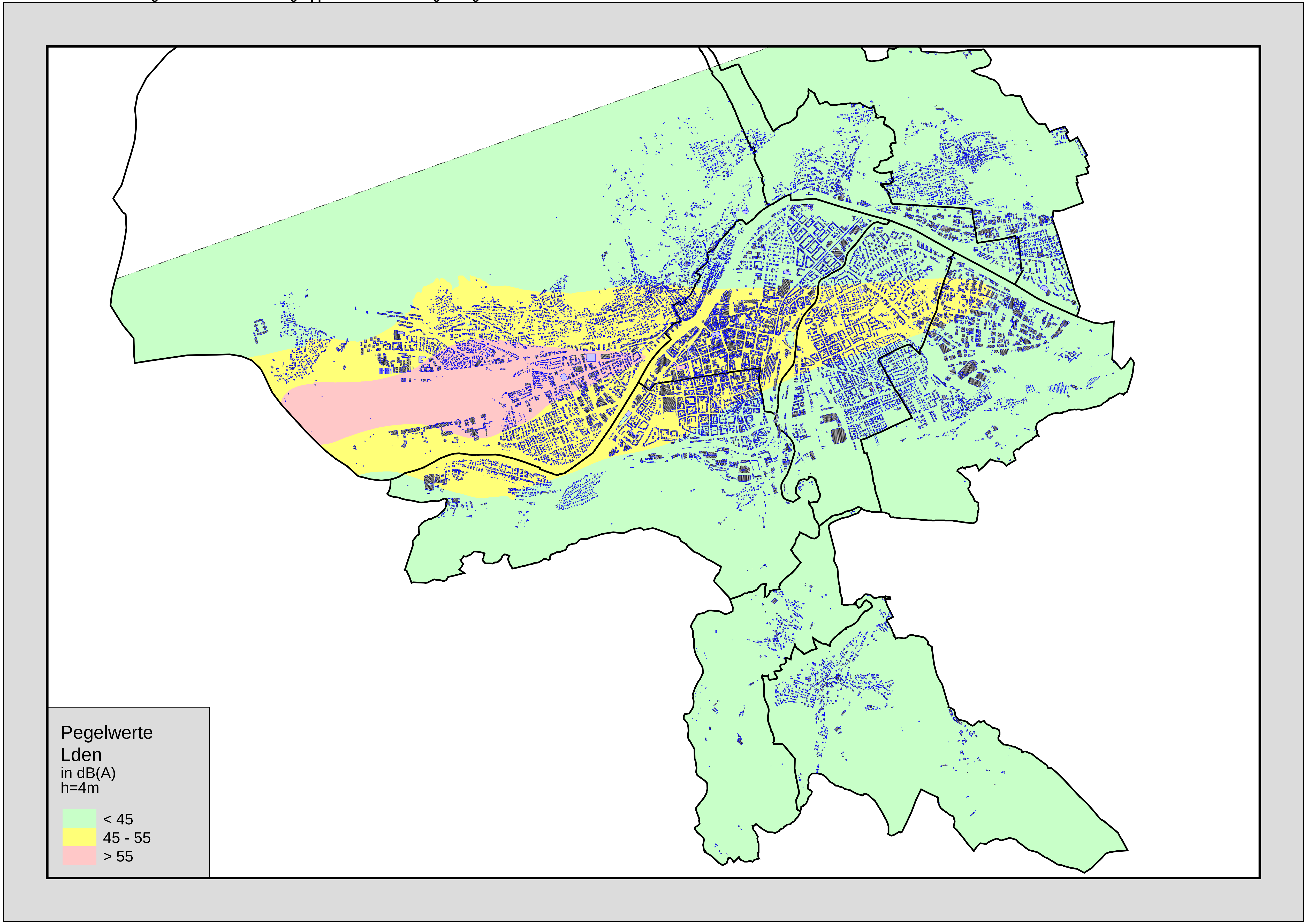

Diese Abbildung zeigt die unterschiedlichen Belastungssituationen durch den Flugbetrieb am Innsbrucker Flughafen. Während sich der Bereich der starken Belastung auf das unmittelbare Flughafenumfeld beschränkt, erstreckt sich das Gebiet der mittleren Belastung in West-Ostrichtung noch über einen weiten Bereich des Stadtgebietes.

Abbildung 9: Rasterlärmkarte 24h-Schienenverkehrslärm L_{den} in Innsbruck gruppiert nach Belastungskategorien

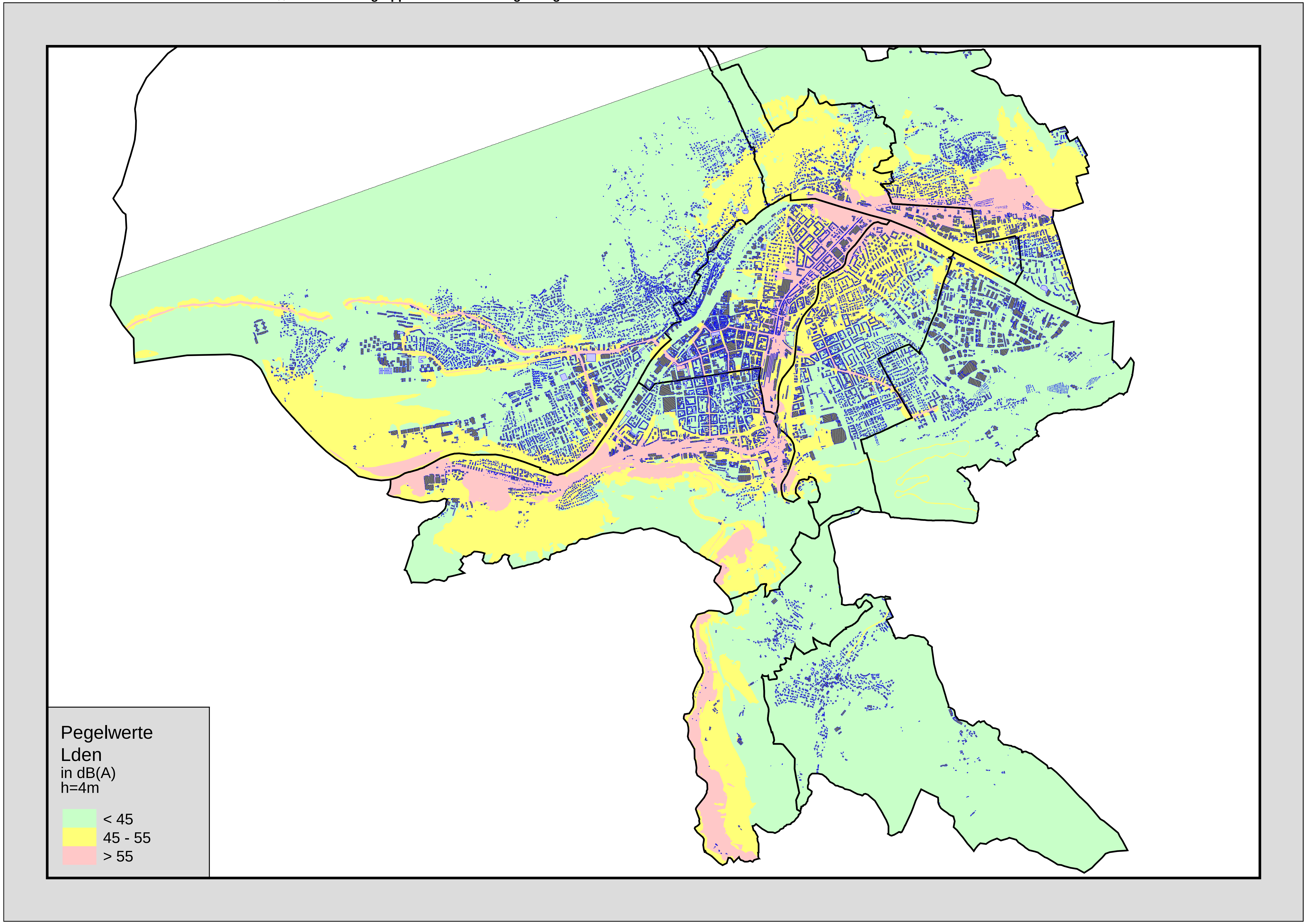

Deutlich erkennbar ist der Schienenkorridor von Ost nach West im Inntal mit der Abzweigung der Brennerbahn in Richtung Süden. Entlang dieses Korridors ergibt sich eine starke Belastung, der Abzweig der Karwendelbahn in Richtung Nordwest ist noch erkennbar, die Straßenbahn tritt optisch kaum in Erscheinung.

Abbildung 10: Rasterlärmkarte 24h-Straßenverkehrslärm L_{den} in Innsbruck gruppiert nach Belastungskategorien

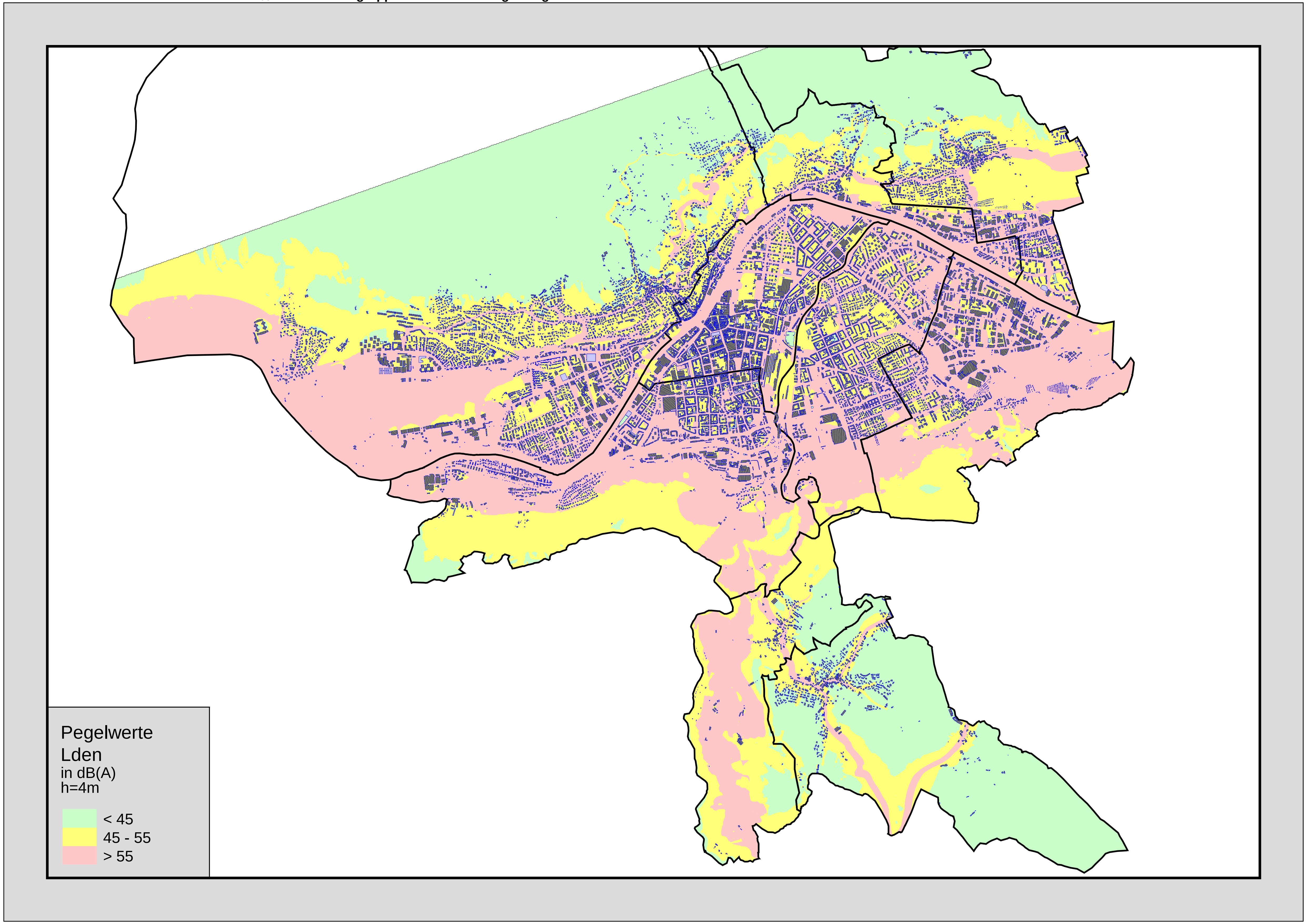

Auf dieser Überblickskarte erscheint der Straßenlärm als die dominierende Schallquelle in Innsbruck. Ausgenommen sind lediglich die südlichen Randgemeinden Igls und Vill, wie auch Teile von Mühlau und Arzl. Die Katastralgemeinden im Stadtkern weisen ein durchgehend hohes Belastungsbild auf. Die im Süden Innsbrucks vorbeiführende Autobahn A12, sowie auch der Abzweig der Brennerautobahn A13 belasten vor allem die Stadtteile Amras, Pradl und Wilten, ebenso wie einen Teil von Hötting. Lärm von der Brennerautobahn wirkt sich vor allem auf unbesiedeltes Gebiet in der Katastralgemeinde Vill aus.

Verkehrslärmbelastung

Die Unterteilung und Auswertung erfolgte geographisch nach den Katastralgemeinden Innsbrucks:

Abbildung 11: Übersicht der ausgewerteten Katastralgemeinden in Innsbruck

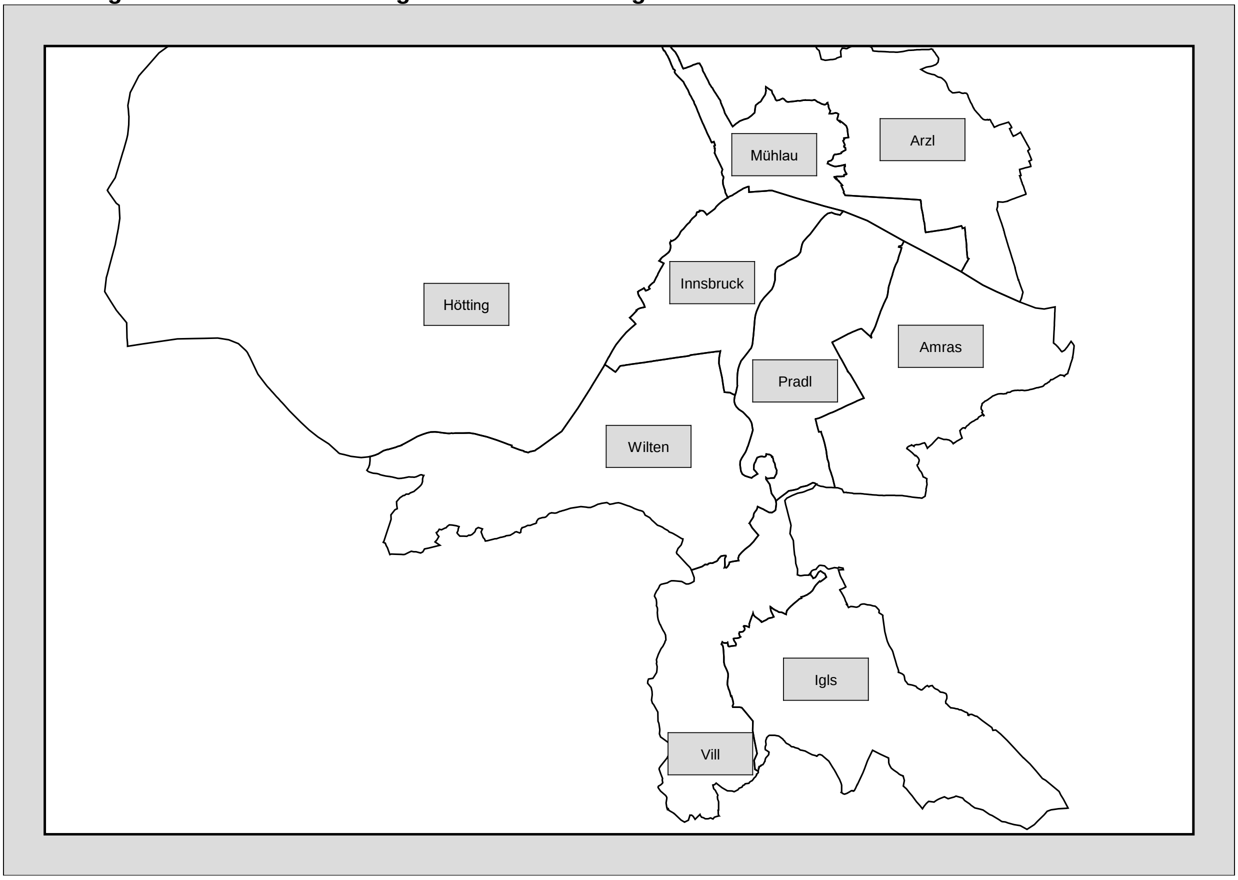

1. 24h-Verkehrslärmbelastung

1.1. Vergleich Innsbruck gesamt – befragtes Kollektiv

Im Mittelwertvergleich der Lärmbelastung nach Quelle zeigt sich eine Dominanz der Straßenverkehrslärmbelastung, welche im Mittel mit rund 55dB Dauerschallbelastung (L_{den}) auf die Gesamtinnsbrucker Wohngebiete einwirkt.

Mit deutlichem Abstand (mehr als 10dB und damit in ihrer Lautheit rund halb so laut[2]) folgen die weiteren Verkehrslärmquellen Fluglärm, Schienenverkehrslärm und Autobahnlärm, die in ihrem mittleren Immissionsdauerschallpegeln fast gleichauf liegen:

[2] Anm.: Die Wahrnehmung der Lautheit einer Schallquelle halbiert und verdoppelt sich in 10dB-Schritten.

Abbildung 12: Mittelwertvergleich der 24h-Lärmbelastung L_{den} nach Quelle in dB

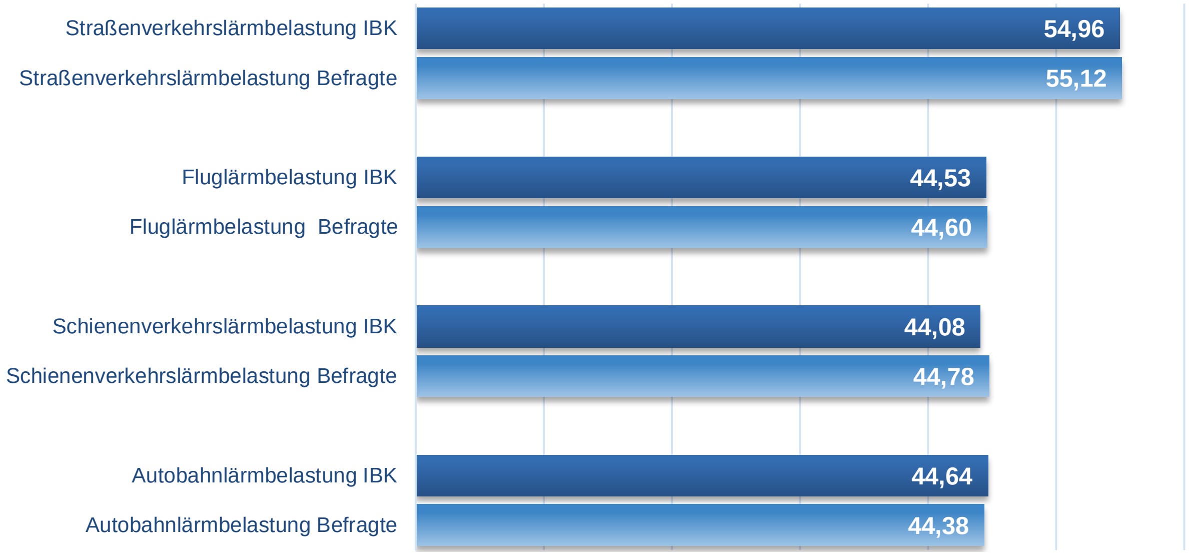

Weiters weist der Mittelwertvergleich auf, dass die Befragungen ein sehr exaktes Abbild der Gesamtlärmbelastungen Innsbrucks bezogen auf Verkehrslärmquellen widerspiegeln:

Tabelle 6: Mittelwerte der 24h-Lärmbelastung L_{den} nach Quelle in dB

	Straßenlärmbelastung IBK	Straßenlärmbelastung Befragte	Fluglärmbelastung IBK	Fluglärmbelastung Befragte	Schienenlärmbelastung IBK	Schienenlärmbelastung Befragte	Autobahnlärmbelastung IBK	Autobahnlärmbelastung Befragte
**MW**	**54,9620**	**55,1247**	**44,5260**	**44,6020**	**44,0790**	**44,7757**	**44,6420**	**44,3756**
N	9711	1031	9711	1031	9711	1031	9711	1031
Median	54,8000	55,1000	44,6000	44,3000	43,1000	43,5000	44,4000	44,2000
SA	7,32110	7,44587	6,70670	6,97139	7,82080	7,62842	6,15680	6,00688
Min.	24,90	24,90	27,30	30,50	0,00	20,70	17,80	30,30
Max.	74,30	72,00	67,20	66,40	77,90	69,00	71,40	65,70

* Bezug Innsbruck (IBK): 9711 gültige Adresspunkte mit im ZMR ausgewiesenen Wohneinheiten – Bezug Befragte: 1031 vollständig geführte Interviews

Auch im absoluten Vergleich des Ausmaßes der Verkehrslärmbelastungen nach Quelle bleibt der Straßenverkehrslärm die dominante Lärmquelle. Während über die Hälfte der Innsbruckerinnen und Innsbrucker vom Flug-, Schienen- und Autobahnlärm nur gering (<45dB) belastet sind, fallen über 90% der Einwohnerinnen und Einwohner Innsbrucks beim Straßenverkehrslärm in die Kategorien der mittelgradig bzw. stark Belasteten.

Gruppiert in die drei Belastungskategorien:

- Gering belastet: unter 45dB
- Mittelgradig belastet: 45-55dB
- Stark belastet: über 55dB

stellt sich das Ausmaß der Dauerlärmbelastung wie folgt dar:

Abbildung 13: Vergleich gruppierte Häufigkeit der 24h-Lärmbelastung L_{den} nach Quelle in Prozent

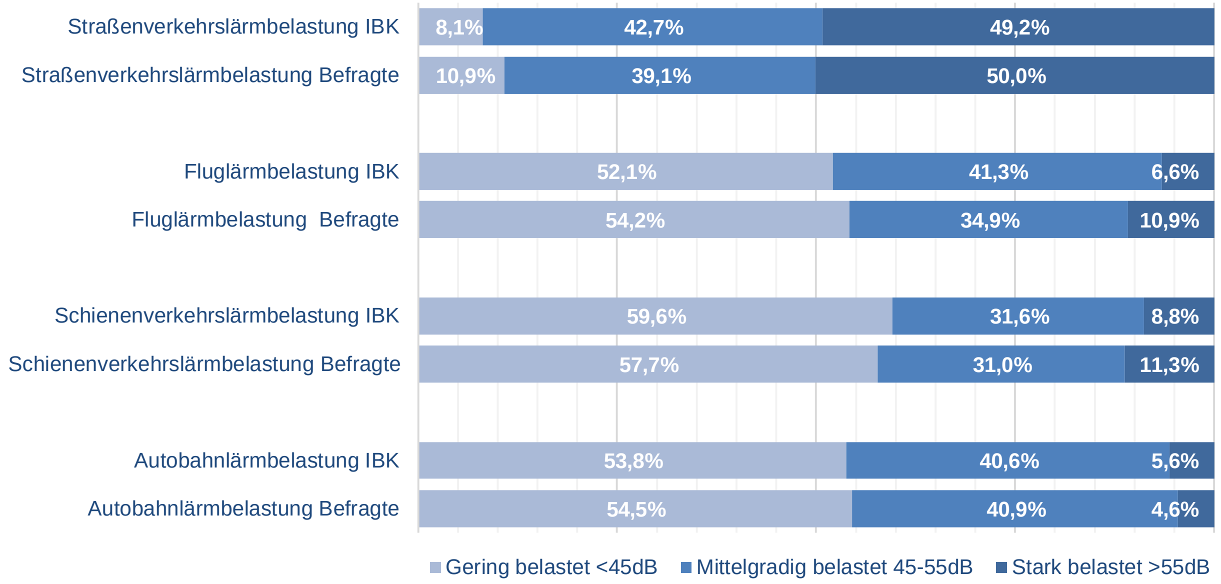

* Bezug Innsbruck (IBK): 9711 gültige Adresspunkte mit im ZMR ausgewiesenen Wohneinheiten – Bezug Befragte: 1031 vollständig geführte Interviews

Wiederum zeigt sich die hohe Repräsentativität der Befragungen in Bezug zur Grundgesamtheit der zu Wohnzwecken benutzten Innsbrucker Gebäude.

1.2. 24h-Verkehrslärmbelastung nach Katastralgemeinden

Der Straßenverkehrslärm ist besonders in den Katastralgemeinden Wilten, Innsbruck, Pradl und Amras sehr ausgeprägt und liegt in diesen Stadtteilen über dem Schwellenwert von 55dB Dauerschallbelastung. Der Fluglärm belastet besonders Hötting und reicht dort im Mittel knapp an die 50dB-Schwelle:

Abbildung 14: Mittelwertvergleich der 24h-Lärmbelastung L_{den} bewohnter Gebäude der Innsbrucker Katastralgemeinden nach Quelle in dB

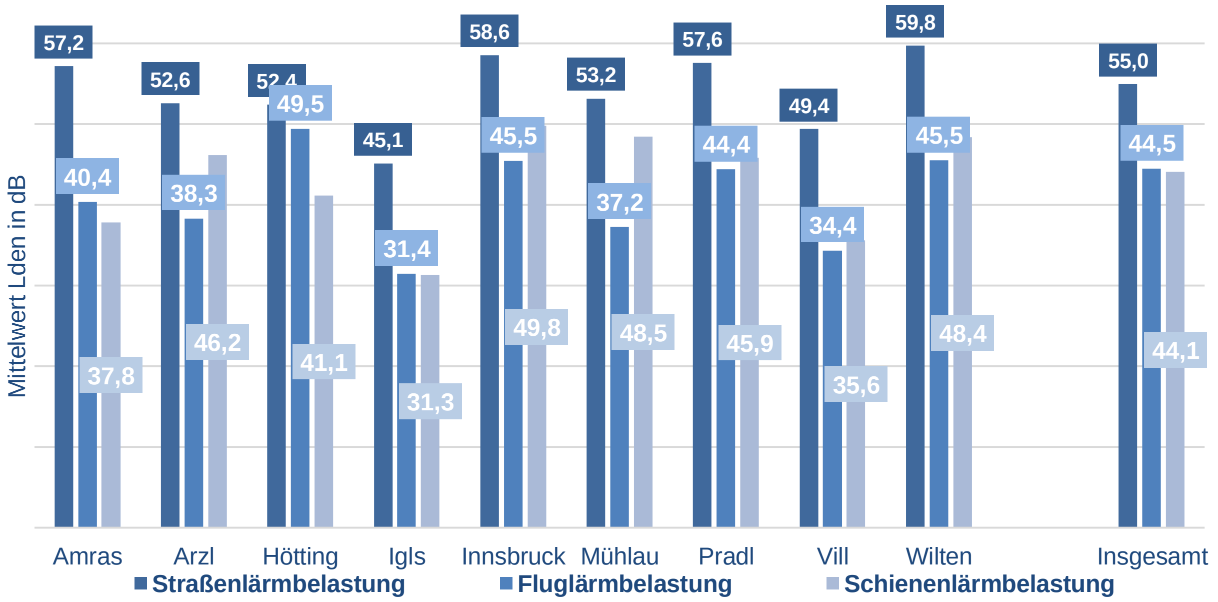

Der Schienenverkehrslärm sticht in den Katastralgemeinden Innsbruck, Mühlau und Wilten hervor, wo ebenfalls knapp eine 50dB-Dauerbelastung der Wohngebäude erreicht wird. Igls und Vill sind die ruhigsten Katastralgemeinden der Stadt Innsbruck.

In der Gegenüberstellung aller 9711 bewohnten Adresspunkte mit dem befragten Kollektiv (N=1031) zeigt sich erneut die sehr hohe Repräsentativität der befragten Stichprobe, auch bezogen auf die Lärmbelastung nach Verkehrsquelle der einzelnen Katastralgemeinden von Innsbruck:

Tabelle 7: Mittelwerte der 24h-Lärmbelastung L_{den} der Innsbrucker Katastralgemeinden nach Quelle in dB (Innsbruck gesamt und Befragte gegenübergestellt)

Katastralgemeinde		Straßenlärmbelastung IBK	Straßenlärmbelastung Befragte	Fluglärmbelastung IBK	Fluglärmbelastung Befragte	Schienenlärmbelastung IBK	Schienenlärmbelastung Befragte
**Amras**	N	496	60	496	60	496	60
	**Mittelwert (dB)**	**57,186**	**56,1133**	**40,350**	**40,8617**	**37,839**	**41,1600**
	Median	56,500	56,2000	39,900	40,2500	38,900	40,5000
	Standardabweichung	5,0737	4,30236	2,1609	2,21789	6,9256	5,56820
	Minimum	48,5	50,10	36,2	37,60	14,3	24,40
	Maximum	74,3	65,80	45,8	44,00	54,0	53,80
**Arzl**	N	890	102	890	102	890	102
	**Mittelwert**	**52,624**	**51,3667**	**38,315**	**38,6657**	**46,153**	**44,9137**
	Median	51,800	51,9000	37,500	37,4000	45,200	44,2000
	Standardabweichung	6,3688	6,11990	2,8300	3,48691	5,5886	5,58685
	Minimum	24,9	24,90	31,5	31,60	32,5	32,50
	Maximum	71,8	63,70	44,3	44,20	72,8	61,10
**Hötting**	N	3178	243	3178	243	3178	243
	**Mittelwert**	**52,441**	**52,2103**	**49,454**	**51,5391**	**41,134**	**40,4959**
	Median	52,100	52,7000	49,200	52,7000	40,200	39,7000
	Standardabweichung	6,6220	7,66306	6,8507	8,01928	5,4833	5,23246
	Minimum	31,8	32,00	32,2	33,30	11,2	27,00
	Maximum	69,9	67,50	67,2	66,40	59,9	58,30
**Igls**	N	435	35	435	35	435	35
	**Mittelwert**	**45,142**	**42,1229**	**31,448**	**31,6971**	**31,329**	**31,9771**
	Median	43,600	41,3000	31,400	31,8000	33,300	32,4000
	Standardabweichung	7,4565	4,85575	1,1296	,64784	7,7740	5,48249
	Minimum	30,9	33,80	27,3	30,50	0,0	20,70
	Maximum	64,5	57,50	35,1	33,20	42,6	39,30
**Innsbruck**	N	1240	176	1240	176	1240	176
	**Mittelwert**	**58,567**	**58,4915**	**45,481**	**45,1858**	**49,818**	**50,2267**
	Median	59,200	57,4000	45,700	44,9000	50,500	51,8000
	Standardabweichung	6,5932	6,49518	4,1193	3,87952	7,9702	8,12525
	Minimum	44,9	45,40	38,5	39,10	36,2	36,50
	Maximum	70,9	70,80	54,0	53,50	73,3	64,80
**Mühlau**	N	429	50	429	50	429	50
	**Mittelwert**	**53,191**	**49,6020**	**37,243**	**35,6180**	**48,483**	**48,5660**
	Median	53,200	47,9500	36,500	35,6000	48,400	49,3500
	Standardabweichung	9,1892	6,56448	3,1156	,75015	4,8228	2,27045
	Minimum	33,9	39,40	32,0	34,30	33,1	41,60
	Maximum	72,9	68,80	44,3	37,70	68,2	51,90
**Pradl**	N	1649	188	1649	188	1649	188
	**Mittelwert**	**57,594**	**58,1899**	**44,390**	**44,3064**	**45,889**	**46,2085**
	Median	56,800	56,8000	44,800	44,6000	45,500	45,3500
	Standardabweichung	5,4493	5,54823	2,5472	2,37319	5,0690	5,38786
	Minimum	44,2	46,40	38,6	39,10	34,8	36,10
	Maximum	73,6	72,00	49,0	48,80	64,2	64,20

Vill	N	143	21	143	21	143	21
	**Mittelwert**	**49,402**	**48,9143**	**34,373**	**34,5000**	**35,626**	**34,9762**
	Median	48,900	47,6000	34,200	34,4000	34,000	34,1000
	Standardabweichung	5,7367	3,76076	1,0497	,51088	6,9741	2,30280
	Minimum	37,9	44,00	30,3	34,10	26,6	32,80
	Maximum	69,1	56,50	36,9	36,10	77,9	41,20
Wilten	N	1251	156	1251	156	1251	156
	**Mittelwert**	**59,766**	**59,7724**	**45,523**	**45,9487**	**48,377**	**47,8417**
	Median	59,100	59,0500	45,500	46,3000	47,600	45,1000
	Standardabweichung	5,1347	4,20240	2,3930	2,19770	7,6643	7,69641
	Minimum	46,8	50,90	36,6	40,70	36,5	37,80
	Maximum	73,5	68,50	50,7	50,30	75,2	69,00
Insge-samt	N	9711	1031	9711	1031	9711	1031
	**Mittelwert**	**54,962**	**55,1247**	**44,526**	**44,6020**	**44,079**	**44,7757**
	Median	54,800	55,1000	44,600	44,3000	43,100	43,5000
	Standardabweichung	7,3211	7,44587	6,7067	6,97139	7,8208	7,62842
	Minimum	24,9	24,90	27,3	30,50	0,0	20,70
	Maximum	74,3	72,00	67,2	66,40	77,9	69,00

Wird die Verkehrslärmbelastung gruppiert in 3 Belastungskategorien und nach Quelle dargestellt, zeigen sich im Extremgruppenvergleich der stark belasteten Adresspunkte (Dauerschallpegel L_{den} >55dB) noch deutlicher die bereits weiter oben beschriebenen Unterschiede zwischen den einzelnen Katastralgemeinden:

Abbildung 15: Stark verkehrslärmbelastete Adresspunkte in Innsbruck nach Quelle und Katastralgemeinde in Prozent

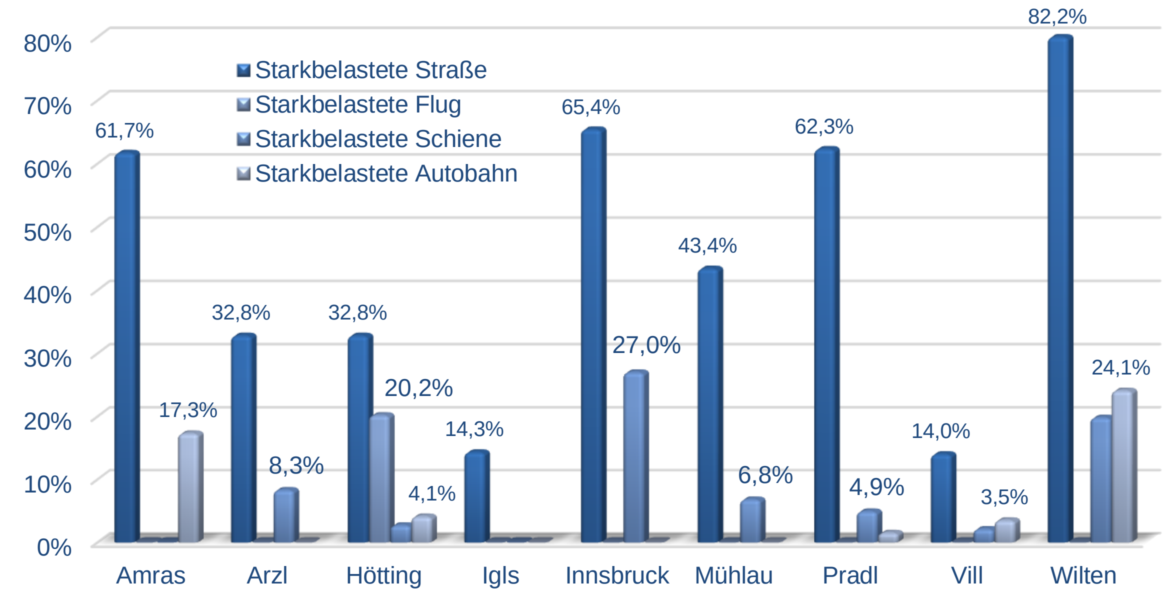

Über 80% aller Adresspunkte in Wilten sind stark vom Straßenverkehrslärm belastet, in den Katastralgemeinden Innsbruck, Pradl und Amras sind es über 60%, in Igls und Vill hingegen nur 14%. Vom Fluglärm starkbelastete Wohngebäude sind ausschließlich in Hötting vorhanden (20,2% aller bewohnten Adresspunkte). Der Schienenverkehrslärm belastet in Innsbruck und Wilten rund ein Viertel aller Gebäude stark. Die Autobahn wirkt sich vor allem in Wilten (24%) und Amras (17,3%) stark lärmbelastend aus:

Tabelle 8: Straßenverkehrslärmbelastung L_{den} in 3 Belastungskategorien nach Katastralgemeinde

		Katastralgemeinde								
		Amras	Arzl	Hötting	Igls	Innsbruck	Mühlau	Pradl	Vill	Wilten
Starkbelastete > 55dB	Anzahl	306	292	1041	62	811	186	1027	20	1028
	Prozent	61,7%	32,8%	32,8%	14,3%	65,4%	43,4%	62,3%	14,0%	82,2%
Mittelbelastete 45-55dB	Anzahl	190	530	1798	116	428	152	620	91	223
	Prozent	38,3%	59,6%	56,6%	26,7%	34,5%	35,4%	37,6%	63,6%	17,8%
Geringbelastete < 45db	Anzahl	0	68	339	257	1	91	2	32	0
	Prozent	0,0%	7,6%	10,7%	59,1%	0,1%	21,2%	0,1%	22,4%	0,0%
Gesamt	Anzahl	496	890	3178	435	1240	429	1649	143	1251
	Prozent	100,0%	100,0%	100,0%	100,0%	100,0%	100,0%	100,0%	100,0%	100,0%

Tabelle 9: Fluglärmbelastung L_{den} in 3 Belastungskategorien nach Katastralgemeinde

		Katastralgemeinde								
		Amras	Arzl	Hötting	Igls	Innsbruck	Mühlau	Pradl	Vill	Wilten
Starkbelastete > 55dB	Anzahl	0	0	643	0	0	0	0	0	0
	Prozent	0,0%	0,0%	20,2%	0,0%	0,0%	0,0%	0,0%	0,0%	0,0%
Mittelbelastete 45-55dB	Anzahl	12	0	1841	0	637	0	774	0	748
	Prozent	2,4%	0,0%	57,9%	0,0%	51,4%	0,0%	46,9%	0,0%	59,8%
Geringbelastete < 45db	Anzahl	484	890	694	435	603	429	875	143	503
	Prozent	97,6%	100,0%	21,8%	100,0%	48,6%	100,0%	53,1%	100,0%	40,2%
Gesamt	Anzahl	496	890	3178	435	1240	429	1649	143	1251
	Prozent	100,0%	100,0%	100,0%	100,0%	100,0%	100,0%	100,0%	100,0%	100,0%

Tabelle 10: Schienenverkehrslärmbelastung L_{den} in 3 Belastungskategorien nach Katastralgemeinde

		Katastralgemeinde								
		Amras	Arzl	Hötting	Igls	Innsbruck	Mühlau	Pradl	Vill	Wilten
Starkbelastete > 55dB	Anzahl	0	74	87	0	335	29	80	3	248
	Prozent	0,0%	8,3%	2,7%	0,0%	27,0%	6,8%	4,9%	2,1%	19,8%
Mittelbelastete 45-55dB	Anzahl	43	398	552	0	487	300	805	3	484
	Prozent	8,7%	44,7%	17,4%	0,0%	39,3%	69,9%	48,8%	2,1%	38,7%
Geringbelastete < 45db	Anzahl	453	418	2539	435	418	100	764	137	519
	Prozent	91,3%	47,0%	79,9%	100,0%	33,7%	23,3%	46,3%	95,8%	41,5%
Gesamt	Anzahl	496	890	3178	435	1240	429	1649	143	1251
	Prozent	100,0%	100,0%	100,0%	100,0%	100,0%	100,0%	100,0%	100,0%	100,0%

Tabelle 11: Autobahnlärmbelastung L_{den} in 3 Belastungskategorien nach Katastralgemeinde

		Katastralgemeinde								
		Amras	Arzl	Hötting	Igls	Innsbruck	Mühlau	Pradl	Vill	Wilten
Starkbelastete > 55dB	Anzahl	86	0	129	0	0	0	25	5	302
	Prozent	17,3%	0,0%	4,1%	0,0%	0,0%	0,0%	1,5%	3,5%	24,1%
Mittelbelastete 45-55dB	Anzahl	399	62	1801	1	120	28	869	18	645
	Prozent	80,4%	7,0%	56,7%	0,2%	9,7%	6,5%	52,7%	12,6%	51,6%
Geringbelastete < 45db	Anzahl	11	828	1248	434	1120	401	755	120	304
	Prozent	2,2%	93,0%	39,3%	99,8%	90,3%	93,5%	45,8%	83,9%	24,3%
Gesamt	Anzahl	496	890	3178	435	1240	429	1649	143	1251
	Prozent	100,0%	100,0%	100,0%	100,0%	100,0%	100,0%	100,0%	100,0%	100,0%

2. Verkehrslärmbelastung in der Nacht

2.1. Vergleich Innsbruck gesamt – befragtes Kollektiv

Die Lärmbelastung nach Quelle verändert sich im Nachtzeitraum (22h bis 6h) stark. Der Straßenverkehrslärm bleibt die mit Abstand dominierende Lärmquelle, nimmt aber im Vergleich zu den 24h-Pegeln und damit vor allem gegenüber dem 24h-Zeitraum um rund 10dB ab und ist damit in seiner Lautheit halbiert.[3] Gleiches gilt für den Autobahnlärm.

Der Schienenverkehrslärm verringert sich im Nachtzeitraum ebenso, allerdings weniger deutlich als der Straßen- und Autobahnverkehrslärm. Im Mittelwertvergleich sinkt die Lärmbelastung der Innsbrucker Wohngebiete durch den Schienenverkehrslärm im Nachtzeitraum um rund 7dB:

Abbildung 16: Mittelwertvergleich der nächtlichen Lärmbelastung L_n nach Quelle in dB

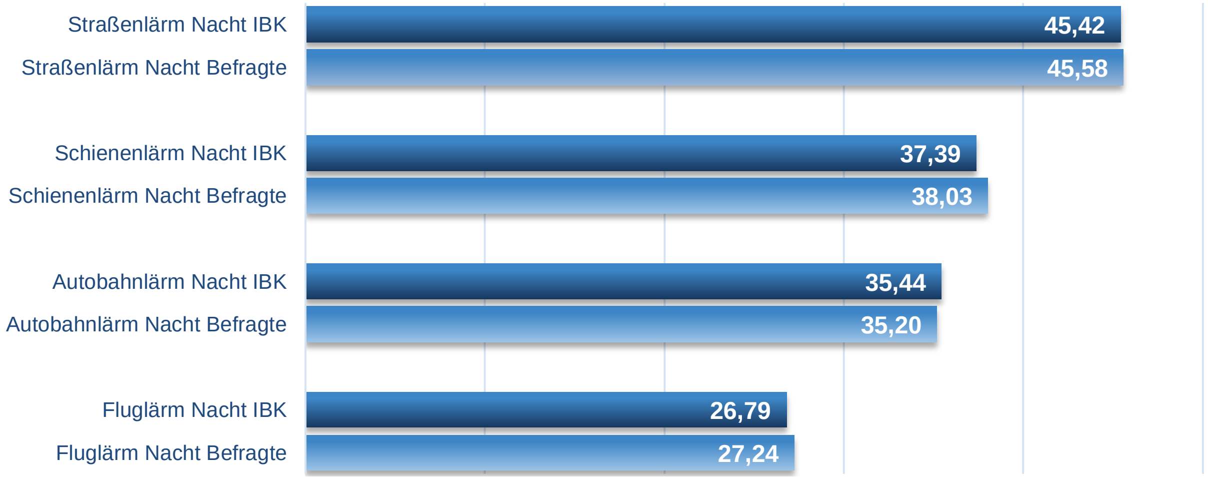

[3] Anm.: Die Wahrnehmung der Lautheit einer Schallquelle halbiert sich in 10dB-Schritten.

Am stärksten nimmt in den Nachtstunden die Lärmbelastung durch den Flugverkehr ab. Diese verringert sich um knapp 18dB gegenüber dem 24h-Pegel und trägt damit im Vergleich der einzelnen Verkehrslärmquellen mit Abstand am wenigsten zur Gesamtlärmbelastung Innsbrucks in der Nacht bei.

Auch der Mittelwertvergleich der Verkehrslärmquellen im Nachtzeitraum zwischen dem Gesamtkollektiv der 9711 bewohnten Adresspunkte in Innsbruck und den 1031 befragten Personen zeigt das sehr genaue Abbild der Grundgesamtheit durch die Stichprobe, das mit den Befragungen erreicht werden konnte:

Tabelle 12: Mittelwerte der nächtlichen Lärmbelastung L_n nach Quelle in dB

	Straßenlärm Nacht IBK	Straßenlärm Nacht Befragte	Schienenlärm Nacht IBK	Schienenlärm Nacht Befragte	Autobahnlärm Nacht IBK	Autobahnlärm Nacht Befragte	Fluglärm Nacht IBK	Fluglärm Nacht Befragte
**MW**	**45,4206**	**45,5835**	**37,3864**	**38,0290**	**35,4407**	**35,1979**	**26,7933**	**27,2378**
N	9711	1031	9711	1031	9711	1031	9711	1031
Median	45,4	45,6	36,5	36,9	35,1	35	27,1	27,6
SA	7,3539	7,4612	7,7932	7,5497	6,1288	5,9720	7,3489	7,4643
Min.	15,3	15,3	0	10,6	9,3	21,2	2,9	7,5
Max.	65,5	63,3	71,7	62,7	62,4	56,7	49,5	47,7

* Bezug Innsbruck (IBK): 9711 gültige Adresspunkte mit im ZMR ausgewiesenen Wohneinheiten – Bezug Befragte: 1031 vollständig geführte Interviews

Aus dem absoluten Vergleich des Ausmaßes der Verkehrslärmbelastungen in der Nacht nach Quelle geht erneut der Straßenverkehrslärm als dominante Lärmquelle hervor.

Über 50% der Innsbruckerinnen und Innsbrucker sind auch in der Nacht mittelgradig bzw. stark vom Straßenverkehrslärm belastet.

Beim Schienenverkehrslärm reduziert sich der Anteil der mittelgradig bzw. stark Belasteten in der Nacht auf unter 14%, bei den Autobahnlärmbelasteten auf unter 8%.

Stark fluglärmbelastete Adresspunkte gibt es im Nachtzeitraum in Innsbruck nicht, deutlich weniger als 1% sind als mittelgradig belastet einzustufen:

Gruppiert in die drei Belastungskategorien:

- Gering belastet: unter 45dB
- Mittelgradig belastet: 45-55dB
- Stark belastet: über 55dB

stellt sich das Ausmaß der Verkehrslärmbelastung in den Nachtstunden wie folgt dar:

Abbildung 17: Vergleich gruppierte Häufigkeit der nächtlichen Lärmbelastung L_n nach Quelle in Prozent

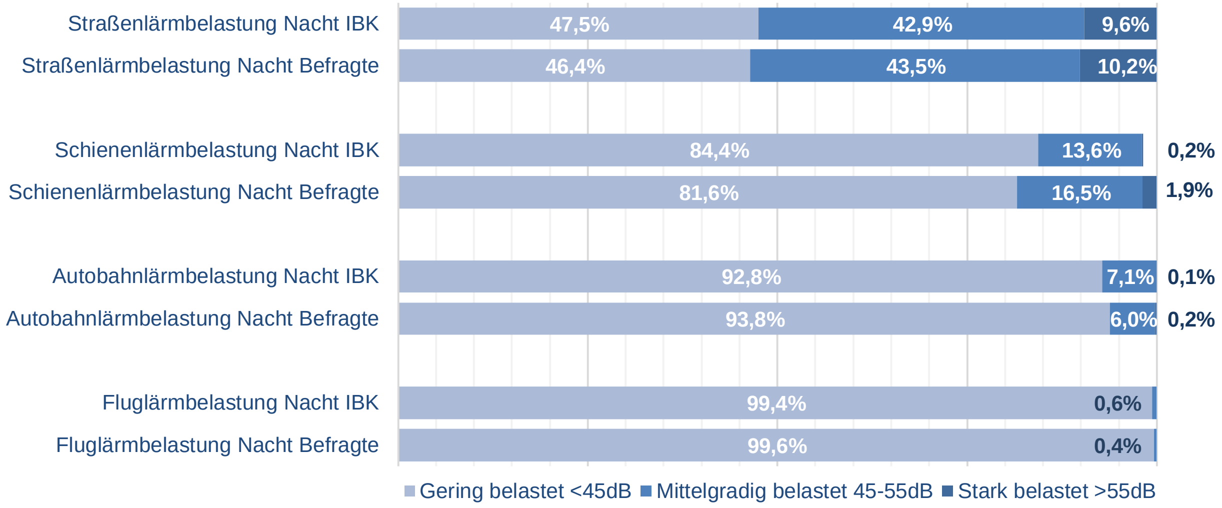

* Bezug Innsbruck (IBK): 9711 gültige Adresspunkte mit im ZMR ausgewiesenen Wohneinheiten – Bezug Befragte: 1031 vollständig geführte Interviews

Auch im Nachtzeitraum bildet das befragte Kollektiv die Verkehrslärmbelastungssituation nach Lärmquellen in Innsbruck insgesamt sehr gut ab.

2.2. Verkehrslärmbelastung in der Nacht nach Katastralgemeinden

Die nächtliche Straßenverkehrslärmbelastung liegt im Mittelwert aller bewohnten Innsbrucker Adresspunkte in Wilten am höchsten und dort auch über dem Schwellenwert von 50dB:

Abbildung 18: Mittelwertvergleich der nächtlichen Lärmbelastung L_n bewohnter Gebäude der Innsbrucker Katastralgemeinden nach Quelle in dB

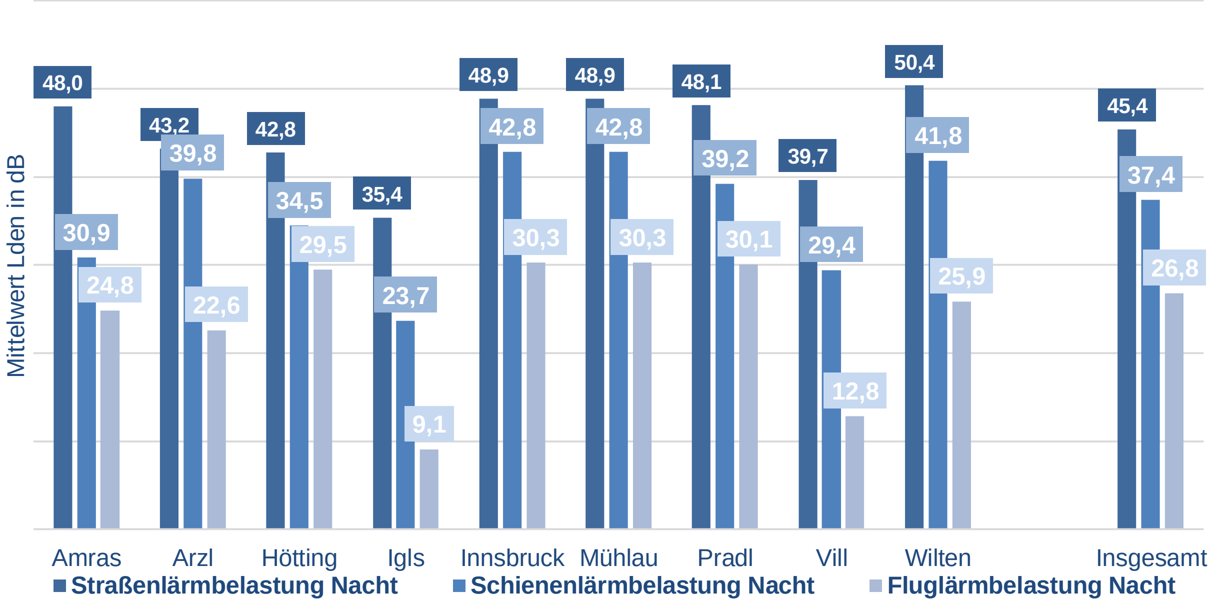

Der Flugverkehrslärm in der Nacht stellt im gesamten Stadtgebiet von Innsbruck keine erhebliche Belastung dar und sinkt auch in der im 24h-Pegel am stärksten belasteten Katastralgemeinde Hötting unter die 30dB-Pegelgrenze. Igls und Vill sind auch im Nachtzeitraum – sowohl nach einzelnen Verkehrsträgern, als auch insgesamt – die ruhigsten Katastralgemeinden der Stadt Innsbruck.

Im Detail ist die Gegenüberstellung aller 9711 bewohnten Adresspunkte Innsbrucks mit dem befragten Kollektiv (N=1031) geordnet nach Verkehrslärmquellen aus der nachfolgenden Tabelle abzulesen:

Tabelle 13: Mittelwerte der nächtlichen Lärmbelastung L_n der Innsbrucker Katastralgemeinden nach Quelle in dB (Innsbruck gesamt und Befragte gegenübergestellt)

Katastralgemeinde		Straßenlärm Nacht IBK	Straßenlärm Nacht Befragte	Schienenlärm Nacht IBK	Schienenlärm Nacht Befragte	Fluglärm Nacht IBK	Fluglärm Nacht Befragte
**Amras**	N	496	60	496	60	496	60
	**Mittelwert in dB**	**48,025**	**46,8500**	**30,854**	**33,9667**	**24,847**	**25,4967**
	Median	47,450	46,6000	32,150	33,8500	24,100	24,3000
	Standardabweichung	5,1096	4,27831	6,7256	4,90516	3,1161	3,10177
	Minimum	39,4	41,00	3,1	17,90	17,6	20,50
	Maximum	65,5	56,80	44,2	44,00	33,1	30,00
**Arzl**	N	890	102	890	102	890	102
	**Mittelwert**	**43,209**	**41,9284**	**39,838**	**38,6000**	**22,592**	**23,1039**
	Median	42,400	42,1000	38,950	37,8000	21,100	21,0000
	Standardabweichung	6,3116	6,08937	5,5787	5,57645	4,3017	5,24559
	Minimum	15,3	15,30	26,3	26,30	11,7	12,60
	Maximum	63,0	54,80	66,5	54,80	31,5	31,40
**Hötting**	N	3178	243	3178	243	3178	243
	**Mittelwert**	**42,770**	**42,5391**	**34,476**	**33,8374**	**29,474**	**31,4189**
	Median	42,400	43,2000	33,700	33,1000	28,800	32,4000
	Standardabweichung	6,6180	7,64341	5,2817	4,99631	7,0919	8,21588
	Minimum	21,9	22,20	,9	20,50	12,1	13,40
	Maximum	60,3	57,80	53,4	51,90	49,5	47,70
**Igls**	N	435	35	435	35	435	35
	**Mittelwert**	**35,401**	**32,3857**	**23,707**	**24,3114**	**9,100**	**9,3629**
	Median	33,800	31,6000	25,700	25,2000	9,300	9,7000
	Standardabweichung	7,3991	4,84040	7,6207	5,75616	1,4287	1,02959
	Minimum	21,2	24,00	0,0	10,60	2,9	7,50
	Maximum	54,6	47,70	33,8	31,90	12,9	11,30
**Innsbruck**	N	1240	176	1240	176	1240	176
	**Mittelwert**	**48,880**	**48,7943**	**42,820**	**43,3034**	**30,285**	**29,9455**
	Median	49,500	47,7000	43,400	44,2000	29,650	29,0500
	Standardabweichung	6,5632	6,48376	7,8254	8,04606	5,3037	4,95196
	Minimum	35,2	35,70	29,7	30,00	21,4	22,40
	Maximum	61,3	61,10	66,9	58,40	40,7	40,30
**Mühlau**	N	429	50	429	50	429	50
	**Mittelwert**	**43,811**	**40,1020**	**42,171**	**42,2560**	**20,378**	**18,0180**
	Median	43,800	38,8000	42,100	43,0500	19,200	17,9000
	Standardabweichung	9,2773	6,46250	4,8089	2,26465	4,9396	1,03227
	Minimum	24,3	29,90	26,8	35,30	11,7	16,20
	Maximum	64,2	59,20	61,8	45,50	31,4	21,10
**Pradl**	N	1649	188	1649	188	1649	188
	**Mittelwert**	**48,134**	**48,7793**	**39,245**	**39,4037**	**30,091**	**29,8798**
	Median	47,300	47,1500	38,900	38,8000	30,700	30,9000
	Standardabweichung	5,4362	5,57047	4,8588	5,10951	3,8449	3,63655
	Minimum	34,8	37,10	28,2	29,40	19,8	21,80
	Maximum	64,9	63,30	57,9	57,90	36,1	36,00
**Vill**	N	143	21	143	21	143	21
	**Mittelwert**	**39,691**	**39,1762**	**29,438**	**28,8048**	**12,849**	**12,8333**
	Median	39,000	37,7000	27,800	27,9000	12,900	13,0000

	Standardabweichung	5,6878	3,78958	6,9836	2,29401	1,6706	1,29009
	Minimum	28,2	34,30	19,8	26,70	8,2	11,00
	Maximum	59,2	47,10	71,7	35,00	16,0	15,70
**Wilten**	N	1251	156	1251	156	1251	156
	**Mittelwert**	**50,379**	**50,3353**	**41,815**	**41,1045**	**25,882**	**26,7635**
	Median	49,700	49,7000	41,100	38,8000	25,900	26,5500
	Standardabweichung	5,1468	4,14134	7,6783	7,67305	3,6059	3,78287
	Minimum	37,2	41,60	30,0	31,30	15,8	19,40
	Maximum	64,7	59,70	68,9	62,70	33,7	33,10
**Insgesamt**	N	9711	1031	9711	1031	9711	1031
	**Mittelwert**	**45,421**	**45,5835**	**37,386**	**38,0290**	**26,793**	**27,2378**
	Median	45,400	45,6000	36,500	36,9000	27,100	27,6000
	Standardabweichung	7,3539	7,46123	7,7932	7,54968	7,3489	7,46429
	Minimum	15,3	15,30	0,0	10,60	2,9	7,50
	Maximum	65,5	63,30	71,7	62,70	49,5	47,70

Wird die nächtliche Verkehrslärmbelastung gruppiert in 3 Belastungskategorien und nach Quelle dargestellt, zeigt sich im Extremgruppenvergleich der stark belasteten Adresspunkte (Schallpegel L_n >55dB), dass die Katastralgemeinde Innsbruck den höchsten Anteil an stark vom Straßenverkehrslärm in der Nacht belasteten Wohngebäuden aufweist:

Abbildung 19: In der Nacht stark verkehrslärmbelastete Adresspunkte in Innsbruck nach Quelle und Katastralgemeinde in Prozent

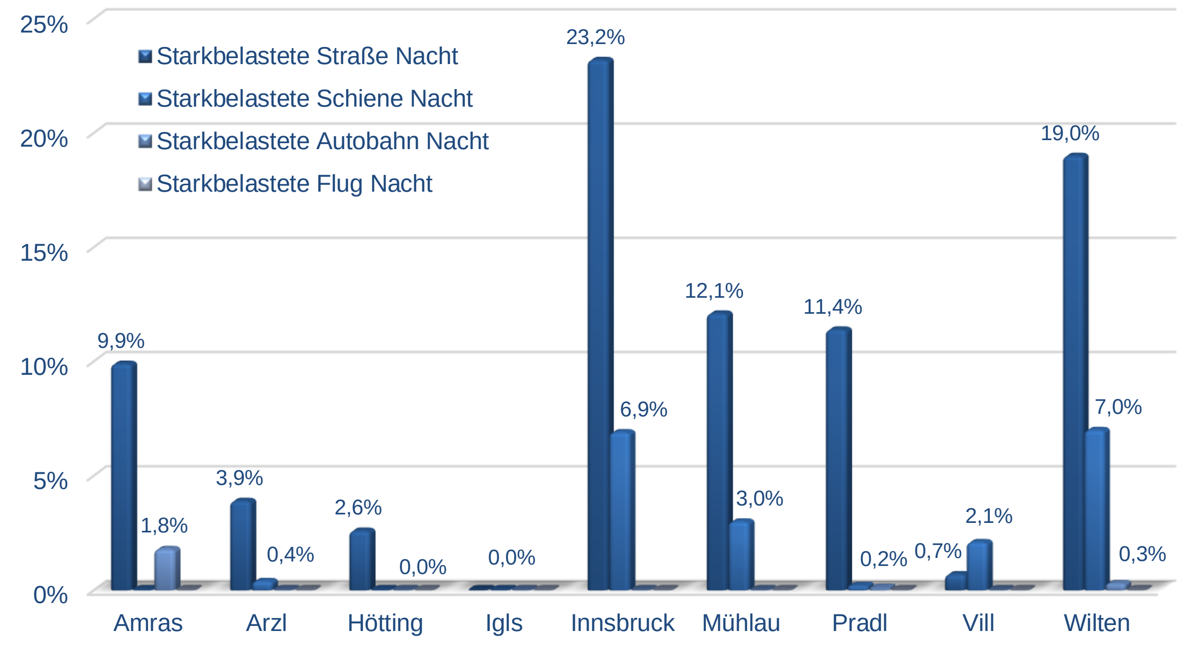

Auch in Wilten ist eine erhebliche Anzahl von Adresspunkten (238 Wohngebäude, was einem Anteil von 19% entspricht) auch in der Nacht starken Lärmpegeln aus dem Straßenverkehr ausgesetzt. Weiters sind Innsbruck und Wilten zum Teil (rund 7%) in der Nacht stark schienenverkehrslärmbelastet.

Stark vom Autobahnlärm in der Nacht belastete Wohngebäude treten nur vereinzelt auf und liegen nur in den Katastralgemeinden Amras (9 Wohngebäude), Wilten (4 Wohngebäude) und Pradl (1 Wohngebäude). Stark vom Flugverkehrslärm belastete Gebäude gibt es im Nachtzeitraum in Innsbruck nicht. In Igls ist unabhängig von der Lärmquelle kein Adresspunkt von Nachtlärmpegeln über 55dB betroffen:

Tabelle 14: Nächtliche Straßenlärmbelastung L_n in 3 Belastungskategorien nach Katastralgemeinde

		Katastralgemeinde								
		Amras	Arzl	Hötting	Igls	Innsbruck	Mühlau	Pradl	Vill	Wilten
Starkbelastete > 55dB	Anzahl	49	35	84	0	288	52	188	1	238
	Prozent	9,9%	3,9%	2,6%	0,0%	23,2%	12,1%	11,4%	0,7%	19,0%
Mittelbelastete 45-55dB	Anzahl	290	271	1023	63	565	146	924	21	860
	Prozent	58,5%	30,4%	32,2%	14,5%	45,6%	34,0%	56,0%	14,7%	68,7%
Geringbelastete < 45db	Anzahl	157	584	2071	372	387	231	537	121	153
	Prozent	31,7%	65,6%	65,2%	85,5%	31,2%	53,8%	32,6%	84,6%	12,2%
Gesamt	Anzahl	496	890	3178	435	1240	429	1649	143	1251
	Prozent	100,0%	100,0%	100,0%	100,0%	100,0%	100,0%	100,0%	100,0%	100,0%

Tabelle 15: Nächtliche Schienenlärmbelastung L_n in 3 Belastungskategorien nach Katastralgemeinde

		Katastralgemeinde								
		Amras	Arzl	Hötting	Igls	Innsbruck	Mühlau	Pradl	Vill	Wilten
Starkbelastete > 55dB	Anzahl	0	4	0	0	85	13	3	3	87
	Prozent	0,0%	0,4%	0,0%	0,0%	6,9%	3,0%	0,2%	2,1%	7,0%
Mittelbelastete 45-55dB	Anzahl	0	138	142	0	437	77	190	0	339
	Prozent	0,0%	15,5%	4,5%	0,0%	35,2%	17,9%	11,5%	0,0%	27,1%
Geringbelastete < 45db	Anzahl	496	748	3036	435	718	339	1456	140	825
	Prozent	100,0%	84,0%	95,5%	100,0%	57,9%	79,0%	88,3%	97,9%	65,9%
Gesamt	Anzahl	496	890	3178	435	1240	429	1649	143	1251
	Prozent	100,0%	100,0%	100,0%	100,0%	100,0%	100,0%	100,0%	100,0%	100,0%

Tabelle 16: Nächtliche Autobahnlärmbelastung L_n in 3 Belastungskategorien n. Katastralgemeinde

		Katastralgemeinde								
		Amras	Arzl	Hötting	Igls	Innsbruck	Mühlau	Pradl	Vill	Wilten
Starkbelastete > 55dB	Anzahl	9	0	0	0	0	0	1	0	4
	Prozent	1,8%	0,0%	0,0%	0,0%	0,0%	0,0%	0,1%	0,0%	0,3%
Mittelbelastete 45-55dB	Anzahl	96	10	158	0	0	0	51	6	367
	Prozent	19,4%	1,1%	5,0%	0,0%	0,0%	0,0%	3,1%	4,2%	29,3%
Geringbelastete < 45db	Anzahl	391	880	3020	435	1240	429	1597	137	880
	Prozent	78,8%	98,9%	95,0%	100,0%	100,0%	100,0%	96,8%	95,8%	70,3%
Gesamt	Anzahl	496	890	3178	435	1240	429	1649	143	1251
	Prozent	100,0%	100,0%	100,0%	100,0%	100,0%	100,0%	100,0%	100,0%	100,0%

Tabelle 17: Nächtliche Fluglärmbelastung L_n in 3 Belastungskategorien nach Katastralgemeinde

		Katastralgemeinde								
		Amras	Arzl	Hötting	Igls	Innsbruck	Mühlau	Pradl	Vill	Wilten
Mittelbelastete 45-55dB	Anzahl	0	0	58	0	0	0	0	0	0
	Prozent	0,0%	0,0%	1,8%	0,0%	0,0%	0,0%	0,0%	0,0%	0,0%
Geringbelastete < 45db	Anzahl	496	890	3120	435	1240	429	1649	143	1251
	Prozent	100,0%	100,0%	98,2%	100,0%	100,0%	100,0%	100,0%	100,0%	100,0%
Gesamt	Anzahl	496	890	3178	435	1240	429	1649	143	1251
	Prozent	100,0%	100,0%	100,0%	100,0%	100,0%	100,0%	100,0%	100,0%	100,0%

* keine Starkbelasteten > 55dB vorhanden

Gesamtlärmbetrachtung Innsbruck

1. Prinzip und Limitationen

Unter Gesamtlärmbetrachtung versteht man die gemeinsame Darstellung von Schallimmissionen aus unterschiedlichen Schallquellen. Dem Grunde nach ist die hier abgebildete Gesamtlärmbetrachtung eine Verkehrslärmbetrachtung, eben aus unterschiedlichen Verkehrslärmquellen. Andere nichtverkehrsinduzierte Schallquellen sind dabei nicht berücksichtigt. Weiters nicht berücksichtigt sind bodengebundene Schallquellen des Flughafens Innsbruck, wie das Betreiben der Triebwerke im Stand und auf dem Weg zum Start, Betrieb von Hilfseinrichtungen oder Triebwerksprobeläufe. Ebenfalls nicht berücksichtigt ist beim Schienenverkehrslärm die Geräuschentwicklung des Verschubbetriebes am Bahnhof Innsbruck, hier werden ausschließlich die Durchfahrten berücksichtigt. Für den Verschubbetrieb fehlen schlicht die Modellierungsdaten. Im Sinne der großräumigen Betrachtung und der konkreten Betroffenheiten sind beim Verschubbetrieb des Bahnhofs keine bedeutenden Abweichungen zu erwarten.

2. Zusammenführen von Schallbelastungen

Bei einer Gesamtlärmbetrachtung kommen unterschiedliche Methoden in Frage. Eine Möglichkeit bildet die reine pegelenergetische Addition der Schallimmissionen der einzelnen Verkehrsträger. Diese Addition bringt zumindest theoretisch denselben Wert zu Tage, der bei demselben Emissionsverhalten aller Quellen und denselben meteorologischen Bedingungen eine Messung an diesem Punkt erbringen würde. Ein weiterer Ansatz der Gesamtlärmdarstellung ist der effektbezogene. Es ist ausreichend bewiesen, dass unterschiedliche Lärmquellen bei gleicher Einwirkung unterschiedlich starke Effekte hervorrufen, wie dies z. B. auch die EU konstatiert (*Position paper on dose response relationships between transportation noise and annoyance,* Office for Official Publications of the European Communities, Luxembourg 2002). Dem Index für die allgemeine Lärmbelastung L_{den}, liegt die (subjektive) Belästigungsreaktion Betroffener als Effekt zu Grunde. Ein Gesamtlärmmodell berücksichtigt nun, wie sich das Ausmaß der Belästigung im Einflussbereich einzelner konkreter Quellen abbildet und bringt dann bei Kombination mehrerer Quellen dasselbe Belästigungsäquivalent zu Tage. Dies kann dann wieder in Form eines Pegels zum Ausdruck gebracht werden, wobei hier eine einheitliche Bezeichnung noch nicht etabliert ist. So wird dieser wirkungsbezogene Gesamtlärmpegel im Good practice guide der EEA (European Environment Agency: *Good practice guide on noise exposure and potential health effects*, EEA Technical report Nr. 11/2010) als kumulierter Summenpegel bezeichnet, in VDI 3722 Blatt 2 (Verein Deutscher Ingenieure Richtlinie 3722 Blatt 2, *Wirkung von Verkehrsgeräuschen. Kenngrößen beim Einwirken mehrerer Quellenarten*, Berlin Mai 2013) als effektbezogener Substitutionspegel. Für die folgende Gesamtlärmbetrachtung wird die Methode der VDI 3722 Blatt 2 angewandt.

Unabhängig von der Methode, sei es die rein physikalische oder effektbezogene Summenbildung, stellen die ermittelten Pegel im Rechenraster oder an den Gebäudefassaden ausgedrückt als L_{den} oder L_{night} die Basis für weitergehende Überlegungen. In der folgenden Abbildung ist die Rasterlärmkarte für den Autobahnverkehr als L_{den} dargestellt. Da es sich bei den sonstigen Straßen (Landesstraßen, Gemeindestraßen) in Innsbruck um dieselbe Lärmquelle handelt, werden die Berechnungsergebnisse für den L_{den} energetisch addiert:

Abbildung 20: Rasterlärmkarte für den Autobahnverkehr als L_{den} in 4 m Höhe

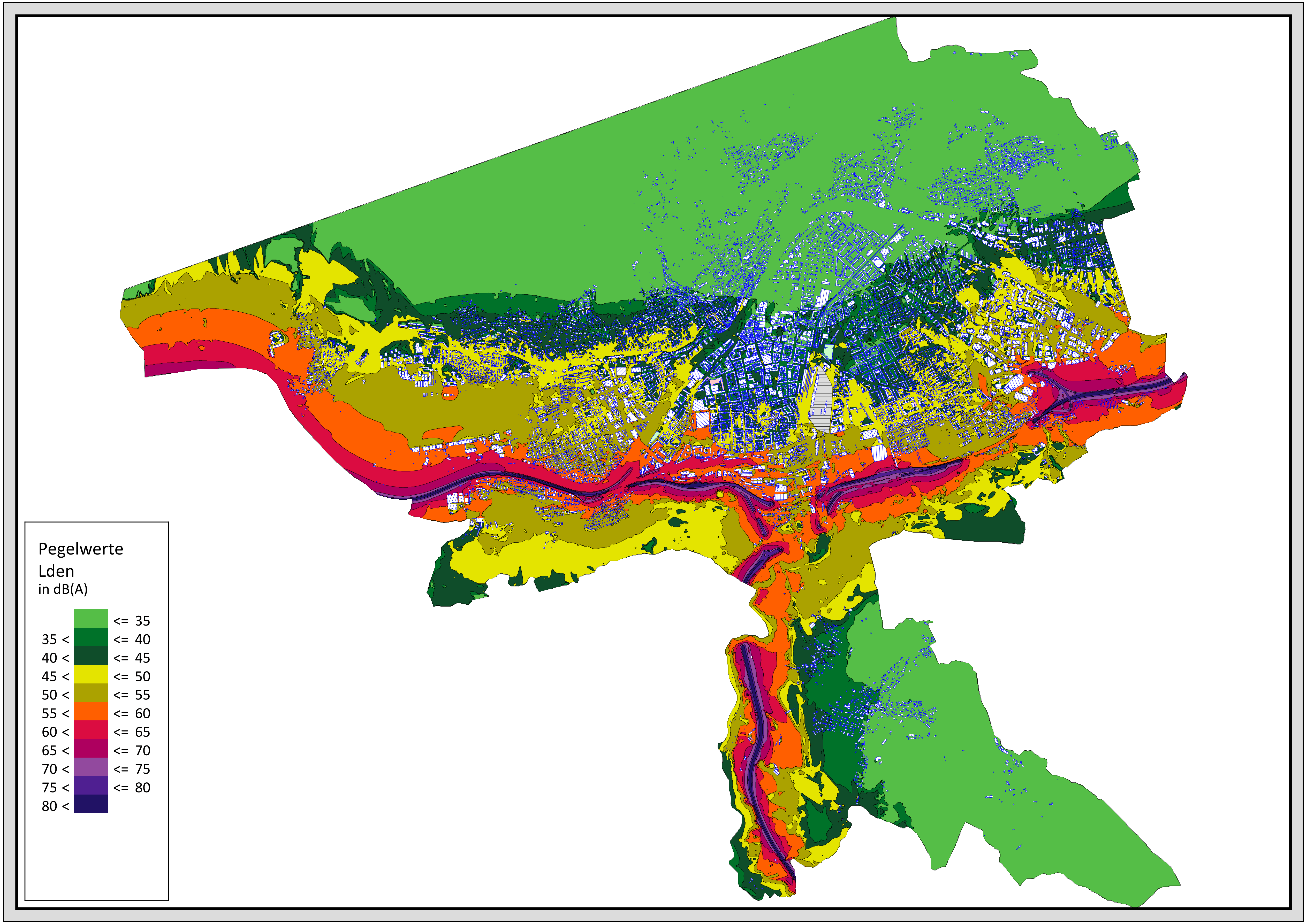

Abbildung 21: Rasterlärmkarte für den Straßenverkehr als L_{den} in 4 m Höhe

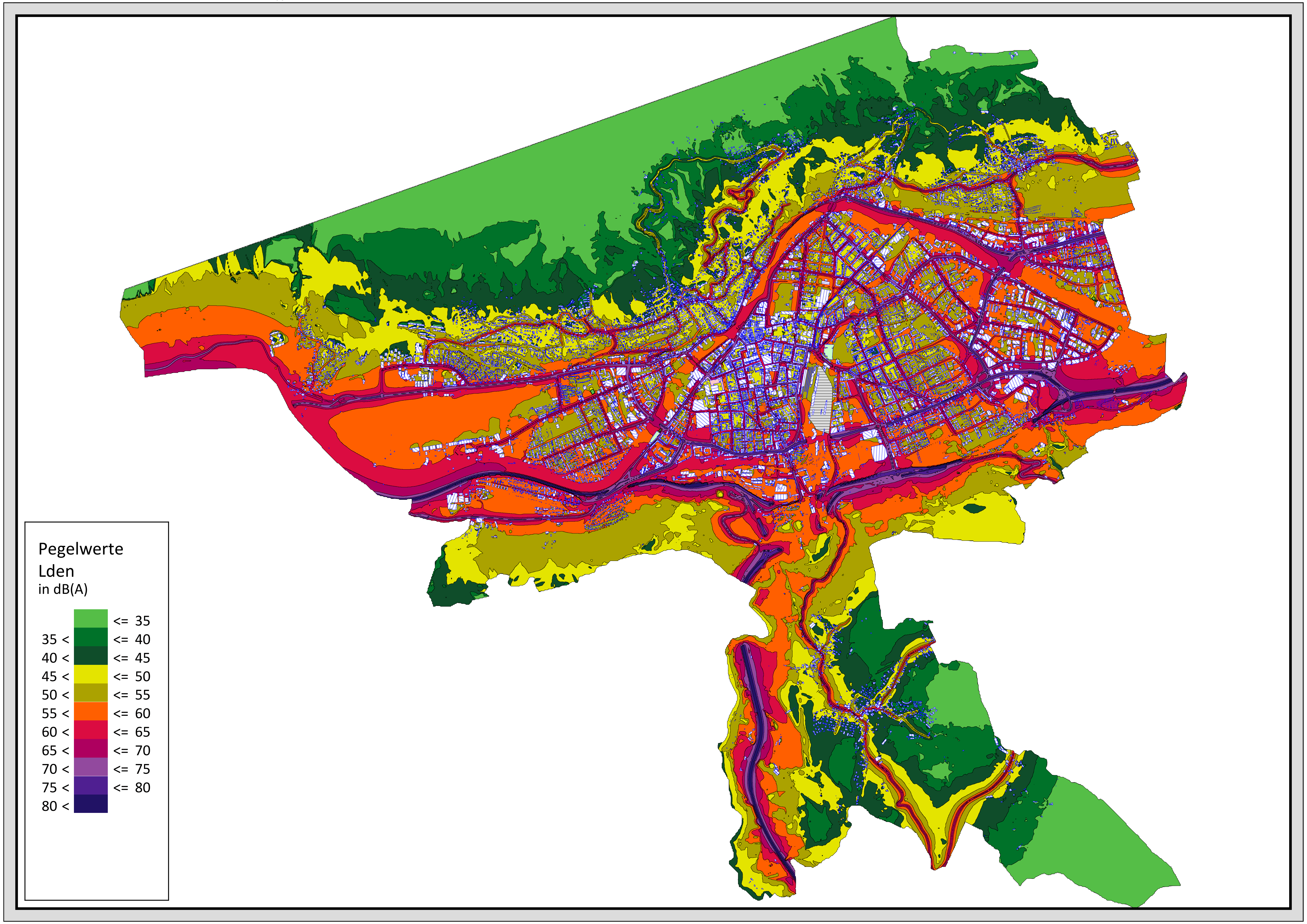

In obiger Abbildung erkennt man, dass im Nahbereich der Autobahn die Linien gleichen Schallpegels (auch Isophonen im Sinne Kurven gleicher Lautstärke genannt) wie in der ursprünglichen Abbildung verlaufen. In Bereichen fern der Autobahn dominieren nun die anderen Straßen. Dort wo es zur gemeinsamen Einwirkung von Autobahnlärm und Lärm aus anderen Straßen kommt, wie dies bei der Anschlussstelle Kranebitten deutlich ersichtlich wird, kommt es zur Überlagerung der Einwirkungen, wodurch zum Beispiel das gesamte Flughafenareal größtenteils von der Pegelzone 50 – 55 dB in die Pegelzone 55 – 60 dB rückt.

Dem Straßenverkehr kommt in der effektbezogenen Gesamtlärmbetrachtung eine besondere Bedeutung zu. Dadurch, dass unterschiedliche Quellen bei selbem Pegel unterschiedliche Effektgrößen erzielen, ist es notwendig, eine Referenzquelle zu definieren, auf die das Belästigungsäquivalent bezogen wird. Aus der Historie der Lärmwirkungsforschung bezieht sich das Belästigungsäquivalent auf die Referenzquelle Straßenverkehr. Der effektbezogene Pegel entspricht damit für Straßenverkehrslärm dem Lärmindex für die allgemeine Lärmbelastung L_{den}.

2. Wirkungsbezogene Darstellung

Da beim Straßenverkehr mit der obigen Abbildung die wirkungsbezogene Datendarstellung bereits gegeben ist, bedarf es bei den beiden anderen untersuchten Schallquellen Flug und Schiene einer Umrechnung auf einen effektbezogenen Substitutionspegel. Die nachstehenden Abbildungen zeigen den Vergleich der Lärmbelastung ausgedrückt als Lärmindex für die allgemeine Lärmbelastung L_{den} und als effektbezogener Substitutionspegel $L_{A,ES}$ für Flug- und Schienenverkehr:

Abbildung 22: Rasterlärmkarte für den Flugverkehr als L_{den} in 4 m Höhe

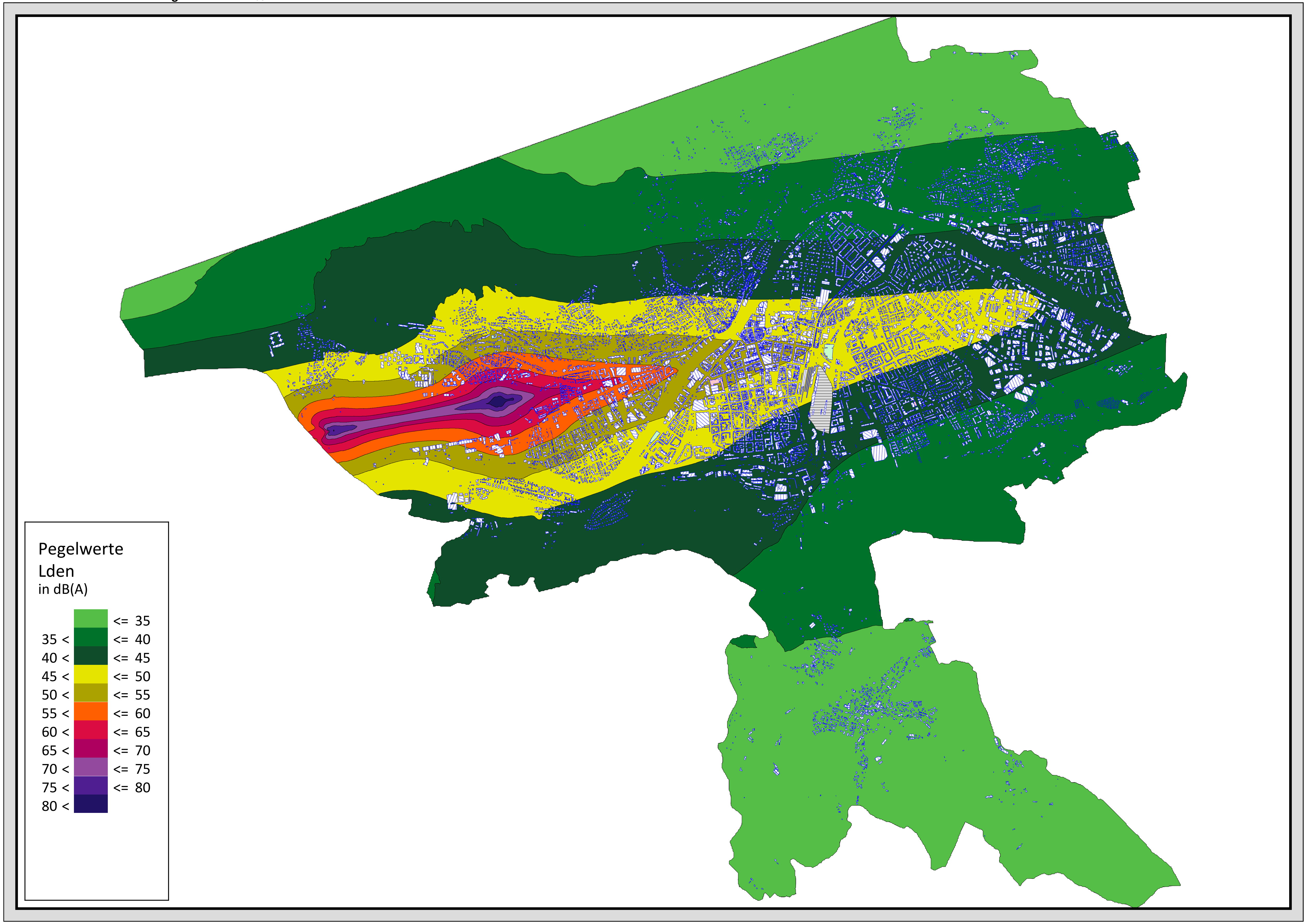

Abbildung 23: Rasterlärmkarte für den Flugverkehr als $L_{A,ES}$ in 4 m Höhe

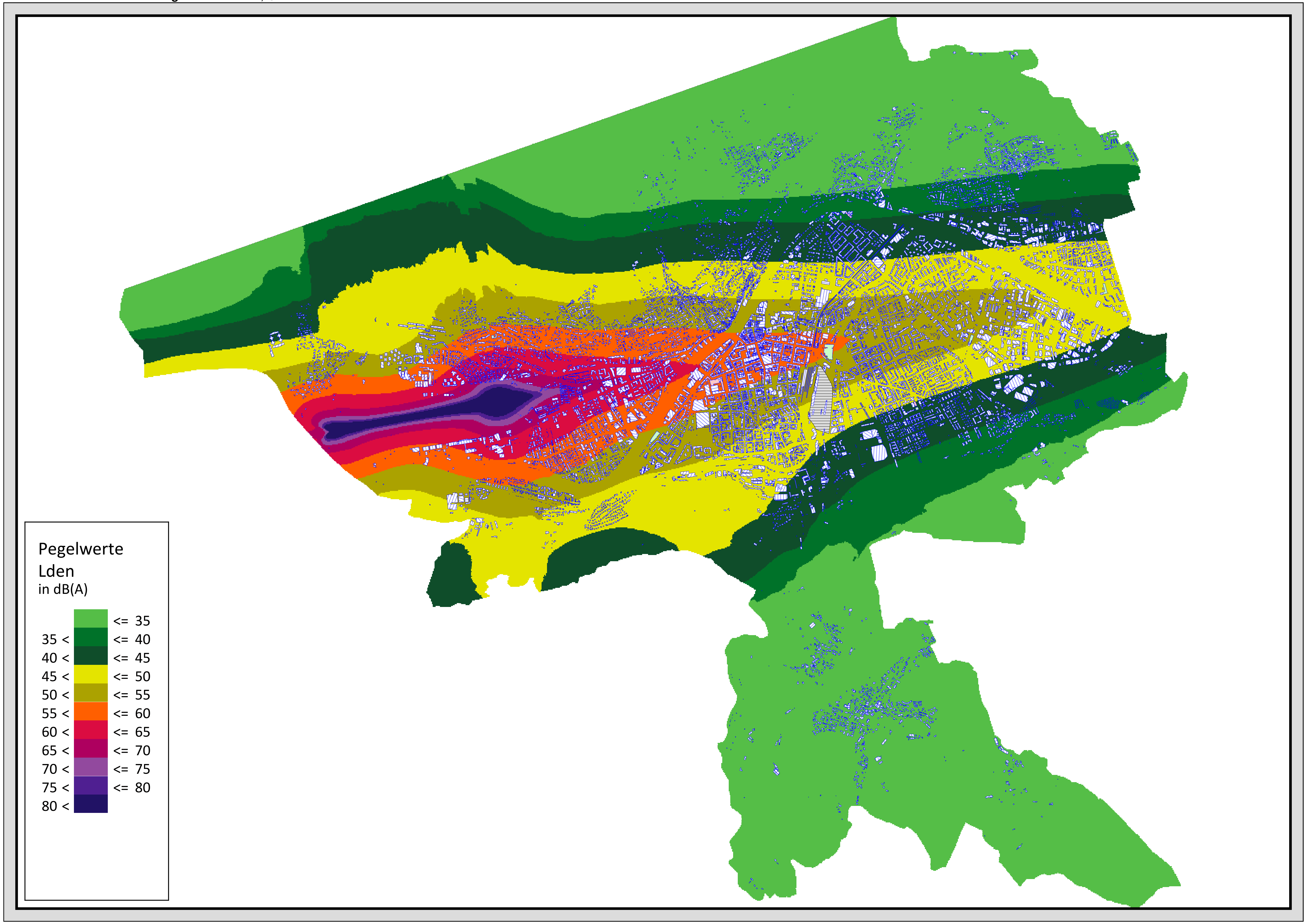

Abbildung 24: Rasterlärmkarte für den Schienenverkehr als L_{den} in 4 m Höhe

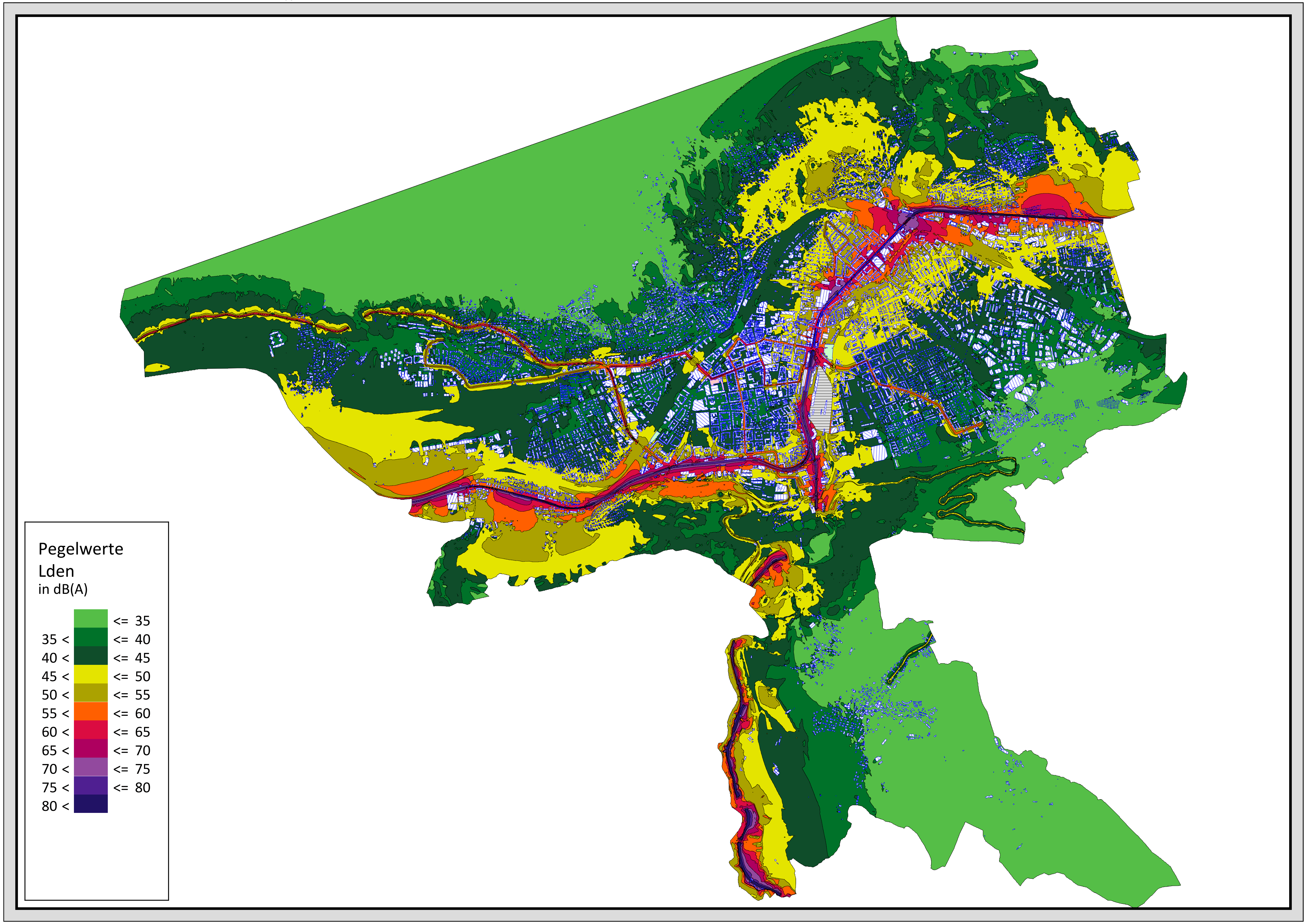

Abbildung 25: Rasterlärmkarte für den Schienenverkehr als $L_{A,ES}$ in 4 m Höhe

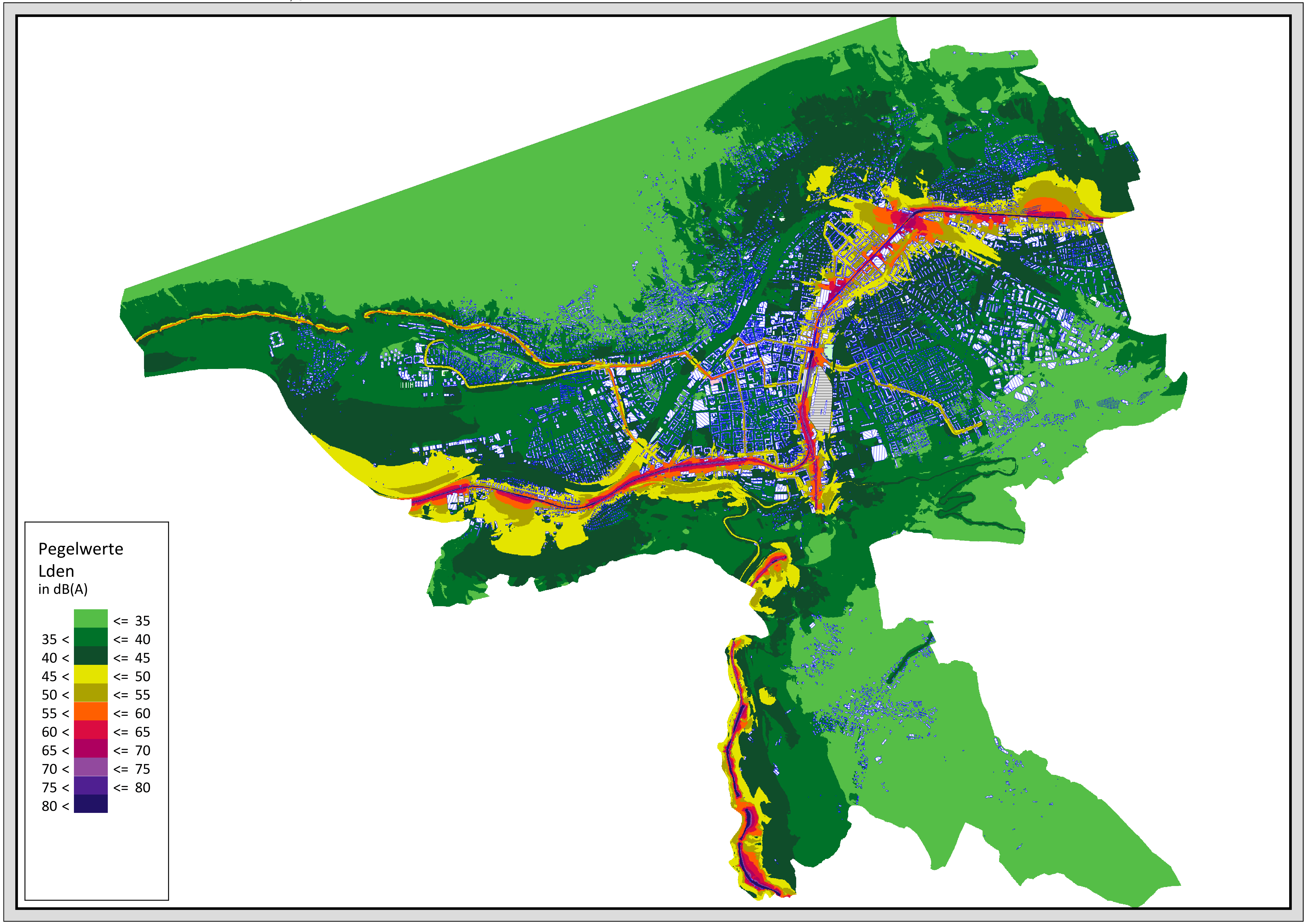

Bei Betrachtung dieser Rasterlärmkarten nach Quellen zeigt sich, dass die Belastungszonen beim Fluglärm deutlich ausgeweitet werden und als effektbezogener Substitutionspegel die Stadtteile Innsbruck, aber auch Reichenau und Pradl deutlicher betroffen werden. Im Gegensatz dazu werden die Belastungszonen ausgedrückt als effektbezogener Substitutionspegel beim Schienenverkehr deutlich kleiner, die Dominanz in einigen Bereichen geht daher deutlich zurück.

Aus den drei Einzelkarten für Straßen-, Flug- und Schienenverkehr wird durch Pegeladdition die Gesamtlärmbelastung nach VDI 3722 Blatt 2 wie in nachstehender Abbildung wiedergegeben ermittelt:

Abbildung 26: Rasterlärmkarte für den Gesamtlärm als effektbezogener Substitutionspegel $L_{A,ES}$ in 4 m Höhe

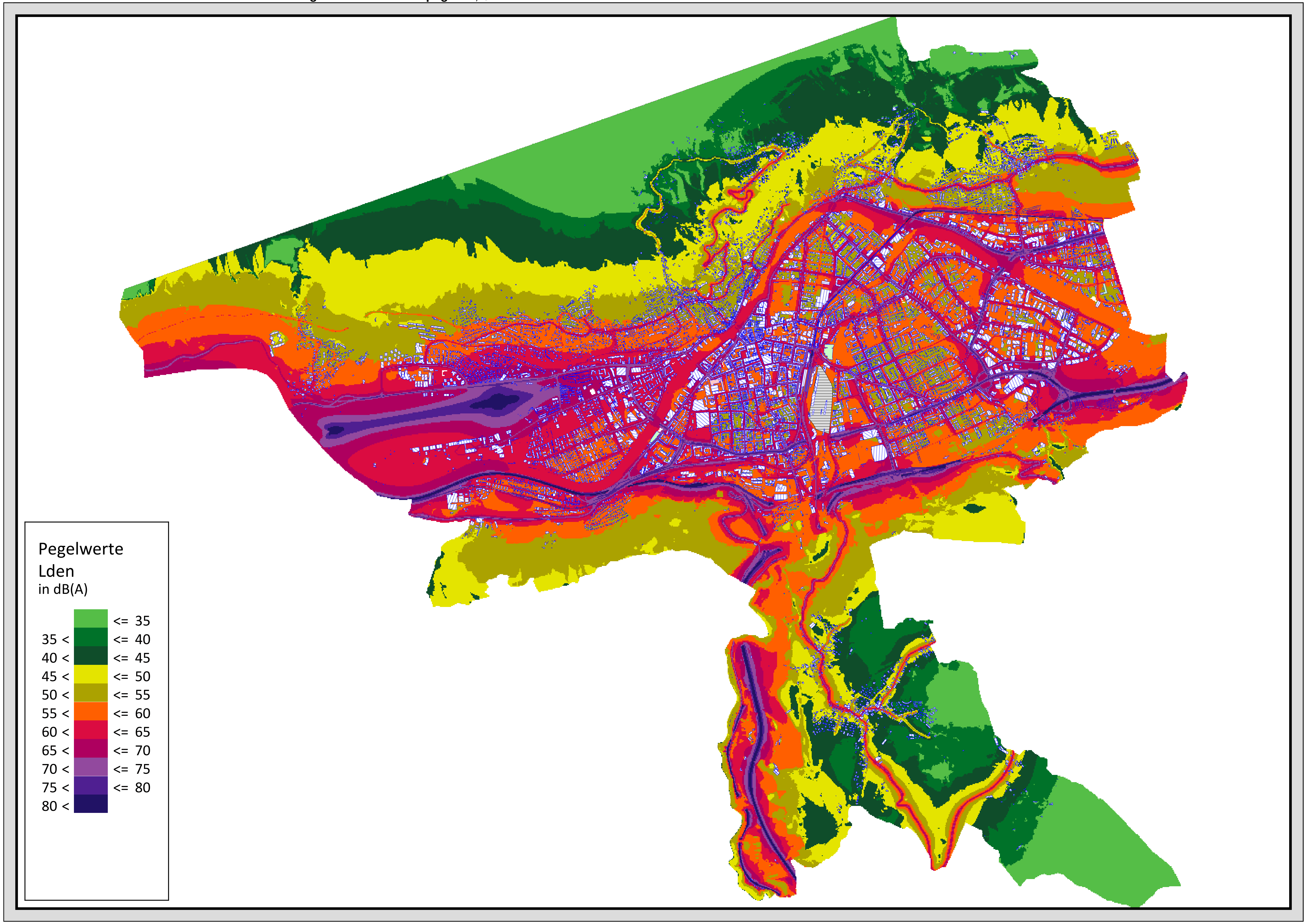

3. Hotspots

Hotspotanalysen dienen primär dazu, eine Priorisierungsmöglichkeit für Maßnahmen zu visualisieren. Dabei wird nicht ausschließlich die flächenhafte Lärmbelastung herangezogen, sondern auch die Betroffenheit in Form der Anzahl der jeweiligen Anwohnerinnen und Anwohner mit den Einwirkungen verknüpft. Im vorliegenden Fall bedeutet dies, dass für jede Quelle an jedem Gebäude die nach Exposition-Wirkung-Beziehungen ermittelte Wahrscheinlichkeit einer Belästigung berechnet wird. Diese Wahrscheinlichkeit wird aufsummiert und mit der Anzahl der Personen im Gebäude, welche dann gleichmäßig auf die Fassadenelemente verteilt werden, zu einer Beeinträchtigungskenngröße aggregiert. Im darauffolgenden Schritt werden über ein vorgegebenes Raster - hier aus Darstellungsgründen gewählt mit einer Seitenlänge von 200 m - diese Belästigungskenngrößen aufsummiert. Daraus ergeben sich Bereiche, in denen vergleichsweise viele Menschen vergleichsweise hohen Gesamtpegeln ausgesetzt sind. Die Besonderheit der nachstehenden Abbildung liegt darin, dass diese Belastungen sich auf die Gesamteinwirkungen der unterschiedlichen Lärmquellen beziehen und so die Verteilungssituation deutlich wird:

Abbildung 27: Hotsptanalyse in 200 m Rasterzellen auf Basis der Lärmkennziffer

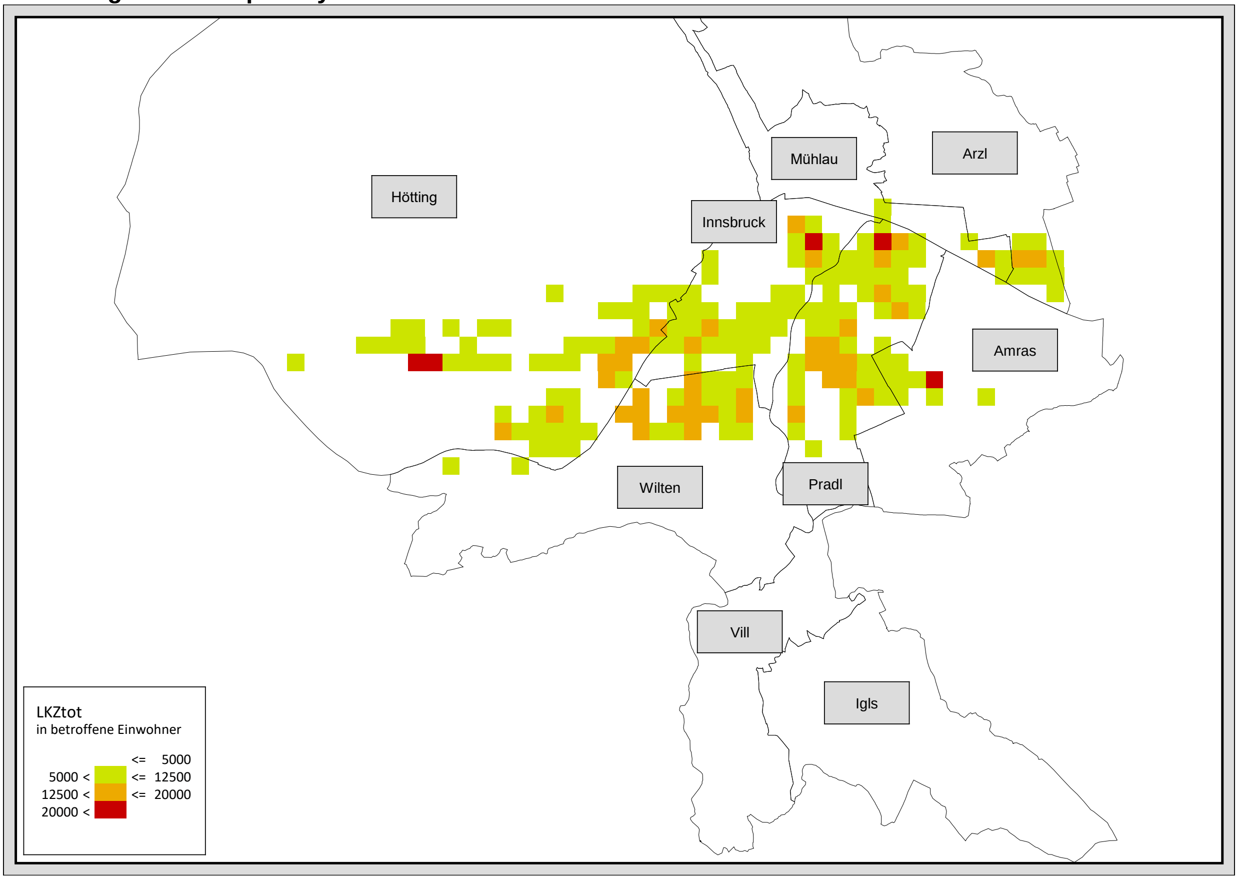

Die oben gezeigte Darstellung ist eine von vielen Möglichkeiten Hotspotanalysen durchzuführen. Je nach angestrebtem Zweck steht der Stadt Innsbruck ein Instrumentarium zur Verfügung, gröbere und feinere Analysen durchzuführen und die Gesamtlärmbelastung als zusätzliche Informationsquelle für Maßnahmenplanung zu nützen.

In der Darstellungsart in obiger Abbildung erscheinen 4 Hotspots mit einer Lärmkennziffer für die Summenlärmbelastung über 20.000. In der Katastralgemeinde Innsbruck scheint dabei der Bereich

um den Haydnplatz auf. Hier ist nicht die Lärmbelastung für sich besonders relevant, sondern die Dichte der Bewohnerinnen und Bewohner am Haydnplatz. Die hohe Anzahl der Betroffenen lässt diesen Bereich als Hotspot erscheinen.

In Pradl kommt bereits ein kumulierender Effekt durch Überlagerung von Schienen- und Straßenverkehrslärm, nämlich der Westbahn und der B 171, im Bereich der General-Eccher-Straße in Kombination mit der hohen Anzahl der Hauptwohnsitzgemeldeten zu tragen.

In Amras ist ebenfalls ein Hotspot zu beobachten dieser entsteht im Bereich der Dr.-Ferdinand-Kogler-Straße aufgrund der Belastungen durch den Erschließungsverkehr des Einkaufszentrums und der Autobahnzubringer wie auch durch die mehrseitigen Belastungen durch das Gemeindestraßennetz. Diese Hotspotanalyse zeigt sehr deutlich auf, welche Bedeutung der baulichen Gestaltung von Gebäuden in lärmbelasteten Bereich zukommt.

Der vierte Hotspot ist im Stadtteil Hötting zu verorten. Er ist in seinem Ausmaß doppelt so groß wie die drei vorhin genannten und entsteht entlang der Kranebitter Allee durch Kumulierung der verschiedenen Geräuschquellen, hier besonders der B 171 und der Schallimmissionen des Flugverkehrs. Die diesbezügliche Analyse zeigt auch, dass sich im Hinblick auf das Beschwerdeverhalten und den Auffälligkeitsgrad durch diese Analysemethode Erklärungsbeiträge liefern lassen.

4. Ruhige Gebiete

Ruhigen Gebieten kommt bereits aus dem Titel der Umgebungslärmrichtlinie eine besondere Bedeutung zu. Der Erhalt ruhiger Gebiete auch im Ballungsraum wird als Ziel dieser Richtlinie genannt. Nun ist es bei Vorliegen unterschiedlicher strategischer Lärmkarten aus verschiedenen Quellen so gut wie unmöglich, ruhige Gebiete im Überblick zu erkennen und damit die potenziell schützenswerten Bereiche ausfindig zu machen. Aber nicht nur die rechtlichen Rahmenbedingungen heben die Bedeutung ruhiger Gebiete hervor, auch die Ergebnisse der Interviewbefragungen sind hier eindeutig.

Für die nachstehende Abbildung wurde ein Schwellenwert für den $L_{A,ES}$ von 55 dB gewählt. Im Sinne der Clusterauswahlkriterien bezeichnet dieser Wert für den L_{den} durch die einzelnen Verkehrsträger die Grenze zur hohen Belastung. Alle Werte darunter sind damit mittel oder gering belastet. Zum Finden dieser wenig belasteter Bereiche ist die Auswahl jedenfalls geeignet:

Abbildung 28: Ausweisung „ruhiger Gebiete“ mit einem effektbezogener Substitutionspegel $L_{A,ES}$ in 4 m Höhe kleiner 55 dB

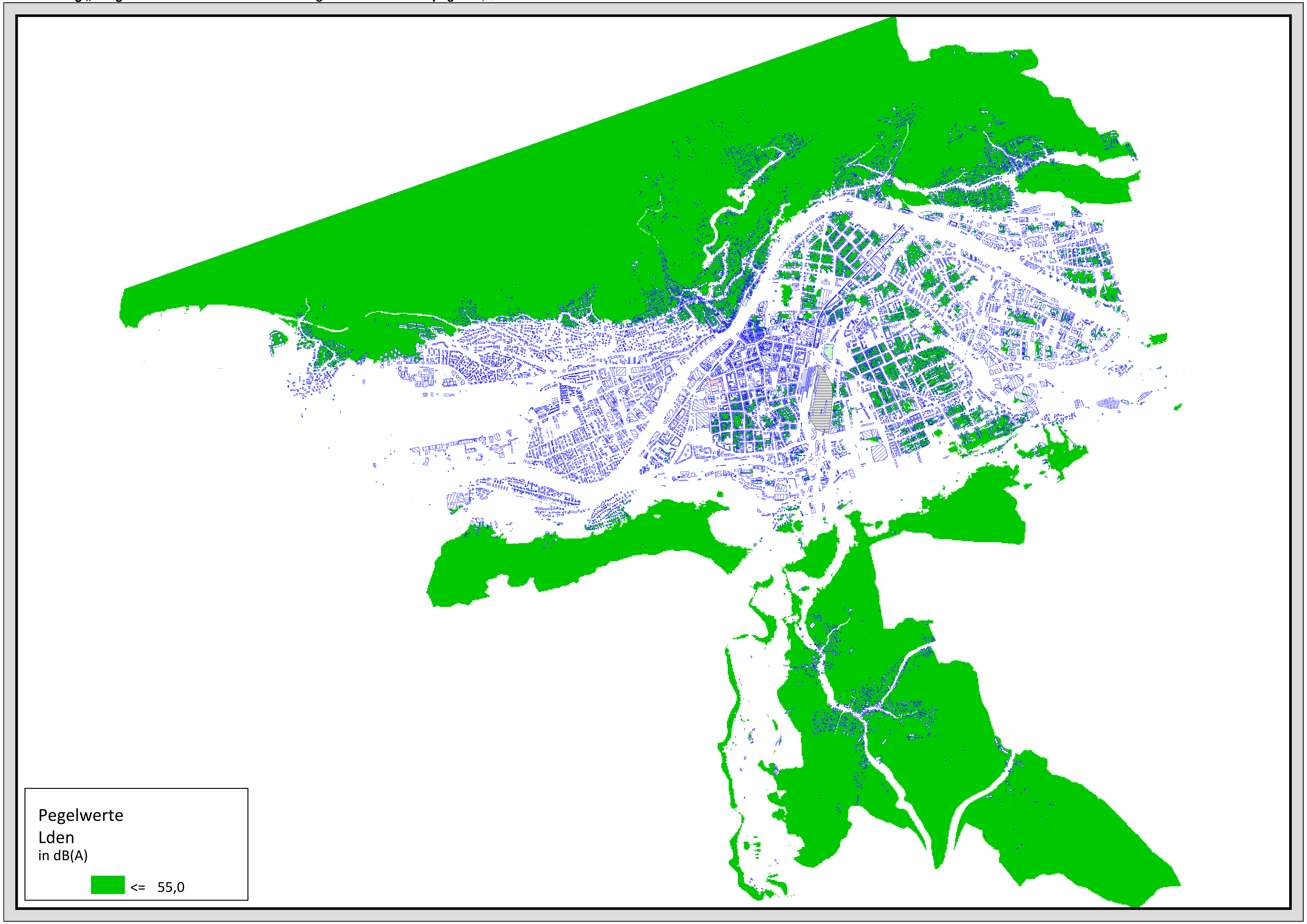

Die Summenlärmkarte für den effektbezogenen Substitutionspegel in der obenstehenden Abbildung zeigt im innerstädtischen Bereich nur wenige Gebiete, welche man als einigermaßen ruhig bezeichnen kann. Darunter fallen der Hofgarten und der Rapoldipark. Diesen zentralen innerstädtischen Naherholungszonen kommt eine hohe Bedeutung nicht nur als nutzbare Grünfläche sondern auch als allgemein zugängliche ruhige Zone im Sinne der Belastung von Verkehrslärm zu.

Viele weitere ruhige Bereiche sind kleinräumig zu finden. Dabei fällt die klassische Blockrandbebauung der Stadtteile Innsbruck (hier primär in Saggen) und Pradl auf. Diese beruhigten Lärmoasen haben für die Belästigungsreaktion eine hohe Bedeutung, stellen sie doch auch die Möglichkeit für Lüftungsmaßnahmen an ruhigen Fassaden her. In obiger Darstellung wird aber auch deutlich, dass im Westen Innsbrucks durch die Fluglärmeinwirkung keine lärmberuhigten Innenhofsituationen mehr bestehen.

Dem Erhalt dieser sehr kleinräumigen „ruhigen Gebiete“ wird aber eine besondere Bedeutung zukommen, dies umso mehr, als die gegenwärtigen Lösungen zur Gebäudekonditionierung in Form von Klimaanlagen und Wärmepumpen das Potential aufweisen, diese ruhigen Zonen mit zumeist tieffrequenten oder tonalen Dauergeräuschen zu gefährden.

Aus diesen Überlegungen lassen sich in Kombination mit den Befragungsergebnissen folgenden Handlungsempfehlungen ableiten:

- Erhalt der innerstädtischen Parks nicht nur als Grün-, sondern auch als Verkehrsruhezonen.
- Erhalt der ruhigen Innenhofbereiche besonders in Randbebauungssituationen durch vorsorglichen Umgang in der Zulassung maschineller Anlagen im Freien wie Klimaanlagen und Wärmepumpen.

Erfreulich aus dem Blickwinkel der Betrachtung ruhiger Gebiete sind die noch im Stadtgebiet Innsbruck vorhandenen, aber außerhalb der Kernbebauung liegenden vielen ruhigen Gebiete die unmittelbar und schnell erreichbar sind und die damit einen wesentlichen Beitrag für die temporäre Entlastung der Innsbruckerinnen und Innsbrucker leisten.

DURCHFÜHRUNGSPARAMETER DER BEFRAGUNGEN

Setting und Untersuchungsumfang der Befragungen

N: 1000 Personen im Stadtgebiet von Innsbruck

Art: Repräsentative Vorortbefragung durch geschulte Interviewerinnen und Interviewer

Fragebogen: geschlossenen Fragen auf Basis eines standardisierten Fragebogens

Stichprobe: Geschichtetes Adress-Cluster-Sample auf Basis der Daten des Zentralen Melderegisters nach Lärmkorridoren (Belastungsgruppen) unter Einbeziehung der einzelnen Verkehrsträger, der demographischen Parameter und nach Katastralgemeinden (Stadtteilen)

Lärmwertzuordnung: Verschneidung aller befragten Adresspunkte mit punktgenauen Fassadenlärmpegeln für den Tag- und den Nachtzeitraum aus dem entwickelten Gesamtlärmmodell (Bezugsjahr 2015) für sämtliche Verkehrsträger

Keine Imputations- / Complete Case-Verfahren

Durchgängige Berücksichtigung der gängigen Qualitäts- und Datenschutzstandards

Die Verschneidung der verschiedenen punktgenauen Fassadenpegel für den Tag- und den Nachtzeitraum mit den Ergebnissen der Befragungen lässt Querauswertungen und Rückschlüsse in alle gewünschten Richtungen zu. Durch eine breit gefächerte Schwerpunktsetzung in der Entwicklung des Fragebogens können zusätzliche Auswertungen jederzeit auch zu einem späteren Zeitpunkt nachgeholt werden. Spezielle Dosis-Wirkungs-Kurven, Sonderauswertungen für spezifische Belastungen, longitudinale oder qualitative Folgestudien, GIS-Modellierungen etc. werden ermöglicht.

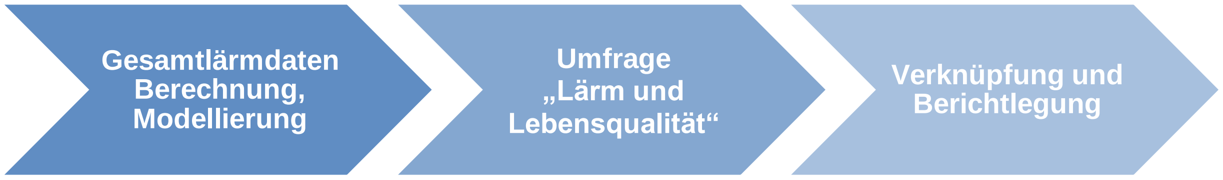

Der Fragebogen

1. Zusammenstellung

Quantitative Datengrundlagen zur Lärmsensibilität, sowie den Belästigungsreaktionen der Bevölkerung Innsbrucks sind bis dato nur in Ansätzen verfügbar und nicht in ausreichendem Umfang vorhanden. Über den entwickelten, zum Teil auf validierten und erprobten Items und Skalen aufgebauten Fragebogen, der sich am aktuellen Stand des Wissens orientiert, können diese Daten hergestellt werden. Das Hauptaugenmerk wurde auf die Wahrnehmung von und Betroffenheit durch Verkehrslärm und die damit einhergehenden Belästigungsreaktionen der Innsbrucker Bevölkerung gelegt.

Die Fragen zur Ausgesetztheit und Belästigung durch Verkehrslärm, sowie die entsprechenden Skalen (größtenteils 11-er Skalen) orientieren sich an den Empfehlungen der ICBEN (International Commission on Biological Effects of Noise), des EU-SILC 2015 und des Mikrozensus „Umweltbedingungen“. Berücksichtigt wurden weiters bereits validierte Items aus vergleichbaren Studien (NORAH, LEF-K Zimmer & Ellermeier zur Lärmempfindlichkeit, 5-Item-Skala zu Schlafproblemen, Vorerhebungen im Tiroler Raum etc.). Die soziodemographischen Fragen konnten zum Großteil standardisiert übernommen werden.

Zu einem erheblichen Teil wurde der Fragebogen jedoch eigens entwickelt und konnte daher nur eingeschränkt auf bestehenden Modulen aufsetzen.

Nach der Konsolidierung und Akkordierung des Fragebogens wurden sowohl statistische Reliabilitätstests, als auch eine Reihe von Testinterviews zur Validierung desselben durchgeführt.

2. Aufbau und Inhaltliche Schwerpunkte

- **Soziodemographie** (Alter, Geschlecht, Berufsstand, Bildungsniveau etc.)
- **Wohnsituation** (Wohnsitzfrage, Art des Hauses, Haushaltsgröße, Stockwerk, Lärmschutzfenster, Barrieren, Innenhöfe etc.)
- **Lärmwahrnehmung** (Ausgesetztheit, Wahrnehmung, Wind- und Wettereinflüsse)
- **Lebensbedingungen und Lebensqualität** (Güte Wohnbedingungen, Verkehrsverbindungen, Sicherheit, Landschaft und Natur, Nachbarschaftsbeziehungen, Bedingungen für Kinder, Verbundenheit zur Stadt, Zufriedenheit mit der Wohnsituation)
- **Subjektive Einschätzung der eigenen Lebensqualität**
- **Subjektive Einschätzung der eigenen Lärmempfindlichkeit**
- **Subjektive Einschätzung der eigenen Gesundheit**
- **Belästigung / Störung durch Lärm**
- **Belästigung / Störung durch Lärm im Schlaf**
- **Lärmcoping** (geschlossene Fenster, veränderte Wohnraumnutzung, Ärger, Hilflosigkeit, Gespräche mit Nachbarn, Gespräche mit Behörden, Veränderung der Lärmbelastung in den letzten Jahren insgesamt etc.)
- **Mobilität** (Nutzung öffentl. Verkehrsmittel, Auto, Bahn, Flugzeuge, Rad, Fußwege, Zufriedenheit mit öffentl. Verkehrsmitteln, Pendlerfrage)

- **Umgang mit Lärm** (eigener Beitrag zum Gesamtlärm, Bekanntheit der Umgebungslärmkarten, Fremdeinschätzung Lärmstörung, Sinnhaftigkeit weiterer Maßnahmen zur Lärmreduktion, Unterschiede zu anderen österr. Städten etc.)

Alle befragten Personen unterschrieben eine Einverständniserklärung zur Teilnahme und Datenverarbeitung.

Die Stichprobe

Um die Gesamtanzahl der 1000 angestrebten Interviews zu erreichen, wurde ein Sampling ausgearbeitet, das sich an 3 Grundparametern gleichermaßen orientierte:

- Demographische Repräsentativität
- Repräsentative Abbildung der 9 Katastralgemeinden von Innsbruck
- Einteilung nach vorgegebenen Lärmkorridoren analog den Lärmbelastungen ausgehend von den Hauptverkehrsträgern Straße, Schiene und Flugverkehr

1. Demographische Repräsentativität

Die Parameter Alter und Geschlecht, sowie das Verhältnis der Anzahl der Gesamtbevölkerung zur Größe der Stichprobe (N) bilden die klassischen Repräsentativitätskriterien für größere Umfragen und kamen auch bei den Befragungen zur Gesamtlärmbetrachtung Innsbruck zum Einsatz, wobei sehr strenge Kriterien in Hinblick auf tolerable Abweichungen (Standardfehler) vorgegeben wurden.

1.1. Alter

Die Altersvorgaben, kategorisiert in 3 Altersgruppen, wurden im Zuge der Befragungen sehr genau abgebildet und weichen in ihrer größten Ausprägung um weniger als 1% von der Realverteilung in der Innsbrucker Gesamtbevölkerung ab:

Tabelle 18: Soll-Ist-Vergleich der Altersgruppen Stichprobe ↔ Interviews

	Vorgegebene Stichprobe in Prozent (entspricht der Realverteilung)	Häufigkeit geführte Interviews	Gültige Prozente Interviews	Abweichung in Prozent
18 bis 40 Jahre	41,8	440	42,7	**0,9**
41 bis 60 Jahre	31	316	30,6	**-0,4**
über 60 Jahre	27,2	275	26,7	**-0,5**
Gesamt	100,0	1031	100,0	**0,0**

1.2. Geschlechterverteilung

Aus dem Soll-Ist-Vergleich Stichprobe ↔ durchgeführte Interviews ergibt sich für die Geschlechterverteilung eine Abweichung von knapp 1,2% zugunsten der weiblichen Befragten:

Tabelle 19: Soll-Ist-Vergleich der Geschlechterverteilung Stichprobe ↔ Interviews

	Vorgegebene Stichprobe in Prozent (entspricht der Realverteilung)	Häufigkeit geführte Interviews	Gültige Prozente Interviews	Abweichung in Prozent
weiblich	51,3	541	52,5	**1,2**
männlich	48,7	490	47,5	**-1,2**
Gesamt	100,0	1031	100,0	**0,0**

Diese Abweichung resultiert aus der Gesamtzahl von 1031 vollständig durchgeführten Befragungen. In der Datenbank wurde eine eigene Variable gebildet, welche insgesamt 17 Fragebögen unberücksichtigt lässt und damit unter Einhaltung sämtlicher anderen Stichprobenkriterien den Standardfehler im Geschlechterverhältnis auf unter 0,4% drückt. Auch bezogen auf die Geschlechterverteilung konnte somit eine sehr genaue Abbildung der Gesamtbevölkerung im Zuge der Befragungsdurchführung erreicht werden.

1.3. Stichprobengröße

Die Gesamtanzahl von 1031 vollständig durchgeführten Interviews (N) entspricht einem Anteil von knapp einem Prozent der Hauptwohnsitzbevölkerung Innsbrucks im befragbaren Alter von über 18 Jahren. Im Zentralen Melderegister waren zum Stichtag 01.01.2016 für Innsbruck 69.888 Wohnungen gemeldet. Da unter Berücksichtigung der anderen Stichprobenkriterien nur ein Interview pro Haushalt als sinnvoll vorgegeben wurde, konnte damit jeder 68. Haushalt erfolgreich befragt werden.

Da als weitere Vorgabe nur jene Personen befragt werden durften, die seit mindestens einem vollen Jahr in ihrer Wohnung lebten und daher die Lärmsituation in ihrem Wohnumfeld ausreichend beurteilen konnten, stellten die Wanderungsbewegungen eine erhebliche Herausforderung für die Durchführung der Befragungen dar. 2016 wurden in Innsbruck-Stadt 12.152 Zuzüge, 10.943 Wegzüge und 12.105 Umzüge verzeichnet,[4] was einem Wanderungsvolumen von fast einem Viertel der Hauptwohnsitzbevölkerung entspricht. Wird dieses Viertel bei einer durchschnittlichen Haushaltsgröße von 1,92 Personen pro Haushalt (letzterhobener Stand 2014) auf die Gesamtzahl der befragbaren Haushalte übertragen, ist davon auszugehen, dass **jeder 46.** – aufgrund der Vorgaben überhaupt mögliche – **Haushalt in Innsbruck erfolgreich befragt** wurde.

Insgesamt kann somit festgehalten werden, dass eine aus demographischer Sicht hochrepräsentative Befragung durchgeführt wurde (siehe dazu auch die Ausführungen im Kapitel *Rücklaufquote*).

2. Repräsentative Abbildung der 9 Katastralgemeinden

Die durchzuführenden Befragungen sollten sämtliche 9 Katastralgemeinden umfassen und somit verteilt auf das gesamt Stadtgebiet von Innsbruck stattfinden.

Tabelle 20: Schlüssel Katastralgemeinden Innsbruck (nach Einwohneranzahl geordnet)

Katastralgemeinde	Einwohner	% anteilig	absolut	Anpassungsfaktor	zu befragen
Hötting	34.416	26,13%	261	75%	**196**
Pradl	32.842	24,94%	249	75%	**187**
Innsbruck	21.464	16,30%	163	125%	**204**
Wilten	17.853	13,56%	136	110%	**149**
Arzl	10.829	8,22%	82	100%	**82**
Amras	5.933	4,50%	45	150%	**68**
Mühlau	5.403	4,10%	41	150%	**62**
Igls	2.416	1,83%	18	175%	**32**
Vill	546	0,41%	4	500%	**21**
**Gesamt**	**131.702**	**100,00%**	**1000**		**1000**

[4] Quelle: Stadtmagistrat Innsbruck: Statistische Quartalsblätter Heft 1, 2017

Um eine gleichmäßigere und repräsentativere Verteilung zu gewährleisten, wurden einwohnerschwächere Katastralgemeinden, sowie das Stadtzentrum (Katastralgemeinde Innsbruck) „aufgewertet“ und die beiden einwohnerstärksten Katastralgemeinden Hötting und Pradl „abgewertet“.

Als Mindestanzahl für die Befragungen wurden 20 Interviews pro Katastralgemeinde festgelegt:

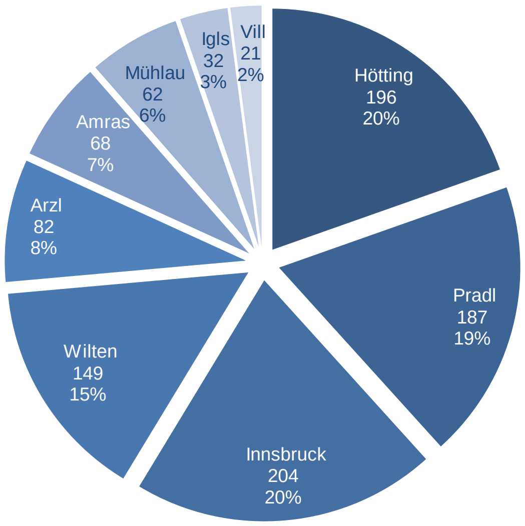

Abbildung 29: Angepasster Befragungsschlüssel Katastralgemeinden Innsbruck

Die untenstehende Tabelle umfasst die Gesamtanzahl der durchgeführten 1031 Interviews:

Tabelle 21: Soll-Ist-Vergleich der Katastralgemeinden Stichprobe ↔ Interviews

Katastralgemeinde (alphabetisch)	Häufigkeit vorgegebene gewichtete Stichprobe	% anteilig gewichtete Stichprobe	Häufigkeit geführte Interviews	% anteilig geführte Interviews	% anteilige Abweichung
Amras	68	6,8	60	5,8	**-1,0**
Arzl	82	8,2	102	9,9	**1,7**
Hötting	196	19,6	243	23,6	**4,0**
Igls	32	3,2	35	3,4	**0,2**
Innsbruck	204	20,4	176	17,1	**-3,3**
Mühlau	62	6,2	50	4,8	**-1,4**
Pradl	187	18,7	188	18,2	**-0,5**
Vill	21	2,1	21	2	**-0,1**
Wilten	149	14,9	156	15,1	**0,2**
Gesamt	1001	100	1031	100	**0,09**

3. Lärmkorridore

Zur Einteilung der Befragungen nach den Lärmbelastungen ausgehend von den Hauptverkehrsträgern Straße, Schiene und Flugverkehr wurden zunächst sämtliche Lärmdaten berechnet und fassadengenau modelliert (siehe dazu das Kapitel *Verwendete Lärmdaten*).

Anschließend wurden insgesamt 9 gewichtete Lärmkorridore erstellt:

Tabelle 22: Matrix Lärmkorridore

		Fluglärm Lden			Schienenlärm Lden			Straßenlärm Lden		
		Starkbelastete > 55dB Adresspunkte	Mittelbelastete 45-55dB Adresspunkte	Geringbelastete < 45dB Adresspunkte	Starkbelastete > 55dB Adresspunkte	Mittelbelastete 45-55dB Adresspunkte	Geringbelastete < 45dB Adresspunkte	Starkbelastete > 55dB Adresspunkte	Mittelbelastete 45-55dB Adresspunkte	Geringbelastete < 45dB Adresspunkte
**Fluglärm Lden**	Starkbelastete > 55dB	643	0	0	19	90	534	281	362	0
	Mittelbelastete 45-55dB	0	4012	0	367	1213	2432	2139	1768	105
	Geringbelastete < 45dB	0	0	5056	470	1769	2817	2353	2018	685
**Schienenlärm Lden**	Starkbelastete > 55dB	19	367	470	856	0	0	653	203	0
	Mittelbelastete 45-55dB	90	1213	1769	0	3072	0	1898	1118	56
	Geringbelastete < 45dB	534	2432	2817	0	0	5783	2222	2827	734
**Straßenlärm Lden**	Starkbelastete > 55dB	281	2139	2353	653	1898	2222	4773	0	0
	Mittelbelastete 45-55dB	362	1768	2018	203	1118	2827	0	4148	0
	Geringbelastete < 45dB	0	105	685	0	56	734	0	0	790

In jedem der 9 Lärmkorridore waren jeweils zumindest 111 +1 vollständige Befragungen durchzuführen, was auch erfolgreich erreicht werden konnte.

Rücklaufquote

Unter der Rücklaufquote oder Response-Rate wird das Verhältnis von erfolgreichen / vollständigen Befragungen zu versuchten Interviews im Falle des Antreffens einer möglichen Befragungsperson verstanden.

Für die Befragungen zur Gesamtlärmbetrachtung Innsbruck wurden Imputations- bzw. Complete-Case-Verfahren von vornherein ausgeschlossen und durch umfangreiche Vorarbeiten auf eine hohe Rücklaufquote gezielt hingearbeitet:

– Alle Interviewerinnen und Interviewer wurden vom Befragungsinstitut sorgfältig ausgewählt und von der Projektleitung zusätzlich geschult, sensibilisiert und mit den wesentlichen Informationen zu Lärmbefragungen vertraut gemacht.
– Die Befragungen wurden auf den Homepages des Landes Tirol und der Stadt Innsbruck, über eine allgemeine Presseaussendung, sowie mehrere Artikel in der *Tiroler Landeszeitung*, in *Innsbruck informiert*, der *Tiroler Tageszeitung*, den *regionews*, *orf.at*, *unsertirol24* u.ä. möglichst publik gemacht.
– Die Lebensrhythmen der Befragten wurden berücksichtigt und Interviews zu verschiedenen Tages(rand-)zeiten durchgeführt, um Berufstätige, Studentinnen und Studenten usw. aller Altersklassen zu erreichen.
– Die zeitliche Dauer der Interviews wurde dergestalt angepasst, dass ein sinnvoller Kompromiss zwischen dem größtmöglichen Informationsgehalt des Fragebogens und nicht allzu überlangen Interviews getroffen wurde, um inkonsistentes Antwortverhalten und Befragungsabbrüche möglichst zu vermeiden.

Offensichtlich bewohnte Adresspunkte an denen keine Personen anzutreffen waren, wurden grundsätzlich zwei Mal zu einem späteren Zeitpunkt erneut aufgesucht. Wurde bei insgesamt drei Versuchen niemand angetroffen, wurde der Adresspunkt gestrichen.

Wurde im Zuge der Befragungen den Interviewerinnen und Interviewern die Wohnungstür geöffnet, stellte sich der / die Interviewer(in) vor, wies sich aus, zeigte das vorbereitete Anschreiben der Projektleitung vor, erläuterte das Vorhaben und bat um ein Interview. Insgesamt wurde den Interviewerinnen und Interviewern im Zuge der Befragungen in 2157 Haushalten die Tür geöffnet.

Nachteilig auf die Rücklaufquote wirkten sich folgende Vorgaben aus:

– Zur Sicherstellung der demographischen Repräsentativität mussten die Interviewerinnen und Interviewer eine repräsentative Verteilung nach Alter und Geschlecht (siehe das Kapitel *Die Stichprobe*) gewährleisten, sodass bei Nichtvorhandensein von gesuchten Zielgruppen in einem Haushalt, ein nächster Haushalt kontaktiert wurde.
– Es konnten nur Personen befragt werden, die zumindest ein Jahr an der jeweiligen Adresse lebten und somit die Lärmsituation ausreichend beurteilen konnten. Die Wanderungsrate in Innsbruck ist aber besonders in den vergangenen Jahren sehr hoch (siehe zu den Wanderungsbewegungen auch die Ausführungen im Unterkapitel *1.3 Stichprobengröße*).
– In Haushalten mit Migrationshintergrund, nicht-deutscher Muttersprache und gleichzeitig niederer Bildungsschicht kann zumeist nur eine bescheidene Antwortquote erzielt werden. Dieser

Umstand führt zu einer allen Befragungen systemimmanenten Verzerrung bzw. einem nicht vermeidbaren Bias.

Insgesamt 25 Interviews wurden – zumeist wegen anstehender Termine, dringenden Telefonaten, von der Schule heimkommenden Kindern etc. – abgebrochen und mussten daher verworfen werden. In etwa jede zweite tatsächlich angetroffene und befragungsfähige Person wollte aus unterschiedlichen Motiven (Zeitmangel, mangelnde Deutschkenntnisse, kein Interesse, Termine etc.) nicht an den Befragungen zur Gesamtlärmbetrachtung Innsbruck teilnehmen.

Umgekehrt war knapp die Hälfte aller angetroffenen Personen spontan bereit, an den Befragungen teilzunehmen und eine **Gesamtrücklaufquote von insgesamt 47,8%** konnte erreicht werden.

Tabelle 23: Rücklauf Befragungen Gesamtlärmbetrachtung Innsbruck

Lärmkorridor	Interview durchgeführt	Nicht angetroffen	Kein Interesse	Interview abgebrochen	Summe Kontaktversuche	Summe angetroffene Kontakte	% Rücklauf angetroffene Kontakte
Flug stark	111	963	184	4	1262	299	37%
Flug mittel	111	491	118	2	722	231	48%
Flug gering	118	528	131	2	779	251	47%
Schiene stark	111	737	121	2	971	234	47%
Schiene mittel	114	512	96	3	725	213	54%
Schiene gering	118	843	126	2	1089	246	48%
Straße stark	118	743	138	1	1000	257	46%
Straße mittel	119	692	135	5	951	259	46%
Straße gering	111	375	52	4	542	167	66%
**Gesamt**	**1031**	**5884**	**1101**	**25**	**8041**	**2157**	**47,8%**

Dieser Wert gewährleistet eine sehr hohe **Repräsentativität der Ergebnisse**, minimiert systemimmanente Bias (Selbstselektion Stichprobenfehler, mangelnde Repräsentativität usw.) und ist für eine Vor-Ort-Befragung in einem urbanen Raum als **außergewöhnlich hoch** einzustufen, liegt diese Rücklaufquote doch um ein Mehrfaches über den Response-Rates aktueller vergleichbarer internationaler Studien.

Durchführung der Befragungen und Datenkontrolle

Mit der Durchführung der Befragungen wurde das Befragungsinstitut *IMAD Marktforschung und Datenanalysen* im Zuge eines Ausschreibungsverfahrens beauftragt.

Zwischen 16. Mai und 8. Juli 2017 wurden insgesamt 1031 vollständige Vor-Ort-Interviews von 23 geschulten Interviewerinnen und Interviewern im Stadtgebiet von Innsbruck durchgeführt. Die Interviews wurden von Montag bis Samstag zu verschiedenen Uhrzeiten in den Wohnungen der zu Befragenden geführt, um möglichst alle Bevölkerungsschichten, insbesonders auch die arbeitende Bevölkerung, ansprechen zu können. An Sonn- und Feiertagen fanden keine Befragungen statt.

Die Interviewerinnen und Interviewer suchten die vorgegebenen Adresspunkte direkt auf und führten neben eigens angefertigten Ausweisen und einem Anschreiben der Projektleitung auch laminierte Handzettel mit sich, auf welchen die unterschiedlichen im Fragebogen verwendeten Skalen ersichtlich waren, um sie den Probandinnen und Probanden zur besseren Übersichtlichkeit vorlegen zu können. Die Ausweise waren gut ersichtlich an der Kleidung angebracht, weiters sorgte die Verwendung von Taschen versehen mit einem großen Logo und Schriftzug der Stadt Innsbruck für eine gute Erkennbarkeit.

Die ausgefüllten Fragebögen wurden unter Einhaltung der gängigen Qualitätssicherungs- und Datenschutzauflagen händisch von den Mitarbeiterinnen und Mitarbeitern der *IMAD Marktforschung und Datenanalysen* digitalisiert, mit den Adress- und Lärmdaten verschnitten und für die weiterführende statistische Auswertung aufbereitet.

Eine ausführliche Eingabe- und Plausibilitätskontrolle der Datenbank, sowie der Originalfragebögen wurde von der Projektleitung vorgenommen:

- Univariate Plausibilitätskontrolle anhand der deskriptiven Statistik, sowie der graphisch ausgegebenen Häufigkeiten
- Kontrolle auf echte fehlende Werte in der Datenbank
- Kontrolle der Originalfragebögen auf Vollständigkeit, Auffälligkeiten und Stimmigkeit: Insbesonders die Unterschriften, die ausgefüllten Adressdaten, Zeitpunkte der Befragungen, Muster der einzelnen Interviewerinnen und Interviewer etc. wurden gesichtet
- Stichprobenhafte Gegenüberstellung der ausgefüllten Fragebögen und der digitalen Datenbank zur Prüfung auf Eingabe- und Übertragungsfehler
- Dateivergleich der Lärm- und Geodaten der Befragungsdatenbank und der ausgegebenen Ausgangsdaten mittels Excel-Spreadsheet inklusive vollständigem Einzelzellenvergleich

Durch die aufwendige Datenkontrolle konnte sichergestellt werden, dass sich die Ausgangsdatenbank für die Auswertungen auf höchstmöglichem Niveau befindet.

Datenschutz

In der Durchführung der Befragungen zur Gesamtlärmbetrachtung Innsbruck wurden personenbezogene Daten erhoben und verarbeitet, welche laut Datenschutzgesetz 2000 idgF zu keinem Zeitpunkt veröffentlicht oder Unbefugten zugänglich gemacht werden dürfen. Auch dürfen diese Daten in ihrer Auswertung einzelnen Probandinnen und Probanden oder Haushalten nicht zuordenbar sein, weshalb auf die Datenanonymisierung großes Augenmerk gelegt wurde. Des Weiteren ist es untersagt, geschützte personenbezogene Daten unbefugt zu einem anderen als dem zur jeweiligen rechtmäßigen Aufgabenerfüllung gehörenden Zweck zu verarbeiten, bekannt zu geben, zugänglich zu machen oder anderweitig zu nutzen.

Im Besonderen war die Sicherung der Daten vor Verlust, Diebstahl und unbefugter Einsichtnahme zu gewährleisten. Alle beteiligten Institutionen/Personen hatten die Einhaltung der Bestimmungen und Sicherheitsmaßnamen des §14 DSG 2000 idgF zu gewährleisten. Das Befragungsinstitut IMAD, das mit der Durchführung der Erhebungen beauftragt wurde, hatte eine DVR-Nummer nachzuweisen, sowie eine durchgängige Dokumentation über die Erhebung, die Sicherung, sowie einzelne Verwendungsschritte der erhobenen Daten zu Kontrollzwecken zu führen. Nach Übergabe der Datenbank an die Projektleitung wurden sämtliche Projektunterlagen und Aufzeichnungen (digital und analog), welche beim Befragungsinstitut verblieben waren, gelöscht bzw. vernichtet.

Im Zuge der Vorortbefragungen waren von allen befragten Personen Einverständniserklärungen zur Datenverarbeitung zu unterschreiben. Die Anonymisierung erfolgte daher zweistufig:

(1) Über die Nichtberücksichtigung der Namensdaten in der digitalen Datenbank: Die Ausgangsdatenbank für die Auswertungen enthält einzelnen Adresspunkten zuordenbare Adress- und Lärmdaten, jedoch keine Namen. Nach den Eingabe- und Plausibilitätskontrollen der Datenbank sowie der Originalfragebögen, wurden diese in Kisten verpackt und im Depot des AdTLR zur Aufbewahrung gemäß DSG 2000 idgF in einem unzugänglichen, versperrten Raum eingelagert.

Im Zuge der Auswertung und Berichtlegung war die digitale Datenbank nur 2 Personen (Dr. Schnaiter und Dipl.-HTL-Ing. Mag Lechner) zugänglich, die eigens dafür angefertigte Datenschutzprotokolle unterschrieben. Die digitale Datenbank wurde ausschließlich verschlüsselt auf internen Datenträgern gespeichert. Sämtliche Projektunterlagen, Aufzeichnungen, Dateien, Auswertungen und Datensätze (digital und analog) sind nach Beendigung des Auftrages zu vernichten.

(2) Über die nicht mehr einer Einzelperson / einem einzelnen Haushalt zuordenbare statistische Auswertung der Daten: Sämtliche Berichte und Präsentationen enthalten Auswertungen und Darstellungen gemäß den Lärmdatenzuordnungen, jedoch keinerlei Adressdaten mehr. Weiters werden keine verortbaren Aussagen über Cluster ≤ 5 (Granularität) gemacht, um Zuordnungen jedweder Art ausschließen zu können.

Statistische Methoden

Die statistischen Auswertungen basieren auf den verorteten Adresspunkten des Zentralen Melderegisters (Stand 01.01.2016) samt den zugeordneten Beurteilungslärmpegeln aus dem Straßen-, Schienen- und Luftverkehr, sowie den erhobenen Daten der Vor-Ort-Befragungen nach der vorgegebenen, geschichteten Random-Adress-Cluster-Stichprobe. Die vollständige Liste aller erfassten Variablen ist in den Anhängen *Deskriptive Statistik der Befragungen* und *Häufigkeitsstatistiken der Befragungen* einsehbar.

Eine elektronische Fassung der korrigierten und konsolidierten Rohdaten inklusive Variablenbenennungen und –Beschriftungen, Wertelabels, Kodierung gruppierter Variablen und Summenscores wurde dem Land Tirol (AdTLR) als Auftraggeberin nach Abschluss der Befragungen am 25.02.2016 schreibgeschützt und verschlüsselt in 2 verschiedenen Dateiformaten (SAV-File SPSS Version 23: Datenbank_Gesamtlaerm IBK_30-07-2017_konsolidiert.sav und XLSX-File MS Excel 2013: Datenbank_Gesamtlaerm IBK_30-07-2017_konsolidiert.xlsx) digital übermittelt.

1. Deskriptive Analyse

Die univariate Auswertung von nominalen bzw. ordinalen Variablen erfolgt durch die Angabe von absoluten bzw. relativen Häufigkeiten, bei ordinalen Parametern mit mehr als zwei Ausprägungen wurden teilweise kumulative Häufigkeiten hinzugefügt. Bei ordinalen Variablen mit einer hohen Anzahl von Ausprägungen (z.B Scores), sowie bei metrisch skalierten Variablen wurde das arithmetische Mittel als Maßzahl der zentralen Tendenz (Lageparameter) verwendet, um die Vergleichbarkeit mit anderen Studien zu ermöglichen. Eine gleichzeitige Darstellung mit anderen Methoden (Median, Modus etc.) wurde v.a. aus Gründen der Lesbarkeit vermieden. Ergänzt werden diese Auswertungen zum Teil durch die Angabe der passenden Streuungsmaße (Standardabweichung bzw. Minimum/Maximum).

Die Skalierung der Diagramme wurde aufgrund des großen Umfangs an Items und verwendeten Skalen, sowie zur Verdeutlichung der dargestellten inhaltlichen Details nicht einheitlich gewählt, sondern jeweils frei angepasst. Dieser Umstand ist in der Interpretation derselben durch die Leserinnen und Leser zu berücksichtigen.

Der statistische Zusammenhang von metrischen Variablen wurde mit Hilfe des Korrelationskoeffizienten nach Pearson evaluiert. Die mehrdimensionale Gegenüberstellung von Auswertungen erfolgt durch entsprechende Kontingenztabellen bzw. passende Grafiken. Zur Darstellung von Merkmalen von Gruppen mit sehr starker / sehr geringer Ausprägung wurden Extremgruppendarstellungen bzw. –Vergleiche verwendet.

2. Extremgruppen

Als wichtiger Indikator und generell zur Herstellung von aussagekräftigen Vergleichen wird in der internationalen Lärmwirkungsforschung und in einer Reihe von vergleichbaren Studien häufig der Prozentsatz von z.B. stark belästigten Personen (highly annoyed) verwendet. Dabei wird das Kollektiv der

Befragten nach Ihrem Antwortverhalten dichotomisiert gruppiert. Diesem Ansatz der Identifizierung von sogenannten Extremgruppen wurde auch in den Analysen dieses Gesamtberichts Rechnung getragen. Als Cut-Off-Point kann entweder ein Prozentsatz von z.B. 75% auf einer Antwortskala gewählt werden, oder es wird ab einer bestimmten Antwortausprägung gruppiert. In den gegenständlichen Auswertungen zur Gesamtlärmbetrachtung Innsbruck wurde für die verwendeten 11-er Skalen vor allem eine Gegenüberstellung der zusammengefassten Antwortkategorien 0 bis 2 und der gruppierten Ausprägungen 8, 9 und 10 verwendet.

3. Weiterführende Auswertungen und Inferenzstatistik

Der Basisbericht zu den Auswertungen der Befragungen zur Gesamtlärmbetrachtung Innsbruck soll auftragsgemäß lediglich erste Einblick in das vorhandene Datenmaterial bieten, sowie vor allem eine Deskription der Häufigkeiten enthalten und einige ausgewählte Zusammenhänge aufzeigen.

Auf Basis des Projektauftrages bleiben daher hypothesenbasierte Auswertungen ebenso wie weiterführende multivariate Analysen, sowie gewichtete adjustierte Dosis-Wirkungskurven etc. einer vertieften wissenschaftlichen Aufarbeitung vorbehalten. Aus diesem Grund und zugunsten der Lesbarkeit wurde größtenteils auf Signifikanztests (Hypothesentests) und die Angabe von p-Werten bzw. Konfidenzintervallen verzichtet. Bei der Überprüfung der Messgenauigkeit der Evaluierung (Cronbachs Alpha) wurde ein p-Wert von 5% als maximal zulässige Irrtumswahrscheinlichkeit bzw. als Signifikanzniveau verwendet.

Auswertung der Befragungen

Demographie

1. Alter und Geschlecht

Die befragte Personengruppe zeigt eine homogene Verteilung nach Alter und Geschlecht:

Abbildung 30: Bevölkerungspyramide der befragten Personen nach Altersgruppe und Geschlecht

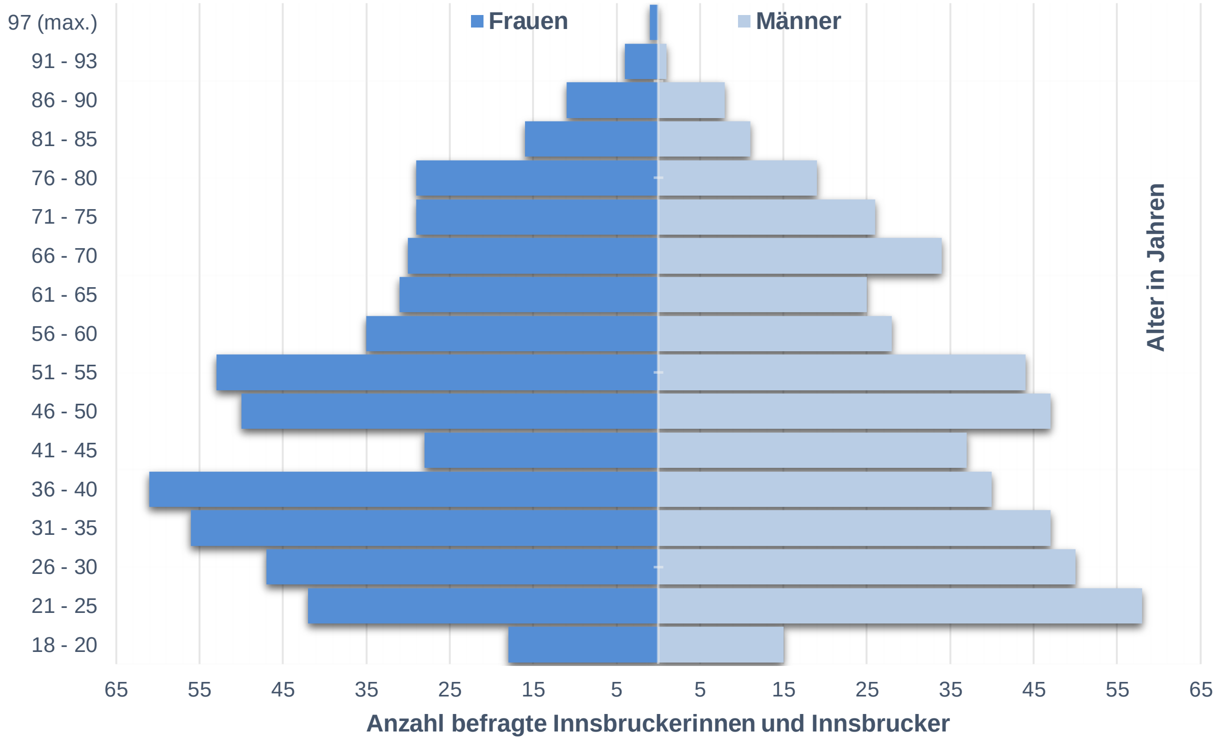

Im Mittel waren die befragten Innsbruckerinnen und Innsbrucker knapp 48 Jahre alt. Die jüngsten 7 Befragten waren zum Zeitpunkt der Befragungen im Sommer 2017 gerade 18 Jahre alt geworden, die älteste Befragte hatte ihren 97. Geburtstag gefeiert:

Tabelle 24: Mittelwert Alter

Mittelwert	Minimum	Maximum	N
47,7905	18,00	97,00	1031

2. Bildung

Um das Bildungsniveau der Befragten zu erheben, wurden die standardisierten 5 Einteilungsklassen zum höchsten bisher erreichten Schulabschluss abgefragt:

Tabelle 25: Höchster bisher erreichter Bildungsabschluss

		Häufigkeit	Gültige Prozente	Kumulierte Prozente
Gültig	Pflichtschule	98	9,5	9,5
	Abgeschlossene Lehre	166	16,1	25,6
	Fachschule ohne Matura	114	11,1	36,7
	Matura	262	25,4	62,1
	Universität, FH oder Vergleichbares	388	37,6	99,7
	Keine Angabe	3	,3	100,0
	Gesamt	1031	100,0	

Abbildung 31: Bildungsschichten kategorisiert nach höchstem erreichten Schulabschluss

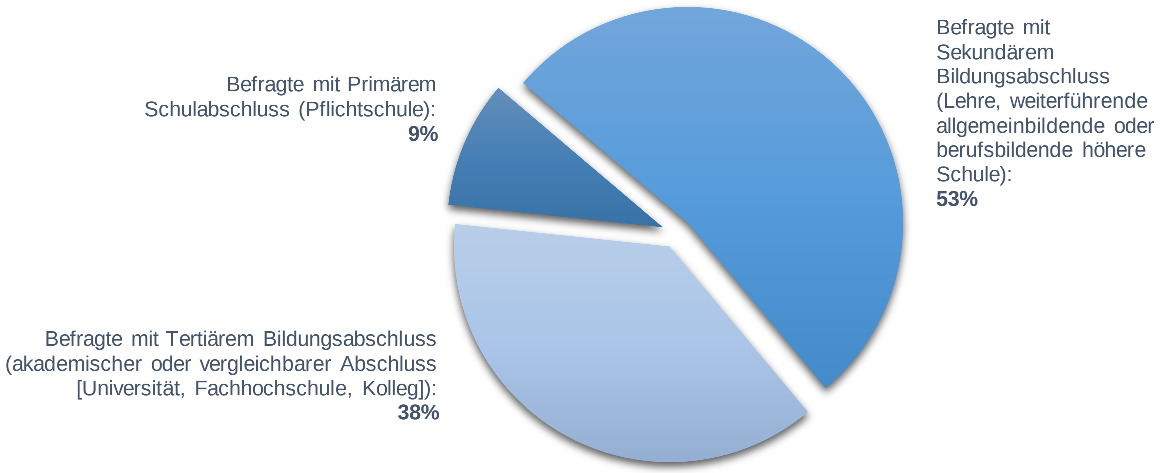

Anm.: 3 Personen machten keine Angaben

Frauen weisen im Vergleich zu den befragten Männern einen höheren Anteil an Personen mit Pflichtschulabschuss auf, liegen allerdings auch bei den tertiären Abschlüssen vor den Männern:

Abbildung 32: Höchster bisher erreichter Bildungsabschluss nach Geschlecht

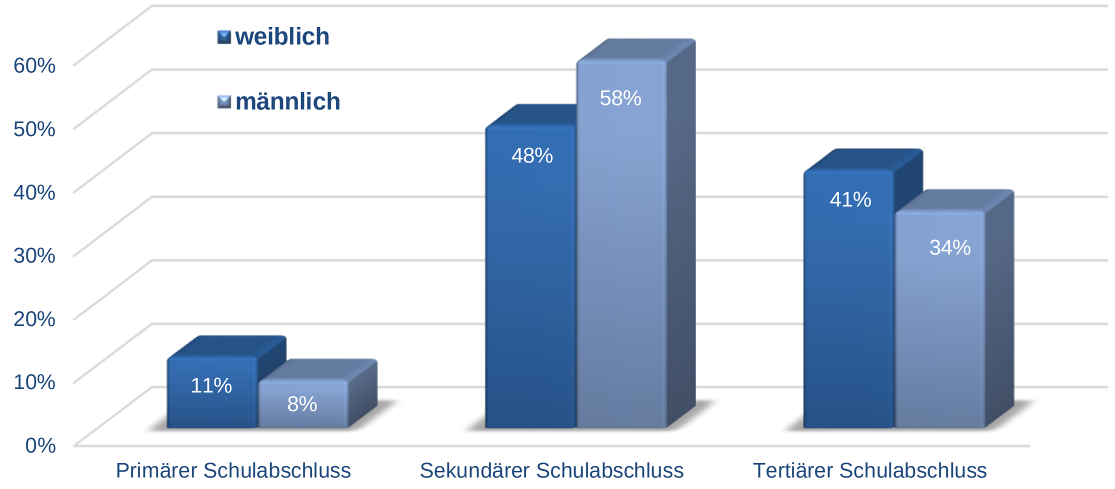

Innerhalb der drei Altersgruppen 18 bis 40, 41 bis 60 und über 60 Jahren lässt sich das ansteigende Bildungsniveau nach Altersklassen ablesen. Dieser Umstand wirkt sich noch deutlicher aus, wenn berücksichtigt wird, dass sich die Gruppe der Studierenden fast zur Gänze in der Altersklasse der unter 40-Jährigen befindet, also ihren höchsten Schulabschluss noch nicht erreicht hat:

Abbildung 33: Höchster Bildungsabschluss innerhalb kategorisierter Altersgruppen

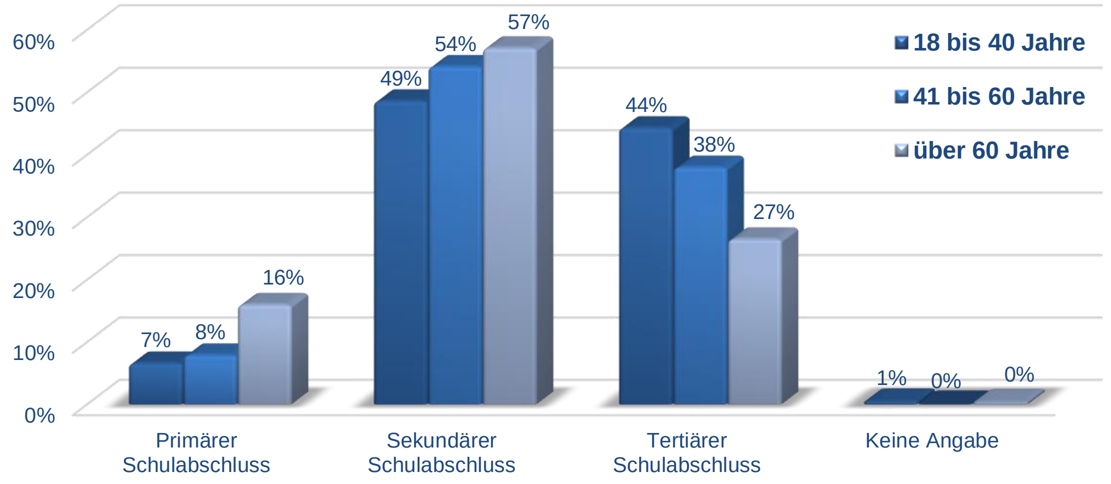

3. Berufliche Stellung

Rund die Hälfte der befragten erwachsenen Hauptwohnsitzbevölkerung Innsbrucks über 18 Jahren ist erwerbstätig, der Großteil als Angestellte(r). Ein Viertel aller Befragten stellen Pensionistinnen und Pensionisten – jede(r) 7. Befragte befindet sich noch in Ausbildung:

Abbildung 34: Berufliche Stellung der Befragten in Prozent

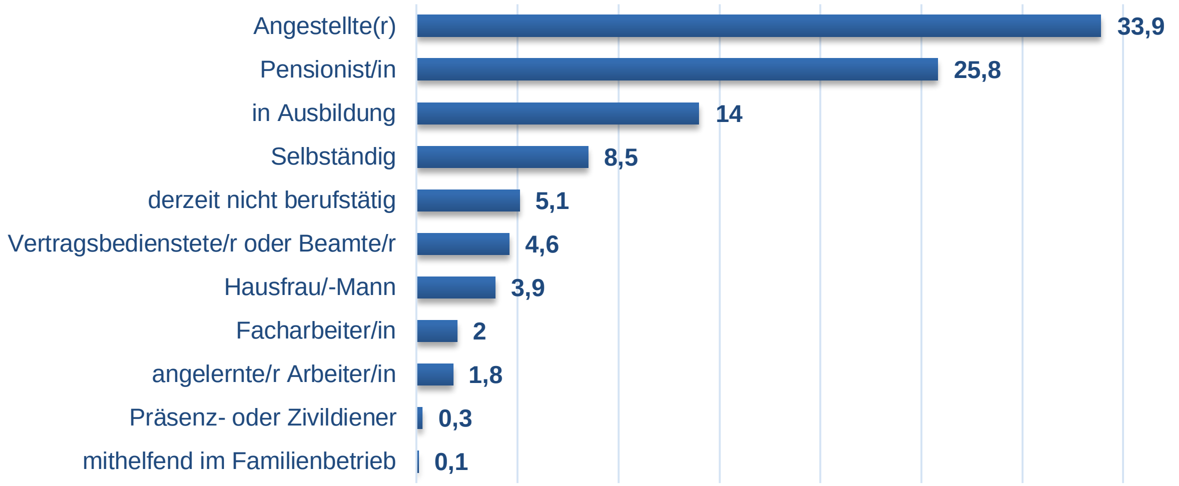

Nach den ILO-Kriterien[5] ergibt sich eine Erwerbstätigenquote von 56,4% im untersuchten Kollektiv. Kategorisiert nach ausbezahltem Erwerbseinkommen durch einen Arbeitgeber bzw. selbstständiges Erwerbseinkommen fällt die Erwerbstätigenquote auf 51,2%.

Die Erwerbstätigenquote, sowie die Art der beruflichen Stellung unterscheiden sich – wie erwartet – auch zwischen den Geschlechtern:

Tabelle 26: Kreuztabelle Geschlecht und derzeitige berufliche Stellung in Prozent

		Frauen	Männer	Gesamt
Selbständig	% innerhalb von berufl. Stellung	36,4%	63,6%	100,0%
	% innerhalb von Geschlecht	5,9%	11,4%	8,5%
Angestellte(r)	% innerhalb von berufl. Stellung	54,4%	45,6%	100,0%
	% innerhalb von Geschlecht	35,1%	32,4%	33,9%
Vertragsbedienstete/r oder Beamte/r	% innerhalb von berufl. Stellung	61,7%	38,3%	100,0%
	% innerhalb von Geschlecht	5,4%	3,7%	4,6%
Facharbeiter/in	% innerhalb von berufl. Stellung	19,0%	81,0%	100,0%
	% innerhalb von Geschlecht	0,7%	3,5%	2,0%
angelernte/r Arbeiter/in	% innerhalb von berufl. Stellung	36,8%	63,2%	100,0%
	% innerhalb von Geschlecht	1,3%	2,4%	1,8%
Hausfrau/-Mann	% innerhalb von berufl. Stellung	90,0%	10,0%	100,0%
	% innerhalb von Geschlecht	6,7%	0,8%	3,9%
in Ausbildung	% innerhalb von berufl. Stellung	45,1%	54,9%	100,0%
	% innerhalb von Geschlecht	12,0%	16,1%	14,0%
Präsenz- oder Zivildiener	% innerhalb von berufl. Stellung	0,0%	100,0%	100,0%
	% innerhalb von Geschlecht	0,0%	0,6%	0,3%
mithelfend im Familienbetrieb	% innerhalb von berufl. Stellung	0,0%	100,0%	100,0%
	% innerhalb von Geschlecht	0,0%	0,2%	0,1%

[5] Die International Labour Organisation untergliedert die Wohnbevölkerung in Erwerbspersonen (erwerbstätig, arbeitslos) und Nicht-Erwerbspersonen (Personen unter 15 Jahren; Personen mit Pensionsbezug; Schülerinnen und Schüler, sowie Studierende 15 Jahre und älter; sonstige Nicht-Erwerbspersonen). Werden Personen mehr als einer Kategorie zugeordnet, so ist die oben angeführte Reihenfolge ausschlaggebend.

Pensionist/in	% innerhalb von berufl. Stellung	54,9%	45,1%	100,0%
	% innerhalb von Geschlecht	27,0%	24,5%	25,8%
derzeit nicht berufstätig	% innerhalb von berufl. Stellung	60,4%	39,6%	100,0%
	% innerhalb von Geschlecht	5,9%	4,3%	5,1%
Gesamt	% innerhalb von berufl. Stellung	52,5%	47,5%	100,0%
	% innerhalb von Geschlecht	100,0%	100,0%	100,0%

Wohnen

1. Wohndauer im selben Haus / derselben Wohnung

Die durchschnittliche Wohndauer der Befragten in derselben Wohnung bzw. demselben Haus liegt im Mittelwert bei 17,6 Jahren. Rund ein Viertel der Befragten lebt jedoch erst seit maximal 3 Jahren in derselben Wohnung und über 11% erst seit einem vollen Jahr, was die ausgeprägte Wanderungshäufigkeit im urbanen Raum verdeutlicht:

Tabelle 27: Wohndauer im selben Haus / derselben Wohnung

		Häufigkeit	Gültige Prozente
Gültig	1 Jahr (min.)	118	11,4
	1 bis 3 Jahre	265	25,7
	1 bis 10 Jahre	533	51,7
	über 10 bis 86 Jahre (max.)	498	48,3

Die Wohndauer der Befragten in derselben Wohnung bzw. demselben Haus ist weiters stark altersabhängig:

Abbildung 35: Wohndauer in derselben Wohnung nach Alter

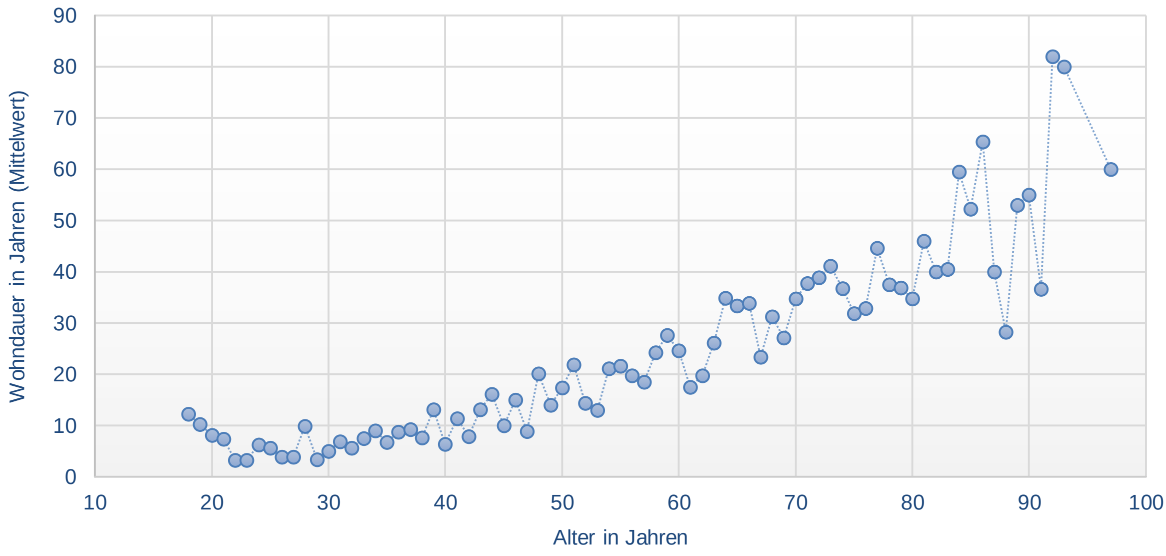

Während die jüngsten Befragten (18+) – meist seit mehreren Jahren – noch gemeinsam mit ihren Eltern wohnen, beträgt die durchschnittliche Wohndauer in der jeweiligen Wohnung bei den jungen Erwachsenen im Mittelwert nur sehr wenige Jahre und steigt mit zunehmendem Alter kontinuierlich an.[6]

[6] Anm.: Die größeren Schwankungen bei den Befragten im Alter von über 80 Jahren sind durch die kleineren Fallzahlen bedingt – Seniorenwohnheime, Krankenanstalten etc. wurden bewusst nicht befragt.

2. Art des Wohnhauses und Eigentumsverhältnisse

Dem urbanen Raum entsprechend leben drei Viertel aller befragten Innsbruckerinnen und Innsbrucker in Mehrparteienhäusern, weniger als 20% in alleinstehenden Häusern bzw. Doppelhaushälften:

Tabelle 28: Art des Wohnhauses

	Häufigkeit	Gültige Prozente	Kumulierte Prozente
Alleinstehendes Haus	142	13,8	13,8
Doppelhaushälfte	48	4,7	18,4
Reihenhaus	58	5,6	24,1
Mehrparteienhaus <10 Wohnungen	412	40,0	64,0
Mehrparteienhaus 10+ Wohnungen	363	35,2	99,2
Anderes	8	,8	100,0
Gesamt	1031	100,0	

Knapp 60% aller Befragten wohnen zur Miete oder sind Mitbewohnerinnen und Mitbewohner, in rund 40% der Fälle besitzen die befragten Personen bzw. deren unmittelbare Familie das Haus / die Wohnung in der sie leben.

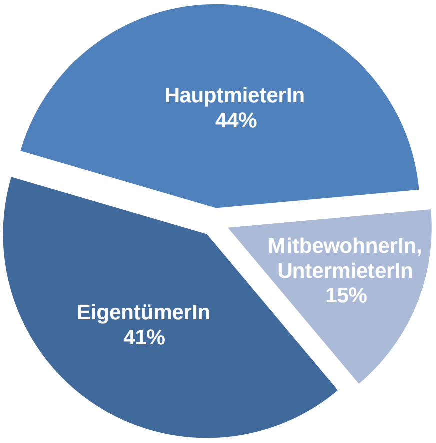

Abbildung 36: Eigentumsverhältnisse der Wohnung / des Hauses

3. Haushaltsgröße

Im Mittel leben knapp 2,5 Personen in einem durchschnittlichen befragten Haushalt. Zweipersonenhaushalte sind mit 36,6% am häufigsten. Etwas mehr als 21% aller Befragten leben allein:

Abbildung 37: Haushaltsgröße in Prozent

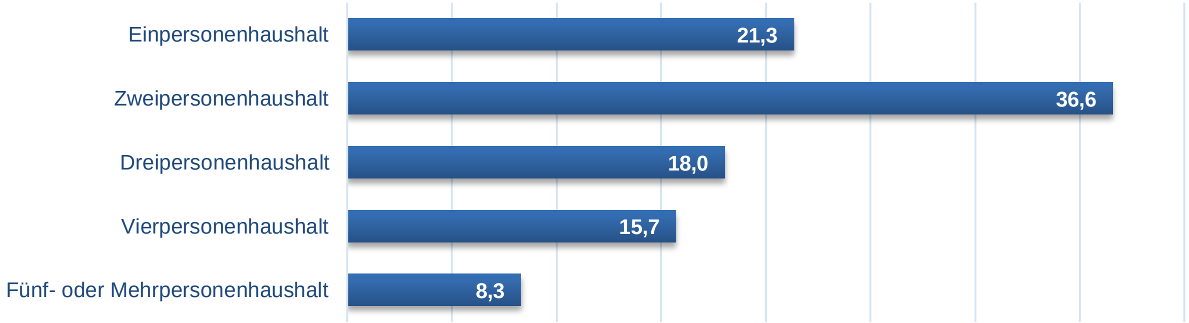

In knapp drei Viertel aller befragten Haushalte leben keine Kinder unter 18 Jahren. Im Mittel wohnen 0,47 Kinder in einem der befragten Innsbrucker Haushalte:

Tabelle 29: Anzahl an Kindern unter 18 Jahren im selben Haushalt

		Häufigkeit	Gültige Prozente	Kumulierte Prozente
Gültig	Keine Kinder	762	73,9	73,9
	1 Kind	112	10,9	84,8
	2 Kinder	111	10,8	95,5
	3 Kinder	36	3,5	99,0
	4 Kinder	7	,7	99,7
	5 Kinder	3	,3	100,0
	Gesamt	1031	100,0	

4. Stockwerk des Schlafzimmers

Die Höhe der Schlafräume über Straßenniveau ist für die Lärmwahrnehmung und –Belastung von großer Bedeutung. Über 80% der befragten Innsbruckerinnen und Innsbrucker haben ihr Schlafzimmer zwischen Erdgeschoß, erstem und zweitem Stockwerk angesiedelt:

Tabelle 30: Stockwerk des Schlafzimmers

		Häufigkeit	Gültige Prozente	Kumulierte Prozente
Gültig	2. Untergeschoß	1	0,1	0,1
	1. Untergeschoß	3	0,3	0,4
	Erdgeschoß	230	22,3	22,7
	1. Stock	363	35,2	57,9
	2. Stock	230	22,3	80,2
	3. Stock	122	11,8	92,0
	4. Stock	63	6,1	98,2
	5. Stock	11	1,1	99,2
	7. Stock	1	0,1	99,3
	8. Stock	3	0,3	99,6
	9. Stock	1	0,1	99,7
	10. Stock	2	0,2	99,9
	11. Stock	1	0,1	100,0
	Gesamt	1031	100,0	

5. Vorhandene Abschirmungen

5.1. Bauliche Abschirmungen

Drei Viertel aller Befragten geben an, dass die Wohnung / das Haus in dem sie wohnen gar nicht oder nur sehr geringfügig durch bauliche Abschirmungen wie Gebäude, Mauern etc. vor Verkehrslärm geschützt ist. Knapp ein Viertel der befragten Innsbruckerinnen und Innsbrucker gibt hingegen an, zum Großteil oder vollständig vom Verkehrslärm abgeschirmt zu wohnen:

Abbildung 38: Schutz vor Verkehrslärm durch bauliche Abschirmungen in Prozent

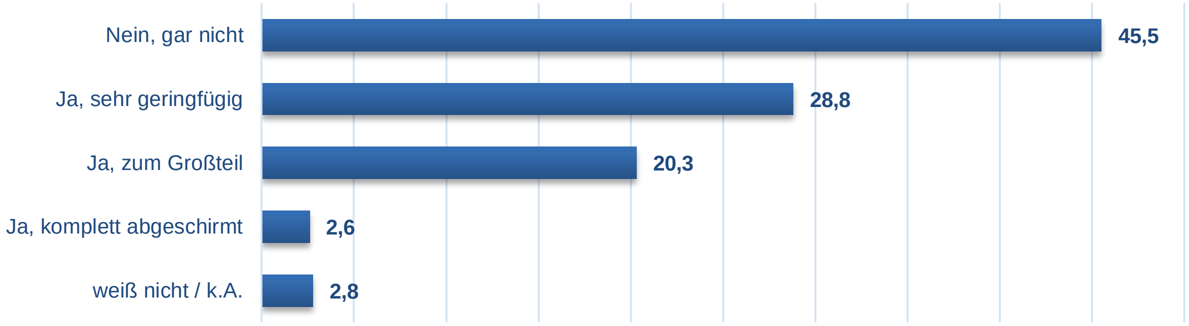

5.2. Innenhof / Lärmabgewandte Seite

Über zwei Drittel der Befragten sagen aus, ihre Wohnung bzw. das Haus, in dem sie wohnen, hätte einen vor Verkehrslärm geschützten Innenhof bzw. eine lärmabgewandte Seite:

Tabelle 31: Schutz vor Verkehrslärm durch Innenhof bzw. lärmabgewandte Seite

		Häufigkeit	Gültige Prozente	Kumulierte Prozente
Gültig	Ja	701	68,0	68,0
	Nein	308	29,9	97,9
	weiß nicht/ k.A.	22	2,1	100,0
	Gesamt	1031	100,0	

Es besteht ein signifikanter Zusammenhang (Pearsons Rho=0,67) zum *Gefühl der Ausgesetztheit gegenüber Verkehrslärm*, der *Belästigung durch Lärm insgesamt* und der *Belästigung durch Lärm in der Nacht*. Befragte, die über einen vor Verkehrslärm geschützten Innenhof verfügen, oder deren Wohnung / Haus eine lärmabgewandte Seite aufweist, fühlen sich im Mittel bei allen 3 Fragestellungen um rund eine halbe Antwortkategorie geringer belästigt bzw. ausgesetzt.

6. Lärmschutzfenster

Dezidiert über Lärmschutzfenster zu verfügen, geben 18% der Befragten an, knapp 17% wissen darüber nicht Bescheid und rund zwei Drittel haben keine Lärmschutzfenster verbaut:

Tabelle 32: Verbaute Lärmschutzfenster in den letzten 10 Jahren

		Häufigkeit	Gültige Prozente	Kumulierte Prozente
Gültig	Ja	187	18,1	18,1
	Nein	672	65,2	83,3
	weiß nicht/ k.A.	172	16,7	100,0
	Gesamt	1031	100,0	

Die 187 Befragten, die angeben über Lärmschutzfenster in ihrer Wohnung / ihrem Haus zu verfügen, wurden weiters gefragt, vor welcher Lärmquelle diese Lärmschutzfenster vorrangig schützen sollen: Knapp 60% geben den Straßenverkehrslärm als Hauptgrund an, gefolgt von Fluglärm und anderen Lärmquellen mit jeweils rund 15%:

Abbildung 39: Vorrangiges Schutzziel der Lärmschutzfenster nach Lärmquelle in Prozent

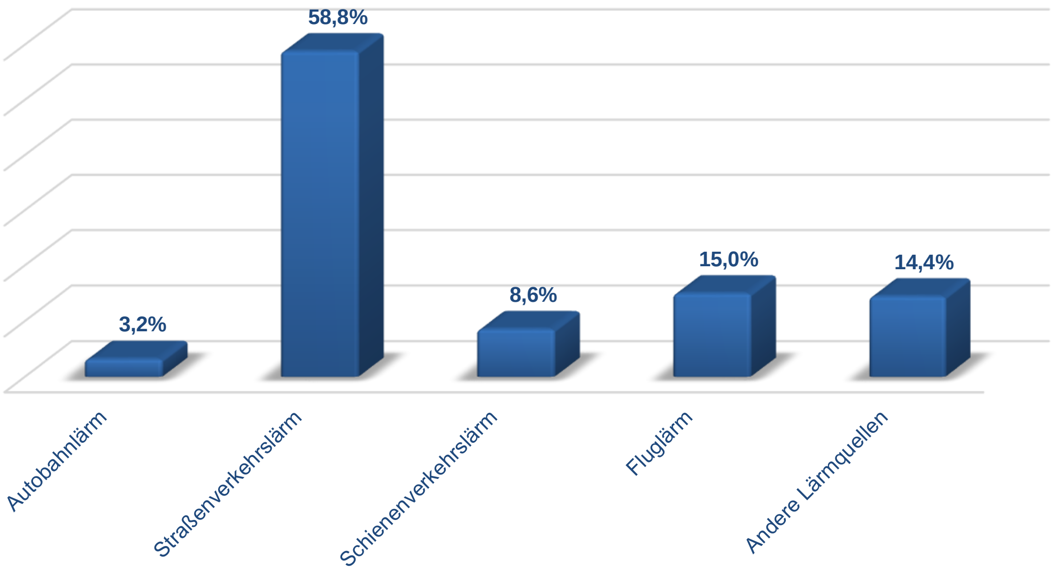

Mobilität

1. Pendlerinnen und Pendler

Nur 9% aller Befragten pendeln zur Arbeits- oder Ausbildungszwecken aus der Tiroler Landeshauptstadt in anderen Umlandgemeinden:

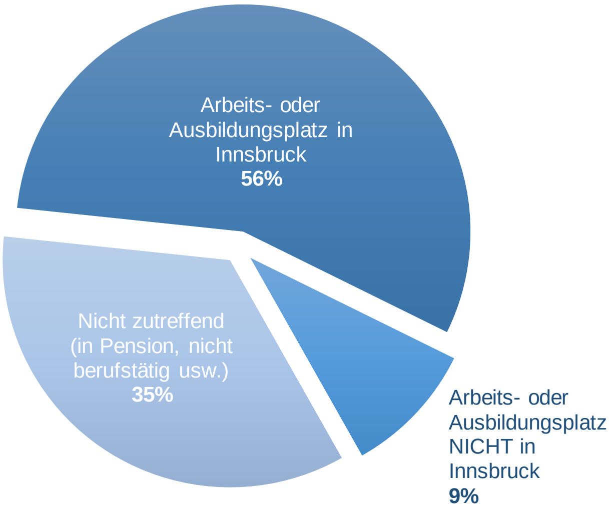

Abbildung 40: Auspendleranteil gesamt in Prozent

Der Anteil an Auspendlerinnen und Auspendlern unter den berufstätigen bzw. sich in Ausbildung befindlichen befragten Innsbruckerinnen und Innsbruckern (ohne Nichtzutreffende, die sich in Pension befinden, nicht berufstätig sind usw.) beträgt 14,6%:

Tabelle 33: Auspendleranteil an den berufstätigen bzw. sich in Ausbildung befindlichen befragten Innsbruckerinnen und Innsbruckern (ohne Nichtzutreffende)

Arbeits- oder Ausbildungsplatz in Innsbruck	573<br>85,4%
Arbeits- oder Ausbildungsplatz NICHT in Innsbruck	98<br>14,6%
Gesamt	671<br>100%
Nicht zutreffend (in Pension, nicht berufstätig etc.)	359
Keine Angabe	1
Gesamt	1031

Das von den berufstätigen bzw. sich in Ausbildung befindlichen Befragten (ohne Nichtzutreffende, die sich in Pension befinden, nicht berufstätig sind usw.) insgesamt vorrangig benutzte Verkehrsmittel auf dem Weg zum Arbeits- oder Ausbildungsplatz ist das Auto. Allerdings nutzen fast gleich viele Befragte (>30%) das Fahrrad, knapp ein Viertel öffentliche Verkehrsmittel und knapp 16% gehen zu Fuß:

Abbildung 41: Vorrangiges Verkehrsmittel auf dem Weg zum Arbeits- oder Ausbildungsplatz in Prozent

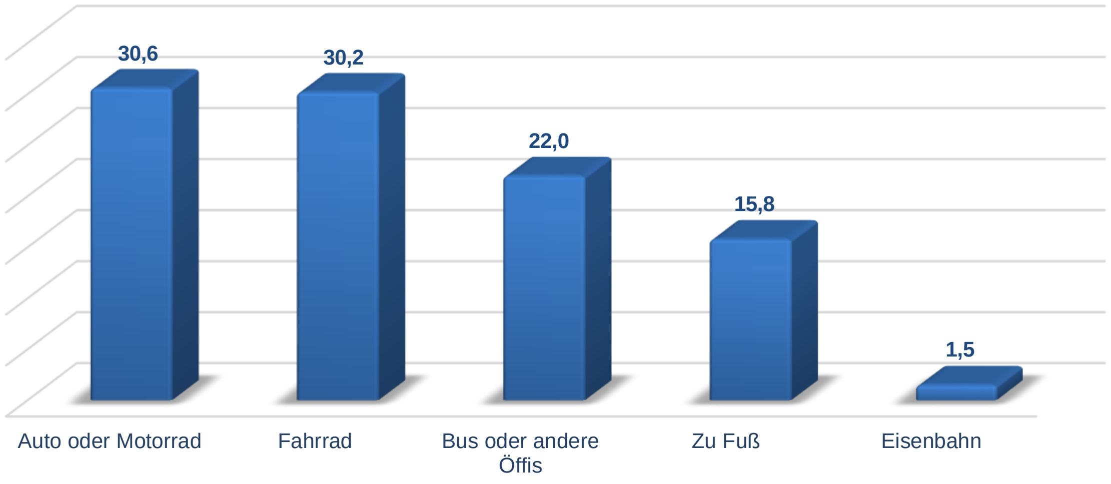

2. Mobilität im Alltag

2.1. Kraftfahrzeugnutzung als Fahrerin / Fahrer

Knapp 50% der Befragten nutzen regelmäßig (mehrmals pro Woche bis fast täglich) ein Auto als Fahrerin / Fahrer, mehr als ein Viertel hingegen nie oder fast nie. Insgesamt verfügen über 70% aller Befragten über einen Führerschein, knapp 30% nicht bzw. nutzen diesen nicht:

Abbildung 42: Häufigkeit der Nutzung von Kraftfahrzeugen als Fahrerin / Fahrer in Prozent

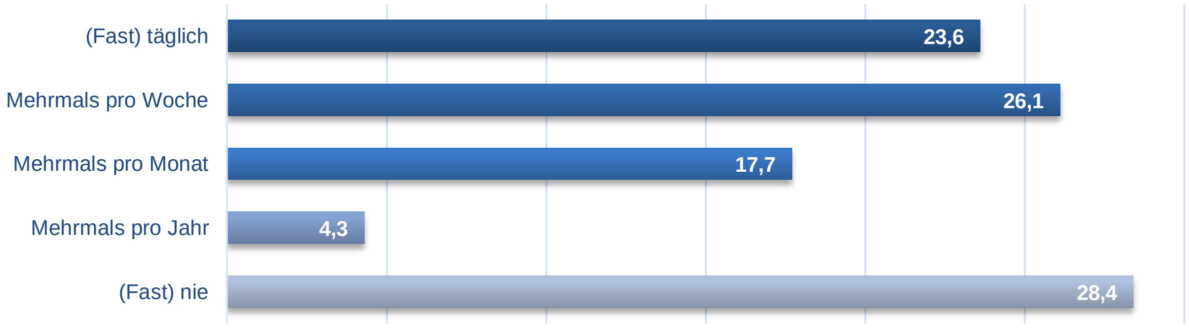

Tabelle 34: Häufigkeit der Nutzung von Kraftfahrzeugen als Fahrerin / Fahrer gruppiert

		Häufigkeit	Gültige Prozente	Kumulierte Prozente
Gültig	Starke Nutzung	512	49,7	49,7
	Gelegentliche Nutzung	182	17,7	67,3
	Geringe Nutzung	337	32,7	100,0
	Gesamt	1031	100,0	

Die Häufigkeit der Nutzung eines Kraftfahrzeuges als Fahrerin / Fahrer hängt stark mit der Lage der eigenen Wohnung / des eigenen Hauses zusammen. Bewohnerinnen und Bewohner des Zentrums

von Innsbruck (am stärksten ausgeprägt in der Katastralgemeinde Innsbruck, aber auch in Hötting, Pradl und Wilten ist dieser Effekt nachzuvollziehen) fahren wesentlich weniger mit dem Auto, als dies in Arzl, Mühlau und besonders in Igls und Vill der Fall ist. Weiters ergibt sich eine Funktion in Abhängigkeit von der Anzahl der Wohnungen, in der die befragte Person lebt: Je mehr Parteien im selben Wohnhaus, desto weniger werden private PKW's genutzt.

Männer benutzen signifikant häufiger ein Kraftfahrzeug als Fahrer als Frauen dies tun und die Altersklasse der 41- bis 60-Jährigen nutzt PKW's deutlich häufiger als die jüngere und die ältere Altersklasse, im Besonderen wenn minderjährige Kinder im selben Haushalt leben. Die Kfz-Nutzung steigt weiters mit steigendem Bildungsgrad an:

Tabelle 35: Nutzung von Kfz als Fahrerin / Fahrer und ausgewählte Variablen (gruppiert)

	Starke Nutzung		Gelegentliche Nutzung		Geringe Nutzung	
	Anzahl	Zeilen (%)	Anzahl	Zeilen (%)	Anzahl	Zeilen (%)
Amras	34	56,7%	4	6,7%	22	36,7%
Arzl	67	65,7%	13	12,7%	22	21,6%
Hötting	120	49,4%	54	22,2%	69	28,4%
Igls	27	77,1%	4	11,4%	4	11,4%
Innsbruck	58	33,0%	36	20,5%	82	46,6%
Mühlau	32	64,0%	8	16,0%	10	20,0%
Pradl	90	47,9%	25	13,3%	73	38,8%
Vill	17	81,0%	2	9,5%	2	9,5%
Wilten	67	42,9%	36	23,1%	53	34,0%
weiblich	246	45,5%	88	16,3%	207	38,3%
männlich	266	54,3%	94	19,2%	130	26,5%
18 bis 40 Jahre	198	45,0%	93	21,1%	149	33,9%
41 bis 60 Jahre	184	58,2%	61	19,3%	71	22,5%
über 60 Jahre	130	47,3%	28	10,2%	117	42,5%
Primärer Schulabschluss	36	36,7%	7	7,1%	55	56,1%
Sekundärer Schulabschluss	270	49,8%	93	17,2%	179	33,0%
Tertiärer Schulabschluss	204	52,6%	82	21,1%	102	26,3%
Haushalt ohne minderjährige Kinder	351	46,1%	136	17,8%	275	36,1%
Haushalt mit minderjährigen Kindern	161	59,9%	46	17,1%	62	23,0%
Alleinstehendes Haus	102	71,8%	14	9,9%	26	18,3%
Doppelhaushälfte	33	68,8%	9	18,8%	6	12,5%
Reihenhaus	35	60,3%	15	25,9%	8	13,8%
Mehrparteienhaus <10 Wohnungen	189	45,9%	75	18,2%	148	35,9%
Mehrparteienhaus 10+ Wohnungen	152	41,9%	68	18,7%	143	39,4%

2.2. Nutzung öffentlicher Verkehrsmittel in der Stadt

Knapp 60% der Befragten nutzen täglich bis mehrmals pro Monat öffentliche Verkehrsmittel in der Stadt. Über ein Viertel tut dies hingegen (fast) nie:

Abbildung 43: Häufigkeit der Nutzung öffentlicher Verkehrsmittel in Innsbuck in Prozent

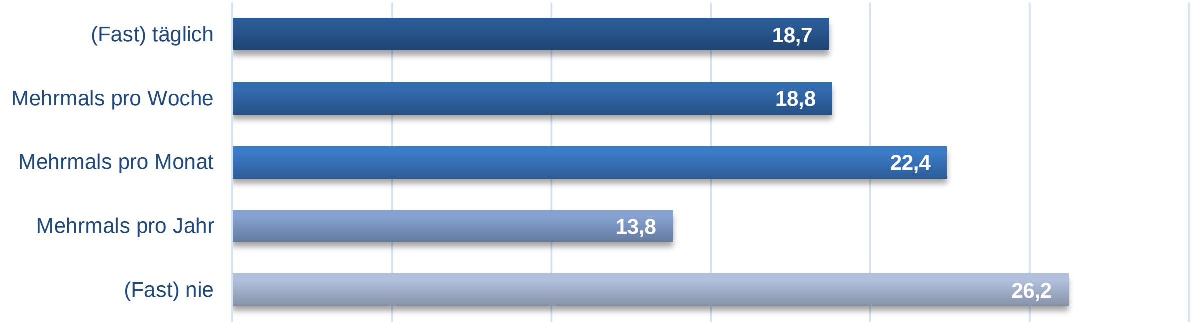

Tabelle 36: Häufigkeit der Nutzung öffentlicher Verkehrsmittel innerhalb der Stadt gruppiert

		Häufigkeit	Gültige Prozente	Kumulierte Prozente
	Starke Nutzung	387	37,5	37,5
	Gelegentliche Nutzung	231	22,4	59,9
Gültig	Geringe Nutzung	412	40,0	99,9
	Keine Angabe	1	,1	100,0
	Gesamt	1031	100,0	

Die Häufigkeit der Nutzung öffentlicher Verkehrsmittel unterscheidet sich zum Teil sehr deutlich zwischen den Katastralgemeinden, Haushalte mit minderjährigen Kindern und Männer fahren weniger häufig mit öffentlichen Verkehrsmitteln. Auch sinkt die Nutzungshäufigkeit mit steigendem Bildungsgrad:

Tabelle 37: Nutzung öffentl. Verkehrsmittel in der Stadt und ausgewählte Variablen (gruppiert)

	Starke Nutzung		Gelegentliche Nutzung		Geringe Nutzung	
	Anzahl	Zeilen (%)	Anzahl	Zeilen (%)	Anzahl	Zeilen (%)
Amras	27	45,0%	10	16,7%	23	38,3%
Arzl	37	36,3%	24	23,5%	41	40,2%
Hötting	101	41,7%	52	21,5%	89	36,8%
Igls	14	40,0%	18	51,4%	3	8,6%
Innsbruck	63	35,8%	37	21,0%	76	43,2%
Mühlau	13	26,0%	12	24,0%	25	50,0%
Pradl	78	41,5%	35	18,6%	75	39,9%
Vill	9	42,9%	6	28,6%	6	28,6%
Wilten	45	28,8%	37	23,7%	74	47,4%
weiblich	219	40,5%	118	21,8%	204	37,7%
männlich	168	34,4%	113	23,1%	208	42,5%

18 bis 40 Jahre	164	37,3%	100	22,7%	176	40,0%
41 bis 60 Jahre	96	30,5%	68	21,6%	151	47,9%
über 60 Jahre	127	46,2%	63	22,9%	85	30,9%
Primärer Schulabschluss	41	42,3%	22	22,7%	34	35,1%
Sekundärer Schulabschluss	209	38,6%	122	22,5%	211	38,9%
Tertiärer Schulabschluss	136	35,1%	86	22,2%	166	42,8%
Haushalt ohne minderjährige Kinder	310	40,7%	161	21,2%	290	38,1%
Haushalt mit minderjährigen Kindern	77	28,6%	70	26,0%	122	45,4%

2.3. Nutzung öffentlicher Verkehrsmittel außerhalb der Stadt

Die Nutzung von öffentlichen Verkehrsmitteln (Eisenbahn, Autobusse) außerhalb der Stadt Innsbruck beschränkt sich bei den Befragten größtenteils auf Gelegenheitsnutzungen, was direkt mit dem geringen Auspendleranteil in der Landeshauptstadt Innsbruck assoziiert ist:

Abbildung 44: Häufigkeit der Nutzung öffentl. Verkehrsmittel außerhalb von Innsbruck in %

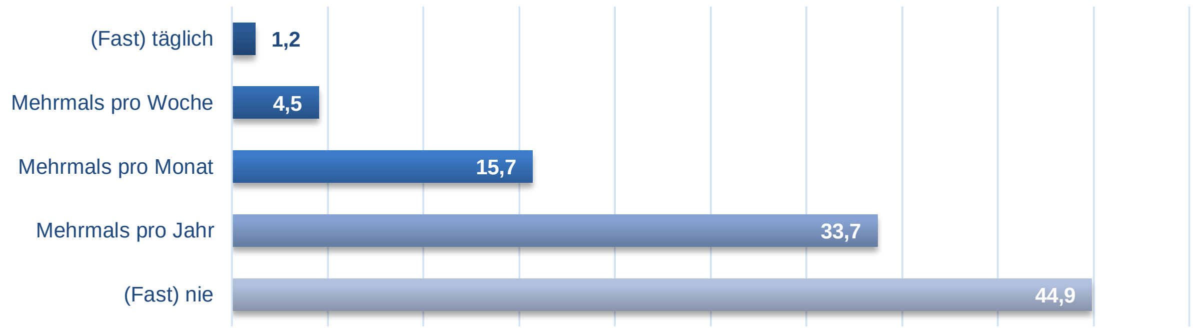

Tabelle 38: Häufigkeit der Nutzung öffentl. Verkehrsmittel außerhalb von Innsbruck gruppiert

		Häufigkeit	Gültige Prozente	Kumulierte Prozente
	Starke Nutzung	58	5,6	5,6
	Gelegentliche Nutzung	162	15,7	21,3
Gültig	Geringe Nutzung	810	78,6	99,9
	Keine Angabe	1	,1	100,0
	Gesamt	1031	100,0	

Tabelle 39: Nutzung öffentl. Verkehrsmittel außerhalb der Stadt u. ausgew. Variablen (gruppiert)

	**Starke Nutzung**		**Gelegentliche Nutzung**		**Geringe Nutzung**	
	Anzahl	Zeilen (%)	Anzahl	Zeilen (%)	Anzahl	Zeilen (%)
Amras	5	8,3%	14	23,3%	41	68,3%
Arzl	5	4,9%	14	13,7%	83	81,4%
Hötting	18	7,4%	39	16,1%	185	76,4%

Igls	0	0,0%	10	28,6%	25	71,4%
Innsbruck	9	5,1%	37	21,0%	130	73,9%
Mühlau	1	2,0%	2	4,0%	47	94,0%
Pradl	6	3,2%	21	11,2%	161	85,6%
Vill	1	4,8%	4	19,0%	16	76,2%
Wilten	13	8,3%	21	13,5%	122	78,2%
weiblich	33	6,1%	77	14,2%	431	79,7%
männlich	25	5,1%	85	17,4%	379	77,5%
18 bis 40 Jahre	32	7,3%	78	17,7%	330	75,0%
41 bis 60 Jahre	13	4,1%	35	11,1%	267	84,8%
über 60 Jahre	13	4,7%	49	17,8%	213	77,5%
Primärer Schulabschluss	6	6,2%	11	11,3%	80	82,5%
Sekundärer Schulabschluss	30	5,5%	81	14,9%	431	79,5%
Tertiärer Schulabschluss	22	5,7%	69	17,8%	297	76,5%
Alleinstehendes Haus	6	4,2%	20	14,1%	116	81,7%
Doppelhaushälfte	0	0,0%	5	10,4%	43	89,6%
Reihenhaus	3	5,2%	6	10,3%	49	84,5%
Mehrparteienhaus <10 Wohnungen	19	4,6%	69	16,7%	324	78,6%
Mehrparteienhaus 10+ Wohnungen	30	8,3%	60	16,5%	273	75,2%
Haushalt ohne minderjährige Kinder	46	6,0%	127	16,7%	588	77,3%
Haushalt mit minderjährigen Kindern	12	4,5%	35	13,0%	222	82,5%

2.4. Nutzung von Fahrrädern

Über 45% aller befragten Innsbruckerinnen und Innsbrucker nutzen über alle Altersklassen hinweg regelmäßig (täglich bis mehrmals pro Woche) ein Fahrrad und halten damit den regelmäßigen Autofahrerinnen und Autofahrern (49,7%) fast die Waage. Rund ein Drittel der Befragten benutzt (fast) nie ein Fahrrad:

Abbildung 45: Häufigkeit der Nutzung von Fahrrädern in Prozent

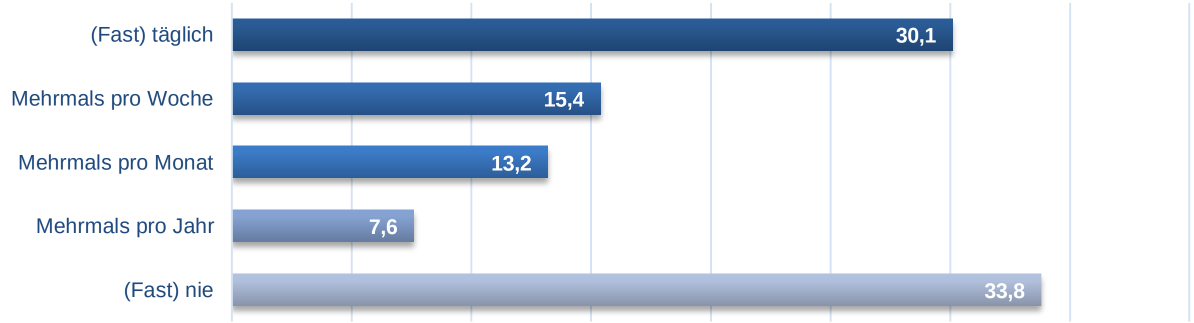

Tabelle 40: Häufigkeit der Nutzung eines Fahrrads gruppiert

		Häufigkeit	Gültige Prozente	Kumulierte Prozente
Gültig	Starke Nutzung	469	45,5	45,5
	Gelegentliche Nutzung	136	13,2	58,7
	Geringe Nutzung	426	41,3	100,0
	Gesamt	1031	100,0	

In den inneren Stadtteilen von Innsbruck wird wesentlich häufiger ein Fahrrad zur Fortbewegung benutzt, als in Amras, Arzl, Igls oder Vill. Mehr als jede(r) Zweite 18-bis 40-Jährige in Innsbruck nutzt regelmäßig ein Fahrrad. Mit zunehmendem Alter sinkt die Häufigkeit der Nutzung von Fahrrädern stark. Männer nutzen häufiger ein Fahrrad für ihre Wege in der Stadt als Frauen. Auffällig ist die starke Zunahme der Fahrradnutzung mit ansteigendem Bildungsniveau, eine Funktion, die auch nach Adjustierung des Altersfaktors erhalten bleibt:

Tabelle 41: Nutzung eines Fahrrads und ausgewählte Variablen (gruppiert)

	**Starke Nutzung**		**Gelegentliche Nutzung**		**Geringe Nutzung**	
	Anzahl	Zeilen (%)	Anzahl	Zeilen (%)	Anzahl	Zeilen (%)
Amras	19	31,7%	8	13,3%	33	55,0%
Arzl	39	38,2%	7	6,9%	56	54,9%
Hötting	117	48,1%	36	14,8%	90	37,0%
Igls	9	25,7%	8	22,9%	18	51,4%
Innsbruck	86	48,9%	29	16,5%	61	34,7%
Mühlau	21	42,0%	12	24,0%	17	34,0%
Pradl	89	47,3%	19	10,1%	80	42,6%
Vill	5	23,8%	2	9,5%	14	66,7%
Wilten	84	53,8%	15	9,6%	57	36,5%
weiblich	233	43,1%	65	12,0%	243	44,9%
männlich	236	48,2%	71	14,5%	183	37,3%
18 bis 40 Jahre	245	55,7%	63	14,3%	132	30,0%
41 bis 60 Jahre	144	45,6%	49	15,5%	123	38,9%
über 60 Jahre	80	29,1%	24	8,7%	171	62,2%
Primärer Schulabschluss	28	28,6%	11	11,2%	59	60,2%
Sekundärer Schulabschluss	224	41,3%	71	13,1%	247	45,6%
Tertiärer Schulabschluss	215	55,4%	54	13,9%	119	30,7%

2.5. Fußläufige Wege

Über 60% der Befragten gehen (fast) täglich länger als 10 Minuten zu Fuß, über 27% tun dies mehrmals pro Woche. Nur rund 5% tun dies (fast) nie oder nur mehrmals pro Jahr. Aus der Zusammenschau mit den Variablen zur Einschätzung des eigenen Gesundheitszustandes und zum Alter der Befragten wird ersichtlich, dass die letztgenannte Gruppe, welche kaum mehr als 10 Minuten am

Stück zu Fuß geht, überwiegend mit gesundheitlichen bzw. physischen Einschränkungen zu kämpfen hat:

Abbildung 46: Häufigkeit fußläufiger Wege in Prozent

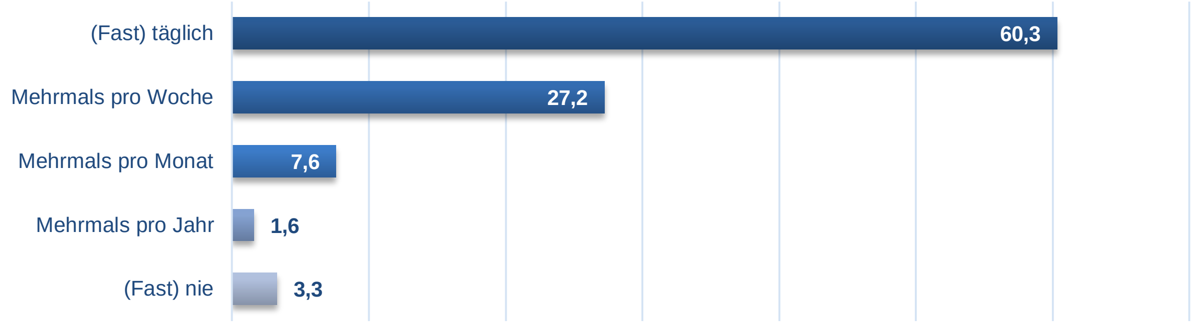

Tabelle 42: Häufigkeit fußläufiger Wege gruppiert

		Häufigkeit	Gültige Prozente	Kumulierte Prozente
Gültig	Regelmäßige FußgeherIn	902	87,5	87,5
	Gelegentliche FußgeherIn	78	7,6	95,1
	Seltene FußgeherIn	51	4,9	100,0
	Gesamt	1031	100,0	

Tabelle 43: Fußläufige Wege und ausgewählte Variablen (gruppiert)

	Regelmäßige FußgeherIn		Gelegentliche FußgeherIn		Seltene FußgeherIn	
	Anzahl	Zeilen (%)	Anzahl	Zeilen (%)	Anzahl	Zeilen (%)
Amras	54	90,0%	3	5,0%	3	5,0%
Arzl	86	84,3%	12	11,8%	4	3,9%
Hötting	204	84,0%	26	10,7%	13	5,3%
Igls	35	100,0%	0	0,0%	0	0,0%
Innsbruck	165	93,8%	7	4,0%	4	2,3%
Mühlau	41	82,0%	5	10,0%	4	8,0%
Pradl	161	85,6%	12	6,4%	15	8,0%
Vill	20	95,2%	1	4,8%	0	0,0%
Wilten	136	87,2%	12	7,7%	8	5,1%
weiblich	481	88,9%	36	6,7%	24	4,4%
männlich	421	85,9%	42	8,6%	27	5,5%
18 bis 40 Jahre	380	86,4%	41	9,3%	19	4,3%
41 bis 60 Jahre	282	89,2%	18	5,7%	16	5,1%
über 60 Jahre	240	87,3%	19	6,9%	16	5,8%
Primärer Schulabschluss	83	84,7%	5	5,1%	10	10,2%
Sekundärer Schulabschluss	464	85,6%	49	9,0%	29	5,4%
Tertiärer Schulabschluss	352	90,7%	24	6,2%	12	3,1%

2.6. Nutzung von Flugzeugen

80% aller befragten Innsbruckerinnen und Innsbrucker nutzen mehrmals pro Jahr oder alle paar Jahre ein Flugzeug. 1,5% sind regelmäßige Nutzerinnen und Nutzer (mehrmals pro Woche bzw. mehrmals pro Monat) und 18% nutzen niemals ein Flugzeug:

Abbildung 47: Häufigkeit der Nutzung von Flugzeugen in Prozent

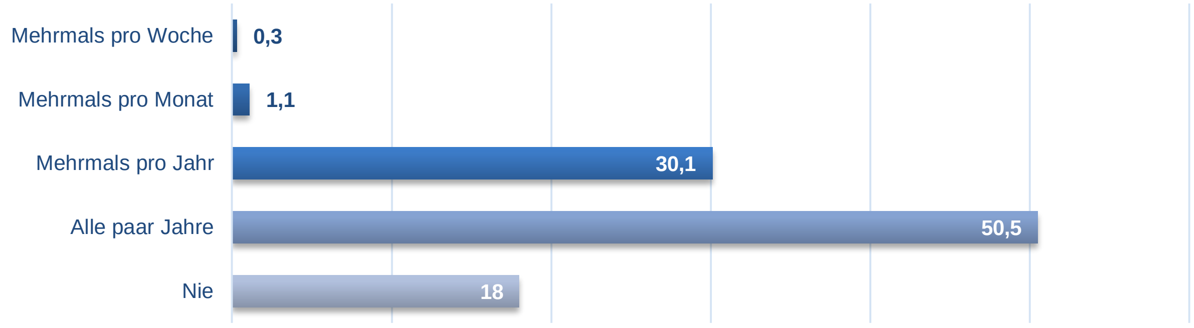

Die Frage nach der Häufigkeit der Nutzung von Flugzeugen unterscheidet sich in den Antwortkategorien – wie auch bei anderen vergleichbaren Studien üblich – von den Fragen zu den anderen Verkehrsmitteln. Dies deshalb, da eine tägliche Nutzung eines Flugzeuges, abgesehen von Flugpersonal, nahezu ausgeschlossen ist.

Die Antwortkategorie *Mehrmals pro Jahr* umfasst hingegen zum Beispiel einen einmal im Jahr stattfindenden Urlaub, der mit dem Flugzeug als Transportmittel erreicht wird und bei dem im Regelfall zumindest zwei Flüge – ein Hin- und ein Rückflug – absolviert werden.[7]

Tabelle 44: Häufigkeit der Nutzung eines Flugzeuges gruppiert

		Häufigkeit	Gültige Prozente	Kumulierte Prozente
Gültig	Starke Nutzung	14	1,4	1,4
	Gelegentliche Nutzung	310	30,1	31,4
	Geringe Nutzung	707	68,6	100,0
	Gesamt	1031	100,0	

In den Analysen zeigen sich im Mittelwertvergleich ebenso wie in den (gruppierten) bi- und multivariaten Auswertungen nur geringfügige Unterschiede zwischen den Geschlechtern, zwischen Familien mit und ohne minderjährigen Kindern im selben Haushalt und anderen Variablen.

Deutlich treten die Unterschiede im Nutzungsverhalten von Flugzeugen nach Alter und höchstem erreichten Schulabschluss hervor: Die Häufigkeit der Nutzung von Flugzeugen steigt mit zunehmendem Bildungsgrad und sinkt mit dem Alter:

[7] Das Item und die Antwortkategorien wurden im Vorfeld erfolgreich getestet und mit anderen, vergleichbaren Studien abgeglichen. In der Durchführung kam es zu keinerlei Schwierigkeiten, Unverständnis oder Nachfragen durch Probandinnen und Probanden. In einem Fall trat jedoch eine Diskrepanz mit einer befragten Person auf, die anmerkte, dass die Antwortkategorie *Einmal im Jahr* als in diesem Fall angebrachte Antwortkategorie fehle. Da sich diese befragte Person nicht auf die vorgegebenen Antwortkategorien *Mehrmals pro Jahr* oder *Alle paar Jahre* festlegen konnte, wurde der gesamte Fragebogen dieser befragten Person aus dem Datensatz entfernt.

Tabelle 45: Nutzung eines Flugzeugs und ausgewählte Variablen (gruppiert)

	Starke Nutzung (*)		Gelegentliche Nutzung		Geringe Nutzung	
	Anzahl	Zeilen (%)	Anzahl	Zeilen (%)	Anzahl	Zeilen (%)
Amras	1	1,7%	6	10,0%	53	88,3%
Arzl	1	1,0%	28	27,5%	73	71,6%
Hötting	4	1,6%	81	33,3%	158	65,0%
Igls	0	0,0%	12	34,3%	23	65,7%
Innsbruck	3	1,7%	56	31,8%	117	66,5%
Mühlau	1	2,0%	21	42,0%	28	56,0%
Pradl	1	0,5%	54	28,7%	133	70,7%
Vill	0	0,0%	6	28,6%	15	71,4%
Wilten	3	1,9%	46	29,5%	107	68,6%
weiblich	5	0,9%	156	28,8%	380	70,2%
männlich	9	1,8%	154	31,4%	327	66,7%
18 bis 40 Jahre	4	0,9%	164	37,3%	272	61,8%
41 bis 60 Jahre	8	2,5%	87	27,5%	221	69,9%
über 60 Jahre	2	0,7%	59	21,5%	214	77,8%
Primärer Schulabschluss	0	0,0%	12	12,2%	86	87,8%
Sekundärer Schulabschluss	5	0,9%	132	24,4%	405	74,7%
Tertiärer Schulabschluss	9	2,3%	165	42,5%	214	55,2%

(*) Aufgrund der kleinen Fallzahlen sind valide Aussagen zur Gruppe mit „Starker Nutzung“ nur sehr eingeschränkt sinnvoll.

Lebensbedingungen in Innsbruck

1. Überblick Lebensbedingungen in Innsbruck

Mittels 8 Fragen und einer 11-teiligen Skala von 0 (*gar nicht gut*) bis 10 (*außerordentlich gut*) wurde die Güte der Wohn- und Lebensbedingungen in der Landeshauptstadt Innsbruck abgefragt.

Die Güte der Lebensbedingungen wird in allen Teilbereichen überwiegend sehr hoch eingeschätzt. Der Mittelwert aller Antworten über alle 8 Fragen hinweg (ohne jene vereinzelten Personen, die zu einzelnen Fragen keine Angaben machen konnten/wollten) liegt bei 6,8 (74,7%):

Abbildung 48: Mittelwertvergleich der Lebensbedingungen

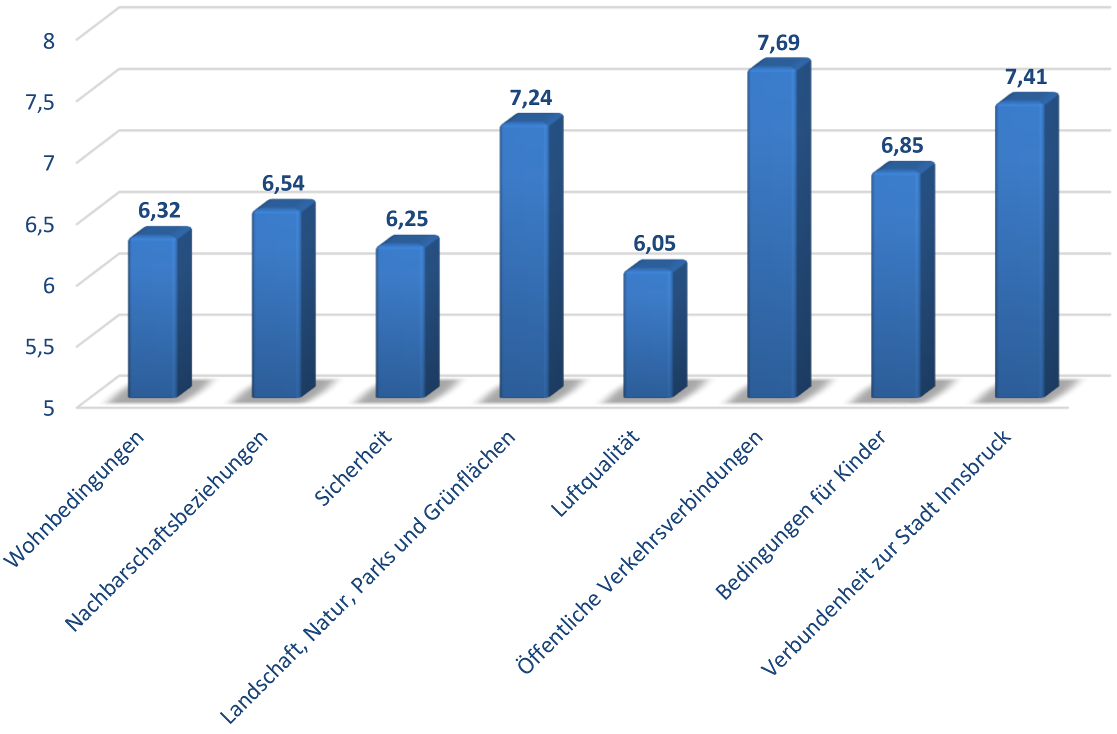

Am höchsten werden die öffentlichen Verkehrsmittel, die eigene Verbundenheit zur Stadt Innsbruck und die Frage nach der Güte von Landschaft, Natur, öffentlichen Parks und Grünflächen beurteilt. Vergleichsweise deutlich niederer die Wohnbedingungen, die Sicherheit und die Luftqualität. Die Nachbarschaftsbeziehungen und die Bedingungen für das Aufwachsen von Kindern liegen dazwischen.

2. Wohnbedingungen in Innsbruck

Knapp 38% aller Befragten beurteilen die Wohnbedingungen in Innsbruck in den drei höchsten Antwortkategorien und somit als sehr gut, 8,1% in den drei Untersten und damit als schlecht. Rund 54% der Antworten verteilen sich auf die fünf mittleren Kategorien. 2 Probandinnen und Probanden geben auf die Frage nach der Güte der Wohnbedingungen in Innsbruck keine Antwort:

Abbildung 49: Beurteilung der Wohnbedingungen in Prozent

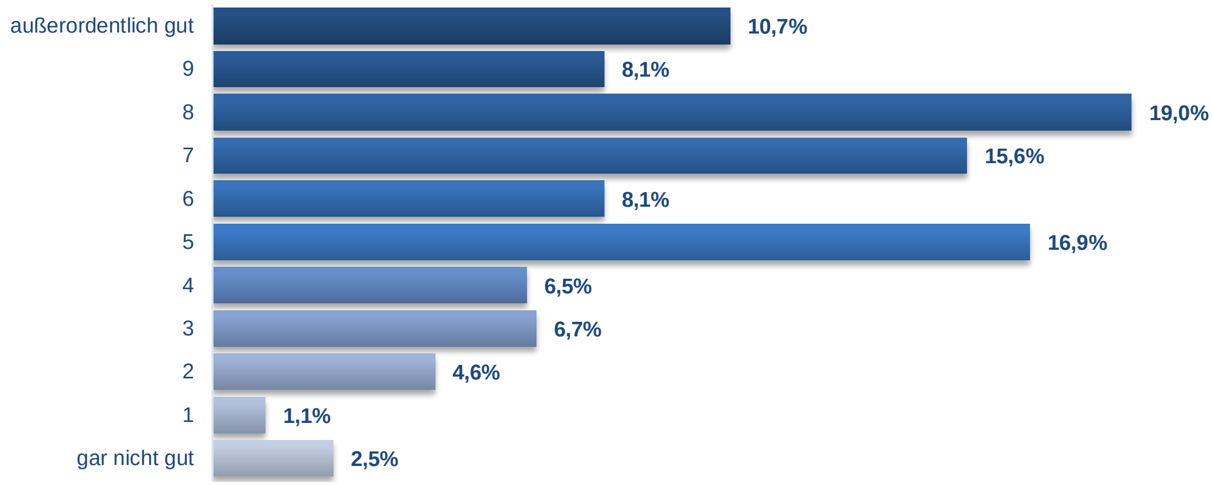

Tabelle 46: Beurteilung der Wohnbedingungen gruppiert

		Häufigkeit	Gültige Prozente	Kumulierte Prozente
Gültig	Nicht gut	84	8,1	8,1
	Durchschnittlich	555	53,8	62,0
	Sehr gut	390	37,8	99,8
	w.n. / k.A.	2	,2	100,0
	Gesamt	1031	100,0	

3. Nachbarschaftsbeziehungen in Innsbruck

Rund 40% aller Befragten beurteilen die Nachbarschaftsbeziehungen in Innsbruck als sehr gut (in den drei höchsten Antwortkategorien), 6,6% als schlecht (in den drei Untersten):

Abbildung 50: Beurteilung der Nachbarschaftsbeziehungen in Prozent

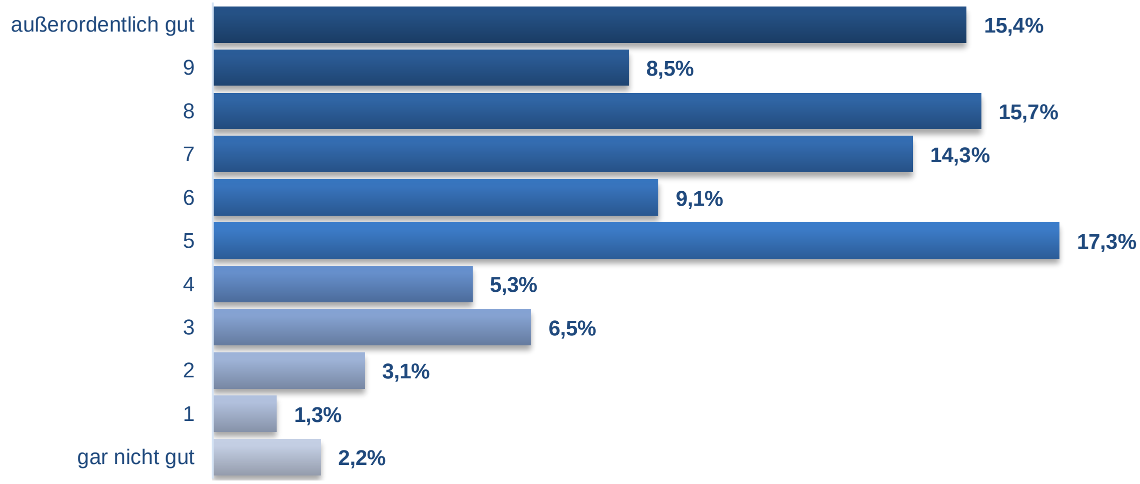

52,5% der Antworten verteilen sich auf die fünf mittleren Kategorien. 13 Probandinnen und Probanden geben auf die Frage nach der Güte der Nachbarschaftsbeziehungen in Innsbruck keine Antwort:

Tabelle 47: Beurteilung der Nachbarschaftsbeziehungen gruppiert

		Häufigkeit	Gültige Prozente	Kumulierte Prozente
Gültig	Nicht gut	68	6,6	6,6
	Durchschnittlich	541	52,5	59,1
	Sehr gut	409	39,7	98,7
	w.n. / k.A.	13	1,3	100,0
	Gesamt	1031	100,0	

4. Sicherheit in Innsbruck

38% aller befragten Innsbruckerinnen und Innsbrucker beurteilen die Sicherheit in Innsbruck in den drei höchsten Antwortkategorien und somit als sehr gut, 10,6% in den drei Untersten und damit als schlecht. Etwas mehr als die Hälfte der Antworten verteilen sich auf die fünf mittleren Kategorien. Eine Probandin bzw. ein Proband gibt auf die Frage nach der Güte der Sicherheit in Innsbruck keine Antwort:

Abbildung 51: Beurteilung der Sicherheit in Prozent

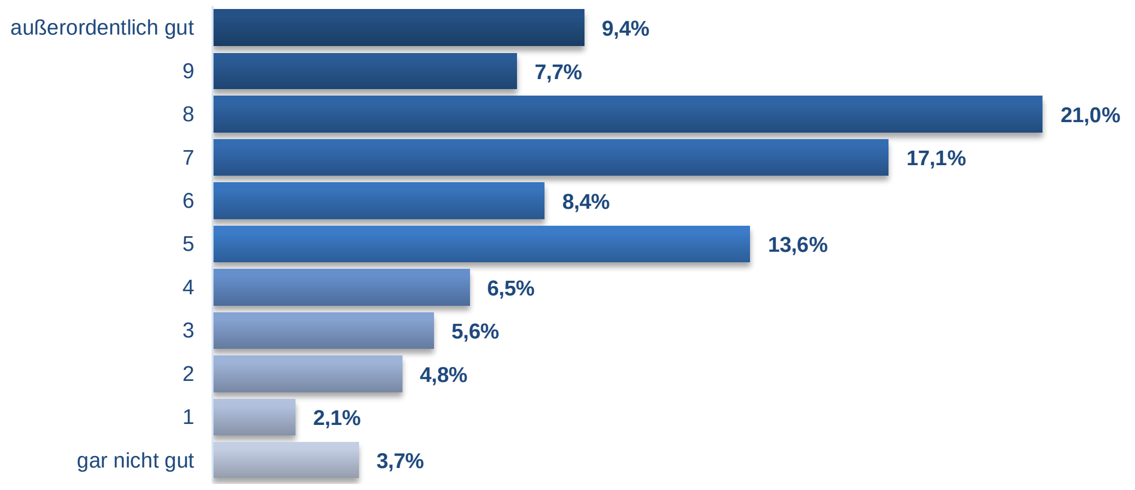

Tabelle 48: Beurteilung der Sicherheit gruppiert

		Häufigkeit	Gültige Prozente	Kumulierte Prozente
Gültig	Nicht gut	109	10,6	10,6
	Durchschnittlich	528	51,2	61,8
	Sehr gut	393	38,1	99,9
	w.n. / k.A.	1	,1	100,0
	Gesamt	1031	100,0	

5. Landschaft, Natur, Parks und Grünflächen in Innsbruck

Knapp 5% aller Befragten beurteilen die Landschaft und Natur, die öffentlichen Parks und Grünflächen in Innsbruck in den drei untersten Antwortkategorien und damit als schlecht, über 55% in den drei Höchsten und somit als sehr gut. Etwas weniger als 40% der Antworten verteilen sich auf die fünf mittleren Kategorien:

Abbildung 52: Beurteilung von Landschaft, Natur, Parks und Grünflächen in Prozent

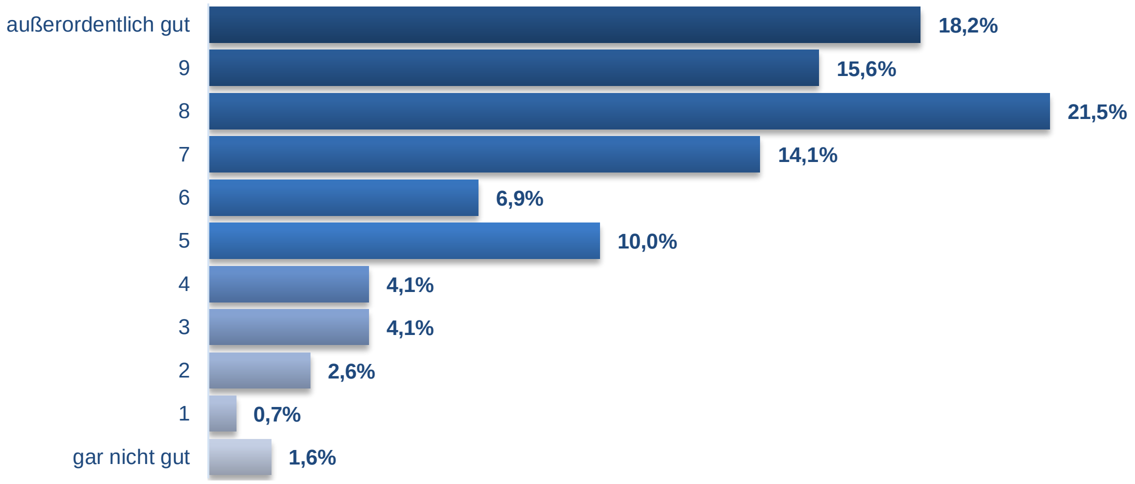

6 Probandinnen und Probanden geben auf die Frage nach der Landschaft und Natur, den öffentlichen Parks und Grünflächen in Innsbruck keine Antwort:

Tabelle 49: Beurteilung der Landschaft, Natur, Parks und Grünflächen gruppiert

		Häufigkeit	Gültige Prozente	Kumulierte Prozente
Gültig	Nicht gut	51	4,9	4,9
	Durchschnittlich	403	39,1	44,0
	Sehr gut	571	55,4	99,4
	w.n. / k.A.	6	,6	100,0
	Gesamt	1031	100,0	

6. Luftqualität in Innsbruck

Rund 58% der Antworten zur Luftqualität in Innsbruck verteilen sich auf die fünf mittleren Kategorien. Etwas mehr als 33% aller Befragten beurteilen die Luftqualität in den drei höchsten Antwortkategorien und somit als sehr gut, 8,2% in den drei Untersten und damit als schlecht. 1 Probandin / Proband gibt auf die Frage nach der Güte der Luftqualität in Innsbruck keine Antwort:

Abbildung 53: Beurteilung der Luftqualität in Prozent

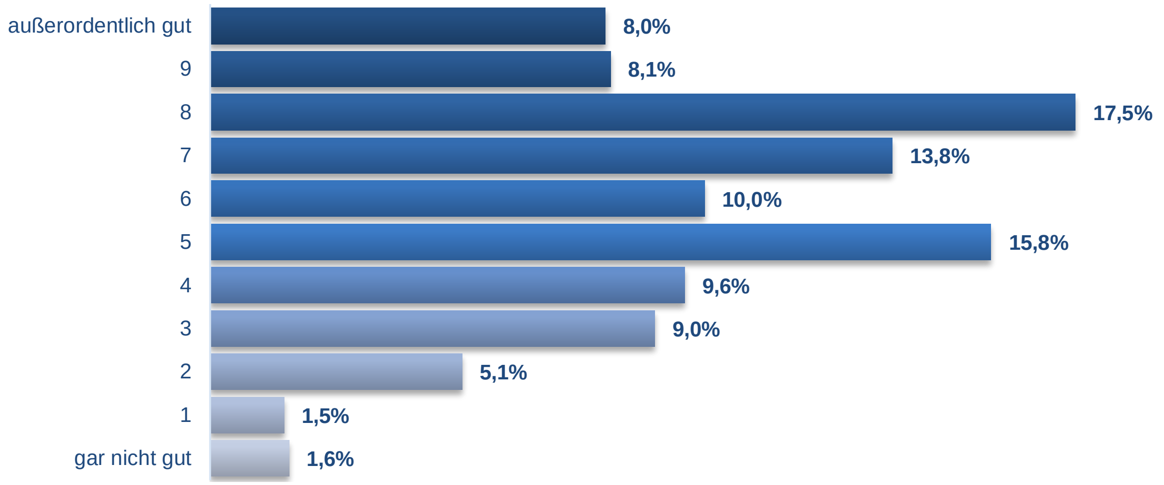

Tabelle 50: Beurteilung der Luftqualität gruppiert

		Häufigkeit	Gültige Prozente	Kumulierte Prozente
Gültig	Nicht gut	85	8,2	8,2
	Durchschnittlich	600	58,2	66,4
	Sehr gut	345	33,5	99,9
	w.n. / k.A.	1	,1	100,0
	Gesamt	1031	100,0	

7. Öffentliche Verkehrsmittel in Innsbruck

Fast 64% aller Befragten beurteilen die öffentlichen Verkehrsmittel in Innsbruck in den drei höchsten Antwortkategorien und somit als sehr gut, 2,6% in den drei Untersten und damit als schlecht:

Abbildung 54: Beurteilung der öffentlichen Verkehrsmittel in Prozent

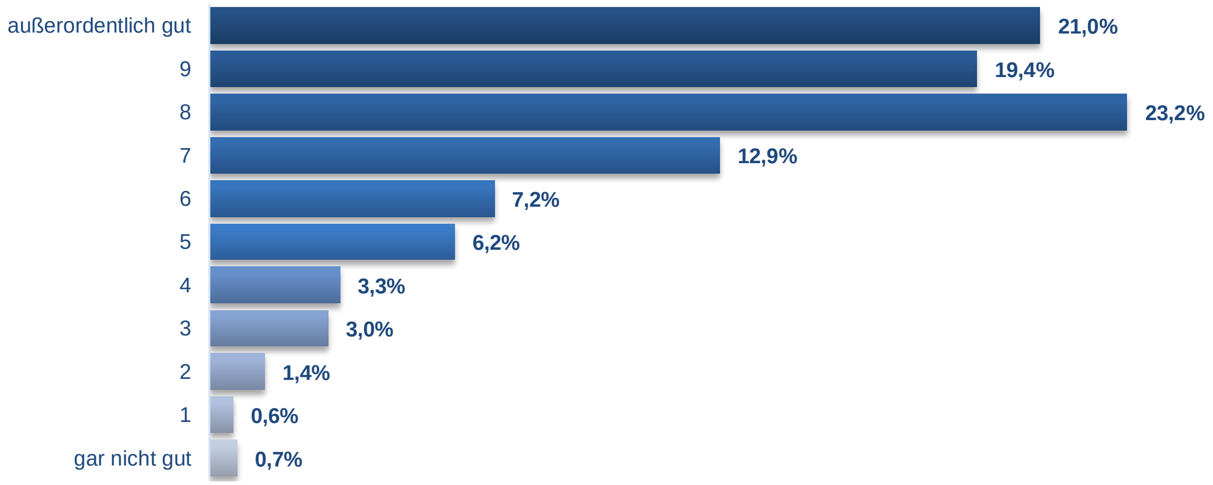

Weniger als ein Drittel der Antworten verteilen sich auf die fünf mittleren Kategorien. 12 Probandinnen und Probanden geben auf die Frage nach der Güte der öffentlichen Verkehrsmittel keine Antwort:

Tabelle 51: Beurteilung der öffentlichen Verkehrsverbindungen gruppiert

		Häufigkeit	Gültige Prozente	Kumulierte Prozente
Gültig	Nicht gut	27	2,6	2,6
	Durchschnittlich	336	32,6	35,2
	Sehr gut	656	63,6	98,8
	w.n. / k.A.	12	1,2	100,0
	Gesamt	1031	100,0	

8. Bedingungen für das Aufwachsen von Kinder in Innsbruck

50% der Antworten zu den Bedingungen für das Aufwachsen von Kinder in Innsbruck entfallen auf die fünf mittleren Kategorien. Etwas mehr als 43% aller Befragten beurteilen diese in den drei höchsten Antwortkategorien und somit als sehr gut, 4,6% in den drei Untersten und damit als schlecht. 22 Probandinnen und Probanden geben auf die Frage nach der Güte der Bedingungen für das Aufwachsen von Kinder in Innsbruck keine Antwort:

Abbildung 55: Beurteilung der Bedingungen für das Aufwachsen von Kinder in Prozent

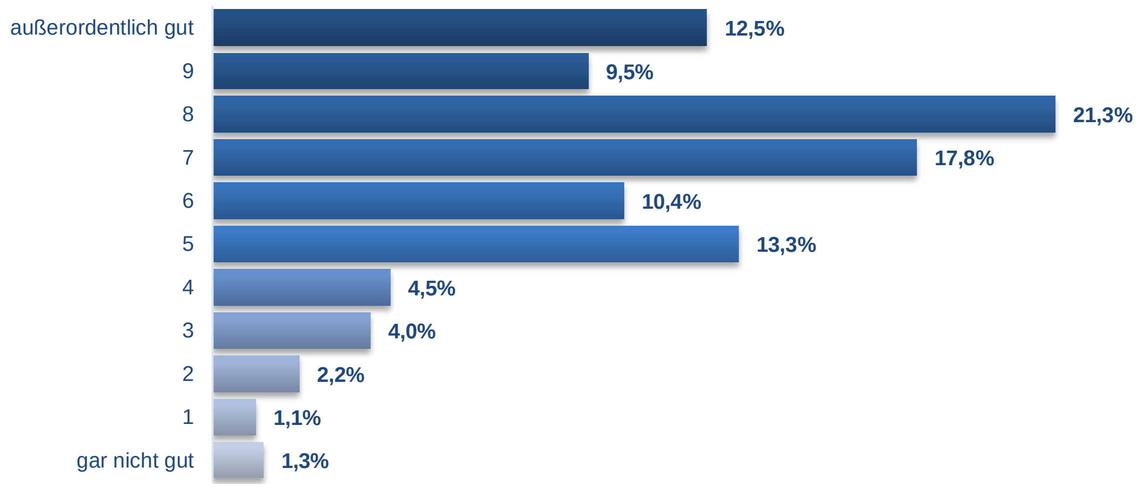

Tabelle 52: Beurteilung der Bedingungen für das Aufwachsen von Kinder gruppiert

		Häufigkeit	Gültige Prozente	Kumulierte Prozente
Gültig	Nicht gut	47	4,6	4,6
	Durchschnittlich	515	50,0	54,5
	Sehr gut	447	43,4	97,9
	w.n. / k.A.	22	2,1	100,0
	Gesamt	1031	100,0	

9. Verbundenheit zur Stadt Innsbruck

Knapp 58% aller Befragten fühlen sich der Stadt Innsbruck sehr verbunden und antworten in den drei höchsten Antwortkategorien, 5,3% sehen sich der Stadt Innsbruck nicht verbunden und antworten in den drei untersten Kategorien. Etwas mehr als ein Drittel der Antworten verteilt sich auf die fünf mittleren Kategorien. 2 Probandinnen und Probanden geben auf die Frage nach der Verbundenheit zur Stadt Innsbruck keine Antwort:

Abbildung 56: Beurteilung der Verbundenheit zur Stadt Innsbruck in Prozent

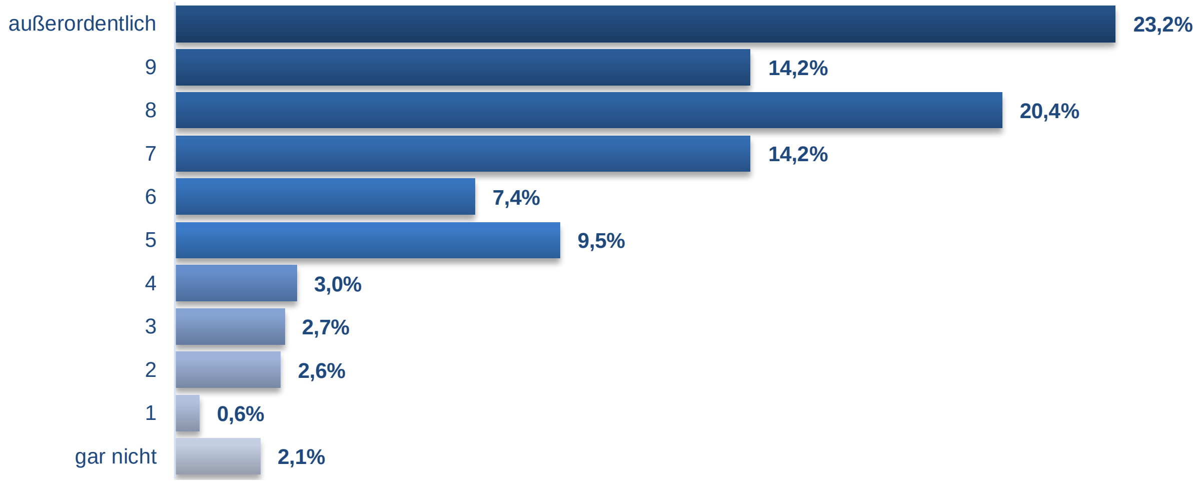

Tabelle 53: Beurteilung der Verbundenheit zur Stadt Innsbruck gruppiert

		Häufigkeit	Gültige Prozente	Kumulierte Prozente
	Nicht verbunden	55	5,3	5,3
	Durchschnittlich	379	36,8	42,1
Gültig	Sehr verbunden	595	57,7	99,8
	w.n. / k.A.	2	,2	100,0
	Gesamt	1031	100,0	

10. Lebensbedingungen und ausgewählte Parameter

Im Folgenden werden eine Reihe von ausgewählten Überblicksauswertungen (verkürzt und zur Veranschaulichung größtenteils über gruppierte Mittelwertvergleiche) dargestellt. Die Antwortkategorien *weiß nicht / keine Angabe* wurden im Sinne einer besseren Übersichtlichkeit ausgeklammert.

10.1. Lebensbedingungen in Innsbruck und Katastralgemeinde

Die Beurteilung der Lebens- und Wohnbedingungen unterscheidet sich ausgeprägt zwischen den einzelnen Katastralgemeinden von Innsbruck. Im Summenscore über alle 8 Fragen hinweg, schätzen die Befragten in Mühlau die Wohn- und Lebensbedingungen am höchsten und die befragten Einwoh-

nerinnen und Einwohner von Amras am niedrigsten ein. Insgesamt kann festgestellt werden, dass die Befragten in den äußeren Katastralgemeinden die Lebensbedingungen in Innsbruck höher einschätzen, als diejenigen in den innerstädtischeren Gemeinden:

Abbildung 57: Summenscore Lebensbedingungen und Katastralgemeinde im Mittelwertvergleich (aufsteigend sortiert)

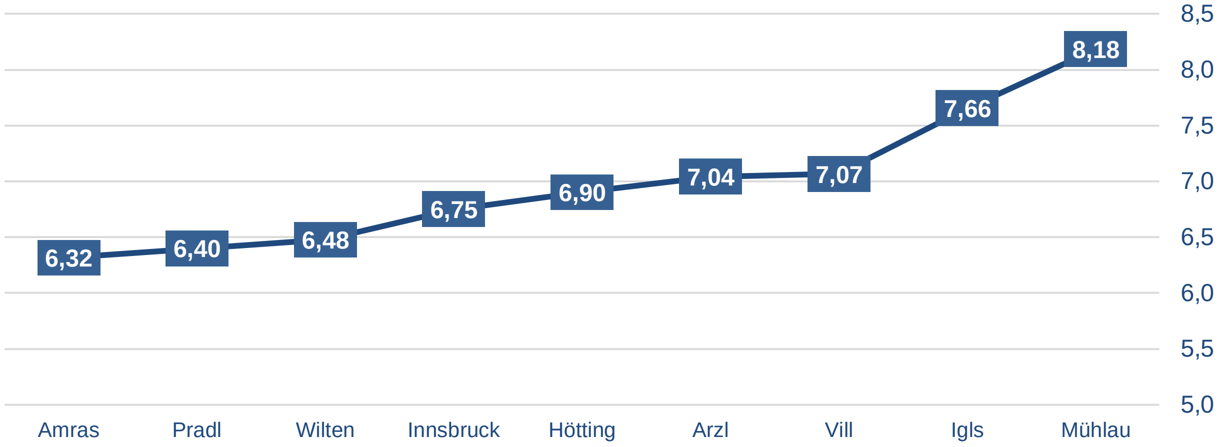

Tabelle 54: Lebensbedingungen und Katastralgemeinde im Mittelwertvergleich

Katastralgemeinde		Wohnbedingungen	Nachbarschaftsbeziehungen	Sicherheit	Landschaft und Natur	Luftqualität	Öffentl. Verkehr	Bedingungen für Kinder	Verbundenheit zu Innsbruck
**Amras**	**Mittelwert**	**5,58**	**5,95**	**5,38**	**6,68**	**5,42**	**8,02**	**6,56**	**6,95**
	N	60	57	60	59	60	60	57	58
	Standardabweichung	2,417	2,621	2,532	2,825	2,280	2,021	2,276	2,837
**Arzl**	**Mittelwert**	**6,45**	**7,49**	**6,05**	**7,60**	**6,37**	**7,58**	**7,40**	**7,40**
	N	102	102	102	102	102	102	102	102
	Standardabweichung	2,362	2,354	2,682	2,112	2,320	2,231	2,050	2,326
**Hötting**	**Mittelwert**	**6,47**	**6,77**	**6,67**	**7,32**	**5,98**	**7,32**	**7,16**	**7,53**
	N	241	234	242	238	243	240	230	243
	Standardabweichung	2,477	2,567	2,201	2,143	2,290	2,264	1,988	2,355
**Igls**	**Mittelwert**	**7,37**	**7,31**	**6,80**	**8,40**	**7,83**	**9,31**	**7,34**	**6,89**
	N	35	35	35	35	35	35	35	35
	Standardabweichung	1,437	1,207	1,389	1,265	1,150	,796	1,514	1,728
**Innsbruck**	**Mittelwert**	**6,12**	**6,14**	**6,07**	**7,57**	**6,10**	**7,59**	**6,76**	**7,65**
	N	176	176	176	176	175	175	173	176
	Standardabweichung	2,472	2,432	2,375	2,307	2,503	2,115	2,264	2,320
**Mühlau**	**Mittelwert**	**8,82**	**7,68**	**8,52**	**9,08**	**7,72**	**7,34**	**9,00**	**7,30**
	N	50	50	50	50	50	50	50	50
	Standardabweichung	1,574	2,226	1,741	1,523	2,176	2,076	1,512	2,757
**Pradl**	**Mittelwert**	**5,88**	**6,10**	**5,30**	**6,65**	**6,08**	**7,88**	**5,91**	**7,40**
	N	188	188	188	188	188	188	188	188
	Standardabweichung	2,552	2,517	3,111	2,647	2,535	2,111	2,496	2,507

Vill	**Mittelwert**	**5,95**	**7,67**	**7,00**	**7,05**	**5,76**	**8,19**	**7,67**	**7,24**
	N	21	21	21	21	21	21	21	21
	Standardabweichung	2,692	2,309	1,924	2,224	3,097	1,662	1,798	2,322
Wilten	**Mittelwert**	**6,06**	**6,09**	**6,42**	**6,57**	**5,19**	**7,78**	**6,44**	**7,28**
	N	156	155	156	156	156	148	153	156
	Standardabweichung	2,450	2,632	2,499	2,383	2,364	1,883	2,083	2,346
Insgesamt	**Mittelwert**	**6,32**	**6,54**	**6,25**	**7,24**	**6,05**	**7,69**	**6,85**	**7,41**
	N	1029	1018	1030	1025	1030	1019	1009	1029
	Standardabweichung	2,491	2,528	2,585	2,379	2,445	2,112	2,246	2,404

10.2. Lebensbedingungen in Innsbruck und Geschlecht

Frauen beurteilen die Nachbarschaftsbeziehungen In Innsbruck besser, die Wohnbedingungen und die Sicherheit schlechter als Männer und vice versa. Die Beurteilung der Lebensbedingungen in Innsbruck zeigt bei den meisten der anderen Einzelfragen nur vergleichsweise geringe Abweichungen zwischen den Geschlechtern:

Tabelle 55: Lebensbedingungen und Geschlecht im Mittelwertvergleich

Geschlecht		Wohnbedingungen	Nachbarschaftsbeziehungen	Sicherheit	Landschaft und Natur	Luftqualität	Öffentl. Verkehr	Bedingungen für Kinder	Verbundenheit zu Innsbruck
weiblich	**Mittelwert**	**6,17**	**6,69**	**6,10**	**7,18**	**6,01**	**7,78**	**6,82**	**7,48**
	N	539	536	541	540	541	534	532	541
	Standardabweichung	2,531	2,613	2,580	2,469	2,428	2,131	2,292	2,361
männlich	**Mittelwert**	**6,49**	**6,38**	**6,41**	**7,30**	**6,10**	**7,59**	**6,88**	**7,33**
	N	490	482	489	485	489	485	477	488
	Standardabweichung	2,438	2,422	2,584	2,275	2,465	2,089	2,194	2,451
Insgesamt	**Mittelwert**	**6,32**	**6,54**	**6,25**	**7,24**	**6,05**	**7,69**	**6,85**	**7,41**
	N	1029	1018	1030	1025	1030	1019	1009	1029
	Standardabweichung	2,491	2,528	2,585	2,379	2,445	2,112	2,246	2,404

10.3. Lebensbedingungen in Innsbruck und Alter

Je älter die Befragten, desto besser werden die Nachbarschaftsbeziehungen eingeschätzt und desto größer die eigene Verbundenheit zur Stadt Innsbruck. Jüngere Befragte (Gruppe der 18 bis 40-Jährigen) schätzen hingegen die Sicherheit und die Luftqualität in der Stadt Innsbruck deutlich höher ein als die beiden älteren Altersgruppen:

Abbildung 58: Lebensbedingungen und Alter (gruppiert) im Mittelwertvergleich

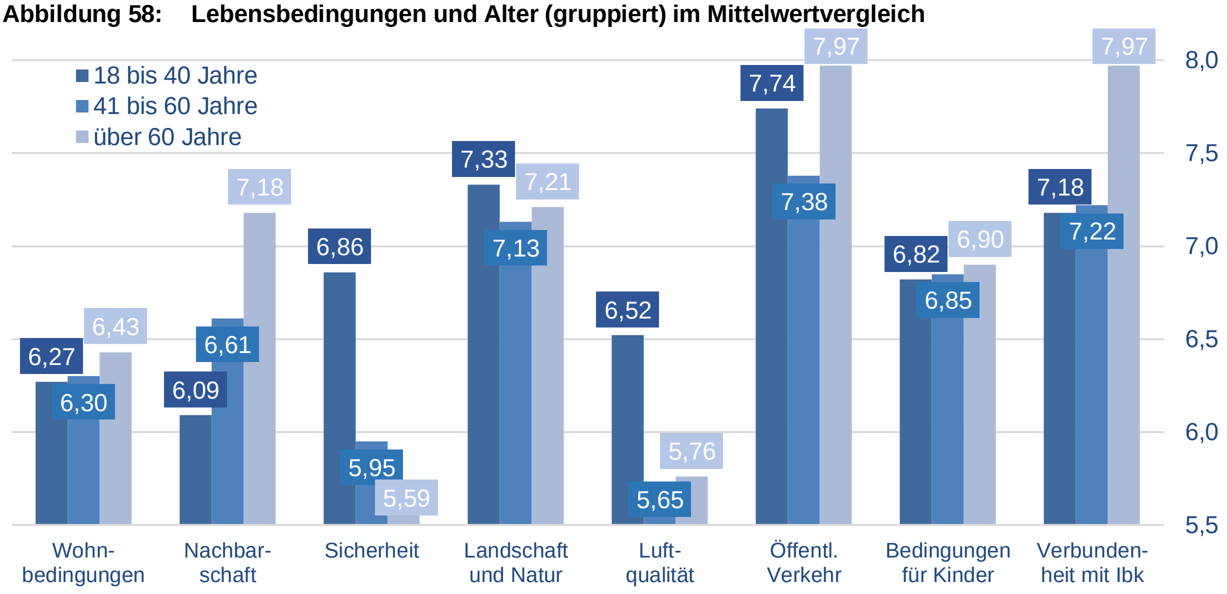

10.4. Lebensbedingungen in Innsbruck und Bildung

Befragte mit Pflichtschulabschluss schätzen die Nachbarschaftsbeziehungen, die Luftqualität und die Güte des öffentlichen Verkehrsnetzes in Innsbruck höher ein als die Befragten der sekundären und tertiären Bildungsschicht:[8]

Abbildung 59: Lebensbedingungen und höchster Schulabschluss (gruppiert) im Mittelwertvergleich

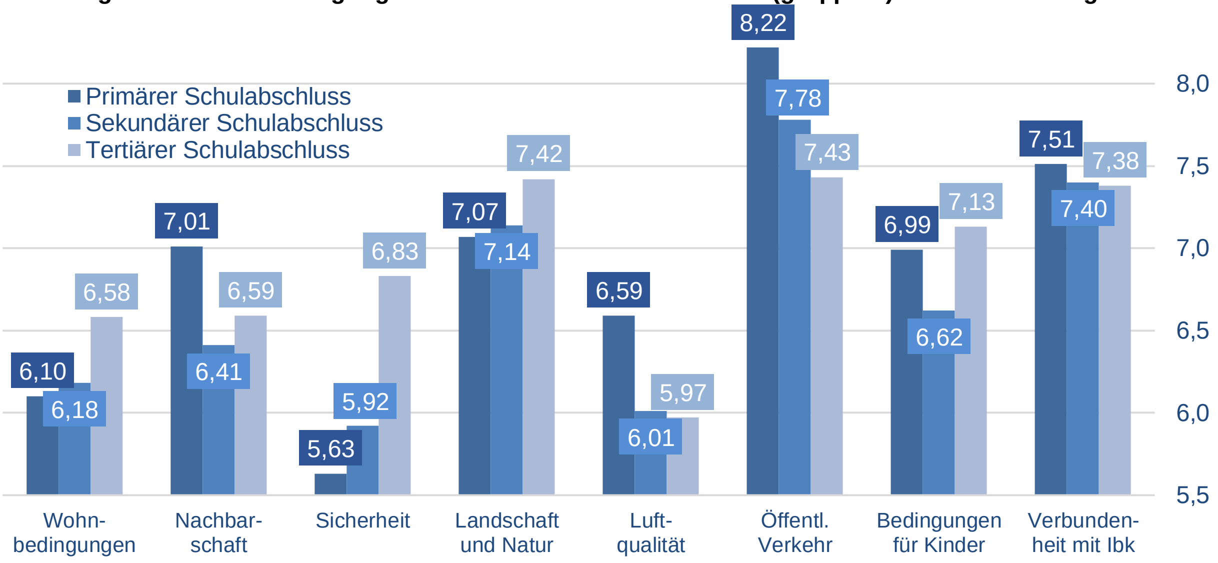

Insgesamt zeigt die mittlere Bildungsschicht im Summenscore die niedrigsten Einschätzungen der Güte der Lebensbedingungen in Innsbruck.

[8] Anm.: Altersbereinigt relativieren sich diese Ausprägungen, bleiben jedoch erhalten.

10.5. Lebensbedingungen in Innsbruck und Gesundheitszustand

Befragte, die ihren Gesundheitszustand als nicht gut beschreiben, schätzen die Lebensbedingungen in Innsbruck – mit Ausnahme der Nachbarschaftsbeziehungen, der Verbundenheit zur Stadt Innsbruck und der Güte des öffentlichen Verkehrs – merklich schlechter ein, als Personen mit zufriedenstellendem oder sehr gutem Gesundheitszustand:

Abbildung 60: Lebensbedingungen und Gesundheitszustand (gruppiert) im Mittelwertvergleich

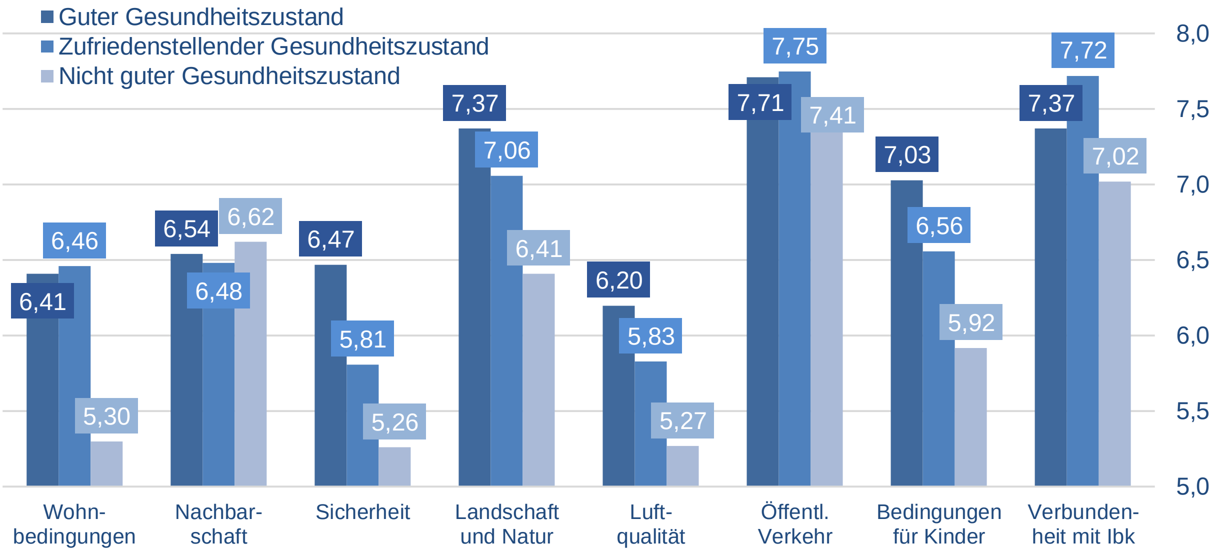

Im Mittel beträgt der Unterschied zu denjenigen Befragten, die von einem sehr guten eigenen Gesundheitszustand ausgehen mehr als eine Antwortkategorie (von insgesamt 11).

10.6. Lebensbedingungen in Innsbruck und Lärmempfindlichkeit

Je stärker eine Person sich selbst als lärmempfindlich einstuft, desto geringer bewertet sie über alle Fragen hinweg die Güte der Lebensbedingungen in Innsbruck – mit 2 Ausnahmen: Die Verbundenheit zur Stadt Innsbruck und die Güte des öffentlichen Verkehrs stehen in keinem deutlichen Zusammenhang zur Lärmempfindlichkeit und werden nicht signifikant niederer bewertet als bei den durchschnittlich bzw. nicht lärmempfindlichen Befragten:

Tabelle 56: Lebensbedingungen und Lärmempfindlichkeit im Mittelwertvergleich

Lärmempfindlichkeit (gruppiert)		Wohnbedingungen	Nachbarschaftsbeziehungen	Sicherheit	Landschaft und Natur	Luftqualität	Öffentl. Verkehr	Bedingungen für Kinder	Verbundenheit zu Innsbruck
**Nicht lärmempfindlich**	**Mittelwert**	**6,60**	**6,82**	**6,42**	**7,34**	**6,69**	**8,03**	**7,14**	**7,67**
	N	275	272	274	275	275	272	271	274
	Standardabweichung	2,743	2,648	2,641	2,496	2,395	2,051	2,416	2,397
**Durchschnittlich lärmempfindlich**	**Mittelwert**	**6,29**	**6,37**	**6,23**	**7,28**	**5,90**	**7,50**	**6,78**	**7,28**
	N	582	577	584	578	583	579	570	583
	Standardabweichung	2,313	2,451	2,522	2,227	2,334	2,149	2,102	2,344
**Sehr lärmempfindlich**	**Mittelwert**	**5,98**	**6,67**	**6,01**	**6,92**	**5,52**	**7,83**	**6,64**	**7,40**
	N	172	169	172	172	172	168	168	172

	Standardabweichung	2,612	2,556	2,700	2,654	2,687	2,015	2,396	2,590
	**Mittelwert**	**6,32**	**6,54**	**6,25**	**7,24**	**6,05**	**7,69**	**6,85**	**7,41**
**Insgesamt**	N	1029	1018	1030	1025	1030	1019	1009	1029
	Standardabweichung	2,491	2,528	2,585	2,379	2,445	2,112	2,246	2,404

10.7. Lebensbedingungen in Innsbruck und Lebensqualität

Befragte, die ihre persönliche Lebensqualität als *nicht gut* einschätzen, beurteilen sämtliche Fragen zu den Lebensbedingungen in Innsbruck sehr deutlich unter dem Mittelwert aller anderen Befragten.

Ganz besonders ausgeprägt sind die Unterschiede im Antwortverhalten zwischen Probandinnen und Probanden mit sehr hoher Selbsteinschätzung der eigenen Lebensqualität und jenen mit sehr geringer persönlicher Lebensqualität bei den Fragen nach der Güte der Wohnbedingungen und dem Ausmaß der Verbundenheit mit der Stadt Innsbruck:

Abbildung 61: Lebensbedingungen und persönl. Lebensqualität (gruppiert) im Mittelwertvergleich

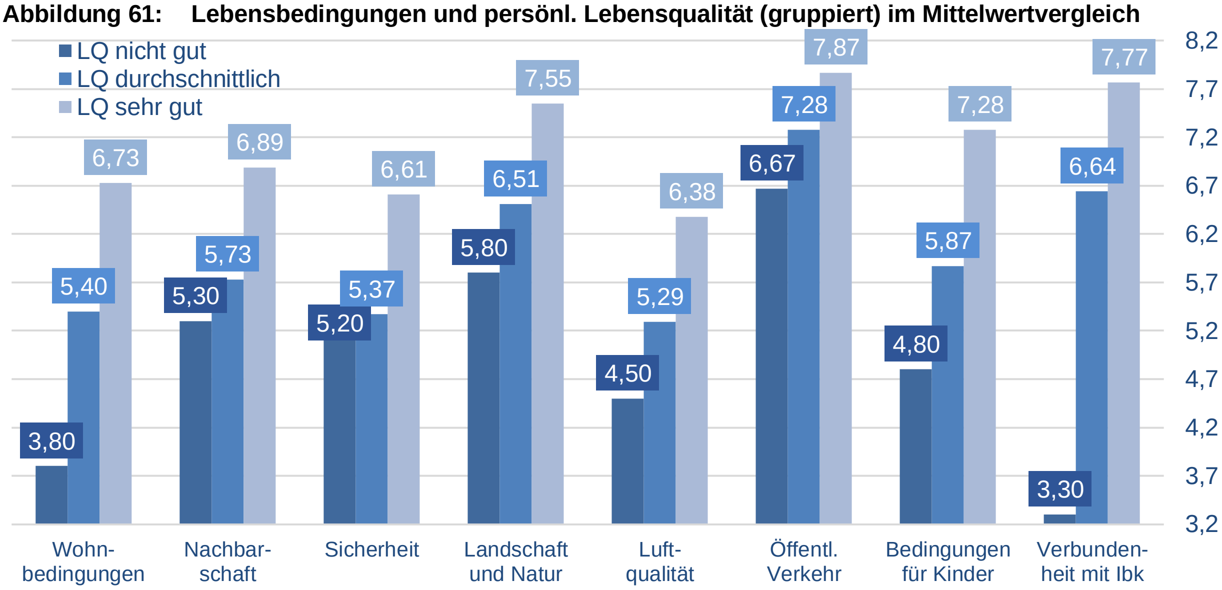

10.8. Lebensbedingungen in Innsbruck und Verkehrslärmbelastung

10.8.1. Straßenverkehrslärmbelastung

Ein Zusammenhang zwischen der Straßenlärmbelastung und der Einschätzung der eigenen Lebensbedingungen ist nachweisbar. Vom 24h-Straßenverkehrslärm gering belastete Befragte schätzen die Lebensbedingungen in Innsbruck deutlich höher ein, als mittelgradig oder stark belastete Probandinnen und Probanden. Ausnahmen bilden die Fragen nach der Güte des öffentlichen Verkehrs und der Verbundenheit zur Stadt Innsbruck:

Abbildung 62: Lebensbedingungen und 24h-Straßenverkehrslärmbelastung L_{den} (gruppiert) im Mittelwertvergleich

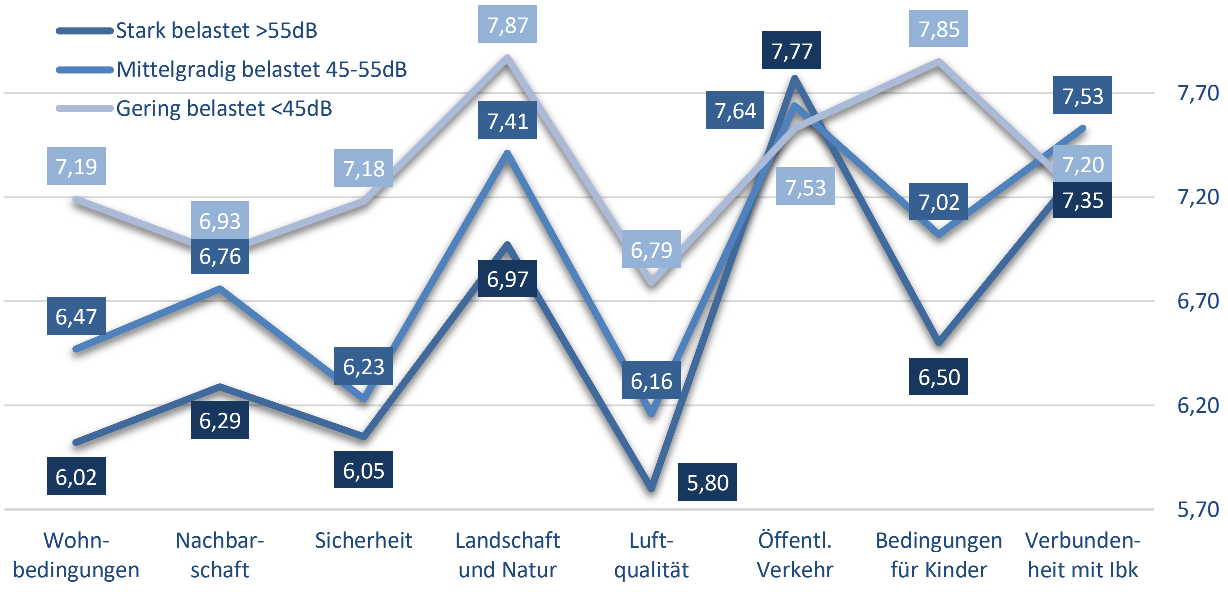

10.8.2. *Schienenverkehrslärmbelastung*

Das Belastungsausmaß durch Schienenverkehrslärm zeigt in Zusammenschau mit der Einschätzung der Lebensbedingungen in Innsbruck keine bis äußerst geringfügige Zusammenhänge. Dies ist neben den statistischen Korrelationsauswertungen auch aus dem Mittelwertvergleich der drei eingeteilten Belastungsgruppen ersichtlich:

Tabelle 57: Lebensbedingungen und 24h-Schienenlärmbelastung L_{den} im Mittelwertvergleich

Schienenlärm 24h gruppiert		Wohnbe-dingungen	Nachbar-schafts-beziehungen	Sicher-heit	Landschaft und Natur	Luft-qualität	Öffentl. Verkehr	Bedingun-gen für Kinder	Verbun-denheit zu Innsbruck
**Stark belastet >55dB**	**Mittelwert**	**6,00**	**6,43**	**6,33**	**7,22**	**5,56**	**7,16**	**6,75**	**7,43**
	N	115	116	116	116	115	115	114	116
	Standardabweichung	2,399	2,544	2,470	2,321	2,535	2,334	2,221	2,389
**Mittelgradig belastet 45-55dB**	**Mittelwert**	**6,48**	**6,40**	**6,02**	**7,17**	**6,21**	**7,67**	**6,69**	**7,36**
	N	320	317	320	320	320	319	318	320
	Standardabweichung	2,555	2,554	2,773	2,536	2,459	2,053	2,383	2,446
**Gering belastet <45dB**	**Mittelwert**	**6,30**	**6,64**	**6,35**	**7,27**	**6,06**	**7,81**	**6,95**	**7,42**
	N	594	585	594	589	595	585	577	593
	Standardabweichung	2,472	2,510	2,497	2,304	2,411	2,085	2,169	2,388
**Insgesamt**	**Mittelwert**	**6,32**	**6,54**	**6,25**	**7,24**	**6,05**	**7,69**	**6,85**	**7,41**
	N	1029	1018	1030	1025	1030	1019	1009	1029
	Standardabweichung	2,491	2,528	2,585	2,379	2,445	2,112	2,246	2,404

10.8.3. *Flugverkehrslärmbelastung*

Auch die 24h-Fluglärmbelastung zeigt im Gegensatz zur 24h-Straßenverkehrslärmbelastung keine eindeutigen Zusammenhänge mit der Einschätzung der Lebensbedingungen in Innsbruck auf:

Tabelle 58: Lebensbedingungen und 24h-Fluglärmbelastung L_{den} im Mittelwertvergleich

Fluglärm 24h gruppiert		Wohnbe-dingungen	Nachbar-schafts-beziehungen	Sicher-heit	Landschaft und Natur	Luft-qualität	Öffentl. Verkehr	Bedingun-gen für Kinder	Verbun-denheit zu Innsbruck
**Stark belastet >55dB**	**Mittelwert**	**6,30**	**6,76**	**6,50**	**7,18**	**5,95**	**7,87**	**6,97**	**7,68**
	N	110	109	112	111	112	111	103	112
	Standardabweichung	2,463	2,592	2,394	2,200	2,201	1,903	2,007	2,428
**Mittelgradig belastet 45-55dB**	**Mittelwert**	**5,93**	**6,21**	**6,06**	**6,88**	**5,67**	**7,51**	**6,35**	**7,29**
	N	360	356	359	357	359	352	351	360
	Standardabweichung	2,574	2,624	2,661	2,494	2,432	2,252	2,343	2,417
**Gering belastet <45dB**	**Mittelwert**	**6,58**	**6,71**	**6,31**	**7,47**	**6,31**	**7,78**	**7,14**	**7,42**
	N	559	553	559	557	559	556	555	557
	Standardabweichung	2,413	2,434	2,569	2,312	2,470	2,055	2,173	2,390
**Insgesamt**	**Mittelwert**	**6,32**	**6,54**	**6,25**	**7,24**	**6,05**	**7,69**	**6,85**	**7,41**
	N	1029	1018	1030	1025	1030	1019	1009	1029
	Standardabweichung	2,491	2,528	2,585	2,379	2,445	2,112	2,246	2,404

10.9. Lebensbedingungen in Innsbruck und Lärmexpositionsempfinden

10.9.1. *Lärmexpositionsempfinden insgesamt*

Je stärker sich Befragte dem Lärm insgesamt ausgesetzt sehen, desto geringer werden die Lebens-bedingungen in Innsbruck eingeschätzt:

Abbildung 63: Lebensbedingungen und Lärmexpositionsempfinden insgesamt (gruppiert) im Mittelwertvergleich

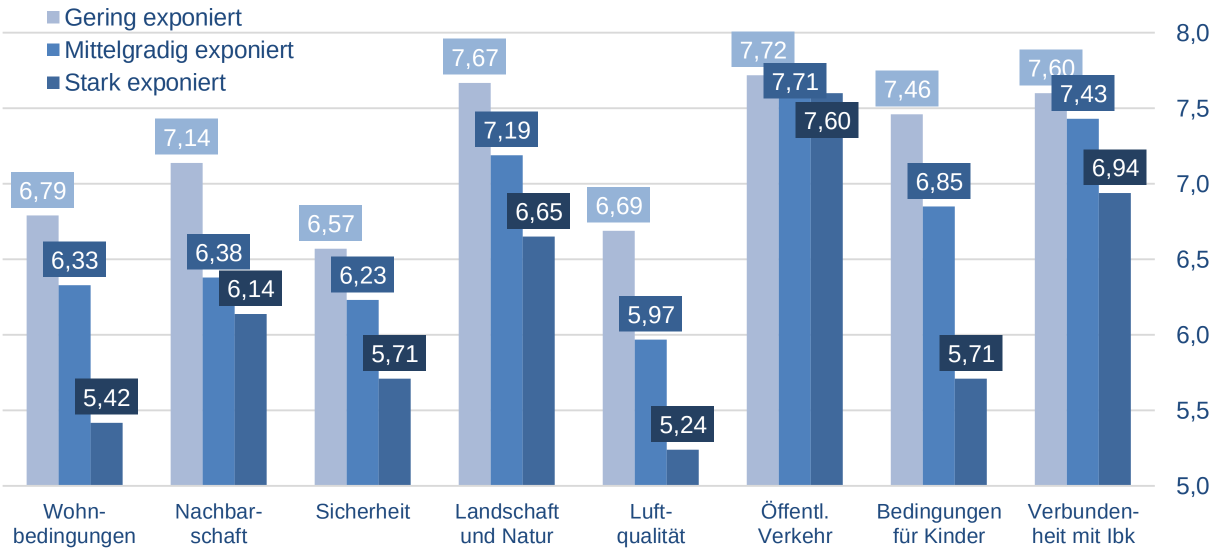

Wiederum bleibt die Frage nach der Güte der öffentlichen Verkehrsverbindungen davon weitestgehend unberührt.

10.9.2. *Straßenlärmexpositionsempfinden*

Ein sehr ähnliches Bild wie in der Zusammenschau mit dem Lärmexpositionsempfinden insgesamt zeigt sich bei der Zuordnung der Einschätzung der Ausgesetztheit der eigenen Wohnung / des eigenen Hauses gegenüber Straßenverkehrslärm, wenn auch etwas geringer ausgeprägt:

Tabelle 59: Lebensbedingungen und Straßenlärmexpositionsempfinden im Mittelwertvergleich

Ausgesetztheit Strassenlärm gruppiert		Wohnbedingungen	Nachbarschaftsbeziehungen	Sicherheit	Landschaft und Natur	Luftqualität	Öffentl. Verkehr	Bedingungen für Kinder	Verbundenheit zu Innsbruck
**Gering exponiert**	**Mittelwert**	**6,70**	**6,80**	**6,55**	**7,47**	**6,55**	**7,70**	**7,25**	**7,46**
	N	429	423	429	424	430	427	421	428
	Standardabweichung	2,496	2,478	2,456	2,353	2,394	2,203	2,199	2,404
**Mittelgradig exponiert**	**Mittelwert**	**6,19**	**6,45**	**6,19**	**7,18**	**5,82**	**7,67**	**6,76**	**7,44**
	N	463	459	464	464	463	456	451	464
	Standardabweichung	2,427	2,478	2,499	2,263	2,348	2,021	2,093	2,251
**Stark exponiert**	**Mittelwert**	**5,57**	**6,04**	**5,47**	**6,69**	**5,23**	**7,76**	**5,93**	**7,12**
	N	137	136	137	137	137	136	137	137
	Standardabweichung	2,494	2,761	3,066	2,735	2,598	2,134	2,563	2,861
**Insgesamt**	**Mittelwert**	**6,32**	**6,54**	**6,25**	**7,24**	**6,05**	**7,69**	**6,85**	**7,41**
	N	1029	1018	1030	1025	1030	1019	1009	1029
	Standardabweichung	2,491	2,528	2,585	2,379	2,445	2,112	2,246	2,404

10.9.3. *Fluglärmexpositionsempfinden*

Abbildung 64: Lebensbedingungen und Fluglärmexpositionsempfinden (gruppiert) im Mittelwertvergleich

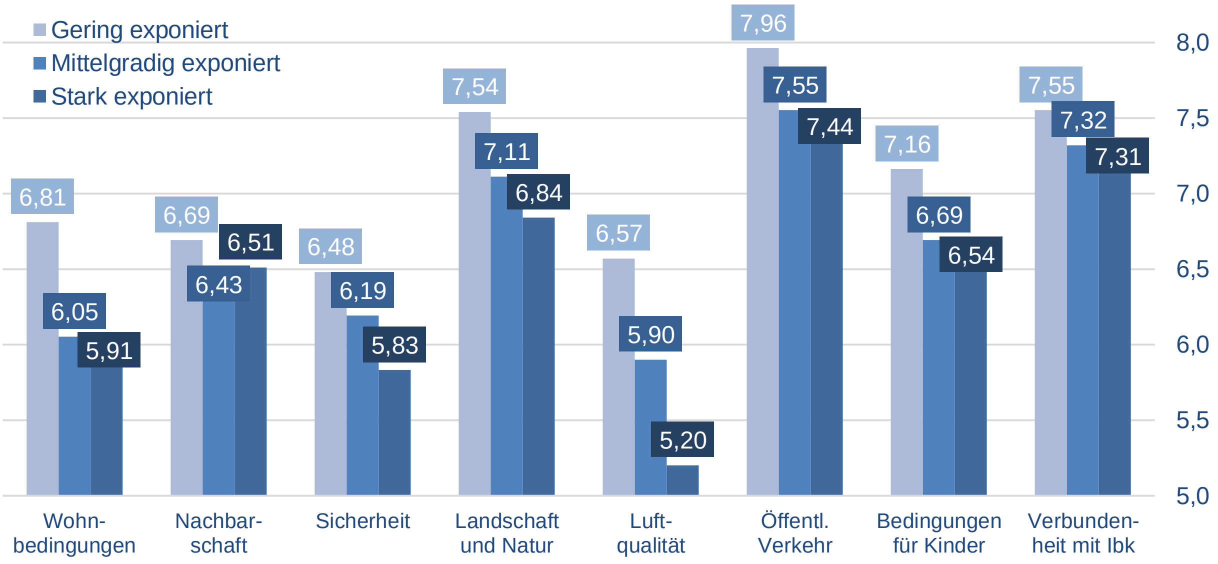

Auch die Einschätzung der eigenen Exposition durch Flugverkehrslärm weist in der Kombination mit der Beurteilung der Güte der Lebensbedingungen in Innsbruck Zusammenhänge auf. Besonders die Wohnbedingungen und die Luftqualität werden von den sich selbst dem Fluglärm stärker ausgesetzt sehenden Befragten niederer eingeschätzt, als von sich weniger exponiert empfindenden Probandinnen und Probanden.

Im Gegensatz zu den Auswertungen bezogen auf die real vorhandene Belastung durch Flugverkehrslärm, zeigt die persönliche Einschätzung der eigenen Ausgesetztheit deutliche Unterschiede in der Bewertung der Lebensbedingungen in Innsbruck auf.

10.9.4. Schienenverkehrslärmexpositionsempfinden

Das Empfinden der Ausgesetztheit gegenüber Schienenverkehrslärm zeigt in der Zusammenschau mit der Beurteilung der Lebensbedingungen in Innsbruck vergleichsweise geringere Zusammenhänge:

Abbildung 65: Lebensbedingungen und Schienenverkehrslärmexpositionsempfinden (gruppiert) im Mittelwertvergleich

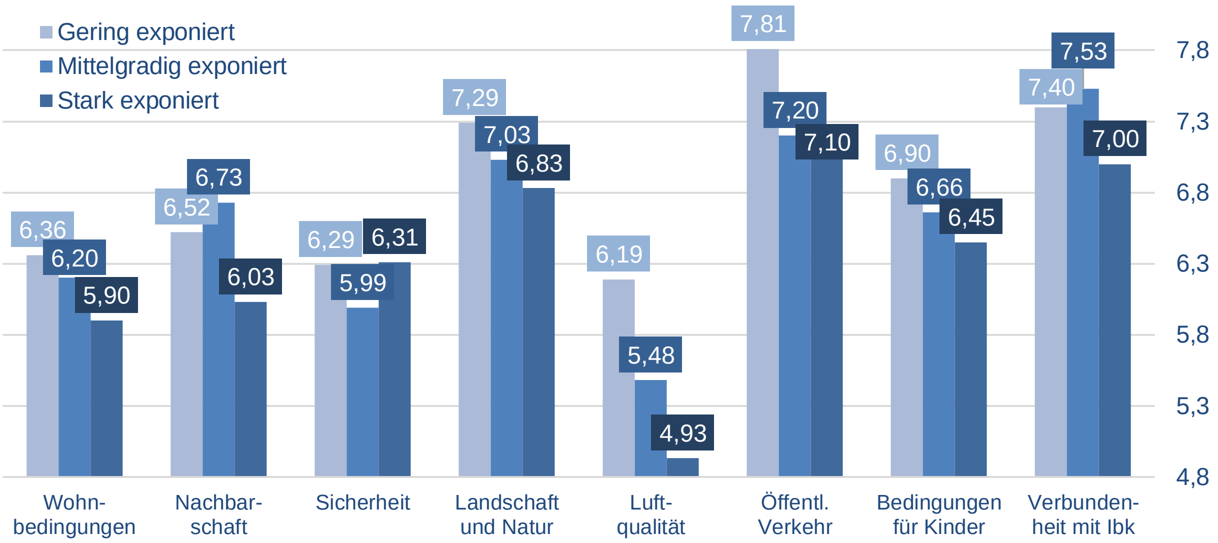

Sich stärker dem Schienenverkehrslärm exponiert fühlenden Befragte beurteilen die Lebensbedingungen insgesamt aber auch in dieser Kombination schlechter als die sich weniger stark exponiert Fühlenden. Am stärksten ausgeprägt sind die Unterschiede bei der Frage nach der Innsbrucker Luftqualität.

10.10. Lebensbedingungen in Innsbruck und Lärmbelästigung

10.10.1. Lärmbelästigung insgesamt

Die Lebensbedingungen in Innsbruck werden von Befragten, welche sich durch den Lärm insgesamt stärker gestört fühlen, deutlich schlechter bewertet, als von Personen, die sich weniger lärmbelästigt fühlen:

Abbildung 66: Lebensbedingungen und Lärmbelästigung insgesamt (gruppiert) im Mittelwertvergleich

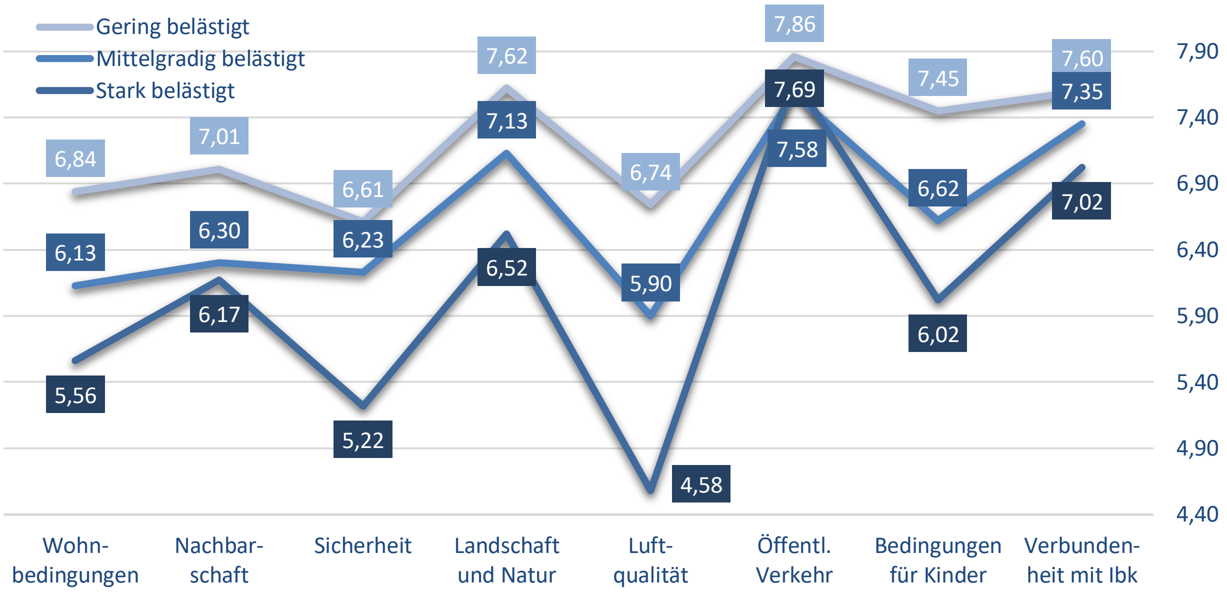

Wie schon bei der Zusammenschau mit den Variablen *vorhandene Lärmbelastung* und *Lärmexpositionsempfinden* bleibt die Bewertung der Güte der öffentlichen Verkehrsverbindungen auch von der Einschätzung der Lärmbelästigung insgesamt weitestgehend unberührt.

10.10.2. Straßenverkehrslärmbelästigung

Dasselbe Bild wie bei der Lärmbelästigung insgesamt, im Vergleich etwas weniger ausgeprägt, zeigt die Zusammenschau der Straßenverkehrslärmbelästigung mit der Einschätzung der Lebensbedingungen:

Abbildung 67: Lebensbedingungen und Straßenlärmbelästigung (gruppiert) im Mittelwertvergleich

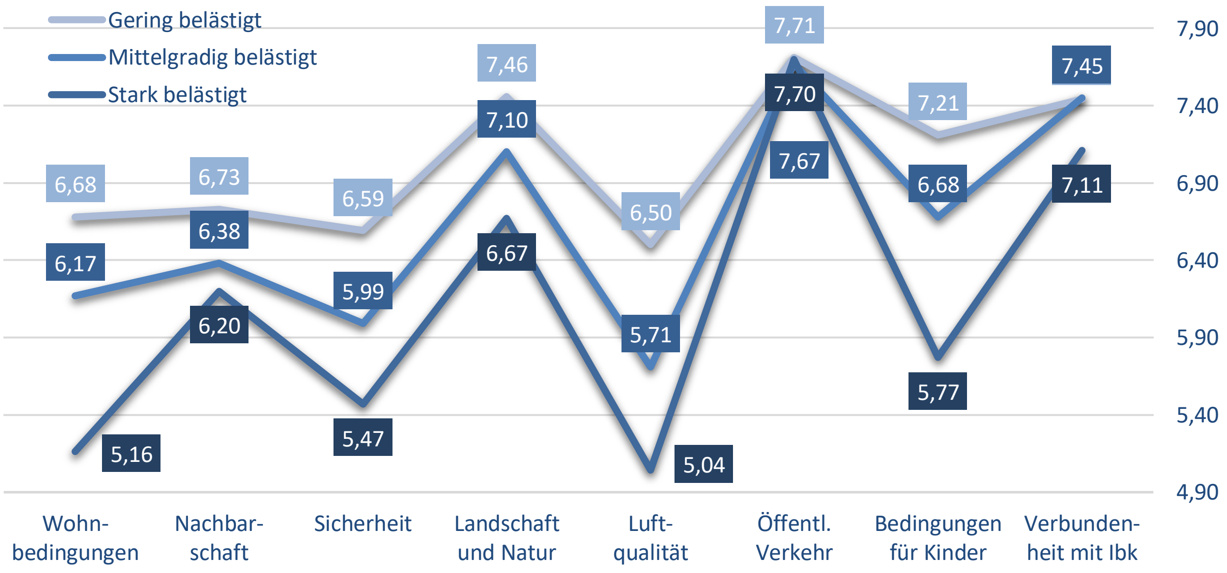

10.10.3. *Flugverkehrslärmbelästigung*

Sehr ähnlich wie bei der Lärmbelästigung insgesamt und der Straßenverkehrslärmbelästigung sinkt die Einschätzung der Lebensbedingungen auch in Zusammenschau mit der Flugverkehrslärmbelästigung bei steigender Belästigung stetig und vice versa:

Abbildung 68: Lebensbedingungen und Fluglärmbelästigung (gruppiert) im Mittelwertvergleich

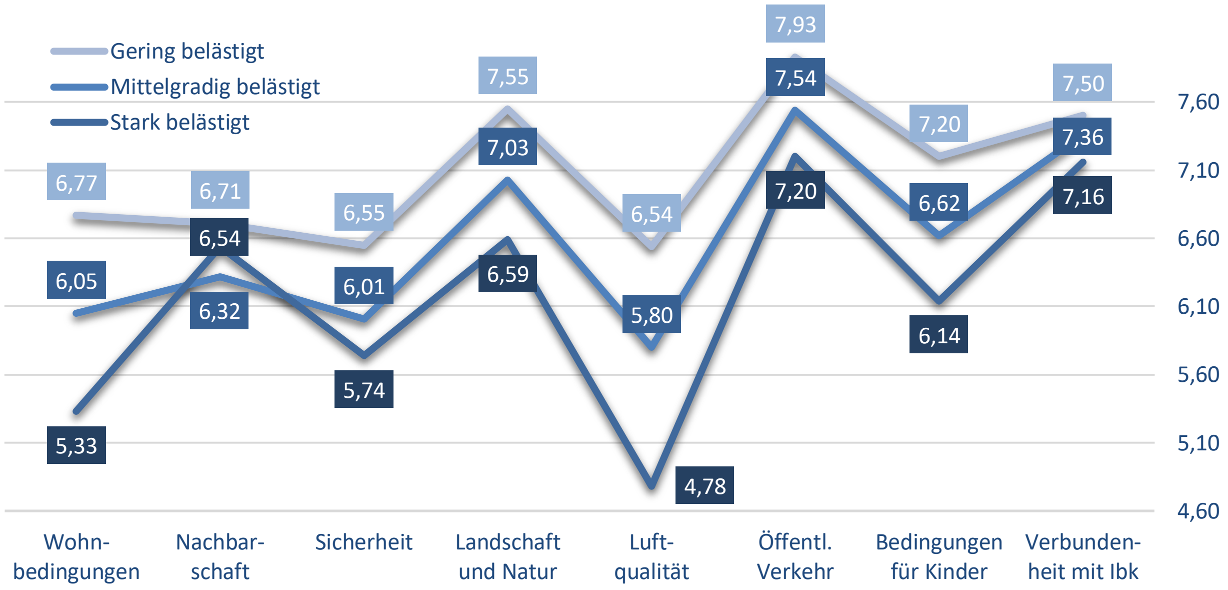

10.10.4. *Schienenverkehrslärmbelästigung*

Vom Schienenverkehrslärm stärker belästigte Befragte beurteilen die Lebensbedingungen in Innsbruck ebenfalls schlechter als sich weniger stark gestört fühlende Personen. Die Unterschiede sind allerdings wesentlich geringer ausgeprägt als in Zusammenschau mit der Lärmbelästigung insgesamt, der Straßen- und der Flugverkehrslärmbelästigung und statistisch nicht eindeutig (eine Ausnahme bildet dabei die Frage nach der Güte der Wohnbedingungen):

Abbildung 69: Lebensbedingungen und Schienenlärmbelästigung (gruppiert) im Mittelwertvergleich

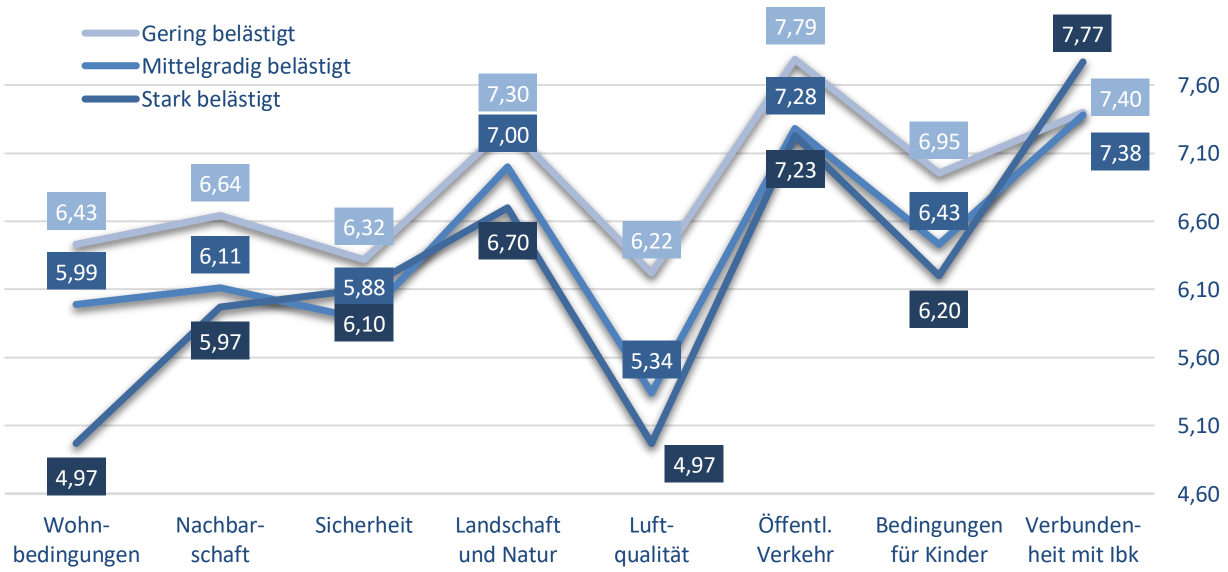

10.10.5. *Nicht verkehrsverursachte Lärmbelästigung*

Auch die sich von nicht verkehrsverursachten Lärm stärker gestört fühlenden Befragten schätzen die Lebensbedingungen in Innsbruck schlechter ein, als dies sich weniger lärmbelästigt empfindende Personen tun. Im Vergleich zu den verkehrsverursachten Belästigungsreaktionen fallen diese jedoch etwas geringer aus:

Tabelle 60: Lebensbedingungen und Belästigung durch Nachbarschaftslärm im Mittelwertvergleich

Belästigung Nachbarschaftslärm gruppiert		Wohnbe-dingungen	Nachbar-schafts-beziehungen	Sicher-heit	Landschaft und Natur	Luft-qualität	Öffentl. Verkehr	Bedingun-gen für Kinder	Verbun-denheit zu Innsbruck
**Gering belästigt**	**Mittelwert**	**6,50**	**6,90**	**6,42**	**7,43**	**6,26**	**7,77**	**7,08**	**7,51**
	N	734	725	735	732	735	727	718	735
	Standardabweichung	2,468	2,476	2,512	2,269	2,373	2,118	2,128	2,319
**Mittelgradig belästigt**	**Mittelwert**	**6,02**	**5,77**	**6,02**	**6,93**	**5,65**	**7,46**	**6,44**	**7,25**
	N	243	242	243	241	243	241	239	242
	Standardabweichung	2,393	2,383	2,558	2,429	2,491	2,129	2,320	2,466
**Stark belästigt**	**Mittelwert**	**5,25**	**5,08**	**4,90**	**5,88**	**4,98**	**7,75**	**5,52**	**6,63**
	N	52	51	52	52	52	51	52	52
	Standardabweichung	2,889	2,629	3,231	3,033	2,747	1,896	2,747	3,074
**Insgesamt**	**Mittelwert**	**6,32**	**6,54**	**6,25**	**7,24**	**6,05**	**7,69**	**6,85**	**7,41**
	N	1029	1018	1030	1025	1030	1019	1009	1029
	Standardabweichung	2,491	2,528	2,585	2,379	2,445	2,112	2,246	2,404

Tabelle 61: Lebensbedingungen und Belästigung durch Baustellenlärm im Mittelwertvergleich

Belästigung Baustellenlärm gruppiert		Wohnbe-dingungen	Nachbar-schafts-beziehungen	Sicher-heit	Landschaft und Natur	Luft-qualität	Öffentl. Verkehr	Bedingun-gen für Kinder	Verbunden-heit zu Innsbruck
**Gering belästigt**	**Mittelwert**	**6,46**	**6,71**	**6,41**	**7,35**	**6,19**	**7,75**	**7,11**	**7,52**
	N	669	660	669	664	670	661	653	668
	Standardabweichung	2,500	2,512	2,514	2,284	2,441	2,066	2,094	2,339
**Mittelgradig belästigt**	**Mittelwert**	**6,23**	**6,17**	**6,02**	**7,06**	**5,91**	**7,61**	**6,59**	**7,17**
	N	263	261	264	264	263	262	259	264
	Standardabweichung	2,375	2,565	2,550	2,538	2,365	2,199	2,298	2,431
**Stark belästigt**	**Mittelwert**	**5,59**	**6,34**	**5,69**	**6,90**	**5,44**	**7,56**	**5,78**	**7,22**
	N	96	96	96	96	96	95	96	96
	Standardabweichung	2,634	2,436	3,034	2,544	2,607	2,187	2,696	2,731
**Insgesamt**	**Mittelwert**	**6,32**	**6,54**	**6,25**	**7,24**	**6,05**	**7,69**	**6,85**	**7,41**
	N	1029	1018	1030	1025	1030	1019	1009	1029
	Standardabweichung	2,491	2,528	2,585	2,379	2,445	2,112	2,246	2,404

Tabelle 62: Lebensbedingungen und Belästigung durch Lärm von Passanten und Gaststätten im Mittelwertvergleich

Belästigung Lärm von Passanten und Gaststätten gruppiert		Wohnbedingungen	Nachbarschaftsbeziehungen	Sicherheit	Landschaft und Natur	Luftqualität	Öffentl. Verkehr	Bedingungen für Kinder	Verbundenheit zu Innsbruck
**Gering belästigt**	**Mittelwert**	**6,57**	**6,75**	**6,46**	**7,39**	**6,21**	**7,68**	**7,13**	**7,44**
	N	746	737	747	742	747	740	730	746
	Standardabweichung	2,403	2,486	2,498	2,293	2,456	2,162	2,111	2,352
**Mittelgradig belästigt**	**Mittelwert**	**5,69**	**5,95**	**5,71**	**6,83**	**5,62**	**7,74**	**6,18**	**7,38**
	N	233	231	233	233	233	231	230	233
	Standardabweichung	2,550	2,560	2,591	2,489	2,368	1,925	2,314	2,363
**Stark belästigt**	**Mittelwert**	**5,54**	**6,24**	**5,46**	**6,90**	**5,70**	**7,73**	**5,82**	**6,94**
	N	50	50	50	50	50	48	49	50
	Standardabweichung	2,859	2,584	3,290	2,859	2,401	2,238	2,870	3,235
**Insgesamt**	**Mittelwert**	**6,32**	**6,54**	**6,25**	**7,24**	**6,05**	**7,69**	**6,85**	**7,41**
	N	1029	1018	1030	1025	1030	1019	1009	1029
	Standardabweichung	2,491	2,528	2,585	2,379	2,445	2,112	2,246	2,404

Tabelle 63: Lebensbedingungen und Belästigung durch Erschütterungen im Mittelwertvergleich

Belästigung Erschütterungen gesamt gruppiert		Wohnbedingungen	Nachbarschaftsbeziehungen	Sicherheit	Landschaft und Natur	Luftqualität	Öffentl. Verkehr	Bedingungen für Kinder	Verbundenheit zu Innsbruck
**Gering belästigt**	**Mittelwert**	**6,52**	**6,71**	**6,38**	**7,39**	**6,21**	**7,76**	**7,05**	**7,46**
	N	860	850	861	856	862	854	843	860
	Standardabweichung	2,443	2,457	2,502	2,303	2,423	2,072	2,132	2,326
**Mittelgradig belästigt**	**Mittelwert**	**5,38**	**5,78**	**5,61**	**6,47**	**5,31**	**7,34**	**5,97**	**7,28**
	N	135	134	135	135	135	131	133	135
	Standardabweichung	2,473	2,565	2,844	2,518	2,339	2,190	2,383	2,541
**Stark belästigt**	**Mittelwert**	**5,09**	**5,38**	**5,24**	**6,47**	**4,91**	**7,38**	**5,21**	**6,47**
	N	34	34	34	34	33	34	33	34
	Standardabweichung	2,621	3,266	3,026	2,967	2,650	2,663	2,955	3,457
**Insgesamt**	**Mittelwert**	**6,32**	**6,54**	**6,25**	**7,24**	**6,05**	**7,69**	**6,85**	**7,41**
	N	1029	1018	1030	1025	1030	1019	1009	1029
	Standardabweichung	2,491	2,528	2,585	2,379	2,445	2,112	2,246	2,404

Tabelle 64: Lebensbedingungen und Belästigung durch Lärm aus Gewerbe und Industrie im Mittelwertvergleich

Belästigung Lärm aus Gewerbe u. Industrie gruppiert		Wohnbedingungen	Nachbarschaftsbeziehungen	Sicherheit	Landschaft und Natur	Luftqualität	Öffentl. Verkehr	Bedingungen für Kinder	Verbundenheit zu Innsbruck
**Gering belästigt**	**Mittelwert**	**6,36**	**6,59**	**6,32**	**7,30**	**6,15**	**7,73**	**6,96**	**7,46**
	N	935	924	936	931	936	927	917	935
	Standardabweichung	2,497	2,523	2,591	2,384	2,430	2,119	2,214	2,394
**Mittelgradig belästigt**	**Mittelwert**	**5,96**	**6,03**	**5,53**	**6,52**	**5,14**	**7,21**	**5,71**	**6,82**
	N	79	79	79	79	79	77	77	79
	Standardabweichung	2,377	2,567	2,352	2,148	2,313	2,015	2,288	2,336
**Stark belästigt**	**Mittelwert**	**5,73**	**6,13**	**5,47**	**6,87**	**4,27**	**8,13**	**5,73**	**7,13**
	N	15	15	15	15	15	15	15	15
	Standardabweichung	2,685	2,446	2,825	2,722	2,492	1,959	2,154	3,067
**Insgesamt**	**Mittelwert**	**6,32**	**6,54**	**6,25**	**7,24**	**6,05**	**7,69**	**6,85**	**7,41**
	N	1029	1018	1030	1025	1030	1019	1009	1029
	Standardabweichung	2,491	2,528	2,585	2,379	2,445	2,112	2,246	2,404

10.11. Lebensbedingungen in Innsbruck und weitere Variablen

Für eine Vielzahl weiterer Variablen lassen sich Zusammenhänge zur Einschätzung der Lebens- und Wohnbedingungen in Innsbruck herstellen. Mit geringerer oder stärkerer Ausprägung zeigen diese zumeist vergleichbare Resultate. Beispielhaft wird an dieser Stelle die Zusammenschau mit der Beurteilung der Lebensbedingungen in Innsbruck und der Frage nach den Schlafgewohnheiten bei offenem / gekipptem oder geschlossenem Fenster dargestellt:

Abbildung 70: Lebensbedingungen nach Nachtschlaf bei offenem oder gekippten Fenster (gruppiert) im Mittelwertvergleich

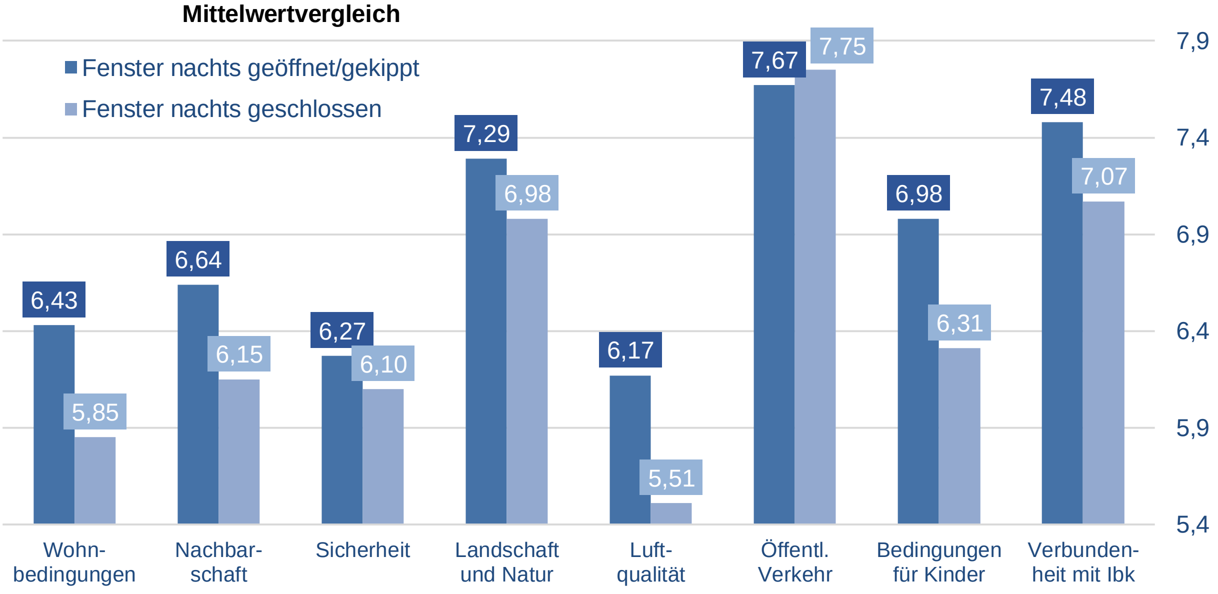

Zufriedenheit mit der eigenen Wohnsituation

Die Zufriedenheit mit der eigenen Wohnsituation insgesamt wurde mit einem zusätzlichen Item abgefragt.

Über 70% aller Befragten sind mit ihrer eigenen Wohnsituation sehr zufrieden und antworten in den drei höchsten Antwortkategorien, 2,7% sind nicht zufrieden und antworten in den drei Untersten. Genau ein Viertel der Antworten verteilt sich auf die fünf mittleren Kategorien. Eine befragte Person gibt auf die Frage nach der Zufriedenheit mit der eigenen Wohnsituation keine Antwort:

Abbildung 71: Zufriedenheit mit der eigenen Wohnsituation in Prozent

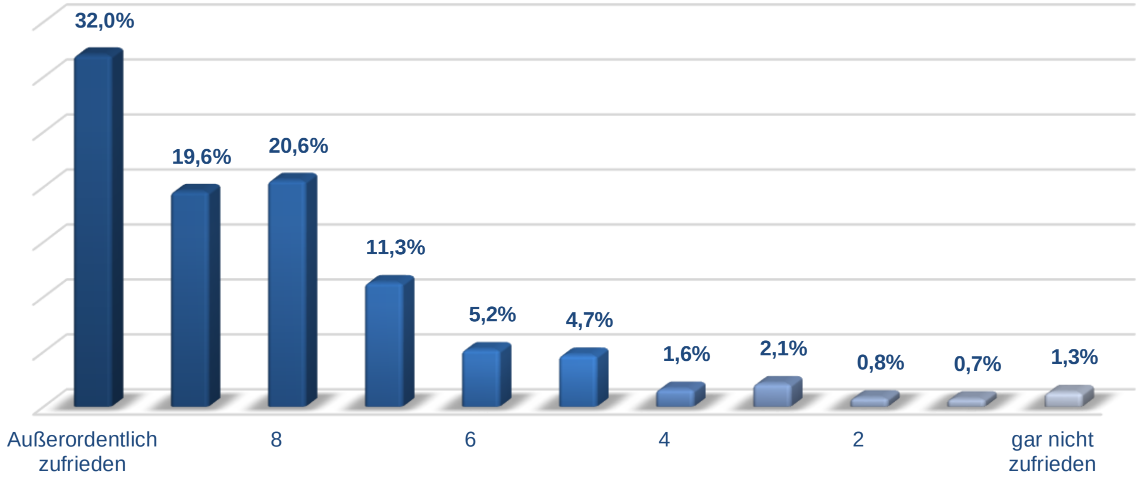

Der Mittelwert der Antworten liegt auf der 11-er Skala von *gar nicht zufrieden* bis *außerordentlich zufrieden* bei 8,11 (Standardfehler 0,07), das entspricht einem durchschnittlichen Zufriedenheitsausmaß mit der eigenen Wohnsituation der befragten Innsbruckerinnen und Innsbrucker von 89,2% vom Maximalwert.

Tabelle 65: Zufriedenheit mit der eigenen Wohnsituation gruppiert

		Häufigkeit	Gültige Prozente	Kumulierte Prozente
Gültig	Nicht zufrieden	28	2,7	2,7
	Durchschnittlich	258	25,0	27,7
	Sehr zufrieden	744	72,2	99,9
	w.n. / k.A.	1	,1	100,0
	Gesamt	1031	100,0	

1. Wohnsituation und Katastralgemeinden

Die Zufriedenheit mit der eigenen Wohnsituation insgesamt unterscheidet sich zwischen den Katastralgemeinden und ist in den äußeren Katastralgemeinden höher als in den inneren Stadtteilen:

Abbildung 72: Zufriedenheit mit der eigenen Wohnsituation und Katastralgemeinde im Mittelwertvergleich (aufsteigend sortiert)

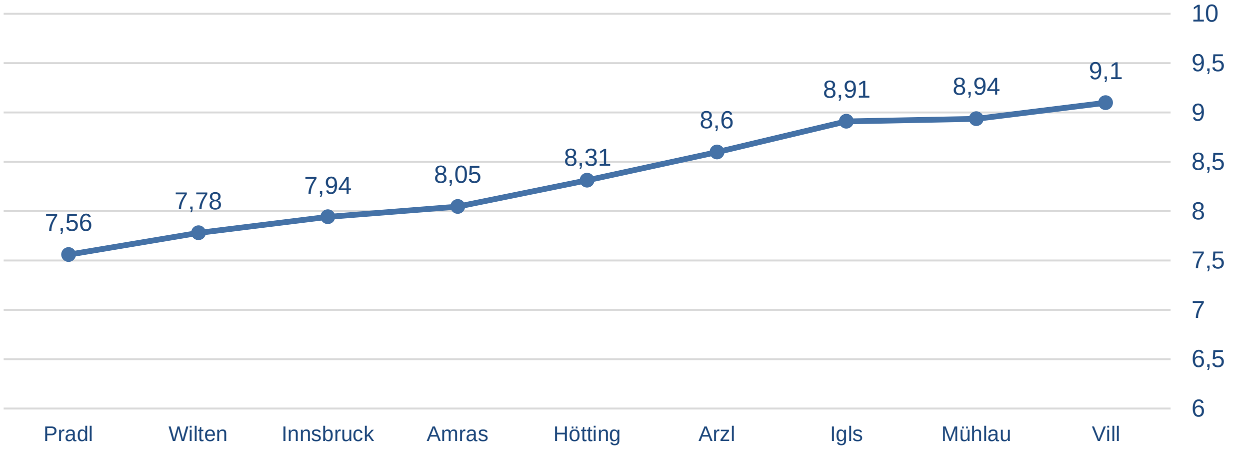

Tabelle 66: Zufriedenheit mit der eigenen Wohnsituation u. Katastralgemeinde im Mittelwertvergleich

Katastralgemeinde	Mittelwert	N	Standardabweichung
Amras	8,05	60	2,354
Arzl	8,60	102	2,079
Hötting	8,31	243	1,920
Igls	8,91	35	,919
Innsbruck	7,94	176	1,938
Mühlau	8,94	50	1,834
Pradl	7,56	188	2,340
Vill	9,10	21	1,136
Wilten	7,78	155	2,297
Insgesamt	8,11	1030	2,105

2. Wohnsituation und ausgewählte Variablen

Im Mittelwertvergleich sind keine Unterschiede in der Zufriedenheit mit der eigenen Wohnsituation zwischen den Geschlechtern erkennbar. Bezogen auf die absoluten Häufigkeiten antworten Männer etwas häufiger in den mittleren Antwortkategorien und Frauen zeigen sich häufiger sehr zufrieden mit ihrer Wohnsituation.

Ein ausgeprägter statistischer Zusammenhang mit dem bisher höchsten erreichten Schulabschluss, dem Vorhandensein minderjähriger Kinder im selben Haushalt, der Kfz-Nutzung, der Güte der Einschätzung des eigenen Gesundheitszustandes, dem Vorhandensein von Lärmschutzfenstern oder baulichen Abschirmungen gegen den Verkehrslärm zeigt sich nicht.

Mit zunehmendem Alter steigt die Zufriedenheit mit den eigenen Wohnbedingungen an.

Eigentümerinnen und Eigentümer ihrer Wohnungen bzw. Wohnhäuser sind deutlich zufriedener mit ihren Wohnverhältnissen, als dies Mieterinnen und Mieter, Mitbewohnerinnen und Mitbewohner und Untermieterinnen und Untermieter sind.

Tabelle 67: Zufriedenheit mit der Wohnsituation und ausgewählte Variablen (gruppiert)

		Ausmaß Zufriedenheit eigene Wohnsituation insgesamt					
		Nicht zufrieden		Durchschnittlich		Sehr zufrieden	
		Anzahl	Zeilen (%)	Anzahl	Zeilen (%)	Anzahl	Zeilen (%)
**Geschlecht**	weiblich	16	3,0%	127	23,5%	397	73,5%
	männlich	12	2,4%	131	26,7%	347	70,8%
**Altersgruppen**	18 bis 40 Jahre	16	3,6%	134	30,5%	289	65,8%
	41 bis 60 Jahre	7	2,2%	81	25,6%	228	72,2%
	über 60 Jahre	5	1,8%	43	15,6%	227	82,5%
**Bildungsschicht**	Primärer Schulabschluss	5	5,1%	19	19,4%	74	75,5%
	Sekundärer Schulabschluss	12	2,2%	144	26,6%	385	71,2%
	Tertiärer Schulabschluss	11	2,8%	94	24,2%	283	72,9%
**Eigentums-verhältnisse**	Eigentümer/in	6	1,4%	63	15,0%	350	83,5%
	Hauptmieter/in	16	3,5%	152	33,6%	285	62,9%
	Mitbewohner/in, Untermieter/in	6	3,8%	43	27,2%	109	69,0%
**Haushalt mit/ ohne mj. Kinder**	Haushalt ohne Kinder	17	2,2%	189	24,8%	555	72,9%
	Haushalt mit Kindern	11	4,1%	69	25,7%	189	70,3%
**Baulicher Schutz vor Verkehrslärm**	Kaum Abschirmung	24	3,1%	193	25,2%	548	71,6%
	Gute Abschirmung	3 (*)	1,3%	53	22,5%	180	76,3%
**Nachtschlaf**	Fenster nachts geöffnet/gekippt	17	2,1%	196	23,9%	607	74,0%
	Fenster nachts geschlossen	11	5,4%	59	29,2%	132	65,3%
**Lärmschutzfenster**	Ja	7	3,7%	48	25,7%	132	70,6%
	Nein	19	2,8%	158	23,5%	494	73,6%
	weiß nicht/ k.A.	2	1,2%	52	30,2%	118	68,6%
**Summenscore Le-bensbedingungen**	Nicht gut (*)	1	20,0%	4	80,0%	0	0,0%
	Durchschnittlich	24	3,6%	217	32,4%	428	64,0%
	Sehr gut	3	0,8%	37	10,4%	316	88,8%
**Lebensqualität**	Nicht gut (*)	2	20,0%	5	50,0%	3	30,0%
	Durchschnittlich	21	7,2%	149	51,4%	120	41,4%
	Sehr gut	5	0,7%	102	14,1%	617	85,2%
**Lärmempfindlichkeit**	Nicht lärmempfindlich	6	2,2%	54	19,7%	214	78,1%
	Durchschnittlich	14	2,4%	160	27,4%	410	70,2%
	Sehr lärmempfindlich	8	4,7%	44	25,6%	120	69,8%
**Gesundheitszustand**	Gut	20	2,7%	183	24,3%	550	73,0%
	Zufriedenstellend	5	2,7%	51	27,4%	130	69,9%
	Nicht gut	3 (*)	3,4%	24	27,0%	62	69,7%

(*) Zu geringe Fallzahlen für solide Auswertungen

Deutlich treten auch Zusammenhänge zwischen der Einschätzung der Lebensbedingungen in Innsbruck, der eigenen Lebensqualität und der eigenen Lärmempfindlichkeit hervor:

Abbildung 73: Anteilige Zufriedenheit mit der Wohnsituation nach Lärmempfindlichkeit (gruppiert)

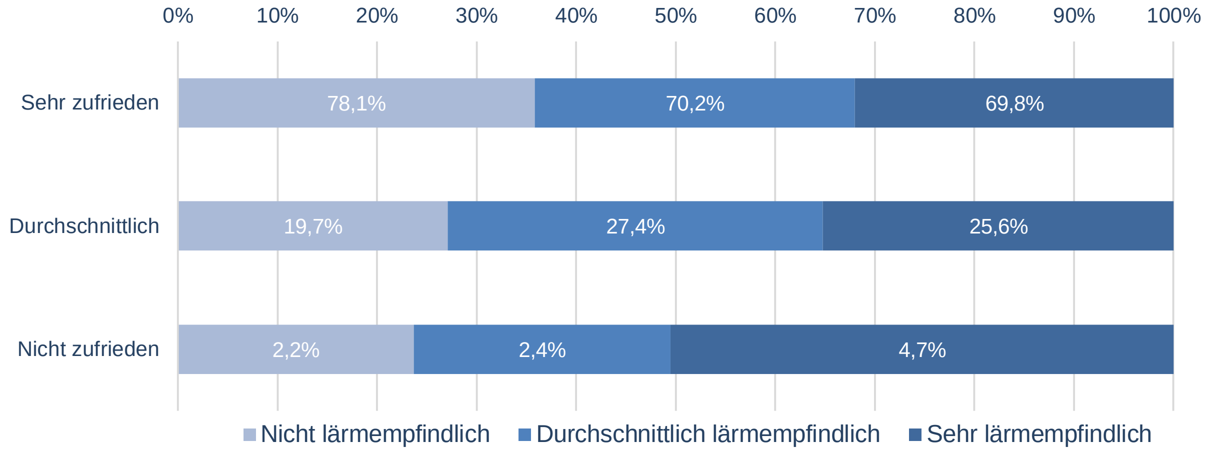

3. Wohnsituation und Lärmbelastung

3.1. Straßenverkehrslärmbelastung

Die Zufriedenheit mit der eigenen Wohnsituation ist bei den geringer vom 24h-Straßenverkehrslärm belasteten Befragten deutlich höher als bei den stärker Belasteten und vice versa:

Abbildung 74: Zufriedenheit mit der Wohnsituation und 24h-Straßenlärmbelastung L_{den} (gruppiert)

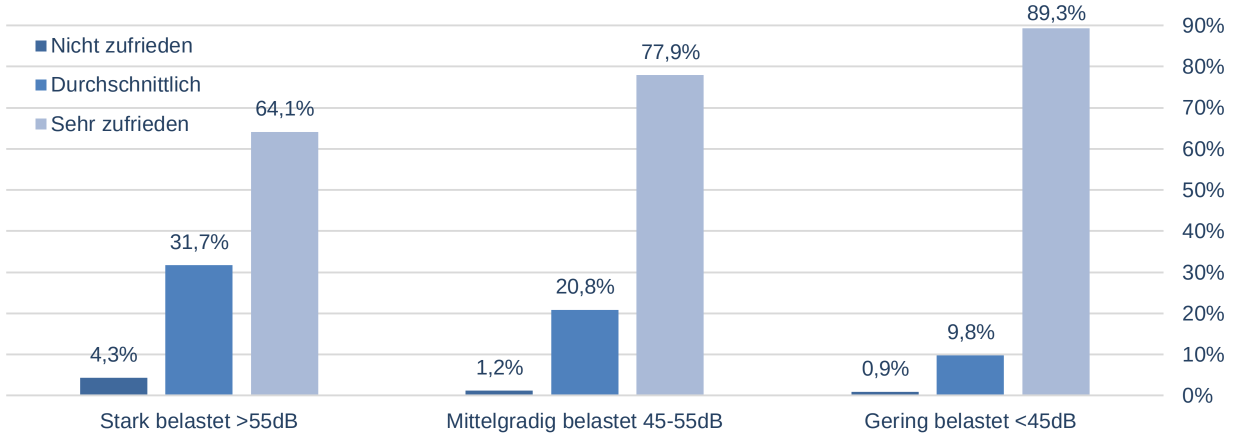

Neben den statistischen Korrelationen ist diese Beziehung auch aus den Mittelwerten nach gegebener Lärmbelastung ersichtlich:

Tabelle 68: Zufriedenheit mit der Wohnsituation und 24h-Straßenverkehrslärmbelastung L_{den} in 10-dB Schritten im Mittelwertvergleich

Straßenlärm 24h in 10-dB Schritten	Mittelwert	N	Standardabweichung
unter 30dB	10,00	2 (*)	,000
30 bis 40dB	8,62	26	1,699
40 bis 50dB	8,87	195	1,569
50 bis 60dB	8,01	531	2,154
über 60dB	7,70	276	2,233
Insgesamt	8,11	1030	2,105

(*) zu kleine Fallzahlen für solide Auswertungen

Abbildung 75: Zufriedenheit mit der Wohnsituation und 24h-Straßenverkehrslärmbelastung L_{den} in 10-dB Schritten im Mittelwertvergleich

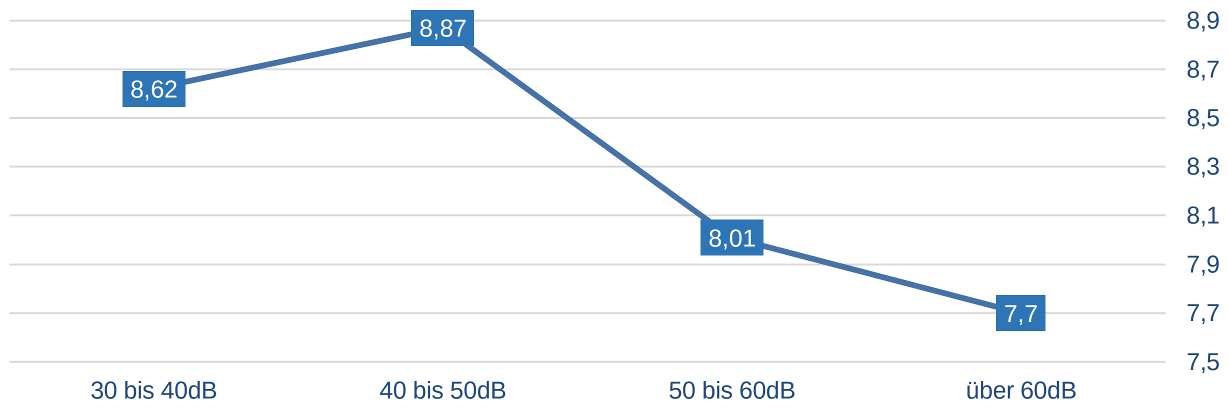

3.2. Flugverkehrslärmbelastung

Die 24h-Flugverkehrslärmbelastung zeigt im Gegensatz zum Straßenverkehrslärm (auch rein statistisch) nur geringe Zusammenhänge zum Ausmaß der Zufriedenheit mit der eigenen Wohnsituation:

Abbildung 76: Zufriedenheit mit der Wohnsituation und 24h-Fluglärmbelastung L_{den} (gruppiert)

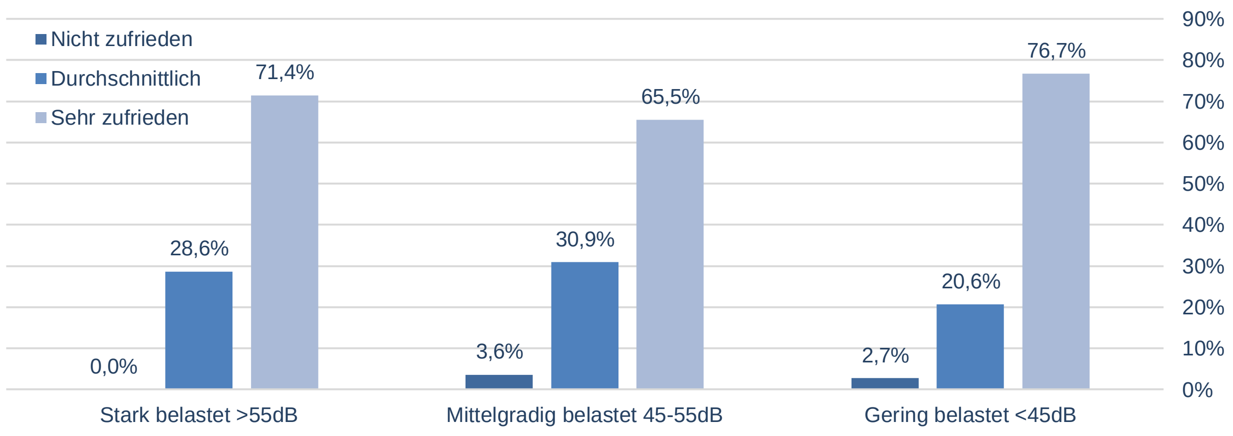

Tabelle 69: Zufriedenheit mit der Wohnsituation und 24h-Fluglärmbelastung L_{den} in 10-dB Schritten im Mittelwertvergleich

Fluglärm 24h in 10-dB Schritten	Mittelwert	N	Standardabweichung
30 bis 40dB	8,77	243	1,776
40 bis 50dB	7,84	620	2,232
50 bis 60dB	8,15	135	1,859
über 60dB	8,28	32	1,764
Insgesamt	8,11	1030	2,105

3.3. Schienenverkehrslärmbelastung

Die 24h-Schienenverkehrslärmbelastung zeigt keine eindeutigen Zusammenhänge zum Ausmaß der Zufriedenheit mit der eigenen Wohnsituation:

Abbildung 77: Zufriedenheit mit der Wohnsituation und 24h-Schienenlärmbelastung L_{den} (gruppiert)

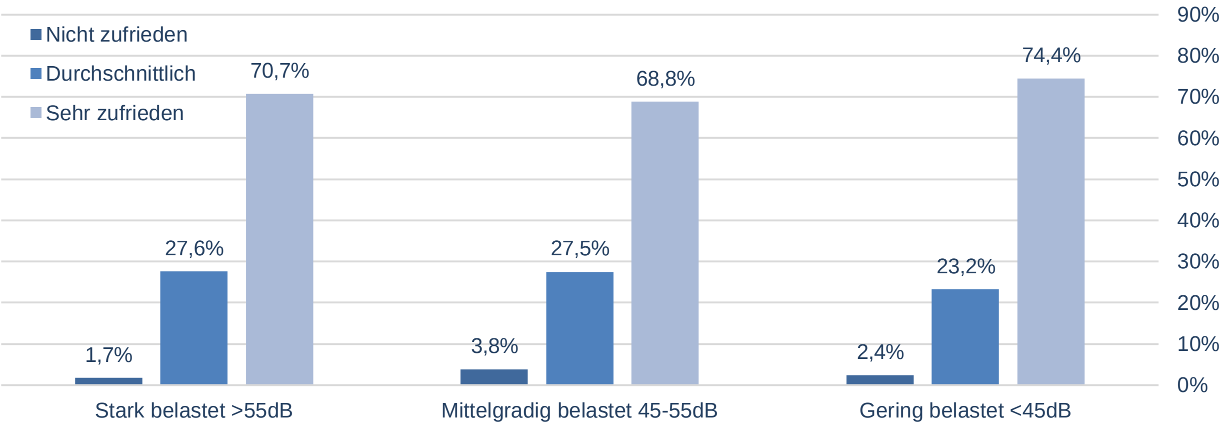

Tabelle 70: Zufriedenheit mit der Wohnsituation und 24h-Schienenverkehrslärmbelastung L_{den} in 10-dB Schritten im Mittelwertvergleich

Schienenlärm 24h in 10-dB Schritten	Mittelwert	N	Standardabweichung
unter 30dB	8,07	15	2,344
30 bis 40dB	8,26	284	2,055
40 bis 50dB	8,08	484	2,100
50 bis 60dB	7,97	209	2,214
über 60dB	8,16	38	1,853
Insgesamt	8,11	1030	2,105

4. Wohnsituation und nächtliche Lärmbelastung

4.1. Straßenverkehrslärmbelastung

Die rein statistischen Beziehungen zwischen dem nächtlichen Straßenverkehrslärm und der Zufriedenheit mit der eigenen Wohnsituation sind nur gering ausgeprägt. Aus den Daten ist ein Schwellenwert bei etwa 40dB nächtlicher Straßenverkehrslärm ersichtlich, ab welchem die Zufriedenheit mit der eigenen Wohnsituation abnimmt. Bis zu diesem Schwellenwert sind keine Auswirkungen des nächtlichen Straßenverkehrslärms auf die Zufriedenheit mit der eigenen Wohnsituation insgesamt erkennbar:

Abbildung 78: Zufriedenheit mit der Wohnsituation und nächtliche Straßenverkehrslärmbelastung L_n in 10-dB Schritten im Mittelwertvergleich

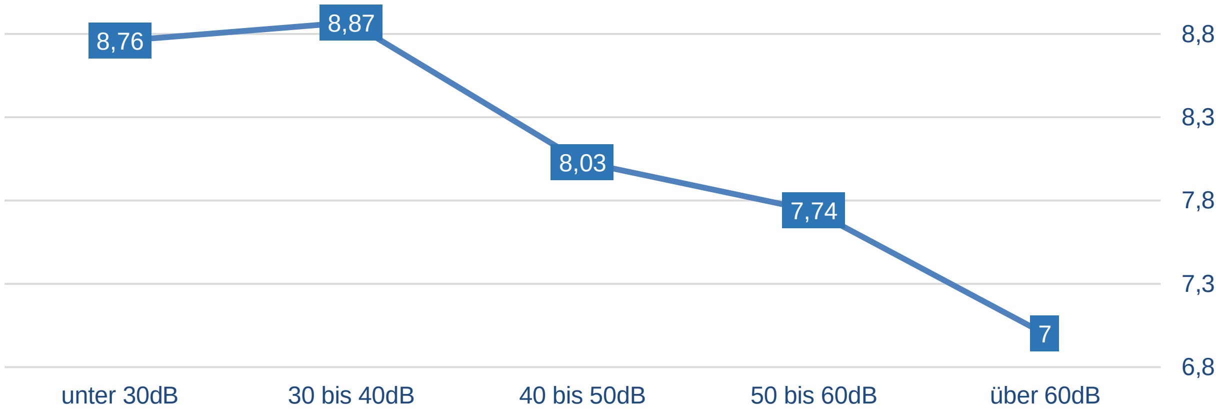

4.2. Flugverkehrslärmbelastung

Das Gleiche gilt für die Beziehung zum Flugverkehrslärm in der Nacht, welche nur gering ausgeprägt ist. Neben dem auch schon in der Zusammenschau mit dem 24h-Flugverkehrslärmpegel gering ausgeprägten Zusammenhang, spielt auch die geringe Flugverkehrslärmbelastung in Innsbruck in der Nacht eine Rolle: Keiner der 9711 zu Wohnzwecken benutzte Adresspunkte in Innsbruck verzeichnet eine nächtliche Flugverkehrslärmbelastung L_n über 50dB:

Tabelle 71: Zufriedenheit mit der Wohnsituation und nächtliche Flugverkehrslärmbelastung L_n in 10-dB Schritten im Mittelwertvergleich

Fluglärm Nacht in 10-dB Schritten	Mittelwert	N	Standardabweichung
unter 30dB	8,32	637	2,003
30 bis 40dB	7,81	349	2,236
40 bis 50dB	7,50	44	2,119
Insgesamt	8,11	1030	2,105

4.3. Schienenverkehrslärmbelastung

Zwischen dem nächtlichen Schienenverkehrslärm und dem Ausmaß der Zufriedenheit mit der eigenen Wohnsituation besteht statistisch kein eindeutig erkennbarer Zusammenhang. Das lässt sich auch über die Mittelwerte der Zufriedenheit mit der eigenen Wohnsituation in Zusammenschau mit den gestaffelten Lärmbelastungen in 10-dB-Schritten nachvollziehen:

Tabelle 72: Zufriedenheit mit der Wohnsituation und nächtliche Schienenverkehrslärmbelastung L_n in 10-dB Schritten im Mittelwertvergleich

Schienenlärm Nacht in 10-dB Schritten	Mittelwert	N	Standardabweichung
unter 30dB	8,83	88	1,656
30 bis 40dB	8,08	584	2,107
40 bis 50dB	7,96	274	2,234
50 bis 60dB	8,04	81	1,978
über 60dB	8,67	3	1,528
Insgesamt	8,11	1030	2,105

5. Wohnsituation und Lärmexpositionsempfinden

5.1. Verkehrsverursachtes Lärmexpositionsempfinden

Befragte, die sich als stark verkehrslärmexponiert ansehen, sind weniger zufrieden mit ihrer persönlichen Wohnsituation als sich weniger dem Verkehrslärm ausgesetzt sehende Probandinnen und Probanden. Auch statistisch zeigen sich die stärksten Zusammenhänge in Bezug auf die Lärmexposition insgesamt und die Ausgesetztheit gegenüber Straßenverkehrslärm, geringer ausgeprägte Beziehungen bestehen zum Expositionsempfinden gegenüber dem Schienen- und dem Flugverkehrslärm:

Abbildung 79: Zufriedenheit mit der Wohnsituation und verkehrsverursachtes Lärmexpositionsempfinden im Mittelwertvergleich

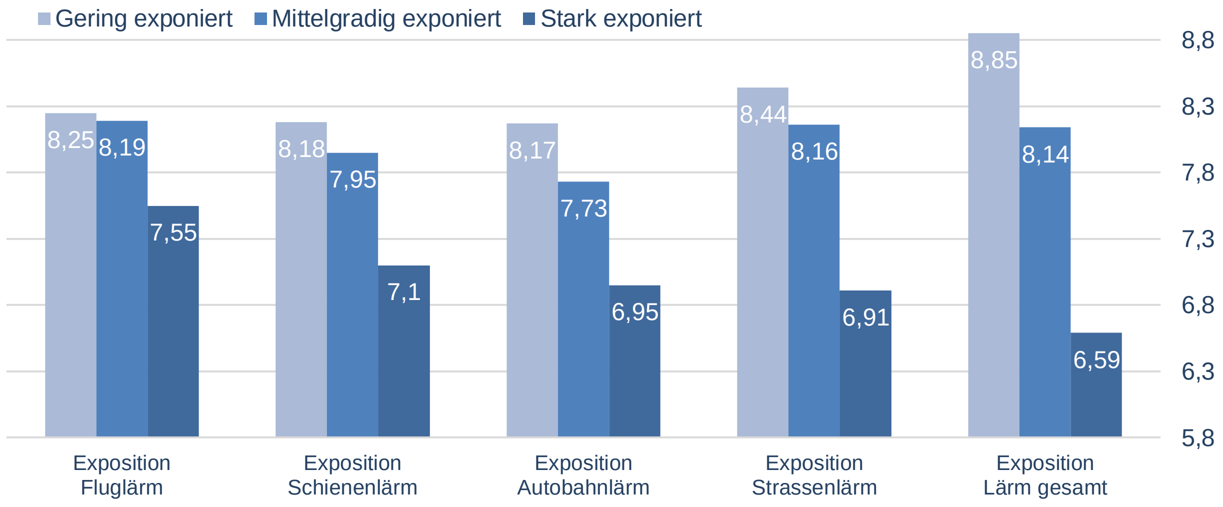

5.2. Nichtverkehrsverursachtes Lärmexpositionsempfinden

Sich stark nichtverkehrsverursachtem Lärm ausgesetzt empfindende Befragte schätzen ihre Zufriedenheit mit der eigenen Wohnsituation deutlich schlechter ein, als dies sich weniger ausgesetzt fühlende Befragte tun. Die Unterschiede treten stärker als beim verkehrsverursachten Lärm zutage und besonders befragte Innsbruckerinnen und Innsbrucker, die sich dem Nachbarschaftslärm oder aber Erschütterungen stark ausgesetzt sehen, beurteilen ihre eigene Wohnsituation insgesamt um mehr als 2 Antwortkategorien schlechter als gering Exponierte angeben. In Zusammenschau mit den Belästigungsreaktionen (siehe das Kapitel: *Belästigung / Störung durch Lärm*) wird ersichtlich, dass Akutbelastungseffekte durch nicht-verkehrsverursachtem Lärm eine deutliche negative Beziehung zu einer Reihe von subjektiven Einschätzungsparametern aufweisen, die in ihrer Ausprägung stärker als beim verkehrsverursachten Lärm zutage treten:

Abbildung 80: Zufriedenheit mit der Wohnsituation und nichtverkehrsverursachtes Lärmexpositionsempfinden im Mittelwertvergleich

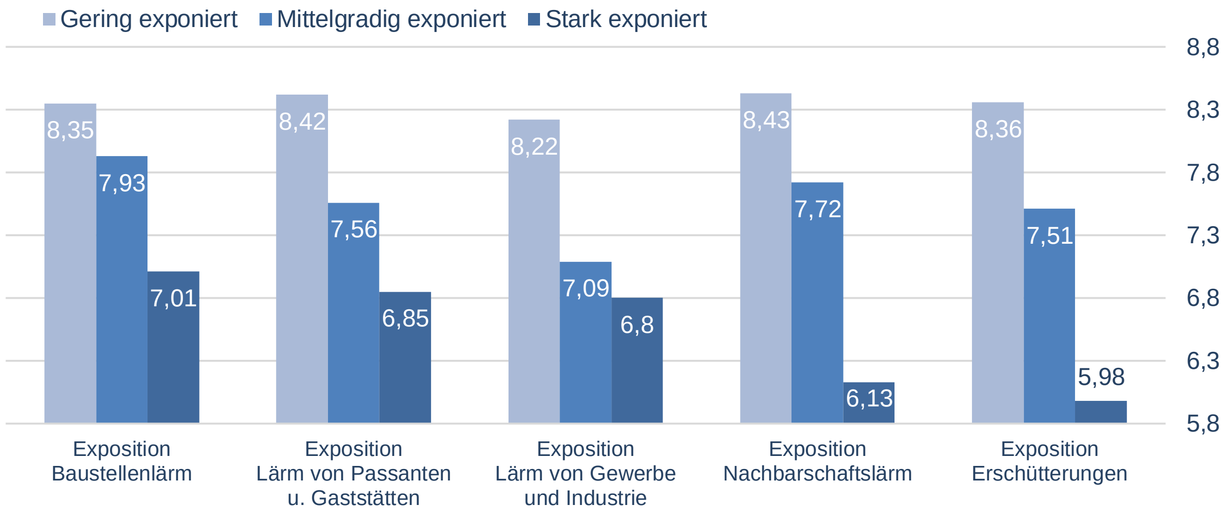

6. Wohnsituation und Lärmbelästigung

Das Ausmaß der empfundenen Lärmbelästigung zeigt einen Zusammenhang zur Zufriedenheit mit der eigenen Wohnsituation:

Abbildung 81: Zufriedenheit mit der Wohnsituation und Lärmbelästigung gesamt im Mittelwertvergleich

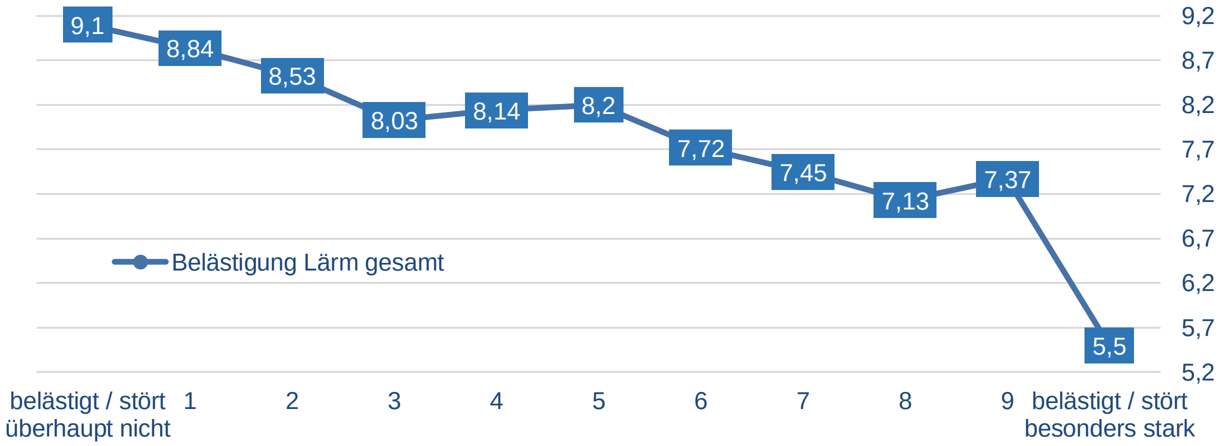

Je stärker die Lärmbelästigung, desto geringer das Ausmaß der Zufriedenheit mit der persönlichen Wohnsituation:

Tabelle 73: Zufriedenheit mit der Wohnsituation und Lärmbelästigung gesamt im Mittelwertvergleich

Belästigung Lärm gesamt	Mittelwert	N	Standardabweichung
belästigt/stört überhaupt nicht	9,10	137	1,592
1	8,84	80	1,513
2	8,53	160	1,598
3	8,03	146	1,960
4	8,14	90	1,758
5	8,20	128	1,829
6	7,72	72	2,177
7	7,45	92	2,160
8	7,13	62	2,773
9	7,37	27	2,589
belästigt/stört besonders stark	5,50	36	3,334
Insgesamt	8,11	1030	2,105

Die Zusammenhänge sind je nach Quelle der Lärmbelästigung unterschiedlich stark ausgeprägt. Am stärksten beim Lärm insgesamt, sowie dem Lärm von Passanten und Gaststätten, Nachbarschaftslärm, Erschütterungen und Straßenverkehrslärm. Am schwächsten bis kaum vorhanden sind statistisch eindeutige Zusammenhänge zwischen der Zufriedenheit mit der eigenen Wohnsituation und Autobahn-, Flug- und Schienenverkehrslärm:

Abbildung 82: Zufriedenheit mit der Wohnsituation und Lärmbelästigung nach Quelle (gruppiert) im Mittelwertvergleich (aufsteigend sortiert nach Pearsons Rho)

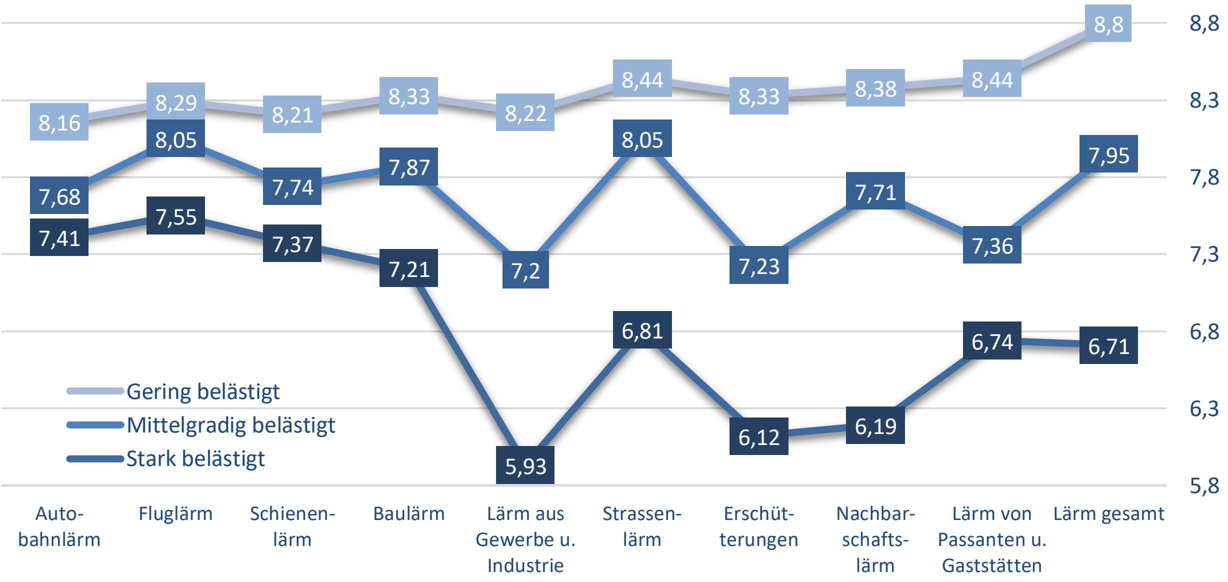

7. Wohnsituation und Lärmbelästigung im Schlaf

Für die Beziehungen zum Belästigungsempfinden in der Nacht ist ebenfalls eine sehr vergleichbare Funktion ablesbar. Je geringer das Belästigungsempfinden durch Lärm in der Nacht, desto größer die Zufriedenheit mit der eigenen Wohnsituation.

Allerdings sind die Ergebnisse für die Extremgruppe der sich stark vom Lärm nach einzelnen Quellen belästigt fühlenden Befragten aufgrund der geringen Fallzahlen weiter streuend und weniger solide:

Abbildung 83: Zufriedenheit mit der Wohnsituation und Lärmbelästigung in der Nacht nach Quelle (gruppiert) im Mittelwertvergleich (aufsteigend sortiert nach Pearsons Rho)

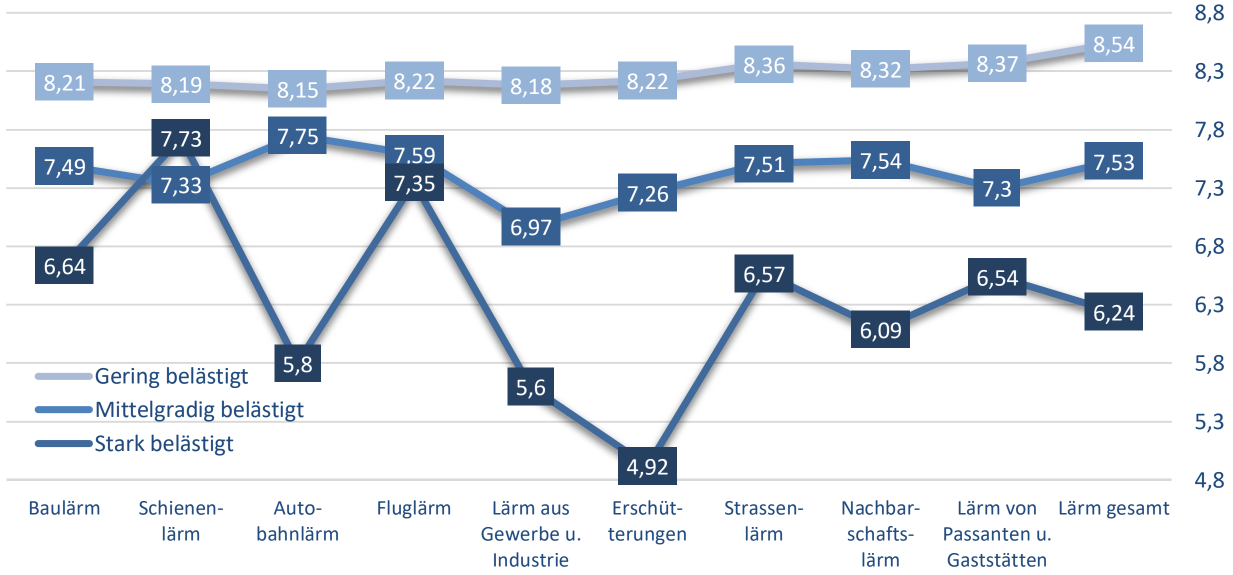

Gesundheitszustand

1. Gesundheitszustand im Überblick

Knapp drei Viertel aller Befragten schätzen ihren Gesundheitszustand als *gut* bzw. *sehr gut* ein, 8,6% als *weniger gut* bzw. *schlecht*. 2 Befragte machen keine Angaben:

Abbildung 84: Einschätzung des eigenen Gesundheitszustandes in Prozent

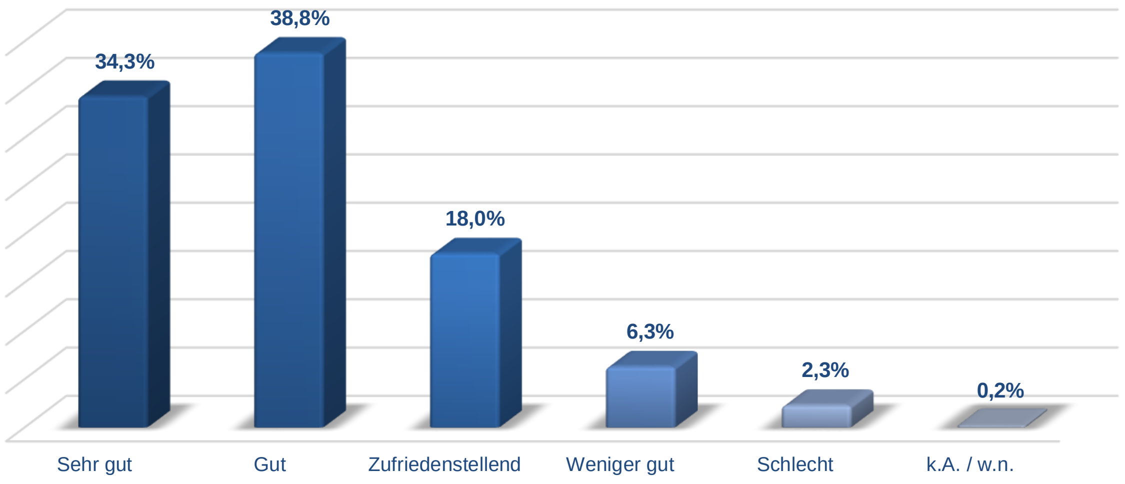

Tabelle 74: Einschätzung des eigenen Gesundheitszustandes gruppiert

		Häufigkeit	Gültige Prozente	Kumulierte Prozente
Gültig	Gut	754	73,1	73,1
	Zufriedenstellend	186	18,0	91,2
	Nicht gut	89	8,6	99,8
	w.n. / k.A.	2	,2	100,0
	Gesamt	1031	100,0	

2. Gesundheitszustand und ausgewählte Parameter

Den größten Zusammenhang zur Einschätzung des eigenen Gesundheitszustandes zeigt erwartungsgemäß das Alter der Befragten.

Männer schätzen ihren eigenen Gesundheitszustand geringfügig besser ein als Frauen.

Die Selbsteinschätzung der eigenen Lärmempfindlichkeit und des eigenen Gesundheitszustandes stehen nur geringfügig in Beziehung zueinander. Gleiches gilt für die Einschätzung des Ausmaßes negativer Auswirkungen von Lärm auf die Gesundheit.

Ausgeprägt ist hingegen die Beziehung mit der Beurteilung der eigenen Lebensqualität: Befragte mit schlechtem selbstreferenzierten Gesundheitszustand bewerten auch ihr Lebensqualität schlechter als Befragte mit gutem Gesundheitszustand und vice versa:

Tabelle 75: Einschätzung des eigenen Gesundheitszustandes (gruppiert) und ausgewählte Variablen

		Güte eigener Gesundheitszustand gruppiert					
		Gut		Zufriedenstellend		Nicht gut	
		Anzahl	Zeilen (%)	Anzahl	Zeilen (%)	Anzahl	Zeilen (%)
**Geschlecht**	weiblich	388	72,0%	97	18,0%	54	10,0%
	männlich	366	74,7%	89	18,2%	35	7,1%
**Altersgruppen**	18 bis 40 Jahre	377	85,7%	51	11,6%	12	2,7%
	41 bis 60 Jahre	221	69,9%	56	17,7%	39	12,3%
	über 60 Jahre	156	57,1%	79	28,9%	38	13,9%
**Bildungsschicht**	Primärer Schulabschluss	54	55,1%	28	28,6%	16	16,3%
	Sekundärer Schulabschluss	375	69,4%	109	20,2%	56	10,4%
	Tertiärer Schulabschluss	323	83,2%	48	12,4%	17	4,4%
**Negative Auswirkung von Lärm auf die Gesundheit**	Wenig	160	73,7%	42	19,4%	15	6,9%
	Stark	573	73,6%	138	17,7%	68	8,7%
**Lebensqualität**	Nicht gut (*)	1	11,1%	2	22,2%	6	66,7%
	Durchschnittlich	177	61,0%	71	24,5%	42	14,5%
	Sehr gut	572	78,9%	113	15,6%	40	5,5%
**Lärmempfindlichkeit**	Nicht lärmempfindlich	206	74,9%	46	16,7%	23	8,4%
	Durchschnittlich	426	73,2%	112	19,2%	44	7,6%
	Sehr lärmempfindlich	122	70,9%	28	16,3%	22	12,8%

* zu kleine Fallzahlen für solide Auswertungen

Auffällig ist die Zusammenschau Gesundheitszustand und höchster erreichter Bildungsabschluss: Befragte mit Pflichtschulabschluss gehen von einem deutlich schlechteren eigenen Gesundheitszustand aus als Befragte mit sekundärem Bildungsabschluss und diese unterscheiden sich wiederum ebenso deutlich von befragten Akademikerinnen und Akademikern. Diese Unterschiede bleiben auch altersbereinigt erhalten und sind besonders in den beiden höheren Altersklassen sehr ausgeprägt:

Abbildung 85: Einschätzung des eigenen Gesundheitszustandes und höchster erreichter Schulabschluss nach Altersgruppen (gruppiert) im Mittelwertvergleich

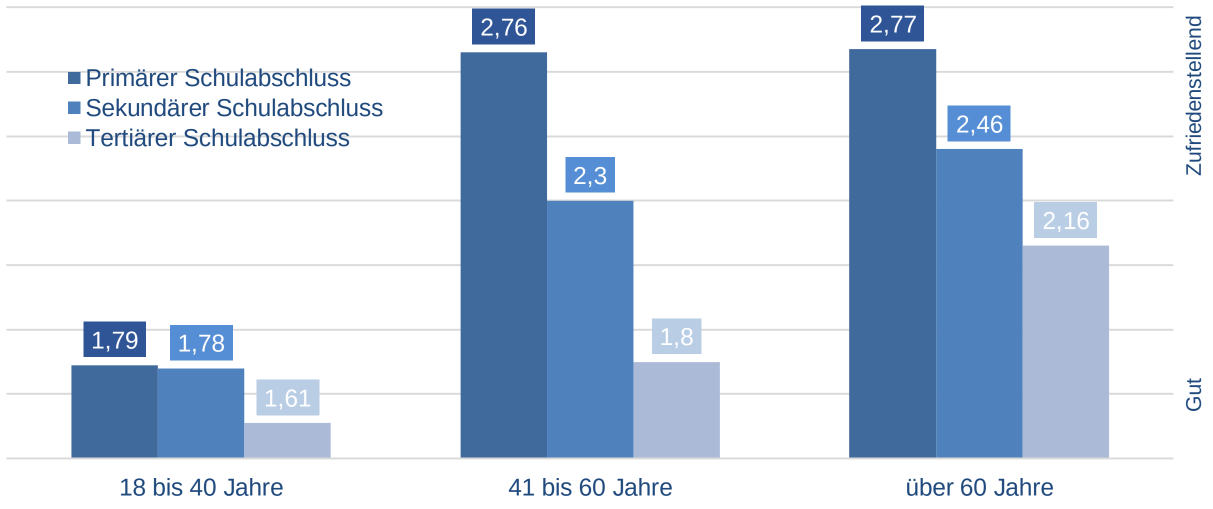

3. Gesundheitszustand und Lärmbelastung

Die Selbsteinschätzung des persönlichen Gesundheitszustandes weist einen Bezug zum Straßenverkehrslärm auf, zeigt aber keine eindeutige Beziehung zur tatsächlich vorhandenen Gesamtlärmbelastung im Wohnumfeld der Befragten:

Tabelle 76: Bivariate Korrelation zu Gesundheitszustand und 24h-Lärmbelastung L_{den} nach Quelle

		Straßenlärm 24h	Schienenlärm 24h	Fluglärm 24h
Güte eigener Gesundheitszustand	Korrelation nach Pearson	,065*	,028	-,016
	Signifikanz (2-seitig)	,037	,363	,617
	N	1029	1029	1029

*. Die Korrelation ist auf dem Niveau von 0,05 (2-seitig) signifikant.

4. Gesundheitszustand und Lärmexpositionsempfinden

Befragte mit schlechterem selbstreferenzierten Gesundheitszustand empfinden sich geringfügig stärker dem Lärm in ihrem Wohnumfeld ausgesetzt, als Innsbruckerinnen und Innsbrucker mit gutem persönlichen Gesundheitszustand und umgekehrt. Dieser Effekt ist nahezu unabhängig von der Lärmquelle vorhanden:

Tabelle 77: Gesundheitszustand und Lärmexpositionsempfinden nach Quelle (gruppier)

		Güte eigener Gesundheitszustand gruppiert					
		**Gut**		**Zufriedenstellend**		**Nicht gut**	
		Anzahl	Zeilen (%)	Anzahl	Zeilen (%)	Anzahl	Zeilen (%)
**Ausgesetztheit Lärm gesamt**	Gering exponiert	197	75,8%	43	16,5%	20	7,7%
	Mittelgradig exponiert	453	72,0%	123	19,6%	53	8,4%
	Stark exponiert	104	74,3%	20	14,3%	16	11,4%
**Ausgesetztheit Fluglärm**	Gering exponiert	298	74,9%	75	18,8%	25	6,3%
	Mittelgradig exponiert	342	73,4%	79	17,0%	45	9,7%
	Stark exponiert	114	69,1%	32	19,4%	19	11,5%
**Ausgesetztheit Strassenlärm**	Gering exponiert	323	75,5%	75	17,5%	30	7,0%
	Mittelgradig exponiert	338	72,8%	85	18,3%	41	8,8%
	Stark exponiert	93	67,9%	26	19,0%	18	13,1%
**Ausgesetztheit Baulärm**	Gering exponiert	501	74,7%	122	18,2%	48	7,2%
	Mittelgradig exponiert	171	68,1%	52	20,7%	28	11,2%
	Stark exponiert	82	76,6%	12	11,2%	13	12,1%
**Ausgesetztheit Nachbarschaftslärm**	Gering exponiert	518	73,1%	130	18,3%	61	8,6%
	Mittelgradig exponiert	196	76,3%	44	17,1%	17	6,6%
	Stark exponiert	40	63,5%	12	19,0%	11	17,5%
**Ausgesetztheit Lärm von Passanten**	Gering exponiert	531	74,7%	122	17,2%	58	8,2%
	Mittelgradig exponiert	184	71,6%	49	19,1%	24	9,3%

**u. Gaststätten**	Stark exponiert	39	63,9%	15	24,6%	7	11,5%
**Ausgesetztheit Erschütterungen**	Gering exponiert	595	74,0%	148	18,4%	61	7,6%
	Mittelgradig exponiert	134	73,2%	30	16,4%	19	10,4%
	Stark exponiert	25	59,5%	8	19,0%	9	21,4%
**Ausgesetztheit Schienenlärm**	Gering exponiert	609	72,2%	163	19,3%	71	8,4%
	Mittelgradig exponiert	121	77,1%	19	12,1%	17	10,8%
	Stark exponiert	24	82,8%	4	13,8%	1 (*)	3,4%
**Ausgesetztheit Autobahnlärm**	Gering exponiert	687	74,0%	165	17,8%	77	8,3%
	Mittelgradig exponiert	53	65,4%	17	21,0%	11	13,6%
	Stark exponiert	14	73,7%	4 (*)	21,1%	1 (*)	5,3%
**Ausgesetztheit Lärm von Gewerbe und Industrie**	Gering exponiert	686	73,8%	161	17,3%	83	8,9%
	Mittelgradig exponiert	53	67,1%	21	26,6%	5	6,3%
	Stark exponiert	15	75,0%	4 (*)	20,0%	1 (*)	5,0%

* zu kleine Fallzahlen für solide Auswertungen

5. Gesundheitszustand und Lärmbelästigung

Zwischen der Einschätzung des eigenen Gesundheitszustandes und dem Ausmaß der Lärmbelästigung besteht nur ein sehr geringer direkter Zusammenhang. Im Vergleich der Extremgruppen lassen sich jedoch Unterschiede ausmachen:

Abbildung 86: Gesundheitszustand und Lärmbelästigung nach Quelle (gruppiert) im Mittelwertvergleich (aufsteigend sortiert nach Extremwertdifferenz)

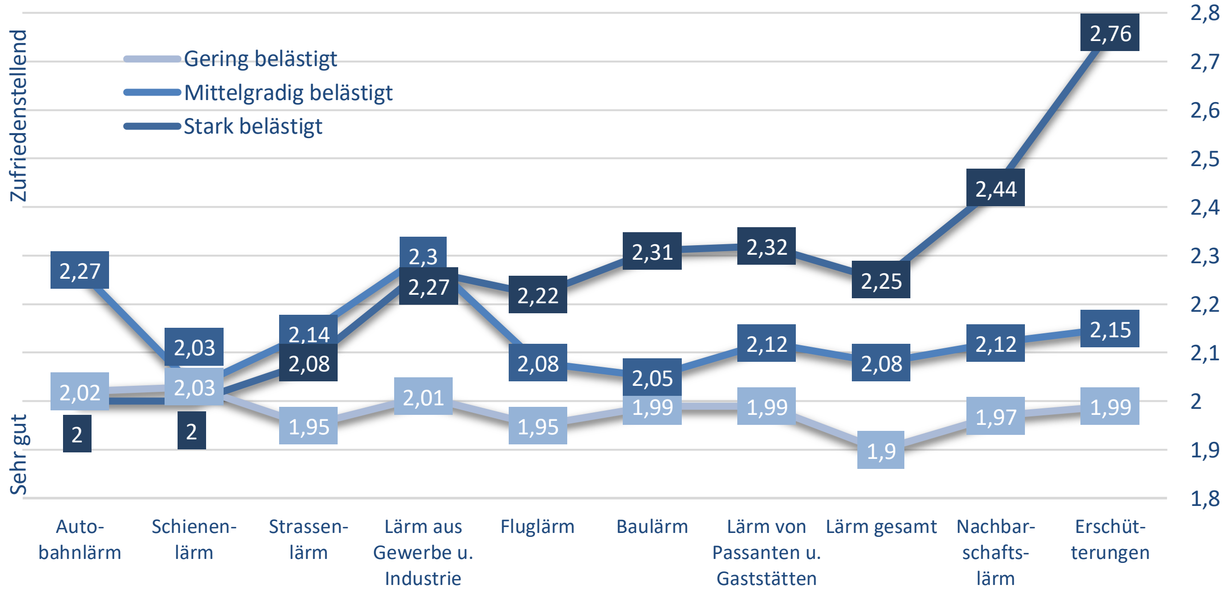

6. Gesundheitszustand und Belästigung durch Lärm im Schlaf

Die Einschätzung des eigenen Gesundheitszustandes und die Belästigung durch Lärm im Schlaf zeigen auch differenziert nach einzelnen Belästigungsquellen keine ausgeprägten Beziehungen zueinander.

Lebensqualität

1. Lebensqualität im Überblick

Genau 1% aller Befragten beurteilt die eigene Lebensqualität in den drei untersten Antwortkategorien und damit als schlecht, über 70% sind hingegen der Meinung, dass die persönliche Lebensqualität sehr hoch ist und antworten in den drei obersten Antwortkategorien. 6 Befragte machen keine Angaben:

Abbildung 87: Einschätzung der persönlichen Lebensqualität in Prozent

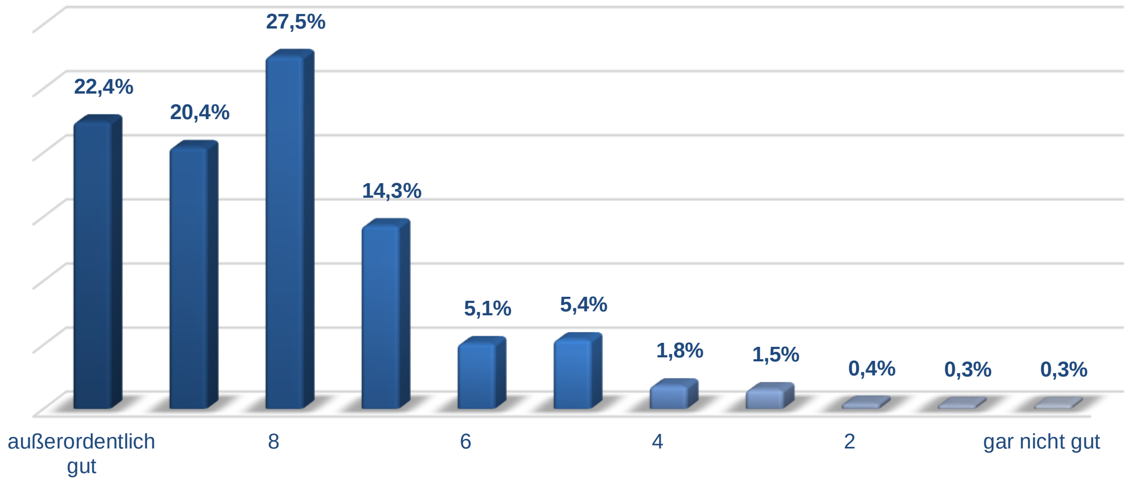

Der arithmetische Mittelwert der Antworten liegt auf der 11-er Skala von *gar nicht gut* bis *außerordentlich gut* bei 8,03 (Standardfehler 0,055), das entspricht einem durchschnittlichen Ausmaß der Einschätzung der persönlichen Lebensqualität der befragten Innsbruckerinnen und Innsbrucker von 88,3% vom Maximalwert.

Tabelle 78: Einschätzung der persönlichen Lebensqualität gruppiert

		Häufigkeit	Gültige Prozente	Kumulierte Prozente
Gültig	Nicht gut	10	1,0	1,0
	Durchschnittlich	290	28,1	29,1
	Sehr gut	725	70,3	99,4
	w.n. / k.A.	6	,6	100,0
	Gesamt	1031	100,0	

2. Lebensqualität und ausgewählte Parameter

Frauen schätzen ihre persönliche Lebensqualität etwas höher als Männer ein.

Die Einschätzung der eigenen Lebensqualität zeigt nur eine geringe Beziehung zum Alter der Befragten:

Tabelle 79: Einschätzung der eigenen Lebensqualität (gruppiert) und ausgewählte Variablen

		Güte eigene Lebensqualität gruppiert					
		Nicht gut (*)		Durchschnittlich		Sehr gut	
		Anzahl	Zeilen (%)	Anzahl	Zeilen (%)	Anzahl	Zeilen (%)
**Geschlecht**	weiblich	8	1,5%	141	26,2%	390	72,4%
	männlich	2	0,4%	149	30,7%	335	68,9%
**Altersgruppen**	18 bis 40 Jahre	2	0,5%	119	27,1%	318	72,4%
	41 bis 60 Jahre	6	1,9%	89	28,4%	218	69,6%
	über 60 Jahre	2	0,7%	82	30,0%	189	69,2%
**Bildungsschicht**	Primärer Schulabschluss	2	2,1%	40	41,7%	54	56,3%
	Sekundärer Schulabschluss	5	0,9%	171	31,7%	364	67,4%
	Tertiärer Schulabschluss	3	0,8%	78	20,2%	305	79,0%
**Gesundheitszustand**	Gut	1	0,1%	177	23,6%	572	76,3%
	Zufriedenstellend	2	1,1%	71	38,2%	113	60,8%
	Nicht gut	6	6,8%	42	47,7%	40	45,5%
**Lärmempfindlichkeit**	Nicht lärmempfindlich	1	0,4%	67	24,5%	206	75,2%
	Durchschnittlich	6	1,0%	174	30,0%	400	69,0%
	Sehr lärmempfindlich	3	1,8%	49	28,7%	119	69,6%
**Lautheit Innsbruck im Vergleich zu anderen Städten Österreichs**	Ruhiger	2	0,7%	73	25,0%	217	74,3%
	Gleich	6	1,1%	139	25,5%	401	73,4%
	Lauter	2	1,1%	73	42,0%	99	56,9%

* zu kleine Fallzahlen für solide Auswertungen

Knapp 80% der Befragten mit tertiärem höchsten erreichten Schulabschluss schätzen ihre eigene Lebensqualität als sehr gut ein, bei den Probandinnen und Probanden aus der sekundären Bildungsschicht sinkt dieser Anteil auf knapp über zwei Drittel und bei Befragten mit Pflichtschulabschluss beurteilen nur mehr 56% ihre persönliche Lebensqualität als sehr gut:

Abbildung 88: Einschätzung der Lebensqualität und höchster erreichter Schulabschluss (gruppiert)

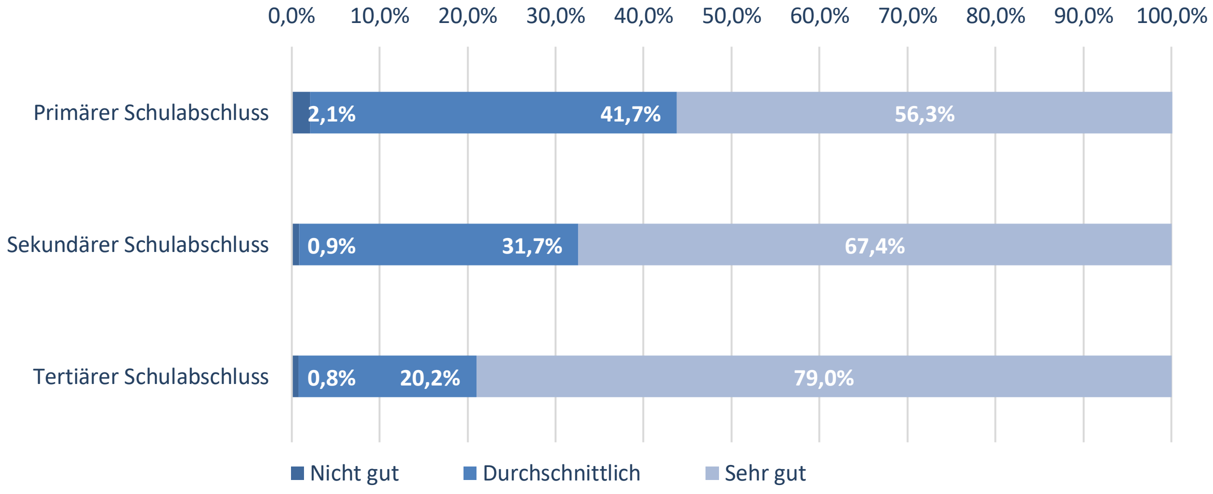

Auch altersbereinigt bleiben diese Unterschiede annähernd bestehen.

Noch stärker zeigen sich Zusammenhänge in Zusammenschau mit dem selbstreferenzierten Gesundheitszustand der Befragten, weniger deutlich sind hingegen die Verbindungen zur Lärmempfindlichkeit.

Befragte Innsbruckerinnen und Innsbrucker, welche der Meinung sind, dass es in Innsbruck im Vergleich zu anderen Städten in Österreich lauter ist, beurteilen auch ihre persönliche Lebensqualität schlechter als Probandinnen und Probanden, die glauben, dass es in Innsbruck insgesamt leiser oder gleich laut wie in vergleichbaren österreichischen Städten ist.

3. Lebensqualität und Lärmbelastung

Die Einschätzung der Güte der eigenen Lebensqualität zeigt nur geringfügige Beziehungen zur tatsächlich vorhandenen Lärmbelastung im Wohnumfeld der Befragten:

Tabelle 80: Bivariate Korrelation zu Lebensqualität und 24h-Lärmbelastung L_{den} nach Quelle

		Straßenlärm 24h	Schienenlärm 24h	Fluglärm 24h
Güte eigene Lebensqualität	Korrelation nach Pearson	-,181**	-,045	-,087**
	Signifikanz (2-seitig)	,000	,149	,006
	N	1025	1025	1025

**. Die Korrelation ist auf dem Niveau von 0,01 (2-seitig) signifikant.

Eine (allerdings statistisch nur gering ausgeprägte) Beziehung besteht zwischen der Einschätzung der eigenen Lebensqualität und der nächtlichen Straßenlärmbelastung. Mit steigenden nächtlichen Straßenlärmpegeln sinkt die Einschätzung der eigenen Lebensqualität deutlich:

Abbildung 89: Einschätzung der eigenen Lebensqualität und nächtliche Straßenverkehrslärmbelastung L_n in 10dB-Schritten im Mittelwertvergleich

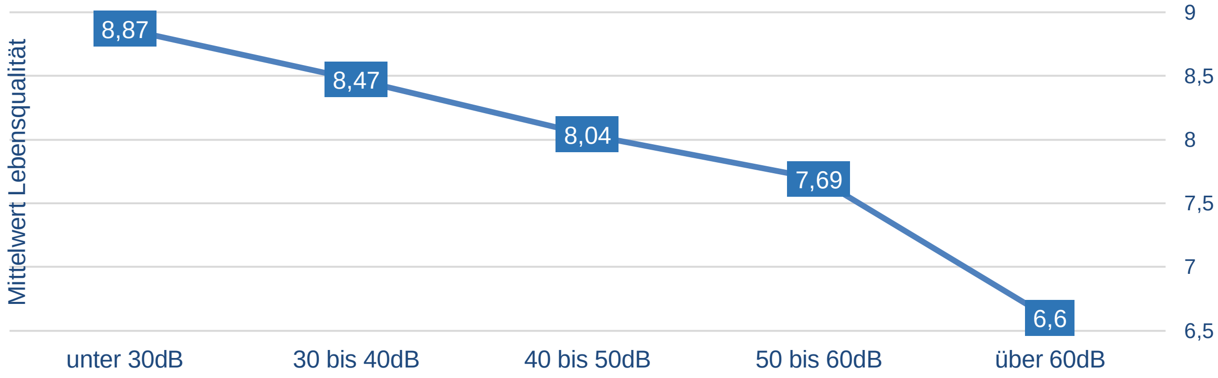

4. Lebensqualität und Lärmexpositionsempfinden

Ausgeprägter als zur tatsächlich gegebenen Lärmbelastung zeigen sich Zusammenhänge zur empfundenen Ausgesetztheit gegenüber Lärm. Vor allem in der Zusammenschau der Einschätzung der eigenen Lebensqualität mit dem Expositionsempfinden gegenüber dem Lärm insgesamt, dem Straßenverkehrslärm und dem Nachbarschaftslärm zeigen sich Bezüge:

Abbildung 90: Lebensqualität und Expositionsempfinden Lärm insgesamt im Mittelwertvergleich

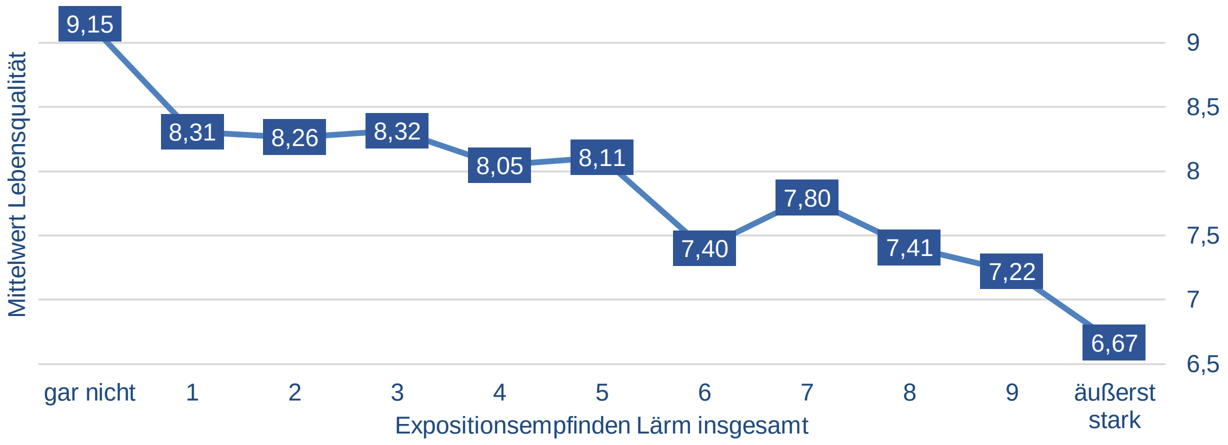

Je stärker sich Befragte lärmexponiert fühlen, desto geringer schätzen sie ihre persönliche Lebensqualität ein:

Tabelle 81: Lebensqualität und Lärmexpositionsempfinden nach Quelle (gruppier)

		Güte eigene Lebensqualität gruppiert					
		Nicht gut (*)		Durchschnittlich		Sehr gut	
		Anzahl (*)	Zeilen (%)	Anzahl	Zeilen (%)	Anzahl	Zeilen (%)
Ausgesetztheit Lärm gesamt	Gering exponiert	2	0,8%	47	18,1%	210	81,1%
	Mittelgradig exponiert	3	0,5%	186	29,7%	437	69,8%
	Stark exponiert	5	3,6%	57	40,7%	78	55,7%
Ausgesetztheit Fluglärm	Gering exponiert	2	0,5%	103	25,9%	292	73,6%
	Mittelgradig exponiert	4	0,9%	135	29,1%	325	70,0%
	Stark exponiert	4	2,4%	52	31,7%	108	65,9%
Ausgesetztheit Strassenlärm	Gering exponiert	5	1,2%	110	25,9%	310	72,9%
	Mittelgradig exponiert	2	0,4%	124	26,7%	338	72,8%
	Stark exponiert	3	2,2%	56	41,2%	77	56,6%
Ausgesetztheit Baulärm	Gering exponiert	4	0,6%	170	25,4%	495	74,0%
	Mittelgradig exponiert	2	0,8%	83	33,2%	165	66,0%
	Stark exponiert	4	3,8%	37	34,9%	65	61,3%
Ausgesetztheit Nachbarschaftslärm	Gering exponiert	7	1,0%	174	24,6%	526	74,4%
	Mittelgradig exponiert	0	0,0%	82	32,0%	174	68,0%
	Stark exponiert	3	4,8%	34	54,8%	25	40,3%
Ausgesetztheit Lärm von Passanten	Gering exponiert	6	0,8%	175	24,7%	527	74,4%
	Mittelgradig exponiert	1	0,4%	97	37,9%	158	61,7%

**u. Gaststätten**	Stark exponiert	3	4,9%	18	29,5%	40	65,6%
**Ausgesetztheit Erschütterungen**	Gering exponiert	4	0,5%	204	25,5%	593	74,0%
	Mittelgradig exponiert	4	2,2%	70	38,5%	108	59,3%
	Stark exponiert	2	4,8%	16	38,1%	24	57,1%
**Ausgesetztheit Schienenlärm**	Gering exponiert	10	1,2%	232	27,7%	597	71,2%
	Mittelgradig exponiert	0	0,0%	48	30,6%	109	69,4%
	Stark exponiert	0	0,0%	10	34,5%	19	65,5%
**Ausgesetztheit Autobahnlärm**	Gering exponiert	8	0,9%	258	27,9%	659	71,2%
	Mittelgradig exponiert	2	2,5%	24	29,6%	55	67,9%
	Stark exponiert	0	0,0%	8	42,1%	11	57,9%
**Ausgesetztheit Lärm von Gewerbe und Industrie**	Gering exponiert	8	0,9%	251	27,1%	667	72,0%
	Mittelgradig exponiert	1	1,3%	34	43,0%	44	55,7%
	Stark exponiert	1	5,0%	5	25,0%	14	70,0%

* zu kleine Fallzahlen für solide Auswertungen

5. Lebensqualität und Lärmbelästigung

Deutlich manifestieren sich die Unterschiede in der Beurteilung der eigenen Lebensqualität in Kombination mit dem Ausmaß der Lärmbelästigung:

Abbildung 91: Lebensqualität und Belästigung durch Lärm insgesamt im Mittelwertvergleich

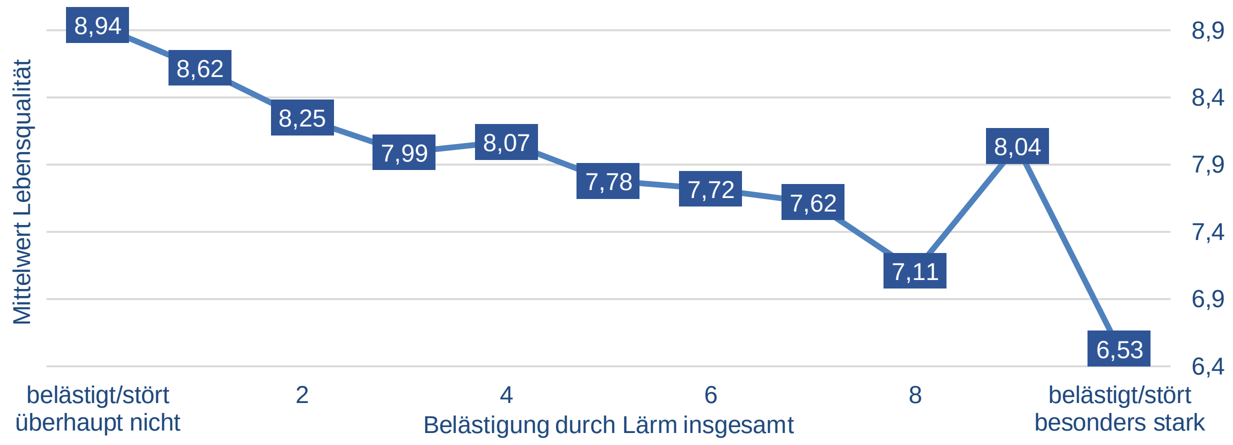

Anm.: Bei Antwortkategorie 9 zeigt sich ein Ausreißer (Standardabweichung 2,128)

Bei der Zusammenschau der Beurteilung der eigenen Lebensqualität und der Lärmbelästigung fällt auf, dass vor allem bei der Belästigung durch Schienenverkehrs- und Autobahnlärm die beiden Extremgruppen der Gering- und der Starkbelästigten in der Beurteilung ihrer persönlichen Lebensqualität nicht sehr weit auseinanderliegen. Sehr deutlich unterscheiden sich diese beiden Extremgruppen hingegen bei der Belästigung durch Straßenlärm, Nachbarschaftslärm, Lärm insgesamt und bei der Belästigung durch Erschütterungen:

Abbildung 92: Lebensqualität und Lärmbelästigung nach Quelle (gruppiert) im Mittelwertvergleich (aufsteigend sortiert nach Extremwertdifferenz)

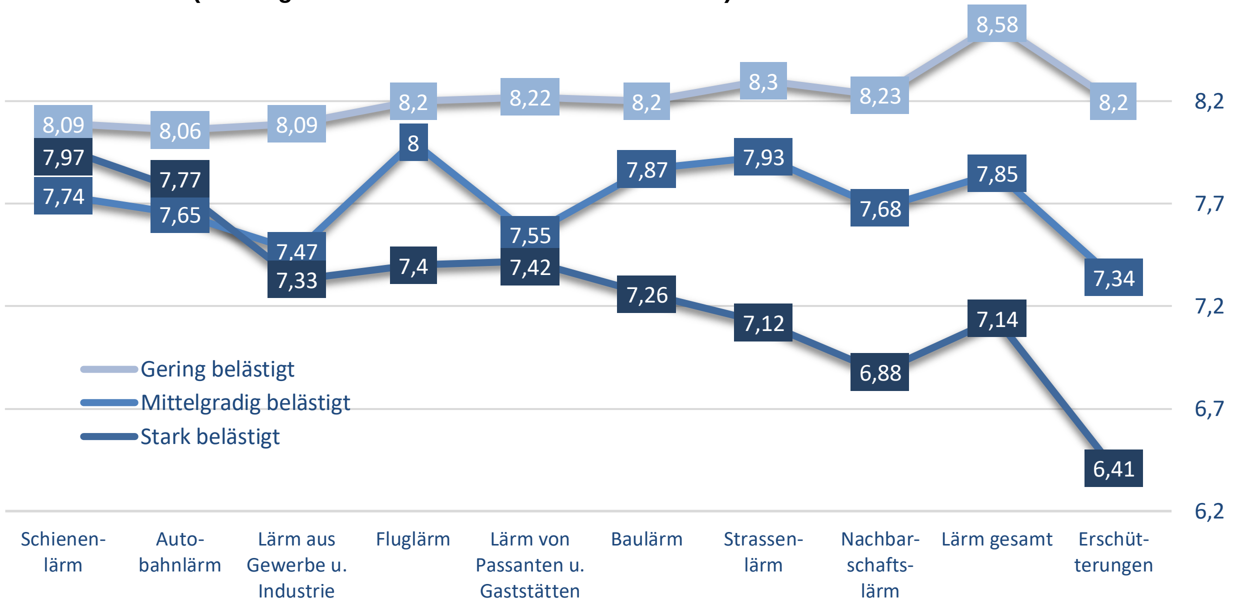

6. Lebensqualität und Belästigung durch Lärm im Schlaf

Die Anzahl an Personen, welche sich im Schlaf durch Lärm stärker gestört fühlen ist begrenzt. Die Belästigungen durch Schienenverkehrs-, Baustellen-, und Autobahnlärm in der Nacht zeigen kaum relevante Bezüge zur Einschätzung der eigenen Lebensqualität. Sehr deutlich treten Beziehungen hingegen bei Störungen durch den Lärm insgesamt, durch Nachbarschaftslärm, Lärm von Gewerbe und Industrie und die Störung durch Erschütterungen zutage. Vor allem bei den drei Letztgenannten wirkt sich das Belästigungsausmaß deutlich auf die Einschätzung der eigenen Lebensqualität aus:

Abbildung 93: Lebensqualität und Lärmbelästigung im Schlaf nach Quelle (gruppiert) im Mittelwertvergleich (aufsteigend sortiert nach Extremwertdifferenz)

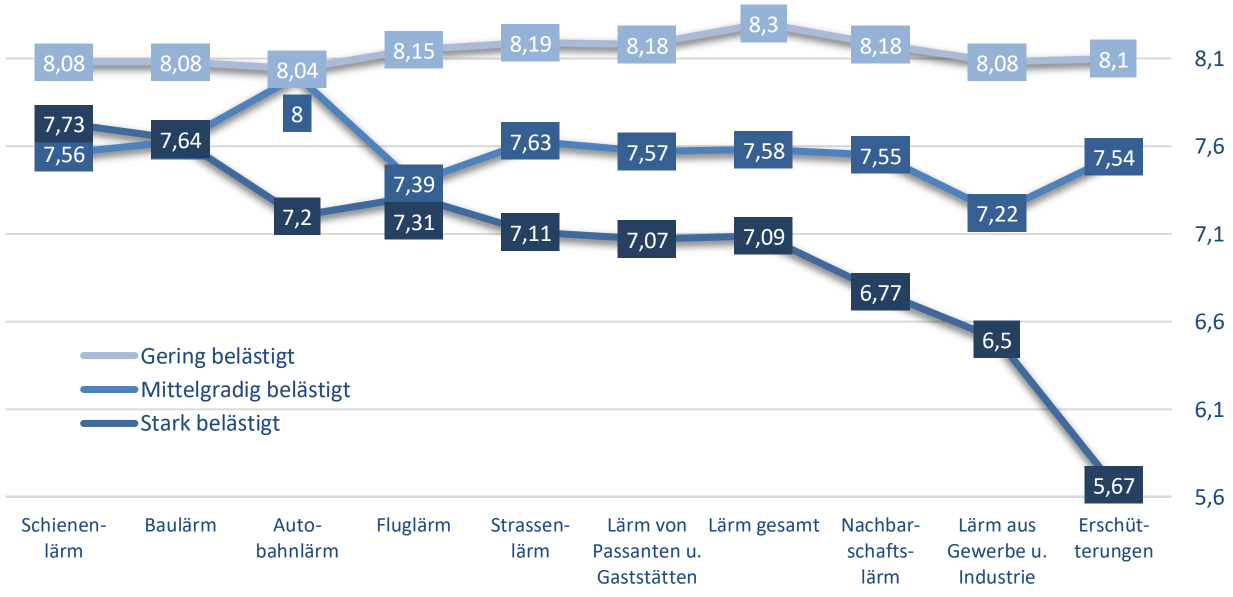

Lärmempfindlichkeit

1. Lärmempfindlichkeit im Überblick

Etwas mehr als ein Viertel aller Befragten schätzt sich selbst als überhaupt nicht bis kaum lärmempfindlich ein (unterste 3 Antwortkategorien), knapp 17% hingegen als stark bis außerordentlich lärmempfindlich (oberste 3 Antwortkategorien). Die Mehrheit der Befragten siedelt das Ausmaß der eigenen Lärmempfindlichkeit in den mittleren 5 Antwortkategorien an. Alle befragten Innsbruckerinnen und Innsbrucker und Innsbrucker machen Angaben zu dieser Frage:

Abbildung 94: Einschätzung der eigenen Lärmempfindlichkeit in Prozent

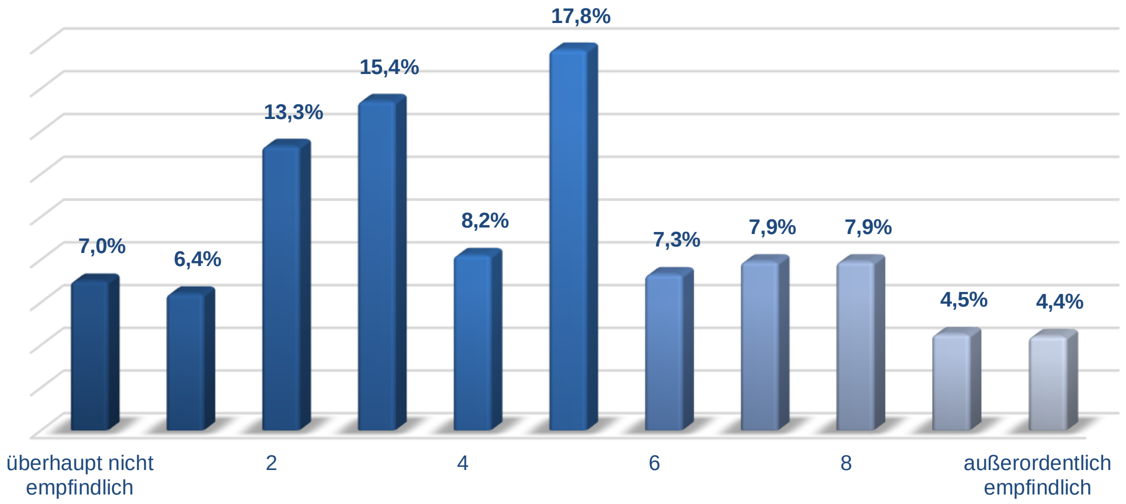

Der Mittelwert der Antworten liegt auf der 11-er Skala von *überhaupt nicht empfindlich* bis *außerordentlich empfindlich* bei 4,47 (Standardfehler 0,084), das entspricht einem durchschnittlichen Ausmaß der Einschätzung der eigenen Lärmempfindlichkeit der befragten Innsbruckerinnen und Innsbrucker von 49,17% vom Maximalwert.

Tabelle 82: Einschätzung der eigenen Lärmempfindlichkeit gruppiert

		Häufigkeit	Gültige Prozente	Kumulierte Prozente
Gültig	Nicht lärmempfindlich	275	26,7	26,7
	Durchschnittlich	584	56,6	83,3
	Sehr lärmempfindlich	172	16,7	100,0
	Gesamt	1031	100,0	

2. Lärmempfindlichkeit und ausgewählte Parameter

Frauen schätzen sich selbst häufiger und auch im Ausmaß stärker als lärmempfindlich ein als Männer.

Die Altersgruppe über 60 Jahren hat den größten Anteil an nicht bis kaum lärmempfindlichen Befragten. Insgesamt empfinden sich jedoch die Befragten der Altersgruppe der 18 bis 40-Jährigen am wenigsten lärmempfindlich, während in der Gruppe der 40 bis 60-Jährigen die meisten und am stärksten

Lärmempfindlichen enthalten sind. Insgesamt ist eine Zunahme der Häufigkeit und des Ausmaßes der Lärmempfindlichkeit mit steigendem Alter bis zur Schwelle des 65. bis 70. Lebensjahres festzustellen.

Sehr deutlich unterscheiden sich (auch altersbereinigt) erneut die Bildungsschichten: Akademikerinnen und Akademiker stufen sich im Vergleich mit Pflichtschulabgängerinnen und –Abgängern sowohl im Ausmaß als auch in der Häufigkeit in etwa doppelt so stark und häufig als sehr lärmempfindlich ein und umgekehrt.

Lärmempfindlichere Befragte schätzen die negativen Auswirkungen von Lärm auf die Gesundheit deutlich stärker ein als weniger Lärmempfindliche.

Probandinnen und Probanden mit selbstreferenziertem schlechteren Gesundheitszustand sind häufiger sehr lärmempfindlich als Befragte mit gutem Gesundheitszustand:

Tabelle 83: Einschätzung der eigenen Lärmempfindlichkeit (gruppiert) und ausgewählte Variablen

		Ausmaß eigene Lärmempfindlichkeit gruppiert					
		Kaum lärmempfindlich		Durchschnittlich lärmempfindlich		Sehr lärmempfindlich	
		Anzahl	Zeilen (%)	Anzahl	Zeilen (%)	Anzahl	Zeilen (%)
**Geschlecht**	weiblich	134	24,8%	306	56,6%	101	18,7%
	männlich	141	28,8%	278	56,7%	71	14,5%
**Altersgruppen**	18 bis 40 Jahre	120	27,3%	266	60,5%	54	12,3%
	41 bis 60 Jahre	77	24,4%	175	55,4%	64	20,3%
	über 60 Jahre	78	28,4%	143	52,0%	54	19,6%
**Bildungsschicht**	Primärer Schulabschluss	42	42,9%	47	48,0%	9	9,2%
	Sekundärer Schulabschluss	149	27,5%	313	57,7%	80	14,8%
	Tertiärer Schulabschluss	82	21,1%	223	57,5%	83	21,4%
**Einschätzung des Ausmaßes negativer Auswirkungen von Lärm auf die Gesundheit**	Wenig	73	33,6%	123	56,7%	21	9,7%
	Stark	188	24,1%	447	57,2%	146	18,7%
**Gesundheitszustand**	Gut	206	27,3%	426	56,5%	122	16,2%
	Zufriedenstellend	46	24,7%	112	60,2%	28	15,1%
	Nicht gut	23	25,8%	44	49,4%	22	24,7%
**Lebensqualität**	Nicht gut (*)	1	10,0%	6	60,0%	3	30,0%
	Durchschnittlich	67	23,1%	174	60,0%	49	16,9%
	Sehr gut	206	28,4%	400	55,2%	119	16,4%
**Lautheit Innsbruck im Vergleich zu anderen Städten Österreichs**	Ruhiger	100	34,2%	157	53,8%	35	12,0%
	Gleich	135	24,5%	315	57,2%	101	18,3%
	Lauter	38	21,8%	102	58,6%	34	19,5%

* zu kleine Fallzahlen für solide Auswertungen

Befragte Innsbruckerinnen und Innsbrucker mit hoher Lebensqualität sehen sich weniger häufig und in geringerem Ausmaß als lärmempfindlich an als Personen mit geringerer Lebensqualität.

Diejenigen Befragten, die davon ausgehen, dass es in Innsbruck im Vergleich zu anderen Städten Österreichs ruhiger ist, sind deutlich weniger häufig und weniger stark lärmempfindlich als Befragte, welche glauben, dass Innsbruck gleich laut oder lauter als andere vergleichbare Städte ist.

3. Lärmempfindlichkeit und Lärmbelastung

Das Ausmaß der Lärmempfindlichkeit der Befragten zeigt keine deutliche Beziehung zur tatsächlich vorhandenen Verkehrslärmbelastung im Wohnumfeld der Befragten:

Tabelle 84: Bivariate Korrelation zu Lärmempfindlichkeit und 24h-Lärmbelastung L_{den} nach Quelle

		Straßenlärm 24h	Schienenlärm 24h	Fluglärm 24h
Ausmaß eigene Lärmempfindlichkeit	Korrelation nach Pearson	-,053	-,028	-,002
	Signifikanz (2-seitig)	,090	,370	,959
	N	1031	1031	1031

Ebenso wenig im Mittelwertvergleich zum Straßenverkehrslärm:

Tabelle 85: Lärmempfindlichkeit und 24h-Straßenlärm L_{den} in 10-dB Schritten im Mittelwertvergleich

Straßenlärm 24h	Mittelwert	N	Standardabweichung
unter 30dB (*)	1,5000	2	,70711
30 bis 40dB	2,0769	26	,56022
40 bis 50dB	2,0205	195	,65770
50 bis 60dB	1,8343	531	,65216
über 60dB	1,9278	277	,63860
Insgesamt	1,9001	1031	,65115

* zu kleine Fallzahlen für solide Auswertungen

Auch für den Flugverkehrslärm sind keine eindeutigen statistischen Korrelationen nachweisbar:

Tabelle 86: Lärmempfindlichkeit und 24h-Fluglärm L_{den} in 10-dB Schritten im Mittelwertvergleich

Fluglärm 24h	Mittelwert	N	Standardabweichung
30 bis 40dB	1,9424	243	,63374
40 bis 50dB	1,8760	621	,66654
50 bis 60dB	1,9111	135	,61652
über 60dB	2,0000	32	,62217
Insgesamt	1,9001	1031	,65115

Dasselbe gilt für den Schienenverkehrslärm:

Tabelle 87: Lärmempfindlichkeit und 24h-Schienenlärm L_{den} in 10-dB Schritten im Mittelwertvergleich

Schienenlärm 24h	Mittelwert	N	Standardabweichung
unter 30dB	2,0000	15	,53452
30 bis 40dB	1,9120	284	,65873
40 bis 50dB	1,9072	485	,64415
50 bis 60dB	1,8565	209	,65667
über 60dB	1,9211	38	,71212
Insgesamt	1,9001	1031	,65115

Für die Zusammenschau der persönlichen Lärmempfindlichkeit mit den tatsächlich gegebenen Verkehrslärmpegeln in der Nacht lassen sich ebenso wie für den 24h-Zeitraum keine eindeutigen statistischen Beziehungen herstellen.

4. Lärmempfindlichkeit und Lärmexpositionsempfinden

Befragte, welche sich stärker lärmexponiert fühlen, schätzen sich auch häufiger als lärmempfindlicher ein und umgekehrt. Die Zusammenhänge sind jedoch weniger ausgeprägt als etwa bei den Beziehungen zur Lebensqualität:

Tabelle 88: Lärmempfindlichkeit und Lärmexpositionsempfinden nach Quelle (gruppier)

		**Ausmaß eigene Lärmempfindlichkeit gruppiert**					
		**Nicht lärmempfindlich**		**Durchschnittlich**		**Sehr lärmempfindlich**	
		Anzahl	Zeilen (%)	Anzahl	Zeilen (%)	Anzahl	Zeilen (%)
**Ausgesetztheit Lärm gesamt**	Gering exponiert	100	38,3%	115	44,1%	46	17,6%
	Mittelgradig exponiert	150	23,8%	385	61,1%	95	15,1%
	Stark exponiert	25	17,9%	84	60,0%	31	22,1%
**Ausgesetztheit Fluglärm**	Gering exponiert	134	33,6%	217	54,4%	48	12,0%
	Mittelgradig exponiert	105	22,5%	278	59,5%	84	18,0%
	Stark exponiert	36	21,8%	89	53,9%	40	24,2%
**Ausgesetztheit Strassenlärm**	Gering exponiert	142	33,0%	216	50,2%	72	16,7%
	Mittelgradig exponiert	111	23,9%	277	59,7%	76	16,4%
	Stark exponiert	22	16,1%	91	66,4%	24	17,5%
**Ausgesetztheit Baulärm**	Gering exponiert	191	28,4%	383	56,9%	99	14,7%
	Mittelgradig exponiert	61	24,3%	138	55,0%	52	20,7%
	Stark exponiert	23	21,5%	63	58,9%	21	19,6%
**Ausgesetztheit Nachbarschaftslärm**	Gering exponiert	204	28,7%	403	56,7%	104	14,6%
	Mittelgradig exponiert	53	20,6%	150	58,4%	54	21,0%
	Stark exponiert	18	28,6%	31	49,2%	14	22,2%
**Ausgesetztheit Lärm von Passanten**	Gering exponiert	194	27,2%	401	56,2%	118	16,5%
	Mittelgradig exponiert	65	25,3%	148	57,6%	44	17,1%

**u. Gaststätten**	Stark exponiert	16	26,2%	35	57,4%	10	16,4%
**Ausgesetztheit Erschütterungen**	Gering exponiert	233	28,9%	446	55,3%	127	15,8%
	Mittelgradig exponiert	32	17,5%	114	62,3%	37	20,2%
	Stark exponiert	10	23,8%	24	57,1%	8	19,0%
**Ausgesetztheit Schienenlärm**	Gering exponiert	234	27,7%	475	56,2%	136	16,1%
	Mittelgradig exponiert	32	20,4%	93	59,2%	32	20,4%
	Stark exponiert	9	31,0%	16	55,2%	4 (*)	13,8%
**Ausgesetztheit Autobahnlärm**	Gering exponiert	259	27,8%	524	56,3%	148	15,9%
	Mittelgradig exponiert	12	14,8%	48	59,3%	21	25,9%
	Stark exponiert	4 (*)	21,1%	12	63,2%	3 (*)	15,8%
**Ausgesetztheit Lärm von Gewerbe und Industrie**	Gering exponiert	256	27,5%	523	56,1%	153	16,4%
	Mittelgradig exponiert	17	21,5%	46	58,2%	16	20,3%
	Stark exponiert	2 (*)	10,0%	15	75,0%	3 (*)	15,0%

* zu kleine Fallzahlen für solide Auswertungen

5. Lärmempfindlichkeit und Lärmbelästigung

Die Selbsteinschätzung der eigenen Lärmempfindlichkeit zeigt eine deutliche wechselseitige Beziehung zur empfundenen Lärmbelästigung:

Abbildung 95: Lärmempfindlichkeit und Belästigung durch Lärm insgesamt im Mittelwertvergleich

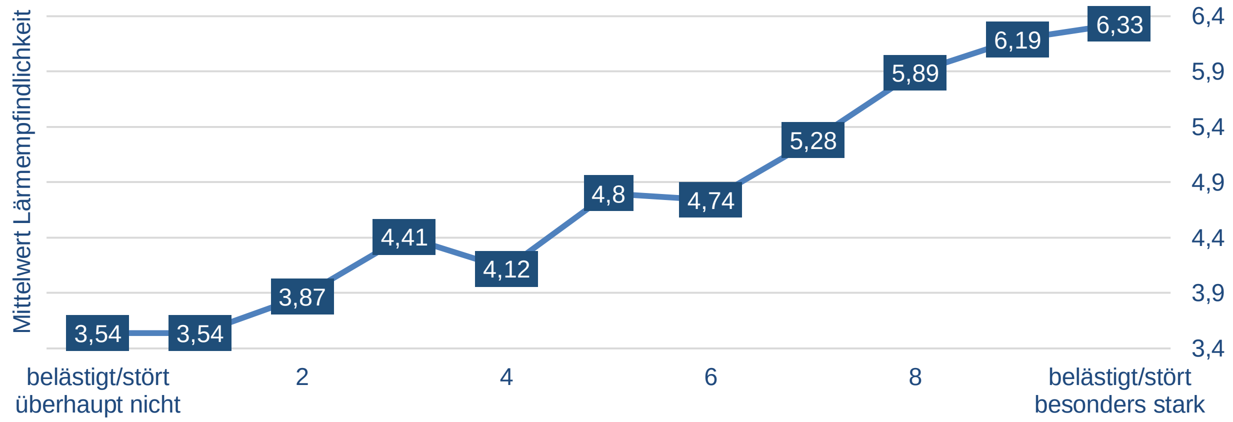

Stark lärmempfindliche Befragte fühlen sich häufiger und stärker vom Lärm belästigt und sich stark vom Lärm gestört empfindende Befragte schätzen sich selbst als lärmempfindlicher ein.

Die Unterschiede im Antwortverhalten sind besonders im Vergleich der Extremgruppen beim Straßenverkehrs- und Fluglärm, sowie dem Lärm insgesamt sehr stark ausgeprägt, während beim nichtverkehrsverursachten Lärm, sowie dem Schienenverkehrslärm die Extremgruppen geringer voneinander abweichen:

Abbildung 96: Lärmempfindlichkeit und Belästigung durch Lärm nach Quelle (gruppiert) im Mittelwertvergleich (aufsteigend sortiert nach Extremwertdifferenz)

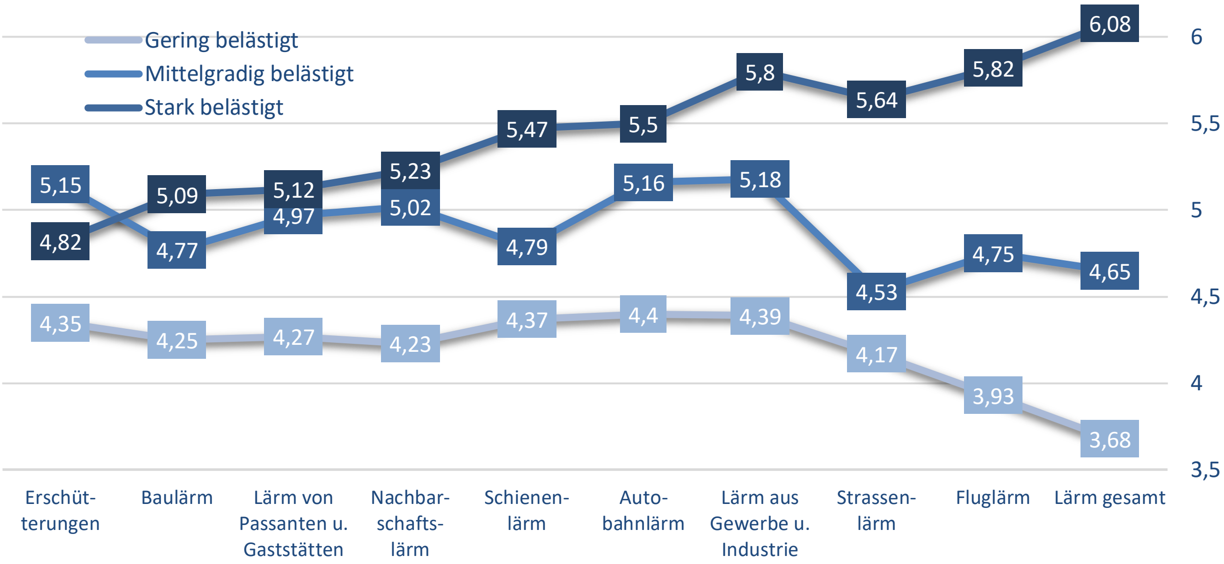

Aus den Auswertungen geht hervor, dass die Einschätzung der Lärmempfindlichkeit insgesamt stark auf den Verkehrslärm referenziert.

Tabelle 89: Lärmempfindlichkeit und Belästigung durch Verkehrslärm nach Quelle gruppiert

		Ausmaß eigene Lärmempfindlichkeit gruppiert					
		Nicht lärmempfindlich		Durchschnittlich		Sehr lärmempfindlich	
		Anzahl	Zeilen (%)	Anzahl	Zeilen (%)	Anzahl	Zeilen (%)
**Belästigung Lärm gesamt**	Gering belästigt	159	42,1%	168	44,4%	51	13,5%
	Mittelgradig belästigt	103	19,5%	346	65,5%	79	15,0%
	Stark belästigt	13	10,4%	70	56,0%	42	33,6%
**Belästigung Fluglärm**	Gering belästigt	179	35,1%	267	52,4%	64	12,5%
	Mittelgradig belästigt	82	20,5%	245	61,3%	73	18,3%
	Stark belästigt	14	11,6%	72	59,5%	35	28,9%
**Belästigung Straßenlärm**	Gering belästigt	177	32,8%	274	50,8%	88	16,3%
	Mittelgradig belästigt	85	22,7%	234	62,4%	56	14,9%
	Stark belästigt	13	11,1%	76	65,0%	28	23,9%
**Belästigung Schienenlärm**	Gering belästigt	237	28,2%	468	55,7%	135	16,1%
	Mittelgradig belästigt	35	21,7%	96	59,6%	30	18,6%
	Stark belästigt	3	10,0%	20	66,7%	7	23,3%

Abbildung 97: **Lärmempfindlichkeit und Belästigung durch Verkehrslärm nach Quelle (gruppiert) im Mittelwertvergleich**

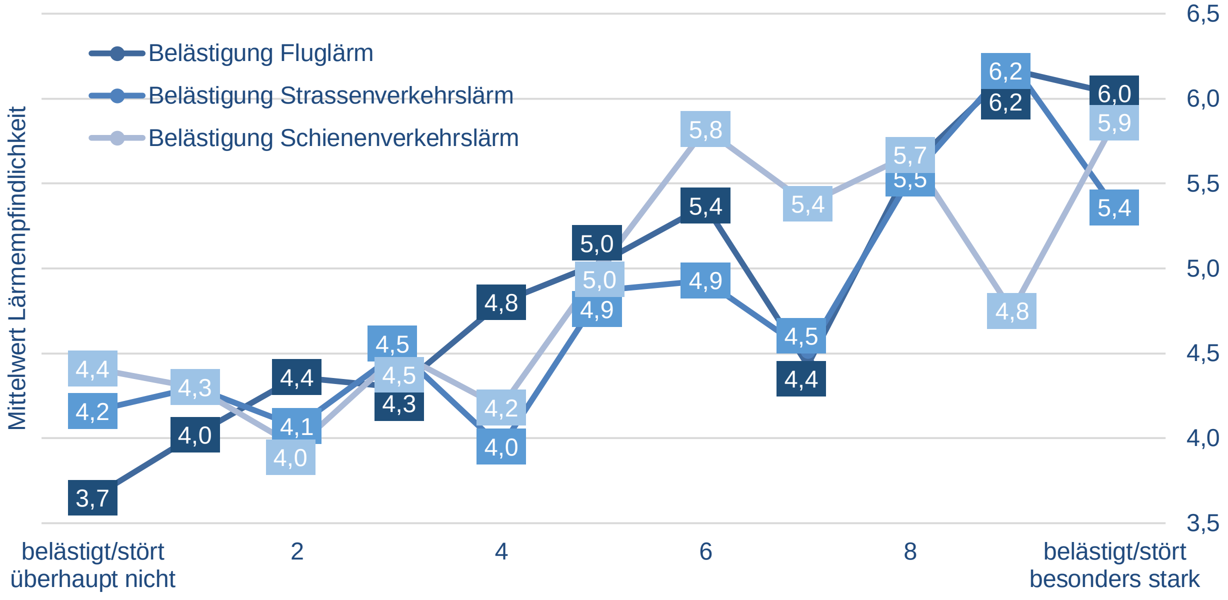

6. Lärmempfindlichkeit und Belästigung durch Lärm im Schlaf

Auch in Beziehung der Einschätzung der eigenen Lärmempfindlichkeit zur empfundenen Lärmbelästigung im Schlaf nach Quelle zeigt sich die Orientierung der Lärmempfindlichkeit am Verkehrslärm. Die Unterschiede zwischen den Extremgruppen fallen sehr deutlich auS. Eine Auffälligkeit tritt in der Zusammenschau der Lärmempfindlichkeit mit der Belästigung durch Lärm aus Gewerbe und Industrie zutage. Die 10 befragten Innsbruckerinnen und Innsbrucker, welche sich stark vom Lärm aus Gewerbe und Industrie im Schlaf gestört fühlen, schätzen sich selbst als sehr lärmempfindlich ein und sorgen für einen außertourlichen Spitzenwert in der Kombination dieser beiden Variablen:

Abbildung 98: **Lärmempfindlichkeit und Belästigung durch Lärm im Schlaf nach Quelle (gruppiert) im Mittelwertvergleich (aufsteigend sortiert nach Extremwertdifferenz)**

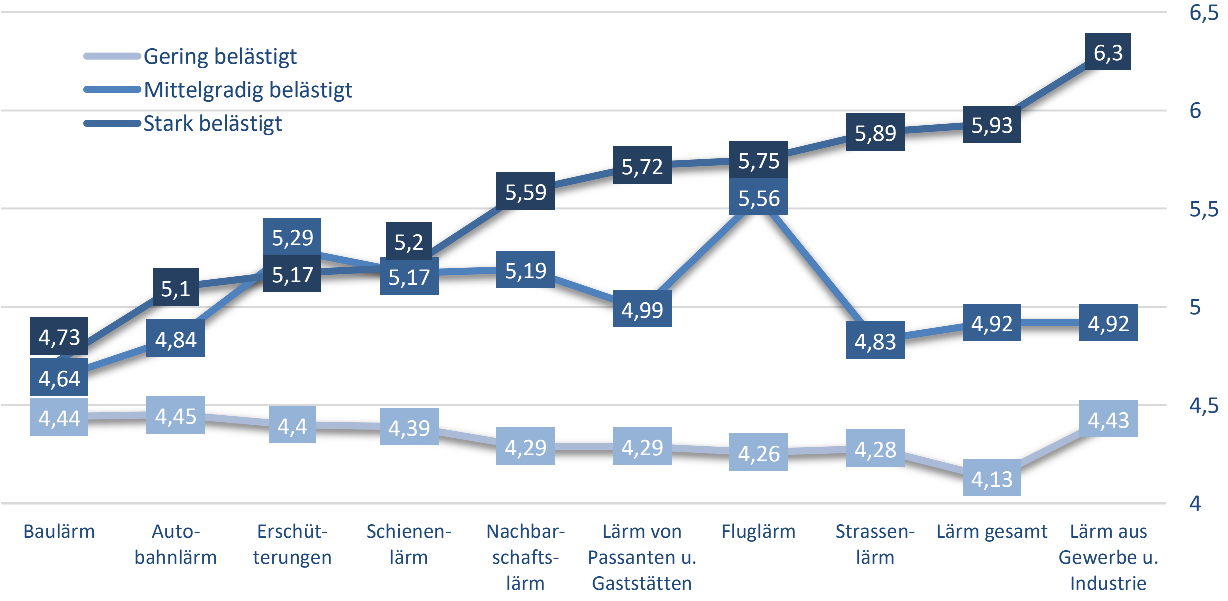

Wind- und Wettereinflüsse auf die Lärmbelastung

1. Wind- und Wettereinflüsse auf den Lärm im Überblick

Zwei Drittel aller befragten Innsbruckerinnen und Innsbrucker glauben, dass die Wind- und Wetterverhältnisse sich g*ar nicht* oder *eher wenig* auf die Lärmbelastung auswirken. 38 Befragte geben an, dass sie diese Frage nicht beurteilen können und 30% der Probandinnen und Probanden sind der Meinung, dass sich die Wind- und Wetterverhältnisse *eher stark* bis *sehr stark* auf die Lärmbelastung insgesamt auswirken:

Abbildung 99: Ausmaß des Einflusses von Wind- und Wetterverhältnissen auf die Lärmbelastung

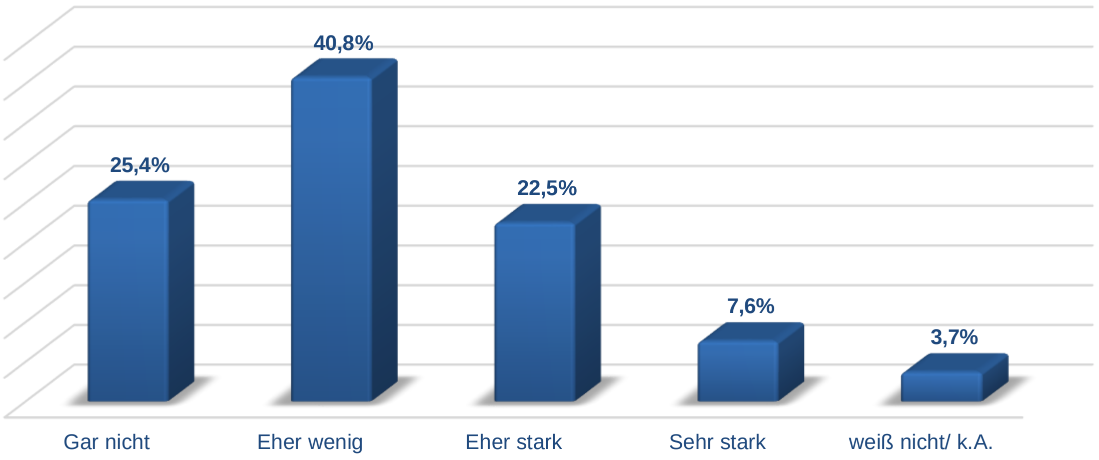

Tabelle 90: Einfluss von Wind- und Wetterverhältnissen auf die Lärmbelastung gruppiert

		Häufigkeit	Gültige Prozente	Kumulierte Prozente
Gültig	Gering	683	66,2	66,2
	Stark	310	30,1	96,3
	k.A. / w.n.	38	3,7	100,0
	Gesamt	1031	100,0	

2. Wind- und Wettereinflüsse und ausgewählte Parameter

Frauen gehen von einem geringfügig stärkeren Einfluss der Wind- und Wetterverhältnissen auf die Lärmbelastung aus als Männer. Ebenso die 40- bis 60-Jährigen im Vergleich zu den beiden anderen Altersgruppen.

Eine deutliche Funktion zeigt sich in Abhängigkeit von der Bildungsschicht: Je höher der bisher erreichte höchste Bildungsabschluss, desto eher (auch altersbereinigt) gehen Befragte von einem stärkeren Einfluss der Wind- und Wetterverhältnissen auf die Lärmbelastung aus und umgekehrt:

Tabelle 91: Einfluss von Wind- und Wetterverhältnissen auf die Lärmbelastung und ausgewählte Parameter (gruppiert, ohne Antwortkategorie k.A./w.n.)

		Ausmaß Windeinfluss auf Lärm (gruppiert)			
		Gering		Stark	
		Anzahl	Zeilen (%)	Anzahl	Zeilen (%)
**Geschlecht**	weiblich	347	66,9%	172	33,1%
	männlich	336	70,9%	138	29,1%
**Altersgruppen**	18 bis 40 Jahre	289	69,6%	126	30,4%
	41 bis 60 Jahre	197	63,5%	113	36,5%
	über 60 Jahre	197	73,5%	71	26,5%
**Bildungsschicht**	Primärer Schulabschluss	73	78,5%	20	21,5%
	Sekundärer Schulabschluss	372	71,4%	149	28,6%
	Tertiärer Schulabschluss	236	62,8%	140	37,2%
**Baulicher Schutz vor Verkehrslärm**	Keine bis geringfügige Abschirmung vorhanden	493	67,0%	243	33,0%
	Gute bis komplette Abschirmung vorhanden	169	73,8%	60	26,2%
**Schutz vor Verkehrslärm durch Innenhof etc.**	Ja	490	72,7%	184	27,3%
	Nein	180	60,6%	117	39,4%
	weiß nicht/ k.A.	13	59,1%	9	40,9%
**Nachtschlaf**	Fenster nachts geöffnet/gekippt	543	68,6%	248	31,4%
	Fenster nachts geschlossen	134	69,1%	60	30,9%
**Lärmschutzfenster in den letzten 10 Jahren eingebaut**	Ja	120	65,2%	64	34,8%
	Nein	437	67,1%	214	32,9%
	weiß nicht/ k.A.	126	79,7%	32	20,3%
**Lautheit Innsbruck im Vergleich zu anderen Städten**	Ruhiger	198	70,5%	83	29,5%
	Gleich	369	69,9%	159	30,1%
	Lauter	105	61,4%	66	38,6%
**Straßenlärm 24h**	Stark belastet >55dB	334	67,7%	159	32,3%
	Mittelgradig belastet 45-55dB	272	69,4%	120	30,6%
	Gering belastet <45dB	77	71,3%	31	28,7%
**Ausgesetztheit Lärm gesamt**	Gering exponiert	199	78,3%	55	21,7%
	Mittelgradig exponiert	408	67,3%	198	32,7%
	Stark exponiert	76	57,1%	57	42,9%
**Belästigung Lärm gesamt**	Gering belästigt	279	76,4%	86	23,6%
	Mittelgradig belästigt	341	67,3%	166	32,7%
	Stark belästigt	63	52,1%	58	47,9%

Befragte in Wohnungen mit keinen bis nur geringfügigen baulichen Abschirmungen gegen den Verkehrslärm gehen von stärkeren Wettereinflüssen auf die Lärmbelastung aus, als gut bis komplett abgeschirmt wohnende Probandinnen und Probanden. Dasselbe gilt für Befragte, die in ihrem Wohnumfeld über eine lärmabgewandte Seite oder einen lärmgeschützten Innenhof verfügen.

Ob die Befragten nachts bei offenem / gekipptem oder geschlossenem Fenster schlafen bzw. ob in den letzten 10 Jahren Lärmschutzfenster verbaut wurden, zeigt keine Zusammenhänge zur Einschätzung des Einflusses von Wind- und Wetterverhältnissen auf die Lärmbelastung.

Auch das Ausmaß des vorhandenen Straßenlärms zeigt keine großen Unterschiede zwischen den einzelnen Belastungsgruppen und deren Einschätzung des Einflusses von Wind- und Wetterverhältnissen auf die Lärmbelastung. In Beziehung zum Ausmaß des vorhandenen Fluglärms sind ebenfalls nur geringe Unterschiede zwischen den Extremgruppen feststellbar.

Deutlich hingegen wirken die Lärmwahrnehmung und die psychologische Disposition: Sich stark dem Lärm ausgesetzt oder von diesem belästigt empfindende Befragte gehen von einem stärkeren Einfluss der Wind- und Wetterverhältnissen auf die Lärmbelastung aus, als sich weniger stark exponiert oder belästigt empfindende Innsbruckerinnen und Innsbrucker. Etwas eingeschränkter gilt dasselbe auch für die Beziehungen zum Ausmaß der Einschätzung der eigenen Lärmempfindlichkeit.

Nachtschlaf bei geöffnetem Fenster

1. Nachtschlaf bei geöffnetem Fenster im Überblick

Fast 50% aller befragten Innsbruckerinnen und Innsbrucker schlafen nachts *häufig bis immer* bei geöffnetem oder gekippten Fenster, weitere 30% tun dies zumindest *gelegentlich*. Nur 0,7% der Befragten verfügen über eine *Wohnraumlüftung*, 6,6% möchten nicht bei geöffnetem oder gekipptem Fenster schlafen und für 12,3% wäre dies *zu laut*. 8 Befragte machen keine Angaben:

Abbildung 100: Nachtschlaf bei geöffnetem oder gekipptem Fenster in Prozent

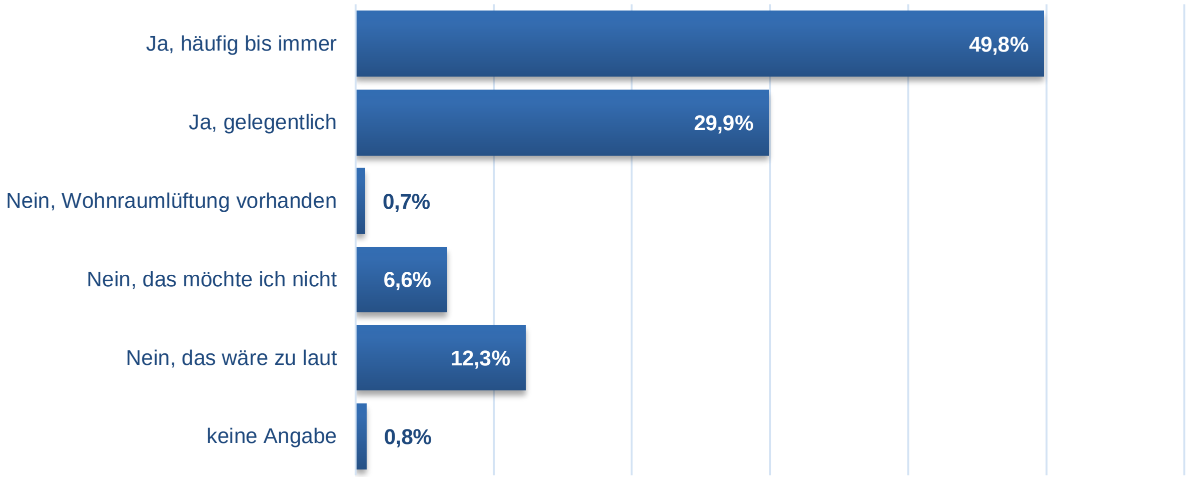

Tabelle 92: Nachtschlaf bei geöffnetem oder gekipptem Fenster gruppiert

		Häufigkeit	Gültige Prozente	Kumulierte Prozente
Gültig	Fenster nachts geöffnet/gekippt	821	79,6	79,6
	Fenster nachts geschlossen	202	19,6	99,2
	Keine Angabe	8	,8	100,0
	Gesamt	1031	100,0	

2. Nachtschlaf und ausgewählte Parameter

Zwischen den Geschlechtern zeigen sich adjustiert nur geringe Unterschiede im Schlafverhalten. Auch die einzelnen Altersgruppen und die Bildungsschicht betreffend sind keine großen Unterschiede sichtbar.

Wie zu erwarten, schlafen Befragte, die über einen lärmgeschützten Innenhof, eine lärmabgewandte Seite oder gute bauliche Abschirmungen gegen den Verkehrslärm verfügen, häufiger bei geöffnetem oder gekipptem Fenster als Befragte, die über keinerlei oder wenig baulichen Lärmschutz verfügen. Die Unterschiede sind allerdings wohl kleiner als erwartet und der reine bauliche Lärmschutz an sich scheint nicht das ausschlaggebende Element für die Entscheidung der Befragten, ob sie bei geöffnetem oder geschlossenem Fenster schlafen, zu sein:

Tabelle 93: Nachtschlaf bei geöffnetem oder gekipptem Fenster und ausgewählte Parameter (gruppiert, ohne Antwortkategorie k.A./w.n.)

		Nachtschlaf bei offenem oder gekippten Fenster gruppiert			
		Fenster nachts geöffnet/gekippt		Fenster nachts geschlossen	
		Anzahl	Zeilen (%)	Anzahl	Zeilen (%)
**Geschlecht**	weiblich	421	78,3%	117	21,7%
	männlich	400	82,5%	85	17,5%
**Altersgruppen**	18 bis 40 Jahre	342	78,3%	95	21,7%
	41 bis 60 Jahre	255	81,2%	59	18,8%
	über 60 Jahre	224	82,4%	48	17,6%
**Bildungsschicht**	Primärer Schulabschluss	79	80,6%	19	19,4%
	Sekundärer Schulabschluss	435	80,9%	103	19,1%
	Tertiärer Schulabschluss	305	79,4%	79	20,6%
**Baulicher Schutz vor Verkehrslärm**	Keine bis geringfügige Abschirmung vorhanden	602	79,2%	158	20,8%
	Gute bis komplette Abschirmung vorhanden	196	83,4%	39	16,6%
**Schutz vor Verkehrslärm durch Innenhof etc.**	Ja	552	79,3%	144	20,7%
	Nein	250	81,7%	56	18,3%
	weiß nicht/ k.A.	19	90,5%	2	9,5%
**Lärmschutzfenster in den letzten 10 Jahren eingebaut**	Ja	134	72,4%	51	27,6%
	Nein	552	82,6%	116	17,4%
	weiß nicht/ k.A.	135	79,4%	35	20,6%
**Lautheit Innsbruck im Vergleich zu anderen Städten**	Ruhiger	234	81,3%	54	18,8%
	Gleich	441	80,5%	107	19,5%
	Lauter	133	76,9%	40	23,1%
**Ausgesetztheit Lärm gesamt**	Gering exponiert	226	87,9%	31	12,1%
	Mittelgradig exponiert	508	81,0%	119	19,0%
	Stark exponiert	87	62,6%	52	37,4%
**Belästigung Lärm gesamt**	Gering belästigt	329	88,0%	45	12,0%
	Mittelgradig belästigt	410	78,1%	115	21,9%
	Stark belästigt	82	66,1%	42	33,9%
**Lärmempfindlichkeit**	Nicht lärmempfindlich	232	84,7%	42	15,3%
	Durchschnittlich	460	79,6%	118	20,4%
	Sehr lärmempfindlich	129	75,4%	42	24,6%
**Lebensbedingungen (Summenscore)**	Nicht gut	3 (*)	60,0%	2 (*)	40,0%
	Durchschnittlich	519	77,8%	148	22,2%
	Sehr gut	299	85,2%	52	14,8%

* zu geringe Fallzahlen für solide Auswertungen

Befragte, die sich als stark lärmempfindlich einstufen, dem Lärm stark ausgesetzt sehen, ihre Lebens- und Wohnbedingungen schlechter einstufen, sich vom Lärm stark belästigt fühlen, oder der Meinung sind, es sei in Innsbruck lauter als in anderen vergleichbaren österreichischen Städten schlafen deutlich häufiger bei geschlossenen Fenstern, als jene die sich weniger ausgesetzt und/oder gestört fühlen bzw. weniger lärmempfindlich sind etc. Jedoch schlafen auch in diesen Extremgruppen rund zwei Drittel häufig bis immer bei geöffneten / gekippten Fenstern.

Deutlich zeigt sich der Zusammenhang zur gegebenen Straßenverkehrslärmbelastung:

Abbildung 101: Nachtschlaf bei geöffnetem oder gekipptem Fenster und nächtliche Straßenverkehrslärmbelastung L_n in 10-db Schritten im Mittelwertvergleich

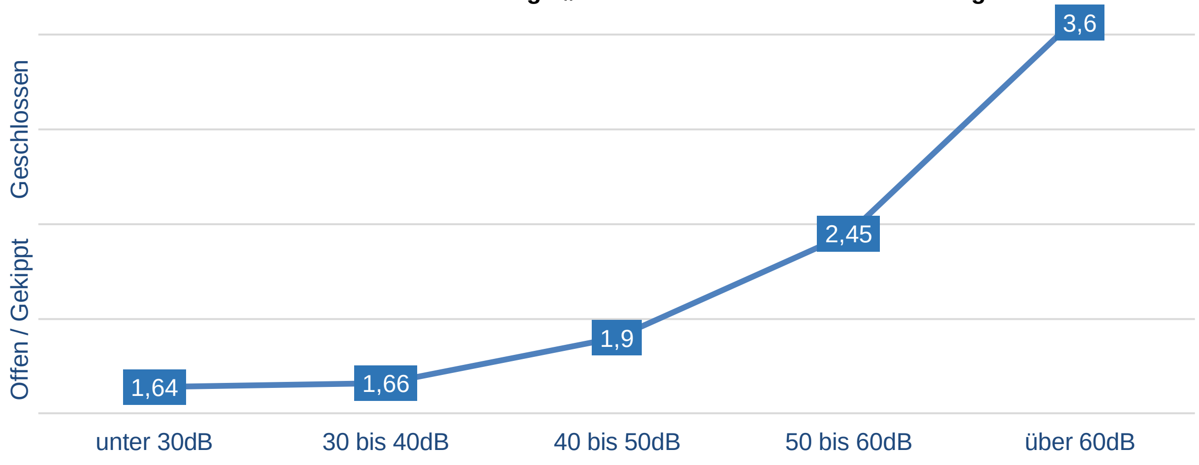

Die Flugverkehrslärmpegel in der Nacht fallen sehr niedrig aus und haben keinen eindeutigen Einfluss auf das Schlafverhalten. Der Anteil an Befragten, die mit geöffnetem / gekipptem Fenster schlafen, entspricht dem Gesamtkollektiv.

Interessant ist die Zusammenschau mit den nächtlichen Schienenverkehrslärmbelastungen: Diese weisen (auch statistisch) keinen starken Zusammenhang zum Schlafverhalten der Innsbruckerinnen und Innsbrucker auf und auch höhere Lärmpegel in der Nacht führen nur zu einem vergleichsweise geringfügig geänderten Schlafverhalten in Bezug auf geöffnete oder geschlossene Fenster:

Tabelle 94: Nachtschlaf bei geöffnetem oder gekipptem Fenster und nächtliche Lärmbelastung L_n nach Quelle (gruppiert)

		Nachtschlaf bei offenem oder gekippten Fenster gruppiert			
		Fenster nachts geöffnet/gekippt		Fenster nachts geschlossen	
		Anzahl	Zeilen (%)	Anzahl	Zeilen (%)
**Straßenlärm 24h**	Stark belastet >55dB	372	72,7%	140	27,3%
	Mittelgradig belastet 45-55dB	345	86,3%	55	13,8%
	Gering belastet <45dB	104	93,7%	7	6,3%
**Straßenlärm Nacht**	Stark belastet >55dB	67	63,8%	38	36,2%
	Mittelgradig belastet 45-55dB	337	75,9%	107	24,1%
	Gering belastet <45dB	417	88,0%	57	12,0%
**Autobahnlärm Nacht**	Stark belastet >55dB	2 (*)	100,0%	0 (*)	0,0%
	Mittelgradig belastet 45-55dB	52	83,9%	10	16,1%
	Gering belastet <45dB	767	80,0%	192	20,0%

	Stark belastet >55dB	15	75,0%	5	25,0%
**Schienenlärm Nacht**	Mittelgradig belastet 45-55dB	126	74,1%	44	25,9%
	Gering belastet <45dB	680	81,6%	153	18,4%
	Stark belastet >55dB	0 (*)	0,0%	0 (*)	0,0%
**Fluglärm Nacht**	Mittelgradig belastet 45-55dB	4 (*)	100,0%	0 (*)	0,0%
	Gering belastet <45dB	817	80,2%	202	19,8%

* zu geringe Fallzahlen für solide Auswertungen

Lärmexpositionsempfinden

1. Überblick empfundene Lärmexposition

1.1. Lärmexposition nach Lärmquelle

Die Einschätzung der Ausgesetztheit der eigenen Wohnung / des eigenen Hauses wurde für jede einzelne Lärmquelle mittels einer 11-teiligen Skala von *gar nicht* bis *äußerst stark* abgefragt.

Im Mittelwertvergleich zeigt sich, dass die Exposition dem Flugverkehrslärm gegenüber im Mittel am höchsten eingeschätzt wird, gefolgt von der Ausgesetztheit gegenüber Straßenverkehrslärm:[9]

Abbildung 102: Mittelwertvergleich der empfundenen Exposition nach Quelle

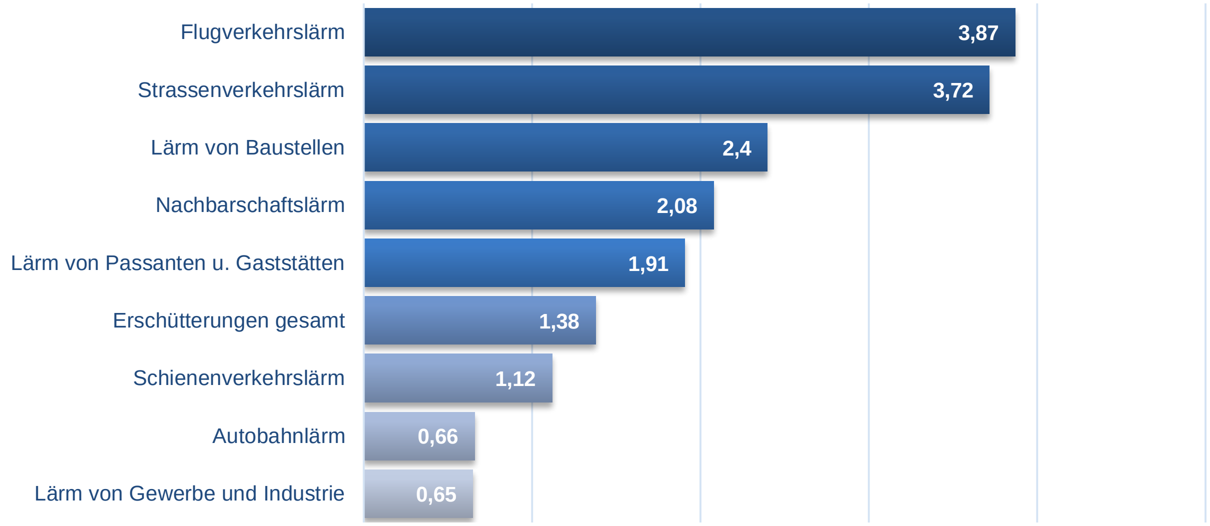

Erst mit deutlichem Abstand folgen Bau- und Nachbarschaftslärm, sowie die weiteren erfassten Lärmquellen und die Erschütterungsexposition. Die Ausgesetztheit der eigenen Wohnung / des eigenen Hauses gegenüber Schienenverkehrs- und Autobahnlärm, sowie dem Lärm von Gewerbe und Industrie ist für die befragten Innsbruckerinnen und Innsbrucker im Durchschnitt nur gering ausgeprägt:

Tabelle 95: Mittelwerte der empfundenen Exposition nach Quelle

	Flug-verkehr	Strassen-verkehr	Bau-stellen	Nachbar-schaft	Passanten u. Gast-stätten	Erschüt-terungen gesamt	Schienen verkehr	Autobahn	Gewerbe und Industrie
Mittelwert	3,87	3,72	2,40	2,08	1,91	1,38	1,12	,66	,65
N	1031	1031	1031	1031	1031	1031	1031	1031	1031
Standard-abweichung	2,943	2,921	3,074	2,581	2,622	2,380	2,208	1,711	1,735
Standardfehler des MW	,092	,091	,096	,080	,082	,074	,069	,053	,054
Minimum	gar nicht	gar nicht	gar nicht	gar nicht	gar nicht	gar nicht	gar nicht	gar nicht	gar nicht
Maximum	äußerst stark	äußerst stark	äußerst stark	äußerst stark	äußerst stark	äußerst stark	äußerst stark	äußerst stark	äußerst stark

[9] Anm.: Die eigenständige Frage nach der empfundenen Ausgesetztheit des eigenen Hauses / der eigenen Wohnung gegenüber *Lärm insgesamt* wird in den folgenden vergleichenden Zusammenstellungen der einzelnen Lärmquellen ausgeklammert und im anschließenden Unterkapitel dargestellt.

Der Mittelwert aller Antworten über alle 9 Fragen hinweg liegt bei 1,98 (21,8%).

Kategorisiert in 3 Expositionsgruppen:

- ***gering ausgesetzt****:* gruppierte Antwortkategorien 0 [=*gar nicht*]), 1 und 2
- ***mittelgradig ausgesetzt***: gruppierte Antwortkategorien 3 bis 7
- ***stark ausgesetzt (highly exposed)****:* gruppierte Antwortkategorien 8, 9 u. 10 [=*äußerst stark*])

stellt sich die empfundene Exposition der eigenen Wohnung / des eigenen Hauses wie folgt dar:

Abbildung 103: Empfundene Exposition nach Quelle kategorisiert in 3 Expositionsgruppen in Prozent

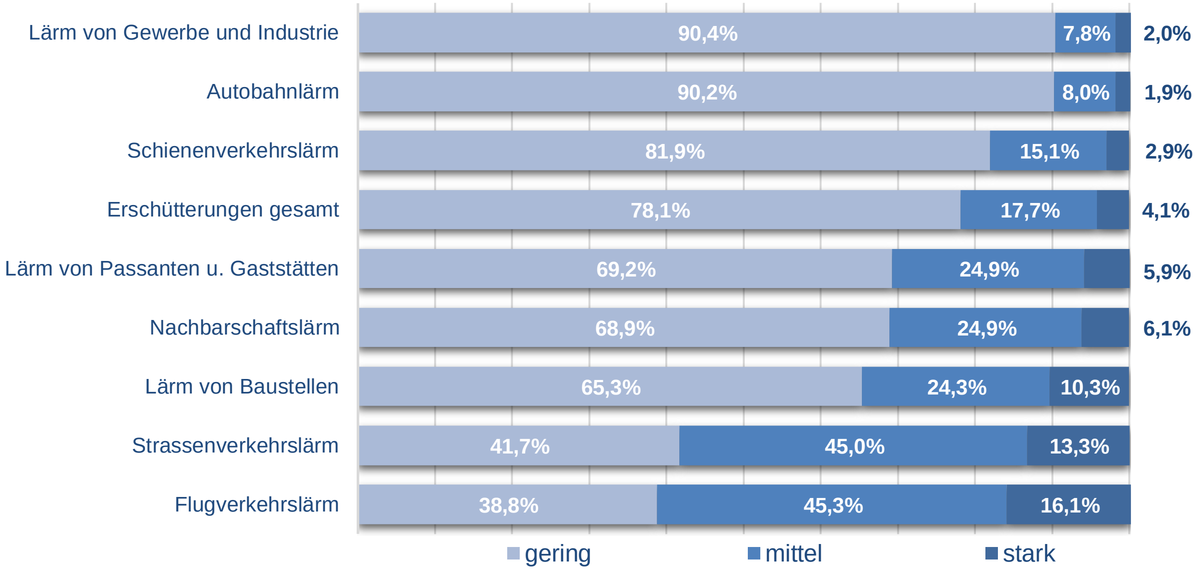

* Nach empfundenem Expositionsausmaß (Mittelwert) aufsteigend sortiert

Wird nur die Extremgruppe der sich stark ausgesetzt fühlenden Befragten (*highly exposed) betrachtet,* zeigt sich im Vergleich der Quellen ein nahezu unverändertes Bild:

Abbildung 104: Extremgruppenvergleich *highly exposed* nach Quelle in Prozent

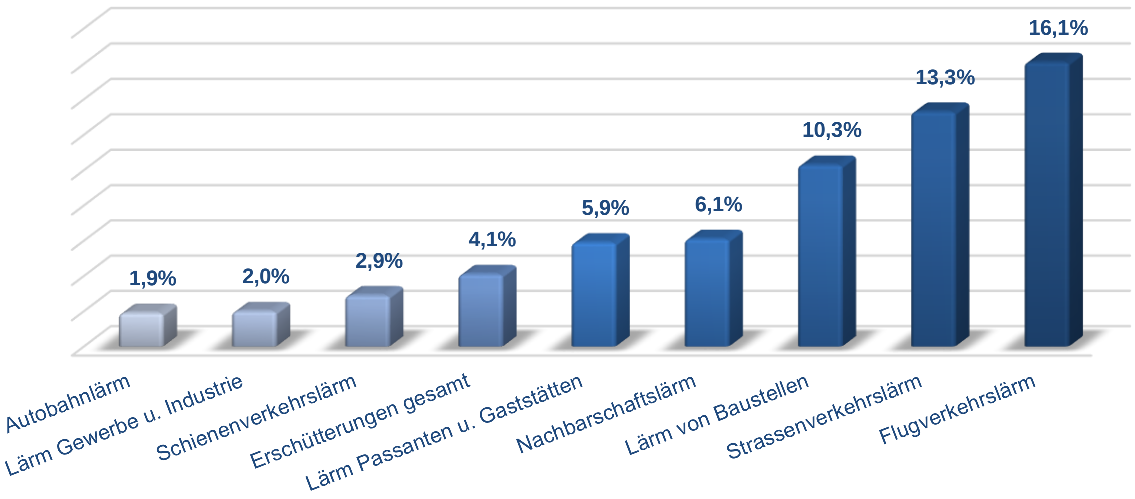

* Nach empfundenem Expositionsausmaß (Häufigkeit) aufsteigend sortiert

1.2. Verkehrslärmexposition und – Belastung

Die empfundene Ausgesetztheit gegenüber Verkehrslärm steht in deutlicher Beziehung zu den tatsächlich gegebenen Lärmpegeln der einzelnen Verkehrsträger (siehe dazu auch die folgenden Unterkapitel), variiert in Zusammenschau mit denselben allerdings je nach Quelle stark.

Weiters beginnt das Expositionsempfinden im sensibleren Nachtzeitraum im Vergleich zu den 24h-Pegeln bereits bei niedereren Lärmbelastungspegeln deutlich stärker zu steigen:

Abbildung 105: Empfundene Lärmexposition und Verkehrslärmbelastung nach Quelle 24h- (L_{den}) und Nachtzeitraum (L_n) gruppiert im Mittelwertvergleich (aufsteigend sortiert)

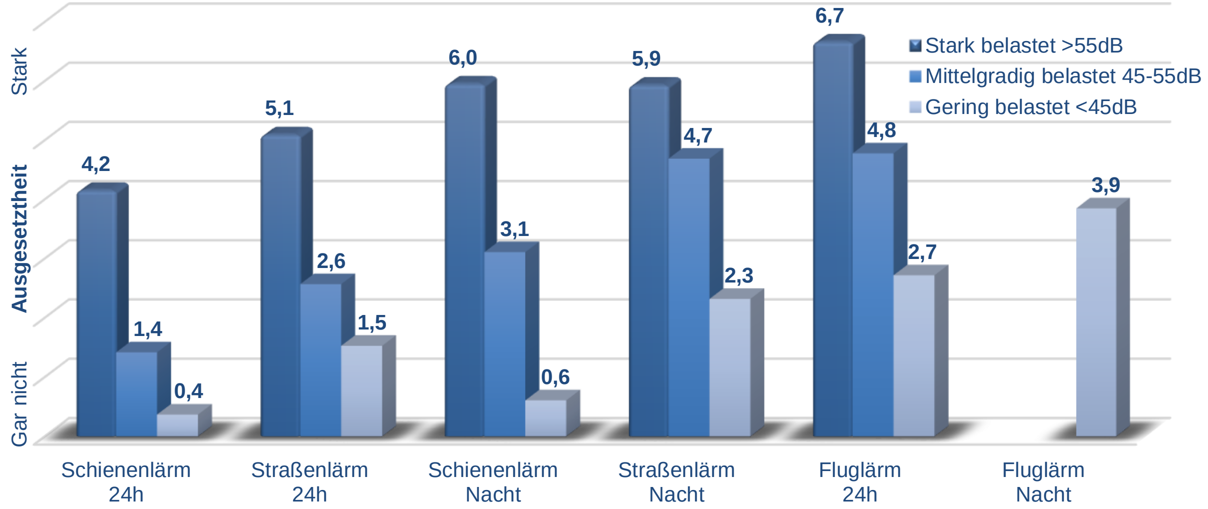

Anm.: In der Nacht gibt es keine vom Fluglärm stark belasteten Befragten und nur 4 mittelgradig Belastete, weshalb diese Belastungskategorien in der obenstehenden Grafik nicht wiedergegeben werden.

Etwas detaillierter ist dies aus dem Vergleich zwischen der empfundenen Lärmexposition und der gegebenen Lärmbelastung durch Straßen- und Flugverkehr jeweils im 24h-Zeitraum (L_{den}) und im Nachtzeitraum (L_n) ablesbar:

Abbildung 106: Empfundene Lärmexposition und Lärmbelastung durch Straßen- und Flugverkehr im 24h- (L_{den}) und Nachtzeitraum (L_n) in 10dB-Schritten im Mittelwertvergleich (absteigend sortiert)

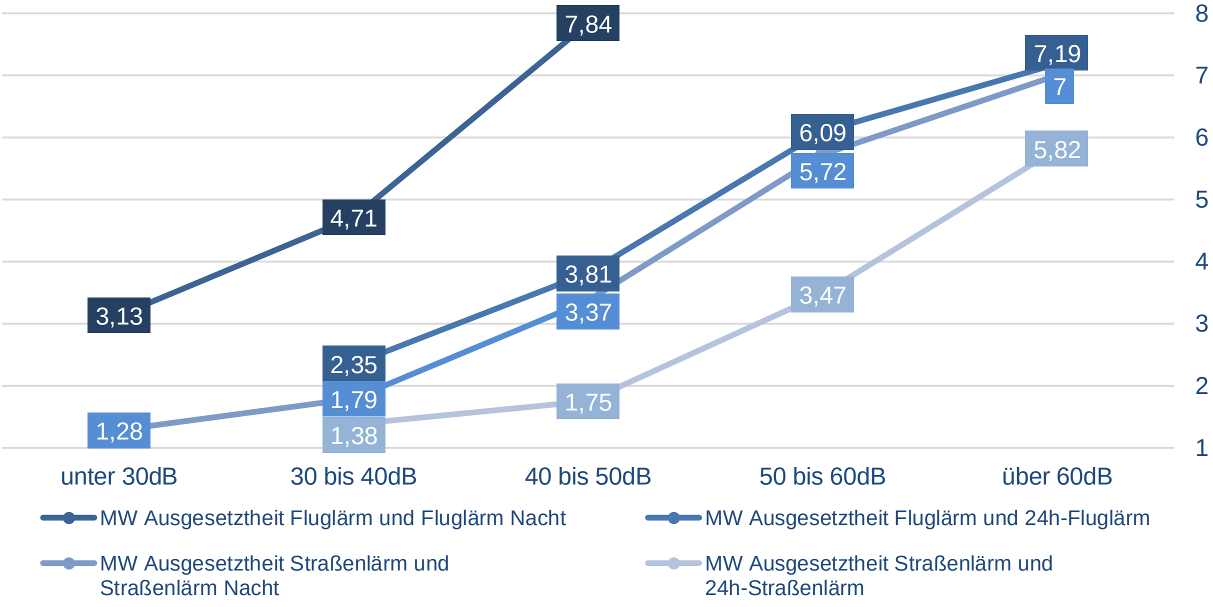

Das gemittelte Empfinden einer verstärkten Ausgesetztheit gegenüber Flugverkehrslärm in der Nacht beginnt bei niedrigen Lärmpegeln, ist in allen Lärmbelastungskategorien deutlich höher als beim Straßenverkehr und übertrifft auch im 24h-Zeitraum das Gefühl der eigenen Lärmexposition gegenüber dem nächtlichen Straßenverkehr.

Das Lärmexpositionsempfinden gegenüber der gegebenen Schienenverkehrslärmbelastung liegt unterhalb dieser Werte und beginnt erst bei vergleichsweise höheren Lärmpegeln stärker anzusteigen:

Abbildung 107: Empfundene Lärmexposition und Schienenverkehrslärmbelastung im 24h- (L_{den}) und Nachtzeitraum (L_n) in 10dB-Schritten im Mittelwertvergleich

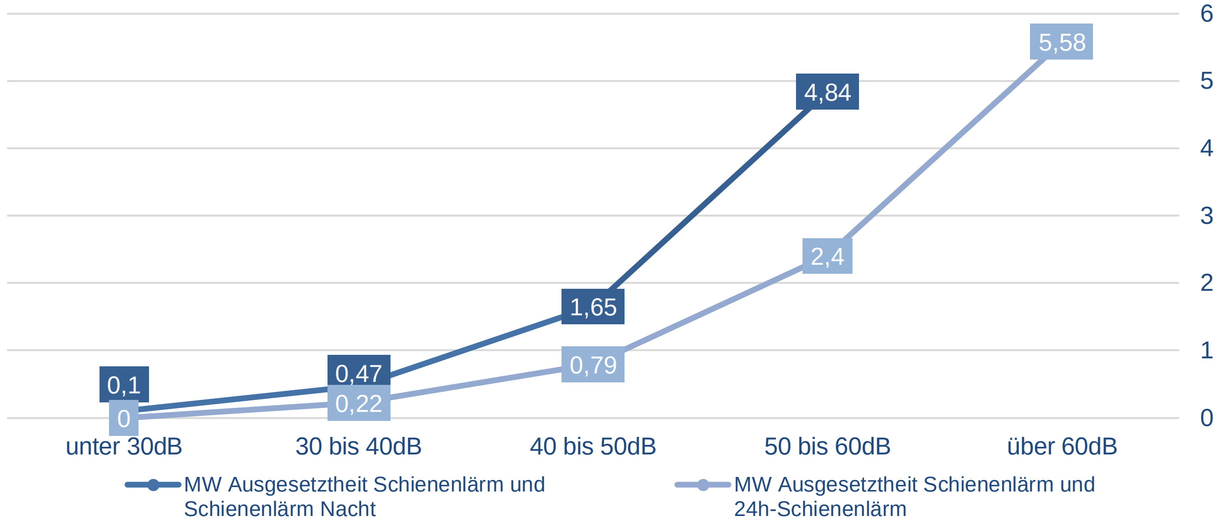

2. Lärmexposition insgesamt

Das Empfinden der Ausgesetztheit des eigenen Hauses / der eigenen Wohnung gegenüber Lärm insgesamt wurde mittels eines eigenen Items zusätzlich abgefragt:

Abbildung 108: Empfundene Lärmexposition insgesamt in Prozent

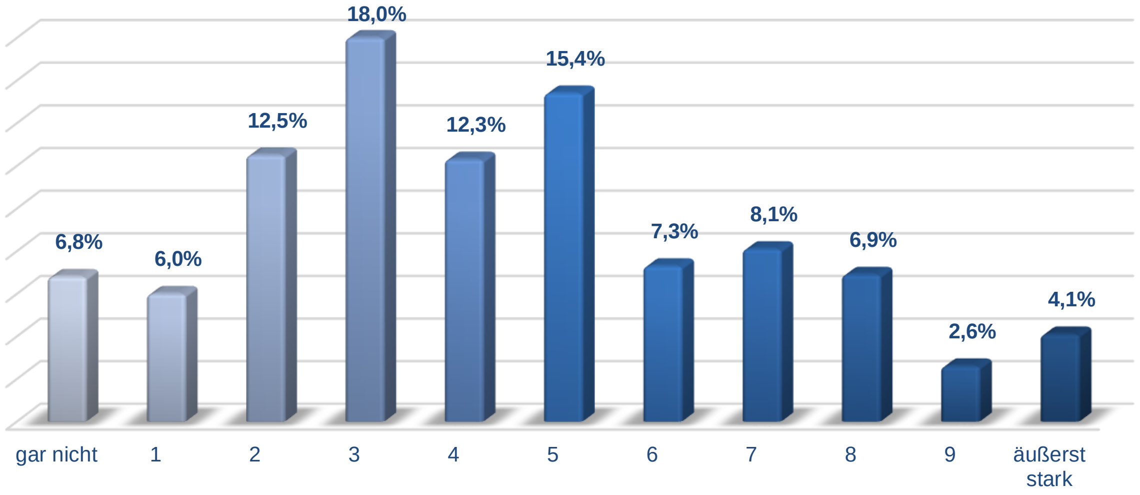

13,6% aller befragten Innsbruckerinnen und Innsbrucker schätzen ihre Wohnung / das Haus in dem sie leben, dem Lärm insgesamt als stark ausgesetzt (*highly exposed*: gruppierte Antwortkategorien 8, 9 und 10 [=*äußerst stark*]) ein, 61% antworten in den mittleren Kategorien und etwas mehr als ein

Viertel aller Probandinnen und Probanden empfindet das eigene Wohnumfeld als gering dem Lärm insgesamt ausgesetzt (gruppierte Antwortkategorien 0 [=*gar nicht*], 1 und 2):

Tabelle 96: Empfundene Lärmexposition insgesamt gruppiert

		Häufigkeit	Gültige Prozente	Kumulierte Prozente
Gültig	Gering exponiert	261	25,3	25,3
	Mittelgradig exponiert	630	61,1	86,4
	Stark exponiert	140	13,6	100,0
	Gesamt	1031	100,0	

Im Mittel liegen die Antworten der befragten Probandinnen und Probanden bei 4,31 (47,4%). Nur knapp 7% aller Befragten sehen sich in ihrem Wohnumfeld Lärm insgesamt überhaupt nicht ausgesetzt.

2.1. Lärmexposition insgesamt und Katastralgemeinden

Im Mittel die größte Lärmexposition insgesamt wird in Wilten empfunden, gefolgt von Innsbruck, Pradl und Hötting. Die geringste Ausgesetztheit gegenüber Lärm insgesamt im eigenen Wohnumfeld empfinden die Befragten in Mühlau, Igls und Vill:

Abbildung 109: Empfundene Lärmexposition insgesamt und Katastralgemeinden im Mittelwertvergleich

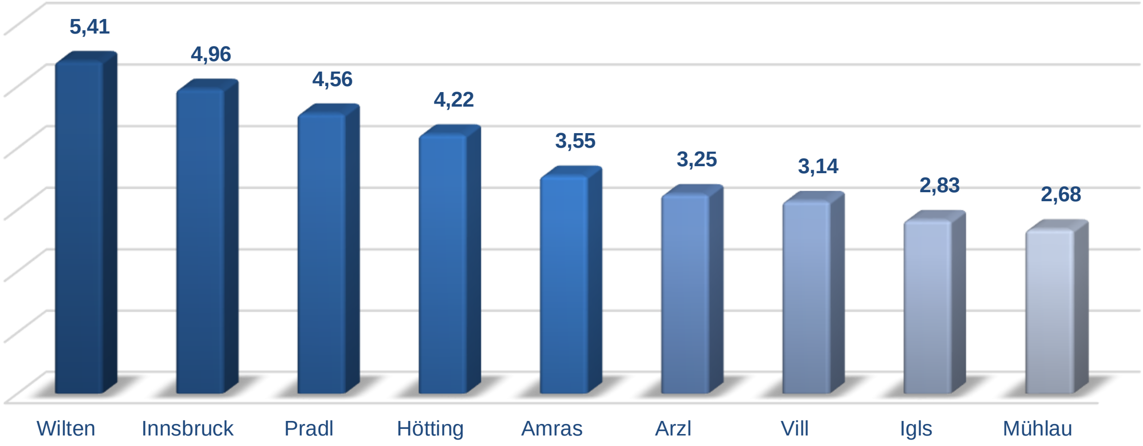

2.2. Lärmexposition insgesamt und ausgewählte Parameter

Die klassischen demographischen Parameter Geschlecht, Alter und Bildung zeigen in Zusammenschau mit der Frage nach der empfundenen Lärmexposition insgesamt nur vergleichsweise geringe Unterschiede in ihren Subgruppen.

Befragte Innsbruckerinnen und Innsbrucker,

- deren Wohnung durch bauliche Abschirmungen (Gebäude, Mauern etc.) vor Verkehrslärm weitestgehend geschützt sind,
- die häufig bei geöffnetem oder gekipptem Fenster schlafen,
- die über keine Lärmschutzfenster verfügen,

empfinden sich dem Lärm insgesamt weniger ausgesetzt.

Auch bei den subjektiven Parametern wird der deutliche Einfluss der Disposition deutlich, die auch nach Adjustierung mit den gegebenen Verkehrslärmpegeln erhalten bleibt: Befragte, welche

- ihre Lebensbedingungen und ihre Lebensqualität besser einschätzen,
- zufriedener mit ihrer Wohnsituation insgesamt sind,
- von keiner Zunahme der Lärmbelastungen in Innsbruck in den vergangenen Jahren ausgehen,
- Innsbruck im Vergleich zu anderen Städten Österreichs für ruhiger halten,
- keine Notwendigkeit für (weitere) Maßnahmen zur Lärmreduktion sehen,
- sich selbst als weniger lärmempfindlich einstufen,

fühlen sich weniger häufig und in geringerem Ausmaß lärmexponiert:

Tabelle 97: Empfundene Lärmexposition insgesamt und ausgewählte Parameter (gruppiert)

		**Ausgesetztheit Lärm gesamt gruppiert**					
		**Gering exponiert**		**Mittelgradig exponiert**		**Stark exponiert**	
		Anzahl	Zeilen (%)	Anzahl	Zeilen (%)	Anzahl	Zeilen (%)
**Geschlecht**	weiblich	129	23,8%	330	61,0%	82	15,2%
	männlich	132	26,9%	300	61,2%	58	11,8%
**Altersgruppen**	18 bis 40 Jahre	99	22,5%	277	63,0%	64	14,5%
	41 bis 60 Jahre	83	26,3%	195	61,7%	38	12,0%
	über 60 Jahre	79	28,7%	158	57,5%	38	13,8%
**Bildungsschicht**	Primärer Schulabschluss	28	28,6%	53	54,1%	17	17,3%
	Sekundärer Schulabschluss	118	21,8%	358	66,1%	66	12,2%
	Tertiärer Schulabschluss	113	29,1%	218	56,2%	57	14,7%
**Baulicher Schutz vor Verkehrslärm**	Keiner bis geringfügig	174	22,7%	473	61,7%	119	15,5%
	Gut bis komplett	77	32,6%	141	59,7%	18	7,6%
**Innenhof bzw. lärmabgewandte Seite**	Ja	174	24,8%	447	63,8%	80	11,4%
	Nein	80	26,0%	172	55,8%	56	18,2%
**Nachtschlaf**	Fenster geöffnet/gekippt	226	27,5%	508	61,9%	87	10,6%
	Fenster geschlossen	31	15,3%	119	58,9%	52	25,7%
**Lärmschutzfenster in den letzten 10 Jahren eingebaut**	Ja	25	13,4%	119	63,6%	43	23,0%
	Nein	197	29,3%	400	59,5%	75	11,2%
	weiß nicht/ k.A.	39	22,7%	111	64,5%	22	12,8%
**Lebensbedingungen (Summenscore)**	Nicht gut	0	0,0%	3 (*)	60,0%	2 (*)	40,0%
	Durchschnittlich	142	21,2%	422	63,0%	106	15,8%
	Sehr gut	119	33,4%	205	57,6%	32	9,0%

**Zufriedenheit Wohnsituation insgesamt**	Nicht zufrieden	5	17,9%	8	28,6%	15	53,6%
	Durchschnittlich	34	13,2%	163	63,2%	61	23,6%
	Sehr zufrieden	222	29,8%	458	61,6%	64	8,6%
**Lebensqualität**	Nicht gut	2 (*)	20,0%	3 (*)	30,0%	5	50,0%
	Durchschnittlich	47	16,2%	186	64,1%	57	19,7%
	Sehr gut	210	29,0%	437	60,3%	78	10,8%
**Lärmempfindlichkeit**	Nicht lärmempfindlich	100	36,4%	150	54,5%	25	9,1%
	Durchschnittlich	115	19,7%	385	65,9%	84	14,4%
	Sehr lärmempfindlich	46	26,7%	95	55,2%	31	18,0%
**Gesundheitszustand**	Gut	197	26,1%	453	60,1%	104	13,8%
	Zufriedenstellend	43	23,1%	123	66,1%	20	10,8%
	Nicht gut	20	22,5%	53	59,6%	16	18,0%
**Zunahme Lärmbelastung**	Ja	56	12,8%	285	65,4%	95	21,8%
	Nein	205	34,5%	345	58,0%	45	7,6%
**Abnahme Lärmbelastung**	Ja	17	29,3%	37	63,8%	4	6,9%
	Nein	244	25,1%	593	60,9%	136	14,0%
**Lautheit Innsbruck im Vergleich**	Ruhiger	85	29,1%	180	61,6%	27	9,2%
	Gleich	139	25,2%	342	62,1%	70	12,7%
	Lauter	30	17,2%	102	58,6%	42	24,1%
**Notwendigkeit (weiterer) Maßnahmen zur Lärmreduktion**	Ja	141	20,4%	431	62,4%	119	17,2%
	Nein	72	37,5%	106	55,2%	14	7,3%
**Belästigung Lärm gesamt**	Gering belästigt	202	53,4%	167	44,2%	9	2,4%
	Mittelgradig belästigt	55	10,4%	408	77,3%	65	12,3%
	Stark belästigt	4 (*)	3,2%	55	44,0%	66	52,8%

(*) Zu geringe Fallzahlen für solide Auswertungen

Beinahe linear zeichnet sich der Zusammenhang zur Belästigung / Störung durch Lärm insgesamt ab, der stark ausgeprägt ist und das Empfinden der Ausgesetztheit auch als Kontrollvariable bestätigt:

Abbildung 110: Empfundene Lärmexposition insgesamt und Belästigung durch Lärm insgesamt im Mittelwertvergleich

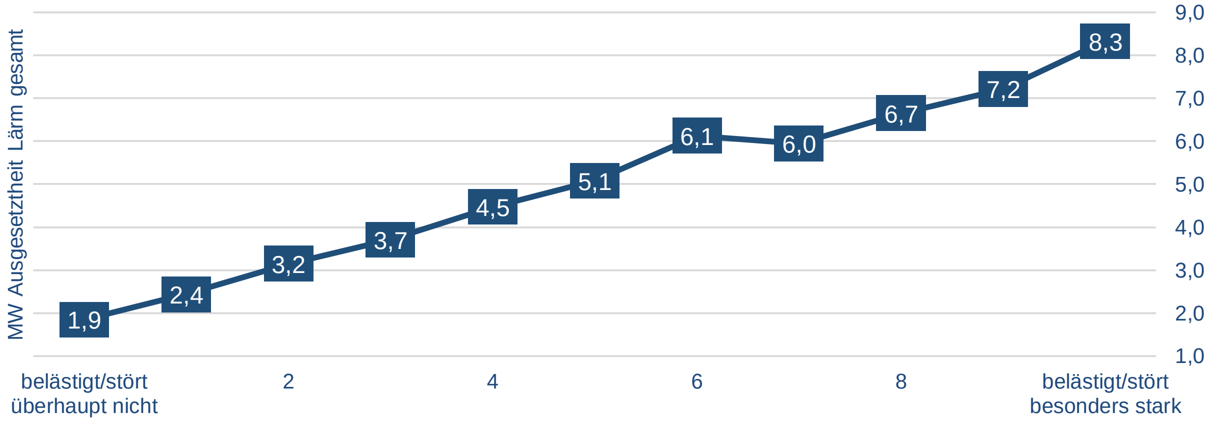

3. Flugverkehrslärmexposition

Knapp 39% aller Befragten sehen sich dem Flugverkehrslärm nicht stark ausgesetzt. Das Empfinden, wie sehr das persönliche Wohnumfeld der Befragten dem Flugverkehrslärm ausgesetzt ist, übertrifft allerdings in der Extremgruppe der sich stark ausgesetzt Empfindenden in seiner Häufigkeit (16%) die Spitzengruppe bei der Frage nach der Lärmexposition insgesamt und liegt auch vor dem Expositionsempfinden durch Straßenverkehrslärm:

Abbildung 111: Empfundene Flugverkehrslärmexposition in Prozent

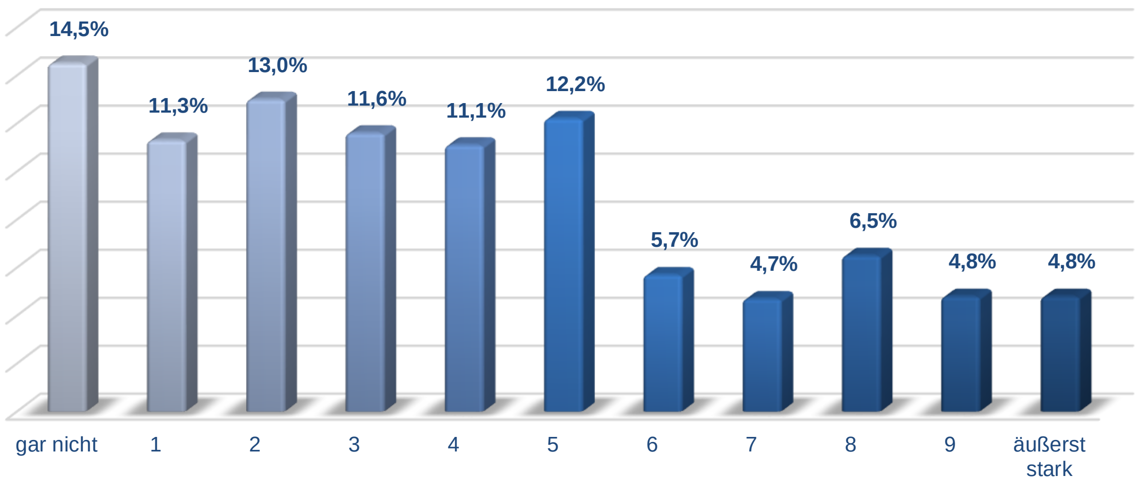

Im Mittel liegen die Antworten der befragten Probandinnen und Probanden bei 3,87 (42,6%) und damit wieder hinter dem Mittelwert der Antworten zur Frage nach der Lärmexposition insgesamt:

Tabelle 98: Empfundene Flugverkehrslärmexposition gruppiert

		Häufigkeit	Gültige Prozente	Kumulierte Prozente
Gültig	Gering exponiert	399	38,7	38,7
	Mittelgradig exponiert	467	45,3	84,0
	Stark exponiert	165	16,0	100,0
	Gesamt	1031	100,0	

3.1. Fluglärmexposition und Fluglärmbelastung

Die empfundene Ausgesetztheit gegenüber Flugverkehrslärm korrespondiert sehr ausgeprägt mit der tatsächlich gegebenen Fluglärmbelastung und zeigt mit steigender gegebener Belastung ein nahezu linear ansteigendes Expositionsempfinden:

Abbildung 112: Empfundene Flugverkehrslärmexposition und 24h-Fluglärmbelastung L_{den} in 10dB-Schritten im Mittelwertvergleich

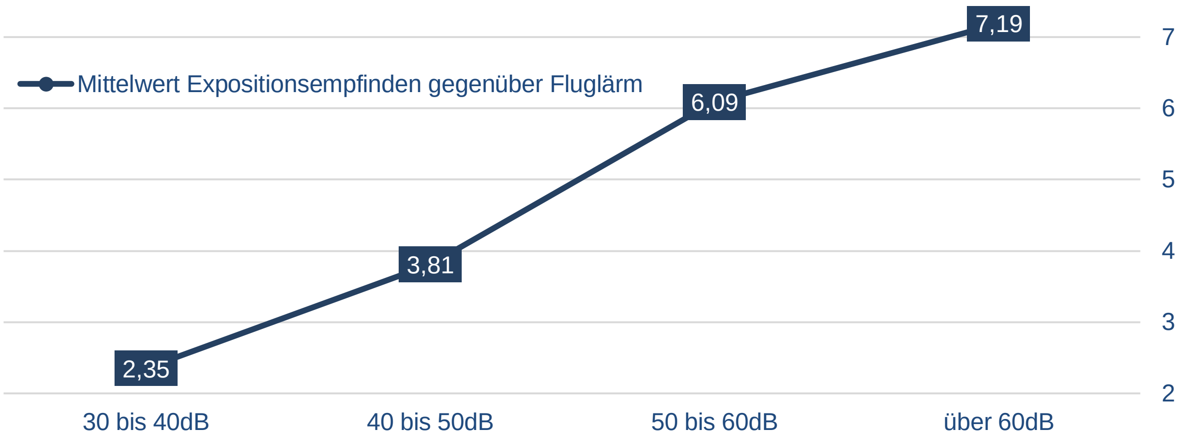

Das gilt auch für den Nachtzeitraum. Das verstärkte Expositionsempfinden beginnt jedoch bereits bei niedrigeren Lärmpegeln und ist deutlich stärker ausgeprägt:

Abbildung 113: Empfundene Flugverkehrslärmexposition und nächtliche Fluglärmbelastung L_n in 10dB-Schritten im Mittelwertvergleich

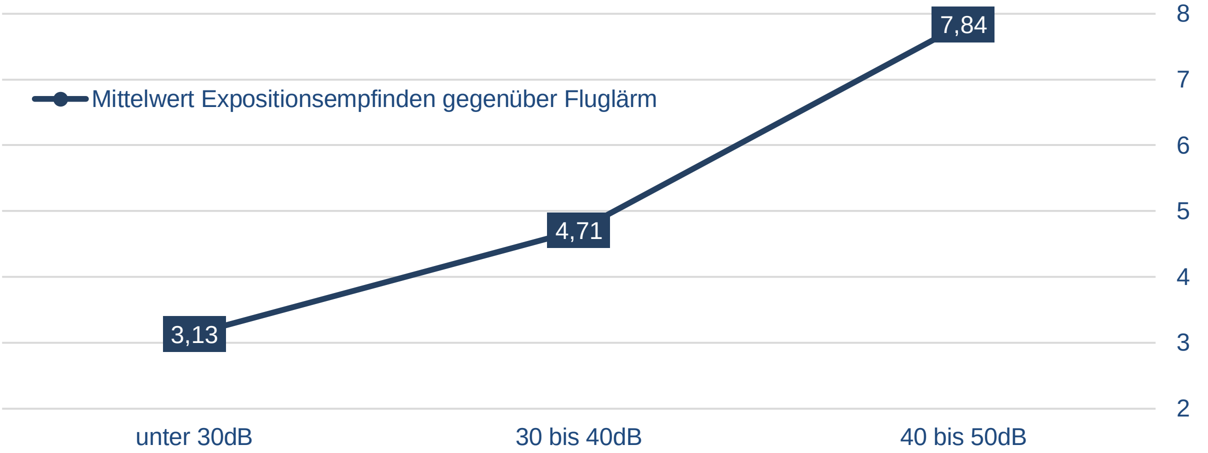

3.2. Fluglärmexposition und Katastralgemeinden

Abbildung 114: Empfundene Fluglärmexposition und Katastralgemeinden im Mittelwertvergleich

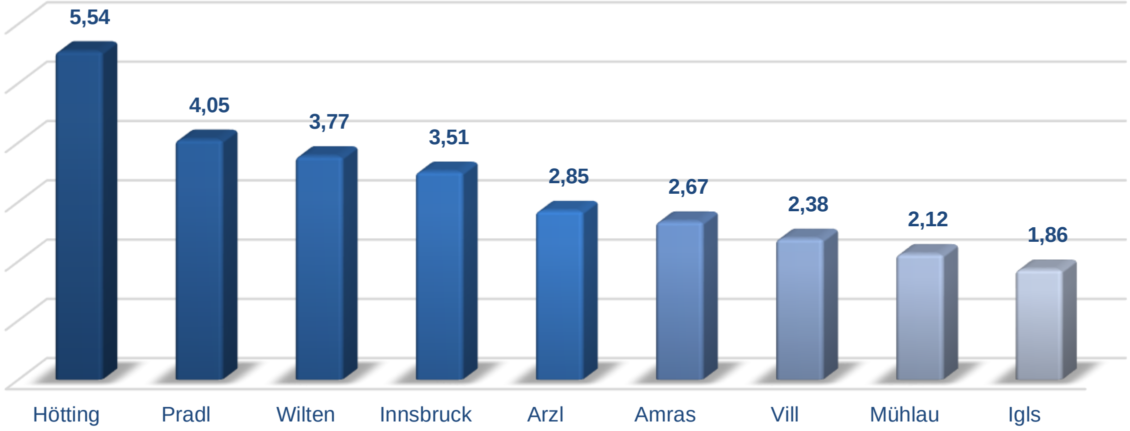

Entsprechend der gegebenen Fluglärmbelastung zeigt sich das mit Abstand stärkste Fluglärmexpositionsempfinden in Hötting. Die geringste Ausgesetztheit gegenüber Fluglärm im eigenen Wohnumfeld empfinden die Befragten in Igls.

3.3. Fluglärmexposition und ausgewählte Parameter

In der Kombination mit einzelnen Variablen zeigen sich sehr ähnliche Auswertungsergebnisse wie schon in der Zusammenschau mit der empfundenen Ausgesetztheit gegenüber Lärm insgesamt:

Tabelle 99: Empfundene Fluglärmexposition und ausgewählte Parameter (gruppiert)

		Ausgesetztheit Flugverkehrslärm gruppiert					
		Gering exponiert		Mittelgradig exponiert		Stark exponiert	
		Anzahl	Zeilen (%)	Anzahl	Zeilen (%)	Anzahl	Zeilen (%)
**Geschlecht**	weiblich	200	37,0%	253	46,8%	88	16,3%
	männlich	199	40,6%	214	43,7%	77	15,7%
**Altersgruppen**	18 bis 40 Jahre	178	40,5%	188	42,7%	74	16,8%
	41 bis 60 Jahre	117	37,0%	152	48,1%	47	14,9%
	über 60 Jahre	104	37,8%	127	46,2%	44	16,0%
**Bildungsschicht**	Primärer Schulabschluss	38	38,8%	48	49,0%	12	12,2%
	Sekundärer Schulabschluss	215	39,7%	248	45,8%	79	14,6%
	Tertiärer Schulabschluss	145	37,4%	169	43,6%	74	19,1%
**Baulicher Schutz vor Verkehrslärm**	Keiner bis geringfügig	295	38,5%	339	44,3%	132	17,2%
	Gut bis komplett	86	36,4%	119	50,4%	31	13,1%
**Innenhof bzw. lärm-abgewandte Seite**	Ja	285	40,7%	324	46,2%	92	13,1%
	Nein	106	34,4%	133	43,2%	69	22,4%
**Nachtschlaf**	Fenster geöffnet/gekippt	319	38,9%	372	45,3%	130	15,8%
	Fenster geschlossen	76	37,6%	93	46,0%	33	16,3%
**Lärmschutzfenster in den letzten 10 Jahren eingebaut**	Ja	56	29,9%	88	47,1%	43	23,0%
	Nein	260	38,7%	312	46,4%	100	14,9%
	weiß nicht/ k.A.	83	48,3%	67	39,0%	22	12,8%
**Lebensbedingungen (Summenscore)**	Nicht gut (*)	1	20,0%	2	40,0%	2	40,0%
	Durchschnittlich	229	34,2%	317	47,3%	124	18,5%
	Sehr gut	169	47,5%	148	41,6%	39	11,0%
**Zufriedenheit Wohnsituation insgesamt**	Nicht zufrieden	10	35,7%	10	35,7%	8	28,6%
	Durchschnittlich	88	34,1%	118	45,7%	52	20,2%
	Sehr zufrieden	301	40,5%	338	45,4%	105	14,1%
**Lebensqualität**	Nicht gut (*)	2	20,0%	4	40,0%	4	40,0%
	Durchschnittlich	103	35,5%	135	46,6%	52	17,9%
	Sehr gut	292	40,3%	325	44,8%	108	14,9%
**Lärmempfindlichkeit**	Nicht lärmempfindlich	134	48,7%	105	38,2%	36	13,1%
	Durchschnittlich	217	37,2%	278	47,6%	89	15,2%

	Sehr lärmempfindlich	48	27,9%	84	48,8%	40	23,3%
Gesundheitszustand	Gut	298	39,5%	342	45,4%	114	15,1%
	Zufriedenstellend	75	40,3%	79	42,5%	32	17,2%
	Nicht gut	25	28,1%	45	50,6%	19	21,3%
Zunahme Lärmbelastung	Ja	133	30,5%	206	47,2%	97	22,2%
	Nein	266	44,7%	261	43,9%	68	11,4%
Abnahme Lärmbelastung	Ja	32	55,2%	21	36,2%	5	8,6%
	Nein	367	37,7%	446	45,8%	160	16,4%
Lautheit Innsbruck im Vergleich	Ruhiger	123	42,1%	120	41,1%	49	16,8%
	Gleich	220	39,9%	248	45,0%	83	15,1%
	Lauter	50	28,7%	91	52,3%	33	19,0%
Notwendigkeit (weiterer) Maßnahmen zur Lärmreduktion	Ja	232	33,6%	323	46,7%	136	19,7%
	Nein	88	45,8%	86	44,8%	18	9,4%
Belästigung Fluglärm	Gering belästigt	365	71,6%	135	26,5%	10	2,0%
	Mittelgradig belästigt	33	8,3%	313	78,3%	54	13,5%
	Stark belästigt	1 (*)	0,8%	19	15,7%	101	83,5%

(*) Zu geringe Fallzahlen für solide Auswertungen

4. Straßenverkehrslärmexposition

Das Ausmaß des Expositionsempfindens gegenüber Straßenverkehrslärm entspricht in seinen Dimensionen größtenteils dem Empfinden der Ausgesetztheit des eigenen Wohnumfeldes gegenüber Flugverkehrslärm:

Abbildung 115: Empfundene Straßenverkehrslärmexposition in Prozent

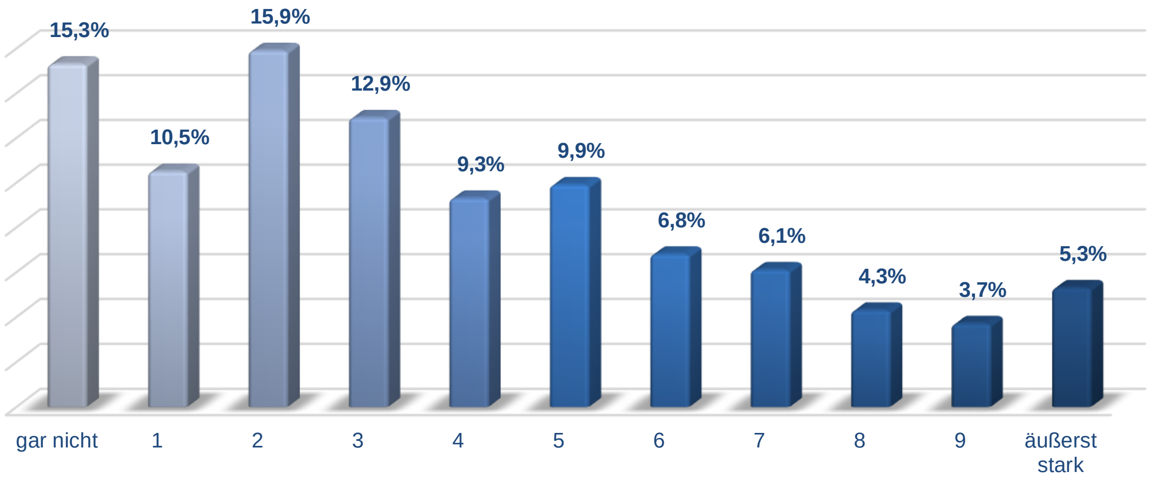

Im Mittel liegen die Antworten der Befragten bei 3,72 (40,9%).

Tabelle 100: Empfundene Straßenverkehrslärmexposition gruppiert

		Häufigkeit	Gültige Prozente	Kumulierte Prozente
Gültig	Gering exponiert	430	41,7	41,7
	Mittelgradig exponiert	464	45,0	86,7
	Stark exponiert	137	13,3	100,0
	Gesamt	1031	100,0	

4.1. Straßenlärmexposition und Straßenlärmbelastung

Auch in der Zusammenschau der empfundenen Straßenverkehrslärmexposition mit der gegebenen Straßenverkehrslärmbelastung zeigt sich ein deutlicher Bezug. Im Unterschied zum Expositionsempfinden beim Fluglärm steigt das Empfinden einen starken Exposition erst bei höheren gegebenen Lärmpegeln an und ist auch in der Kategorie der mit über 60dB-Dauerschallpegel stark belasteten Befragten geringer ausgeprägt als beim Empfinden der sich stark dem Flugverkehrslärm ausgesetzt und auch tatsächlich stark belasteten Befragten:

Abbildung 116: Empfundene Straßenverkehrslärmexposition und 24h-Straßenverkehrslärmbelastung L_{den} in 10dB-Schritten im Mittelwertvergleich

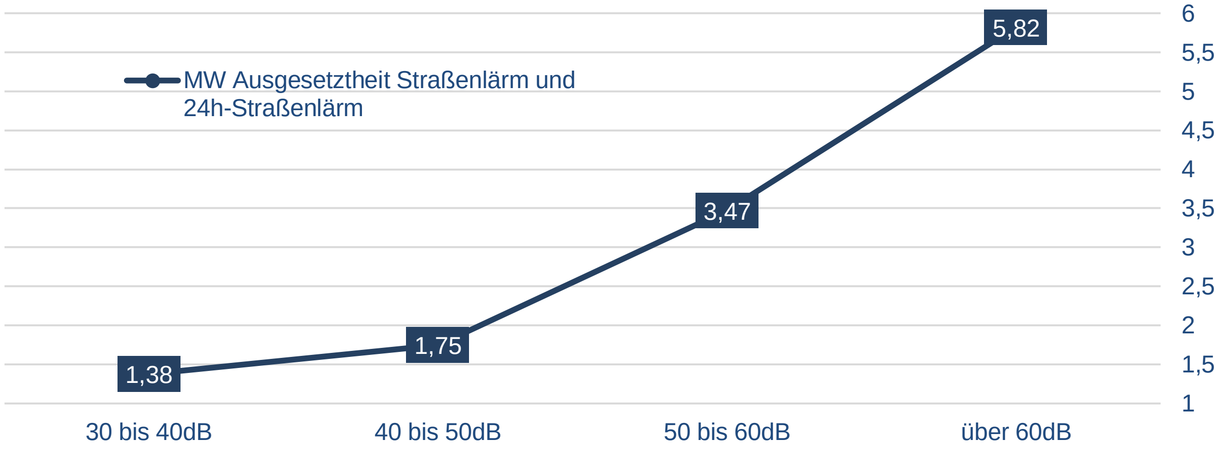

Wie schon beim Flugverkehrslärm zeigt sich auch beim Empfinden der eigenen Ausgesetztheit gegenüber Straßenverkehrslärm eine ausgeprägt stärkere Sensitivität im Nachtzeitraum. Das Gefühl der der eigenen Ausgesetztheit beginnt bereits bei niedrigeren Lärmpegeln, steigt stärker an und ist deutlich stärker ausgeprägt als im 24h-Zeitraum:

Abbildung 117: Empfundene Straßenverkehrslärmexposition und nächtliche Straßenverkehrslärmbelastung L_n in 10dB-Schritten im Mittelwertvergleich

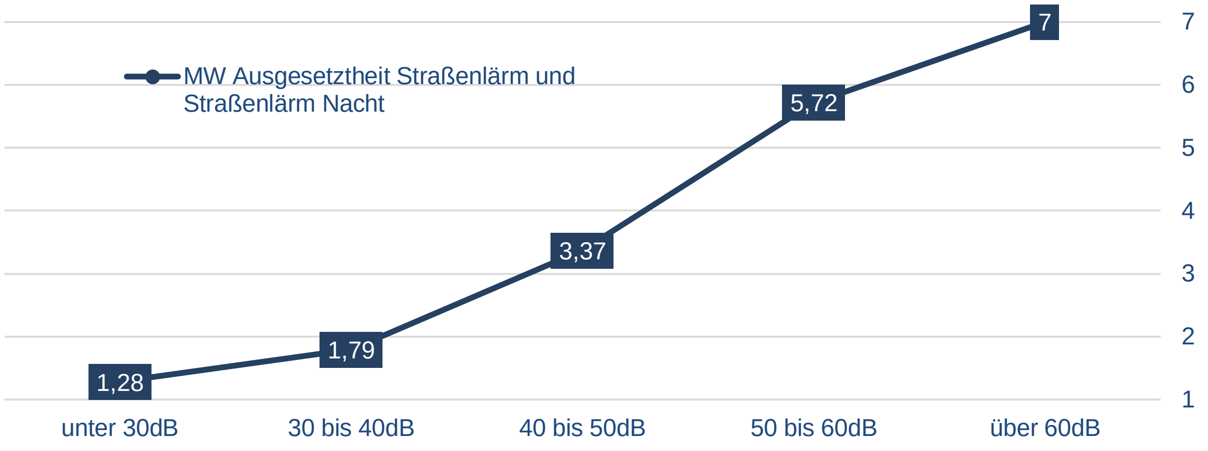

4.2. Straßenlärmexposition und Katastralgemeinden

Das größte Ausmaß an empfundener Straßenlärmexposition wird mit deutlichem Abstand in Wilten empfunden, gefolgt von Pradl, Innsbruck und AmraS. Die geringste Ausgesetztheit gegenüber Straßenverkehrslärm im eigenen Wohnumfeld empfinden die Befragten in Igls, Mühlau und Vill:

Abbildung 118: Empfundene Straßenlärmexposition und Katastralgemeinden im Mittelwertvergleich

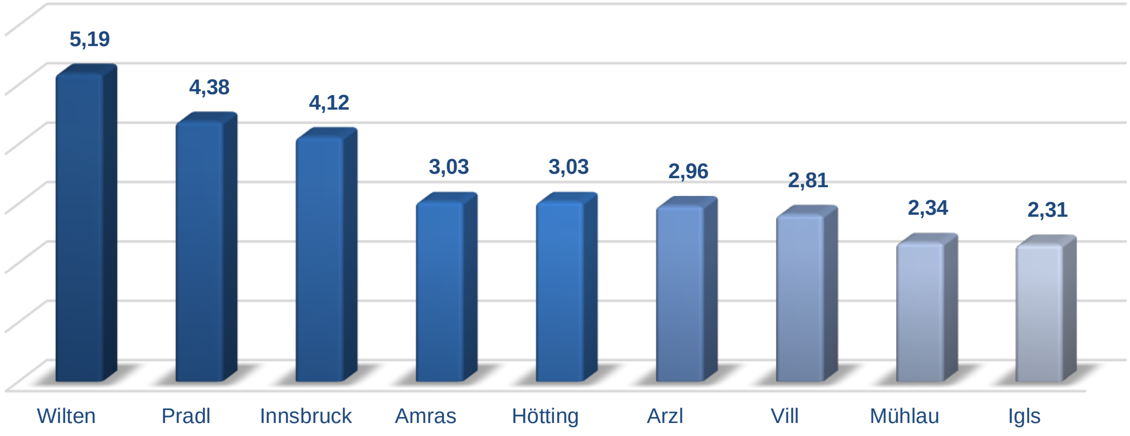

4.3. Straßenlärmexposition und ausgewählte Parameter

Frauen empfinden sich stärker dem Straßenverkehrslärm ausgesetzt als Männer, während das Alter und der höchste bisher erreiche Schulabschluss nur eine untergeordnete Rolle einnehmen.

Wiederum zeigen sich starke Beziehungen zu den verschiedenen Einschätzungsfragen der eigenen Wohn- und Lebensumstände: Je besser diese bewertet werden, desto weniger fühlen sich die befragten Innsbruckerinnen und Innsbrucker dem Straßenverkehrslärm ausgesetzt und vice versa.

Die stärkste Beziehung besteht erneut zum Ausmaß der Belästigung durch den Straßenverkehrslärm. Wie schon in Abhängigkeit von der Belästigung durch Flugverkehrslärm steigt das Ausmaß der empfundenen Ausgesetztheit auch in Bezug zur Straßenverkehrslärmbelästigung nahezu linear an:

Abbildung 119: Empfundene Straßenverkehrslärmexposition und Belästigung durch Straßenverkehrslärm im Mittelwertvergleich

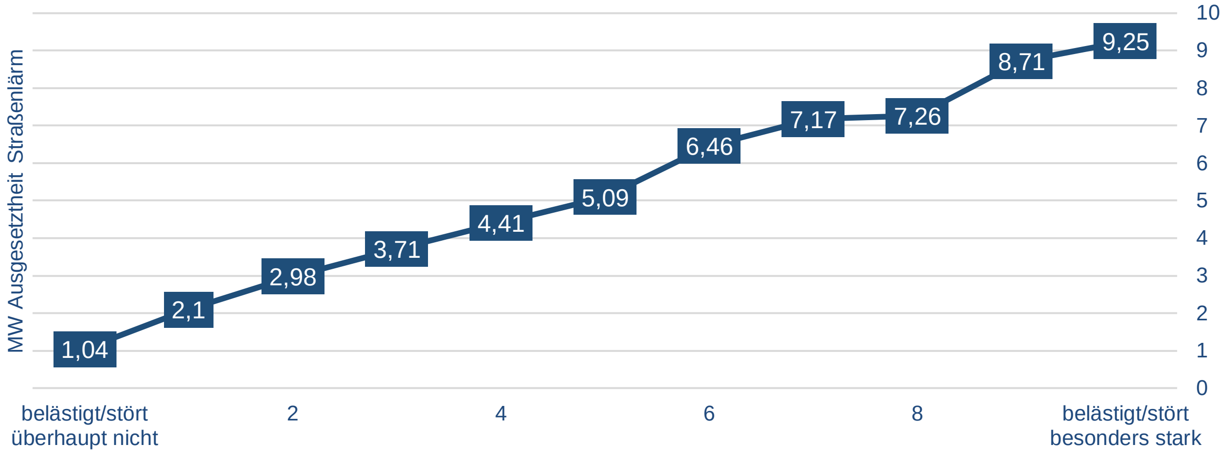

Tabelle 101: Empfundene Straßenlärmexposition und ausgewählte Parameter (gruppiert)

		Ausgesetztheit Straßenverkehrslärm gruppiert					
		Gering exponiert		Mittelgradig exponiert		Stark exponiert	
		Anzahl	Zeilen (%)	Anzahl	Zeilen (%)	Anzahl	Zeilen (%)
**Geschlecht**	weiblich	213	39,4%	246	45,5%	82	15,2%
	männlich	217	44,3%	218	44,5%	55	11,2%
**Baulicher Schutz vor Verkehrslärm**	Keiner bis geringfügig	287	37,5%	357	46,6%	122	15,9%
	Gut bis komplett	132	55,9%	93	39,4%	11	4,7%
**Innenhof bzw. lärm-abgewandte Seite**	Ja	293	41,8%	330	47,1%	78	11,1%
	Nein	128	41,6%	124	40,3%	56	18,2%
**Nachtschlaf**	Fenster geöffnet/gekippt	372	45,3%	365	44,5%	84	10,2%
	Fenster geschlossen	53	26,2%	96	47,5%	53	26,2%
**Lärmschutzfenster in den letzten 10 Jahren eingebaut**	Ja	59	31,6%	80	42,8%	48	25,7%
	Nein	299	44,5%	303	45,1%	70	10,4%
	weiß nicht/ k.A.	72	41,9%	81	47,1%	19	11,0%
**Lebensbedingungen (Summenscore)**	Nicht gut (*)	2	40,0%	2	40,0%	1	20,0%
	Durchschnittlich	243	36,3%	319	47,6%	108	16,1%
	Sehr gut	185	52,0%	143	40,2%	28	7,9%
**Zufriedenheit Wohnsituation insgesamt**	Nicht zufrieden	12	42,9%	8	28,6%	8	28,6%
	Durchschnittlich	75	29,1%	118	45,7%	65	25,2%
	Sehr zufrieden	343	46,1%	338	45,4%	63	8,5%
**Lebensqualität**	Nicht gut	5	50,0%	2 (*)	20,0%	3 (*)	30,0%
	Durchschnittlich	110	37,9%	124	42,8%	56	19,3%
	Sehr gut	310	42,8%	338	46,6%	77	10,6%
**Lärmempfindlichkeit**	Nicht lärmempfindlich	142	51,6%	111	40,4%	22	8,0%

	Durchschnittlich	216	37,0%	277	47,4%	91	15,6%
	Sehr lärmempfindlich	72	41,9%	76	44,2%	24	14,0%
**Gesundheitszustand**	Gut	323	42,8%	338	44,8%	93	12,3%
	Zufriedenstellend	75	40,3%	85	45,7%	26	14,0%
	Nicht gut	30	33,7%	41	46,1%	18	20,2%
**Zunahme Lärmbelastung**	Ja	120	27,5%	229	52,5%	87	20,0%
	Nein	310	52,1%	235	39,5%	50	8,4%
**Abnahme Lärmbelastung**	Ja	27	46,6%	27	46,6%	4	6,9%
	Nein	403	41,4%	437	44,9%	133	13,7%
**Lautheit Innsbruck im Vergleich**	Ruhiger	133	45,5%	132	45,2%	27	9,2%
	Gleich	229	41,6%	251	45,6%	71	12,9%
	Lauter	59	33,9%	77	44,3%	38	21,8%
**Notwendigkeit (weitere) Maßnahmen zur Lärmreduktion**	Ja	253	36,6%	329	47,6%	109	15,8%
	Nein	109	56,8%	70	36,5%	13	6,8%
**Belästigung Straßenlärm**	Gering belästigt	385	71,4%	149	27,6%	5	0,9%
	Mittelgradig belästigt	42	11,2%	288	76,8%	45	12,0%
	Stark belästigt	3 (*)	2,6%	27	23,1%	87	74,4%

(*) Zu geringe Fallzahlen für solide Auswertungen

5. Baustellenlärmexposition

Das Empfinden der Exposition durch den Lärm von Baustellen steht in seinem Ausmaß an dritter Stelle hinter dem Flug- und dem Straßenverkehrslärm, unterscheidet sich aber in seinen Ausprägungen sehr deutlich von diesen beiden:

Abbildung 120: Empfundene Baustellenlärmexposition in Prozent

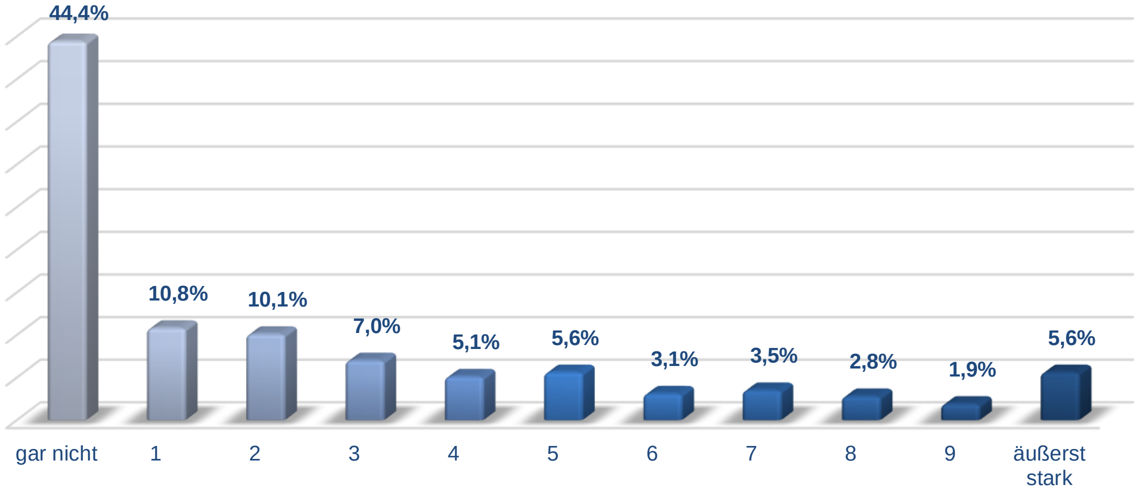

Mit über 44% ist der Anteil an Befragten, die sich überhaupt nicht dem Baustellenlärm exponiert fühlen mehr als dreimal höher als beim Flug- und Straßenverkehrslärm. Knapp zwei Drittel aller Probandin-

nen und Probanden empfinden sich in ihrem Wohnumfeld als gering dem Lärm von Baustellen ausgesetzt (gruppierte Antwortkategorien 0 [=*gar nicht*], 1 und 2), etwas mehr als 10% als stark ausgesetzt (*highly exposed*: gruppierte Antwortkategorien 8, 9 und 10 [=*äußerst stark*]).

Im Mittel liegen die Antworten der befragten Probandinnen und Probanden bei 2,4 (26,4%) und damit deutlich mehr als eine Antwortkategorie unter dem Niveau der Fragen nach dem Flugverkehrs- und dem Straßenverkehrslärm.

Tabelle 102: Empfundene Baustellenlärmexposition gruppiert

		Häufigkeit	Gültige Prozente	Kumulierte Prozente
Gültig	Gering exponiert	673	65,3	65,3
	Mittelgradig exponiert	251	24,3	89,6
	Stark exponiert	107	10,4	100,0
	Gesamt	1031	100,0	

5.1. Baustellenlärmexposition und Katastralgemeinden

Die Befragten in Pradl geben gemittelt das größte Ausmaß an empfundener Baulärmexposition an, die befragten Innsbruckerinnen und Innsbrucker in Vill das mit Abstand niedrigste:

Abbildung 121: Empfundene Baustellenlärmexposition und Katastralgemeinden im Mittelwertvergleich

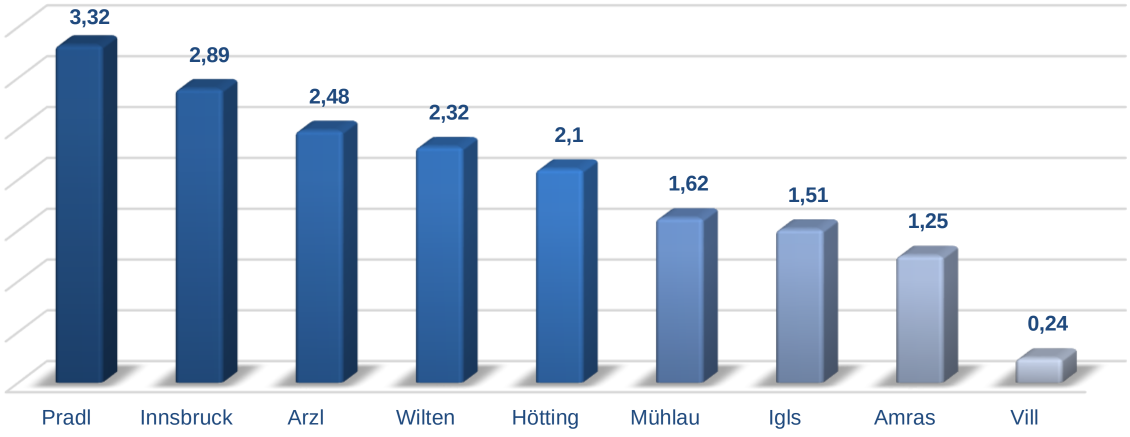

Anmerkung: Baustellenlärm ist stark auf Akutbelastungen bezogen und daher ist ein Vergleich der Katastralgemeinden zu einem sehr erheblichen Anteil nur eine Momentaufnahme.

5.2. Baustellenlärmexposition und ausgewählte Parameter

Männer empfinden sich stärker baulärmexponiert als Frauen.

Mit zunehmendem Alter verringert sich das Ausmaß der empfundenen Ausgesetztheit bei den Befragten.

Die Beziehungen zu anderen ausgewählten Parametern unterscheiden sich nur geringfügig von den Auswertungen zu den Ausgesetztheitsempfindungen gegenüber Verkehrslärm:

Tabelle 103: Empfundene Baustellenlärmexposition und ausgewählte Parameter (gruppiert)

		Ausgesetztheit Lärm von Baustellen gruppiert					
		Gering exponiert		Mittelgradig exponiert		Stark exponiert	
		Anzahl	Zeilen (%)	Anzahl	Zeilen (%)	Anzahl	Zeilen (%)
Geschlecht	weiblich	331	61,2%	144	26,6%	66	12,2%
	männlich	342	69,8%	107	21,8%	41	8,4%
Altersgruppen	18 bis 40 Jahre	276	62,7%	114	25,9%	50	11,4%
	41 bis 60 Jahre	207	65,5%	77	24,4%	32	10,1%
	über 60 Jahre	190	69,1%	60	21,8%	25	9,1%
Bildungsschicht	Primärer Schulabschluss	67	68,4%	24	24,5%	7	7,1%
	Sekundärer Schulabschluss	349	64,4%	131	24,2%	62	11,4%
	Tertiärer Schulabschluss	255	65,7%	95	24,5%	38	9,8%
Baulicher Schutz vor Verkehrslärm	Keiner bis geringfügig	488	63,7%	189	24,7%	89	11,6%
	Gut bis komplett	163	69,1%	59	25,0%	14	5,9%
Innenhof bzw. lärmabgewandte Seite	Ja	453	64,6%	187	26,7%	61	8,7%
	Nein	204	66,2%	60	19,5%	44	14,3%
Nachtschlaf	Fenster geöffnet/gekippt	547	66,6%	192	23,4%	82	10,0%
	Fenster geschlossen	123	60,9%	56	27,7%	23	11,4%
Lärmschutzfenster in den letzten 10 Jahren eingebaut	Ja	106	56,7%	54	28,9%	27	14,4%
	Nein	456	67,9%	154	22,9%	62	9,2%
	weiß nicht/ k.A.	111	64,5%	43	25,0%	18	10,5%
Lebensbedingungen (Summenscore)	Nicht gut (*)	3	60,0%	1	20,0%	1	20,0%
	Durchschnittlich	419	62,5%	173	25,8%	78	11,6%
	Sehr gut	251	70,5%	77	21,6%	28	7,9%
Zufriedenheit Wohnsituation insgesamt	Nicht zufrieden	17	60,7%	6	21,4%	5	17,9%
	Durchschnittlich	139	53,9%	70	27,1%	49	19,0%
	Sehr zufrieden	516	69,4%	175	23,5%	53	7,1%
Lebensqualität	Nicht gut (*)	4	40,0%	2	20,0%	4	40,0%
	Durchschnittlich	170	58,6%	83	28,6%	37	12,8%
	Sehr gut	495	68,3%	165	22,8%	65	9,0%
Lärmempfindlichkeit	Nicht lärmempfindlich	191	69,5%	61	22,2%	23	8,4%
	Durchschnittlich	383	65,6%	138	23,6%	63	10,8%
	Sehr lärmempfindlich	99	57,6%	52	30,2%	21	12,2%
Gesundheitszustand	Gut	501	66,4%	171	22,7%	82	10,9%
	Zufriedenstellend	122	65,6%	52	28,0%	12	6,5%
	Nicht gut	48	53,9%	28	31,5%	13	14,6%
Zunahme Lärmbelastung	Ja	225	51,6%	132	30,3%	79	18,1%
	Nein	448	75,3%	119	20,0%	28	4,7%
Abnahme Lärmbelastung	Ja	42	72,4%	13	22,4%	3 (*)	5,2%
	Nein	631	64,9%	238	24,5%	104	10,7%

**Lautheit Innsbruck im Vergleich**	Ruhiger	197	67,5%	65	22,3%	30	10,3%
	Gleich	362	65,7%	140	25,4%	49	8,9%
	Lauter	105	60,3%	45	25,9%	24	13,8%
**Notwendigkeit (weiterer) Maßnahmen zur Lärmreduktion**	Ja	426	61,6%	180	26,0%	85	12,3%
	Nein	134	69,8%	42	21,9%	16	8,3%
**Belästigung Baulärm**	Gering belästigt	613	91,5%	52	7,8%	5	0,7%
	Mittelgradig belästigt	56	21,2%	179	67,8%	29	11,0%
	Stark belästigt	4 (*)	4,2%	20	20,8%	72	75,0%

(*) Zu geringe Fallzahlen für solide Auswertungen

6. Nachbarschaftslärmexposition

Sehr ähnlich der empfundenen Baustellenlärmexposition stellt sich das Expositionsempfinden beim Nachbarschaftslärm dar. Der Anteil an Personen, die sich in ihrem Wohnumfeld als stark exponiert ansehen, schrumpft auf knapp über 6% und fast 70% aller Befragten schätzen ihr Wohnumfeld als gering exponiert ein (gruppierte Antwortkategorien 0 [=*gar nicht*], 1 und 2):

Abbildung 122: Empfundene Nachbarschaftslärmexposition in Prozent

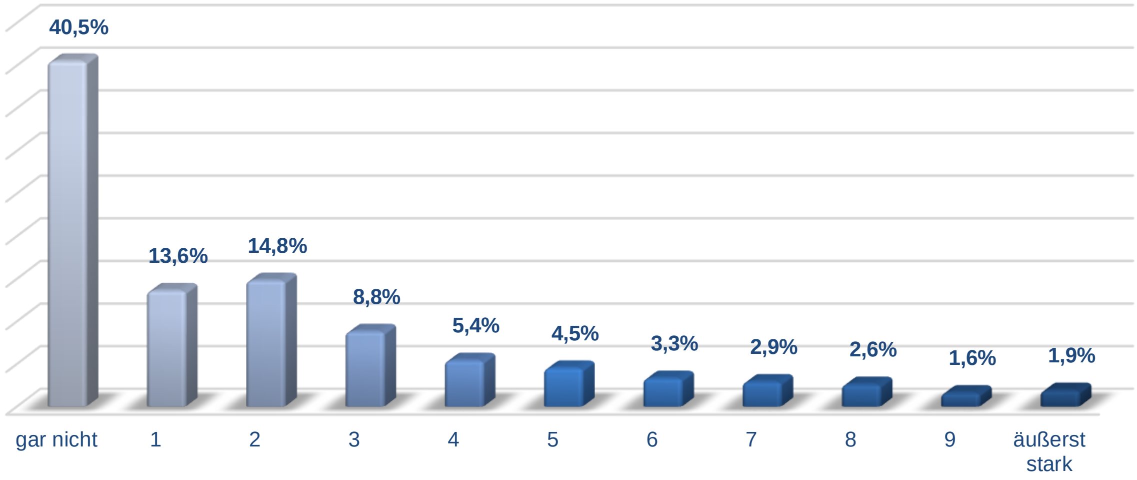

Tabelle 104: Empfundene Nachbarschaftslärmexposition gruppiert

		Häufigkeit	Gültige Prozente	Kumulierte Prozente
Gültig	Gering exponiert	711	69,0	69,0
	Mittelgradig exponiert	257	24,9	93,9
	Stark exponiert	63	6,1	100,0
	Gesamt	1031	100,0	

Im Mittel liegen die Antworten der befragten Probandinnen und Probanden bei 2,08 (22,9%).

6.1. Nachbarschaftslärmexposition und Katastralgemeinden

In Wilten, Innsbruck und Pradl fühlen sich die Befragten am stärksten dem Nachbarschaftslärm ausgesetzt, in Igls, Vill und Mühlau am geringsten:

Abbildung 123: Empfundene Nachbarschaftslärmexposition und Katastralgemeinden im Mittelwertvergleich

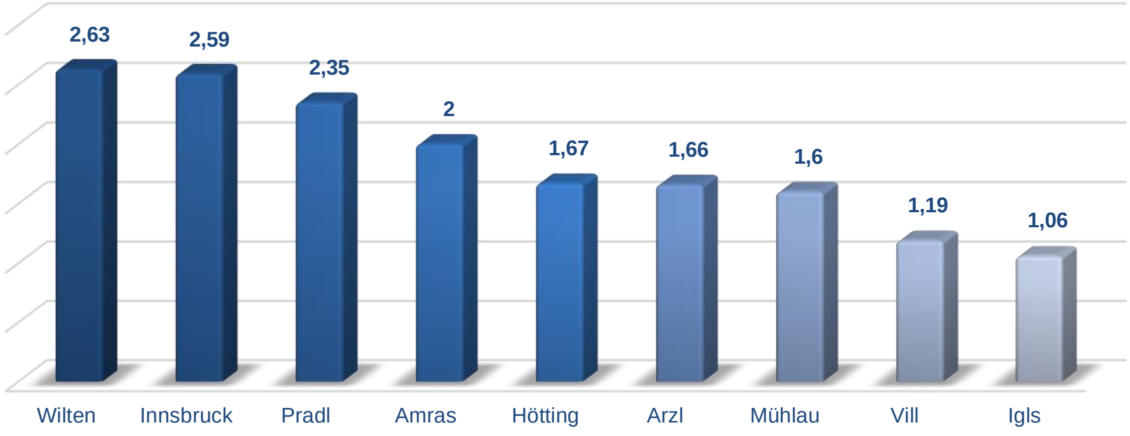

6.2. Nachbarschaftslärmexposition und ausgewählte Parameter

Die Gruppe der über 60-Jährigen empfindet sich weniger dem Nachbarschaftslärm ausgesetzt als die beiden jüngeren Altersgruppen. Ein ähnlich gelagerter Effekt bei den Pflichtschulabsolventinnen und –Absolventen verliert sich nach Altersadjustierung.

Die sich ergebenden Unterschiede zwischen einzelnen Gruppen in Bezug zu anderen ausgewählten Variablen sind vergleichsweise stark ausgeprägt und streuen stark:

Tabelle 105: Empfundene Nachbarschaftslärmexposition und ausgewählte Parameter (gruppiert)

		Ausgesetztheit Nachbarschaftslärm gruppiert					
		Gering exponiert		Mittelgradig exponiert		Stark exponiert	
		Anzahl	Zeilen (%)	Anzahl	Zeilen (%)	Anzahl	Zeilen (%)
**Geschlecht**	weiblich	368	68,0%	134	24,8%	39	7,2%
	männlich	343	70,0%	123	25,1%	24	4,9%
**Altersgruppen**	18 bis 40 Jahre	290	65,9%	129	29,3%	21	4,8%
	41 bis 60 Jahre	207	65,5%	79	25,0%	30	9,5%
	über 60 Jahre	214	77,8%	49	17,8%	12	4,4%
**Bildungsschicht**	Primärer Schulabschluss	72	73,5%	20	20,4%	6	6,1%
	Sekundärer Schulabschluss	373	68,8%	131	24,2%	38	7,0%
	Tertiärer Schulabschluss	263	67,8%	106	27,3%	19	4,9%
**Baulicher Schutz vor Verkehrslärm**	Keiner bis geringfügig	530	69,2%	187	24,4%	49	6,4%
	Gut bis komplett	161	68,2%	63	26,7%	12	5,1%
**Innenhof bzw. lärmabgewandte Seite**	Ja	462	65,9%	191	27,2%	48	6,8%
	Nein	232	75,3%	63	20,5%	13	4,2%

**Nachtschlaf**	Fenster geöffnet/gekippt	580	70,6%	198	24,1%	43	5,2%
	Fenster geschlossen	125	61,9%	57	28,2%	20	9,9%
**Lärmschutzfenster in den letzten 10 Jahren eingebaut**	Ja	114	61,0%	52	27,8%	21	11,2%
	Nein	483	71,9%	152	22,6%	37	5,5%
	weiß nicht/ k.A.	114	66,3%	53	30,8%	5	2,9%
**Lebensbedingungen (Summenscore)**	Nicht gut (*)	3	60,0%	0	0,0%	2	40,0%
	Durchschnittlich	425	63,4%	194	29,0%	51	7,6%
	Sehr gut	283	79,5%	63	17,7%	10	2,8%
**Zufriedenheit Wohnsituation insgesamt**	Nicht zufrieden	10	35,7%	9	32,1%	9	32,1%
	Durchschnittlich	147	57,0%	82	31,8%	29	11,2%
	Sehr zufrieden	553	74,3%	166	22,3%	25	3,4%
**Lebensqualität**	Nicht gut	7	70,0%	0 (*)	0,0%	3 (*)	30,0%
	Durchschnittlich	174	60,0%	82	28,3%	34	11,7%
	Sehr gut	526	72,6%	174	24,0%	25	3,4%
**Lärmempfindlichkeit**	Nicht lärmempfindlich	204	74,2%	53	19,3%	18	6,5%
	Durchschnittlich	403	69,0%	150	25,7%	31	5,3%
	Sehr lärmempfindlich	104	60,5%	54	31,4%	14	8,1%
**Gesundheitszustand**	Gut	518	68,7%	196	26,0%	40	5,3%
	Zufriedenstellend	130	69,9%	44	23,7%	12	6,5%
	Nicht gut	61	68,5%	17	19,1%	11	12,4%
**Zunahme Lärmbelastung**	Ja	261	59,9%	137	31,4%	38	8,7%
	Nein	450	75,6%	120	20,2%	25	4,2%
**Abnahme Lärmbelastung**	Ja	43	74,1%	13	22,4%	2 (*)	3,4%
	Nein	668	68,7%	244	25,1%	61	6,3%
**Lautheit Innsbruck im Vergleich**	Ruhiger	200	68,5%	75	25,7%	17	5,8%
	Gleich	391	71,0%	133	24,1%	27	4,9%
	Lauter	108	62,1%	47	27,0%	19	10,9%
**Notwendigkeit (weiterer) Maßnahmen zur Lärmreduktion**	Ja	450	65,1%	188	27,2%	53	7,7%
	Nein	149	77,6%	37	19,3%	6	3,1%
**Belästigung Nachbarschaftslärm**	Gering belästigt	660	89,7%	70	9,5%	6	0,8%
	Mittelgradig belästigt	48	19,8%	174	71,6%	21	8,6%
	Stark belästigt	3 (*)	5,8%	13	25,0%	36	69,2%

(*) Zu geringe Fallzahlen für solide Auswertungen

Die empfundene Ausgesetztheit gegenüber Nachbarschaftslärm steht auch in Beziehung zur Einschätzung der Güte der Nachbarschaftsbeziehungen im eigenen Wohnumfeld (und umgekehrt):

Abbildung 124: Empfundene Nachbarschaftslärmexposition und Güte der Nachbarschaftsbeziehungen im Mittelwertvergleich

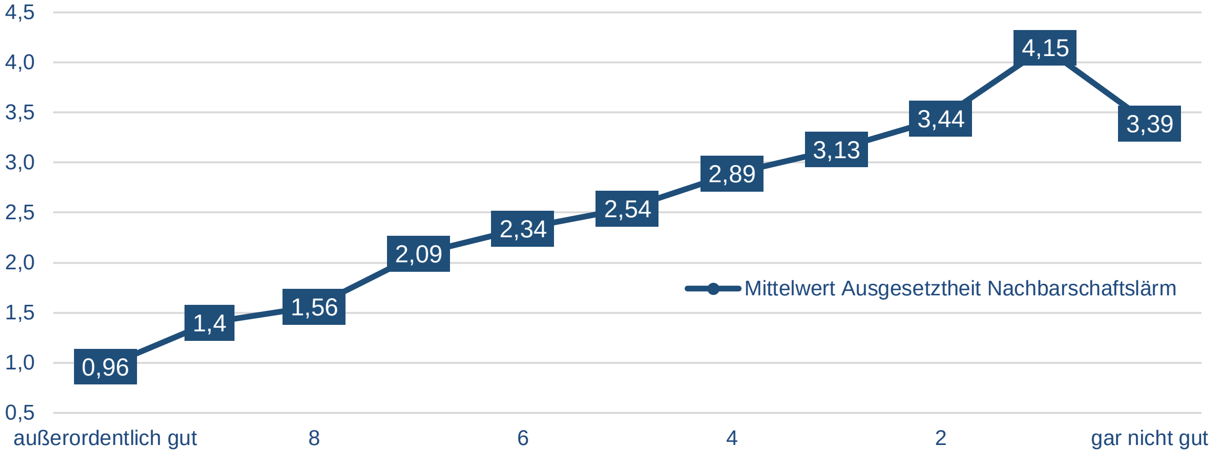

7. Lärmexposition durch Passanten und Gaststätten

Die Einschätzung der Ausgesetztheit der eigenen Wohnung / des eigenen Wohnhauses gegenüber dem Lärm von Passanten und Gaststätten fällt in ihren Ausmaßen nahezu deckungsgleich mit den Einschätzungen des Nachbarschaftslärms aus, liegt jedoch noch einmal geringfügig darunter:

Abbildung 125: Empfundene Lärmexposition durch Passanten und Gaststätten in Prozent

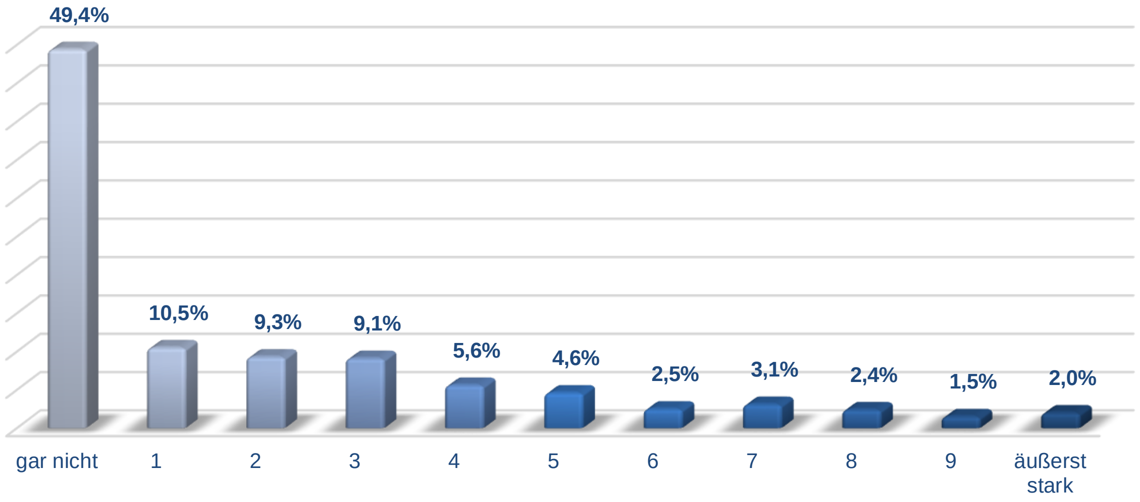

Tabelle 106: Empfundene Lärmexposition durch Passanten und Gaststätten gruppiert

		Häufigkeit	Gültige Prozente	Kumulierte Prozente
Gültig	Gering exponiert	713	69,2	69,2
	Mittelgradig exponiert	257	24,9	94,1
	Stark exponiert	61	5,9	100,0
	Gesamt	1031	100,0	

Wiederum sehen sich knapp 6% in ihrem Wohnumfeld als stark exponiert an (*highly exposed*: gruppierte Antwortkategorien 8, 9 und 10 [=*äußerst stark*]), der überwiegende Großteil der Befragten hingegen als nur gering ausgesetzt (über 70%).

Im Mittel liegen die Antworten der befragten Probandinnen und Probanden unterhalb der zweiten Antwortkategorie bei 1,91 (21,0%).

7.1. Lärmexposition Passanten / Gaststätten und Katastralgemeinden

Das Empfinden der eigenen Ausgesetztheit gegenüber dem Lärm von Passanten und Gaststätten unterscheidet sich am deutlichsten aller Expositionsfragen zwischen den einzelnen Katastralgemeinden Innsbrucks und ist in direkter Abhängigkeit von der Anzahl der vorhandenen Lokale, Sehenswürdigkeiten etc. zu sehen, wodurch sich die inneren Stadtbezirke deutlich von den äußeren unterscheiden:

Abbildung 126: Empfundene Lärmexposition durch Passanten und Gaststätten und Katastralgemeinden im Mittelwertvergleich

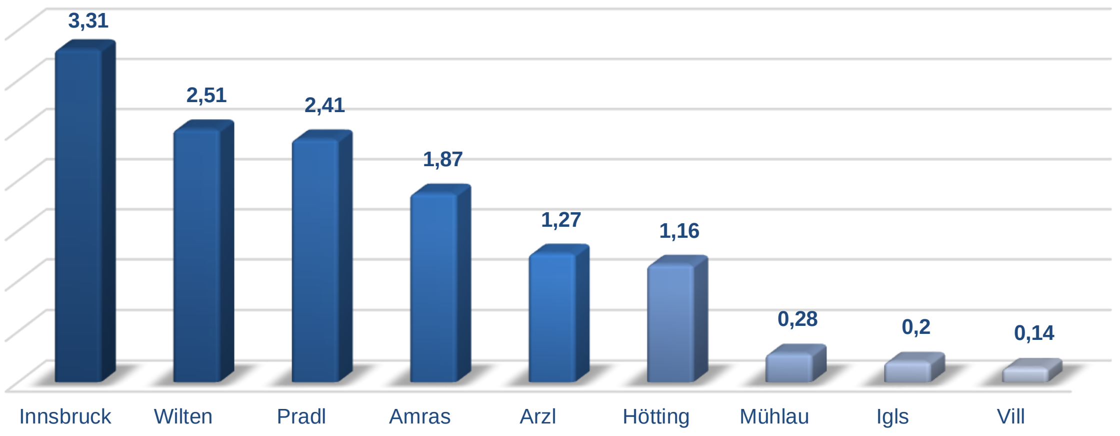

7.2. Lärmexposition Passanten / Gaststätten und einzelne Parameter

Die Ergebnisse der Zusammenschau mit ausgewählten anderen Variablen entsprechen weitestgehend den gemachten Auswertungen in Zusammenhang mit den anderen Lärmexpositionsfragen. Aufgrund der kleineren Fallzahlen der sich als stark vom Lärm verursacht von Passanten und Gaststätten exponiert empfindenden Befragten, kommt es in dieser Extremgruppe zu starken Streuungen:

Tabelle 107: Empfundene Lärmexposition durch Passanten und Gaststätten und ausgewählte Parameter (gruppiert)

		Ausgesetztheit Lärm von Passanten u. Gaststätten gruppiert					
		Gering exponiert		Mittelgradig exponiert		Stark exponiert	
		Anzahl	Zeilen (%)	Anzahl	Zeilen (%)	Anzahl	Zeilen (%)
**Geschlecht**	weiblich	373	68,9%	136	25,1%	32	5,9%
	männlich	340	69,4%	121	24,7%	29	5,9%

**Altersgruppen**	18 bis 40 Jahre	296	67,3%	114	25,9%	30	6,8%
	41 bis 60 Jahre	211	66,8%	82	25,9%	23	7,3%
	über 60 Jahre	206	74,9%	61	22,2%	8	2,9%
**Bildungsschicht**	Primärer Schulabschluss	62	63,3%	32	32,7%	4 (*)	4,1%
	Sekundärer Schulabschluss	378	69,7%	130	24,0%	34	6,3%
	Tertiärer Schulabschluss	271	69,8%	94	24,2%	23	5,9%
**Baulicher Schutz vor Verkehrslärm**	Keiner bis geringfügig	514	67,1%	199	26,0%	53	6,9%
	Gut bis komplett	178	75,4%	51	21,6%	7	3,0%
**Innenhof bzw. lärm-abgewandte Seite**	Ja	465	66,3%	198	28,2%	38	5,4%
	Nein	234	76,0%	51	16,6%	23	7,5%
**Nachtschlaf**	Fenster geöffnet/gekippt	594	72,4%	191	23,3%	36	4,4%
	Fenster geschlossen	114	56,4%	64	31,7%	24	11,9%
**Lärmschutzfenster in den letzten 10 Jahren eingebaut**	Ja	122	65,2%	49	26,2%	16	8,6%
	Nein	475	70,7%	159	23,7%	38	5,7%
	weiß nicht/ k.A.	116	67,4%	49	28,5%	7	4,1%
**Lebensbedingungen (Summenscore)**	Nicht gut (*)	2	40,0%	2	40,0%	1	20,0%
	Durchschnittlich	430	64,2%	196	29,3%	44	6,6%
	Sehr gut	281	78,9%	59	16,6%	16	4,5%
**Zufriedenheit Wohnsituation insgesamt**	Nicht zufrieden	15	53,6%	8	28,6%	5	17,9%
	Durchschnittlich	145	56,2%	88	34,1%	25	9,7%
	Sehr zufrieden	552	74,2%	161	21,6%	31	4,2%
**Lebensqualität**	Nicht gut	6	60,0%	1 (*)	10,0%	3 (*)	30,0%
	Durchschnittlich	175	60,3%	97	33,4%	18	6,2%
	Sehr gut	527	72,7%	158	21,8%	40	5,5%
**Zunahme Lärmbelastung**	Ja	264	60,6%	136	31,2%	36	8,3%
	Nein	449	75,5%	121	20,3%	25	4,2%
**Abnahme Lärmbelastung**	Ja	43	74,1%	13	22,4%	2 (*)	3,4%
	Nein	670	68,9%	244	25,1%	59	6,1%
**Lautheit Innsbruck im Vergleich**	Ruhiger	214	73,3%	68	23,3%	10	3,4%
	Gleich	392	71,1%	123	22,3%	36	6,5%
	Lauter	96	55,2%	63	36,2%	15	8,6%
**Notwendigkeit (weite-rer) Maßnahmen zur Lärmreduktion**	Ja	462	66,9%	175	25,3%	54	7,8%
	Nein	144	75,0%	44	22,9%	4 (*)	2,1%
**Belästigung Lärm durch Passan-ten und Gaststätten**	Gering belästigt	667	89,2%	76	10,2%	5	0,7%
	Mittelgradig belästigt	45	19,3%	171	73,4%	17	7,3%
	Stark belästigt	1 (*)	2,0%	10	20,0%	39	78,0%

(*) Zu geringe Fallzahlen für solide Auswertungen

8. Exposition gegenüber Erschütterungen

Das Empfinden einer verstärkten Erschütterungsexposition beginnt bereits bei der Fühlbarkeitsschwelle und zieht im Regelfall sehr starke Belästigungsreaktionen nach sich (siehe dazu auch das folgende Kapitel *Belästigung / Störung durch Lärm*). Neben der direkten Übertragung von Erschütterungen sind auch Erschütterungen verursacht von sekundärem Luftschall zu berücksichtigen.

Knapp 80% aller befragten Innsbruckerinnen und Innsbrucker sehen ihr Wohnumfeld als nur gering erschütterungsexponiert an, 4,1% als Erschütterungen stark ausgesetzt:

Abbildung 127: Empfundene Exposition gegenüber Erschütterungen in Prozent

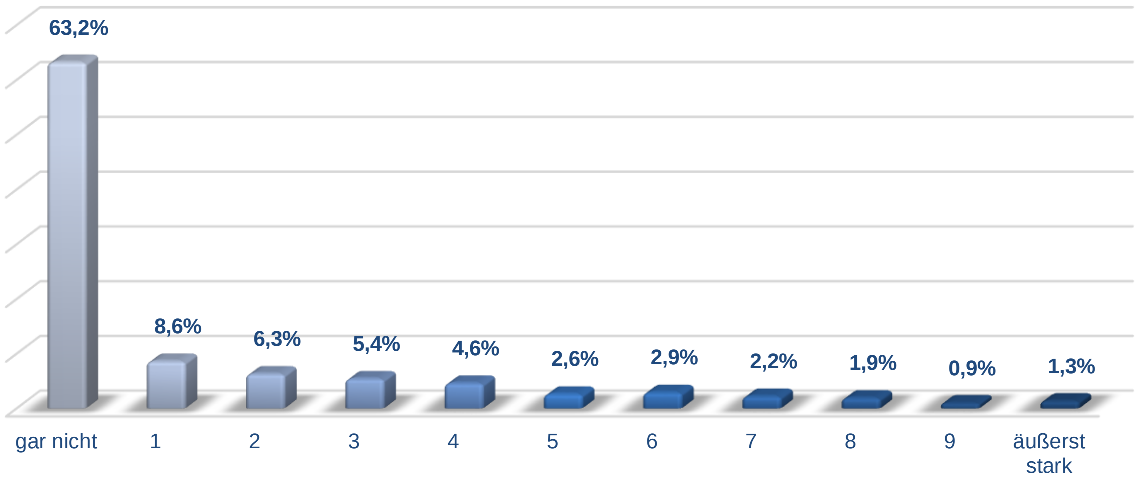

Im Mittel liegen die Antworten der befragten Probandinnen und Probanden bei 1,38 (15,2%) und damit kommt der Mittelwert innerhalb der ersten Antwortkategorie *gar nicht ausgesetzt* zu liegen.

Tabelle 108: Empfundene Exposition gegenüber Erschütterungen gruppiert

		Häufigkeit	Gültige Prozente	Kumulierte Prozente
Gültig	Gering exponiert	806	78,2	78,2
	Mittelgradig exponiert	183	17,7	95,9
	Stark exponiert	42	4,1	100,0
	Gesamt	1031	100,0	

8.1. Erschütterungsexposition und Katastralgemeinden

Das größte Ausmaß an Erschütterungsexposition wird in Wilten und Innsbruck empfunden, das geringste in Vill und Mühlau:

Abbildung 128: Empfundene Erschütterungsexposition und Katastralgemeinden im Mittelwertvergleich

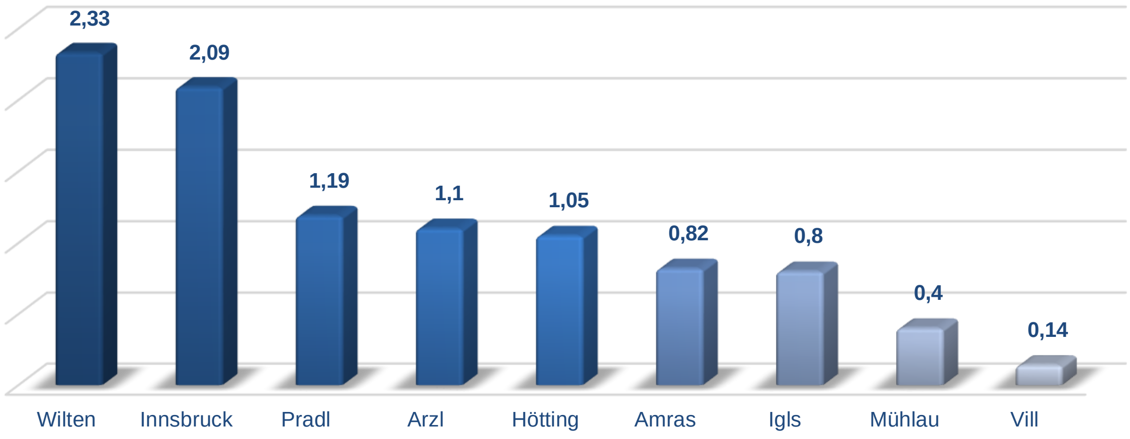

8.2. Erschütterungsexposition und ausgewählte Parameter

Auch bei der empfundenen Erschütterungsexposition kommt es in der Zusammenschau mit anderen ausgewählten Variablen zu keinen größeren Abweichungen im Vergleich zu den gemachten Auswertungen in Verbindung mit den Lärmexpositionsfragen. Die Gruppe der sich stark Erschütterungen ausgesetzt empfindenden Befragten umfasst nur einen sehr begrenzten Umfang (N=42). Aus diesem Grund sind einige der Querauswertungen in Bezug zu dieser Extremgruppe aufgrund kleiner Fallzahlen nur bedingt zu interpretieren:

Tabelle 109: Empfundene Erschütterungsexposition und ausgewählte Parameter (gruppiert)

		Ausgesetztheit Erschütterungen gesamt gruppiert					
		Gering exponiert		Mittelgradig exponiert		Stark exponiert	
		Anzahl	Zeilen (%)	Anzahl	Zeilen (%)	Anzahl	Zeilen (%)
**Geschlecht**	weiblich	408	75,4%	104	19,2%	29	5,4%
	männlich	398	81,2%	79	16,1%	13	2,7%
**Altersgruppen**	18 bis 40 Jahre	339	77,0%	86	19,5%	15	3,4%
	41 bis 60 Jahre	237	75,0%	61	19,3%	18	5,7%
	über 60 Jahre	230	83,6%	36	13,1%	9	3,3%
**Bildungsschicht**	Primärer Schulabschluss	84	85,7%	9	9,2%	5	5,1%
	Sekundärer Schulabschluss	419	77,3%	100	18,5%	23	4,2%
	Tertiärer Schulabschluss	300	77,3%	74	19,1%	14	3,6%
**Baulicher Schutz vor Verkehrslärm**	Keiner bis geringfügig	584	76,2%	144	18,8%	38	5,0%
	Gut bis komplett	197	83,5%	35	14,8%	4	1,7%
**Innenhof bzw. lärmabgewandte Seite**	Ja	552	78,7%	123	17,5%	26	3,7%
	Nein	237	76,9%	55	17,9%	16	5,2%
**Nachtschlaf**	Fenster geöffnet/gekippt	659	80,3%	140	17,1%	22	2,7%
	Fenster geschlossen	141	69,8%	42	20,8%	19	9,4%
**Lärmschutzfenster in den letzten 10 Jahren**	Ja	131	70,1%	41	21,9%	15	8,0%
	Nein	535	79,6%	117	17,4%	20	3,0%

**eingebaut**	weiß nicht/ k.A.	140	81,4%	25	14,5%	7	4,1%
**Lebensbedingungen (Summenscore)**	Nicht gut	3	60,0%	2	40,0%	0	0,0%
	Durchschnittlich	499	74,5%	140	20,9%	31	4,6%
	Sehr gut	304	85,4%	41	11,5%	11	3,1%
**Zufriedenheit Wohnsituation insgesamt**	Nicht zufrieden	14	50,0%	8	28,6%	6	21,4%
	Durchschnittlich	176	68,2%	63	24,4%	19	7,4%
	Sehr zufrieden	616	82,8%	111	14,9%	17	2,3%
**Lebensqualität**	Nicht gut	4	40,0%	4	40,0%	2	20,0%
	Durchschnittlich	204	70,3%	70	24,1%	16	5,5%
	Sehr gut	593	81,8%	108	14,9%	24	3,3%
**Lärmempfindlichkeit**	Nicht lärmempfindlich	233	84,7%	32	11,6%	10	3,6%
	Durchschnittlich	446	76,4%	114	19,5%	24	4,1%
	Sehr lärmempfindlich	127	73,8%	37	21,5%	8	4,7%
**Gesundheitszustand**	Gut	595	78,9%	134	17,8%	25	3,3%
	Zufriedenstellend	148	79,6%	30	16,1%	8	4,3%
	Nicht gut	61	68,5%	19	21,3%	9	10,1%
**Zunahme Lärmbelastung**	Ja	310	71,1%	99	22,7%	27	6,2%
	Nein	496	83,4%	84	14,1%	15	2,5%
**Abnahme Lärmbelastung**	Ja	51	87,9%	6	10,3%	1	1,7%
	Nein	755	77,6%	177	18,2%	41	4,2%
**Lautheit Innsbruck im Vergleich**	Ruhiger	244	83,6%	41	14,0%	7	2,4%
	Gleich	429	77,9%	95	17,2%	27	4,9%
	Lauter	119	68,4%	47	27,0%	8	4,6%
**Notwendigkeit (weiterer) Maßnahmen zur Lärmreduktion**	Ja	508	73,5%	145	21,0%	38	5,5%
	Nein	164	85,4%	25	13,0%	3	1,6%
**Belästigung Erschütterungen**	Gering belästigt	789	91,5%	69	8,0%	4	0,5%
	Mittelgradig belästigt	15	11,1%	104	77,0%	16	11,9%
	Stark belästigt	2	5,9%	10	29,4%	22	64,7%

Anm.: In mehreren Subgruppen sind die Fallzahlen für solide Auswertungen zu gering

9. Schienenverkehrslärmexposition

70% aller Befragten empfinden sich in ihrem Wohnumfeld als überhaupt nicht dem Schienenverkehrslärm ausgesetzt. 82% sehen sich gruppiert als in ihrem Wohnumfeld gering dem Lärm ausgehend von der Eisenbahn ausgesetzt (gruppierte Antwortkategorien 0 [=*gar nicht*], 1 und 2), 2,8% als stark ausgesetzt (*highly exposed*: gruppierte Antwortkategorien 8, 9 und 10 [=*äußerst stark*]):

Abbildung 129: Empfundene Schienenverkehrslärmexposition in Prozent

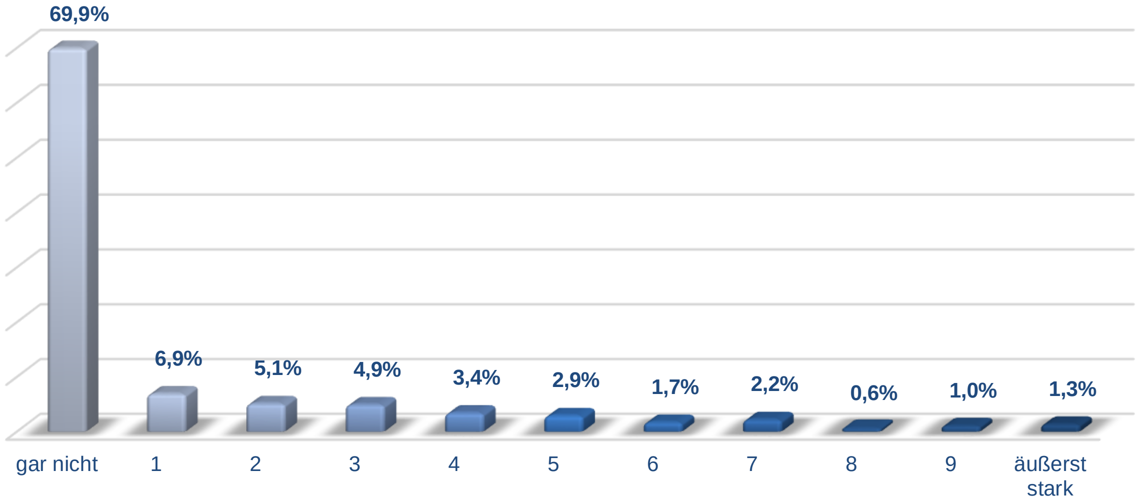

Im Mittel liegen die Antworten der befragten Probandinnen und Probanden bei 1,12 (12,3%).

Tabelle 110: Empfundene Schienenverkehrslärmexposition gruppiert

		Häufigkeit	Gültige Prozente	Kumulierte Prozente
Gültig	Gering exponiert	845	82,0	82,0
	Mittelgradig exponiert	157	15,2	97,2
	Stark exponiert	29	2,8	100,0
	Gesamt	1031	100,0	

9.1. Schienenlärmexposition und Schienenlärmbelastung

In der Zusammenschau zwischen dem Empfinden der Ausgesetztheit im eigenen Wohnumfeld und der gegebenen Schienenlärmbelastung zeigt sich bis zu 24h-Lärmpegeln von über 50dB nur eine sehr eingeschränkte Steigerung des Gefühls der eigenen Exposition:

Abbildung 130: Empfundene Schienenverkehrslärmexposition und 24h-Schienenverkehrslärmbelastung L_{den} in 10dB-Schritten im Mittelwertvergleich

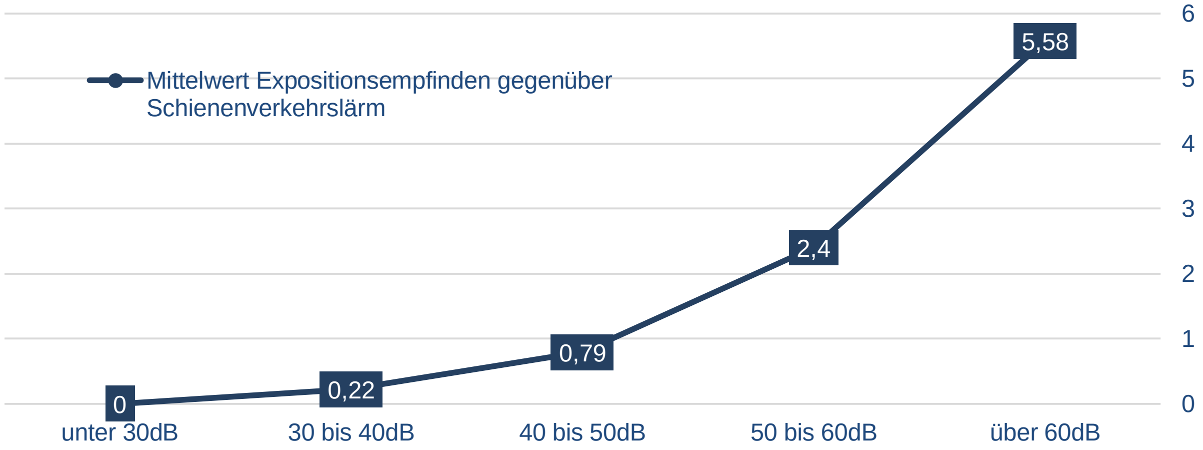

Im Nachtzeitraum liegt dieser „Schwellenwert“ niedriger. Das eigene Expositionsempfinden gegenüber Schienenverkehrslärm steigt jedoch ab diesen Werten sehr stark an.

Im Vergleich zum Flugverkehrslärm und etwas eingeschränkter auch zum Straßenverkehrslärm ist von einer größeren Toleranz von Dauerschallpegeln unter 50dB ausgehend vom Schienenverkehr auszugehen, bis Befragte für sich selbst eine mittelgradige oder darüber hinausgehende Lärmexposition aus dem Schienenverkehr annehmen:

Abbildung 131: Empfundene Schienenverkehrslärmexposition und nächtliche Schienenverkehrslärmbelastung L_n in 10dB-Schritten im Mittelwertvergleich

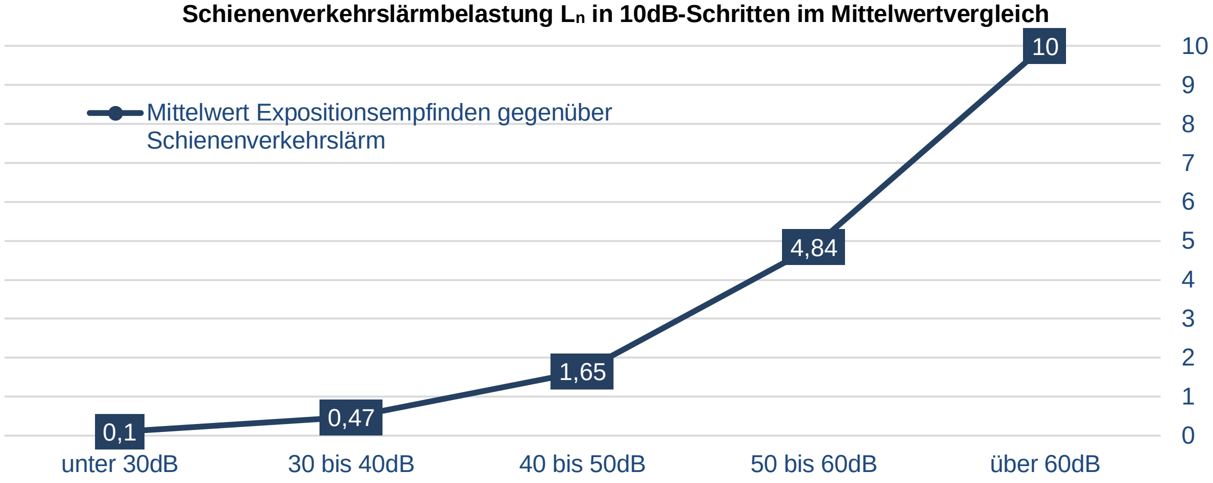

Anm.: Bei den mit über 60dB im Nachtzeitraum stark belasteten Befragten handelt es sich um 4 Personen, weshalb die Aussagekraft der statistischen Auswertung für dieses Datum stark eingeschränkt ist.

9.2. Schienenlärmexposition und Katastralgemeinden

Entsprechend der gegebenen Schienenverkehrslärmbelastung zeigt sich das mit Abstand stärkste Schienenlärmexpositionsempfinden in Wilten. Die geringste Ausgesetztheit gegenüber Schienenlärm im eigenen Wohnumfeld empfinden die Befragten in Igls, Vill und Amras:

Abbildung 132: Empfundene Schienenverkehrslärmexposition und Katastralgemeinden im Mittelwertvergleich

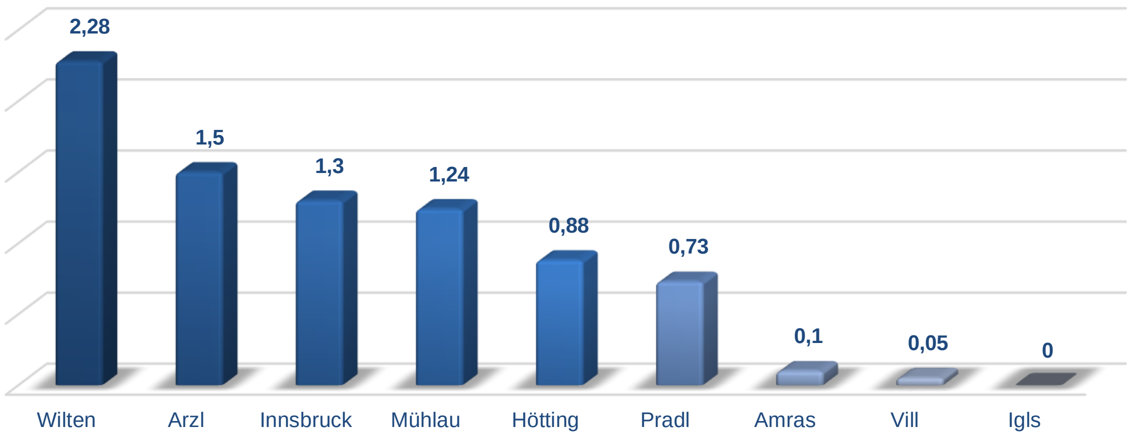

9.3. Schienenlärmexposition und ausgewählte Parameter

Die Auswertungen der empfundenen Schienenverkehrslärmexposition in Zusammenschau mit anderen Variablen entsprechen weitestgehend den Analysen der anderen Lärmexpositionsquellen in den vorangegangenen Subkapiteln.

Die Gruppe der sich stark schienenverkehrslärmexponiert empfindenden Befragten umfasst nur einen sehr begrenzten Umfang (N=29). Aus diesem Grund sind einige der Querauswertungen in Bezug zu dieser Extremgruppe aufgrund kleiner Fallzahlen nur bedingt zu interpretieren:

Tabelle 111: Empfundene Schienenlärmexposition und ausgewählte Parameter (gruppiert)

		Ausgesetztheit Schienenverkehrslärm gruppiert					
		Gering exponiert		Mittelgradig exponiert		Stark exponiert	
		Anzahl	Zeilen (%)	Anzahl	Zeilen (%)	Anzahl	Zeilen (%)
**Geschlecht**	weiblich	442	81,7%	84	15,5%	15	2,8%
	männlich	403	82,2%	73	14,9%	14	2,9%
**Altersgruppen**	18 bis 40 Jahre	359	81,6%	65	14,8%	16	3,6%
	41 bis 60 Jahre	254	80,4%	51	16,1%	11	3,5%
	über 60 Jahre	232	84,4%	41	14,9%	2	0,7%
**Bildungsschicht**	Primärer Schulabschluss	88	89,8%	7	7,1%	3	3,1%
	Sekundärer Schulabschluss	441	81,4%	92	17,0%	9	1,7%
	Tertiärer Schulabschluss	313	80,7%	58	14,9%	17	4,4%
**Baulicher Schutz vor Verkehrslärm**	Keiner bis geringfügig	620	80,9%	124	16,2%	22	2,9%
	Gut bis komplett	202	85,6%	27	11,4%	7	3,0%
**Innenhof bzw. lärmabgewandte Seite**	Ja	590	84,2%	93	13,3%	18	2,6%
	Nein	239	77,6%	58	18,8%	11	3,6%
**Nachtschlaf**	Fenster geöffnet/gekippt	676	82,3%	124	15,1%	21	2,6%
	Fenster geschlossen	161	79,7%	33	16,3%	8	4,0%
**Lärmschutzfenster in den letzten 10 Jahren eingebaut**	Ja	144	77,0%	37	19,8%	6	3,2%
	Nein	556	82,7%	100	14,9%	16	2,4%
	weiß nicht/ k.A.	145	84,3%	20	11,6%	7	4,1%
**Lebensbedingungen (Summenscore)**	Nicht gut	2	40,0%	3	60,0%	0	0,0%
	Durchschnittlich	537	80,1%	108	16,1%	25	3,7%
	Sehr gut	306	86,0%	46	12,9%	4	1,1%
**Zufriedenheit Wohnsituation insgesamt**	Nicht zufrieden	19	67,9%	7	25,0%	2	7,1%
	Durchschnittlich	204	79,1%	43	16,7%	11	4,3%
	Sehr zufrieden	621	83,5%	107	14,4%	16	2,2%
**Lärmempfindlichkeit**	Nicht lärmempfindlich	234	85,1%	32	11,6%	9	3,3%
	Durchschnittlich	475	81,3%	93	15,9%	16	2,7%
	Sehr lärmempfindlich	136	79,1%	32	18,6%	4	2,3%
**Zunahme Lärmbelastung**	Ja	351	80,5%	72	16,5%	13	3,0%
	Nein	494	83,0%	85	14,3%	16	2,7%
**Abnahme Lärmbelastung**	Ja	49	84,5%	7	12,1%	2	3,4%
	Nein	796	81,8%	150	15,4%	27	2,8%
**Lautheit Innsbruck im**	Ruhiger	242	82,9%	46	15,8%	4	1,4%

**Vergleich**	Gleich	457	82,9%	78	14,2%	16	2,9%
	Lauter	132	75,9%	33	19,0%	9	5,2%
**Notwendigkeit (weiterer) Maßnahmen zur Lärmreduktion**	Ja	548	79,3%	119	17,2%	24	3,5%
	Nein	166	86,5%	25	13,0%	1	0,5%
**Belästigung Schienenverkehrslärm**	Gering belästigt	782	93,1%	54	6,4%	4	0,5%
	Mittelgradig belästigt	54	33,5%	97	60,2%	10	6,2%
	Stark belästigt	9	30,0%	6	20,0%	15	50,0%

Anm.: In mehreren Subgruppen sind die Fallzahlen für solide Auswertungen zu gering

10. Autobahnlärmexposition

Etwas weniger als 2% aller befragten Innsbruckerinnen und Innsbrucker sehen sich in ihrem Wohnumfeld dem Autobahnlärm stark exponiert, über 90% nur gering, davon knapp 80% gar nicht ausgesetzt:

Abbildung 133: Empfundene Autobahnlärmexposition in Prozent

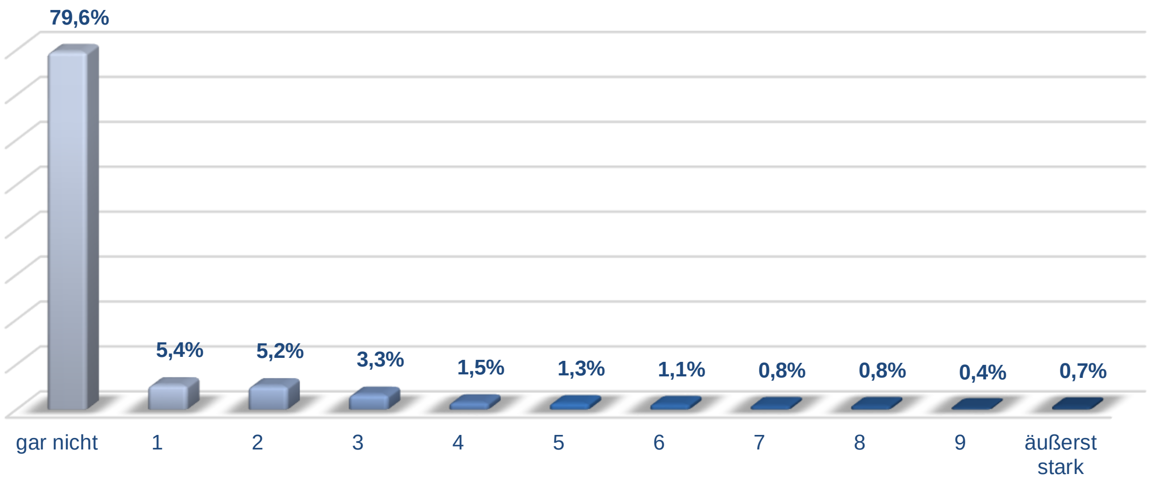

Im Mittel liegen die Antworten der befragten Probandinnen und Probanden bei 0,66 (7,3%).

Tabelle 112: Empfundene Autobahnlärmexposition gruppiert

		Häufigkeit	Gültige Prozente	Kumulierte Prozente
Gültig	Gering exponiert	931	90,3	90,3
	Mittelgradig exponiert	81	7,9	98,2
	Stark exponiert	19	1,8	100,0
	Gesamt	1031	100,0	

10.1. Autobahnlärmexposition und Katastralgemeinden

Das im Mittel stärkste Autobahnlärmexpositionsempfinden zeigt sich in Wilten, gefolgt von Vill. Die geringste Ausgesetztheit gegenüber Autobahnlärm im eigenen Wohnumfeld empfinden die Befragten in der Innenstadt (Katastralgemeinde Innsbruck), sowie in Igls, Pradl und Mühlau:

Abbildung 134: Empfundene Autobahnlärmexposition und Katastralgemeinden im Mittelwertvergleich

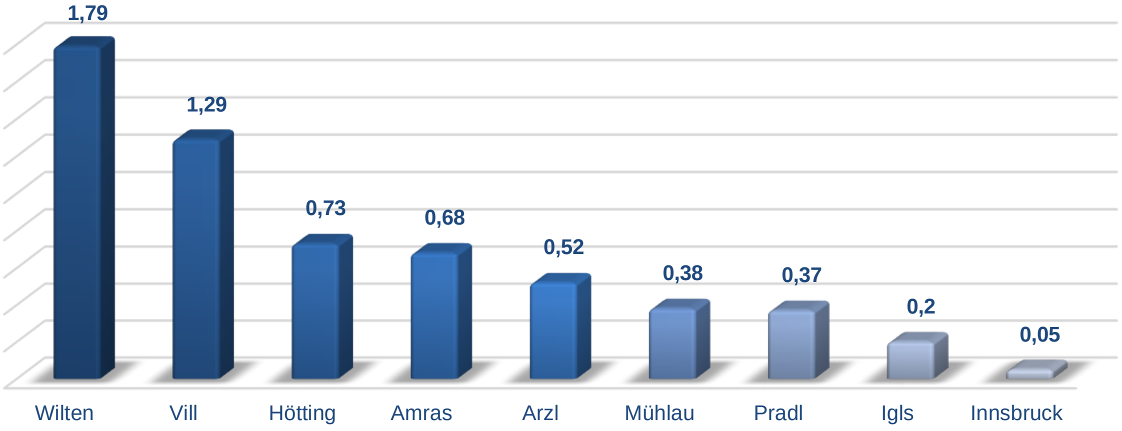

10.2. Autobahnlärmexposition und ausgewählte Parameter

Die Gruppe der sich stark autobahnlärmexponiert (*highly exposed*) empfindenden Befragten umfasst lediglich 19 Personen. In Bezug zu anderen Variablen gesetzt, vermindern sich die Fallzahlen auf eine zu kleine Anzahl für solide Auswertungen, weshalb diese Extremgruppe in der nachstehenden Tabelle nicht berücksichtigt wurde:

Tabelle 113: Empfundene Autobahnlärmexposition und ausgewählte Parameter (gruppiert)

		Ausgesetztheit Autobahnlärm gruppiert			
		Gering exponiert		Mittelgradig exponiert	
		Anzahl	Zeilen (%)	Anzahl	Zeilen (%)
**Geschlecht**	weiblich	489	90,4%	40	7,4%
	männlich	442	90,2%	41	8,4%
**Altersgruppen**	18 bis 40 Jahre	398	90,5%	37	8,4%
	41 bis 60 Jahre	287	90,8%	20	6,3%
	über 60 Jahre	246	89,5%	24	8,7%
**Bildungsschicht**	Primärer Schulabschluss	89	90,8%	4	4,1%
	Sekundärer Schulabschluss	491	90,6%	43	7,9%
	Tertiärer Schulabschluss	348	89,7%	34	8,8%
**Baulicher Schutz vor Verkehrslärm**	Keiner bis geringfügig	681	88,9%	67	8,7%
	Gut bis komplett	224	94,9%	11	4,7%
**Innenhof bzw. lärm-abgewandte Seite**	Ja	651	92,9%	40	5,7%
	Nein	260	84,4%	40	13,0%
**Lärmschutzfenster**	Ja	160	85,6%	20	10,7%

**in den letzten 10**	Nein	610	90,8%	51	7,6%
**Jahren eingebaut**	weiß nicht/ k.A.	161	93,6%	10	5,8%
**Zufriedenheit**	Nicht zufrieden	22	78,6%	4	14,3%
**Wohnsituation**	Durchschnittlich	227	88,0%	24	9,3%
**insgesamt**	Sehr zufrieden	681	91,5%	53	7,1%
	Nicht gut	8	80,0%	2	20,0%
**Lebensqualität**	Durchschnittlich	258	89,0%	24	8,3%
	Sehr gut	659	90,9%	55	7,6%
	Nicht lärmempfindlich	259	94,2%	12	4,4%
**Lärmempfindlichkeit**	Durchschnittlich	524	89,7%	48	8,2%
	Sehr lärmempfindlich	148	86,0%	21	12,2%
	Ruhiger	268	91,8%	19	6,5%
**Lautheit Innsbruck im Vergleich**	Gleich	503	91,3%	41	7,4%
	Lauter	147	84,5%	20	11,5%
**Notwendigkeit (weiterer) Maßnahmen**	Ja	614	88,9%	61	8,8%
**zur Lärmreduktion**	Nein	178	92,7%	12	6,3%
	Gering belästigt	915	96,6%	30	3,2%
**Belästigung Autobahnlärm**	Mittelgradig belästigt	13	21,0%	44	71,0%
	Stark belästigt	3	13,6%	7	31,8%

Anm.: In mehreren Subgruppen sind die Fallzahlen für solide Auswertungen zu gering

11. Lärmexposition durch Gewerbe und Industrie

Am geringsten im Vergleich aller Lärmquellen sehen die befragten Innsbruckerinnen und Innsbrucker ihre Wohnungen dem Lärm von Gewerbe und Industrie ausgesetzt:

Abbildung 135: Empfundene Lärmexposition durch Gewerbe und Industrie in Prozent

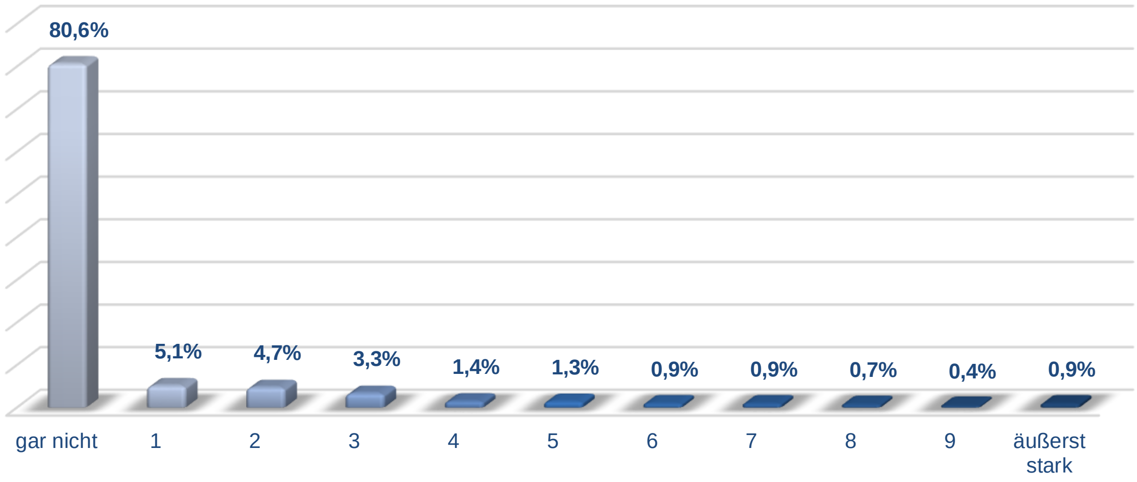

Über 80% erkennen gar keine Lärmexposition durch Gewerbe und Industrie, über 90% nur eine geringe (gruppierte Antwortkategorien 0 [=*gar nicht*], 1 und 2), 2% sehen ihr Wohnumfeld als stark ausgesetzt an (*highly exposed*: gruppierte Antwortkategorien 8, 9 und 10 [=*äußerst stark*]):

Tabelle 114: Empfundene Lärmexposition durch Gewerbe und Industrie gruppiert

		Häufigkeit	Gültige Prozente	Kumulierte Prozente
Gültig	Gering exponiert	932	90,4	90,4
	Mittelgradig exponiert	79	7,7	98,1
	Stark exponiert	20	1,9	100,0
	Gesamt	1031	100,0	

Im Mittel liegen die Antworten der befragten Probandinnen und Probanden bei 0,65 (7,2%).

11.1. Lärmexposition Gewerbe und Industrie und Katastralgemeinden

Das Empfinden der eigenen Ausgesetztheit gegenüber dem Lärm von Gewerbe und Industrie ist in Vill und Mühlau am geringsten und in Wilten, Innsbruck und Arzl am stärksten ausgeprägt:

Abbildung 136: Empfundene Lärmexposition durch Gewerbe und Industrie und Katastralgemeinden im Mittelwertvergleich

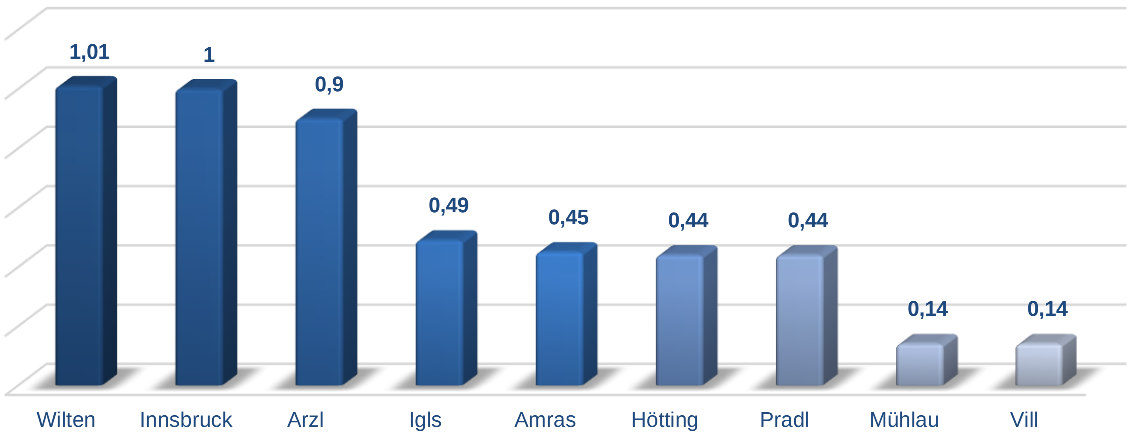

11.2. Lärmexposition Gewerbe und Industrie und einzelne Parameter

Die Gruppe der sich stark dem Lärm von Gewerbe und Industrie exponiert empfindenden Befragten (*highly exposed*) umfasst lediglich 20 Personen. In Bezug zu anderen Variablen gesetzt, vermindern sich die Fallzahlen auf eine zu kleine Anzahl für solide Auswertungen, weshalb diese Extremgruppe in der nachstehenden Tabelle nicht berücksichtigt wurde:

Tabelle 115: Empfundene Lärmexposition durch Gewerbe und Industrie und ausgewählte Parameter

		Ausgesetztheit Lärm von Gewerbe und Industrie gruppiert			
		Gering exponiert		Mittelgradig exponiert	
		Anzahl	Zeilen (%)	Anzahl	Zeilen (%)
**Geschlecht**	weiblich	493	91,1%	36	6,7%
	männlich	439	89,6%	43	8,8%
**Altersgruppen**	18 bis 40 Jahre	401	91,1%	32	7,3%
	41 bis 60 Jahre	284	89,9%	24	7,6%
	über 60 Jahre	247	89,8%	23	8,4%
**Bildungsschicht**	Primärer Schulabschluss	89	90,8%	7	7,1%
	Sekundärer Schulabschluss	492	90,8%	40	7,4%
	Tertiärer Schulabschluss	348	89,7%	32	8,2%
**Baulicher Schutz vor Verkehrslärm**	Keiner bis geringfügig	678	88,5%	69	9,0%
	Gut bis komplett	225	95,3%	10	4,2%
**Innenhof bzw. lärm-abgewandte Seite**	Ja	639	91,2%	56	8,0%
	Nein	271	88,0%	23	7,5%
**Lebensbedingungen (Summenscore)**	Nicht gut	4	80,0%	0	0,0%
	Durchschnittlich	595	88,8%	58	8,7%
	Sehr gut	333	93,5%	21	5,9%
**Zufriedenheit Wohnsituation insgesamt**	Nicht zufrieden	20	71,4%	7	25,0%
	Durchschnittlich	219	84,9%	28	10,9%
	Sehr zufrieden	693	93,1%	43	5,8%
**Lebensqualität**	Nicht gut	8	80,0%	1	10,0%
	Durchschnittlich	251	86,6%	34	11,7%
	Sehr gut	667	92,0%	44	6,1%
**Lärmempfindlichkeit**	Nicht lärmempfindlich	256	93,1%	17	6,2%
	Durchschnittlich	523	89,6%	46	7,9%
	Sehr lärmempfindlich	153	89,0%	16	9,3%
**Zunahme Lärmbelastung**	Ja	376	86,2%	44	10,1%
	Nein	556	93,4%	35	5,9%
**Abnahme Lärmbelastung**	Ja	58	100,0%	0	0,0%
	Nein	874	89,8%	79	8,1%
**Lautheit Innsbruck im Vergleich**	Ruhiger	275	94,2%	9	3,1%
	Gleich	494	89,7%	47	8,5%
	Lauter	149	85,6%	23	13,2%
**Notwendigkeit (weiterer) Maßnahmen zur Lärmreduktion**	Ja	611	88,4%	62	9,0%
	Nein	183	95,3%	8	4,2%
**Belästigung Lärm von Gewerbe und Industrie**	Gering belästigt	895	95,5%	38	4,1%
	Mittelgradig belästigt	36	45,6%	39	49,4%
	Stark belästigt	1	6,7%	2	13,3%

Anm.: In mehreren Subgruppen sind die Fallzahlen für solide Auswertungen zu gering

Belästigung / Störung durch Lärm

1. Überblick Belästigung / Störung durch Lärm

1.1. Belästigung nach Lärmquelle

Die Einschätzung der Belästigung / Störung durch Lärm wurde für jede einzelne Lärmquelle mittels einer 11-teiligen Skala von 0=*belästigt / stört überhaupt nicht* bis 10=*belästigt / stört besonders stark* abgefragt.

Im Mittelwertvergleich zeigt sich, dass die Belästigung / Störung durch den Flugverkehrslärm am höchsten eingeschätzt wird, gefolgt von der Belästigung durch den Straßenverkehrslärm. Die weiteren Lärmquellen folgen mit deutlichem Abstand. Die Störung durch Nachbarschaftslärm und den Lärm verursacht durch Passanten und Gaststätten fällt geringer aus, als die Belästigungswahrnehmung von Baustellen. Wiederum in ihrer Ausprägung deutlich dahinter schließen sich Schienenverkehrslärm und die Störung durch Erschütterungen an. Die Belästigungsreaktion auf Lärm aus Gewerbe und Industrie und durch Autobahnlärm fällt im Vergleich noch einmal deutlich geringer aus:[10]

Abbildung 137: Mittelwertvergleich der Belästigung nach Quelle

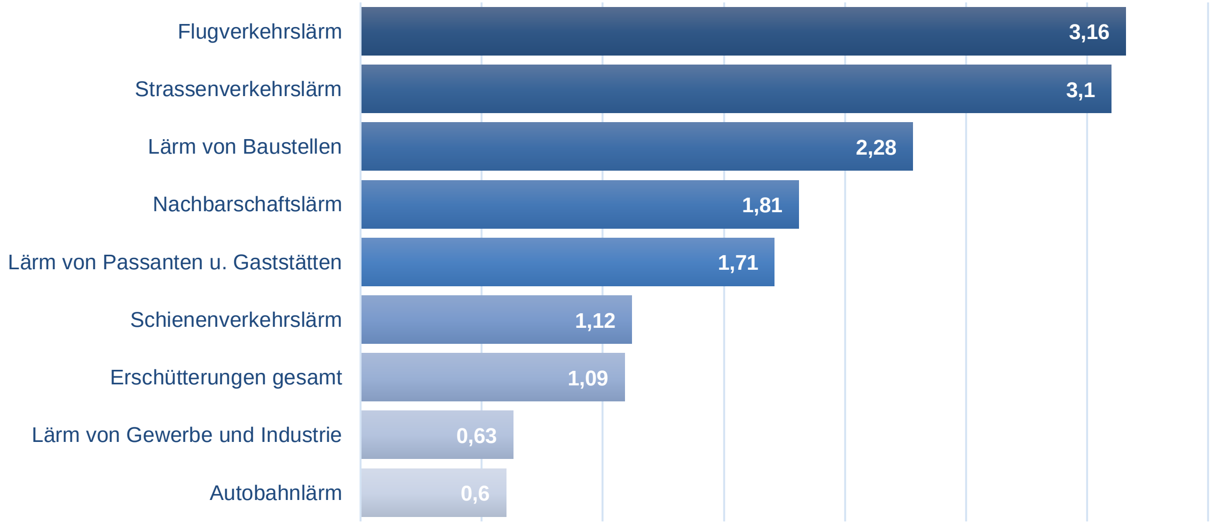

Tabelle 116: Mittelwerte der Belästigung nach Quelle

	Flug-verkehr	Strassen-verkehr	Bau-stellen	Nach-barschaft	Passanten u. Gaststätten	Schienen-verkehr	Erschütte-rungen	Gewerbe u. Industrie	Auto-bahn
Mittelwert	3,16	3,10	2,28	1,81	1,71	1,12	1,09	,63	,60
N	1031	1031	1030	1031	1031	1031	1031	1031	1031
Standard-abweichung	2,868	2,893	3,006	2,457	2,473	2,151	2,171	1,672	1,702
Standardfehler des MW	,089	,090	,094	,077	,077	,067	,068	,052	,053

[10] Anm.: Die eigenständige Frage nach der Belästigung / Störung durch *Lärm insgesamt* wird in den folgenden vergleichenden Zusammenstellungen der einzelnen Lärmquellen ausgeklammert und im anschließenden Unterkapitel dargestellt.

Die Vergleiche der Belästigung nach Quelle decken sich weitgehend mit den Auswertungen zur empfundenen Exposition, was auch die Validität und Reliabilität der Items unterstreicht. Der Mittelwert aller Antworten über alle 9 Fragen hinweg liegt bei 1,72 (18,9%).

Kategorisiert in 3 Belästigungsgruppen:

– ***gering belästigt****:* gruppierte Antwortkategorien 0 [=*belästigt / stört überhaupt nicht*]), 1 und 2
– ***mittelgradig belästigt***: gruppierte Antwortkategorien 3 bis 7
– ***stark belästigt (highly annoyed)****:* gruppierte Antwortkategorien 8, 9 und 10 [=*belästigt / stört besonders stark*])

stellt sich die Belästigungsreaktion nach Quelle wie folgt dar:

Abbildung 138: Belästigung nach Quelle kategorisiert in 3 Expositionsgruppen in Prozent

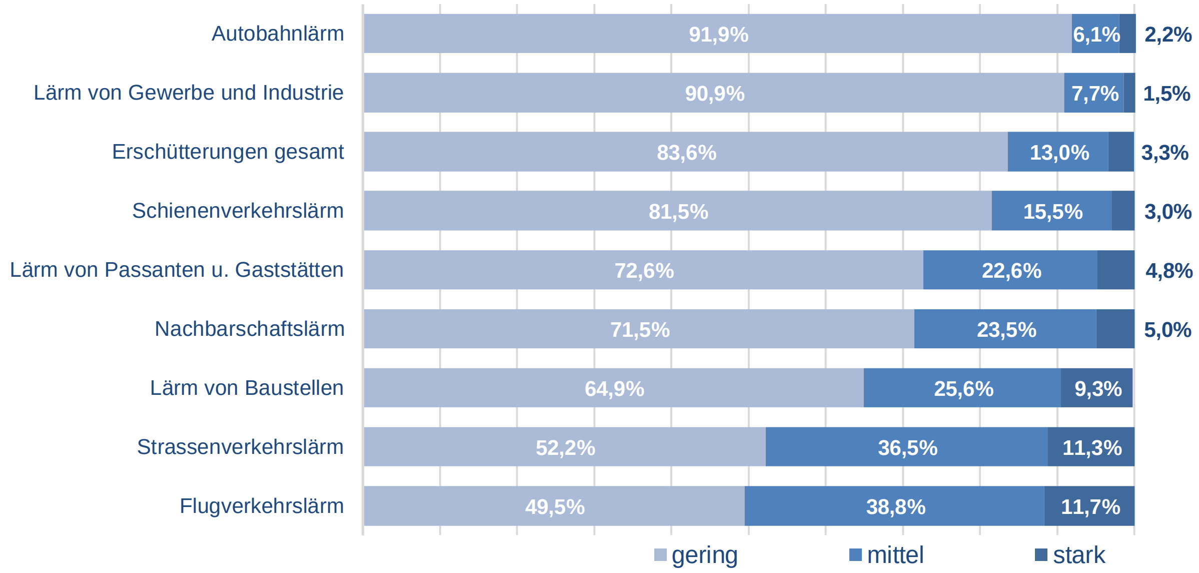

* Nach Belästigungsausmaß (Mittelwert) aufsteigend sortiert

Wird nur die Extremgruppe der sich stark belästigt fühlenden Befragten (*highly annoyed) betrachtet,* zeigt sich im Vergleich der Quellen nahezu dasselbe Bild:

Abbildung 139: Extremgruppenvergleich *highly annoyed* nach Quelle in Prozent

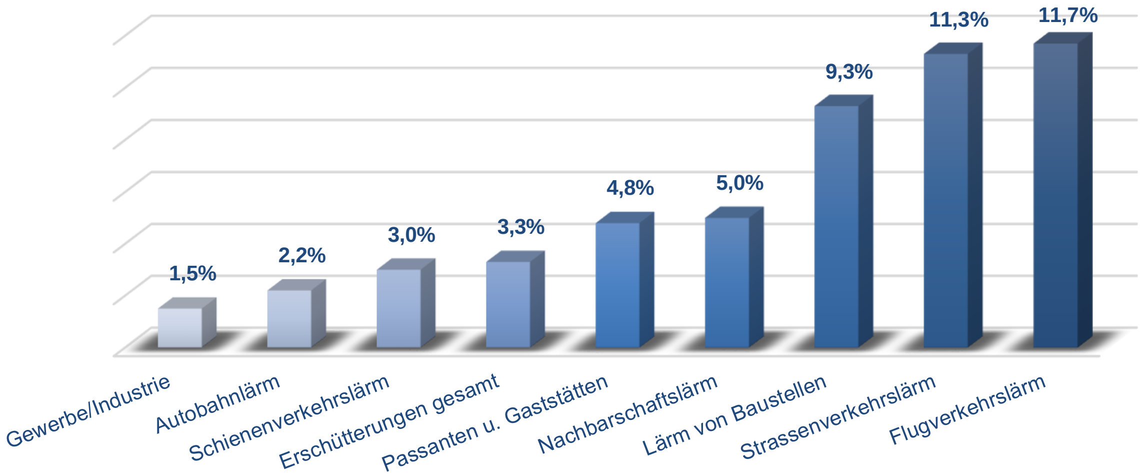

* Nach Belästigungsausmaß (Häufigkeit) aufsteigend sortiert

1.2. Belästigung und Verkehrslärmbelastung

Das Ausmaß der Belästigung durch Verkehrslärm zeigt einen deutlichen Zusammenhang zur gegebenen Lärmbelastung: Je höher die Verkehrslärmbelastung, desto höher die Belästigung. Allerdings unterscheiden sich die Belästigungsreaktionen in ihrer Ausprägung je nach Verkehrslärmquelle stark voneinander. Flugverkehrslärm wird in allen Belastungskategorien als störender eingestuft als der Straßenverkehrslärm und dieser wiederum als deutlich belästigender als der Schienenverkehrslärm:

Abbildung 140: Belästigung und Verkehrslärmbelastung nach Quelle 24h-Pegel (L_{den}) gruppiert im Mittelwertvergleich

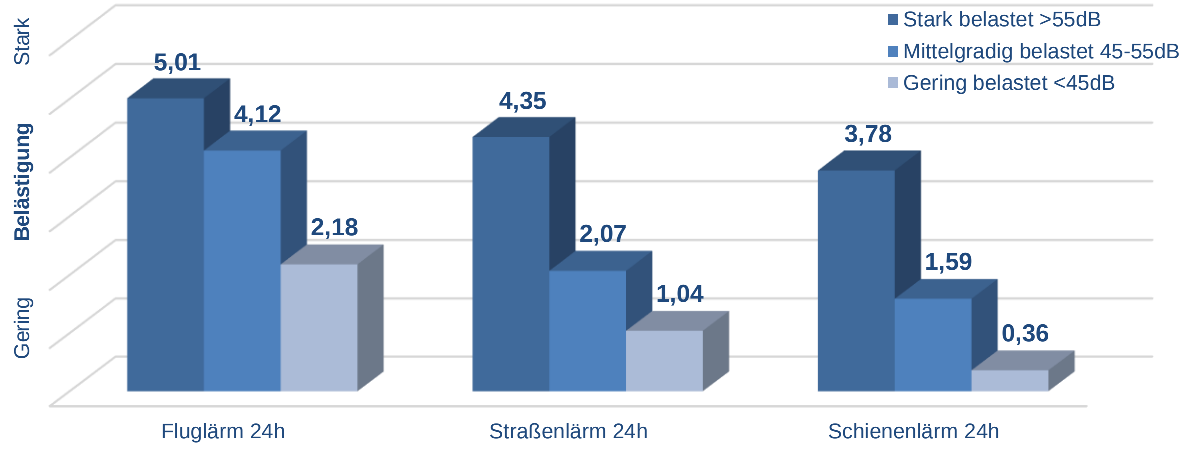

Wird das gemittelte Belästigungsausmaß nach vorhandener Lärmbelastung in 10dB-Schritten (24-Pegel L_{den}) nach Verkehrslärmquelle verglichen, wird deutlich, dass das Belästigungsausmaß beim Straßen- und Schienenverkehr erst bei Dauerschallpegeln über 50dB stärker zu steigen beginnt, bis zu diesen Pegeln jedoch von den Befragten relativ niedrig eingeschätzt wird. Ab diesen „Schwellenwerten" steigt das Belästigungsausmaß stark an und erreicht bei Dauerschallpegeln über 60dB ähnliche Ausprägungen für alle 3 Verkehrslärmquellen:[11]

Abbildung 141: Belästigung und Verkehrslärmbelastung nach Quelle 24h-Pegel (L_{den}) in 10dB-Schritten im Mittelwertvergleich

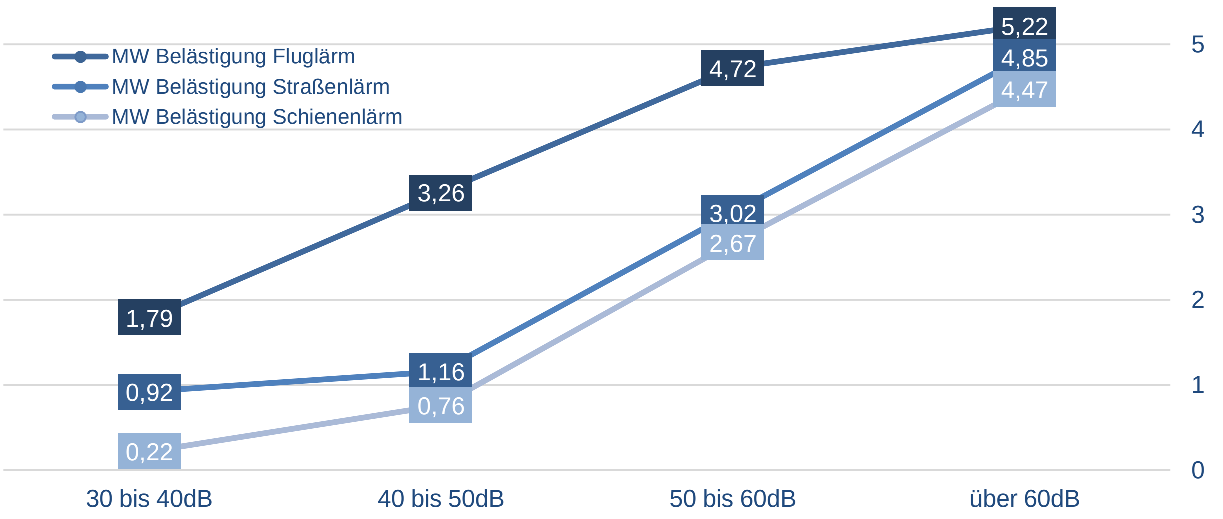

[11] Anm.: Die Darstellung zeigt nachvollziehbare Zusammenhänge auf, enthält jedoch keine Prüfungen auf Kovariablen und andere Prädiktoren und ist somit nicht den Dosis-Wirkungskurven aus der gängigen Fachliteratur gleichzusetzen.

Für den Flugverkehrslärm lässt sich diese Schwelle nicht festmachen. Das Belästigungsausmaß liegt bereits bei niedrigen Dauerlärmpegeln höher als beim Straßen- und Schienenverkehrslärm und steigt linearer an.

Die gegebenen Belastungen durch den Schienenverkehrslärm verursachen durchgehend eine geringere Belästigungsreaktion bei den befragten Innsbruckerinnen und Innsbruckern als der Straßen- und Flugverkehrslärm.

In der Kombination der Fragen zur Belästigung mit denjenigen zur empfundenen Ausgesetztheit gegenüber den einzelnen Verkehrslärmquellen, sowie der gegebenen Lärmbelastung zeigt sich die hohe Reliabilität der verwendeten Items und Skalen (siehe dazu auch die folgenden Kapitel zur spezifischen Flug-, Straßen- und Schienenverkehrslärmbelästigung).

1.3. Belästigung und Katastralgemeinden

1.3.1. Belästigung nach Quelle und Katastralgemeinden

Insgesamt am stärksten vom Lärm belästigt / gestört zeigen sich die befragten Innsbruckerinnen und Innsbrucker in Wilten, gefolgt von Innsbruck, Pradl und Hötting. In der Summe aller Lärmquellen am geringsten belästigt empfinden sich die Bewohnerinnen und Bewohner von Igls, Vill und Mühlau:

Abbildung 142: Belästigung nach Lärmquelle und Katastralgemeinde im Mittelwertvergleich

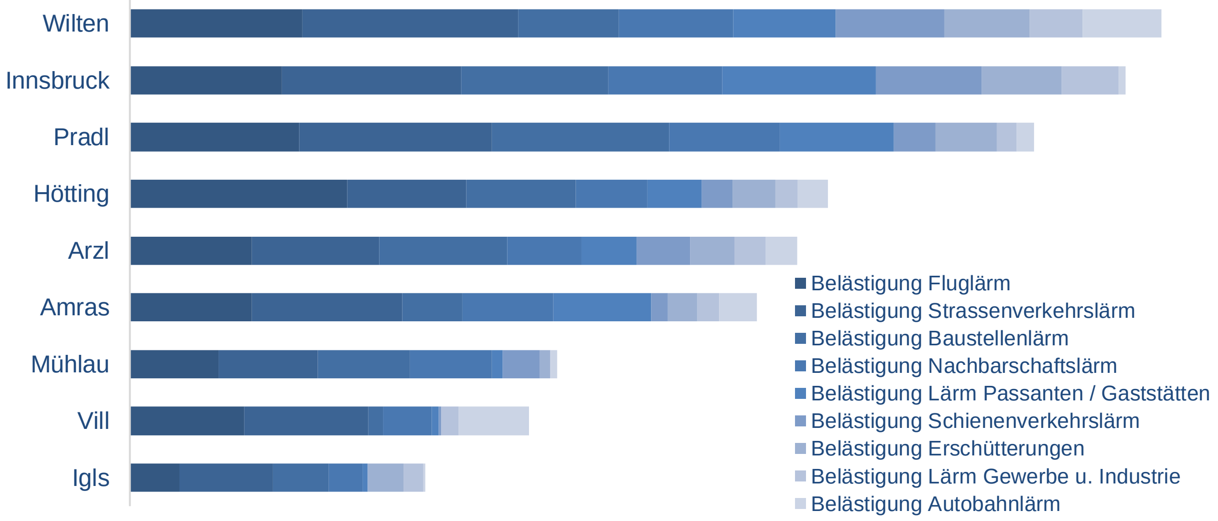

* Nach Belästigungsausmaß (Häufigkeit) absteigend sortiert

Tabelle 117: Belästigung nach Lärmquelle und Katastralgemeinde im Mittelwertvergleich

		Belästigung durch Lärm ausgehend von								
Katastralgemeinde		Flug	Straße	Baustellen	Nachbarschaft	Passanten / Gaststätten	Schiene	Erschütterungen	Gewerbe / Industrie	Autobahn
	MW	**2,38**	**2,95**	**1,18**	**1,78**	**1,92**	**,32**	**,58**	**,43**	**,73**
**Amras**	N	60	60	60	60	60	60	60	60	60
	SA	2,100	2,770	1,742	2,565	2,424	,792	1,576	1,332	1,561

	**MW**	**2,38**	**2,50**	**2,51**	**1,45**	**1,08**	**1,05**	**,86**	**,62**	**,61**
**Arzl**	N	102	102	102	102	102	102	102	102	102
	SA	2,413	2,695	2,824	2,146	2,047	2,140	1,872	1,516	1,684
	**MW**	**4,25**	**2,33**	**2,15**	**1,40**	**1,06**	**,61**	**,84**	**,44**	**,59**
**Hötting**	N	243	243	243	243	243	243	243	243	243
	SA	3,265	2,781	6,873	2,109	1,953	1,569	1,991	1,542	1,439
	**MW**	**,97**	**1,83**	**1,09**	**,66**	**,11**	**,00**	**,69**	**,40**	**,03**
**Igls**	N	35	35	35	35	35	35	35	35	35
	SA	1,272	1,723	1,853	1,282	,471	,000	1,231	1,063	,169
	**MW**	**2,98**	**3,50**	**2,88**	**2,24**	**3,01**	**2,06**	**1,57**	**1,11**	**,14**
**Innsbruck**	N	176	176	176	176	176	176	176	176	176
	SA	2,644	2,820	2,892	2,563	2,808	2,690	2,187	2,146	1,035
	**MW**	**1,74**	**1,94**	**1,80**	**1,60**	**,22**	**,72**	**,20**	**,02**	**,12**
**Mühlau**	N	50	50	50	50	50	50	50	50	50
	SA	1,925	2,280	2,499	2,185	,616	1,885	,782	,141	,435
	**MW**	**3,31**	**3,78**	**3,47**	**2,16**	**2,24**	**,81**	**1,20**	**,39**	**,34**
**Pradl**	N	188	188	188	188	188	188	188	188	188
	SA	2,861	3,159	3,682	2,664	2,752	1,805	2,454	1,158	1,275
	**MW**	**2,24**	**2,43**	**,29**	**,95**	**,14**	**,05**	**,00**	**,33**	**1,38**
**Vill**	N	21	21	21	21	21	21	21	21	21
	SA	1,758	2,181	,561	1,830	,478	,218	,000	,966	2,598
	**MW**	**3,38**	**4,22**	**1,97**	**2,24**	**2,01**	**2,13**	**1,66**	**1,04**	**1,54**
**Wilten**	N	156	156	156	156	156	156	156	156	156
	SA	2,899	2,815	2,828	2,838	2,561	2,673	2,725	2,186	2,769
	**MW**	**3,16**	**3,10**	**2,38**	**1,81**	**1,71**	**1,12**	**1,09**	**,63**	**,60**
**Gesamt**	N	1031	1031	1031	1031	1031	1031	1031	1031	1031
	SA	2,868	2,893	4,254	2,457	2,473	2,151	2,171	1,672	1,702

1.3.2. *Verkehrslärmbelästigung und Katastralgemeinden*

Abbildung 143: Belästigung nach Verkehrslärmquelle und Katastralgemeinde im Mittelwertvergleich

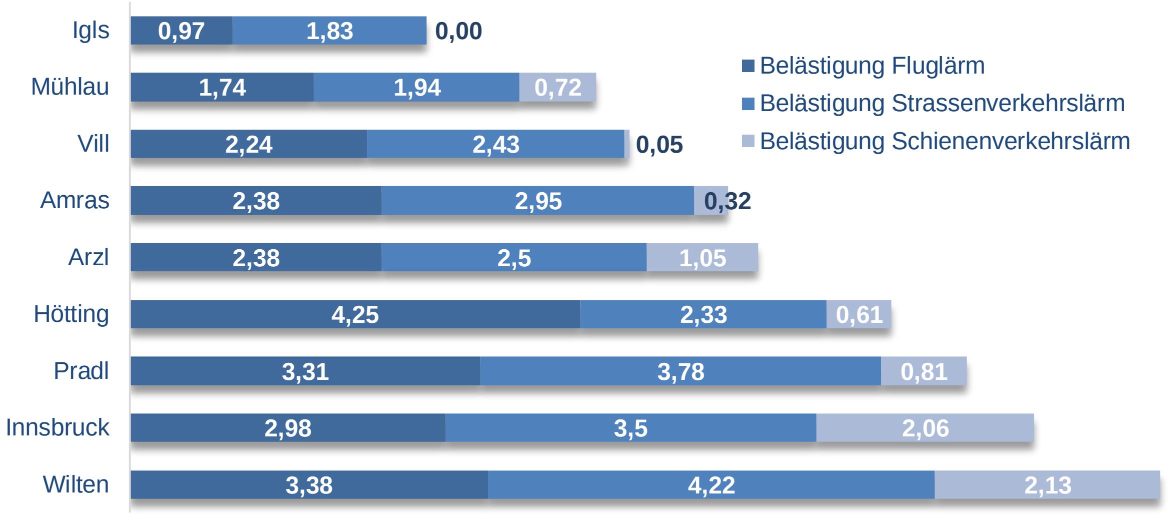

* Nach Belästigungsausmaß (Häufigkeit) aufsteigend sortiert

Die stärksten Lärmbelästigungen gehen vom Verkehrslärm aus, daher zeigt sich in der Betrachtung des Ausmaßes der reinen Verkehrslärmbelästigung ein beinahe identes Ranking der Katastralgemeinden. In Vill sind die Belästigungen durch Straßenverkehrslärm (Autobahn) etwas höher, weshalb Vill mit Mühlau die Plätze tauscht.

1.3.3. *Nicht verkehrsverursachte Belästigung und Katastralgemeinden*

Baustellenlärm, Erschütterungen, aber auch der Lärm aus Gewerbe und Industrie sind zum Teil ebenfalls verkehrsbedingt. Werden dennoch die Mittelwerte der nicht direkt verkehrsverursachten Lärmbelästigungsausmaße summiert dargestellt, ändert sich die Verteilung der Katastralgemeinden:

Abbildung 144: Belästigung nach nicht verkehrsverursachten Lärmquellen und Katastralgemeinde im Mittelwertvergleich

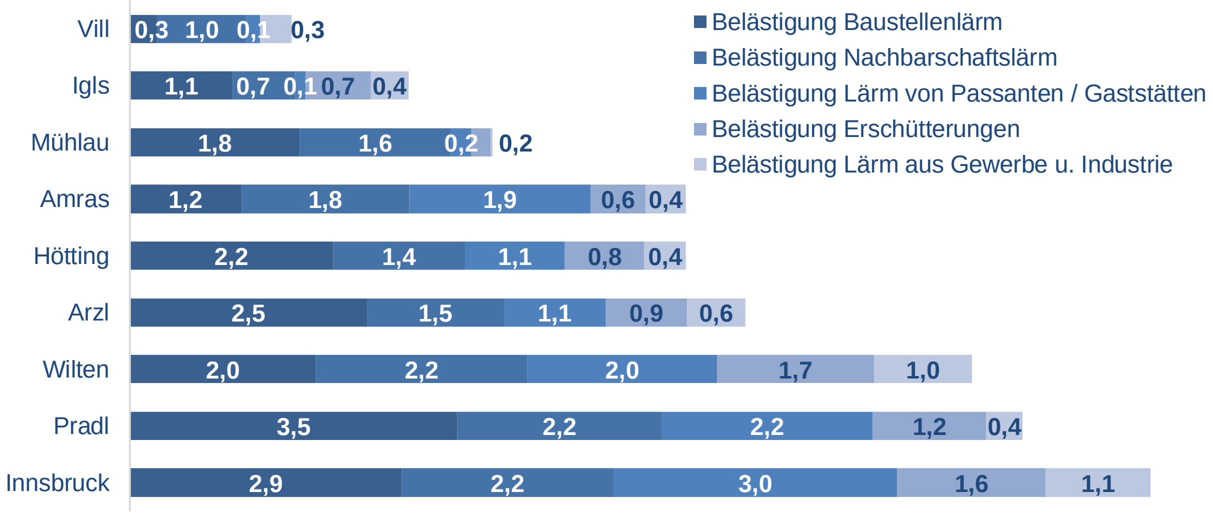

* Nach Belästigungsausmaß (Häufigkeit) aufsteigend sortiert

2. Belästigung durch Lärm insgesamt

Die Belästigung durch Lärm insgesamt wurde mittels einem eigenen Item zusätzlich abgefragt:

Abbildung 145: Belästigung durch Lärm insgesamt in Prozent

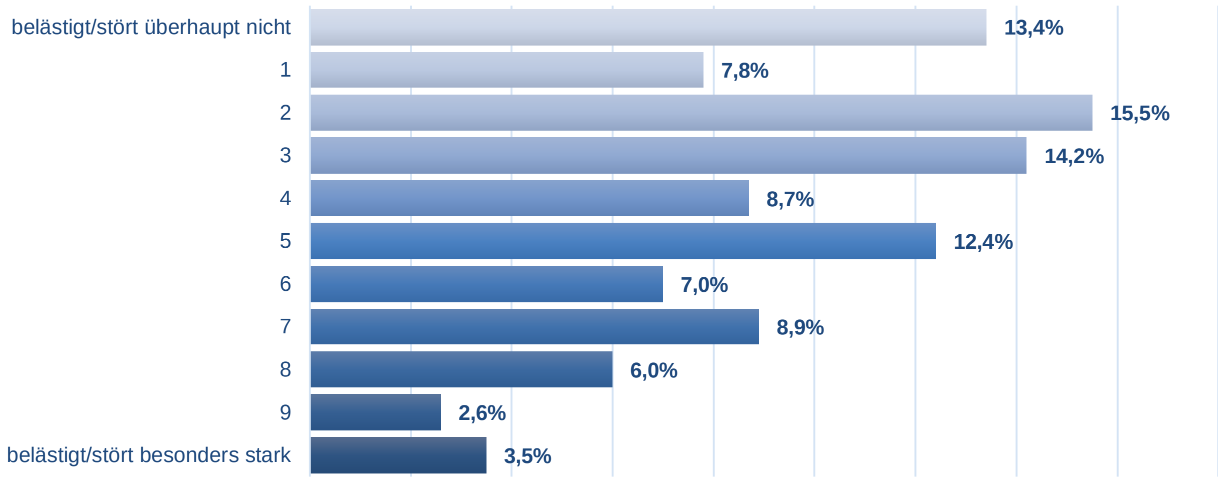

12,1% aller befragten Innsbruckerinnen und Innsbrucker schätzen sich selbst als vom Lärm insgesamt stark belästigt ein (*highly annoyed*: gruppierte Antwortkategorien 8, 9 und 10 [=*belästigt / stört besonders stark*]), etwas mehr als die Hälfte (51,2%) antworten in den mittleren Kategorien und 36,7% aller Probandinnen und Probanden sehen sich selbst als nur gering lärmbelästigt an (gruppierte Antwortkategorien 0 [=*belästigt / stört überhaupt nicht*], 1 und 2).

Tabelle 118: Belästigung durch Lärm insgesamt gruppiert

		Häufigkeit	Gültige Prozente	Kumulierte Prozente
Gültig	Gering belästigt	378	36,7	36,7
	Mittelgradig belästigt	528	51,2	87,9
	Stark belästigt	125	12,1	100,0
	Gesamt	1031	100,0	

Im Mittel liegen die Antworten der befragten Probandinnen und Probanden bei 3,89 (42,8%).

2.1. Belästigung durch Lärm insgesamt und Katastralgemeinden

Im Mittel die größte Störung durch Lärm insgesamt wird in Wilten und Innsbruck empfunden, dicht gefolgt von Pradl. Die geringste Belästigung durch Lärm insgesamt im eigenen Wohnumfeld empfinden die Befragten in Igls, Mühlau und Arzl:

Abbildung 146: Belästigung durch Lärm insgesamt und Katastralgemeinden im Mittelwertvergleich

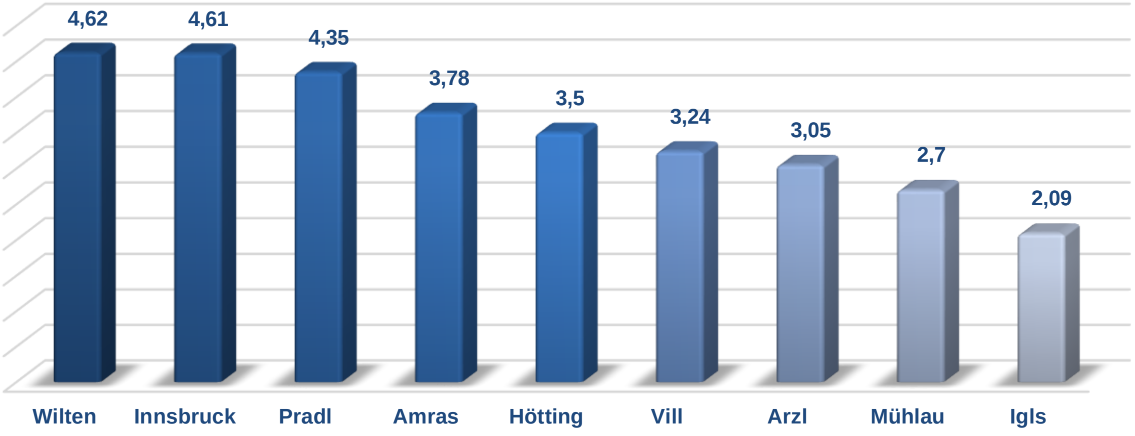

2.2. Belästigung durch Lärm insgesamt und ausgewählte Parameter

Frauen empfinden sich insgesamt stärker vom Lärm belästigt als Männer, ebenso die mittlere Altersgruppe der 41- bis 60-Jährigen im Vergleich zu den Jüngeren und Älteren.

Befragte, in deren Haushalt minderjährige Kinder leben, die zumeist bei geöffnetem oder gekippten Fenster schlafen, die in ihrem Wohnumfeld über eine lärmabgewandte Seite oder einen Innenhof verfügen, empfinden sich weniger vom Lärm gestört.

Der starke Bezug zum Verkehrslärm zeigt sich auch in Zusammenschau mit der Frage nach baulichem Schutz vor demselben:

Abbildung 147: Belästigung durch Lärm insgesamt und baulicher Schutz vor Verkehrslärm im Extremgruppenvergleich in Prozent

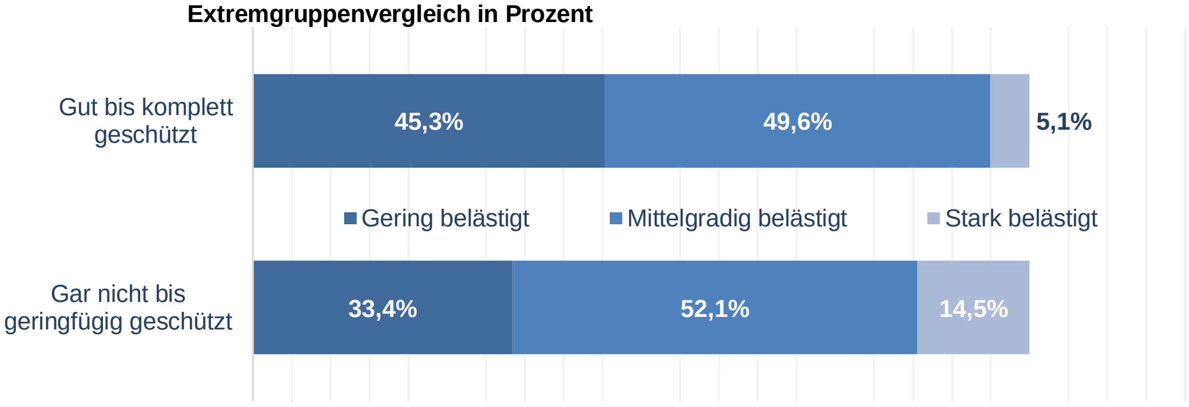

Deutlich zeigen sich auch Beziehungen zwischen dem Ausmaß der Belästigung durch Lärm insgesamt und der Einschätzung und Zufriedenheit mit den eigenen Lebens- und Wohnbedingungen, den Bedingungen für das Aufwachsen von Kindern, oder auch der Verbundenheit zur Stadt Innsbruck:

Je geringer das persönliche Ausmaß der Belästigung durch Lärm bewertet wird, desto höher werden die eigenen Lebens- und Wohnbedingungen eingeschätzt und vice versa:

Tabelle 119: Bedingungen für das Aufwachsen von Kindern und Belästigung durch Lärm insgesamt

Belästigung Lärm gesamt	Mittelwert	N	Standardabweichung	Standardfehler MW
belästigt/stört überhaupt nicht	**7,78**	136	2,152	,185
1	**7,40**	78	1,783	,202
2	**7,18**	158	2,044	,163
3	**6,85**	142	2,131	,179
4	**6,80**	86	2,034	,219
5	**6,67**	126	2,161	,193
6	**6,27**	71	2,104	,250
7	**6,28**	88	2,387	,255
8	**6,28**	61	2,511	,321
9	**6,59**	27	2,406	,463
belästigt/stört besonders stark	**5,14**	36	2,949	,491
Insgesamt	**6,85**	1009	2,246	,071

Abbildung 148: Zufriedenheit mit der eigenen Wohnsituation und Belästigung durch Lärm insgesamt im Mittelwertvergleich

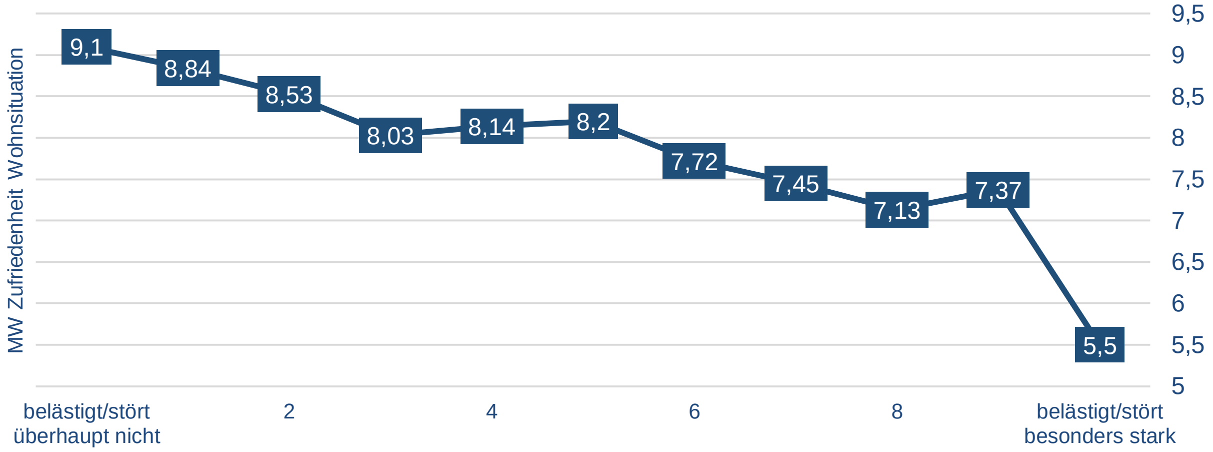

Dasselbe gilt auch für die Beziehungen zur Einschätzung der eigenen Lebensqualität und Lärmempfindlichkeit und einer ganzen Reihe anderer Parameter: Befragte Innsbruckerinnen und Innsbrucker, die sich stärker lärmbelästigt als andere fühlen,

- beurteilen das Lärmniveau in Innsbruck im Vergleich zu anderen österreichischen Städten schlechter,
- gehen häufiger davon aus, dass der Lärm insgesamt in den letzten Jahren zugenommen hat,
- schätzen die negativen Wirkungen von Lärm auf die Gesundheit stärker ein
- wenden häufiger Lärmadaptationsmechanismen an,
- sind wesentlich häufiger für (weitere) Maßnahmen zur Lärmreduktion,
- zeigen insgesamt in Bezug zu Beurteilungen der eigenen Wohn- und Lebensbedingungen eine deutlich negativere Grundhaltung.

Abbildung 149: Lärmempfindlichkeit und Belästigung durch Lärm insgesamt im Mittelwertvergleich

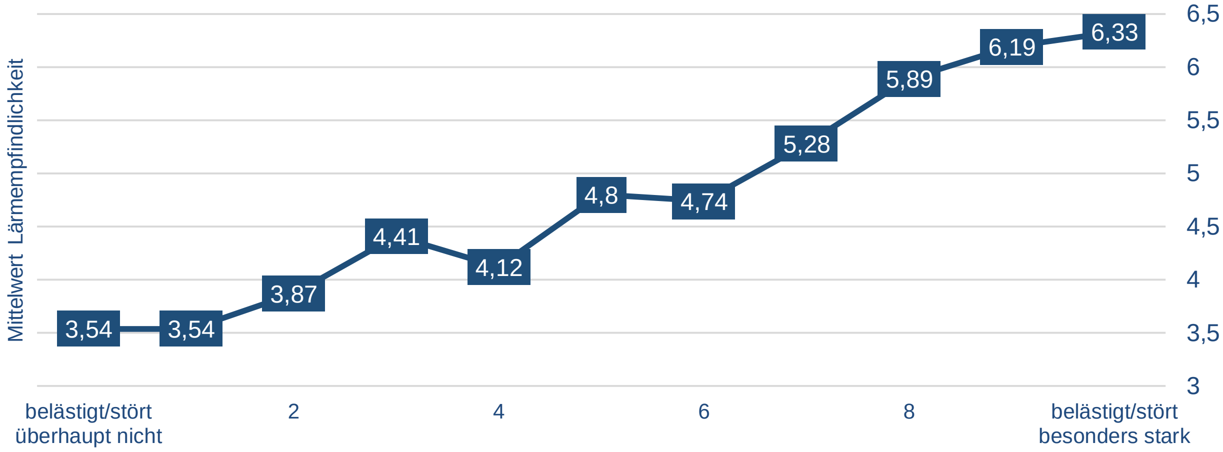

Tabelle 120: Belästigung durch Lärm insgesamt und ausgewählte Parameter (gruppiert)

		Belästigung Lärm gesamt gruppiert					
		Gering belästigt		Mittelgradig belästigt		Stark belästigt	
		Anzahl	Zeilen (%)	Anzahl	Zeilen (%)	Anzahl	Zeilen (%)
**Geschlecht**	weiblich	183	33,8%	287	53,0%	71	13,1%
	männlich	195	39,8%	241	49,2%	54	11,0%
**Altersgruppen**	18 bis 40 Jahre	159	36,1%	240	54,5%	41	9,3%
	41 bis 60 Jahre	117	37,0%	150	47,5%	49	15,5%
	über 60 Jahre	102	37,1%	138	50,2%	35	12,7%
**Bildungsschicht**	Primärer Schulabschluss	40	40,8%	43	43,9%	15	15,3%
	Sekundärer Schulabschluss	187	34,5%	295	54,4%	60	11,1%
	Tertiärer Schulabschluss	148	38,1%	190	49,0%	50	12,9%
**Haushalt mit/ohne minderjährige Kinder**	Haushalt ohne Kinder	264	34,6%	400	52,5%	98	12,9%
	Haushalt mit Kindern	114	42,4%	128	47,6%	27	10,0%
**Baulicher Schutz vor Verkehrslärm**	Keiner bis geringfügig	256	33,4%	399	52,1%	111	14,5%
	Gut bis komplett	107	45,3%	117	49,6%	12	5,1%
**Innenhof bzw. lärm-abgewandte Seite**	Ja	254	36,2%	372	53,1%	75	10,7%
	Nein	117	38,0%	143	46,4%	48	15,6%
**Nachtschlaf**	Fenster geöffnet/gekippt	329	40,1%	410	49,9%	82	10,0%
	Fenster geschlossen	45	22,3%	115	56,9%	42	20,8%
**Wohnbedingungen**	Nicht gut	30	35,7%	39	46,4%	15	17,9%
	Durchschnittlich	172	31,0%	307	55,3%	76	13,7%
	Sehr gut	175	44,9%	181	46,4%	34	8,7%
**Bedingungen für Kinder**	Nicht gut	9	19,1%	23	48,9%	15	31,9%
	Durchschnittlich	153	29,7%	291	56,5%	71	13,8%
	Sehr gut	210	47,0%	199	44,5%	38	8,5%
**Verbundenheit zu Innsbruck**	Nicht verbunden	13	23,6%	27	49,1%	15	27,3%
	Durchschnittlich	139	36,7%	201	53,0%	39	10,3%
	Sehr verbunden	225	37,8%	299	50,3%	71	11,9%

**Lebensbedingungen (Summenscore)**	Nicht gut (*)	1	20,0%	2	40,0%	2	40,0%
	Durchschnittlich	204	30,4%	373	55,7%	93	13,9%
	Sehr gut	173	48,6%	153	43,0%	30	8,4%
**Zufriedenheit eigene Wohnsituation**	Nicht zufrieden	2 (*)	7,1%	12	42,9%	14	50,0%
	Durchschnittlich	57	22,1%	153	59,3%	48	18,6%
	Sehr zufrieden	318	42,7%	363	48,8%	63	8,5%
**Lebensqualität**	Nicht gut (*)	1	10,0%	4	40,0%	5	50,0%
	Durchschnittlich	65	22,4%	176	60,7%	49	16,9%
	Sehr gut	308	42,5%	346	47,7%	71	9,8%
**Lärmempfindlichkeit**	Nicht lärmempfindlich	159	57,8%	103	37,5%	13	4,7%
	Durchschnittlich	168	28,8%	346	59,2%	70	12,0%
	Sehr lärmempfindlich	51	29,7%	79	45,9%	42	24,4%
**Summenscore Lärmcoping**	Coping ausgeprägt	20	8,3%	147	60,7%	75	31,0%
	Coping nicht ausgeprägt	358	45,4%	381	48,4%	49	6,2%
**Gefühlte Zunahme Lärmbelastung**	Ja	69	15,8%	272	62,4%	95	21,8%
	Nein	309	51,9%	256	43,0%	30	5,0%
**Gefühlte Abnahme Lärmbelastung**	Ja	32	55,2%	24	41,4%	2 (*)	3,4%
	Nein	346	35,6%	504	51,8%	123	12,6%
**Negative Gesund-heitswirkungen Lärm**	Gering	100	46,1%	102	47,0%	15	6,9%
	Stark	261	33,4%	411	52,6%	109	14,0%
**Lautheit Innsbruck im Vergleich zu anderen Städten Österreichs**	Ruhiger	129	44,2%	139	47,6%	24	8,2%
	Gleich	198	35,9%	291	52,8%	62	11,3%
	Lauter	42	24,1%	95	54,6%	37	21,3%
**Einschätzung eigener Lärmbeitrag**	Gering	296	38,2%	383	49,4%	96	12,4%
	Durchschnittlich	70	30,8%	134	59,0%	23	10,1%
	Groß	12	41,4%	11	37,9%	6	20,7%
**Notwendigkeit (weite-rer) Maßnahmen Lärm**	Maßnahmen notwendig	200	28,9%	383	55,4%	108	15,6%
	Keine Maßnahmen nötig	107	55,7%	73	38,0%	12	6,3%

(*) zu kleine Fallzahlen für solide Auswertungen

Befragte, die sich insgesamt stark lärmbelästigt fühlen (das gilt auch für die anderen Lärmbelästigungsquellen), schätzen ihren eigenen Beitrag zur Gesamtlärmsituation in Innsbruck höher ein, als sich weniger stark belästigt fühlende Befragte.

Eine gewisse Auffälligkeit stellen diejenigen Befragten[12] dar, die in der höchsten Antwortkategorie 10 (=*belästigt / stört besonders stark*) antworten. Diese Personen zeigen – auch in den anderen Belästigungsauswertungen unabhängig von der Lärmquelle und in Zusammenschau mit anderen Items – eine starke Tendenz zu negativeren Beurteilungen als die restlichen Befragten. – Die psychologische Disposition ist bei sämtlichen Auswertungen als wesentliche Komponente mitzudenken.

[12] Für das Item *Belästigung durch Lärm insgesamt* handelt es sich dabei um 36 befragte Personen.

3. Belästigung durch Flugverkehrslärm

Der Fluglärm löst im befragten Kollektiv knapp vor dem Straßenverkehrslärm die stärkste Belästigungsreaktion auS. 11,7% ordnen sich der Gruppe der Starkbelästigten zu, 39% antworten in den mittleren Belästigungskategorien und knapp die Hälfte aller befragten Innsbruckerinnen und Innsbrucker schätzen sich als gering vom Fluglärm belästigt ein.

Im Mittel liegen die Antworten der Befragten bei 3,16 (34,8%):

Abbildung 150: Belästigung durch Flugverkehrslärm in Prozent

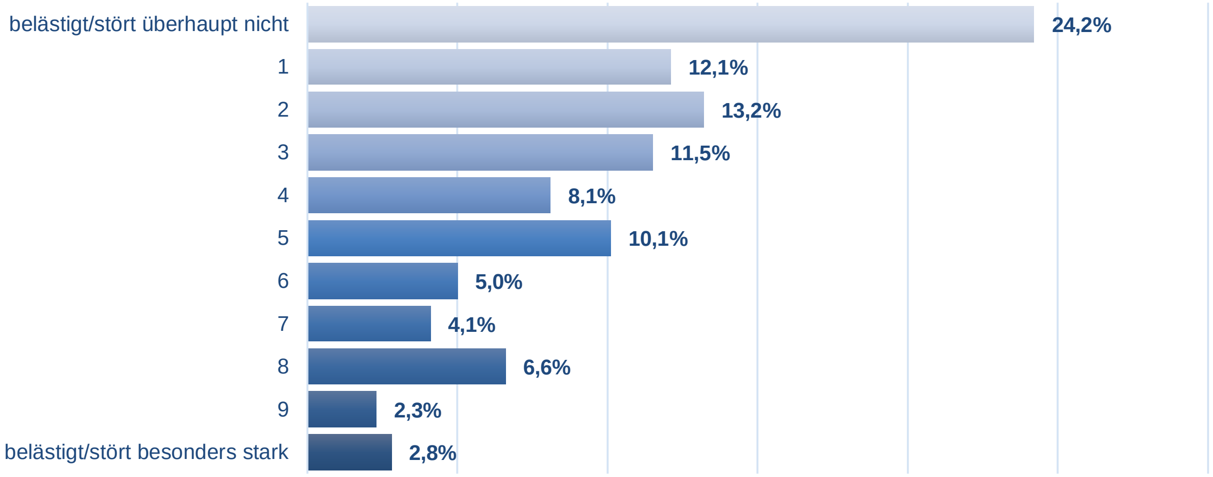

Tabelle 121: Belästigung durch Fluglärm gruppiert

		Häufigkeit	Gültige Prozente	Kumulierte Prozente
Gültig	Gering belästigt	510	49,5	49,5
	Mittelgradig belästigt	400	38,8	88,3
	Stark belästigt	121	11,7	100,0
	Gesamt	1031	100,0	

3.1. Fluglärmbelästigung, -Belastung und -Expositionsempfinden

Die Belästigung durch Flugverkehrslärm korrespondiert sehr ausgeprägt mit der tatsächlich gegebenen Fluglärmbelastung und zeigt mit steigender gegebener Belastung ein stark ansteigendes Belästigungsausmaß.

Das Expositionsempfinden gegenüber Fluglärm liegt dabei insgesamt höher als das Belästigungsempfinden und steigt besonders bei hohen vorhandenen Fluglärmbelastungen stärker und linearer an:

Abbildung 151: Fluglärmbelästigung, –Expositionsempfinden und 24h-Fluglärmbelastung L_{den} in 10dB-Schritten im Mittelwertvergleich

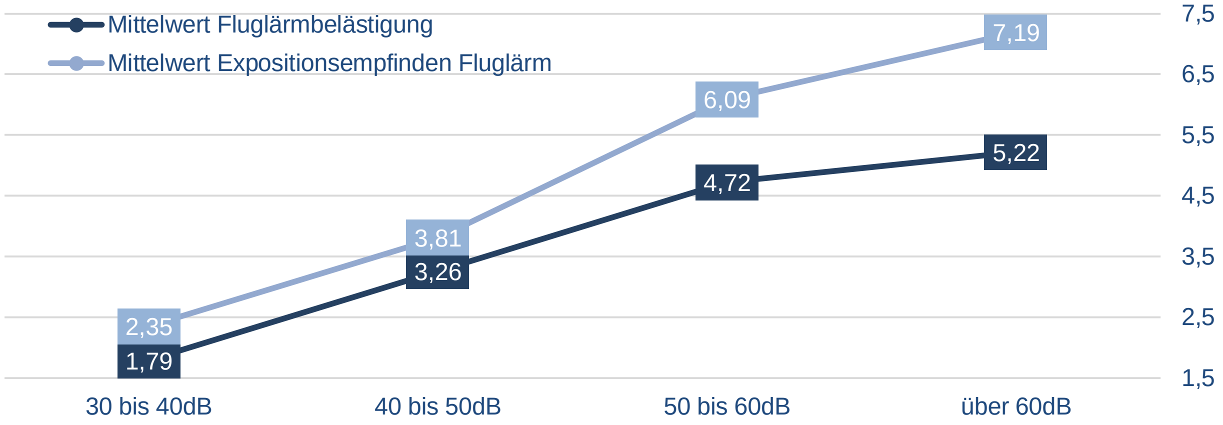

Anm.: Die Darstellung zeigt nachvollziehbare Zusammenhänge auf, enthält jedoch keine Prüfungen auf Kovariablen und andere Prädiktoren und ist somit nicht den Dosis-Wirkungskurven aus der gängigen Fachliteratur gleichzusetzen.

3.2. Fluglärmbelästigung und Katastralgemeinden

Entsprechend der gegebenen Fluglärmbelastung zeigt sich das mit Abstand stärkste Fluglärmbelästigungsempfinden in Hötting. Die geringste Störung durch Fluglärm im eigenen Wohnumfeld nehmen die Befragten in Igls wahr:

Abbildung 152: Fluglärmbelästigung und Katastralgemeinden im Mittelwertvergleich

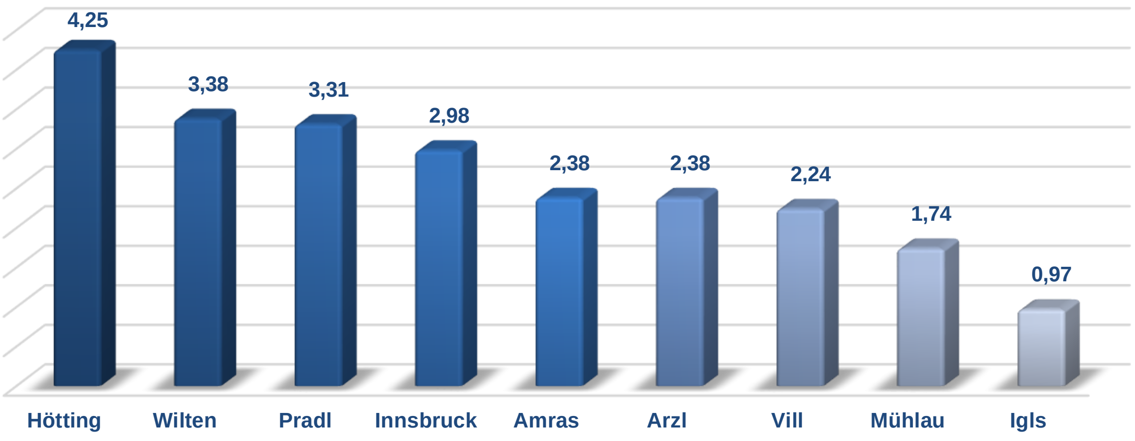

3.3. Fluglärmbelästigung und ausgewählte Parameter

Die Unterschiede zwischen einzelnen Subgruppen bezogen auf demographische Merkmale sind für das Ausmaß der Belästigung durch Fluglärm vergleichsweise gering ausgeprägt. Baulicher Lärmschutz, lärmabgewandte Seiten der Wohnungen oder Innenhöfe spielen für die Belästigung durch Fluglärm mit Ausnahme der flughafennahen Befragten ebenso keine größere Rolle.

Für die subjektiven Parameter der Einschätzung der eigenen Wohn- und Lebensbedingungen, sowie die verschiedenen Fragen zur Beurteilungen des Lärms in Innsbruck, zeigt sich ein sehr vergleichbares Bild zu den Bezügen zur Belästigung durch Lärm insgesamt:

Abbildung 153: Wohnbedingungen und Belästigung durch Fluglärm im Extremgruppenvergleich

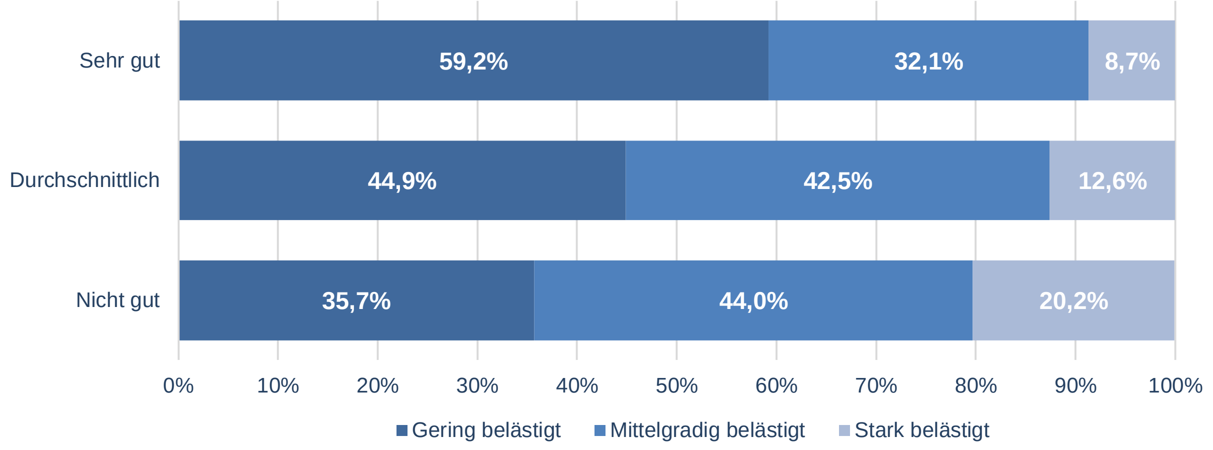

Eine starke Beziehung des Belästigungsausmaßes durch Flugverkehrslärm besteht wiederum zur Lärmempfindlichkeit:

Abbildung 154: Lärmempfindlichkeit und Belästigung durch Fluglärm im Mittelwertvergleich

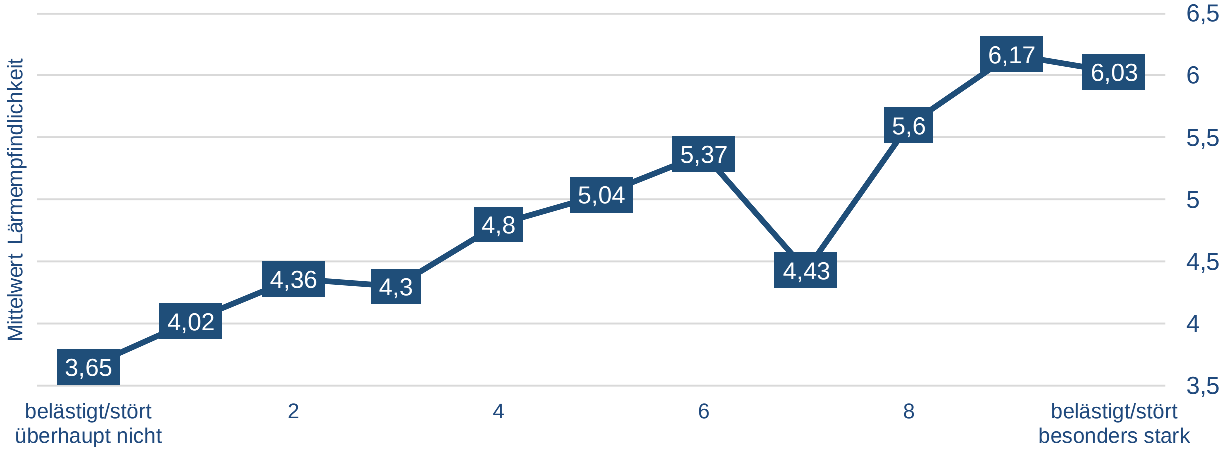

Tabelle 122: Belästigung durch Fluglärm und ausgewählte Parameter (gruppiert)

		Belästigung Fluglärm gruppiert					
		Gering belästigt		Mittelgradig belästigt		Stark belästigt	
		Anzahl	Zeilen (%)	Anzahl	Zeilen (%)	Anzahl	Zeilen (%)
**Geschlecht**	weiblich	261	48,2%	219	40,5%	61	11,3%
	männlich	249	50,8%	181	36,9%	60	12,2%
**Altersgruppen**	18 bis 40 Jahre	227	51,6%	170	38,6%	43	9,8%
	41 bis 60 Jahre	147	46,5%	132	41,8%	37	11,7%
	über 60 Jahre	136	49,5%	98	35,6%	41	14,9%
**Bildungsschicht**	Primärer Schulabschluss	54	55,1%	32	32,7%	12	12,2%
	Sekundärer Schulabschluss	267	49,3%	218	40,2%	57	10,5%

	Tertiärer Schulabschluss	188	48,5%	148	38,1%	52	13,4%
**Haushalt mit/ohne minderjährige Kinder**	Haushalt ohne Kinder	368	48,3%	300	39,4%	94	12,3%
	Haushalt mit Kindern	142	52,8%	100	37,2%	27	10,0%
**Baulicher Schutz vor Verkehrslärm**	Keiner bis geringfügig	376	49,1%	294	38,4%	96	12,5%
	Gut bis komplett	116	49,2%	96	40,7%	24	10,2%
**Innenhof bzw. lärm-abgewandte Seite**	Ja	355	50,6%	285	40,7%	61	8,7%
	Nein	145	47,1%	107	34,7%	56	18,2%
**Nachtschlaf**	Fenster geöffnet/gekippt	414	50,4%	315	38,4%	92	11,2%
	Fenster geschlossen	91	45,0%	84	41,6%	27	13,4%
**Wohnbedingungen**	Nicht gut	30	35,7%	37	44,0%	17	20,2%
	Durchschnittlich	249	44,9%	236	42,5%	70	12,6%
	Sehr gut	231	59,2%	125	32,1%	34	8,7%
**Bedingungen für Kinder**	Nicht gut	20	42,6%	19	40,4%	8	17,0%
	Durchschnittlich	227	44,1%	219	42,5%	69	13,4%
	Sehr gut	254	56,8%	155	34,7%	38	8,5%
**Verbundenheit zu Innsbruck**	Nicht verbunden	24	43,6%	20	36,4%	11	20,0%
	Durchschnittlich	179	47,2%	157	41,4%	43	11,3%
	Sehr verbunden	306	51,4%	222	37,3%	67	11,3%
**Zufriedenheit eigene Wohnsituation**	Nicht zufrieden	14	50,0%	8	28,6%	6	21,4%
	Durchschnittlich	104	40,3%	119	46,1%	35	13,6%
	Sehr zufrieden	391	52,6%	273	36,7%	80	10,8%
**Lebensbedingungen (Summenscore)**	Nicht gut (*)	2	40,0%	2	40,0%	1	20,0%
	Durchschnittlich	290	43,3%	285	42,5%	95	14,2%
	Sehr gut	218	61,2%	113	31,7%	25	7,0%
**Lebensqualität**	Nicht gut (*)	2	20,0%	4	40,0%	4	40,0%
	Durchschnittlich	131	45,2%	113	39,0%	46	15,9%
	Sehr gut	374	51,6%	281	38,8%	70	9,7%
**Lärmempfindlichkeit**	Nicht lärmempfindlich	179	65,1%	82	29,8%	14	5,1%
	Durchschnittlich	267	45,7%	245	42,0%	72	12,3%
	Sehr lärmempfindlich	64	37,2%	73	42,4%	35	20,3%
**Summenscore Lärmcoping**	Coping ausgeprägt	82	33,9%	98	40,5%	62	25,6%
	Coping nicht ausgeprägt	428	54,3%	301	38,2%	59	7,5%
**Gefühlte Zunahme Lärmbelastung**	Ja	162	37,2%	191	43,8%	83	19,0%
	Nein	348	58,5%	209	35,1%	38	6,4%
**Gefühlte Abnahme Lärmbelastung**	Ja	36	62,1%	20	34,5%	2 (*)	3,4%
	Nein	474	48,7%	380	39,1%	119	12,2%
**Notwendigkeit (weite-rer) Maßnahmen Lärm**	Maßnahmen notwendig	291	42,1%	294	42,5%	106	15,3%
	Keine Maßnahmen nötig	123	64,1%	60	31,3%	9	4,7%
**Negative Gesund-heitswirkungen Lärm**	Gering	126	58,1%	78	35,9%	13	6,0%
	Stark	364	46,6%	316	40,5%	101	12,9%
**Lautheit Innsbruck im Vergleich zu anderen Städten Österreichs**	Ruhiger	150	51,4%	114	39,0%	28	9,6%
	Gleich	285	51,7%	206	37,4%	60	10,9%
	Lauter	67	38,5%	74	42,5%	33	19,0%

**Einschätzung eigener Lärmbeitrag**	Gering	395	51,0%	292	37,7%	88	11,4%
	Durchschnittlich	100	44,1%	100	44,1%	27	11,9%
	Groß	15	51,7%	8	27,6%	6	20,7%

(*) zu kleine Fallzahlen für solide Auswertungen

4. Belästigung durch Straßenverkehrslärm

Die Belästigung durch Straßenverkehrslärm fällt in ihrem Ausmaß sehr ähnlich derjenigen auf Fluglärm aus und fällt in ihren Ausprägungen, auch in Belastungsgruppen kategorisiert, nur sehr geringfügig hinter den Flugverkehrslärm zurück. Die Anzahl an Personen, die mit der höchstmöglichen Kategorie 10 (=*belästigt / stört besonders stark*) antwortete, ist mit 3,5% sogar etwas höher als beim Fluglärm. Mehr als die Hälfte aller Befragten schätzt sich selbst als nur gering vom Straßenverkehrslärm gestört ein. Ähnlich wie beim Flugverkehrslärm fühlt sich jede(r) 9. Befragte stark vom Straßenverkehrslärm belästigt:

Abbildung 155: Belästigung durch Strassenverkehrslärm in Prozent

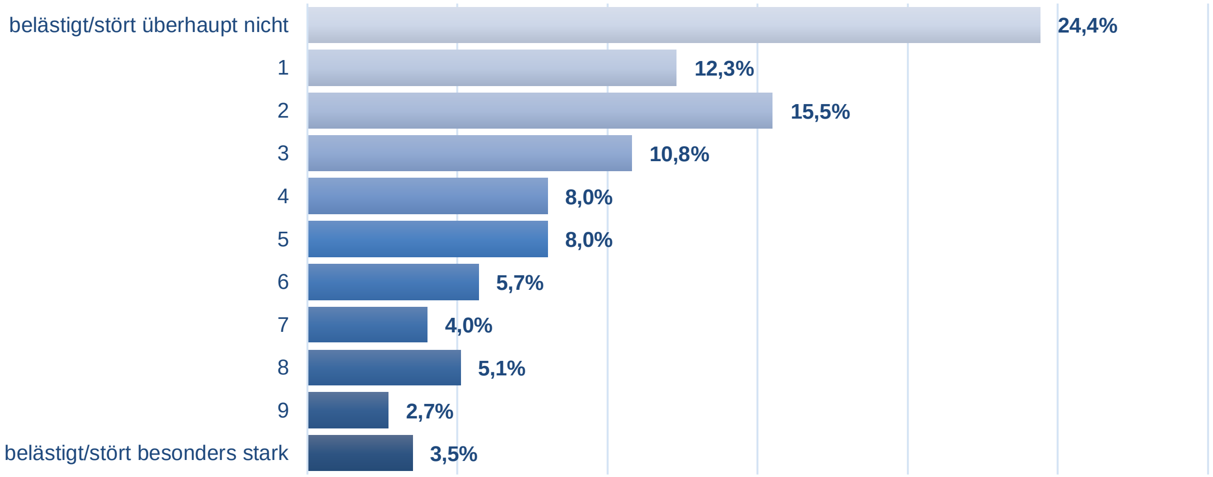

Im Mittel liegen die Antworten der befragten Innsbruckerinnen und Innsbrucker bei 3,1 (34,1%).

Tabelle 123: Belästigung durch Straßenverkehrslärm gruppiert

		Häufigkeit	Gültige Prozente	Kumulierte Prozente
Gültig	Gering belästigt	539	52,3	52,3
	Mittelgradig belästigt	375	36,4	88,7
	Stark belästigt	117	11,3	100,0
	Gesamt	1031	100,0	

4.1. Straßenlärmbelästigung, -Belastung und -Expositionsempfinden

Auch in der Zusammenschau der Belästigung durch Straßenverkehrslärm mit der gegebenen Straßenverkehrslärmbelastung zeigt sich ein deutlicher Zusammenhang. Wie schon beim Fluglärm liegt das Ausmaß der empfundenen Störung durch den Straßenverkehrslärm etwas unterhalb der empfundenen Ausgesetztheit.

Im Unterschied zur Belästigung durch Fluglärm steigt das Belästigungsausmaß in Bezug zum Straßenverkehrslärm erst bei höheren gegebenen Lärmpegeln über 50dB L_{den} stark an:

Abbildung 156: Straßenlärmbelästigung und –Expositionsempfinden und 24h-Straßenlärmbelastung L_{den} in 10dB-Schritten im Mittelwertvergleich

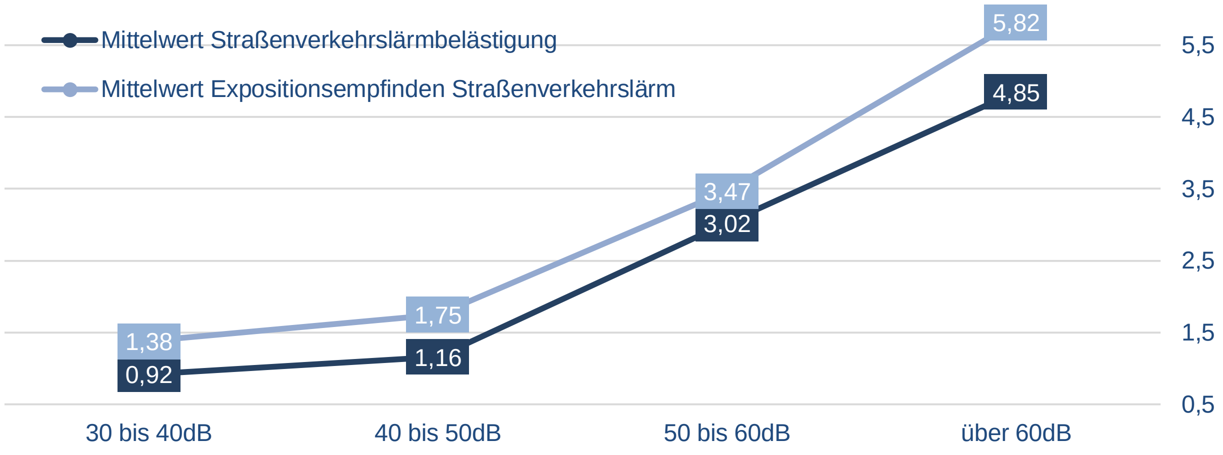

Anm.: Die Darstellung zeigt nachvollziehbare Zusammenhänge auf, enthält jedoch keine Prüfungen auf Kovariablen und andere Prädiktoren und ist somit nicht den Dosis-Wirkungskurven aus der gängigen Fachliteratur gleichzusetzen.

4.2. Straßenlärmbelästigung und Katastralgemeinden

Das größte Ausmaß an Belästigung durch Straßenverkehrslärm wird in Wilten empfunden, gefolgt von Pradl, Innsbruck und AmraS. Die geringste Störung durch Straßenverkehrslärm im eigenen Wohnumfeld empfinden die Befragten in Igls, Mühlau und Vill:

Abbildung 157: Straßenlärmbelästigung und Katastralgemeinden im Mittelwertvergleich

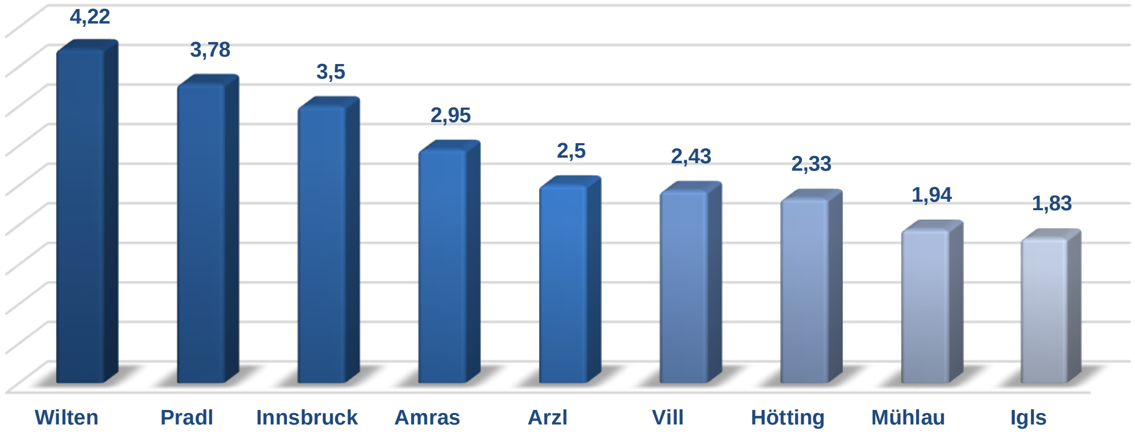

4.3. Straßenverkehrslärmbelästigung und ausgewählte Parameter

Ältere und befragte Innsbruckerinnen und Innsbrucker unter 40 Jahren empfinden sich weniger vom Straßenverkehrslärm belästigt als die mittlere Altersgruppe der 41- bis 60-Jährigen.

Frauen geben an, sich deutlich stärker vom Straßenverkehrslärm gestört zu fühlen als Männer:

Tabelle 124: Geschlecht und Belästigung durch Straßenverkehrslärm gruppiert

			Belästigung Straßenverkehrslärm			Gesamt
			Gering belästigt	Mittelgradig belästigt	Stark belästigt	
Geschlecht	weiblich	Anzahl	263	207	71	541
		Prozent	48,6%	38,3%	13,1%	100,0%
	männlich	Anzahl	276	168	46	490
		Prozent	56,3%	34,3%	9,4%	100,0%
Gesamt		Anzahl	539	375	117	1031
		Prozent	52,3%	36,4%	11,3%	100,0%

Durch bauliche Abschirmungen (Gebäude, Mauern etc.) vor Verkehrslärm geschützte Personen, sind deutlich weniger vom Straßenverkehr lärmbelästigt als ungeschützte:

Abbildung 158: Belästigung durch Straßenverkehrslärm und baulicher Schutz vor Verkehrslärm im Extremgruppenvergleich in Prozent

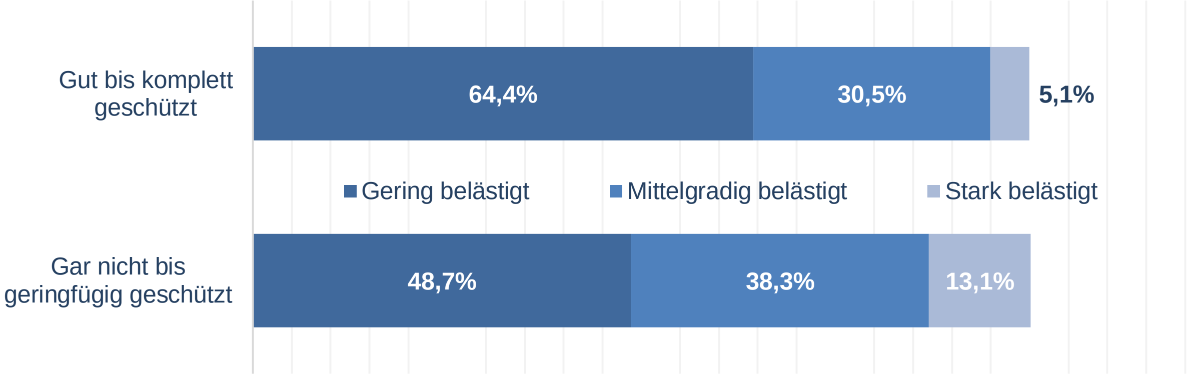

Die meisten der untersuchten Parameter unterscheiden sich nicht stark von Belästigungsauswertungen in Zusammenschau mit anderen Lärmquellen. So auch die Einschätzung der Güte der persönlichen Wohn- und Lebensbedingungen (in der folgenden Grafik als Summenscore dargestellt), welche bei zunehmender Straßenverkehrslärmbelästigung sinkt:

Abbildung 159: Lebensbedingungen (Summenscore) und Belästigung durch Straßenverkehrslärm im Mittelwertvergleich

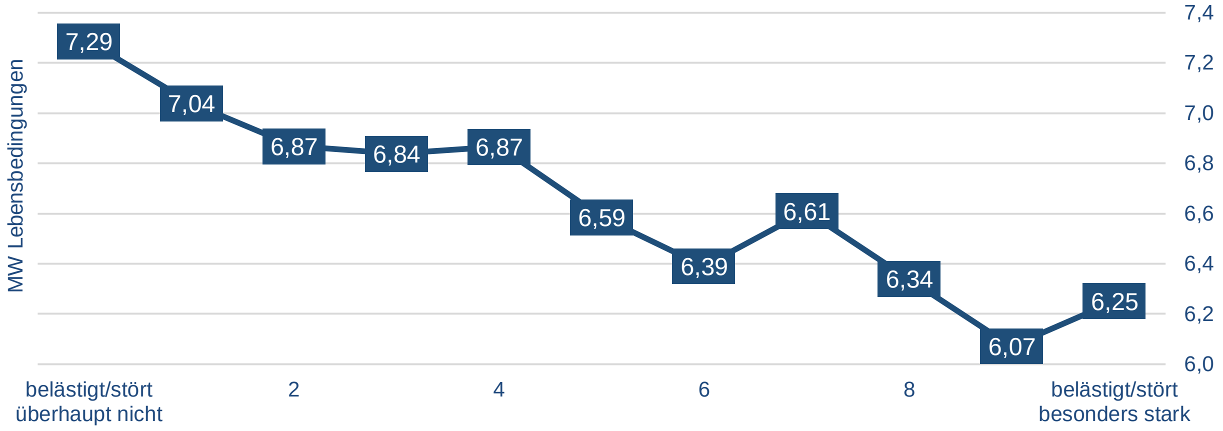

Tabelle 125: Belästigung durch Straßenverkehrslärm und ausgewählte Parameter (gruppiert)

		Belästigung Straßenverkehrslärm					
		Gering belästigt		Mittelgradig belästigt		Stark belästigt	
		Anzahl	Zeilen (%)	Anzahl	Zeilen (%)	Anzahl	Zeilen (%)
**Geschlecht**	weiblich	263	48,6%	207	38,3%	71	13,1%
	männlich	276	56,3%	168	34,3%	46	9,4%
**Altersgruppen**	18 bis 40 Jahre	237	53,9%	161	36,6%	42	9,5%
	41 bis 60 Jahre	164	51,9%	107	33,9%	45	14,2%
	über 60 Jahre	138	50,2%	107	38,9%	30	10,9%
**Bildungsschicht**	Primärer Schulabschluss	52	53,1%	33	33,7%	13	13,3%
	Sekundärer Schulabschluss	270	49,8%	211	38,9%	61	11,3%
	Tertiärer Schulabschluss	214	55,2%	131	33,8%	43	11,1%
**Haushalt mit/ohne minderjährige Kinder**	Haushalt ohne Kinder	393	51,6%	280	36,7%	89	11,7%
	Haushalt mit Kindern	146	54,3%	95	35,3%	28	10,4%
**Baulicher Schutz vor Verkehrslärm**	Keiner bis geringfügig	373	48,7%	293	38,3%	100	13,1%
	Gut bis komplett	152	64,4%	72	30,5%	12	5,1%
**Innenhof bzw. lärm-abgewandte Seite**	Ja	368	52,5%	265	37,8%	68	9,7%
	Nein	162	52,6%	99	32,1%	47	15,3%
**Nachtschlaf**	Fenster geöffnet/gekippt	462	56,3%	295	35,9%	64	7,8%
	Fenster geschlossen	74	36,6%	77	38,1%	51	25,2%
**Wohnbedingungen**	Nicht gut	38	45,2%	27	32,1%	19	22,6%
	Durchschnittlich	266	47,9%	218	39,3%	71	12,8%
	Sehr gut	234	60,0%	129	33,1%	27	6,9%
**Bedingungen für Kinder**	Nicht gut	15	31,9%	19	40,4%	13	27,7%
	Durchschnittlich	238	46,2%	201	39,0%	76	14,8%
	Sehr gut	273	61,1%	148	33,1%	26	5,8%
**Verbundenheit zu Innsbruck**	Nicht verbunden	21	38,2%	20	36,4%	14	25,5%
	Durchschnittlich	214	56,5%	130	34,3%	35	9,2%
	Sehr verbunden	303	50,9%	224	37,6%	68	11,4%

**Zufriedenheit eigene Wohnsituation**	Nicht zufrieden	13	46,4%	6	21,4%	9	32,1%
	Durchschnittlich	96	37,2%	111	43,0%	51	19,8%
	Sehr zufrieden	430	57,8%	257	34,5%	57	7,7%
**Lebensbedingungen (Summenscore)**	Nicht gut (*)	1	20,0%	3	60,0%	1	20,0%
	Durchschnittlich	316	47,2%	261	39,0%	93	13,9%
	Sehr gut	222	62,4%	111	31,2%	23	6,5%
**Lebensqualität**	Nicht gut (*)	5	50,0%	1	10,0%	4	40,0%
	Durchschnittlich	121	41,7%	125	43,1%	44	15,2%
	Sehr gut	408	56,3%	249	34,3%	68	9,4%
**Lärmempfindlichkeit**	Nicht lärmempfindlich	177	64,4%	85	30,9%	13	4,7%
	Durchschnittlich	274	46,9%	234	40,1%	76	13,0%
	Sehr lärmempfindlich	88	51,2%	56	32,6%	28	16,3%
**Summenscore Lärmcoping**	Coping ausgeprägt	73	30,2%	101	41,7%	68	28,1%
	Coping nicht ausgeprägt	466	59,1%	274	34,8%	48	6,1%
**Gefühlte Zunahme Lärmbelastung**	Ja	155	35,6%	197	45,2%	84	19,3%
	Nein	384	64,5%	178	29,9%	33	5,5%
**Gefühlte Abnahme Lärmbelastung**	Ja	37	63,8%	17	29,3%	4 (*)	6,9%
	Nein	502	51,6%	358	36,8%	113	11,6%
**Notwendigkeit (weiterer) Maßnahmen Lärm**	Maßnahmen notwendig	322	46,6%	268	38,8%	101	14,6%
	Keine Maßnahmen nötig	132	68,8%	50	26,0%	10	5,2%
**Negative Gesundheitswirkungen Lärm**	Gering	130	59,9%	70	32,3%	17	7,8%
	Stark	392	50,2%	291	37,3%	98	12,5%
**Lautheit Innsbruck im Vergleich zu anderen Städten Österreichs**	Ruhiger	187	64,0%	83	28,4%	22	7,5%
	Gleich	280	50,8%	208	37,7%	63	11,4%
	Lauter	63	36,2%	79	45,4%	32	18,4%
**Einschätzung eigener Lärmbeitrag**	Gering	411	53,0%	273	35,2%	91	11,7%
	Durchschnittlich	114	50,2%	95	41,9%	18	7,9%
	Groß	14	48,3%	7	24,1%	8	27,6%

(*) zu kleine Fallzahlen für solide Auswertungen

5. Belästigung durch Baustellenlärm

Wie schon bei der Einschätzung der Lärmexposition der eigenen Wohnung / des eigenen Hauses fällt die Belästigungsreaktion durch Baustellenlärm und die folgenden Belästigungsreaktionen deutlich anders aus, als Störungswahrnehmung durch Flug- und Straßenverkehrslärm. Fast die Hälfte aller befragten Innsbruckerinnen und Innsbrucker *stört* der Baustellenlärm in der Stadt *überhaupt nicht* und fast zwei Drittel (65%) aller Probandinnen und Probanden sehen sich selbst als nur gering vom Baustellenlärm belästigt an (gruppierte Antwortkategorien 0 [=*belästigt / stört überhaupt nicht*], 1 und 2). Über 9% sehen sich hingegen als stark belästigt (*highly annoyed*: gruppierte Antwortkategorien 8, 9 und 10 [=*belästigt / stört besonders stark*]) und rund ein Viertel der Befragten (25,6%) antwortet in den mittleren Kategorien. Eine Person macht zu dieser Frage keine Angaben:

Abbildung 160: Belästigung durch Baustellenlärm in Prozent

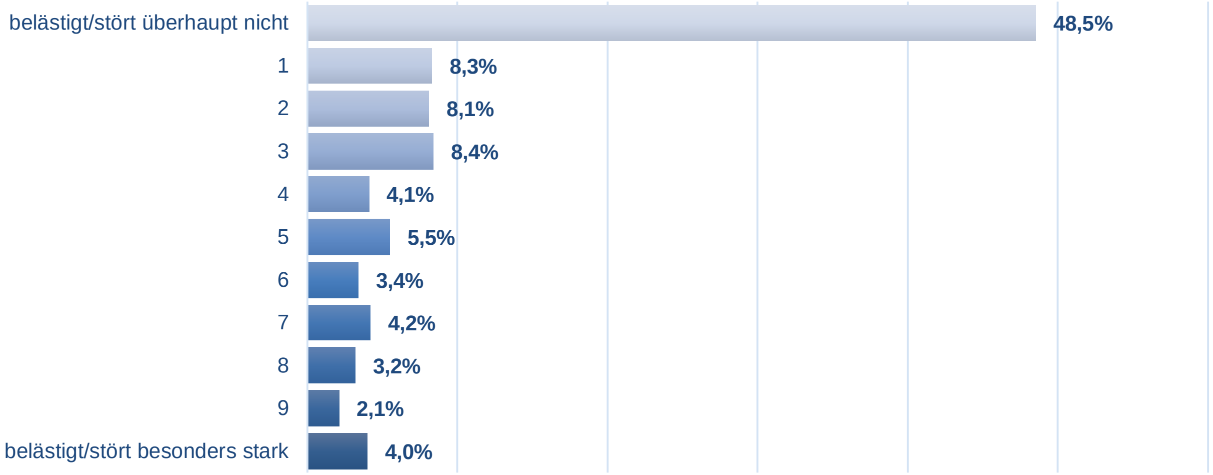

Im Mittel liegen die Antworten der befragten Probandinnen und Probanden bei 2,28 (25,1%) und damit im Mittelwert um knapp eine Ausprägung auf der 11-teiligen Antwortskala hinter der Belästigungsreaktion auf Flug- und Straßenverkehrslärm.

Tabelle 126: Belästigung durch Baustellenlärm gruppiert

		Häufigkeit	Gültige Prozente	Kumulierte Prozente
	Gering belästigt	670	65,0	65,0
	Mittelgradig belästigt	264	25,6	90,6
Gültig	Stark belästigt	96	9,3	99,9
	w.n. / k.A.	1	,1	100,0
	Gesamt	1031	100,0	

5.1. Baulärmbelästigung und Katastralgemeinden

Die Befragten in Pradl geben gemittelt das größte Ausmaß an Belästigung durch Baustellenlärm an, die befragten Innsbruckerinnen und Innsbrucker in Vill das mit Abstand niedrigste:

Abbildung 161: Belästigung durch Baustellenlärm und Katastralgemeinden im Mittelwertvergleich

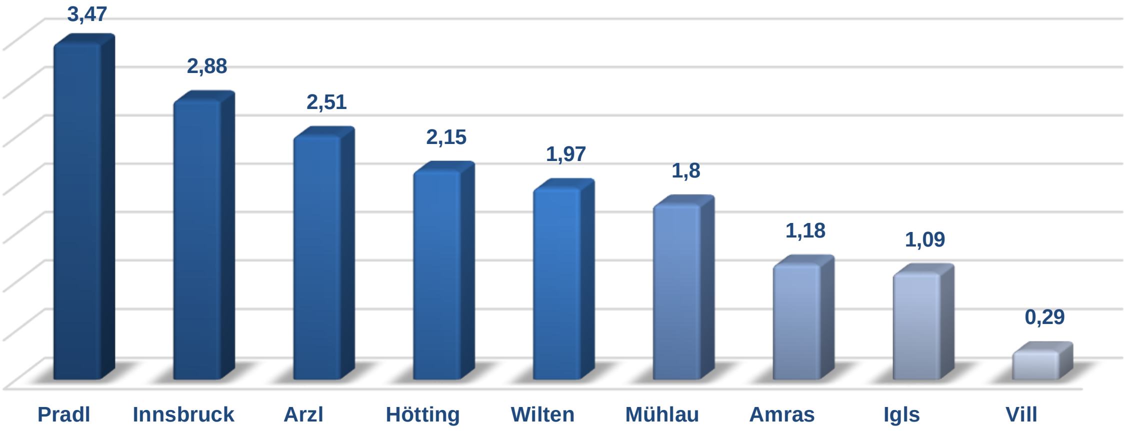

Anmerkung: Baustellenlärm ist stark auf Akutbelastungen bezogen und daher ist ein Vergleich der Katastralgemeinden zu einem sehr erheblichen Anteil nur eine Momentaufnahme.

5.2. Baulärmbelästigung und ausgewählte Parameter

Die Belästigung ausgehend von Baustellenlärm ist zum einen häufig ein zeitlich begrenztes Ärgernis und zum anderen zumindest teilweise unabhängig von anderen Wohn- und Lebensbedingungen. Das spiegelt sich auch in den Beantwortungen der einzelnen Befragten wieder. Die Unterschiede zwischen einzelnen Subgruppen fallen zum Teil vergleichsweise kleiner aus und streuen stärker.

Im Folgenden einige ausgewählte Parameter, jeweils in Extremgruppen zusammengefasst, in Zusammenschau mit der Belästigung durch Baulärm:

Tabelle 127: Belästigung durch Baustellenlärm und ausgewählte Parameter (gruppiert)

		Belästigung Baustellenlärm gruppiert					
		Gering belästigt		Mittelgradig belästigt		Stark belästigt	
		Anzahl	Zeilen (%)	Anzahl	Zeilen (%)	Anzahl	Zeilen (%)
**Geschlecht**	weiblich	330	61,0%	150	27,7%	61	11,3%
	männlich	340	69,5%	114	23,3%	35	7,2%
**Altersgruppen**	18 bis 40 Jahre	275	62,6%	125	28,5%	39	8,9%
	41 bis 60 Jahre	200	63,3%	84	26,6%	32	10,1%
	über 60 Jahre	195	70,9%	55	20,0%	25	9,1%
**Haushalt mit/ohne minderjährige Kinder**	Haushalt ohne Kinder	501	65,8%	190	25,0%	70	9,2%
	Haushalt mit Kindern	169	62,8%	74	27,5%	26	9,7%
**Baulicher Schutz vor Verkehrslärm**	Keiner bis geringfügig	491	64,2%	195	25,5%	79	10,3%
	Gut bis komplett	158	66,9%	64	27,1%	14	5,9%
**Innenhof bzw. lärm-abgewandte Seite**	Ja	454	64,8%	188	26,8%	59	8,4%
	Nein	201	65,5%	70	22,8%	36	11,7%
**Nachtschlaf**	Fenster geöffnet/gekippt	542	66,1%	207	25,2%	71	8,7%
	Fenster geschlossen	125	61,9%	53	26,2%	24	11,9%
**Wohnbedingungen**	Nicht gut	50	59,5%	21	25,0%	13	15,5%
	Durchschnittlich	349	62,9%	151	27,2%	55	9,9%
	Sehr gut	270	69,4%	91	23,4%	28	7,2%
**Bedingungen für Kinder**	Nicht gut	21	44,7%	15	31,9%	11	23,4%
	Durchschnittlich	317	61,7%	142	27,6%	55	10,7%
	Sehr gut	315	70,5%	102	22,8%	30	6,7%
**Verbundenheit zu Innsbruck**	Nicht verbunden	29	52,7%	16	29,1%	10	18,2%
	Durchschnittlich	236	62,4%	111	29,4%	31	8,2%
	Sehr verbunden	403	67,7%	137	23,0%	55	9,2%
**Zufriedenheit eigene Wohnsituation**	Nicht zufrieden	18	64,3%	7	25,0%	3 (*)	10,7%
	Durchschnittlich	137	53,1%	81	31,4%	40	15,5%
	Sehr zufrieden	514	69,2%	176	23,7%	53	7,1%

**Lebensbedingungen (Summenscore)**	Nicht gut (*)	3	60,0%	1	20,0%	1	20,0%
	Durchschnittlich	418	62,5%	180	26,9%	71	10,6%
	Sehr gut	249	69,9%	83	23,3%	24	6,7%
**Lebensqualität**	Nicht gut (*)	5	50,0%	1	10,0%	4	40,0%
	Durchschnittlich	168	57,9%	90	31,0%	32	11,0%
	Sehr gut	492	67,9%	173	23,9%	60	8,3%
**Lärmempfindlichkeit**	Nicht lärmempfindlich	200	72,7%	59	21,5%	16	5,8%
	Durchschnittlich	369	63,2%	156	26,7%	59	10,1%
	Sehr lärmempfindlich	101	59,1%	49	28,7%	21	12,3%
**Summenscore Lärmcoping**	Coping ausgeprägt	112	46,3%	87	36,0%	43	17,8%
	Coping nicht ausgeprägt	558	70,9%	177	22,5%	52	6,6%
**Gefühlte Zunahme Lärmbelastung**	Ja	212	48,6%	148	33,9%	76	17,4%
	Nein	458	77,1%	116	19,5%	20	3,4%
**Gefühlte Abnahme Lärmbelastung**	Ja	40	69,0%	16	27,6%	2	3,4%
	Nein	630	64,8%	248	25,5%	94	9,7%
**Notwendigkeit (weitere) Maßnahmen Lärm**	Maßnahmen notwendig	435	63,0%	179	25,9%	76	11,0%
	Keine Maßnahmen nötig	129	67,2%	47	24,5%	16	8,3%
**Negative Gesundheitswirkungen Lärm**	Gering	160	73,7%	42	19,4%	15	6,9%
	Stark	490	62,8%	214	27,4%	76	9,7%
**Lautheit Innsbruck im Vergleich zu anderen Städten Österreichs**	Ruhiger	199	68,2%	69	23,6%	24	8,2%
	Gleich	362	65,7%	138	25,0%	51	9,3%
	Lauter	100	57,5%	54	31,0%	20	11,5%

(*) zu kleine Fallzahlen für solide Auswertungen

6. Belästigung durch Nachbarschaftslärm

Über 70% aller Befragten fühlen sich durch Nachbarschaftslärm gar nicht oder nur geringfügig gestört, 5% hingegen stark belästigt:

Abbildung 162: Belästigung durch Nachbarschaftslärm in Prozent

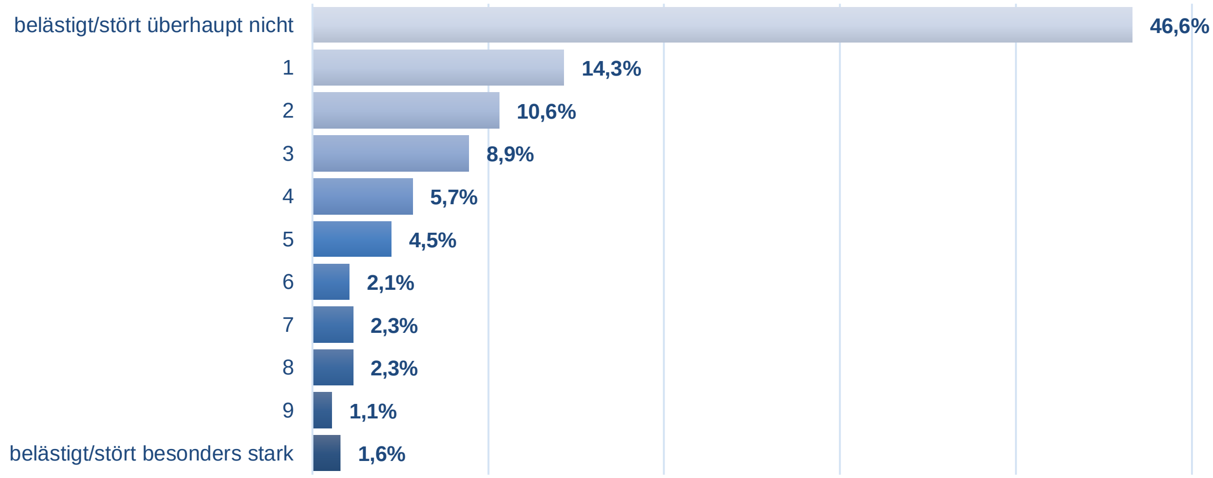

Im Mittel liegen die Antworten der befragten Probandinnen und Probanden bei 1,81 (19,9%):

Tabelle 128: Belästigung durch Nachbarschaftslärm gruppiert

		Häufigkeit	Gültige Prozente	Kumulierte Prozente
Gültig	Gering belästigt	736	71,4	71,4
	Mittelgradig belästigt	243	23,6	95,0
	Stark belästigt	52	5,0	100,0
	Gesamt	1031	100,0	

6.1. Nachbarschaftslärmbelästigung und Katastralgemeinden

In Innsbruck, Wilten und Pradl fühlen sich die Befragten am stärksten vom Nachbarschaftslärm belästigt, in Igls, Vill und Mühlau am wenigsten:

Abbildung 163: Nachbarschaftslärmbelästigung und Katastralgemeinden im Mittelwertvergleich

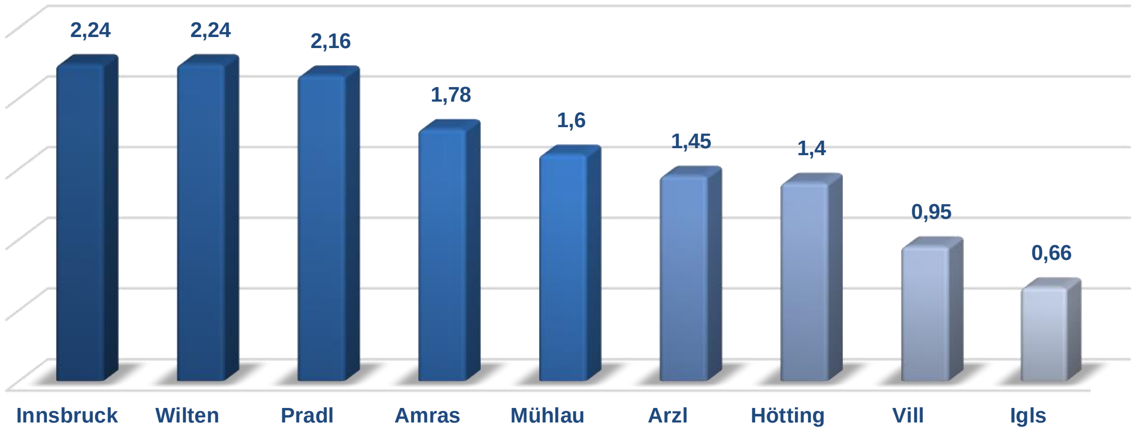

6.2. Nachbarschaftslärmbelästigung und ausgewählte Parameter

Wie schon bei den anderen Belästigungsauswertungen fühlt sich die mittlere Altersgruppe der 41 bis 60-Jährigen auch vom Nachbarschaftslärm am stärksten gestört. Zwischen den Geschlechtern gibt es hingegen kaum Unterschiede in der Belästigungsreaktion.

Aus den Auswertungen ist ersichtlich, dass Nachbarschaftslärm und die daraus resultierende Belästigung, sofern diese als stark wahrgenommen wird, sich ganz erheblich auf eine ganze Reihe von Einschätzungen zu den persönlichen Wohn- und Lebensbedingungen auswirkt. Diese werden von den stärker vom Nachbarschaftslärm belästigten befragten Innsbruckerinnen und Innsbruckern deutlich schlechter eingestuft, als von sich nur geringfügig lärmgestört Empfindenden.

Gleiches gilt, jeweils wechselseitig, auch für die Einschätzung der eigenen Lärmempfindlichkeit und der eigenen Lebensqualität.

Abbildung 164: Zufriedenheit mit der eigenen Wohnsituation und Belästigung durch Nachbarschaftslärm im Mittelwertvergleich

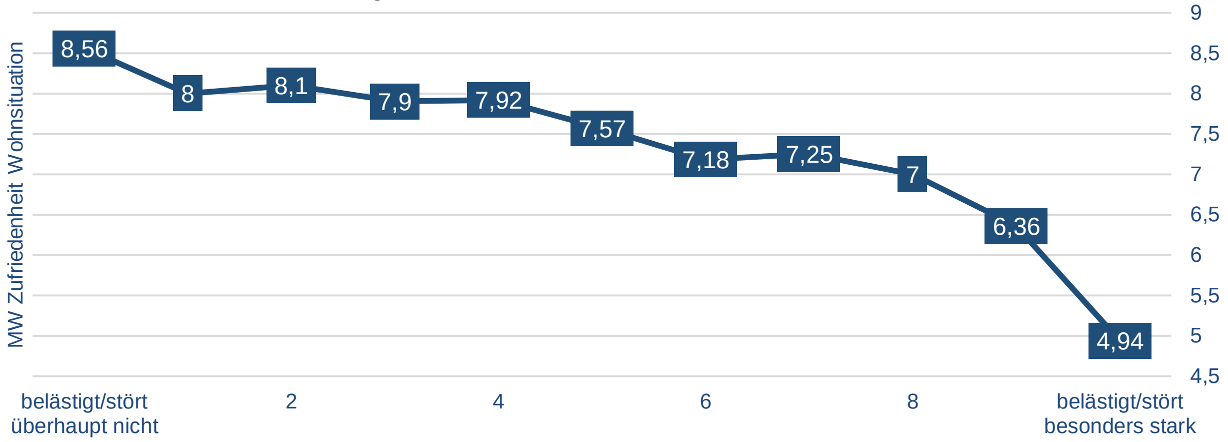

Tabelle 129: Belästigung durch Nachbarschaftslärm und ausgewählte Parameter (gruppiert)

		Belästigung Nachbarschaftslärm gruppiert					
		Gering belästigt		Mittelgradig belästigt		Stark belästigt	
		Anzahl	Zeilen (%)	Anzahl	Zeilen (%)	Anzahl	Zeilen (%)
**Geschlecht**	weiblich	388	71,7%	123	22,7%	30	5,5%
	männlich	348	71,0%	120	24,5%	22	4,5%
**Altersgruppen**	18 bis 40 Jahre	312	70,9%	111	25,2%	17	3,9%
	41 bis 60 Jahre	210	66,5%	79	25,0%	27	8,5%
	über 60 Jahre	214	77,8%	53	19,3%	8	2,9%
**Bildungsschicht**	Primärer Schulabschluss	71	72,4%	20	20,4%	7	7,1%
	Sekundärer Schulabschluss	388	71,6%	130	24,0%	24	4,4%
	Tertiärer Schulabschluss	274	70,6%	93	24,0%	21	5,4%
**Haushalt mit/ohne minderjährige Kinder**	Haushalt ohne Kinder	533	69,9%	187	24,5%	42	5,5%
	Haushalt mit Kindern	203	75,5%	56	20,8%	10	3,7%
**Nachtschlaf**	Fenster geöffnet/gekippt	599	73,0%	189	23,0%	33	4,0%
	Fenster geschlossen	131	64,9%	52	25,7%	19	9,4%
**Wohnbedingungen**	Nicht gut	52	61,9%	20	23,8%	12	14,3%
	Durchschnittlich	380	68,5%	150	27,0%	25	4,5%
	Sehr gut	302	77,4%	73	18,7%	15	3,8%
**Bedingungen für Kinder**	Nicht gut	22	46,8%	16	34,0%	9	19,1%
	Durchschnittlich	351	68,2%	138	26,8%	26	5,0%
	Sehr gut	345	77,2%	85	19,0%	17	3,8%
**Verbundenheit zu Innsbruck**	Nicht verbunden	33	60,0%	13	23,6%	9	16,4%
	Durchschnittlich	268	70,7%	97	25,6%	14	3,7%
	Sehr verbunden	434	72,9%	132	22,2%	29	4,9%
**Zufriedenheit eigene Wohnsituation**	Nicht zufrieden	13	46,4%	6	21,4%	9	32,1%
	Durchschnittlich	153	59,3%	87	33,7%	18	7,0%
	Sehr zufrieden	569	76,5%	150	20,2%	25	3,4%
**Lebensbedingungen**	Nicht gut (*)	3	60,0%	1	20,0%	1	20,0%

**(Summenscore)**	Durchschnittlich	443	66,1%	186	27,8%	41	6,1%
	Sehr gut	290	81,5%	56	15,7%	10	2,8%
	Nicht gut (*)	5	50,0%	2	20,0%	3	30,0%
**Lebensqualität**	Durchschnittlich	178	61,4%	87	30,0%	25	8,6%
	Sehr gut	549	75,7%	152	21,0%	24	3,3%
	Nicht lärmempfindlich	220	80,0%	46	16,7%	9	3,3%
**Lärmempfindlichkeit**	Durchschnittlich	409	70,0%	146	25,0%	29	5,0%
	Sehr lärmempfindlich	107	62,2%	51	29,7%	14	8,1%
**Summenscore**	Coping ausgeprägt	125	51,7%	83	34,3%	34	14,0%
**Lärmcoping**	Coping nicht ausgeprägt	611	77,5%	160	20,3%	17	2,2%
**Notwendigkeit (weite-**	Maßnahmen notwendig	467	67,6%	181	26,2%	43	6,2%
**rer) Maßnahmen Lärm**	Keine Maßnahmen nötig	152	79,2%	36	18,8%	4	2,1%
**Negative Gesund-**	Gering	171	78,8%	43	19,8%	3 (*)	1,4%
**heitswirkungen Lärm**	Stark	545	69,8%	188	24,1%	48	6,1%
**Lautheit Innsbruck im**	Ruhiger	207	70,9%	71	24,3%	14	4,8%
**Vergleich zu anderen**	Gleich	404	73,3%	124	22,5%	23	4,2%
**Städten Österreichs**	Lauter	112	64,4%	47	27,0%	15	8,6%
	Gering	560	72,3%	184	23,7%	31	4,0%
**Einschätzung eigener Lärmbeitrag**	Durchschnittlich	155	68,3%	55	24,2%	17	7,5%
	Groß	21	72,4%	4 (*)	13,8%	4 (*)	13,8%

(*) zu kleine Fallzahlen für solide Auswertungen

7. Belästigung durch Lärm von Passanten und Gaststätten

Die Belästigungsreaktion auf den Lärm von Passanten und Gaststätten ist nahezu deckungsgleich mit derjenigen auf Nachbarschaftslärm. 4,8% fühlen sich ganz erheblich gestört, 72,6% hingegen nur sehr geringfügig (davon knapp 53% *überhaupt nicht*). Im Mittel liegen die Antworten der befragten Probandinnen und Probanden bei 1,71 (18,8%):

Abbildung 165: Belästigung durch Lärm von Passanten und Gaststätten in Prozent

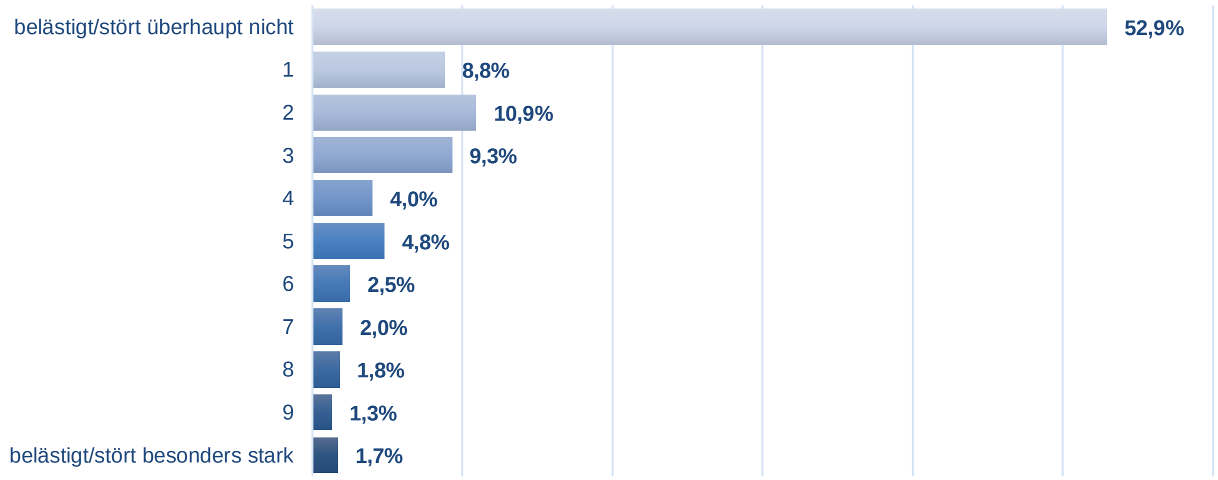

Tabelle 130: Belästigung durch Lärm von Passanten und Gaststätten gruppiert

		Häufigkeit	Gültige Prozente	Kumulierte Prozente
Gültig	Gering belästigt	748	72,6	72,6
	Mittelgradig belästigt	233	22,6	95,2
	Stark belästigt	50	4,8	100,0
	Gesamt	1031	100,0	

7.1. Lärmbelästigung Passanten / Gaststätten und Katastralgemeinden

Das Empfinden der eigenen Belästigung durch den Lärm von Passanten und Gaststätten unterscheidet sich am deutlichsten aller Belästigungsfragen zwischen den einzelnen Katastralgemeinden Innsbrucks und ist in Abhängigkeit von der Anzahl der vorhandenen Lokale, Sehenswürdigkeiten, der Bevölkerungsdichte etc. zu sehen, wodurch sich die inneren Stadtbezirke von den äußeren abheben:

Abbildung 166: Lärmbelästigung Passanten / Gaststätten und Katastralgemeinden im Mittelwertvergleich

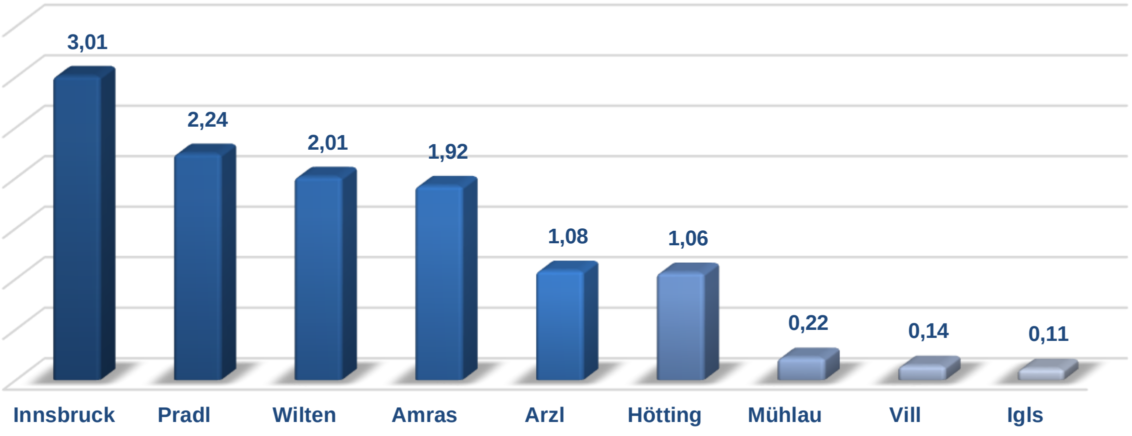

7.2. Lärmbelästigung Passanten / Gaststätten und einzelne Parameter

Im Folgenden einige ausgewählte Parameter, jeweils in Extremgruppen zusammengefasst, in Zusammenschau mit der Belästigung durch den Lärm ausgehend von Passanten und Gaststätten:

Tabelle 131: Belästigung durch Lärm von Passanten und Gaststätten und ausgewählte Parameter

		Belästigung Lärm von Passanten und Gaststätten gruppiert					
		Gering belästigt		Mittelgradig belästigt		Stark belästigt	
		Anzahl	Zeilen (%)	Anzahl	Zeilen (%)	Anzahl	Zeilen (%)
**Geschlecht**	weiblich	382	70,6%	132	24,4%	27	5,0%
	männlich	366	74,7%	101	20,6%	23	4,7%
**Altersgruppen**	18 bis 40 Jahre	306	69,5%	111	25,2%	23	5,2%
	41 bis 60 Jahre	229	72,5%	65	20,6%	22	7,0%

	über 60 Jahre	213	77,5%	57	20,7%	5	1,8%
**Bildungsschicht**	Primärer Schulabschluss	74	75,5%	20	20,4%	4	4,1%
	Sekundärer Schulabschluss	390	72,0%	123	22,7%	29	5,4%
	Tertiärer Schulabschluss	282	72,7%	89	22,9%	17	4,4%
**Innenhof bzw. lärm-abgewandte Seite**	Ja	491	70,0%	179	25,5%	31	4,4%
	Nein	242	78,6%	47	15,3%	19	6,2%
**Nachtschlaf**	Fenster geöffnet/gekippt	620	75,5%	170	20,7%	31	3,8%
	Fenster geschlossen	123	60,9%	60	29,7%	19	9,4%
**Wohnbedingungen**	Nicht gut	49	58,3%	26	31,0%	9	10,7%
	Durchschnittlich	386	69,5%	145	26,1%	24	4,3%
	Sehr gut	311	79,7%	62	15,9%	17	4,4%
**Bedingungen für Kinder**	Nicht gut	23	48,9%	15	31,9%	9	19,1%
	Durchschnittlich	351	68,2%	139	27,0%	25	4,9%
	Sehr gut	356	79,6%	76	17,0%	15	3,4%
**Verbundenheit zu Innsbruck**	Nicht verbunden	35	63,6%	12	21,8%	8	14,5%
	Durchschnittlich	282	74,4%	85	22,4%	12	3,2%
	Sehr verbunden	429	72,1%	136	22,9%	30	5,0%
**Zufriedenheit eigene Wohnsituation**	Nicht zufrieden	17	60,7%	8	28,6%	3	10,7%
	Durchschnittlich	142	55,0%	94	36,4%	22	8,5%
	Sehr zufrieden	588	79,0%	131	17,6%	25	3,4%
**Lebensbedingungen (Summenscore)**	Nicht gut	2	40,0%	2	40,0%	1	20,0%
	Durchschnittlich	457	68,2%	177	26,4%	36	5,4%
	Sehr gut	289	81,2%	54	15,2%	13	3,7%
**Lebensqualität**	Nicht gut	6	60,0%	0	0,0%	4	40,0%
	Durchschnittlich	176	60,7%	102	35,2%	12	4,1%
	Sehr gut	561	77,4%	130	17,9%	34	4,7%
**Lärmempfindlichkeit**	Nicht lärmempfindlich	222	80,7%	42	15,3%	11	4,0%
	Durchschnittlich	413	70,7%	145	24,8%	26	4,5%
	Sehr lärmempfindlich	113	65,7%	46	26,7%	13	7,6%
**Summenscore Lärmcoping**	Coping ausgeprägt	121	50,0%	92	38,0%	29	12,0%
	Coping nicht ausgeprägt	627	79,6%	140	17,8%	21	2,7%
**Gefühlte Zunahme Lärmbelastung**	Ja	271	62,2%	133	30,5%	32	7,3%
	Nein	477	80,2%	100	16,8%	18	3,0%
**Gefühlte Abnahme Lärmbelastung**	Ja	40	69,0%	15	25,9%	3	5,2%
	Nein	708	72,8%	218	22,4%	47	4,8%
**Notwendigkeit (weite-rer) Maßnahmen Lärm**	Maßnahmen notwendig	476	68,9%	171	24,7%	44	6,4%
	Keine Maßnahmen nötig	152	79,2%	36	18,8%	4	2,1%
**Negative Gesund-heitswirkungen Lärm**	Gering	167	77,0%	46	21,2%	4	1,8%
	Stark	557	71,3%	179	22,9%	45	5,8%
**Lautheit Innsbruck im Vergleich zu anderen Städten Österreichs**	Ruhiger	227	77,7%	57	19,5%	8	2,7%
	Gleich	406	73,7%	115	20,9%	30	5,4%
	Lauter	104	59,8%	58	33,3%	12	6,9%

Anm.: In mehreren Subgruppen sind die Fallzahlen für solide Auswertungen zu gering

Die Beziehungen zwischen ausgewählten Items aus dem Fragebogen und der Belästigung durch Passanten und Gaststätten gestaltet sich sehr ähnlich den anderen Belästigungslärmquellen.

8. Belästigung durch Schienenverkehrslärm

Deutlich über zwei Drittel aller befragten Innsbruckerinnen und Innsbrucker *stört* der Schienenverkehrslärm in ihrem Wohnumfeld *überhaupt nicht* und über 80% aller Probandinnen und Probanden sehen sich selbst als nur gering vom Schienenverkehrslärm belästigt an (gruppierte Antwortkategorien 0 [=*belästigt / stört überhaupt nicht*], 1 und 2). Rund 3% sehen sich hingegen als starkbelästigt (*highly annoyed*: gruppierte Antwortkategorien 8, 9 und 10 [=*belästigt / stört besonders stark*]) und 15,6% der Befragten antwortet in den mittleren Kategorien:

Abbildung 167: Belästigung durch Schienenverkehrslärm in Prozent

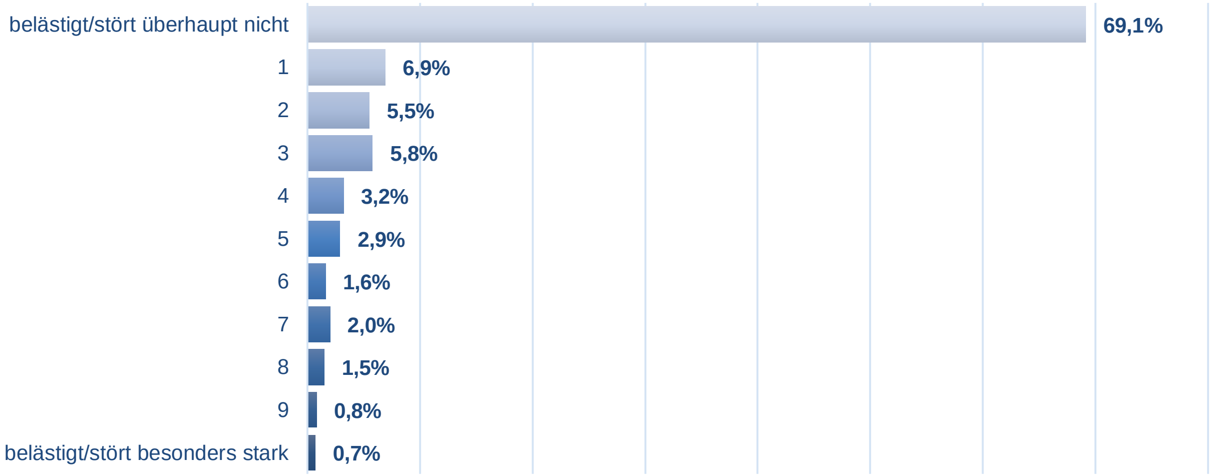

Im Mittel liegen die Antworten der Befragten bei 1,12 (12,3%):

Tabelle 132: Belästigung durch Schienenverkehrslärm gruppiert

		Häufigkeit	Gültige Prozente	Kumulierte Prozente
Gültig	Gering belästigt	840	81,5	81,5
	Mittelgradig belästigt	161	15,6	97,1
	Stark belästigt	30	2,9	100,0
	Gesamt	1031	100,0	

8.1. Schienenlärmbelästigung, -Belastung und -Expositionsempfinden

Im Vergleich mit der Belästigung durch den Straßenverkehrslärm und insbesondere mit dem Flugverkehrslärm ist das deutlich geringere Belästigungsausmaß durch den Schienenverkehrslärm auffällig. Diese geringere Störung der befragten Innsbruckerinnen und Innsbrucker durch den Schienenverkehrslärm spiegelt sich auch in der Zusammenschau der Belästigung durch den Schienenverkehrs-

lärm mit der gegebenen Schienenlärmbelastung. Das Belästigungsausmaß im niederen Belastungsbereich fällt sehr gering aus und steigt erst bei höheren gegebenen Lärmpegeln über 50dB L_{den} stärker an. Das Ausmaß der empfundenen Störung durch den Schienenverkehrslärm deckt sich dabei nahezu 1:1 mit der empfundenen Ausgesetztheit gegenüber demselben. Lediglich in der höchsten Belastungskategorie über 60dB-Dauerbelastung (24h-L_{den}) wird – wie schon beim Flug- und beim Straßenverkehrslärm – die Ausgesetztheit von den Befragten höher angesetzt als die Belästigung:

Abbildung 168: Schienenlärmbelästigung und –Expositionsempfinden und 24h-Schienenlärmbelastung L_{den} in 10dB-Schritten im Mittelwertvergleich

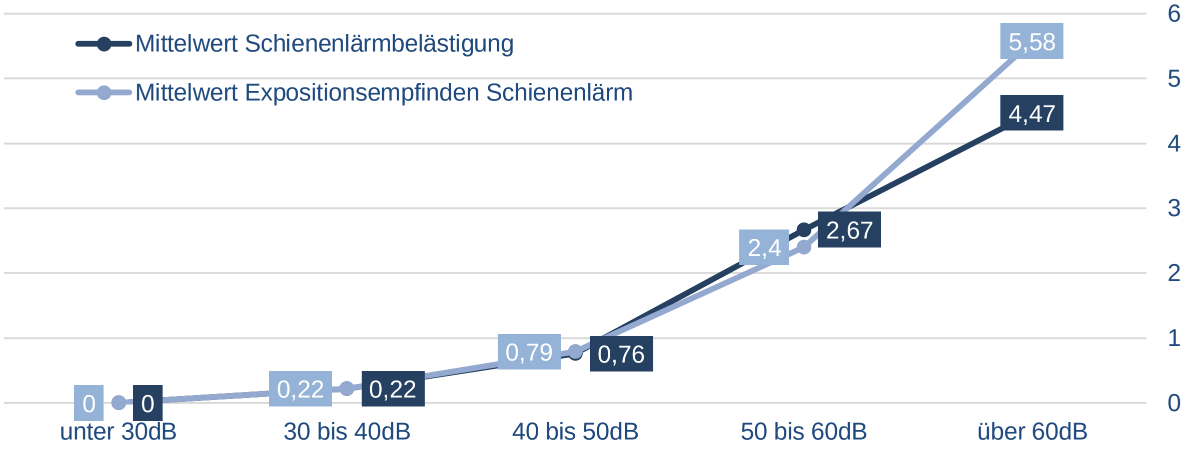

Anm.: Die Darstellung zeigt nachvollziehbare Zusammenhänge auf, enthält jedoch keine Prüfungen auf Kovariablen und andere Prädiktoren und ist somit nicht den Dosis-Wirkungskurven aus der gängigen Fachliteratur gleichzusetzen.

8.2. Schienenlärmbelästigung und Katastralgemeinden

Entsprechend der gegebenen Schienenverkehrslärmbelastung zeigt sich die stärkste Schienenlärmbelästigung in Wilten und Innsbruck. Die geringste Störung durch Schienenlärm im eigenen Wohnumfeld empfinden die Befragten in Igls, Vill und Amras, wo fast überhaupt keine Belästigung durch den Schienenverkehrslärm von den Befragten festgestellt wird:

Abbildung 169: Schienenverkehrslärmbelästigung und Katastralgemeinden im Mittelwertvergleich

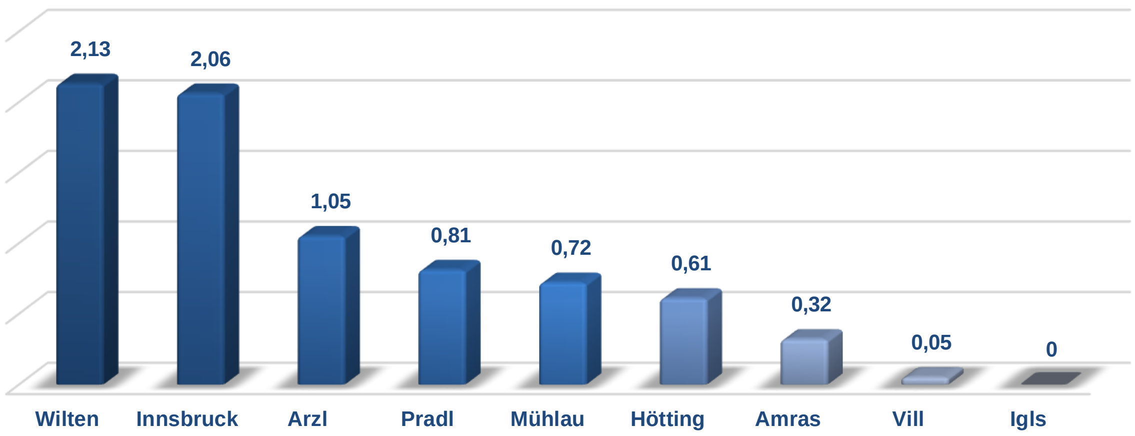

8.3. Schienenlärmbelästigung und ausgewählte Parameter

Wie schon bei den anderen Verkehrslärmbelästigungsauswertungen zeigen sich Frauen auch vom Schienenverkehrslärm stärker belästigt als Männer. Auch steigt das Belästigungsausmaß mit zunehmendem höchsten erreichten Schulabschluss an:

Tabelle 133: Schienenverkehrslärmbelästigung und Bildungsschicht im Mittelwertvergleich

Belästigung Schienenverkehrslärm

Bildungsschicht	Mittelwert	N	Standardabweichung
Primärer Schulabschluss	,51	98	1,548
Sekundärer Schulabschluss	1,09	542	2,039
Tertiärer Schulabschluss	1,34	388	2,397
Insgesamt	1,13	1028	2,153

Die Schienenverkehrslärmbelästigung zeigt insgesamt in der Kombination mit anderen Parametern geringere Bezüge als die Belästigung durch Straßen- und Flugverkehrslärm, aber dieselben Tendenzen: Je höher das Belästigungsausmaß, desto geringer werden Wohn- und Lebensbedingungen etc. eingeschätzt:

Abbildung 170: Güte der Wohnbedingungen und Belästigung durch Schienenverkehrslärm nach 24h-Schienenlärmbelastung L_{den} in 10dB-Schritten im Mittelwertvergleich

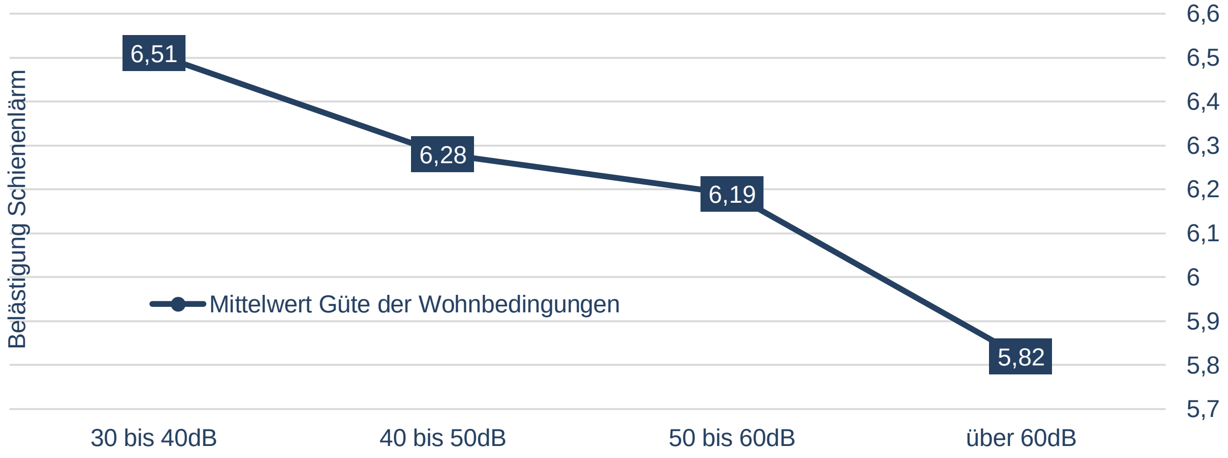

Tabelle 134: Belästigung durch Schienenverkehrslärm und ausgewählte Parameter (gruppiert)

		Belästigung Schienenverkehrslärm gruppiert					
		Gering belästigt		Mittelgradig belästigt		Stark belästigt	
		Anzahl	Zeilen (%)	Anzahl	Zeilen (%)	Anzahl	Zeilen (%)
Geschlecht	weiblich	432	79,9%	91	16,8%	18	3,3%
	männlich	408	83,3%	70	14,3%	12	2,4%
Altersgruppen	18 bis 40 Jahre	357	81,1%	69	15,7%	14	3,2%
	41 bis 60 Jahre	255	80,7%	49	15,5%	12	3,8%
	über 60 Jahre	228	82,9%	43	15,6%	4	1,5%
Bildungsschicht	Primärer Schulabschluss	91	92,9%	5	5,1%	2	2,0%

	Sekundärer Schulabschluss	444	81,9%	87	16,1%	11	2,0%
	Tertiärer Schulabschluss	302	77,8%	69	17,8%	17	4,4%
**Baulicher Schutz vor Verkehrslärm**	Keiner bis geringfügig	608	79,4%	134	17,5%	24	3,1%
	Gut bis komplett	207	87,7%	23	9,7%	6	2,5%
**Innenhof bzw. lärm-abgewandte Seite**	Ja	580	82,7%	104	14,8%	17	2,4%
	Nein	244	79,2%	51	16,6%	13	4,2%
**Nachtschlaf**	Fenster geöffnet/gekippt	683	83,2%	118	14,4%	20	2,4%
	Fenster geschlossen	149	73,8%	43	21,3%	10	5,0%
**Wohnbedingungen**	Nicht gut	66	78,6%	14	16,7%	4	4,8%
	Durchschnittlich	441	79,5%	92	16,6%	22	4,0%
	Sehr gut	331	84,9%	55	14,1%	4	1,0%
**Bedingungen für Kinder**	Nicht gut	34	72,3%	10	21,3%	3	6,4%
	Durchschnittlich	409	79,4%	90	17,5%	16	3,1%
	Sehr gut	379	84,8%	57	12,8%	11	2,5%
**Zufriedenheit eigene Wohnsituation**	Nicht zufrieden	20	71,4%	6	21,4%	2	7,1%
	Durchschnittlich	199	77,1%	49	19,0%	10	3,9%
	Sehr zufrieden	620	83,3%	106	14,2%	18	2,4%
**Lebensbedingungen (Summenscore)**	Nicht gut	2	40,0%	3	60,0%	0	0,0%
	Durchschnittlich	525	78,4%	117	17,5%	28	4,2%
	Sehr gut	313	87,9%	41	11,5%	2	0,6%
**Lebensqualität**	Nicht gut	10	100,0%	0	0,0%	0	0,0%
	Durchschnittlich	227	78,3%	55	19,0%	8	2,8%
	Sehr gut	597	82,3%	106	14,6%	22	3,0%
**Lärmempfindlichkeit**	Nicht lärmempfindlich	237	86,2%	35	12,7%	3	1,1%
	Durchschnittlich	468	80,1%	96	16,4%	20	3,4%
	Sehr lärmempfindlich	135	78,5%	30	17,4%	7	4,1%
**Summenscore Lärmcoping**	Coping ausgeprägt	166	68,6%	60	24,8%	16	6,6%
	Coping nicht ausgeprägt	673	85,4%	101	12,8%	14	1,8%
**Gefühlte Zunahme Lärmbelastung**	Ja	338	77,5%	81	18,6%	17	3,9%
	Nein	502	84,4%	80	13,4%	13	2,2%
**Gefühlte Abnahme Lärmbelastung**	Ja	45	77,6%	11	19,0%	2	3,4%
	Nein	795	81,7%	150	15,4%	28	2,9%
**Notwendigkeit (weite-rer) Maßnahmen Lärm**	Maßnahmen notwendig	544	78,7%	121	17,5%	26	3,8%
	Keine Maßnahmen nötig	167	87,0%	24	12,5%	1	0,5%
**Negative Gesund-heitswirkungen Lärm**	Gering	184	84,8%	27	12,4%	6	2,8%
	Stark	626	80,2%	132	16,9%	23	2,9%
**Lautheit Innsbruck im Vergleich zu anderen Städten Österreichs**	Ruhiger	246	84,2%	44	15,1%	2	0,7%
	Gleich	450	81,7%	81	14,7%	20	3,6%
	Lauter	133	76,4%	33	19,0%	8	4,6%
**Einschätzung eigener Lärmbeitrag**	Gering	634	81,8%	113	14,6%	28	3,6%
	Durchschnittlich	181	79,7%	44	19,4%	2	0,9%
	Groß	25	86,2%	4	13,8%	0	0,0%

Anm.: In mehreren Subgruppen sind die Fallzahlen für solide Auswertungen zu gering

9. Belästigung durch Erschütterungen

Die Belästigungsreaktion auf Erschütterungen ist in ihren Dimensionen der auf Schienenverkehrslärm sehr ähnlich. Über 70% werden von Erschütterungen *überhaupt nicht gestört*, 83,6% des befragten Kollektivs sind der Kategorie der Geringbelästigten zuzuordnen, wohingegen 3,3% sich selbst als stark belästigt einschätzen. Im Mittel liegen die Antworten der befragten Probandinnen und Probanden bei 1,09 (12,0%):

Abbildung 171: Belästigung durch Erschütterungen in Prozent

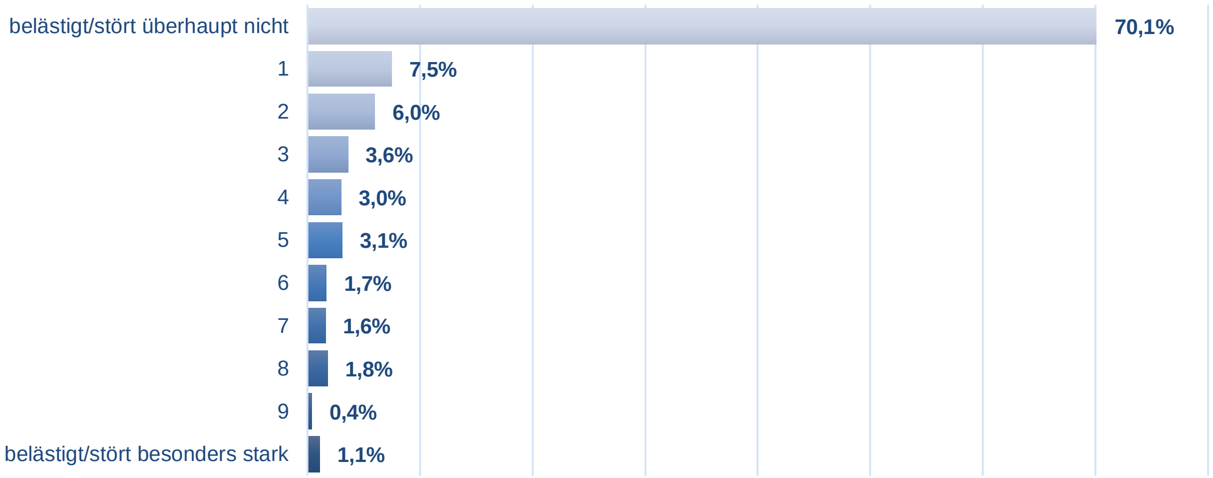

Tabelle 135: Belästigung durch Erschütterungen gruppiert

		Häufigkeit	Gültige Prozente	Kumulierte Prozente
Gültig	Gering belästigt	862	83,6	83,6
	Mittelgradig belästigt	135	13,1	96,7
	Stark belästigt	34	3,3	100,0
	Gesamt	1031	100,0	

9.1. Belästigung durch Erschütterungen und Katastralgemeinden

Abbildung 172: Belästigung durch Erschütterungen und Katastralgemeinden im Mittelwertvergleich

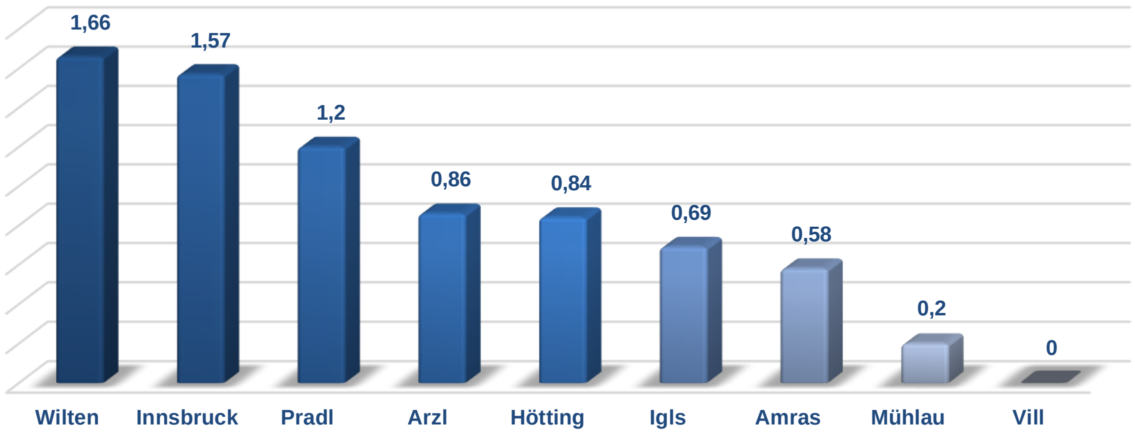

Das größte Ausmaß an Belästigung durch Erschütterungen wird in Wilten und Innsbruck empfunden, das geringste in Vill und Mühlau:

9.2. Belästigung durch Erschütterungen und ausgewählte Parameter

Im Folgenden einige ausgewählte Parameter, jeweils in Extremgruppen zusammengefasst, in Zusammenschau mit der Belästigung durch Erschütterungen:

Tabelle 136: Belästigung durch Erschütterungen und ausgewählte Parameter (gruppiert)

		Belästigung Erschütterungen gesamt gruppiert					
		Gering belästigt		Mittelgradig belästigt		Stark belästigt	
		Anzahl	Zeilen (%)	Anzahl	Zeilen (%)	Anzahl	Zeilen (%)
**Geschlecht**	weiblich	449	83,0%	72	13,3%	20	3,7%
	männlich	413	84,3%	63	12,9%	14	2,9%
**Altersgruppen**	18 bis 40 Jahre	370	84,1%	62	14,1%	8	1,8%
	41 bis 60 Jahre	254	80,4%	44	13,9%	18	5,7%
	über 60 Jahre	238	86,5%	29	10,5%	8	2,9%
**Bildungsschicht**	Primärer Schulabschluss	84	85,7%	11	11,2%	3	3,1%
	Sekundärer Schulabschluss	448	82,7%	72	13,3%	22	4,1%
	Tertiärer Schulabschluss	327	84,3%	52	13,4%	9	2,3%
**Haushalt mit/ohne minderjährige Kinder**	Haushalt ohne Kinder	627	82,3%	110	14,4%	25	3,3%
	Haushalt mit Kindern	235	87,4%	25	9,3%	9	3,3%
**Baulicher Schutz vor Verkehrslärm**	Keiner bis geringfügig	629	82,1%	104	13,6%	33	4,3%
	Gut bis komplett	207	87,7%	28	11,9%	1	0,4%
**Innenhof bzw. lärm-abgewandte Seite**	Ja	586	83,6%	98	14,0%	17	2,4%
	Nein	259	84,1%	32	10,4%	17	5,5%
**Nachtschlaf**	Fenster geöffnet/gekippt	703	85,6%	100	12,2%	18	2,2%
	Fenster geschlossen	153	75,7%	33	16,3%	16	7,9%
**Wohnbedingungen**	Nicht gut	62	73,8%	17	20,2%	5	6,0%
	Durchschnittlich	449	80,9%	84	15,1%	22	4,0%
	Sehr gut	349	89,5%	34	8,7%	7	1,8%
**Bedingungen für Kinder**	Nicht gut	27	57,4%	14	29,8%	6	12,8%
	Durchschnittlich	418	81,2%	77	15,0%	20	3,9%
	Sehr gut	398	89,0%	42	9,4%	7	1,6%
**Verbundenheit zu Innsbruck**	Nicht verbunden	39	70,9%	9	16,4%	7	12,7%
	Durchschnittlich	319	84,2%	51	13,5%	9	2,4%
	Sehr verbunden	502	84,4%	75	12,6%	18	3,0%
**Zufriedenheit eigene Wohnsituation**	Nicht zufrieden	16	57,1%	8	28,6%	4	14,3%
	Durchschnittlich	192	74,4%	49	19,0%	17	6,6%
	Sehr zufrieden	653	87,8%	78	10,5%	13	1,7%
**Lebensbedingungen**	Nicht gut	3	60,0%	2	40,0%	0	0,0%

**(Summenscore)**	Durchschnittlich	536	80,0%	107	16,0%	27	4,0%
	Sehr gut	323	90,7%	26	7,3%	7	2,0%
	Nicht gut	4	40,0%	3	30,0%	3	30,0%
**Lebensqualität**	Durchschnittlich	219	75,5%	55	19,0%	16	5,5%
	Sehr gut	634	87,4%	76	10,5%	15	2,1%
	Nicht lärmempfindlich	247	89,8%	21	7,6%	7	2,5%
**Lärmempfindlichkeit**	Durchschnittlich	478	81,8%	88	15,1%	18	3,1%
	Sehr lärmempfindlich	137	79,7%	26	15,1%	9	5,2%
**Summenscore**	Coping ausgeprägt	156	64,5%	64	26,4%	22	9,1%
**Lärmcoping**	Coping nicht ausgeprägt	706	89,6%	71	9,0%	11	1,4%
**Gefühlte Zunahme**	Ja	335	76,8%	74	17,0%	27	6,2%
**Lärmbelastung**	Nein	527	88,6%	61	10,3%	7	1,2%
**Gefühlte Abnahme**	Ja	51	87,9%	7	12,1%	0	0,0%
**Lärmbelastung**	Nein	811	83,4%	128	13,2%	34	3,5%
**Notwendigkeit (weite-**	Maßnahmen notwendig	554	80,2%	109	15,8%	28	4,1%
**rer) Maßnahmen Lärm**	Keine Maßnahmen nötig	168	87,5%	20	10,4%	4	2,1%
**Negative Gesund-**	Gering	196	90,3%	18	8,3%	3	1,4%
**heitswirkungen Lärm**	Stark	635	81,3%	115	14,7%	31	4,0%
**Lautheit Innsbruck im**	Ruhiger	258	88,4%	31	10,6%	3	1,0%
**Vergleich zu anderen**	Gleich	461	83,7%	70	12,7%	20	3,6%
**Städten Österreichs**	Lauter	129	74,1%	34	19,5%	11	6,3%

Anm.: In mehreren Subgruppen sind die Fallzahlen für solide Auswertungen zu gering

10. Belästigung durch Lärm aus Gewerbe und Industrie

1,5% aller befragten Innsbruckerinnen und Innsbrucker fühlen sich durch den Lärm verursacht von Gewerbe und Industrie stark belästigt, über 90% hingegen nur geringfügig gestört, unter diesen über 80% *überhaupt nicht*:

Abbildung 173: Belästigung durch Lärm aus Gewerbe und Industrie in Prozent

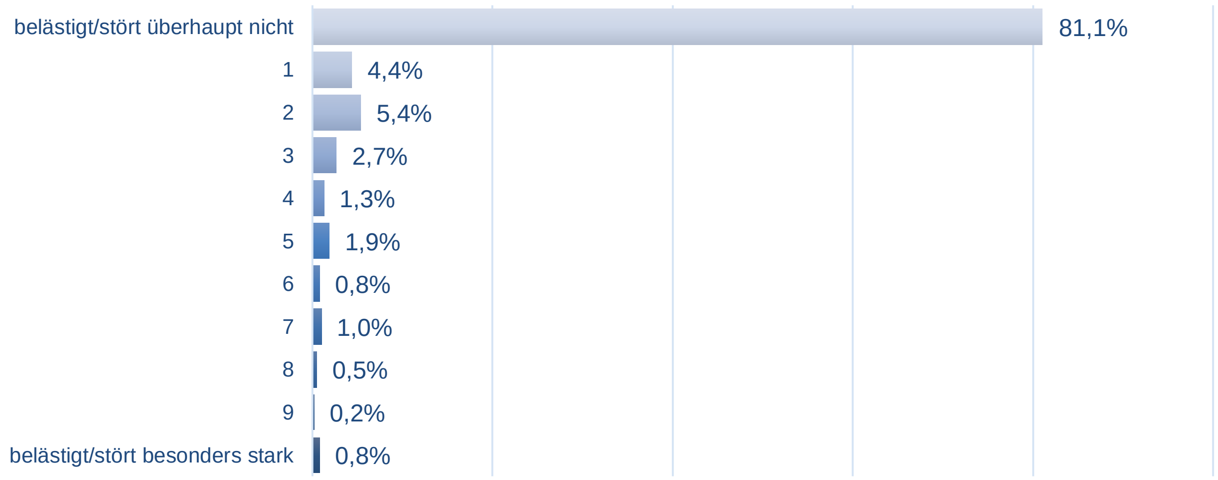

Im Mittel liegen die Antworten der befragten Innsbruckerinnen und Innsbrucker bei 0,63 (6,9%).

Tabelle 137: Belästigung durch Lärm aus Gewerbe u. Industrie gruppiert

		Häufigkeit	Gültige Prozente	Kumulierte Prozente
Gültig	Gering belästigt	937	90,9	90,9
	Mittelgradig belästigt	79	7,7	98,5
	Stark belästigt	15	1,5	100,0
	Gesamt	1031	100,0	

10.1. Lärmbelästigung Gewerbe und Industrie und Katastralgemeinden

Die Störung durch Lärm von Gewerbe und Industrie ist in Mühlau und Vill am geringsten und in Innsbruck, Wilten und Arzl am stärksten ausgeprägt:

Abbildung 174: Lärmbelästigung Gewerbe und Industrie und Katastralgemeinden im Mittelwertvergleich

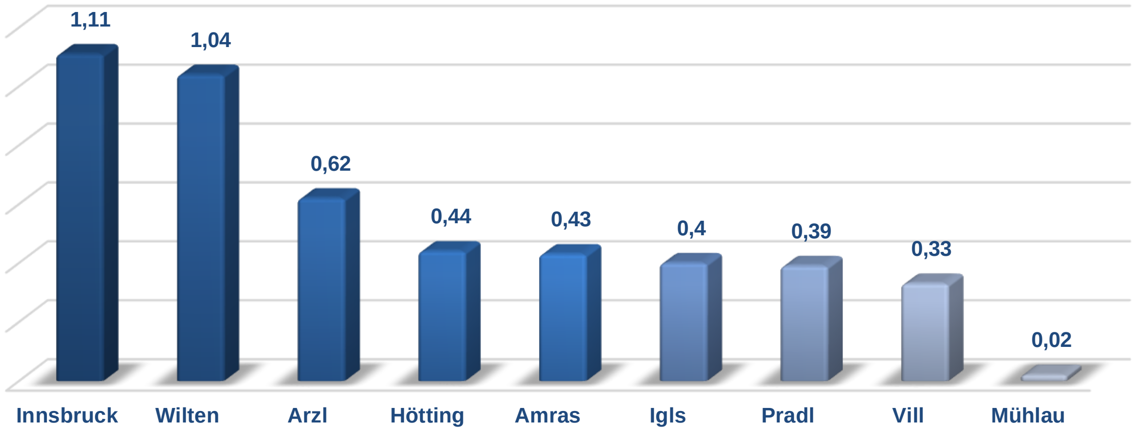

10.2. Lärmbelästigung Gewerbe und Industrie und einzelne Parameter

Die Auswertungen zur Belästigung durch den Lärm aus Gewerbe und Industrie zeigen keine Auffälligkeiten im Vergleich zu den anderen Belästigungslärmquellen.

Im Folgenden einige ausgewählte Parameter, jeweils in Extremgruppen zusammengefasst, in Zusammenschau mit der Belästigung durch den Lärm aus Gewerbe und Industrie:

Tabelle 138: Belästigung durch Lärm aus Gewerbe und Industrie und ausgewählte Parameter

		Belästigung Lärm aus Gewerbe u. Industrie gruppiert			
		Gering belästigt		Mittelgradig belästigt	
		Anzahl	Zeilen (%)	Anzahl	Zeilen (%)
**Geschlecht**	weiblich	497	91,9%	35	6,5%
	männlich	440	89,8%	44	9,0%
**Altersgruppen**	18 bis 40 Jahre	397	90,2%	37	8,4%
	41 bis 60 Jahre	286	90,5%	26	8,2%
	über 60 Jahre	254	92,4%	16	5,8%
**Bildungsschicht**	Primärer Schulabschluss	92	93,9%	4	4,1%
	Sekundärer Schulabschluss	497	91,7%	39	7,2%
	Tertiärer Schulabschluss	345	88,9%	36	9,3%
**Haushalt mit/ohne minderjährige Kinder**	Haushalt ohne Kinder	688	90,3%	63	8,3%
	Haushalt mit Kindern	249	92,6%	16	5,9%
**Baulicher Schutz vor Verkehrslärm**	Keiner bis geringfügig	686	89,6%	66	8,6%
	Gut bis komplett	223	94,5%	12	5,1%
**Innenhof bzw. lärm-abgewandte Seite**	Ja	645	92,0%	52	7,4%
	Nein	272	88,3%	25	8,1%
**Nachtschlaf**	Fenster geöffnet/gekippt	754	91,8%	58	7,1%
	Fenster geschlossen	176	87,1%	20	9,9%
**Wohnbedingungen**	Nicht gut	77	91,7%	5	6,0%
	Durchschnittlich	498	89,7%	49	8,8%
	Sehr gut	360	92,3%	25	6,4%
**Bedingungen für Kinder**	Nicht gut	36	76,6%	10	21,3%
	Durchschnittlich	457	88,7%	46	8,9%
	Sehr gut	424	94,9%	21	4,7%
**Verbundenheit zu Innsbruck**	Nicht verbunden	47	85,5%	6	10,9%
	Durchschnittlich	340	89,7%	33	8,7%
	Sehr verbunden	548	92,1%	40	6,7%
**Zufriedenheit eigene Wohnsituation**	Nicht zufrieden	21	75,0%	5	17,9%
	Durchschnittlich	219	84,9%	31	12,0%
	Sehr zufrieden	696	93,5%	43	5,8%
**Lebensbedingungen (Summenscore)**	Nicht gut	4	80,0%	0	0,0%
	Durchschnittlich	597	89,1%	61	9,1%
	Sehr gut	336	94,4%	18	5,1%
**Lebensqualität**	Nicht gut	8	80,0%	1	10,0%
	Durchschnittlich	252	86,9%	35	12,1%
	Sehr gut	671	92,6%	43	5,9%
**Lärmempfindlichkeit**	Nicht lärmempfindlich	263	95,6%	11	4,0%
	Durchschnittlich	524	89,7%	49	8,4%
	Sehr lärmempfindlich	150	87,2%	19	11,0%
**Summenscore Lärmcoping**	Coping ausgeprägt	189	78,1%	41	16,9%
	Coping nicht ausgeprägt	747	94,8%	38	4,8%

**Gefühlte Zunahme Lärmbelastung**	Ja	376	86,2%	48	11,0%
	Nein	561	94,3%	31	5,2%
**Gefühlte Abnahme Lärmbelastung**	Ja	57	98,3%	1	1,7%
	Nein	880	90,4%	78	8,0%
**Notwendigkeit (weite-rer) Maßnahmen Lärm**	Maßnahmen notwendig	615	89,0%	62	9,0%
	Keine Maßnahmen nötig	182	94,8%	10	5,2%
**Negative Gesund-heitswirkungen Lärm**	Gering	207	95,4%	10	4,6%
	Stark	698	89,4%	68	8,7%
**Lautheit Innsbruck im Vergleich zu anderen Städten Österreichs**	Ruhiger	274	93,8%	14	4,8%
	Gleich	501	90,9%	42	7,6%
	Lauter	148	85,1%	23	13,2%
**Einschätzung eigener Lärmbeitrag**	Gering	712	91,9%	54	7,0%
	Durchschnittlich	201	88,5%	22	9,7%
	Groß	24	82,8%	3	10,3%

Anm.: Die Fallzahlen in der Extremgruppe der Starkbelästigten (highly annoyed, N=15) sind für solide Auswertungen zu gering, weshalb diese Gruppe in der obenstehenden Tabelle ausgespart wurde.

11. Belästigung durch Autobahnlärm

Die Belästigung durch Autobahnlärm im urbanen Stadtgebiet von Innsbruck fällt im befragten Kollektiv vergleichsweise gering aus und nimmt unter allen 10 abgefragten Lärmquellen die geringsten Ausprägungen an. 2,1% empfinden sich als stark belästigt (*highly annoyed*: gruppierte Antwortkategorien 8, 9 und 10 [=*belästigt / stört besonders stark*]), 91,9% hingegen als *überhaupt nicht belästigt* (82,3%) bis gering vom Autobahnlärm gestört (gruppierte Antwortkategorien 0 [=*belästigt / stört überhaupt nicht*], 1 und 2). Im Mittel liegen die Antworten der befragten Probandinnen und Probanden bei 0,6 (6,6%):

Abbildung 175: Belästigung durch Autobahnlärm in Prozent

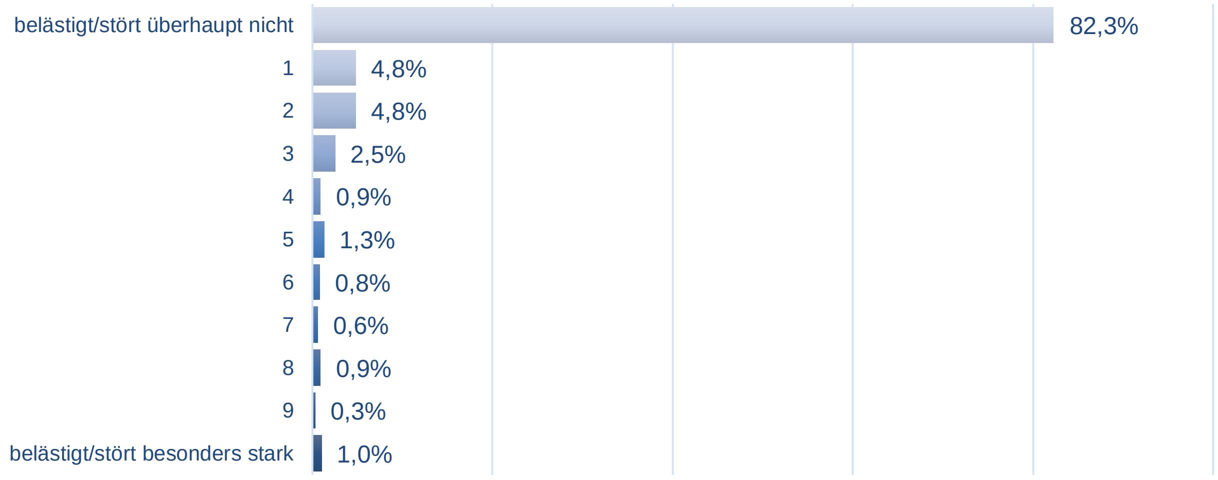

Tabelle 139: Belästigung durch Autobahnlärm gruppiert

		Häufigkeit	Gültige Prozente	Kumulierte Prozente
Gültig	Gering belästigt	947	91,9	91,9
	Mittelgradig belästigt	62	6,0	97,9
	Stark belästigt	22	2,1	100,0
	Gesamt	1031	100,0	

11.1. Autobahnlärmbelästigung, -Belastung und -Expositionsempfinden

Auch in der Zusammenschau der Belästigung durch Autobahnlärm mit der gegebenen Autobahnlärmbelastung zeigt sich ein deutlicher Zusammenhang. Das Ausmaß der empfundenen Störung durch den Autobahnlärm deckt sich dabei nahezu 1:1 mit der empfundenen Ausgesetztheit.

Sehr ähnlich dem Bezug zum Straßenverkehrslärm ist das Belästigungsausmaß im niederen Belastungsbereich sehr gering und steigt bei höheren gegebenen Lärmpegeln über 50dB L_{den} stark an:

Abbildung 176: Autobahnlärmbelästigung und –Expositionsempfinden und 24h-Autobahnlärmbelastung L_{den} in 10dB-Schritten im Mittelwertvergleich

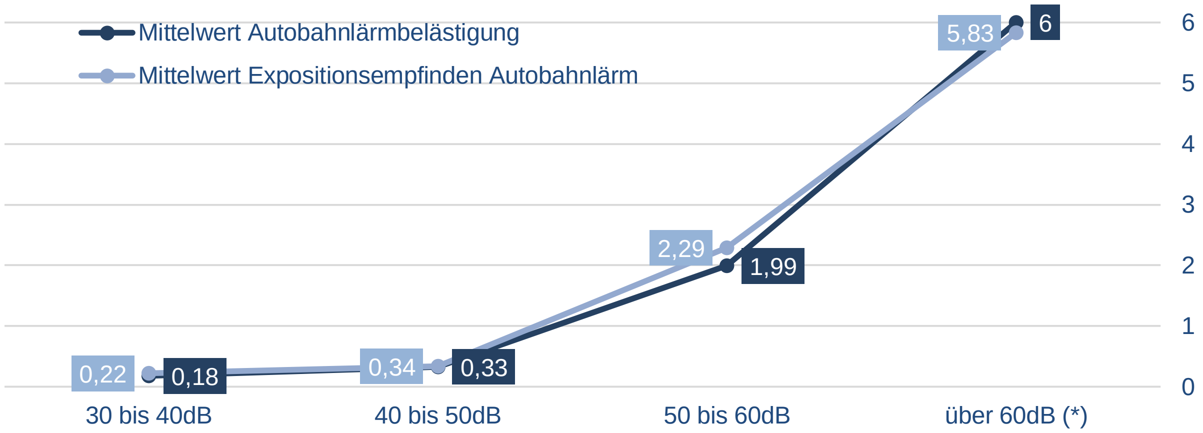

(*) N umfasst in dieser Belastungskategorie lediglich 6 befragte Personen

Anm.: Die Darstellung zeigt nachvollziehbare Zusammenhänge auf, enthält jedoch keine Prüfungen auf Kovariablen und andere Prädiktoren und ist somit nicht den Dosis-Wirkungskurven aus der gängigen Fachliteratur gleichzusetzen.

11.2. Belästigung durch Autobahnlärm und Katastralgemeinden

Die im Mittel stärkste Belästigung durch Autobahnlärm zeigt sich in Wilten, gefolgt von Vill. Die geringste Störung durch Autobahnlärm im eigenen Wohnumfeld empfinden die Befragten in der Innenstadt (Katastralgemeinde Innsbruck), sowie in Igls, Pradl und Mühlau:

Abbildung 177: Belästigung durch Autobahnlärm und Katastralgemeinden im Mittelwertvergleich

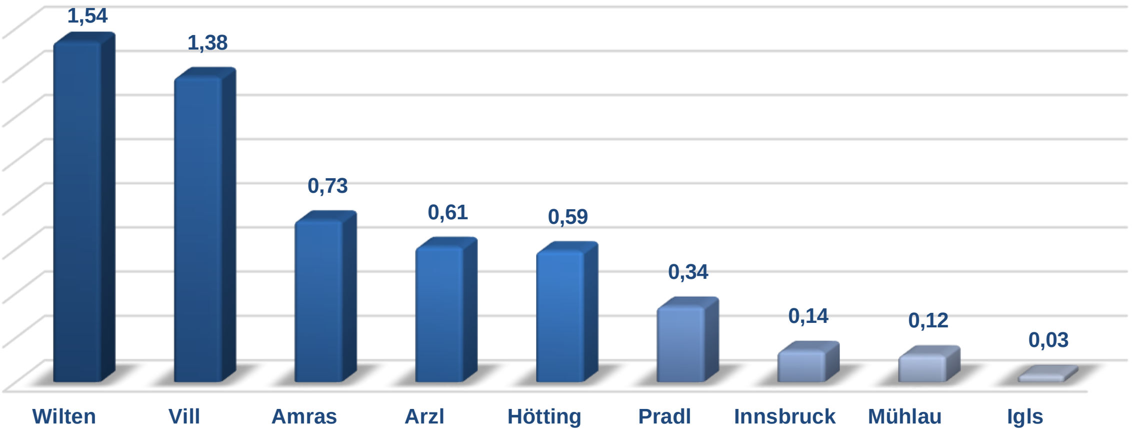

11.3. Belästigung durch Autobahnlärm und ausgewählte Parameter

Die Auswertungen zur Belästigung durch Autobahnlärm zeigen keine Auffälligkeiten im Vergleich zu den anderen Belästigungslärmquellen. Allerdings sind die Fallzahlen der Mittel- und Starkbelasteten sehr gering, weshalb die Aussagekraft der Extremgruppenvergleiche eingeschränkt ist. Besonders in der Extremgruppe der Starkbelästigten (*highly annoyed*, N=22) sind zu wenige Fälle für solide Auswertungen enthalten, weshalb diese Gruppe in der untenstehenden Tabelle ganz ausgespart wurde.

Im Folgenden einige ausgewählte Parameter, jeweils in die beiden Gruppen der gering und der mittelgradig Belasteten zusammengefasst, in Zusammenschau mit der Belästigung durch Autobahnlärm:

Tabelle 140: Belästigung durch Autobahnlärm und ausgewählte Parameter (gruppiert)

		Belästigung Autobahnlärm gruppiert			
		Gering belästigt		Mittelgradig belästigt	
		Anzahl	Zeilen (%)	Anzahl	Zeilen (%)
**Geschlecht**	weiblich	495	91,5%	31	5,7%
	männlich	452	92,2%	31	6,3%
**Altersgruppen**	18 bis 40 Jahre	409	93,0%	24	5,5%
	41 bis 60 Jahre	287	90,8%	19	6,0%
	über 60 Jahre	251	91,3%	19	6,9%
**Bildungsschicht**	Primärer Schulabschluss	92	93,9%	4	4,1%
	Sekundärer Schulabschluss	494	91,1%	38	7,0%
	Tertiärer Schulabschluss	358	92,3%	20	5,2%
**Haushalt mit/ohne minderjährige Kinder**	Haushalt ohne Kinder	702	92,1%	49	6,4%
	Haushalt mit Kindern	245	91,1%	13	4,8%
**Baulicher Schutz vor Verkehrslärm**	Keiner bis geringfügig	694	90,6%	53	6,9%
	Gut bis komplett	226	95,8%	8	3,4%
**Innenhof bzw. lärmabgewandte Seite**	Ja	659	94,0%	33	4,7%
	Nein	268	87,0%	29	9,4%
**Nachtschlaf**	Fenster geöffnet/gekippt	754	91,8%	50	6,1%

	Fenster geschlossen	186	92,1%	12	5,9%
**Wohnbedingungen**	Nicht gut	74	88,1%	6	7,1%
	Durchschnittlich	507	91,4%	38	6,8%
	Sehr gut	364	93,3%	18	4,6%
**Bedingungen für Kinder**	Nicht gut	41	87,2%	4	8,5%
	Durchschnittlich	461	89,5%	41	8,0%
	Sehr gut	424	94,9%	16	3,6%
**Verbundenheit zu Innsbruck**	Nicht verbunden	51	92,7%	2	3,6%
	Durchschnittlich	349	92,1%	23	6,1%
	Sehr verbunden	545	91,6%	37	6,2%
**Zufriedenheit eigene Wohnsituation**	Nicht zufrieden	22	78,6%	4	14,3%
	Durchschnittlich	236	91,5%	17	6,6%
	Sehr zufrieden	688	92,5%	41	5,5%
**Lebensbedingungen (Summenscore)**	Nicht gut (*)	3	60,0%	1	20,0%
	Durchschnittlich	608	90,7%	48	7,2%
	Sehr gut	336	94,4%	13	3,7%
**Lebensqualität**	Nicht gut	8	80,0%	2 (*)	20,0%
	Durchschnittlich	260	89,7%	23	7,9%
	Sehr gut	673	92,8%	37	5,1%
**Lärmempfindlichkeit**	Nicht lärmempfindlich	261	94,9%	10	3,6%
	Durchschnittlich	533	91,3%	39	6,7%
	Sehr lärmempfindlich	153	89,0%	13	7,6%
**Summenscore Lärmcoping**	Coping ausgeprägt	209	86,4%	20	8,3%
	Coping nicht ausgeprägt	737	93,5%	42	5,3%
**Gefühlte Zunahme Lärmbelastung**	Ja	389	89,2%	32	7,3%
	Nein	558	93,8%	30	5,0%
**Gefühlte Abnahme Lärmbelastung**	Ja	55	94,8%	2 (*)	3,4%
	Nein	892	91,7%	60	6,2%
**Notwendigkeit (weiterer) Maßnahmen Lärm**	Maßnahmen notwendig	623	90,2%	49	7,1%
	Keine Maßnahmen nötig	181	94,3%	8	4,2%
**Negative Gesundheitswirkungen Lärm**	Gering	203	93,5%	10	4,6%
	Stark	714	91,4%	50	6,4%
**Lautheit Innsbruck im Vergleich zu anderen Städten Österreichs**	Ruhiger	272	93,2%	16	5,5%
	Gleich	511	92,7%	33	6,0%
	Lauter	151	86,8%	12	6,9%
**Einschätzung eigener Lärmbeitrag**	Gering	720	92,9%	39	5,0%
	Durchschnittlich	203	89,4%	19	8,4%
	Groß	24	82,8%	4 (*)	13,8%

(*) zu kleine Fallzahlen für solide Auswertungen

Belästigung / Störung durch Lärm im Schlaf

1. Überblick Belästigung / Störung durch Lärm im Schlaf

1.1. Belästigung im Schlaf nach Lärmquelle

Die Einschätzung der Belästigung / Störung durch Lärm im Schlaf wurde für jede einzelne Lärmquelle mittels einer 11-teiligen Skala von 0=*belästigt / stört überhaupt nicht* bis 10=*belästigt / stört besonders stark* abgefragt.

Im Mittelwertvergleich der einzelnen Quellen verändert sich das Bild der Belästigungsreaktion auf Lärm im Schlaf im Vergleich zur Belästigungsausprägung auf Lärm im 24h-Zeitraum: Zunächst ist festzuhalten, dass sich das Ausmaß der Belästigung im Nachtzeitraum etwa halbiert. Des Weiteren rückt der Straßenverkehrslärm an die erste Stelle als störendste Lärmquelle, gefolgt vom verursachten Lärm durch Passanten und Gaststätten und dem Nachbarschaftslärm:

Abbildung 178: Mittelwertvergleich der Belästigung im Schlaf nach Quelle

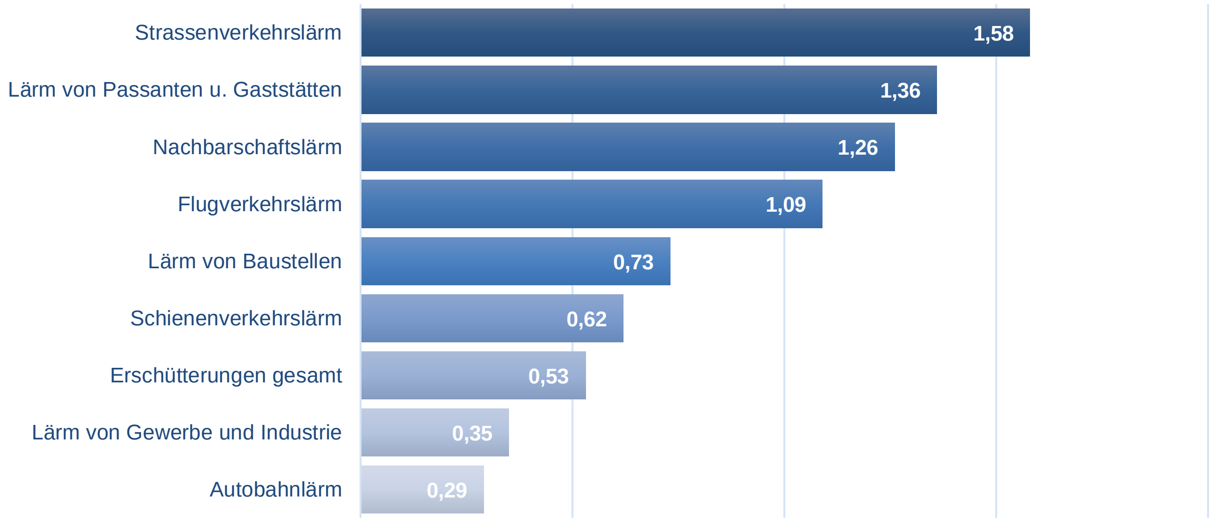

Der Flugverkehrslärm fällt an die vierte Stelle, der Baustellenlärm an die fünfte Stelle zurück. Erschütterungen, Lärm von Gewerbe und Industrie und Autobahnlärm verbleiben in dieser Reihenfolge auf den Plätzen, der am wenigsten belästigendsten (Lärm-)Quellen.[13] Der Mittelwert aller Antworten über alle 9 Fragen hinweg liegt bei 0,87 (9,5%):

Tabelle 141: Mittelwerte der Belästigung im Schlaf nach Quelle

	Strassen-verkehr	Passanten u. Gaststätten	Nach-barschaft	Flug-verkehr	Bau-stellen	Schienen-verkehr	Erschüt-terungen	Gewerbe u. Industrie	Auto-bahn
Mittelwert	1,58	1,36	1,26	1,09	,73	,62	,53	,35	,29
N	1031	1031	1031	1031	1030	1031	1031	1031	1031
Standard-abweichung	2,519	2,475	2,337	2,232	1,914	1,725	1,523	1,348	1,238
Standardfehler des MW	,078	,077	,073	,070	,060	,054	,047	,042	,039

[13] Anm.: Die eigenständige Frage nach der Belästigung / Störung durch *Lärm im Schlaf insgesamt* wird in den folgenden vergleichenden Zusammenstellungen der einzelnen Lärmquellen ausgeklammert und im anschließenden Unterkapitel dargestellt.

Kategorisiert in 3 Belästigungsgruppen:

– ***gering belästigt****:* gruppierte Antwortkategorien 0 [=*belästigt / stört überhaupt nicht*]), 1 und 2
– ***mittelgradig belästigt***: gruppierte Antwortkategorien 3 bis 7
– ***stark belästigt (highly annoyed sleep)****:* gruppierte Antwortkategorien 8, 9 und 10 [=*belästigt / stört besonders stark*])

stellt sich die Belästigungsreaktion im Schlaf nach Quelle wie folgt dar:

Abbildung 179: Belästigung im Schlaf nach Quelle kategorisiert in 3 Expositionsgruppen in Prozent

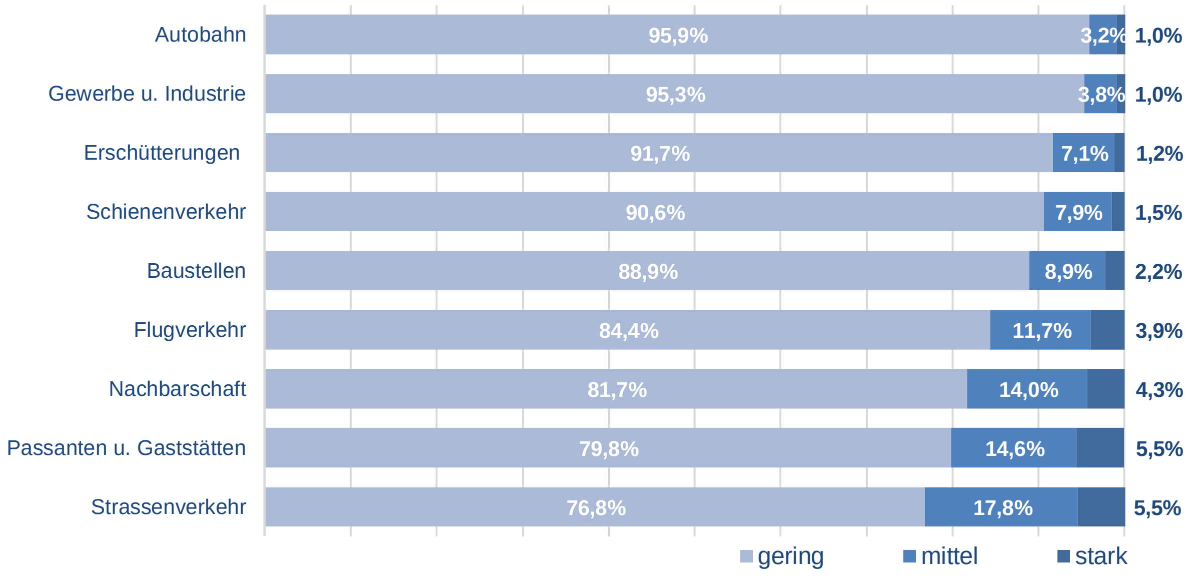

* Nach Belästigungsausmaß (Mittelwert) aufsteigend sortiert

Wird nur die Extremgruppe der sich im Schlaf stark belästigt fühlenden Befragten (*highly annoyed sleep)* betrachtet*,* zeigt sich im Vergleich der Quellen dasselbe Bild, wobei sich die Belästigungsreaktionen auf den Lärm von Passanten und Gaststätten und auf den Straßenverkehrslärm ebenso die Waage halten, wie die Ausprägung der Extremgruppen in Bezug auf den Autobahnlärm und den Lärm von Gewerbe und Industrie:

Abbildung 180: Extremgruppenvergleich *highly annoyed sleep* nach Quelle in Prozent

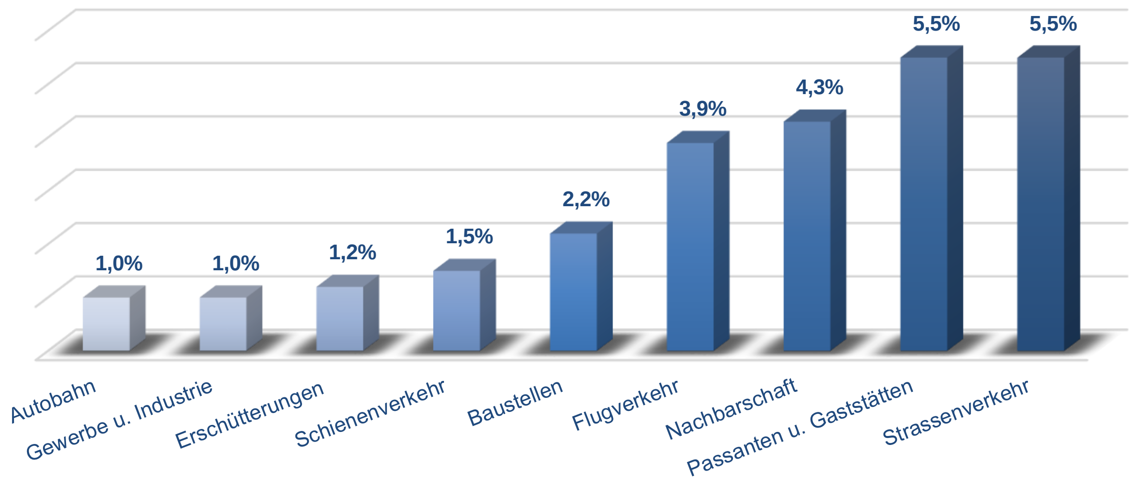

* Nach Belästigungsausmaß (Häufigkeit) aufsteigend sortiert

1.2. Belästigung im Schlaf und Verkehrslärmbelastung

Das Ausmaß der Belästigung durch Verkehrslärm zeigt auch für den Nachtzeitraum (22h bis 6h) einen starken Zusammenhang zur gegebenen Lärmbelastung: Je höher die Verkehrslärmbelastung, desto höher die Belästigung. Die Unterschiede zwischen den drei Verkehrslärmquellen Straße – Schiene – Flugverkehr nivellieren sich jedoch.

Aufgrund der geringeren Flugverkehrslärmbelastung der Innsbruckerinnen und Innsbrucker in der Nacht ist dieser im gruppierten Vergleich nur für die Geringbelasteten <45dB sinnvoll heranzuziehen, nimmt in dieser Belastungsgruppe aber auch in den Nachtstunden den Platz als störendste Lärmquelle ein. Die im Schlaf störendste Verkehrslärmquelle insgesamt ist der Straßenverkehr. Der für den 24h-Zeitraum deutliche Abstand zum Belästigungsausmaß des Schienenverkehrs verringert sich:

Abbildung 181: Belästigung im Schlaf und Verkehrslärmbelastung nach Quelle Nachtlärmpegel (L_n) gruppiert im Mittelwertvergleich

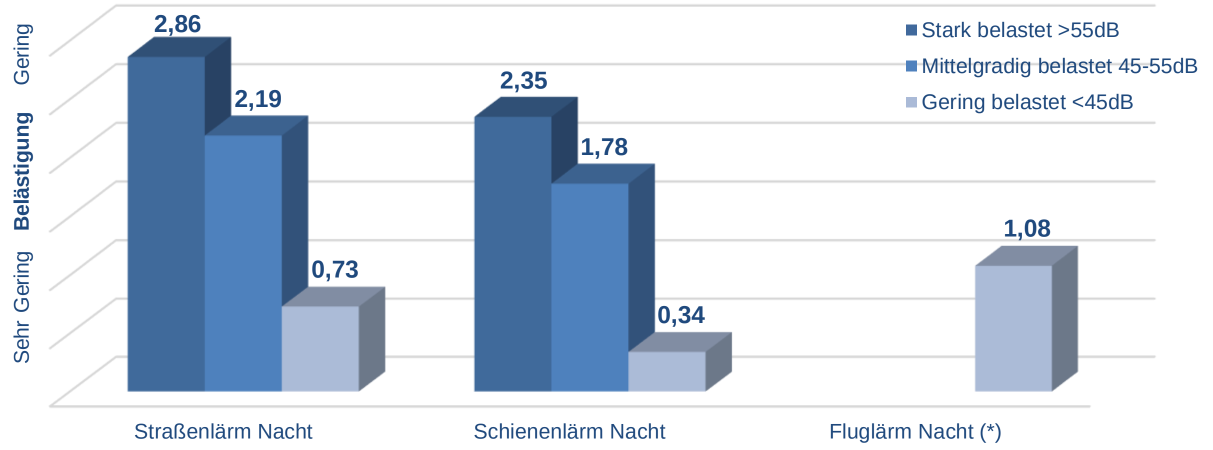

(*) Keine stark Fluglärmbelasteten in der Nacht, lediglich 4 mittelgradig Fluglärmbelastete

Im Vergleich des gemittelten Belästigungsausmaßes im Schlaf nach vorhandener Lärmbelastung in 10dB-Schritten (Nachtlärmpegel L_n) nach Verkehrslärmquelle zeigt sich erneut das höhere Belästigungsausmaß des Flugverkehrslärms gegenüber dem Straßen- und Schienenverkehrslärm:

Abbildung 182: Belästigung im Schlaf und Verkehrslärmbelastung nach Quelle Nachtlärmpegel (L_n) in 10dB-Schritten im Mittelwertvergleich

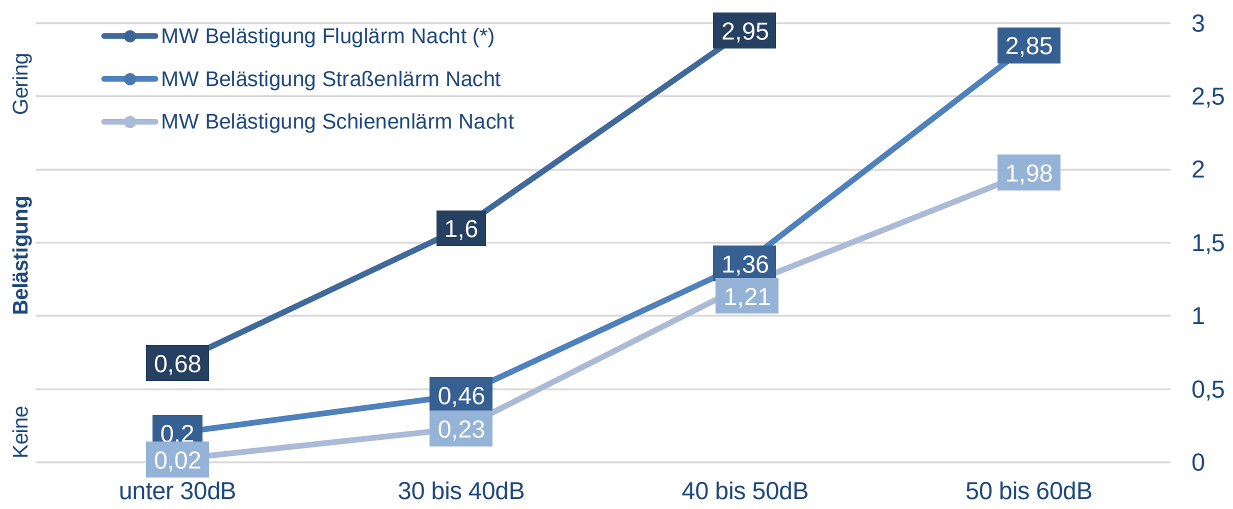

Die Darstellung zeigt nachvollziehbare Zusammenhänge auf, enthält jedoch keine Prüfungen auf Kovariablen und andere Prädiktoren und ist somit nicht den Dosis-Wirkungskurven aus der gängigen Fachliteratur gleichzusetzen.

Weiters ist erkennbar, dass die Belästigungsreaktion sich bereits bei deutlich niedrigeren Lärmpegeln zu verstärken beginnt und kontinuierlicher ansteigt als in Zusammenschau mit den 24h-Pegeln. Ein „Schwellenwert" ab dem eine gesteigerte Belästigung / Störung durch den Verkehrslärm eintritt, ist für die Belästigung im Schlaf nicht auf den ersten Blick auszumachen.

1.3. Belästigung im Schlaf und Katastralgemeinden

1.3.1. Belästigung im Schlaf nach Quelle und Katastralgemeinden

Insgesamt am stärksten im Schlaf vom Lärm belästigt / gestört zeigen sich die Befragten in Wilten und Innsbruck mit deutlichem Abstand gefolgt von Pradl und Hötting. In der Summe aller Lärmquellen am geringsten belästigt empfinden sich die Bewohnerinnen und Bewohner von Igls, Vill und Mühlau:

Abbildung 183: Belästigung im Schlaf nach Lärmquelle und Katastralgemeinde im Mittelwertvergleich

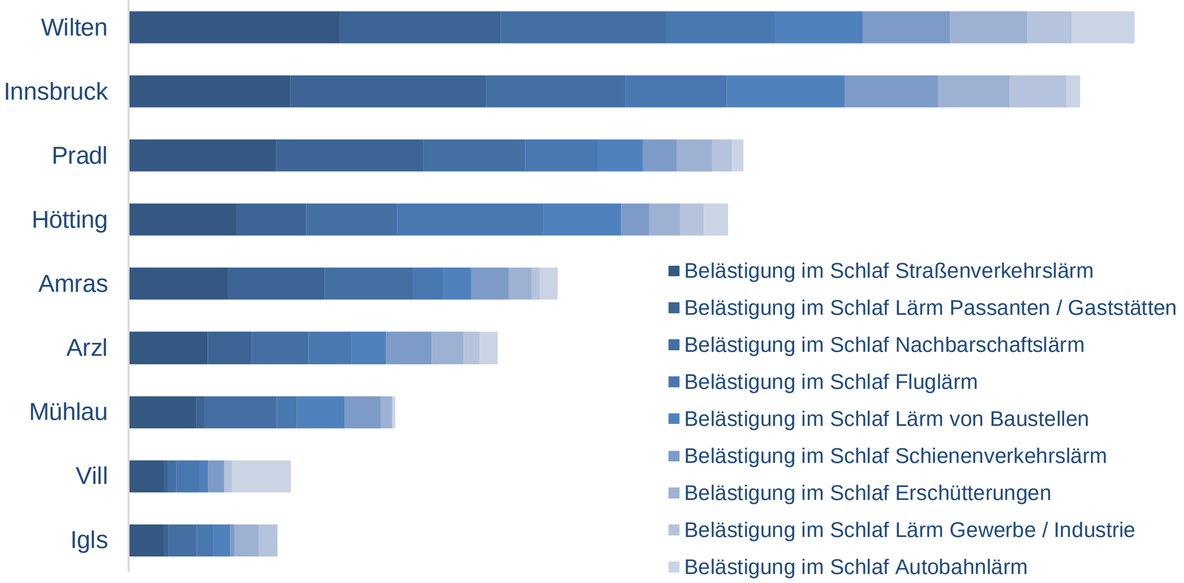

* Nach Belästigungsausmaß (Häufigkeit) absteigend sortiert

Tabelle 142: Belästigung im Schlaf nach Lärmquelle und Katastralgemeinde im Mittelwertvergleich

		Belästigung im Schlaf durch Lärm ausgehend von								
**Katastralgemeinde**		**Straße**	**Passanten / Gaststätten**	**Nachbarschaft**	**Flug**	**Baustellen**	**Schiene**	**Erschütterungen**	**Gewerbe / Industrie**	**Autobahn**
**Amras**	**MW**	**1,20**	**1,18**	**1,07**	**,38**	**,33**	**,45**	**,28**	**,10**	**,22**
	N	60	60	60	60	60	60	60	60	60
	SA	2,413	2,274	2,462	1,342	1,020	1,799	1,303	,440	,993
**Arzl**	**MW**	**,96**	**,54**	**,68**	**,52**	**,43**	**,55**	**,39**	**,19**	**,22**
	N	102	102	102	102	102	102	102	102	102
	SA	1,934	1,615	1,549	1,233	1,583	1,633	1,365	,909	1,021
**Hötting**	**MW**	**1,31**	**,85**	**1,10**	**1,77**	**,95**	**,34**	**,37**	**,29**	**,29**

	N	243	243	243	243	243	243	243	243	243
	SA	2,434	2,058	2,167	2,913	6,550	1,365	1,343	1,403	1,206
	**MW**	**,43**	**,06**	**,34**	**,20**	**,20**	**,06**	**,29**	**,23**	**,00**
**Igls**	N	35	35	35	35	35	35	35	35	35
	SA	,778	,236	,838	,632	,473	,338	,860	1,031	,000
	**MW**	**1,96**	**2,38**	**1,69**	**1,23**	**1,43**	**1,13**	**,87**	**,69**	**,16**
**Innsbruck**	N	176	176	176	176	176	176	176	176	176
	SA	2,619	3,040	2,479	2,337	2,551	2,268	1,779	1,830	1,078
	**MW**	**,82**	**,10**	**,88**	**,24**	**,58**	**,44**	**,14**	**,00**	**,04**
**Mühlau**	N	50	50	50	50	50	50	50	50	50
	SA	1,987	,416	2,017	,797	1,774	1,593	,756	,000	,283
	**MW**	**1,79**	**1,79**	**1,23**	**,88**	**,55**	**,41**	**,43**	**,24**	**,14**
**Pradl**	N	188	188	188	188	188	188	188	188	188
	SA	2,745	2,618	2,305	1,833	1,633	1,347	1,357	1,157	,860
	**MW**	**,43**	**,05**	**,10**	**,29**	**,10**	**,19**	**,00**	**,10**	**,71**
**Vill**	N	21	21	21	21	21	21	21	21	21
	SA	,870	,218	,436	1,102	,436	,873	,000	,436	2,101
	**MW**	**2,56**	**1,96**	**2,00**	**1,33**	**1,05**	**1,07**	**,93**	**,54**	**,76**
**Wilten**	N	156	156	156	156	156	156	156	156	156
	SA	2,771	2,809	2,969	2,397	2,225	2,042	2,029	1,567	1,928
	**MW**	**1,58**	**1,36**	**1,26**	**1,09**	**,83**	**,62**	**,53**	**,35**	**,29**
**Gesamt**	N	1031	1031	1031	1031	1031	1031	1031	1031	1031
	SA	2,519	2,475	2,337	2,232	3,609	1,725	1,523	1,348	1,238

1.3.2. Verkehrslärmbelästigung im Schlaf und Katastralgemeinden

Abbildung 184: Belästigung im Schlaf nach Verkehrslärmquelle und Katastralgemeinde im Mittelwertvergleich

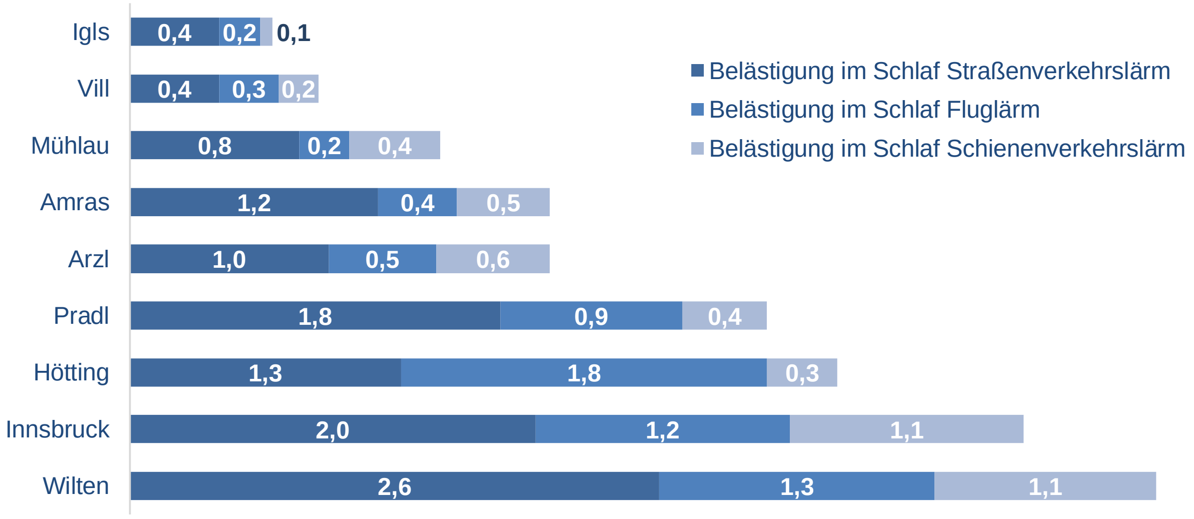

* Nach Belästigungsausmaß (Häufigkeit) aufsteigend sortiert

Das Ranking der Katastralgemeinden bezogen auf das Ausmaß der reinen Verkehrslärmbelästigung im Schlaf fällt beinahe ident dem Ranking der Lärmbelästigung aller Lärmquellen in der Nacht auS. Durch die höhere Fluglärmbelästigungsreaktion in Hötting auch in der Nacht, tauschen Hötting und Pradl die Plätze.

1.3.3. *Nicht verkehrsverursachte Lärmbelästigung im Schlaf und Katastralgemeinden*

Baustellenlärm, Erschütterungen, aber auch der Lärm aus Gewerbe und Industrie sind zum Teil ebenfalls verkehrsbedingt. Werden dennoch die Mittelwerte der nicht direkt verkehrsverursachten Lärmbelästigungsausmaße summiert dargestellt, ändert sich die Verteilung der Katastralgemeinden:

Abbildung 185: Belästigung im Schlaf nach nicht verkehrsverursachten Lärmquellen und Katastralgemeinde im Mittelwertvergleich

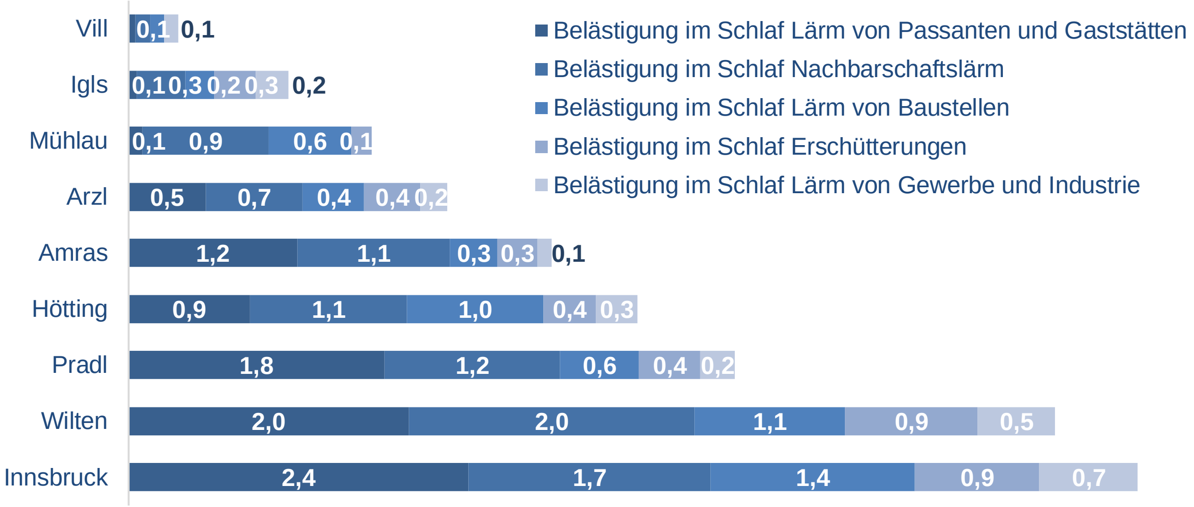

* Nach Belästigungsausmaß (Häufigkeit) aufsteigend sortiert

2. Belästigung im Schlaf durch Lärm insgesamt

Die Belästigung durch Lärm im Schlaf insgesamt wurde mittels einem eigenen Item zusätzlich abgefragt.

7,8% aller befragten Innsbruckerinnen und Innsbrucker schätzen sich selbst als vom Lärm insgesamt im Schlaf stark belästigt ein (*highly annoyed sleep*: gruppierte Antwortkategorien 8, 9 und 10 [=*belästigt / stört besonders stark*]), knapp ein Viertel antwortet in den mittleren Kategorien und über zwei Drittel aller Probandinnen und Probanden sehen sich selbst als nur gering im Schlaf lärmbelästigt an (gruppierte Antwortkategorien 0 [=*belästigt / stört überhaupt nicht*], 1 und 2), davon über 45% *überhaupt nicht belästigt*:

Abbildung 186: Belästigung durch Lärm im Schlaf insgesamt in Prozent

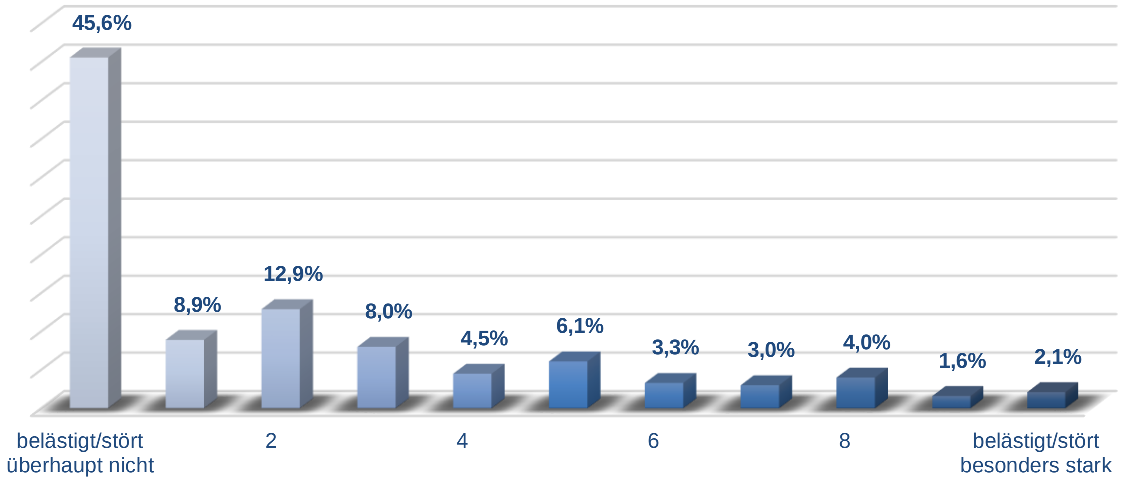

Im Mittel liegen die Antworten der befragten Probandinnen und Probanden bei 2,16 (23,8%).

Tabelle 143: Belästigung im Schlaf durch Lärm gesamt gruppiert

		Häufigkeit	Gültige Prozente	Kumulierte Prozente
Gültig	Gering belästigt	695	67,4	67,4
	Mittelgradig belästigt	256	24,8	92,2
	Stark belästigt	80	7,8	100,0
	Gesamt	1031	100,0	

2.1. Belästigung im Schlaf durch Lärm insgesamt u. Katastralgemeinden

Im Mittel die größte Störung im Schlaf durch Lärm insgesamt wird in Wilten und Innsbruck empfunden, gefolgt im Abstand von einer Antwortkategorie von Pradl, Amras und Hötting:

Abbildung 187: Belästigung im Schlaf durch Lärm insgesamt und Katastralgemeinden im Mittelwertvergleich

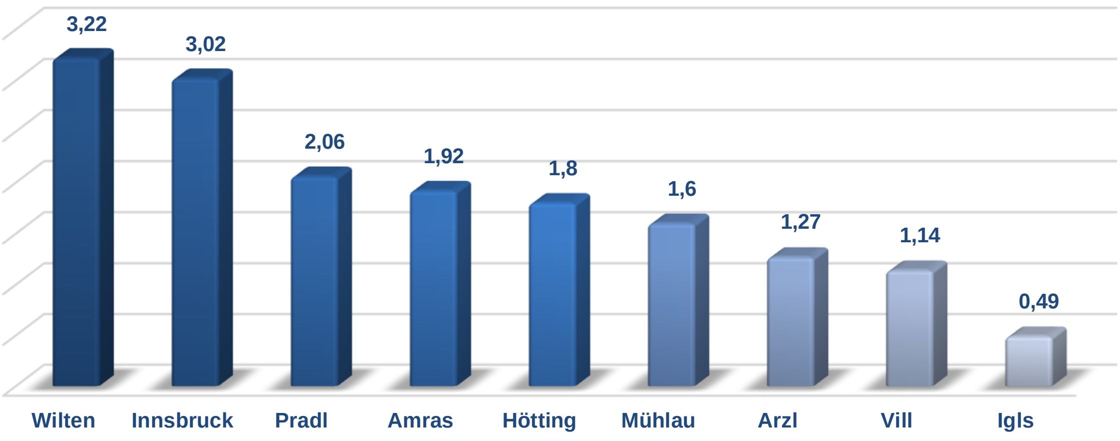

Die geringste Belästigung im Schlaf durch Lärm insgesamt im eigenen Wohnumfeld empfinden die Befragten in Igls, Vill und Arzl:

2.2. Belästigung im Schlaf durch Lärm insgesamt und ausgewählte Parameter

Frauen empfinden sich insgesamt deutlich stärker vom Lärm im Schlaf belästigt als Männer, insbesonders in der Extremgruppe der sich stark im Schlaf gestört Empfindenden (*highly annoyed*, Antwortkategorien 8, 9 und 10=*belästigt / stört besonders stark*):

Abbildung 188: Belästigung im Schlaf durch Lärm insgesamt nach Geschlecht (anteilig)

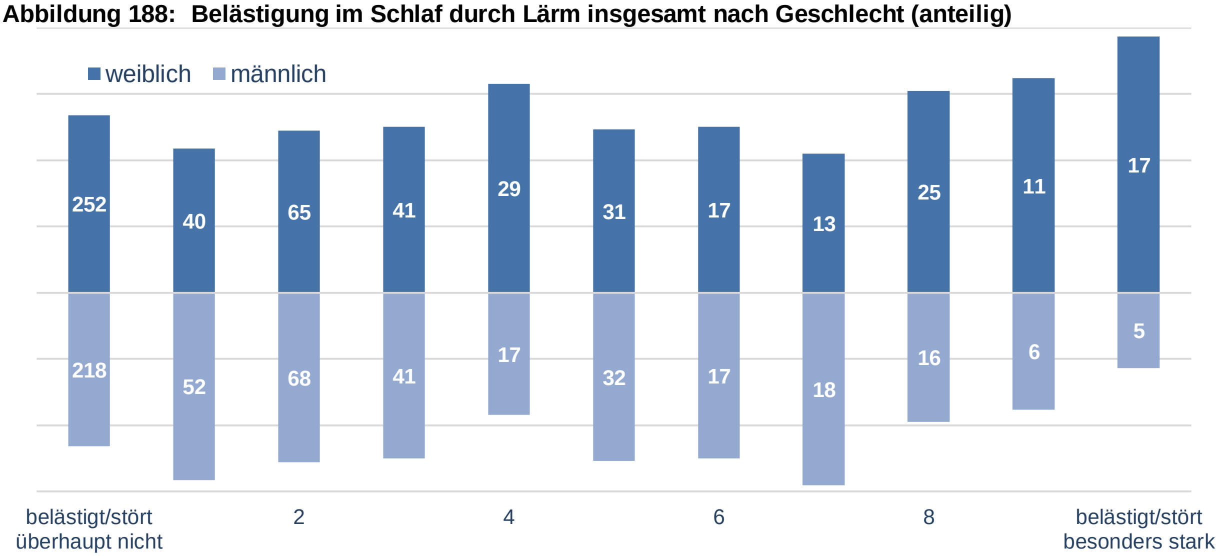

Ähnliches gilt für die mittlere Altersgruppe der 41- bis 60-Jährigen im Vergleich zu jüngeren und älteren Befragten. Der höchste erreichte Schulabschluss korrespondiert ebenfalls mit dem Belästigungsausmaß durch Lärm insgesamt im Schlaf: Akademikerinnen und Akademiker empfinden sich überdurchschnittlich, Pflichtschulabgängerinnen und –Abgänger hingegen unterdurchschnittlich stark belästigt.

Befragte, in deren Haushalt minderjährige Kinder leben, die zumeist bei geöffnetem oder gekippten Fenster schlafen, die in ihrem Wohnumfeld über eine lärmabgewandte Seite oder einen Innenhof verfügen, empfinden sich auch im Schlaf weniger vom Lärm gestört:

Tabelle 144: Belästigung im Schlaf durch Lärm insgesamt und Nachtschlaf im Mittelwertvergleich

Nachtschlaf gruppiert	Mittelwert	N	Standardabweichung	Standardfehler MW
Fenster nachts geöffnet/gekippt	1,34	821	,582	,020
Fenster nachts geschlossen	1,67	202	,741	,052
Insgesamt	1,40	1023	,630	,020

Der starke Bezug zum Verkehrslärm zeigt sich, wie schon für den 24h-Zeitraum, auch in der Nacht unter anderem in Zusammenschau mit der Frage nach baulichem Schutz vor demselben:

Abbildung 189: Belästigung im Schlaf durch Lärm insgesamt und baulicher Schutz vor Verkehrslärm im Extremgruppenvergleich in Prozent

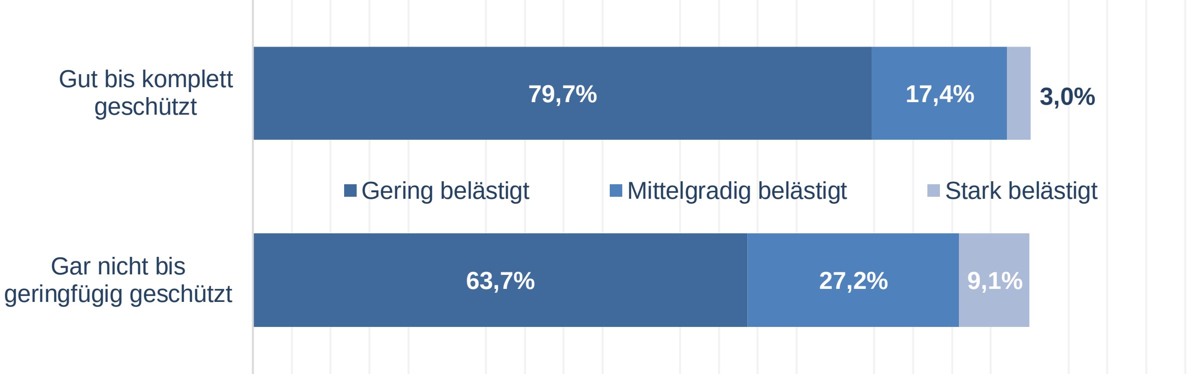

Erkennbar sich auch Beziehungen zwischen dem Ausmaß der Belästigung im Schlaf durch Lärm insgesamt und der Einschätzung und Zufriedenheit mit den eigenen Lebens- und Wohnbedingungen, den Bedingungen für das Aufwachsen von Kindern etc. Die Bezüge sind allerdings geringer ausgeprägt als für die Belästigung durch Lärm insgesamt im 24h-Zeitraum:

Abbildung 190: Güte der eigenen Wohnbedingunegn und Belästigung im Schlaf durch Lärm insgesamt im Mittelwertvergleich

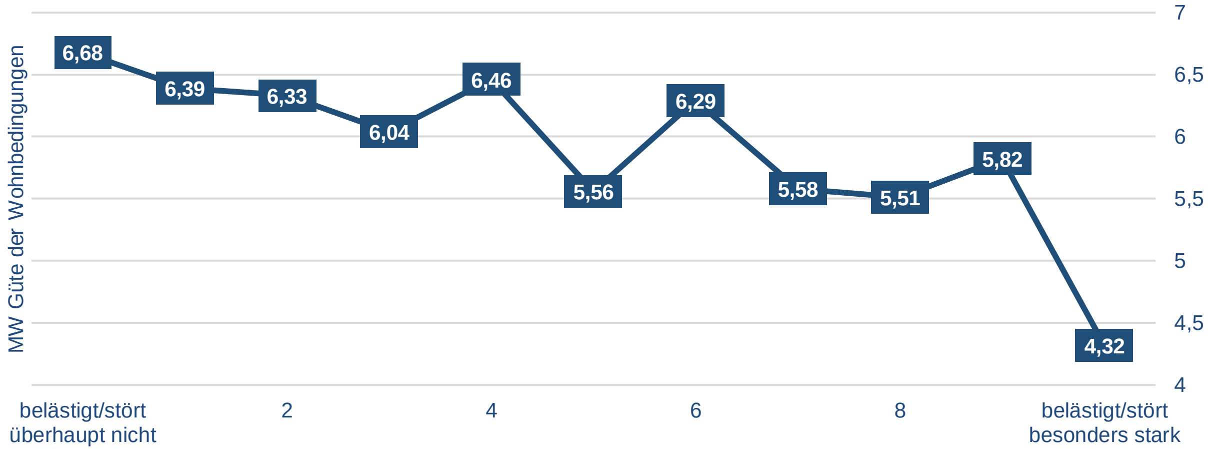

Dasselbe gilt auch für die Beziehungen zur Einschätzung der eigenen Lebensqualität und Lärmempfindlichkeit und einer ganzen Reihe anderer Parameter: Befragte Innsbruckerinnen und Innsbrucker, die sich im Schlaf lärmbelästigt fühlen,

- beurteilen das Lärmniveau in Innsbruck im Vergleich zu anderen österreichischen Städten schlechter,
- gehen häufiger davon aus, dass der Lärm insgesamt in den letzten Jahren zugenommen hat,
- schätzen die negativen Wirkungen von Lärm auf die Gesundheit stärker ein
- wenden häufiger Lärmadaptationsmechanismen an,
- sind wesentlich häufiger für (weitere) Maßnahmen zur Lärmreduktion,
- zeigen insgesamt in Bezug zu Beurteilungen der eigenen Wohn- und Lebensbedingungen eine deutlich negativere Grundhaltung.

Abbildung 191: Lebensqualität und Belästigung im Schlaf durch Lärm insgesamt im Mittelwertvergleich

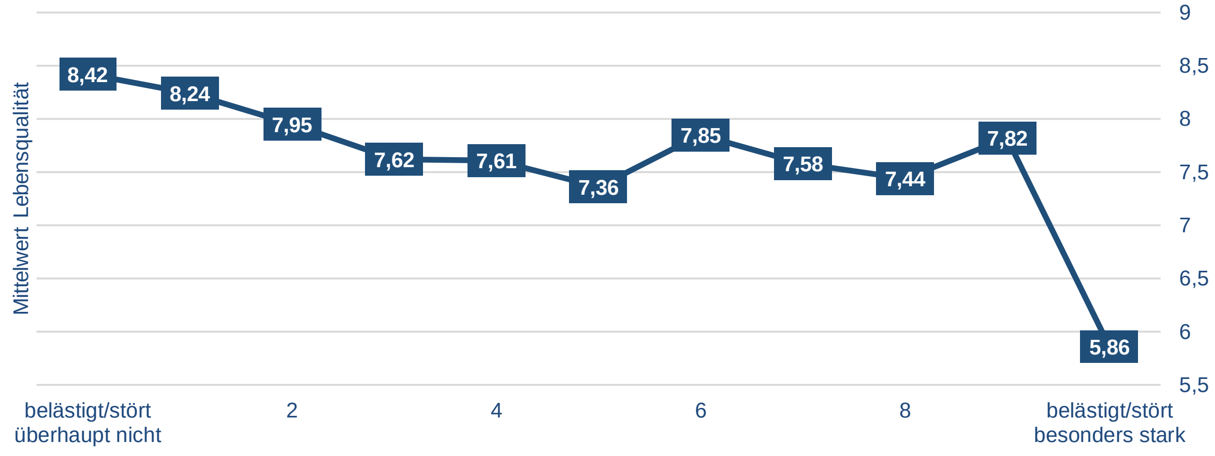

Auffällig zeigen sich, wie schon bei den anderen Fragen zur Lärmbelästigung nach Quelle, erneut diejenigen Befragten, die in der höchsten Antwortkategorie 10 (=*belästigt / stört besonders stark*) antworten.[14] Diese Personen zeigen – auch in den anderen Belästigungsauswertungen für den Nachtzeitraum unabhängig von der Lärmquelle und in Zusammenschau mit anderen Items – eine starke Tendenz zu negativeren Beurteilungen als die restlichen Befragten (vergleiche u.a. die oben dargestellten Abbildung zur Beurteilung der persönlichen Lebensqualität in Zusammenschau mit der Belästigung im Schlaf durch Lärm insgesamt.

Tabelle 145: Belästigung im Schlaf durch Lärm insgesamt und ausgewählte Parameter

		Belästigung im Schlaf Lärm gesamt gruppiert					
		Gering belästigt		Mittelgradig belästigt		Stark belästigt	
		Anzahl	Zeilen (%)	Anzahl	Zeilen (%)	Anzahl	Zeilen (%)
**Geschlecht**	weiblich	357	66,0%	131	24,2%	53	9,8%
	männlich	338	69,0%	125	25,5%	27	5,5%
**Altersgruppen**	18 bis 40 Jahre	294	66,8%	113	25,7%	33	7,5%
	41 bis 60 Jahre	195	61,7%	86	27,2%	35	11,1%
	über 60 Jahre	206	74,9%	57	20,7%	12	4,4%
**Bildungsschicht**	Primärer Schulabschluss	75	76,5%	17	17,3%	6	6,1%
	Sekundärer Schulabschluss	362	66,8%	144	26,6%	36	6,6%
	Tertiärer Schulabschluss	255	65,7%	95	24,5%	38	9,8%
**Haushalt mit/ohne minderjährige Kinder**	Haushalt ohne Kinder	504	66,1%	203	26,6%	55	7,2%
	Haushalt mit Kindern	191	71,0%	53	19,7%	25	9,3%
**Baulicher Schutz vor Verkehrslärm**	Keiner bis geringfügig	488	63,7%	208	27,2%	70	9,1%
	Gut bis komplett	188	79,7%	41	17,4%	7	3,0%
**Innenhof bzw. lärmabgewandte Seite**	Ja	483	68,9%	170	24,3%	48	6,8%
	Nein	196	63,6%	81	26,3%	31	10,1%
**Nachtschlaf**	Fenster geöffnet/gekippt	591	72,0%	183	22,3%	47	5,7%

[14] Für das Item *Belästigung im Schlaf durch Lärm insgesamt* handelt es sich dabei um 22 befragte Personen.

	Fenster geschlossen	99	49,0%	70	34,7%	33	16,3%
**Wohnbedingungen**	Nicht gut	53	63,1%	19	22,6%	12	14,3%
	Durchschnittlich	344	62,0%	163	29,4%	48	8,6%
	Sehr gut	296	75,9%	74	19,0%	20	5,1%
**Bedingungen für Kinder**	Nicht gut	20	42,6%	13	27,7%	14	29,8%
	Durchschnittlich	321	62,3%	152	29,5%	42	8,2%
	Sehr gut	338	75,6%	86	19,2%	23	5,1%
**Verbundenheit zu Innsbruck**	Nicht verbunden	28	50,9%	17	30,9%	10	18,2%
	Durchschnittlich	249	65,7%	98	25,9%	32	8,4%
	Sehr verbunden	416	69,9%	141	23,7%	38	6,4%
**Zufriedenheit eigene Wohnsituation**	Nicht zufrieden	12	42,9%	7	25,0%	9	32,1%
	Durchschnittlich	124	48,1%	98	38,0%	36	14,0%
	Sehr zufrieden	558	75,0%	151	20,3%	35	4,7%
**Lebensbedingungen (Summenscore)**	Nicht gut (*)	1	20,0%	2	40,0%	2	40,0%
	Durchschnittlich	414	61,8%	194	29,0%	62	9,3%
	Sehr gut	280	78,7%	60	16,9%	16	4,5%
**Lebensqualität**	Nicht gut (*)	5	50,0%	2	20,0%	3	30,0%
	Durchschnittlich	159	54,8%	98	33,8%	33	11,4%
	Sehr gut	527	72,7%	154	21,2%	44	6,1%
**Lärmempfindlichkeit**	Nicht lärmempfindlich	231	84,0%	35	12,7%	9	3,3%
	Durchschnittlich	357	61,1%	182	31,2%	45	7,7%
	Sehr lärmempfindlich	107	62,2%	39	22,7%	26	15,1%
**Summenscore Lärmcoping**	Coping ausgeprägt	75	31,0%	112	46,3%	55	22,7%
	Coping nicht ausgeprägt	620	78,7%	144	18,3%	24	3,0%
**Gefühlte Zunahme Lärmbelastung**	Ja	220	50,5%	160	36,7%	56	12,8%
	Nein	475	79,8%	96	16,1%	24	4,0%
**Gefühlte Abnahme Lärmbelastung**	Ja	44	75,9%	13	22,4%	1 (*)	1,7%
	Nein	651	66,9%	243	25,0%	79	8,1%
**Notwendigkeit (weiterer) Maßnahmen Lärm**	Maßnahmen notwendig	423	61,2%	197	28,5%	71	10,3%
	Keine Maßnahmen nötig	159	82,8%	29	15,1%	4 (*)	2,1%
**Negative Gesundheitswirkungen Lärm**	Gering	164	75,6%	46	21,2%	7	3,2%
	Stark	511	65,4%	197	25,2%	73	9,3%
**Lautheit Innsbruck im Vergleich zu anderen Städten Österreichs**	Ruhiger	226	77,4%	48	16,4%	18	6,2%
	Gleich	370	67,2%	143	26,0%	38	6,9%
	Lauter	87	50,0%	64	36,8%	23	13,2%
**Einschätzung eigener Lärmbeitrag**	Gering	529	68,3%	188	24,3%	58	7,5%
	Durchschnittlich	148	65,2%	62	27,3%	17	7,5%
	Groß	18	62,1%	6	20,7%	5	17,2%

(*) zu kleine Fallzahlen für solide Auswertungen

3. Belästigung im Schlaf durch Straßenverkehrslärm

Durch den Straßenverkehrslärm im Schlaf stark belästigt empfinden sich 5,4% aller Befragten, mehr als die Hälfte aller Befragten nimmt keine Belästigung im Schlaf wahr und insgesamt ordnen sich über drei Viertel aller Befragten der Gruppe der gering im Schlaf Lärmbelästigten zu. Im Mittel liegen die Antworten der befragten Probandinnen und Probanden bei 1,58 (17,4%) auf der 11-teiligen Antwortskala:

Abbildung 192: Belästigung durch Straßenverkehrslärm im Schlaf in Prozent

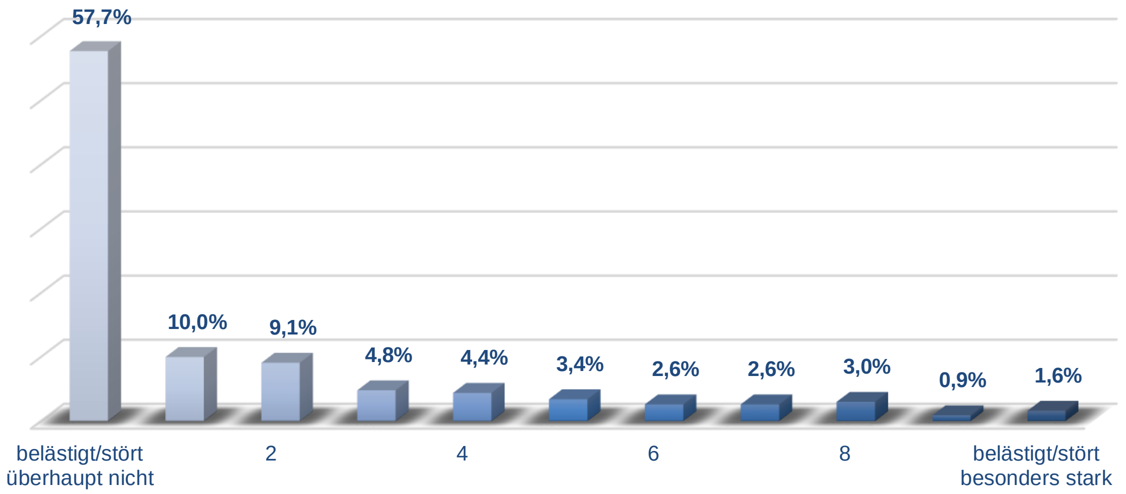

Tabelle 146: Belästigung im Schlaf durch Straßenverkehrslärm gruppiert

		Häufigkeit	Gültige Prozente	Kumulierte Prozente
Gültig	Gering belästigt	792	76,8	76,8
	Mittelgradig belästigt	183	17,7	94,6
	Stark belästigt	56	5,4	100,0
	Gesamt	1031	100,0	

3.1. Straßenlärmbelästigung im Schlaf und Straßenlärmbelastung

In der Zusammenschau der Belästigung durch Straßenverkehrslärm im Schlaf mit der gegebenen nächtlichen Straßenverkehrslärmbelastung zeigt sich ein dem 24h-Zeitraum sehr vergleichbares, etwas kontinuierlicheres Ansteigen des Belästigungsausmaßes alS. Ein „Schwellenwert“ ab welchem ein stärkeres Ansteigen der Belästigungsreaktion auftritt, ist im Gegensatz zum 24h-Zeitraum nicht eindeutig festzumachen:

Abbildung 193: Straßenlärmbelästigung im Schlaf und nächtliche Straßenlärmbelastung L_n in 10dB-Schritten im Mittelwertvergleich

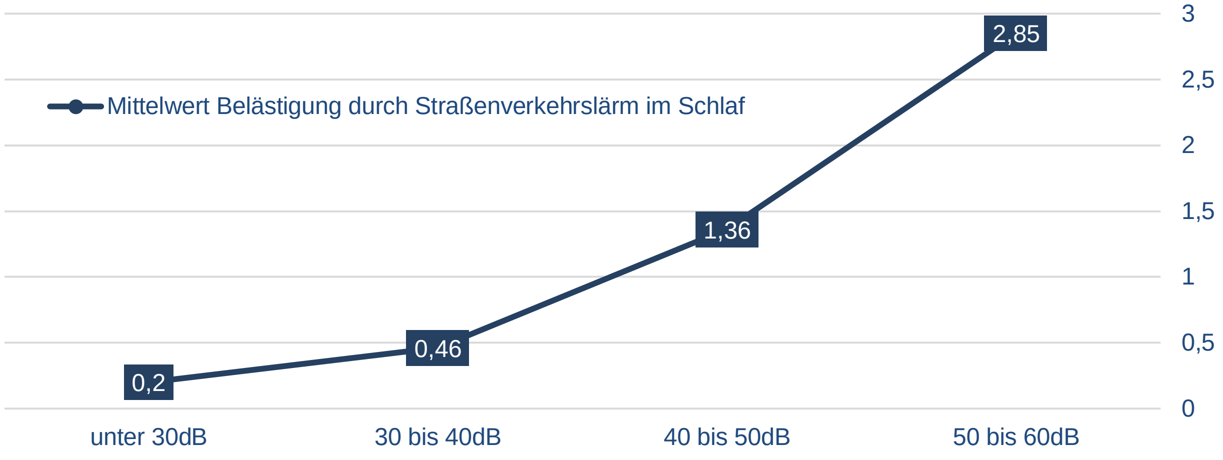

Anm.: Die Darstellung zeigt nachvollziehbare Zusammenhänge auf, enthält jedoch keine Prüfungen auf Kovariablen und andere Prädiktoren und ist somit nicht den Dosis-Wirkungskurven aus der gängigen Fachliteratur gleichzusetzen.

3.2. Straßenlärmbelästigung im Schlaf und Katastralgemeinden

Das größte Ausmaß an Belästigung durch Straßenverkehrslärm im Schlaf wird mit einigem Abstand in Wilten empfunden, gefolgt von Innsbruck, Pradl und wiederum mit einigem Abstand Hötting. Die geringste Störung durch Straßenverkehrslärm im Schlaf im eigenen Wohnumfeld empfinden die Befragten in Vill und Igls:

Abbildung 194: Straßenlärmbelästigung im Schlaf und Katastralgemeinden im Mittelwertvergleich

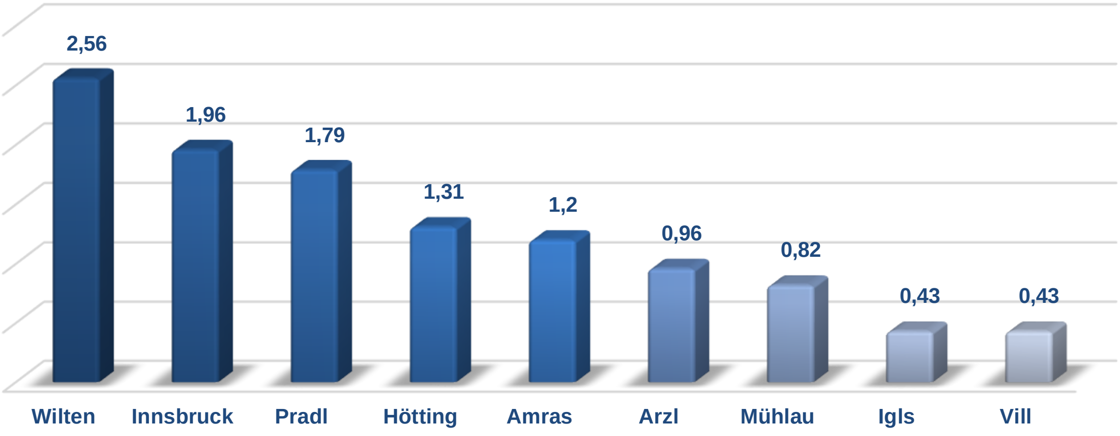

3.3. Straßenverkehrslärmbelästigung im Schlaf und andere Parameter

Die Trends, welche sich aus der Kombination der Belästigung im Schlaf durch Lärm insgesamt mit anderen Items ergeben (siehe das vorhergehende Kapitel), lassen sich auf die Belästigung im Schlaf durch Straßenverkehrslärm weitestgehend umlegen. Das gilt für die demographischen Parameter ebenso wie für die Einschätzung der Wohn- und Lebensbedingungen, das Ausmaß der Lärmempfind-

lichkeit, Nachtschlaf bei geöffnetem oder geschlossenen Fenstern, oder die vorhandene Abschirmung gegenüber Verkehrslärm usw.:

Abbildung 195: Belästigung im Schlaf durch Straßenverkehrslärm und baulicher Schutz vor Verkehrslärm im Extremgruppenvergleich in Prozent

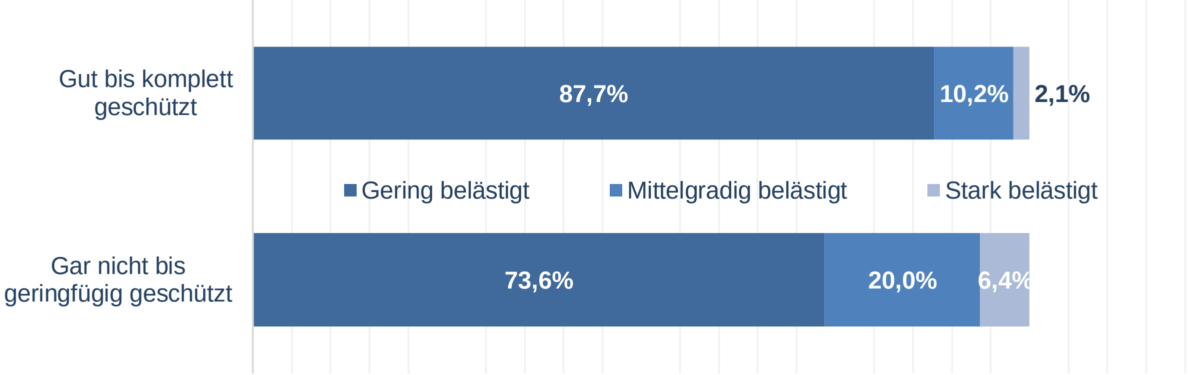

Auch das Antwortverhalten zur Einschätzung des Lärmniveaus in Innsbruck im Vergleich zu anderen Städten Österreichs verändert sich bei zunehmender Belästigung durch Straßenverkehrslärm in der Nacht (gilt ebenso für andere Verkehrslärmquellen und auch für den 24h-Zeitraum):

Abbildung 196: Einschätzung des Lärmniveaus in Innsbruck im Vergleich zu anderen Städten Österreichs und Belästigung im Schlaf durch Straßenverkehrslärm im Mittelwertvergleich

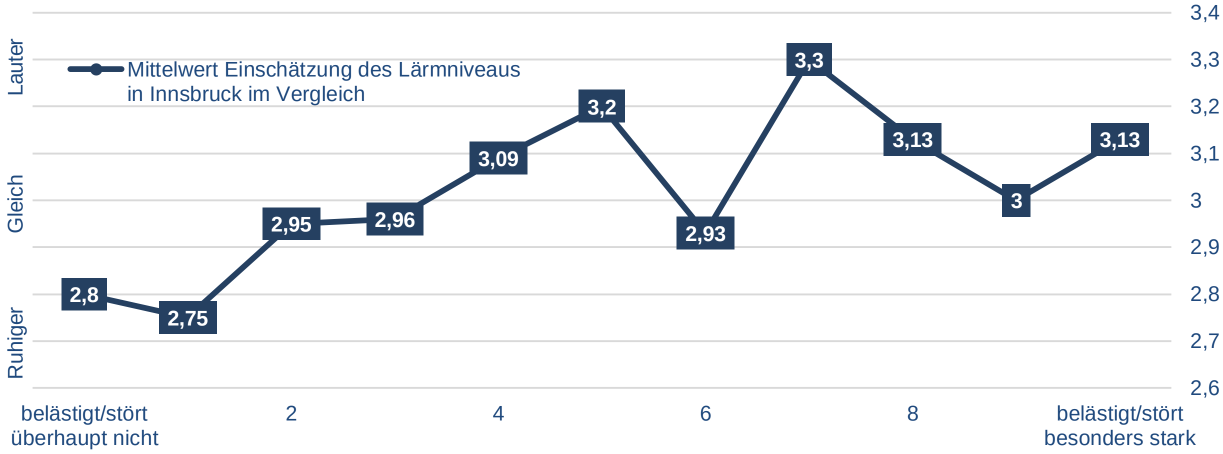

Spannend sind Zusammenhänge einzelner Fragestellung mit der Belästigung im Schlaf durch Straßenverkehrslärm (wie auch für andere Lärmquellen) in Abhängigkeit von der vorhandenen nächtlichen Straßenverkehrslärmbelastung.

So wird etwa die (beabsichtigt) diffus gestellte Frage nach der Einschätzung der Güte der Sicherheit in Innsbruck von stärker belasteten und belästigten Befragten deutlich schlechter eingestuft, als von weniger Lärmbelasteten und –Belästigten:[15]

[15] Anm.: Diese Beziehungen zeigen sich auch bei anderen Lärmbelästigungsquellen.

Abbildung 197: Einschätzung der Sicherheit und Belästigung im Schlaf durch Straßenverkehrslärm nach nächtlicher Straßenverkehrslärmbelastung L_n in 10dB-Schritten im Mittelwertvergleich

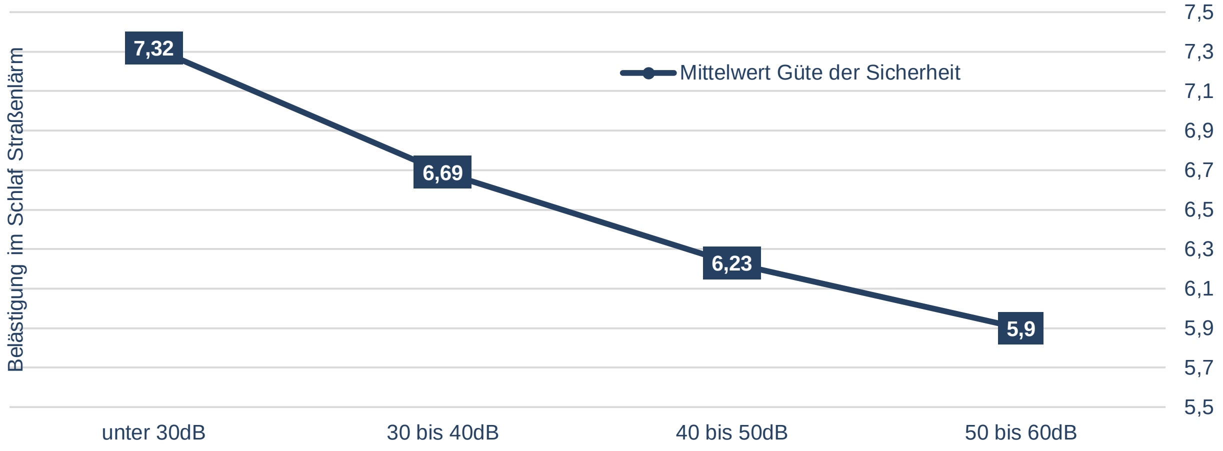

Tabelle 147: Belästigung im Schlaf durch Straßenverkehrslärm und ausgewählte Parameter

		Belästigung im Schlaf Straßenverkehrslärm gruppiert					
		Gering belästigt		Mittelgradig belästigt		Stark belästigt	
		Anzahl	Zeilen (%)	Anzahl	Zeilen (%)	Anzahl	Zeilen (%)
**Geschlecht**	weiblich	409	75,6%	97	17,9%	35	6,5%
	männlich	383	78,2%	86	17,6%	21	4,3%
**Altersgruppen**	18 bis 40 Jahre	337	76,6%	79	18,0%	24	5,5%
	41 bis 60 Jahre	236	74,7%	58	18,4%	22	7,0%
	über 60 Jahre	219	79,6%	46	16,7%	10	3,6%
**Bildungsschicht**	Primärer Schulabschluss	81	82,7%	13	13,3%	4 (*)	4,1%
	Sekundärer Schulabschluss	413	76,2%	101	18,6%	28	5,2%
	Tertiärer Schulabschluss	295	76,0%	69	17,8%	24	6,2%
**Haushalt mit/ohne minderjährige Kinder**	Haushalt ohne Kinder	587	77,0%	136	17,8%	39	5,1%
	Haushalt mit Kindern	205	76,2%	47	17,5%	17	6,3%
**Baulicher Schutz vor Verkehrslärm**	Keiner bis geringfügig	564	73,6%	153	20,0%	49	6,4%
	Gut bis komplett	207	87,7%	24	10,2%	5	2,1%
**Innenhof bzw. lärm-abgewandte Seite**	Ja	546	77,9%	123	17,5%	32	4,6%
	Nein	229	74,4%	55	17,9%	24	7,8%
**Nachtschlaf**	Fenster geöffnet/gekippt	658	80,1%	132	16,1%	31	3,8%
	Fenster geschlossen	128	63,4%	49	24,3%	25	12,4%
**Wohnbedingungen**	Nicht gut	67	79,8%	9	10,7%	8	9,5%
	Durchschnittlich	396	71,4%	123	22,2%	36	6,5%
	Sehr gut	327	83,8%	51	13,1%	12	3,1%
**Bedingungen für Kinder**	Nicht gut	30	63,8%	9	19,1%	8	17,0%
	Durchschnittlich	367	71,3%	112	21,7%	36	7,0%
	Sehr gut	376	84,1%	59	13,2%	12	2,7%
**Verbundenheit zu Innsbruck**	Nicht verbunden	37	67,3%	13	23,6%	5	9,1%
	Durchschnittlich	297	78,4%	67	17,7%	15	4,0%
	Sehr verbunden	456	76,6%	103	17,3%	36	6,1%

**Zufriedenheit eigene Wohnsituation**	Nicht zufrieden	19	67,9%	5	17,9%	4 (*)	14,3%
	Durchschnittlich	163	63,2%	69	26,7%	26	10,1%
	Sehr zufrieden	610	82,0%	108	14,5%	26	3,5%
**Lebensbedingungen (Summenscore)**	Nicht gut (*)	2	40,0%	2	40,0%	1	20,0%
	Durchschnittlich	481	71,8%	144	21,5%	45	6,7%
	Sehr gut	309	86,8%	37	10,4%	10	2,8%
**Lebensqualität**	Nicht gut (*)	7	70,0%	2	20,0%	1	10,0%
	Durchschnittlich	199	68,6%	68	23,4%	23	7,9%
	Sehr gut	582	80,3%	112	15,4%	31	4,3%
**Lärmempfindlichkeit**	Nicht lärmempfindlich	237	86,2%	33	12,0%	5	1,8%
	Durchschnittlich	425	72,8%	124	21,2%	35	6,0%
	Sehr lärmempfindlich	130	75,6%	26	15,1%	16	9,3%
**Summenscore Lärmcoping**	Coping ausgeprägt	130	53,7%	76	31,4%	36	14,9%
	Coping nicht ausgeprägt	662	84,0%	107	13,6%	19	2,4%
**Gefühlte Zunahme Lärmbelastung**	Ja	284	65,1%	114	26,1%	38	8,7%
	Nein	508	85,4%	69	11,6%	18	3,0%
**Gefühlte Abnahme Lärmbelastung**	Ja	45	77,6%	11	19,0%	2 (*)	3,4%
	Nein	747	76,8%	172	17,7%	54	5,5%
**Notwendigkeit (weite-rer) Maßnahmen Lärm**	Maßnahmen notwendig	495	71,6%	143	20,7%	53	7,7%
	Keine Maßnahmen nötig	174	90,6%	18	9,4%	0 (*)	0,0%
**Negative Gesund-heitswirkungen Lärm**	Gering	185	85,3%	26	12,0%	6	2,8%
	Stark	584	74,8%	149	19,1%	48	6,1%
**Lautheit Innsbruck im Vergleich zu anderen Städten Österreichs**	Ruhiger	243	83,2%	40	13,7%	9	3,1%
	Gleich	424	77,0%	92	16,7%	35	6,4%
	Lauter	112	64,4%	50	28,7%	12	6,9%
**Einschätzung eigener Lärmbeitrag**	Gering	605	78,1%	128	16,5%	42	5,4%
	Durchschnittlich	166	73,1%	51	22,5%	10	4,4%
	Groß	21	72,4%	4 (*)	13,8%	4 (*)	13,8%

(*) zu kleine Fallzahlen für solide Auswertungen

4. Belästigung im Schlaf durch Lärm von Passanten und Gaststätten

Die wahrgenommene Belästigung im Schlaf durch den Lärm verursacht von Passanten und Gaststätten ist in ihren Ausprägungen nahezu deckungsgleich mit jener durch den Straßenverkehrslärm.

Im Mittel liegen die Antworten der befragten Innsbruckerinnen und Innsbrucker jedoch unterhalb der Werte vom Straßenverkehrslärm bei 1,36 (15,0%):

Abbildung 198: Belästigung durch Lärm von Passanten und Gaststätten im Schlaf in Prozent

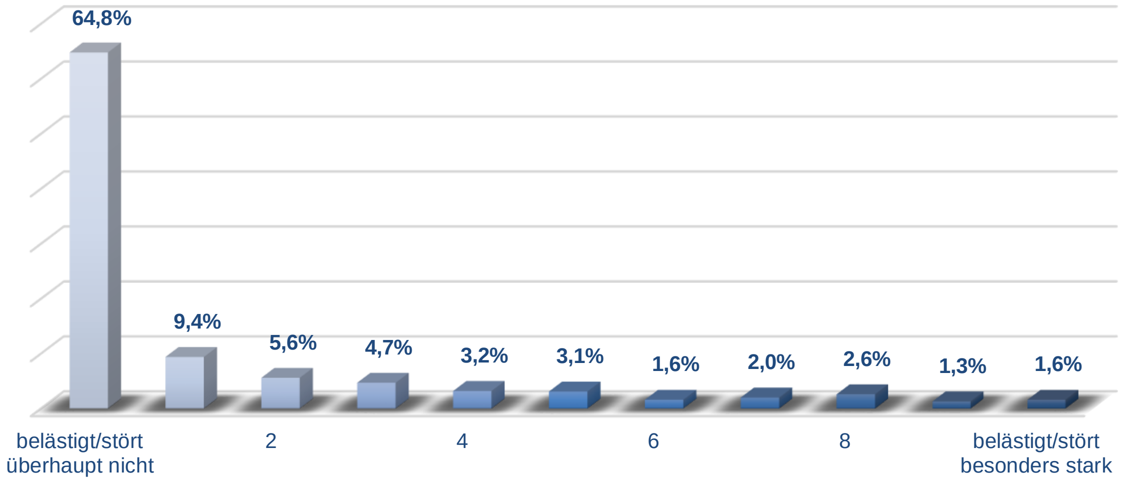

Tabelle 148: Belästigung im Schlaf durch Lärm von Passanten und Gaststätten gruppiert

		Häufigkeit	Gültige Prozente	Kumulierte Prozente
Gültig	Gering belästigt	823	79,8	79,8
	Mittelgradig belästigt	151	14,6	94,5
	Stark belästigt	57	5,5	100,0
	Gesamt	1031	100,0	

4.1. Lärmbelästigung im Schlaf durch Passanten und Gaststätten und Katastralgemeinden

Die Belästigung im Schlaf durch den Lärm von Passanten und Gaststätten ist in den inneren Stadtbezirken naturgemäß größer und in Vill, Igls und Mühlau de facto nicht existent:

Abbildung 199: Lärmbelästigung im Schlaf durch Passanten und Gaststätten und Katastralgemeinden im Mittelwertvergleich

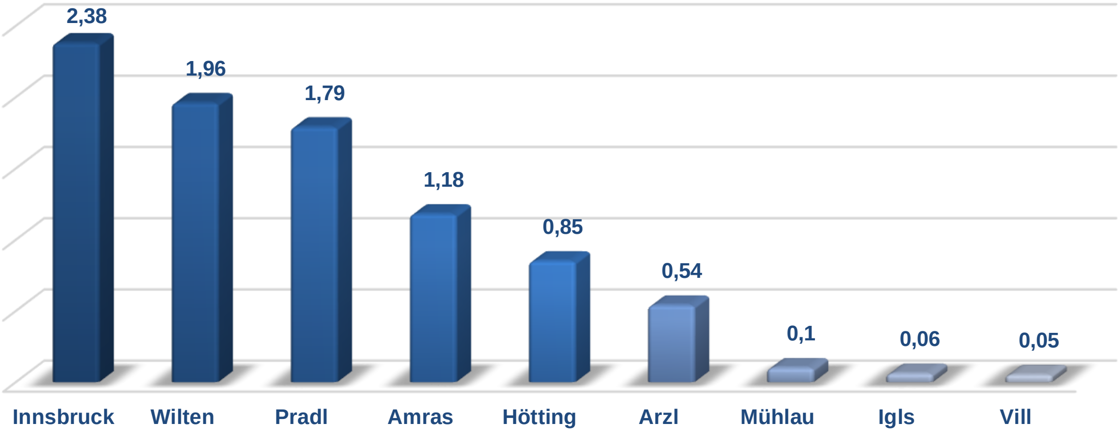

4.2. Lärmbelästigung im Schlaf durch Passanten und Gaststätten und ausgewählte Parameter

Auch für die Belästigung im Schlaf durch den Lärm von Passanten und Gaststätten zeigen sich sehr ähnliche Zusammenhänge zu anderen Items, wie schon im Falle der Auswertungen zur Belästigung im Schlaf durch Lärm insgesamt oder auch durch den nächtlichen Straßenverkehrslärm.

Die Unterschiede zwischen den Extremgruppen und den Mittelwertvergleichen entsprechen in ihren Ausmaßen ebenso den Analysen zur Belästigung im Schlaf durch Verkehrslärm oder den Lärm insgesamt.

Abbildung 200: Belästigung im Schlaf durch Lärm von Passanten und Gaststätten und Verbundenheit zur Stadt Innsbruck im Extremgruppenvergleich in Prozent

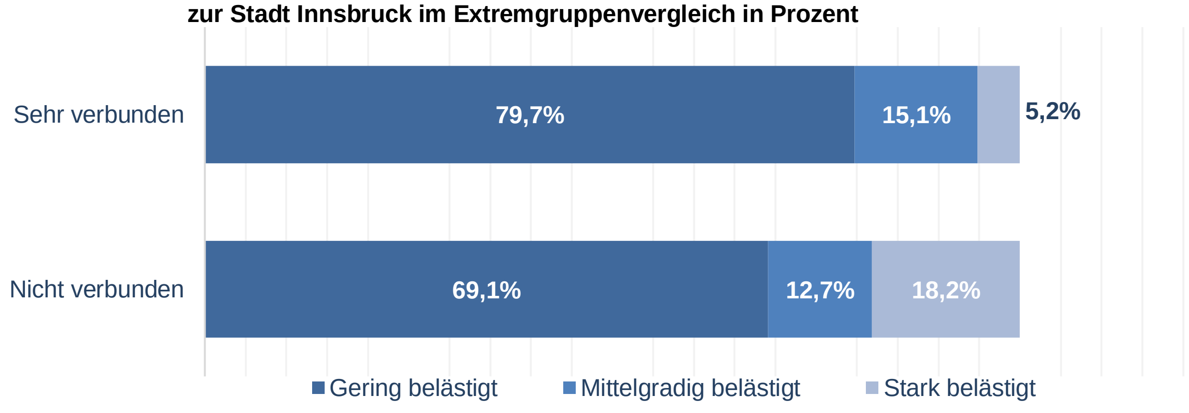

Im Folgenden einige ausgewählte Parameter, jeweils in 3 Belästigungsgruppen zusammengefasst, in Zusammenschau mit der Belästigung im Schlaf durch den Lärm von Passanten und Gaststätten:

Tabelle 149: Belästigung im Schlaf durch Passanten und Gaststätten und ausgewählte Parameter

		Belästigung im Schlaf Lärm von Passanten und Gaststätten					
		Gering belästigt		Mittelgradig belästigt		Stark belästigt	
		Anzahl	Zeilen (%)	Anzahl	Zeilen (%)	Anzahl	Zeilen (%)
**Geschlecht**	weiblich	422	78,0%	85	15,7%	34	6,3%
	männlich	401	81,8%	66	13,5%	23	4,7%
**Altersgruppen**	18 bis 40 Jahre	346	78,6%	65	14,8%	29	6,6%
	41 bis 60 Jahre	249	78,8%	44	13,9%	23	7,3%
	über 60 Jahre	228	82,9%	42	15,3%	5	1,8%
**Bildungsschicht**	Primärer Schulabschluss	82	83,7%	11	11,2%	5	5,1%
	Sekundärer Schulabschluss	423	78,0%	91	16,8%	28	5,2%
	Tertiärer Schulabschluss	315	81,2%	49	12,6%	24	6,2%
**Haushalt mit/ohne minderjährige Kinder**	Haushalt ohne Kinder	600	78,7%	116	15,2%	46	6,0%
	Haushalt mit Kindern	223	82,9%	35	13,0%	11	4,1%
**Baulicher Schutz vor Verkehrslärm**	Keiner bis geringfügig	598	78,1%	118	15,4%	50	6,5%
	Gut bis komplett	202	85,6%	27	11,4%	7	3,0%
**Innenhof bzw. lärmabgewandte Seite**	Ja	547	78,0%	116	16,5%	38	5,4%
	Nein	256	83,1%	33	10,7%	19	6,2%
**Nachtschlaf**	Fenster geöffnet/gekippt	673	82,0%	113	13,8%	35	4,3%

	Fenster geschlossen	143	70,8%	37	18,3%	22	10,9%
**Wohnbedingungen**	Nicht gut	58	69,0%	13	15,5%	13	15,5%
	Durchschnittlich	425	76,6%	95	17,1%	35	6,3%
	Sehr gut	338	86,7%	43	11,0%	9	2,3%
**Bedingungen für Kinder**	Nicht gut	24	51,1%	15	31,9%	8	17,0%
	Durchschnittlich	391	75,9%	89	17,3%	35	6,8%
	Sehr gut	389	87,0%	45	10,1%	13	2,9%
**Verbundenheit zu Innsbruck**	Nicht verbunden	38	69,1%	7	12,7%	10	18,2%
	Durchschnittlich	309	81,5%	54	14,2%	16	4,2%
	Sehr verbunden	474	79,7%	90	15,1%	31	5,2%
**Zufriedenheit eigene Wohnsituation**	Nicht zufrieden	17	60,7%	7	25,0%	4 (*)	14,3%
	Durchschnittlich	170	65,9%	62	24,0%	26	10,1%
	Sehr zufrieden	635	85,3%	82	11,0%	27	3,6%
**Lebensbedingungen (Summenscore)**	Nicht gut (*)	2	40,0%	1	20,0%	2	40,0%
	Durchschnittlich	504	75,2%	118	17,6%	48	7,2%
	Sehr gut	317	89,0%	32	9,0%	7	2,0%
**Lebensqualität**	Nicht gut (*)	6	60,0%	0	0,0%	4	40,0%
	Durchschnittlich	209	72,1%	63	21,7%	18	6,2%
	Sehr gut	604	83,3%	87	12,0%	34	4,7%
**Lärmempfindlichkeit**	Nicht lärmempfindlich	247	89,8%	21	7,6%	7	2,5%
	Durchschnittlich	448	76,7%	104	17,8%	32	5,5%
	Sehr lärmempfindlich	128	74,4%	26	15,1%	18	10,5%
**Summenscore Lärmcoping**	Coping ausgeprägt	137	56,6%	70	28,9%	35	14,5%
	Coping nicht ausgeprägt	685	86,9%	81	10,3%	22	2,8%
**Gefühlte Zunahme Lärmbelastung**	Ja	309	70,9%	92	21,1%	35	8,0%
	Nein	514	86,4%	59	9,9%	22	3,7%
**Gefühlte Abnahme Lärmbelastung**	Ja	47	81,0%	9	15,5%	2 (*)	3,4%
	Nein	776	79,8%	142	14,6%	55	5,7%
**Notwendigkeit (weiterer) Maßnahmen Lärm**	Maßnahmen notwendig	527	76,3%	112	16,2%	52	7,5%
	Keine Maßnahmen nötig	174	90,6%	16	8,3%	2 (*)	1,0%
**Negative Gesundheitswirkungen Lärm**	Gering	190	87,6%	22	10,1%	5	2,3%
	Stark	606	77,6%	123	15,7%	52	6,7%
**Lautheit Innsbruck im Vergleich zu anderen Städten Österreichs**	Ruhiger	253	86,6%	29	9,9%	10	3,4%
	Gleich	440	79,9%	81	14,7%	30	5,4%
	Lauter	118	67,8%	40	23,0%	16	9,2%
**Einschätzung eigener Lärmbeitrag**	Gering	626	80,8%	111	14,3%	38	4,9%
	Durchschnittlich	175	77,1%	36	15,9%	16	7,0%
	Groß	22	75,9%	4 (*)	13,8%	3 (*)	10,3%

(*) zu kleine Fallzahlen für solide Auswertungen

5. Belästigung im Schlaf durch Nachbarschaftslärm

Die Belästigung im Schlaf durch Nachbarschaftslärm steht auf Platz drei der am meisten störenden Lärmquellen. 4,3% aller Probandinnen und Probanden schätzen sich als stark vom Nachbarschaftslärm im Schlaf belästigt ein, über 80% als gering belästigt, davon knapp zwei Drittel *überhaupt nicht gestört*:

Abbildung 201: Belästigung durch Nachbarschaftslärm im Schlaf in Prozent

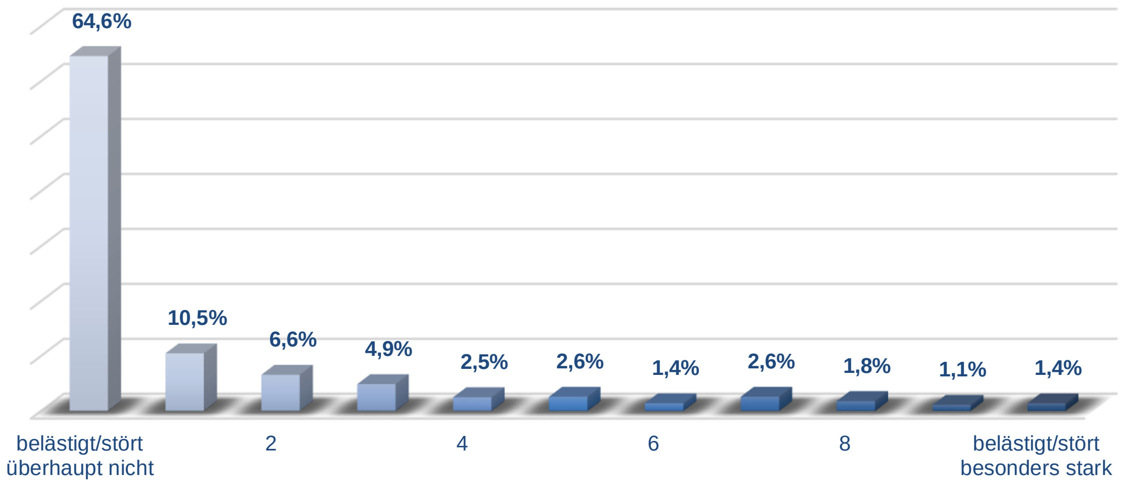

Im Mittel liegen die Antworten der Befragten bei 1,26 (13,9%).

Tabelle 150: Belästigung im Schlaf durch Nachbarschaftslärm gruppiert

		Häufigkeit	Gültige Prozente	Kumulierte Prozente
Gültig	Gering belästigt	842	81,7	81,7
	Mittelgradig belästigt	145	14,1	95,7
	Stark belästigt	44	4,3	100,0
	Gesamt	1031	100,0	

5.1. Nachbarschaftslärmbelästigung im Schlaf und Katastralgemeinden

Die Belästigung durch Nachbarschaftslärm im Schlaf nach Katastralgemeinden unterscheidet sich vom 24h-Zeitraum: Liegen Innsbruck, Wilten und Pradl im Antwortverhalten der Befragten im 24h-Zeitraum nahezu gleichauf, so sehen sich die befragten Wiltenerinnen und Wiltener im Nachtzeitraum stärker vom Nachbarschaftslärm im Schlaf gestört als die Einwohnerinnen und Einwohner der Katastralgemeinde Innsbruck und diese wiederum stärker als die Pradlerinnen und Pradler. Am wenigsten vom Nachbarschaftslärm im Schlaf gestört empfinden sich die Befragten in Vill und Igls:

Abbildung 202: Nachbarschaftslärmbelästigung im Schlaf und Katastralgemeinden im Mittelwertvergleich

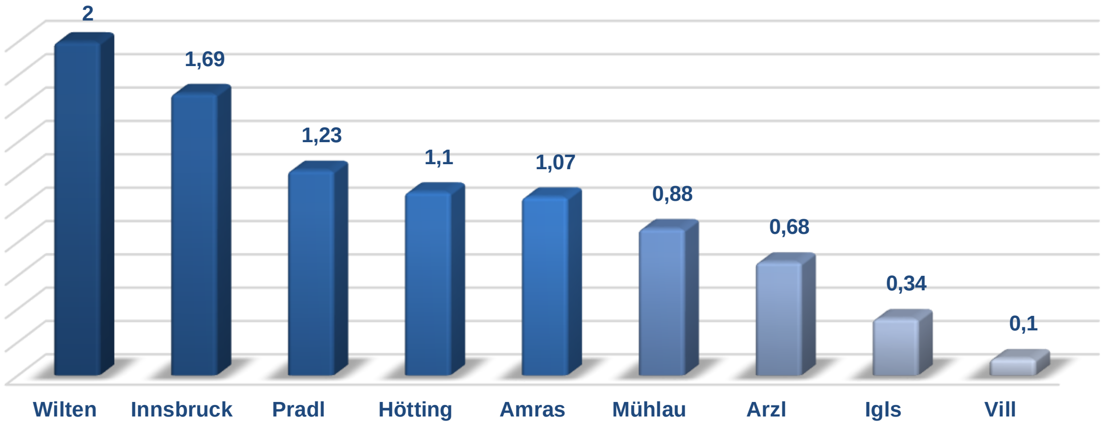

5.2. Nachbarschaftslärmbelästigung im Schlaf und einzelne Parameter

Auch bei der Belästigung im Schlaf durch Nachbarschaftslärm werden dieselben grundsätzlichen Zusammenhänge wie schon bei den vorhergehenden Auswertungen zu anderen Lärmbelästigungsquellen sichtbar. Die Störung durch Nachbarschaftslärm in der Nacht zeigt jedoch vergleichsweise stark ausgeprägte Beziehungen zu den Fragen zu den eigenen Wohn- und Lebensbedingungen. Sich stark vom Nachbarschaftslärm im Schlaf belästigte Innsbruckerinnen und Innsbrucker beurteilen diese deutlich schlechter als gar nicht oder nur gering belästigte Befragte:

Abbildung 203: Zufriedenheit mit der eigenen Wohnsituation insgesamt und Belästigung im Schlaf durch Nachbarschaftslärm im Mittelwertvergleich

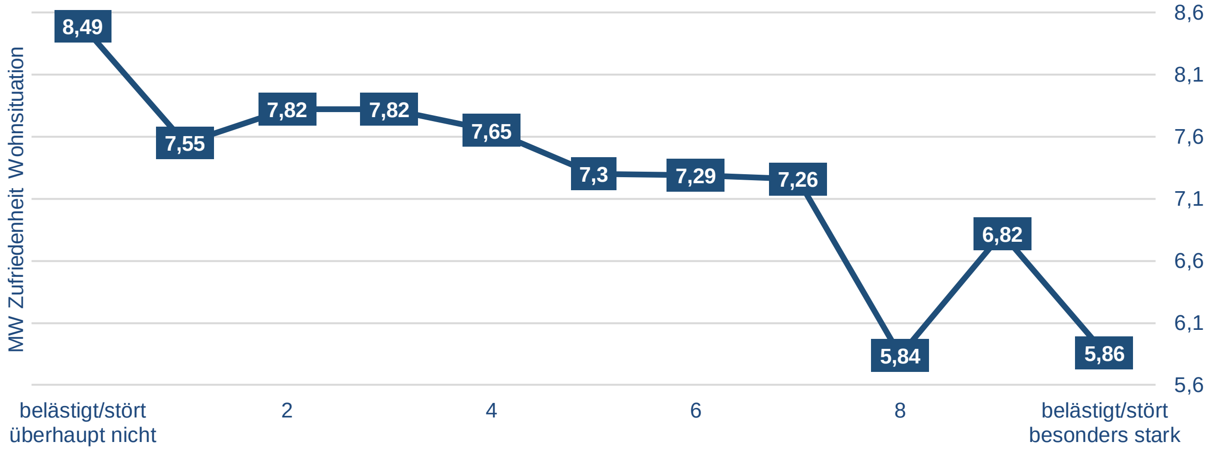

Im Folgenden einige ausgewählte Parameter, jeweils in 3 Belästigungsgruppen zusammengefasst, in Zusammenschau mit der Belästigung im Schlaf durch Nachbarschaftslärm:

Tabelle 151: Belästigung im Schlaf durch Nachbarschaftslärm und ausgewählte Parameter

	Belästigung im Schlaf Nachbarschaftslärm gruppiert					
	Gering belästigt		Mittelgradig belästigt		Stark belästigt	
	Anzahl	Zeilen (%)	Anzahl	Zeilen (%)	Anzahl	Zeilen (%)

**Geschlecht**	weiblich	435	80,4%	79	14,6%	27	5,0%
	männlich	407	83,1%	66	13,5%	17	3,5%
**Altersgruppen**	18 bis 40 Jahre	357	81,1%	64	14,5%	19	4,3%
	41 bis 60 Jahre	245	77,5%	50	15,8%	21	6,6%
	über 60 Jahre	240	87,3%	31	11,3%	4 (*)	1,5%
**Bildungsschicht**	Primärer Schulabschluss	86	87,8%	9	9,2%	3 (*)	3,1%
	Sekundärer Schulabschluss	443	81,7%	79	14,6%	20	3,7%
	Tertiärer Schulabschluss	310	79,9%	57	14,7%	21	5,4%
**Haushalt mit/ohne minderjährige Kinder**	Haushalt ohne Kinder	616	80,8%	114	15,0%	32	4,2%
	Haushalt mit Kindern	226	84,0%	31	11,5%	12	4,5%
**Nachtschlaf**	Fenster geöffnet/gekippt	685	83,4%	114	13,9%	22	2,7%
	Fenster geschlossen	151	74,8%	29	14,4%	22	10,9%
**Wohnbedingungen**	Nicht gut	58	69,0%	17	20,2%	9	10,7%
	Durchschnittlich	440	79,3%	89	16,0%	26	4,7%
	Sehr gut	342	87,7%	39	10,0%	9	2,3%
**Verbundenheit zu Innsbruck**	Nicht verbunden	36	65,5%	10	18,2%	9	16,4%
	Durchschnittlich	300	79,2%	67	17,7%	12	3,2%
	Sehr verbunden	504	84,7%	68	11,4%	23	3,9%
**Zufriedenheit eigene Wohnsituation**	Nicht zufrieden	17	60,7%	5	17,9%	6	21,4%
	Durchschnittlich	188	72,9%	53	20,5%	17	6,6%
	Sehr zufrieden	636	85,5%	87	11,7%	21	2,8%
**Lebensbedingungen (Summenscore)**	Nicht gut (*)	1	20,0%	3	60,0%	1	20,0%
	Durchschnittlich	517	77,2%	115	17,2%	38	5,7%
	Sehr gut	324	91,0%	27	7,6%	5	1,4%
**Lebensqualität**	Nicht gut (*)	4	40,0%	3	30,0%	3	30,0%
	Durchschnittlich	214	73,8%	55	19,0%	21	7,2%
	Sehr gut	620	85,5%	85	11,7%	20	2,8%
**Lärmempfindlichkeit**	Nicht lärmempfindlich	249	90,5%	19	6,9%	7	2,5%
	Durchschnittlich	465	79,6%	96	16,4%	23	3,9%
	Sehr lärmempfindlich	128	74,4%	30	17,4%	14	8,1%
**Summenscore Lärmcoping**	Coping ausgeprägt	149	61,6%	64	26,4%	29	12,0%
	Coping nicht ausgeprägt	693	87,9%	81	10,3%	14	1,8%
**Notwendigkeit (weitere-rer) Maßnahmen Lärm**	Maßnahmen notwendig	545	78,9%	111	16,1%	35	5,1%
	Keine Maßnahmen nötig	169	88,0%	18	9,4%	5	2,6%
**Negative Gesundheitswirkungen Lärm**	Gering	196	90,3%	16	7,4%	5	2,3%
	Stark	620	79,4%	122	15,6%	39	5,0%
**Lautheit Innsbruck im Vergleich zu anderen Städten Österreichs**	Ruhiger	247	84,6%	36	12,3%	9	3,1%
	Gleich	451	81,9%	80	14,5%	20	3,6%
	Lauter	132	75,9%	27	15,5%	15	8,6%
**Einschätzung eigener Lärmbeitrag**	Gering	635	81,9%	110	14,2%	30	3,9%
	Durchschnittlich	185	81,5%	30	13,2%	12	5,3%
	Groß	22	75,9%	5	17,2%	2 (*)	6,9%

(*) zu kleine Fallzahlen für solide Auswertungen

6. Belästigung im Schlaf durch Flugverkehrslärm

Knapp 4% aller befragten Innsbruckerinnen und Innsbrucker fühlen sich im Schlaf durch Flugverkehrslärm stark gestört (*highly annoyed sleep*: gruppierte Antwortkategorien 8, 9 und 10 [=*belästigt / stört besonders stark*]), knapp 85% aller Probandinnen und Probanden sehen sich selbst als nur gering im Schlaf fluglärmbelästigt an (gruppierte Antwortkategorien 0 [=*belästigt / stört überhaupt nicht*], 1 und 2), davon über 70% *überhaupt nicht belästigt*:

Abbildung 204: Belästigung im Schlaf durch Flugverkehrslärm in Prozent

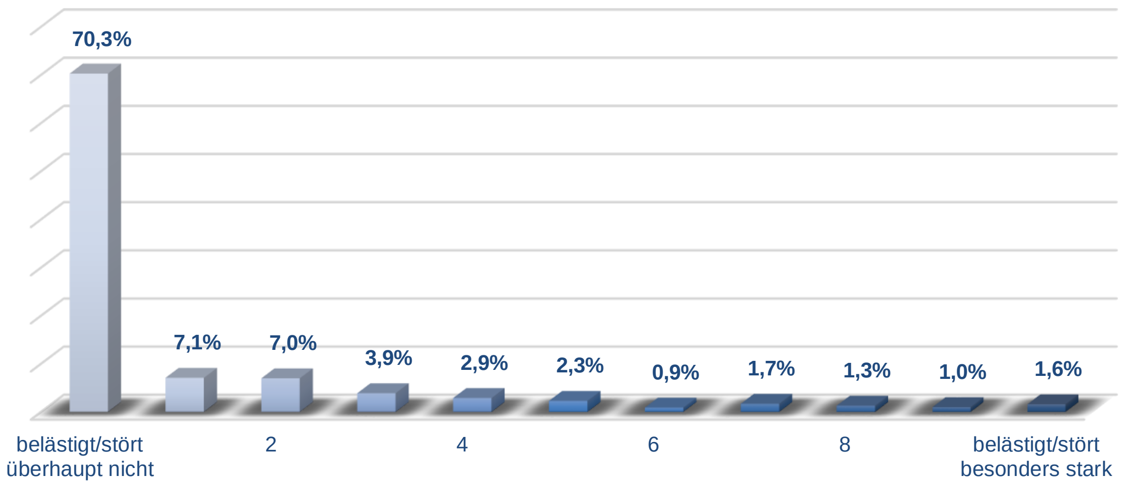

Im Mittel liegen die Antworten im befragten Kollektiv bei 1,09 (12,0%) auf der 11-teiligen Antwortskala.

Tabelle 152: Belästigung im Schlaf durch Fluglärm gruppiert

		Häufigkeit	Gültige Prozente	Kumulierte Prozente
Gültig	Gering belästigt	870	84,4	84,4
	Mittelgradig belästigt	121	11,7	96,1
	Stark belästigt	40	3,9	100,0
	Gesamt	1031	100,0	

6.1. Fluglärmbelästigung im Schlaf und Fluglärmbelastung

Die Belästigung im Schlaf durch den nächtlichen Flugverkehrslärm ist in den einzelnen – vergleichsweise niederen – Lärmbelastungsklassen im Unterschied zur empfundenen Störung im Schlaf durch den nächtlichen Straßen- und Schienenverkehrslärm in etwa gleich hoch wie im 24h-Zeitraum und steigt sehr linear mit zunehmender Belastung an. Für den Nachtzeitraum sind keine Befragten mit Dauerschallpegeln über 50dB belastet, weshalb das Belästigungsempfinden durch Flugverkehrslärm in der Nacht insgesamt vergleichsweise gering ausgeprägt ist:

Abbildung 205: Fluglärmbelästigung im Schlaf und nächtliche Fluglärmbelastung L_n in 10dB-Schritten im Mittelwertvergleich

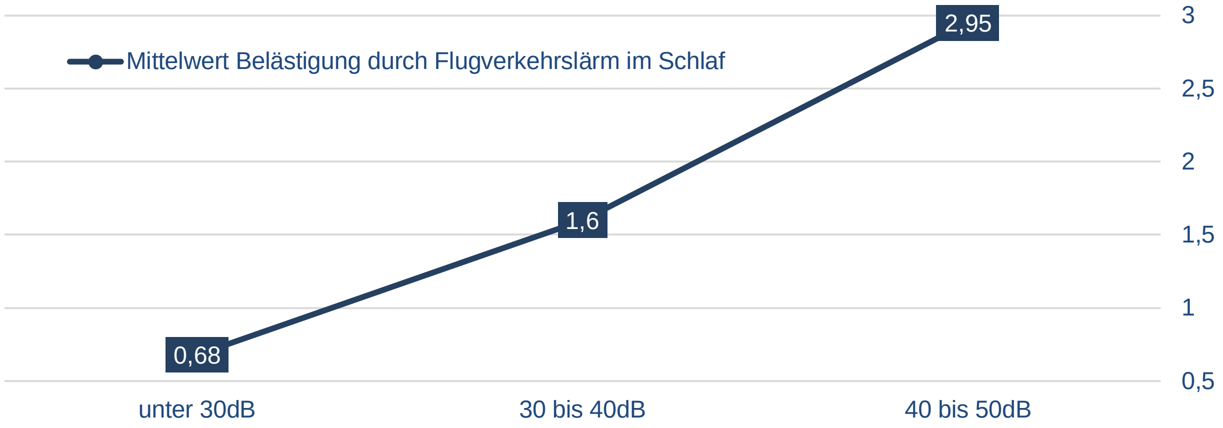

Anm.: Die Darstellung zeigt nachvollziehbare Zusammenhänge auf, enthält jedoch keine Prüfungen auf Kovariablen und andere Prädiktoren und ist somit nicht den Dosis-Wirkungskurven aus der gängigen Fachliteratur gleichzusetzen.

6.2. Fluglärmbelästigung im Schlaf und Katastralgemeinden

Entsprechend der gegebenen Fluglärmbelastung zeigt sich auch in der Nacht das stärkste Fluglärmbelästigungsempfinden in Hötting. Die geringste Störung durch Fluglärm im eigenen Wohnumfeld nehmen die Befragten in Igls, Mühlau und Vill wahr:

Abbildung 206: Fluglärmbelästigung im Schlaf und Katastralgemeinden im Mittelwertvergleich

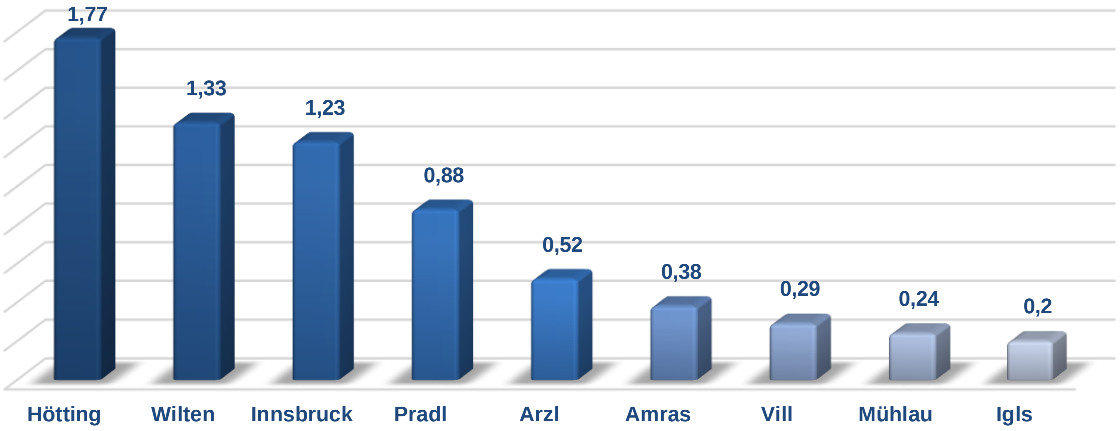

6.3. Fluglärmbelästigung im Schlaf und ausgewählte Parameter

Wie schon bei der Frage nach der Belästigung durch Flugverkehrslärm allgemein, sind die Unterschiede in den diversen Subgruppen für die demographischen Parameter vergleichsweise gering ausgeprägt. Ein deutlicher Bezug besteht hingegen zum höchsten (bisher) erreichten Schulabschluss. Je höher dieser ausfällt, desto stärker wird auch eine Belästigung im Schlaf durch Flugverkehrslärm wahrgenommen:

Tabelle 153: Belästigung im Schlaf durch Flugverkehrslärm und höchster erreichter Bildungsabschluss im Mittelwertvergleich

Bildungsschicht	Mittelwert	N	Standardabweichung	Standardfehler Mittelwert
Primärer Schulabschluss	,66	98	1,805	,182
Sekundärer Schulabschluss	1,00	542	2,110	,091
Tertiärer Schulabschluss	1,32	388	2,469	,125
Insgesamt	1,09	1028	2,234	,070

In der Zusammenschau mit verschiedenen Items entsprechen die Analysen weitestgehend den zu anderen Belästigungslärmquellen gemachten Auswertungen. Baulicher Schutz vor Verkehrslärm durch vorgelagerte Gebäude, Mauern etc. ist für die Belästigung durch Fluglärm von geringerer Bedeutung im Vergleich zu anderen Verkehrslärmquellen:

Tabelle 154: Belästigung im Schlaf durch Flugverkehrslärm und ausgewählte Parameter

		Belästigung im Schlaf Fluglärm gruppiert					
		Gering belästigt		Mittelgradig belästigt		Stark belästigt	
		Anzahl	Zeilen (%)	Anzahl	Zeilen (%)	Anzahl	Zeilen (%)
**Geschlecht**	weiblich	462	85,4%	61	11,3%	18	3,3%
	männlich	408	83,3%	60	12,2%	22	4,5%
**Altersgruppen**	18 bis 40 Jahre	369	83,9%	58	13,2%	13	3,0%
	41 bis 60 Jahre	267	84,5%	35	11,1%	14	4,4%
	über 60 Jahre	234	85,1%	28	10,2%	13	4,7%
**Bildungsschicht**	Primärer Schulabschluss	89	90,8%	7	7,1%	2	2,0%
	Sekundärer Schulabschluss	469	86,5%	54	10,0%	19	3,5%
	Tertiärer Schulabschluss	309	79,6%	60	15,5%	19	4,9%
**Haushalt mit/ohne minderjährige Kinder**	Haushalt ohne Kinder	640	84,0%	91	11,9%	31	4,1%
	Haushalt mit Kindern	230	85,5%	30	11,2%	9	3,3%
**Baulicher Schutz vor Verkehrslärm**	Keiner bis geringfügig	639	83,4%	92	12,0%	35	4,6%
	Gut bis komplett	203	86,0%	28	11,9%	5	2,1%
**Innenhof bzw. lärmabgewandte Seite**	Ja	604	86,2%	80	11,4%	17	2,4%
	Nein	247	80,2%	39	12,7%	22	7,1%
**Nachtschlaf**	Fenster geöffnet/gekippt	701	85,4%	90	11,0%	30	3,7%
	Fenster geschlossen	162	80,2%	30	14,9%	10	5,0%
**Wohnbedingungen**	Nicht gut	70	83,3%	10	11,9%	4	4,8%
	Durchschnittlich	458	82,5%	78	14,1%	19	3,4%
	Sehr gut	341	87,4%	32	8,2%	17	4,4%
**Bedingungen für Kinder**	Nicht gut	33	70,2%	10	21,3%	4	8,5%
	Durchschnittlich	430	83,5%	65	12,6%	20	3,9%
	Sehr gut	390	87,2%	41	9,2%	16	3,6%
**Verbundenheit zu Innsbruck**	Nicht verbunden	41	74,5%	10	18,2%	4	7,3%
	Durchschnittlich	315	83,1%	51	13,5%	13	3,4%
	Sehr verbunden	512	86,1%	60	10,1%	23	3,9%

**Zufriedenheit eigene Wohnsituation**	Nicht zufrieden	22	78,6%	4 (*)	14,3%	2	7,1%
	Durchschnittlich	206	79,8%	38	14,7%	14	5,4%
	Sehr zufrieden	641	86,2%	79	10,6%	24	3,2%
**Lebensbedingungen (Summenscore)**	Nicht gut (*)	2	40,0%	2	40,0%	1	20,0%
	Durchschnittlich	551	82,2%	90	13,4%	29	4,3%
	Sehr gut	317	89,0%	29	8,1%	10	2,8%
**Lebensqualität**	Nicht gut (*)	5	50,0%	4	40,0%	1	10,0%
	Durchschnittlich	228	78,6%	46	15,9%	16	5,5%
	Sehr gut	633	87,3%	70	9,7%	22	3,0%
**Lärmempfindlichkeit**	Nicht lärmempfindlich	259	94,2%	12	4,4%	4	1,5%
	Durchschnittlich	478	81,8%	81	13,9%	25	4,3%
	Sehr lärmempfindlich	133	77,3%	28	16,3%	11	6,4%
**Summenscore Lärmcoping**	Coping ausgeprägt	171	70,7%	50	20,7%	21	8,7%
	Coping nicht ausgeprägt	698	88,6%	71	9,0%	19	2,4%
**Gefühlte Zunahme Lärmbelastung**	Ja	338	77,5%	71	16,3%	27	6,2%
	Nein	532	89,4%	50	8,4%	13	2,2%
**Gefühlte Abnahme Lärmbelastung**	Ja	54	93,1%	4 (*)	6,9%	0	0,0%
	Nein	816	83,9%	117	12,0%	40	4,1%
**Notwendigkeit (weiterer) Maßnahmen Lärm**	Maßnahmen notwendig	558	80,8%	95	13,7%	38	5,5%
	Keine Maßnahmen nötig	179	93,2%	12	6,3%	1	0,5%
**Negative Gesundheitswirkungen Lärm**	Gering	202	93,1%	14	6,5%	1	0,5%
	Stark	640	81,9%	104	13,3%	37	4,7%
**Lautheit Innsbruck im Vergleich zu anderen Städten Österreichs**	Ruhiger	257	88,0%	29	9,9%	6	2,1%
	Gleich	464	84,2%	61	11,1%	26	4,7%
	Lauter	136	78,2%	30	17,2%	8	4,6%
**Einschätzung eigener Lärmbeitrag**	Gering	665	85,8%	80	10,3%	30	3,9%
	Durchschnittlich	182	80,2%	38	16,7%	7	3,1%
	Groß	23	79,3%	3 (*)	10,3%	3	10,3%

(*) zu kleine Fallzahlen für solide Auswertungen
Anm.: Die Fallzahlen in der Gruppe der Starkbelästigten (*highly annoyed*, N=40) sind in einigen der obenstehenden Subgruppen zu gering für solide Auswertungen.

7. Belästigung im Schlaf durch Baustellenlärm

Für die Nachtstunden ist nicht von ausgeprägten Baustellentätigkeiten auszugehen, was sich auch in der Beantwortung durch die Befragten nach der Belästigung im Schlaf durch Baustellen widerspiegelt: Knapp 90% aller Befragten sehen sich überhaupt nicht bis gering von Baustellenlärm im Schlaf gestört, 2,1% gehören hingegen der Gruppe der stark im Schlaf Lärmbelästigten an. Eine Person macht zu dieser Frage keine Angaben.

Im Mittel liegen die Antworten der befragten Innsbruckerinnen und Innsbrucker bei 0,73 (8,0%):

Abbildung 207: Belästigung durch Baustellenlärm im Schlaf in Prozent

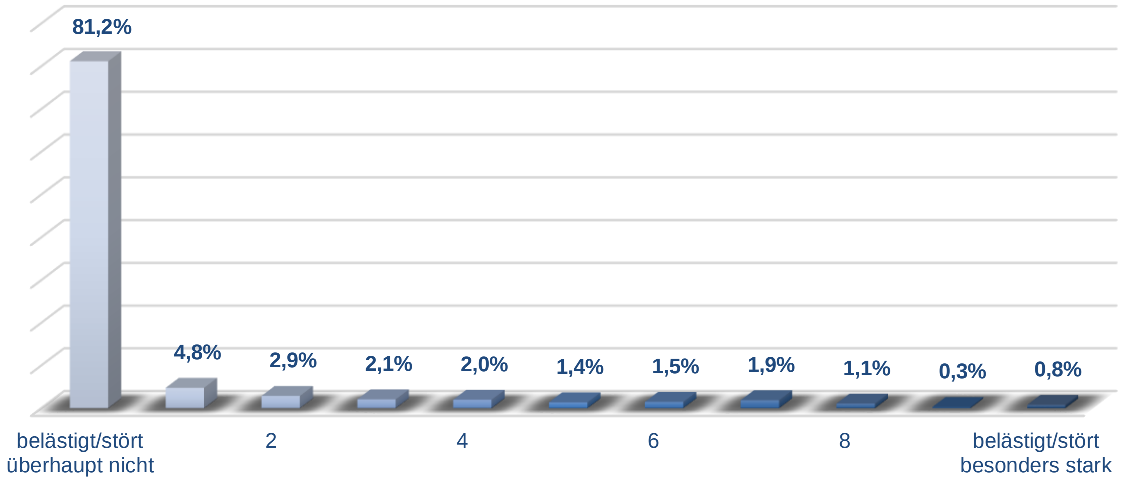

Tabelle 155: Belästigung im Schlaf durch Lärm von Baustellen gruppiert

		Häufigkeit	Gültige Prozente	Kumulierte Prozente
Gültig	Gering belästigt	916	88,8	88,8
	Mittelgradig belästigt	92	8,9	97,8
	Stark belästigt	22	2,1	99,9
	w.n. / k.A.	1	,1	100,0
	Gesamt	1031	100,0	

7.1. Baulärmbelästigung im Schlaf und Katastralgemeinden

Baustellenlärm ist stark auf Akutbelastungen bezogen und daher ist ein Vergleich der Katastralgemeinden zu einem sehr erheblichen Anteil nur eine Momentaufnahme. Durch die geringen Belästigungsausmaße verursacht von Baustellenlärm in der Nacht, verstärkt sich dieser Effekt. Die Befragten in der Katastralgemeinde Innsbruck geben gemittelt das größte Ausmaß an Belästigung durch Baustellenlärm in der Nacht an, die befragten in Vill, Igls und Amras das niedrigste:

Abbildung 208: Belästigung im Schlaf durch Baulärm und Katastralgemeinden im Mittelwertvergleich

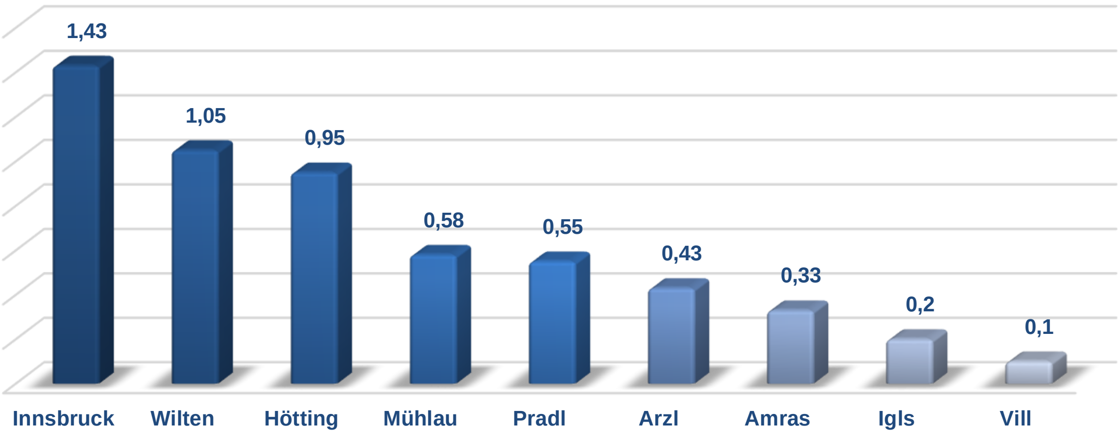

7.2. Baulärmbelästigung im Schlaf und ausgewählte Parameter

Die Unterschiede zwischen den einzelnen Subgruppen in der Kombination mit anderen Items sind insgesamt geringer ausgeprägt als bei anderen Belästigungslärmquellen.

Im Folgenden einige ausgewählte Parameter, jeweils in 3 Belästigungsgruppen zusammengefasst, in Zusammenschau mit der Belästigung im Schlaf durch Baulärm:

Tabelle 156: Belästigung im Schlaf durch Baustellenlärm und ausgewählte Parameter

		Belästigung im Schlaf Lärm von Baustellen gruppiert					
		Gering belästigt		Mittelgradig belästigt		Stark belästigt	
		Anzahl	Zeilen (%)	Anzahl	Zeilen (%)	Anzahl	Zeilen (%)
**Geschlecht**	weiblich	483	89,3%	48	8,9%	10	1,8%
	männlich	433	88,5%	44	9,0%	12	2,5%
**Altersgruppen**	18 bis 40 Jahre	378	86,1%	48	10,9%	13	3,0%
	41 bis 60 Jahre	281	88,9%	28	8,9%	7	2,2%
	über 60 Jahre	257	93,5%	16	5,8%	2	0,7%
**Bildungsschicht**	Primärer Schulabschluss	92	93,9%	5	5,1%	1	1,0%
	Sekundärer Schulabschluss	477	88,0%	57	10,5%	8	1,5%
	Tertiärer Schulabschluss	344	88,9%	30	7,8%	13	3,4%
**Baulicher Schutz vor Verkehrslärm**	Keiner bis geringfügig	674	88,1%	73	9,5%	18	2,4%
	Gut bis komplett	216	91,5%	18	7,6%	2	0,8%
**Innenhof bzw. lärm-abgewandte Seite**	Ja	625	89,2%	67	9,6%	9	1,3%
	Nein	270	87,9%	24	7,8%	13	4,2%
**Nachtschlaf**	Fenster geöffnet/gekippt	735	89,6%	64	7,8%	21	2,6%
	Fenster geschlossen	174	86,1%	27	13,4%	1	0,5%
**Wohnbedingungen**	Nicht gut	73	86,9%	9	10,7%	2	2,4%
	Durchschnittlich	492	88,6%	54	9,7%	9	1,6%
	Sehr gut	350	90,0%	28	7,2%	11	2,8%
**Bedingungen für Kinder**	Nicht gut	37	78,7%	8	17,0%	2	4,3%
	Durchschnittlich	451	87,7%	53	10,3%	10	1,9%
	Sehr gut	410	91,7%	27	6,0%	10	2,2%
**Verbundenheit zu Innsbruck**	Nicht verbunden	48	87,3%	6	10,9%	1	1,8%
	Durchschnittlich	327	86,5%	41	10,8%	10	2,6%
	Sehr verbunden	539	90,6%	45	7,6%	11	1,8%
**Zufriedenheit eigene Wohnsituation**	Nicht zufrieden	25	89,3%	3 (*)	10,7%	0	0,0%
	Durchschnittlich	208	80,6%	36	14,0%	14	5,4%
	Sehr zufrieden	682	91,8%	53	7,1%	8	1,1%
**Lebensbedingungen (Summenscore)**	Nicht gut (*)	4	80,0%	1	20,0%	0	0,0%
	Durchschnittlich	588	87,9%	67	10,0%	14	2,1%
	Sehr gut	324	91,0%	24	6,7%	8	2,2%
**Lebensqualität**	Nicht gut (*)	8	80,0%	1	10,0%	1	10,0%
	Durchschnittlich	251	86,6%	33	11,4%	6	2,1%
	Sehr gut	653	90,1%	57	7,9%	15	2,1%

**Lärmempfindlichkeit**	Nicht lärmempfindlich	253	92,0%	18	6,5%	4	1,5%
	Durchschnittlich	508	87,0%	62	10,6%	14	2,4%
	Sehr lärmempfindlich	155	90,6%	12	7,0%	4	2,3%
**Summenscore Lärmcoping**	Coping ausgeprägt	194	80,2%	36	14,9%	12	5,0%
	Coping nicht ausgeprägt	722	91,7%	56	7,1%	9	1,1%
**Gefühlte Zunahme Lärmbelastung**	Ja	359	82,3%	60	13,8%	17	3,9%
	Nein	557	93,8%	32	5,4%	5	0,8%
**Gefühlte Abnahme Lärmbelastung**	Ja	55	94,8%	3	5,2%	0	0,0%
	Nein	861	88,6%	89	9,2%	22	2,3%
**Notwendigkeit (weiterer) Maßnahmen Lärm**	Maßnahmen notwendig	605	87,7%	69	10,0%	16	2,3%
	Keine Maßnahmen nötig	177	92,2%	11	5,7%	4	2,1%
**Negative Gesundheitswirkungen Lärm**	Gering	201	92,6%	14	6,5%	2	0,9%
	Stark	688	88,2%	73	9,4%	19	2,4%
**Lautheit Innsbruck im Vergleich zu anderen Städten Österreichs**	Ruhiger	263	90,1%	24	8,2%	5	1,7%
	Gleich	489	88,7%	49	8,9%	13	2,4%
	Lauter	151	86,8%	19	10,9%	4	2,3%
**Einschätzung eigener Lärmbeitrag**	Gering	686	88,6%	71	9,2%	17	2,2%
	Durchschnittlich	205	90,3%	18	7,9%	4	1,8%
	Groß	25	86,2%	3 (*)	10,3%	1	3,4%

(*) zu kleine Fallzahlen für solide Auswertungen
Anm.: Die Fallzahlen in der Gruppe der Starkbelästigten (*highly annoyed*, N=22) sind in einigen der obenstehenden Subgruppen zu gering für solide Auswertungen.

8. Belästigung im Schlaf durch Schienenverkehrslärm

Keine Belästigung durch Schienenverkehrslärm im Schlaf nehmen über 83% der Befragten wahr, insgesamt sind über 90% überhaupt nicht bis gering lärmbelästigt. 1,5% sehen sich als stark vom Schienenverkehrslärm im Schlaf gestört an:

Abbildung 209: Belästigung durch Schienenverkehrslärm im Schlaf in Prozent

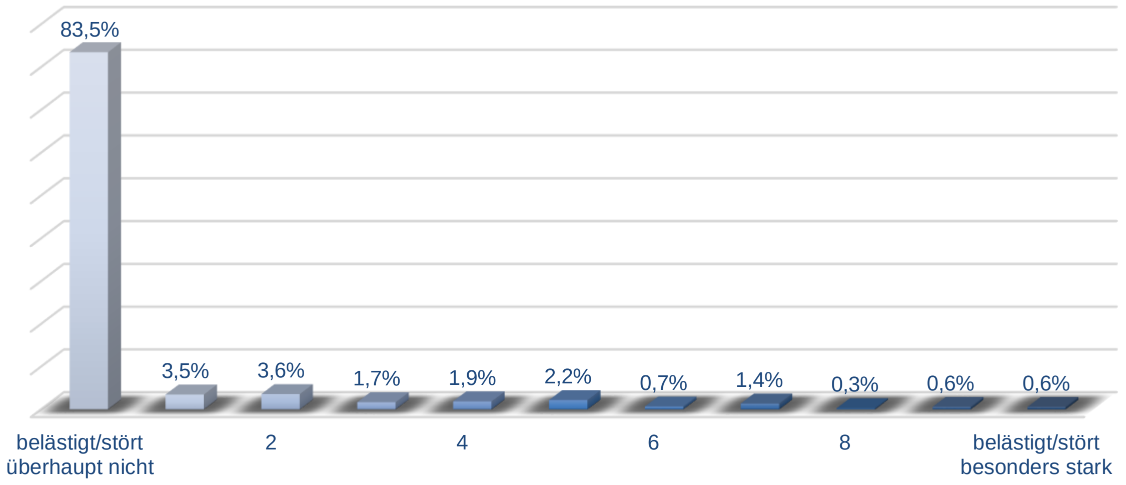

Im Mittel liegen die Antworten im befragten Kollektiv bei 0,62 (6,8%) auf der 11-teiligen Antwortskala:

Tabelle 157: Belästigung im Schlaf durch Schienenverkehrslärm gruppiert

		Häufigkeit	Gültige Prozente	Kumulierte Prozente
Gültig	Gering belästigt	934	90,6	90,6
	Mittelgradig belästigt	82	8,0	98,5
	Stark belästigt	15	1,5	100,0
	Gesamt	1031	100,0	

8.1. Schienenlärmbelästigung im Schlaf und Schienenlärmbelastung

Eine Belästigung durch den Schienenverkehrslärm im Schlaf ist bis zu Dauerbelastungen von 40dB im Wohnumfeld der Befragten de facto nicht gegeben und fällt auch bei nächtlichen Dauerlärmpegeln über 50dB vergleichsweise gering auS. Eine steigende Belästigungsreaktion (auf niedrigem Niveau) beginnt bei etwa 10dB niedrigeren Lärmpegeln als im 24h-Zeitraum einzusetzen:

Abbildung 210: Schienenlärmbelästigung im Schlaf und nächtliche Schienenlärmbelastung L_n in 10dB-Schritten im Mittelwertvergleich

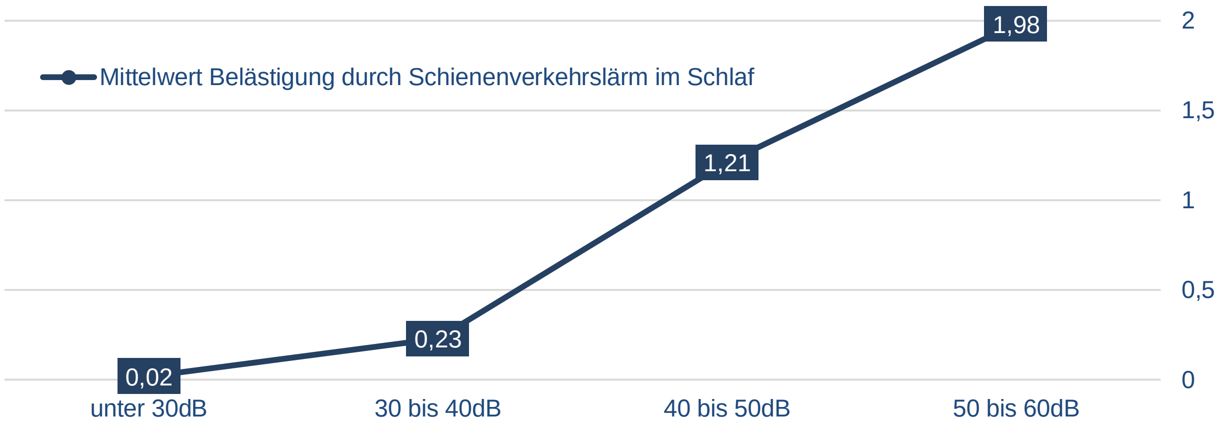

Anm.: Die Darstellung zeigt nachvollziehbare Zusammenhänge auf, enthält jedoch keine Prüfungen auf Kovariablen und andere Prädiktoren und ist somit nicht den Dosis-Wirkungskurven aus der gängigen Fachliteratur gleichzusetzen.

8.2. Schienenlärmbelästigung im Schlaf und Katastralgemeinden

Entsprechend der gegebenen Schienenverkehrslärmbelastung zeigt sich auch für den Nachtzeitraum die stärkste Schienenlärmbelästigung in Innsbruck und Wilten. Die geringste Störung durch Schienenlärm im Schlaf im eigenen Wohnumfeld empfinden die Befragten in Igls und Vill, wo – wie schon für den 24h-Zeitraum – fast überhaupt keine Belästigung durch den Schienenverkehrslärm von den Befragten festgestellt wird:

Abbildung 211: Schienenlärmbelästigung im Schlaf und Katastralgemeinden im Mittelwertvergleich

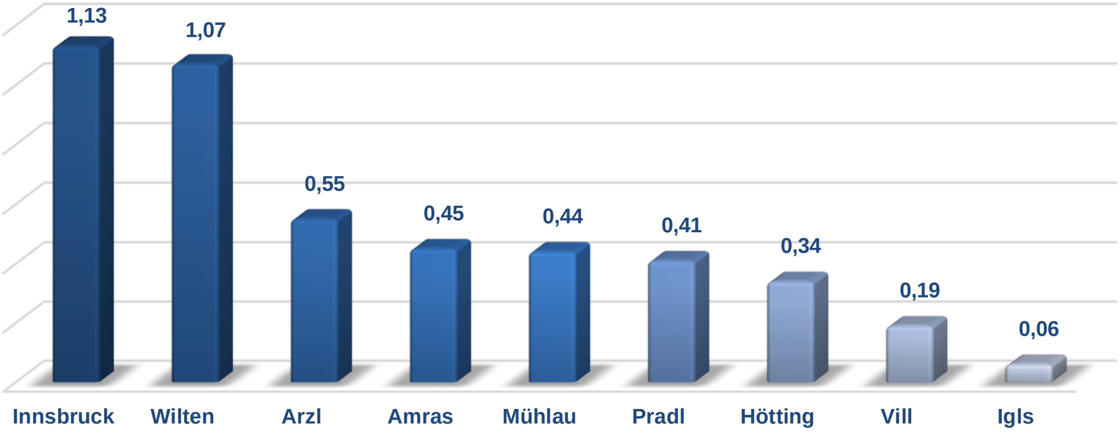

8.3. Schienenlärmbelästigung im Schlaf und ausgewählte Parameter

Die Fallzahlen im Bereich abseits der *überhaupt nicht* oder nur sehr geringfügig im Schlaf durch Schienenverkehrslärm belästigten Befragten sind ausgesprochen klein, weshalb nur sehr eingeschränkt gültige Ableitungen aus bi- und multivariaten Auswertungen zulässig sind. Größere Auffälligkeiten im Vergleich zu den anderen Belästigungslärmquellen oder der Frage nach der Belästigung durch Schienenverkehrslärm allgemein konnten nicht festgemacht werden.

Im Folgenden einige ausgewählte Parameter, jeweils in 3 Belästigungsgruppen zusammengefasst, in Zusammenschau mit der Belästigung im Schlaf durch Schienenverkehrslärm:

Tabelle 158: Belästigung im Schlaf durch Schienenverkehrslärm und ausgewählte Parameter

		Belästigung im Schlaf Schienenverkehrslärm gruppiert					
		Gering belästigt		Mittelgradig belästigt		Stark belästigt	
		Anzahl	Zeilen (%)	Anzahl	Zeilen (%)	Anzahl	Zeilen (%)
**Geschlecht**	weiblich	491	90,8%	40	7,4%	10	1,8%
	männlich	443	90,4%	42	8,6%	5	1,0%
**Altersgruppen**	18 bis 40 Jahre	395	89,8%	38	8,6%	7	1,6%
	41 bis 60 Jahre	285	90,2%	24	7,6%	7	2,2%
	über 60 Jahre	254	92,4%	20	7,3%	1	0,4%
**Bildungsschicht**	Primärer Schulabschluss	92	93,9%	6	6,1%	0	0,0%
	Sekundärer Schulabschluss	496	91,5%	40	7,4%	6	1,1%
	Tertiärer Schulabschluss	343	88,4%	36	9,3%	9	2,3%
**Haushalt mit/ohne minderjährige Kinder**	Haushalt ohne Kinder	693	90,9%	59	7,7%	10	1,3%
	Haushalt mit Kindern	241	89,6%	23	8,6%	5	1,9%
**Baulicher Schutz vor Verkehrslärm**	Keiner bis geringfügig	685	89,4%	68	8,9%	13	1,7%
	Gut bis komplett	222	94,1%	12	5,1%	2	0,8%
**Innenhof bzw. lärm-abgewandte Seite**	Ja	637	90,9%	56	8,0%	8	1,1%
	Nein	277	89,9%	24	7,8%	7	2,3%
**Nachtschlaf**	Fenster geöffnet/gekippt	756	92,1%	58	7,1%	7	0,9%

	Fenster geschlossen	170	84,2%	24	11,9%	8	4,0%
**Wohnbedingungen**	Nicht gut	75	89,3%	7	8,3%	2	2,4%
	Durchschnittlich	495	89,2%	50	9,0%	10	1,8%
	Sehr gut	363	93,1%	24	6,2%	3	0,8%
**Bedingungen für Kinder**	Nicht gut	41	87,2%	5	10,6%	1	2,1%
	Durchschnittlich	458	88,9%	49	9,5%	8	1,6%
	Sehr gut	414	92,6%	27	6,0%	6	1,3%
**Verbundenheit zu Innsbruck**	Nicht verbunden	51	92,7%	3 (*)	5,5%	1	1,8%
	Durchschnittlich	340	89,7%	34	9,0%	5	1,3%
	Sehr verbunden	541	90,9%	45	7,6%	9	1,5%
**Zufriedenheit eigene Wohnsituation**	Nicht zufrieden	23	82,1%	5	17,9%	0	0,0%
	Durchschnittlich	225	87,2%	28	10,9%	5	1,9%
	Sehr zufrieden	685	92,1%	49	6,6%	10	1,3%
**Lebensbedingungen (Summenscore)**	Nicht gut (*)	2	40,0%	2	40,0%	1	20,0%
	Durchschnittlich	594	88,7%	64	9,6%	12	1,8%
	Sehr gut	338	94,9%	16	4,5%	2	0,6%
**Lebensqualität**	Nicht gut (*)	10	100,0%	0	0,0%	0	0,0%
	Durchschnittlich	255	87,9%	31	10,7%	4	1,4%
	Sehr gut	665	91,7%	49	6,8%	11	1,5%
**Lärmempfindlichkeit**	Nicht lärmempfindlich	261	94,9%	12	4,4%	2	0,7%
	Durchschnittlich	519	88,9%	55	9,4%	10	1,7%
	Sehr lärmempfindlich	154	89,5%	15	8,7%	3	1,7%
**Summenscore Lärmcoping**	Coping ausgeprägt	193	79,8%	39	16,1%	10	4,1%
	Coping nicht ausgeprägt	740	93,9%	43	5,5%	5	0,6%
**Gefühlte Zunahme Lärmbelastung**	Ja	383	87,8%	42	9,6%	11	2,5%
	Nein	551	92,6%	40	6,7%	4	0,7%
**Gefühlte Abnahme Lärmbelastung**	Ja	52	89,7%	6	10,3%	0	0,0%
	Nein	882	90,6%	76	7,8%	15	1,5%
**Notwendigkeit (weiterer) Maßnahmen Lärm**	Maßnahmen notwendig	609	88,1%	69	10,0%	13	1,9%
	Keine Maßnahmen nötig	185	96,4%	7	3,6%	0	0,0%
**Negative Gesundheitswirkungen Lärm**	Gering	201	92,6%	13	6,0%	3	1,4%
	Stark	701	89,8%	68	8,7%	12	1,5%
**Lautheit Innsbruck im Vergleich zu anderen Städten Österreichs**	Ruhiger	274	93,8%	16	5,5%	2	0,7%
	Gleich	495	89,8%	44	8,0%	12	2,2%
	Lauter	151	86,8%	22	12,6%	1	0,6%
**Einschätzung eigener Lärmbeitrag**	Gering	704	90,8%	56	7,2%	15	1,9%
	Durchschnittlich	203	89,4%	24	10,6%	0	0,0%
	Groß	27	93,1%	2 (*)	6,9%	0	0,0%

(*) zu kleine Fallzahlen für solide Auswertungen
Anm.: Die Fallzahlen in der Gruppe der Starkbelästigten (*highly annoyed*, N=15) sind in einigen der obenstehenden Subgruppen zu gering für solide Auswertungen.

9. Belästigung im Schlaf durch Erschütterungen

Die Belästigungsausprägungen durch Erschütterungen im Schlaf sind in ihren Dimensionen nahezu deckungsgleich jenen des Schienenverkehrslärms und fallen sehr gering auS. Im Mittel liegen die Antworten der Befragten bei 0,53 (5,8%):

Abbildung 212: Belästigung durch Erschütterungen im Schlaf in Prozent

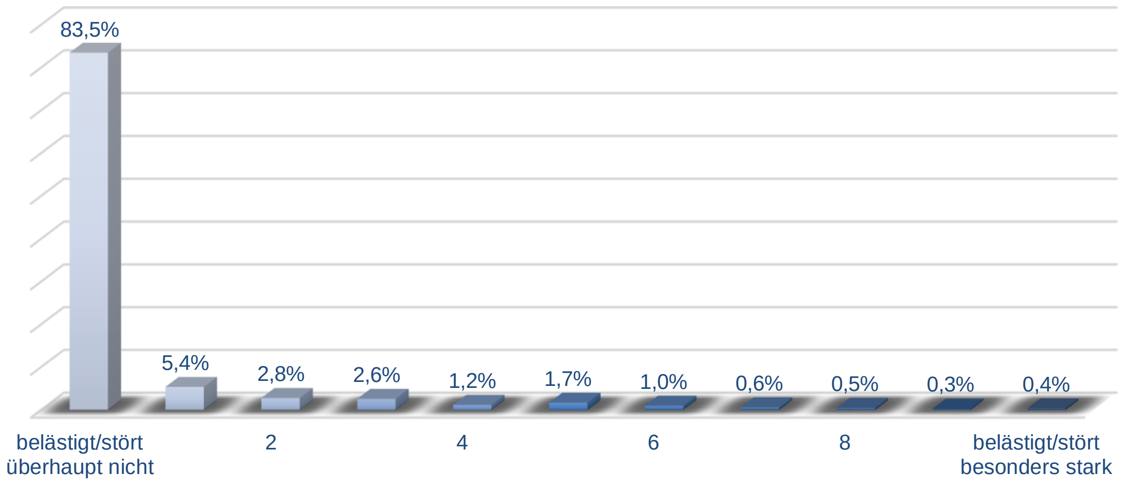

Tabelle 159: Belästigung im Schlaf durch Erschütterungen gruppiert

		Häufigkeit	Gültige Prozente	Kumulierte Prozente
Gültig	Gering belästigt	946	91,8	91,8
	Mittelgradig belästigt	73	7,1	98,8
	Stark belästigt	12	1,2	100,0
	Gesamt	1031	100,0	

9.1. Belästigung im Schlaf durch Erschütterungen und Katastralgemeinden

Abbildung 213: Belästigung im Schlaf durch Erschütterungen und Katastralgemeinden im Mittelwertvergleich

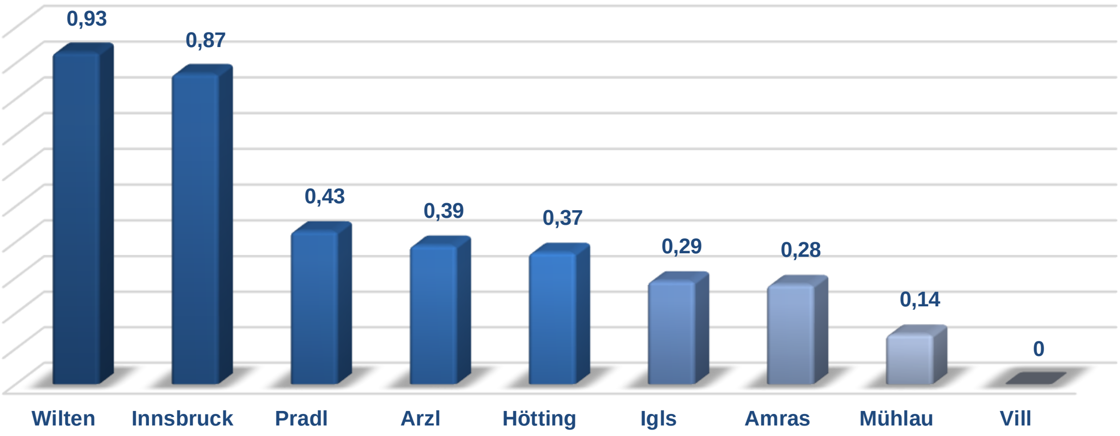

Das größte Ausmaß an Belästigung im Schlaf durch Erschütterungen wird in Wilten und Innsbruck empfunden, das geringste in Vill und Mühlau.

9.2. Belästigung im Schlaf durch Erschütterungen und ausgewählte Parameter

Die Auswertungen zur Belästigung im Schlaf durch Erschütterungen zeigen keine größeren Auffälligkeiten im Vergleich zu den anderen Belästigungslärmquellen. Die Fallzahlen in der Extremgruppe der Starkbelästigten (*highly annoyed*, N=12) sind zu gering für solide Auswertungen, weshalb diese Gruppe in der untenstehenden Tabelle ganz ausgespart wurde.

Im Folgenden einige ausgewählte Parameter, jeweils in die beiden Gruppen der gering und der mittelgradig Belasteten zusammengefasst, in Zusammenschau mit der Belästigung im Schlaf durch Erschütterungen:

Tabelle 160: Belästigung im Schlaf durch Erschütterungen und ausgewählte Parameter

		Belästigung im Schlaf Erschütterungen gesamt gruppiert			
		Gering belästigt		Mittelgradig belästigt	
		Anzahl	Zeilen (%)	Anzahl	Zeilen (%)
**Geschlecht**	weiblich	497	91,9%	35	6,5%
	männlich	449	91,6%	38	7,8%
**Altersgruppen**	18 bis 40 Jahre	404	91,8%	31	7,0%
	41 bis 60 Jahre	281	88,9%	29	9,2%
	über 60 Jahre	261	94,9%	13	4,7%
**Bildungsschicht**	Primärer Schulabschluss	92	93,9%	5	5,1%
	Sekundärer Schulabschluss	495	91,3%	42	7,7%
	Tertiärer Schulabschluss	356	91,8%	26	6,7%
**Haushalt mit/ohne minderjährige Kinder**	Haushalt ohne Kinder	697	91,5%	56	7,3%
	Haushalt mit Kindern	249	92,6%	17	6,3%
**Baulicher Schutz vor Verkehrslärm**	Keiner bis geringfügig	695	90,7%	59	7,7%
	Gut bis komplett	223	94,5%	13	5,5%
**Innenhof bzw. lärm-abgewandte Seite**	Ja	646	92,2%	46	6,6%
	Nein	279	90,6%	26	8,4%
**Nachtschlaf**	Fenster geöffnet/gekippt	762	92,8%	51	6,2%
	Fenster geschlossen	177	87,6%	21	10,4%
**Wohnbedingungen**	Nicht gut	74	88,1%	7	8,3%
	Durchschnittlich	505	91,0%	43	7,7%
	Sehr gut	365	93,6%	23	5,9%
**Bedingungen für Kinder**	Nicht gut	34	72,3%	10	21,3%
	Durchschnittlich	469	91,1%	39	7,6%
	Sehr gut	423	94,6%	22	4,9%
**Verbundenheit zu**	Nicht verbunden	44	80,0%	8	14,5%

**Innsbruck**	Durchschnittlich	347	91,6%	29	7,7%
	Sehr verbunden	553	92,9%	36	6,1%
**Zufriedenheit eigene Wohnsituation**	Nicht zufrieden	23	82,1%	3 (*)	10,7%
	Durchschnittlich	222	86,0%	28	10,9%
	Sehr zufrieden	700	94,1%	42	5,6%
**Lebensbedingungen (Summenscore)**	Nicht gut (*)	5	100,0%	0	0,0%
	Durchschnittlich	601	89,7%	58	8,7%
	Sehr gut	340	95,5%	15	4,2%
**Lebensqualität**	Nicht gut (*)	6	60,0%	2	20,0%
	Durchschnittlich	260	89,7%	24	8,3%
	Sehr gut	675	93,1%	46	6,3%
**Lärmempfindlichkeit**	Nicht lärmempfindlich	262	95,3%	10	3,6%
	Durchschnittlich	531	90,9%	48	8,2%
	Sehr lärmempfindlich	153	89,0%	15	8,7%
**Summenscore Lärmcoping**	Coping ausgeprägt	198	81,8%	38	15,7%
	Coping nicht ausgeprägt	748	94,9%	35	4,4%
**Gefühlte Zunahme Lärmbelastung**	Ja	387	88,8%	40	9,2%
	Nein	559	93,9%	33	5,5%
**Gefühlte Abnahme Lärmbelastung**	Ja	56	96,6%	2 (*)	3,4%
	Nein	890	91,5%	71	7,3%
**Notwendigkeit (weiterer) Maßnahmen Lärm**	Maßnahmen notwendig	618	89,4%	61	8,8%
	Keine Maßnahmen nötig	184	95,8%	8	4,2%
**Negative Gesundheitswirkungen Lärm**	Gering	212	97,7%	4 (*)	1,8%
	Stark	702	89,9%	68	8,7%
**Lautheit Innsbruck im Vergleich zu anderen Städten Österreichs**	Ruhiger	275	94,2%	17	5,8%
	Gleich	508	92,2%	32	5,8%
	Lauter	149	85,6%	24	13,8%
**Einschätzung eigener Lärmbeitrag**	Gering	714	92,1%	52	6,7%
	Durchschnittlich	207	91,2%	18	7,9%
	Groß	25	86,2%	3 (*)	10,3%

(*) zu kleine Fallzahlen für solide Auswertungen

10. Belästigung im Schlaf durch Lärm aus Gewerbe und Industrie

Eine Belästigung in den Nachtstunden durch Lärm aus Gewerbe und Industrie ist für die überwiegende Mehrheit der Befragten in Innsbruck nicht vorhanden. Mehr als 90% sehen sich überhaupt nicht gestört, rund ein Prozent (10 von 1031 befragten Personen) fällt in die Gruppe der stark vom Lärm aus Gewerbe und Industrie im Schlaf Belästigten. Im Mittel liegen die Antworten im befragten Kollektiv bei 0,35 (3,9,0%) auf der 11-teiligen Antwortskala:

Abbildung 214: Belästigung im Schlaf durch Lärm von Gewerbe und Industrie in Prozent

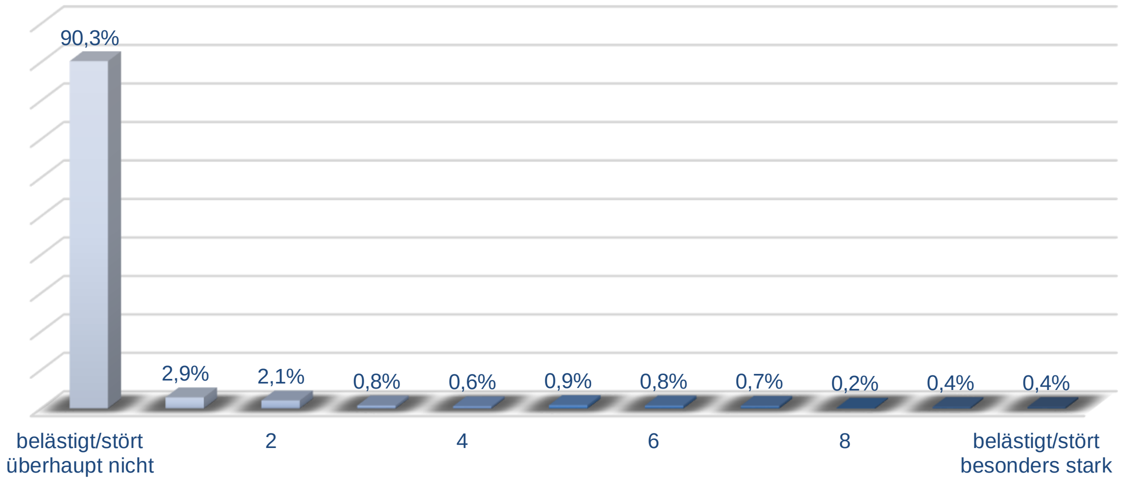

Tabelle 161: Belästigung im Schlaf durch Lärm von Gewerbe und Industrie gruppiert

		Häufigkeit	Gültige Prozente	Kumulierte Prozente
Gültig	Gering belästigt	983	95,3	95,3
	Mittelgradig belästigt	38	3,7	99,0
	Stark belästigt	10	1,0	100,0
	Gesamt	1031	100,0	

10.1. Belästigung im Schlaf durch Lärm von Gewerbe und Industrie und Katastralgemeinden

Die Störung im Schlaf durch Lärm von Gewerbe und Industrie ist in Mühlau und Vill am geringsten und in den Katastralgemeinden Innsbruck und Wilten am stärksten ausgeprägt:

Abbildung 215: Belästigung im Schlaf durch Lärm von Gewerbe und Industrie und Katastralgemeinden im Mittelwertvergleich

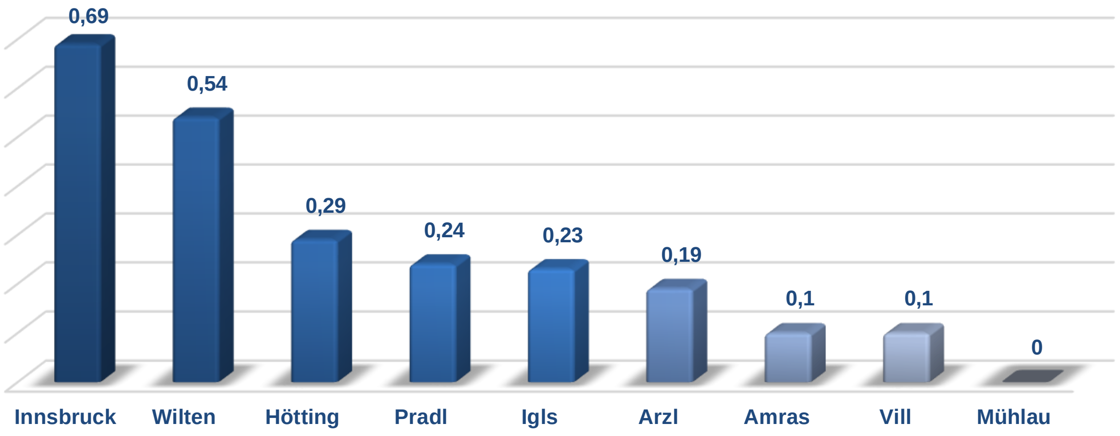

10.2. Belästigung im Schlaf durch Lärm von Gewerbe und Industrie und ausgewählte Parameter

Die Auswertungen zur Belästigung im Schlaf durch den Lärm von Gewerbe und Industrie zeigen keine Auffälligkeiten im Vergleich zu den anderen Belästigungslärmquellen. Allerdings sind die Fallzahlen der Mittel- und Starkbelasteten sehr gering, weshalb die Aussagekraft der Extremgruppenvergleiche stark eingeschränkt ist. Besonders in der Extremgruppe der Starkbelästigten (*highly annoyed*, N=10) sind zu wenige Fälle für solide Auswertungen enthalten, weshalb diese Gruppe in der untenstehenden Tabelle ganz ausgespart wurde.

Im Folgenden einige ausgewählte Parameter, jeweils in die beiden Gruppen der gering und der mittelgradig Belasteten zusammengefasst, in Zusammenschau mit der Belästigung im Schlaf durch den Lärm von Gewerbe und Industrie:

Tabelle 162: Belästigung im Schlaf durch Lärm von Gewerbe / Industrie und ausgewählte Parameter

		Belästigung im Schlaf Lärm von Gewerbe und Industrie gruppiert			
		Gering belästigt		Mittelgradig belästigt	
		Anzahl	Zeilen (%)	Anzahl	Zeilen (%)
**Geschlecht**	weiblich	521	96,3%	13	2,4%
	männlich	462	94,3%	25	5,1%
**Altersgruppen**	18 bis 40 Jahre	416	94,5%	16	3,6%
	41 bis 60 Jahre	299	94,6%	16	5,1%
	über 60 Jahre	268	97,5%	6	2,2%
**Bildungsschicht**	Primärer Schulabschluss	96	98,0%	1	1,0%
	Sekundärer Schulabschluss	515	95,0%	23	4,2%
	Tertiärer Schulabschluss	369	95,1%	14	3,6%
**Haushalt mit/ohne minderjährige Kinder**	Haushalt ohne Kinder	727	95,4%	29	3,8%
	Haushalt mit Kindern	256	95,2%	9	3,3%
**Baulicher Schutz vor Verkehrslärm**	Keiner bis geringfügig	723	94,4%	33	4,3%
	Gut bis komplett	232	98,3%	4	1,7%
**Innenhof bzw. lärm-abgewandte Seite**	Ja	673	96,0%	24	3,4%
	Nein	289	93,8%	13	4,2%
**Nachtschlaf**	Fenster geöffnet/gekippt	788	96,0%	28	3,4%
	Fenster geschlossen	188	93,1%	9	4,5%
**Wohnbedingungen**	Nicht gut	79	94,0%	4	4,8%
	Durchschnittlich	529	95,3%	20	3,6%
	Sehr gut	373	95,6%	14	3,6%
**Bedingungen für Kinder**	Nicht gut	41	87,2%	5	10,6%
	Durchschnittlich	488	94,8%	18	3,5%
	Sehr gut	433	96,9%	14	3,1%
**Verbundenheit zu Innsbruck**	Nicht verbunden	47	85,5%	7	12,7%
	Durchschnittlich	359	94,7%	14	3,7%
	Sehr verbunden	575	96,6%	17	2,9%
**Zufriedenheit eigene Wohnsituation**	Nicht zufrieden	25	89,3%	3	10,7%
	Durchschnittlich	234	90,7%	15	5,8%

	Sehr zufrieden	723	97,2%	20	2,7%
**Lebensbedingungen (Summenscore)**	Nicht gut (*)	4	80,0%	0	0,0%
	Durchschnittlich	632	94,3%	29	4,3%
	Sehr gut	347	97,5%	9	2,5%
**Lebensqualität**	Nicht gut (*)	8	80,0%	1	10,0%
	Durchschnittlich	270	93,1%	16	5,5%
	Sehr gut	700	96,6%	20	2,8%
**Lärmempfindlichkeit**	Nicht lärmempfindlich	268	97,5%	7	2,5%
	Durchschnittlich	552	94,5%	24	4,1%
	Sehr lärmempfindlich	163	94,8%	7	4,1%
**Summenscore Lärmcoping**	Coping ausgeprägt	212	87,6%	22	9,1%
	Coping nicht ausgeprägt	770	97,7%	16	2,0%
**Gefühlte Zunahme Lärmbelastung**	Ja	406	93,1%	21	4,8%
	Nein	577	97,0%	17	2,9%
**Gefühlte Abnahme Lärmbelastung**	Ja	58	100,0%	0	0,0%
	Nein	925	95,1%	38	3,9%
**Notwendigkeit (weiterer) Maßnahmen Lärm**	Maßnahmen notwendig	648	93,8%	33	4,8%
	Keine Maßnahmen nötig	189	98,4%	3	1,6%
**Negative Gesundheitswirkungen Lärm**	Gering	216	99,5%	1	0,5%
	Stark	735	94,1%	36	4,6%
**Lautheit Innsbruck im Vergleich zu anderen Städten Österreichs**	Ruhiger	282	96,6%	6	2,1%
	Gleich	528	95,8%	19	3,4%
	Lauter	159	91,4%	13	7,5%
**Einschätzung eigener Lärmbeitrag**	Gering	739	95,4%	28	3,6%
	Durchschnittlich	217	95,6%	9	4,0%
	Groß	27	93,1%	1	3,4%

(*) zu kleine Fallzahlen für solide Auswertungen
Anm.: Die Fallzahlen in der Gruppe der mittelgradig Belästigten (N=38) sind in einigen der obenstehenden Subgruppen zu gering für solide Auswertungen.

11. Belästigung durch Autobahnlärm im Schlaf

Autobahnlärm spielt wie schon bei der Frage nach der Belästigung allgemein auch im Nachtzeitraum für nur sehr wenige befragte Innsbruckerinnen und Innsbrucker eine größere Rolle. Nur rund 1% der 1031 Befragten stört der Autobahnlärm im Schlaf stark (*highly annoyed sleep*: gruppierte Antwortkategorien 8, 9 und 10 [=*belästigt / stört besonders stark*]), knapp 96% aller Befragten sehen sich selbst als nur gering im Schlaf autobahnlärmbelästigt an (gruppierte Antwortkategorien 0 [=*belästigt / stört überhaupt nicht*], 1 und 2), davon fast 92% *überhaupt nicht belästigt*.

Im Mittel liegen die Antworten der befragten Innsbruckerinnen und Innsbrucker bei 0,29 (3,2%) auf der 11-teiligen Antwortskala, was den niedersten Wert aller abgefragten Lärmquellen darstellt:

Abbildung 216: Belästigung durch Autobahnlärm im Schlaf in Prozent

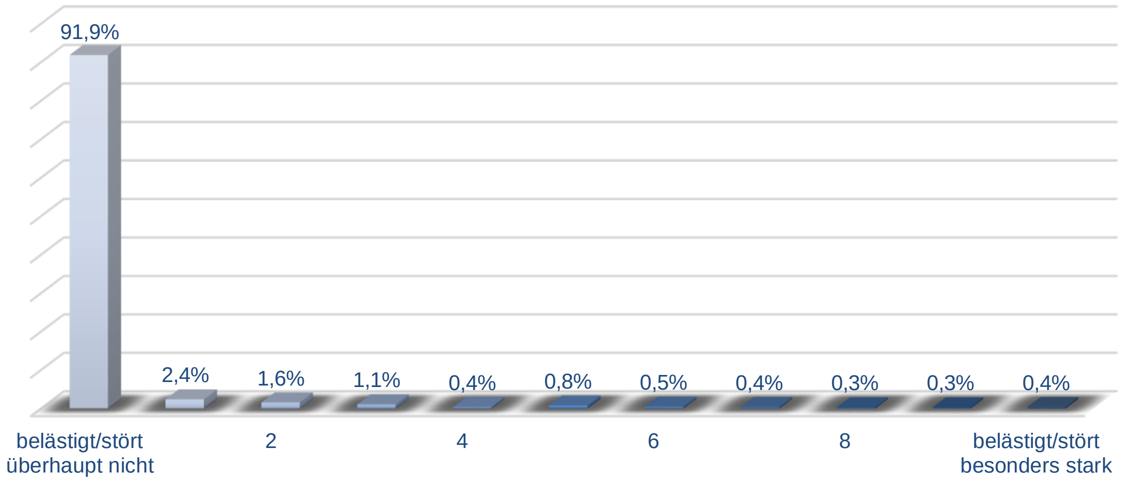

Tabelle 163: Belästigung im Schlaf durch Autobahnlärm gruppiert

		Häufigkeit	Gültige Prozente	Kumulierte Prozente
Gültig	Gering belästigt	989	95,9	95,9
	Mittelgradig belästigt	32	3,1	99,0
	Stark belästigt	10	1,0	100,0
	Gesamt	1031	100,0	

11.1. Belästigung im Schlaf durch Autobahnlärm und Autobahnlärmbelastung

Die Belästigung im Schlaf durch den nächtlichen Lärm von der Autobahn gestaltet sich sehr ähnlich dem Bezug der Belästigungsreaktion zum nächtlichen Straßenverkehrslärm, fällt aber insgesamt (auch in der Belastungsgruppe der Starkbelasteten) geringer aus:

Abbildung 217: Autobahnlärmbelästigung und und nächtliche Autobahnlärmbelastung L_n in 10dB-Schritten im Mittelwertvergleich

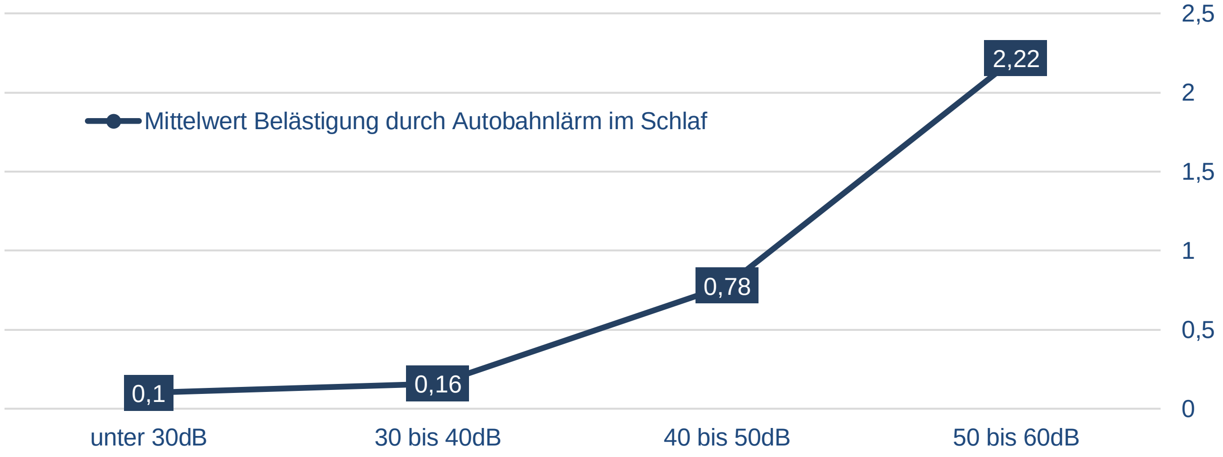

Anm.: Die Darstellung zeigt nachvollziehbare Zusammenhänge auf, enthält jedoch keine Prüfungen auf Kovariablen und andere Prädiktoren und ist somit nicht den Dosis-Wirkungskurven aus der gängigen Fachliteratur gleichzusetzen.

Das Belästigungsausmaß im niederen Belastungsbereich ist sehr gering ausgeprägt, beginnt aber im Vergleich zum 24h-Zeitraum bei rund 10dB niedrigeren Dauerlärmpegeln, ab Belastungen von etwa 40dB L_n, auf niedrigem Niveau anzusteigen.

11.2. Belästigung im Schlaf durch Autobahnlärm und Katastralgemeinden

Die im Mittel stärkste Belästigung im Schlaf durch Autobahnlärm zeigt sich in Wilten und Vill. Die geringste Störung durch Autobahnlärm in der Nacht im eigenen Wohnumfeld empfinden die Befragten in Igls, Mühlau und Pradl, sowie in der Innenstadt (Katastralgemeinde Innsbruck):

Abbildung 218: Belästigung im Schlaf durch Autobahnlärm und Katastralgemeinden im Mittelwertvergleich

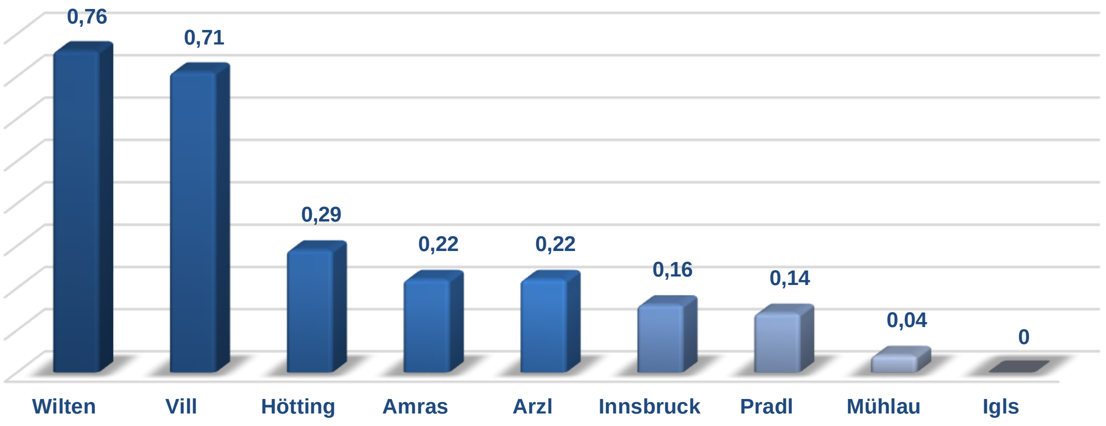

11.3. Belästigung im Schlaf durch Autobahnlärm und einzelne Parameter

Die Auswertungen zur Belästigung im Schlaf durch Autobahnlärm zeigen im Vergleich zu den anderen Belästigungslärmquellen geringere Bezüge zu anderen Parametern. Die Fallzahlen der Mittel- und Starkbelasteten sind sehr klein, weshalb die Aussagekraft der Extremgruppenvergleiche stark eingeschränkt ist. Besonders in der Extremgruppe der Starkbelästigten (*highly annoyed*, N=10) sind zu wenige Fälle für solide Auswertungen enthalten, weshalb diese Gruppe in der untenstehenden Tabelle ganz ausgespart wurde.

Im Folgenden einige ausgewählte Parameter, jeweils in die beiden Gruppen der gering und der mittelgradig Belasteten zusammengefasst, in Zusammenschau mit der Belästigung im Schlaf durch Autobahnlärm:

Tabelle 164: Belästigung im Schlaf durch Autobahnlärm und ausgewählte Parameter

		Belästigung im Schlaf Autobahnlärm gruppiert			
		Gering belästigt		Mittelgradig belästigt	
		Anzahl	Zeilen (%)	Anzahl	Zeilen (%)
Geschlecht	weiblich	522	96,5%	12	2,2%
	männlich	467	95,3%	20	4,1%

**Altersgruppen**	18 bis 40 Jahre	423	96,1%	14	3,2%
	41 bis 60 Jahre	302	95,6%	9	2,8%
	über 60 Jahre	264	96,0%	9	3,3%
**Bildungsschicht**	Primärer Schulabschluss	94	95,9%	3	3,1%
	Sekundärer Schulabschluss	521	96,1%	17	3,1%
	Tertiärer Schulabschluss	371	95,6%	12	3,1%
**Haushalt mit/ohne minderjährige Kinder**	Haushalt ohne Kinder	731	95,9%	25	3,3%
	Haushalt mit Kindern	258	95,9%	7	2,6%
**Baulicher Schutz vor Verkehrslärm**	Keiner bis geringfügig	732	95,6%	25	3,3%
	Gut bis komplett	230	97,5%	6	2,5%
**Innenhof bzw. lärm-abgewandte Seite**	Ja	680	97,0%	17	2,4%
	Nein	289	93,8%	15	4,9%
**Nachtschlaf**	Fenster geöffnet/gekippt	788	96,0%	27	3,3%
	Fenster geschlossen	193	95,5%	5	2,5%
**Wohnbedingungen**	Nicht gut	80	95,2%	1	1,2%
	Durchschnittlich	528	95,1%	22	4,0%
	Sehr gut	379	97,2%	9	2,3%
**Bedingungen für Kinder**	Nicht gut	45	95,7%	1	2,1%
	Durchschnittlich	492	95,5%	19	3,7%
	Sehr gut	431	96,4%	11	2,5%
**Verbundenheit zu Innsbruck**	Nicht verbunden	53	96,4%	1	1,8%
	Durchschnittlich	360	95,0%	14	3,7%
	Sehr verbunden	574	96,5%	17	2,9%
**Zufriedenheit eigene Wohnsituation**	Nicht zufrieden	26	92,9%	0	0,0%
	Durchschnittlich	243	94,2%	11	4,3%
	Sehr zufrieden	719	96,6%	21	2,8%
**Lebensbedingungen (Summenscore)**	Nicht gut (*)	4	80,0%	0	0,0%
	Durchschnittlich	636	94,9%	28	4,2%
	Sehr gut	349	98,0%	4	1,1%
**Lebensqualität**	Nicht gut (*)	10	100,0%	0	0,0%
	Durchschnittlich	275	94,8%	11	3,8%
	Sehr gut	699	96,4%	20	2,8%
**Lärmempfindlichkeit**	Nicht lärmempfindlich	268	97,5%	5	1,8%
	Durchschnittlich	558	95,5%	20	3,4%
	Sehr lärmempfindlich	163	94,8%	7	4,1%
**Summenscore Lärmcoping**	Coping ausgeprägt	220	90,9%	13	5,4%
	Coping nicht ausgeprägt	768	97,5%	19	2,4%
**Gefühlte Zunahme Lärmbelastung**	Ja	411	94,3%	16	3,7%
	Nein	578	97,1%	16	2,7%
**Gefühlte Abnahme Lärmbelastung**	Ja	58	100,0%	0	0,0%
	Nein	931	95,7%	32	3,3%
**Notwendigkeit (weite-rer) Maßnahmen Lärm**	Maßnahmen notwendig	654	94,6%	27	3,9%
	Keine Maßnahmen nötig	189	98,4%	3	1,6%

**Negative Gesund-heitswirkungen Lärm**	Gering	212	97,7%	4	1,8%
	Stark	744	95,3%	28	3,6%
**Lautheit Innsbruck im Vergleich zu anderen Städten Österreichs**	Ruhiger	285	97,6%	5	1,7%
	Gleich	530	96,2%	17	3,1%
	Lauter	161	92,5%	9	5,2%
**Einschätzung eigener Lärmbeitrag**	Gering	749	96,6%	18	2,3%
	Durchschnittlich	215	94,7%	12	5,3%
	Groß	25	86,2%	2	6,9%

(*) zu kleine Fallzahlen für solide Auswertungen

Anm.: Die Fallzahlen in der Gruppe der mittelgradig Belästigten (N=32) sind in einigen der obenstehenden Subgruppen zu gering für solide Auswertungen.

Lärmcoping

1. Überblick und Summenscore

Aus den Auswertungen der vorangegangen Kapitel ist ersichtlich, dass die Wahrnehmung und Beurteilung von (Verkehrs-)Lärm und die daraus resultierenden Belästigungsreaktionen eng mit dem Ausmaß der vorhandenen Lärmbelastung einhergehen. Jedoch auch, dass die gemachten Erfahrungen jedes einzelnen und die daraus resultierenden Adaptations- und Bewältigungsmechanismen (Copingmöglichkeiten) entscheidend für die entwickelten Emotionen und daher die letztendliche persönliche Beurteilung des Ausmaßes des wichtigsten Umweltstressors (Verkehrs-)Lärm sind.

Für die Verursachung von (negativen) gesundheitlich wirksamen Effekten ist nicht allein das Ausmaß der Belastung durch den Stressor Lärm ausschlaggebend, sondern zu einem sehr erheblichen Anteil zunächst die Einschätzung der Belastung (primary appraisal) und schließlich der individuelle Umgang (secondary appraisal) mit demselben.

Lärmcopingmechanismen als Reaktionen zur Bewältigung von Lärmbelastungen wurden in Ansätzen mittels 7 Fragen erhoben (aktives und passives instrumentelles, emotionales und politisches Copingverhalten) und mittels Ja/Nein-Antwortmöglichkeiten abgefragt:

Abbildung 219: Lärmcopingreaktionen im Vergleich in Prozent (nach Häufigkeit absteigend sortiert)

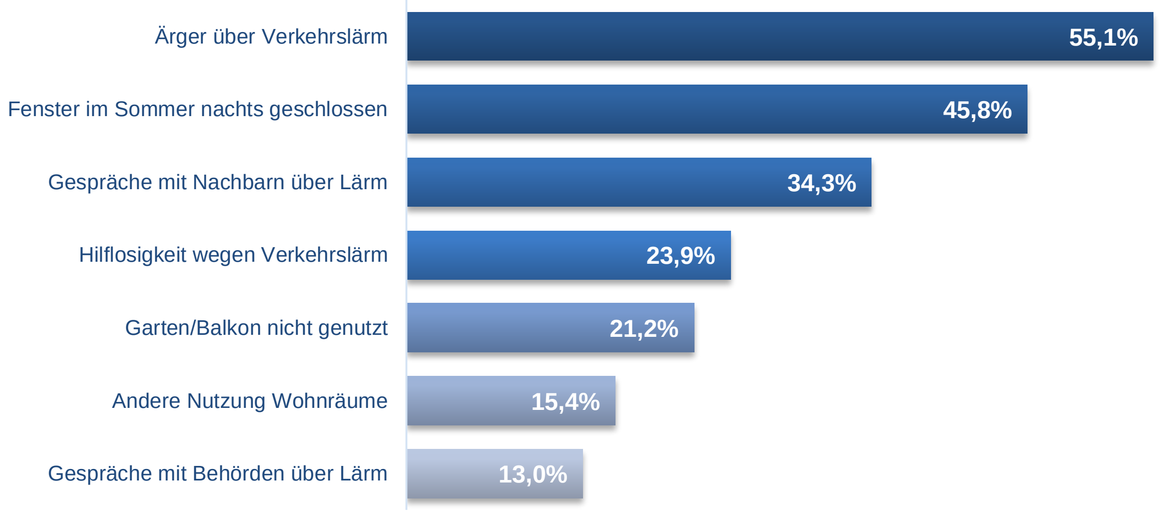

Tabelle 165: Lärmcopingreaktionen im Mittelwertvergleich (absteigend sortiert)

	Ärger über Verkehrslärm	Fenster im Sommer nachts geschlossen	Gespräche mit Nachbarn über Lärm	Hilflosigkeit wegen Verkehrslärm	Garten/Balkon nicht genutzt	Andere Nutzung Wohnräume	Gespräche mit Behörden über Lärm
Mittelwert	1,45	1,54	1,66	1,76	1,79	1,85	1,87
N	1031	1031	1031	1031	1030	1031	1031
Standardabweichung	0,498	0,498	0,475	0,426	0,409	0,361	0,336

Über alle 7 Fragen hinweg (Summenscore) zeigen 23,5% der Befragten ein ausgeprägtes Lärmadaptationsverhalten, rund drei Viertel nicht. Eine Person macht keine Angaben:

Abbildung 220: Summenscore Lärmcoping in Prozent

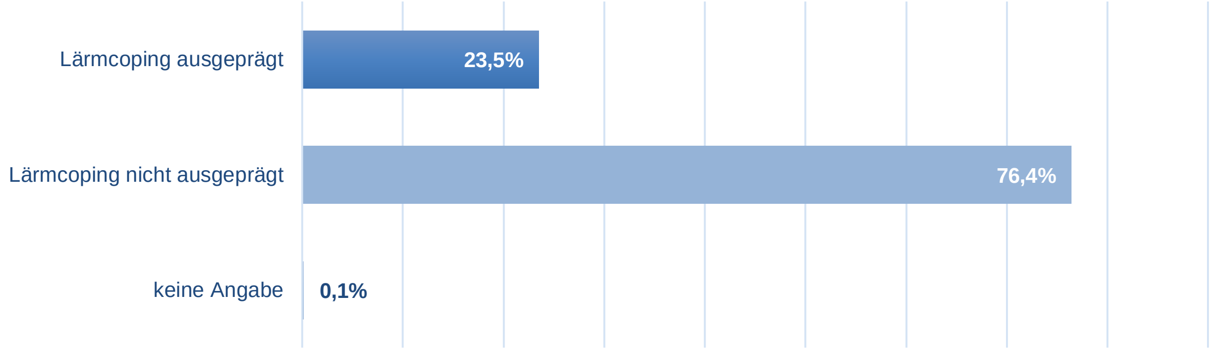

2. Instrumentelles Lärmcoping

Fast die Hälfte (45,8%) aller befragten Innsbruckerinnen und Innsbrucker hat schon zumindest einmal aufgrund der Lärmbelästigung ihre Fenster auch im Sommer nachts geschlossen. Über 21% haben ihren Garten oder Balkon aus diesem Grund nicht genutzt und über 15% im untersuchten Kollektiv haben schon zumindest einmal ihre Wohnräume anders genutzt, um der Belästigung durch Lärm zu entgehen:

Abbildung 221: Instrumentelles Lärmcoping in Prozent

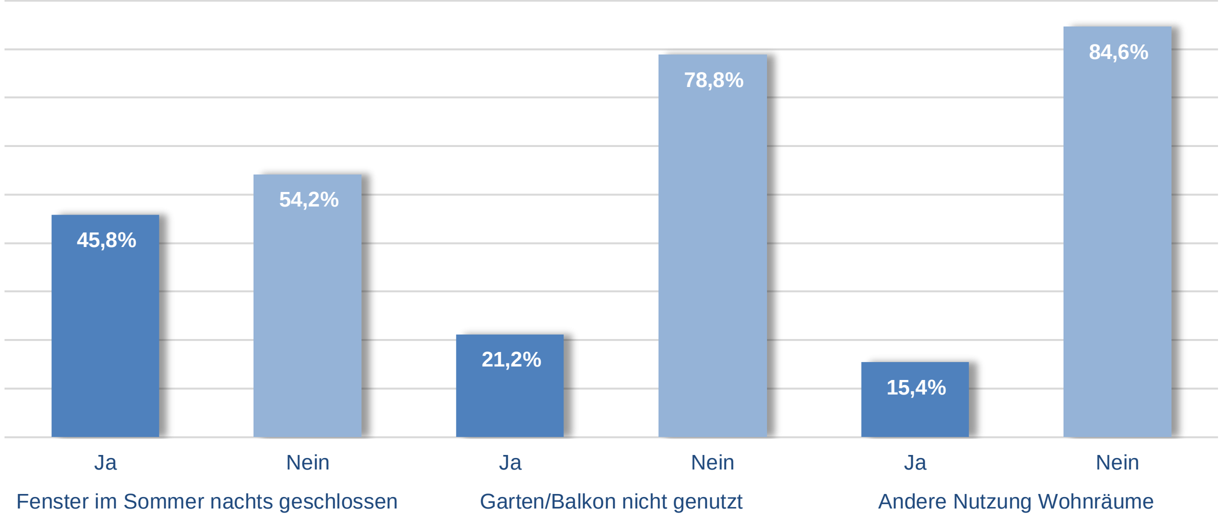

2.1. Instrumentelles Lärmcoping und ausgewählte Parameter

Eine Reihe von Faktoren zeigt Zusammenhänge zum instrumentellen Lärmcoping. Unter anderem die vorhandene Lärmbelastung, die Lärmempfindlichkeit und das Gefühl der Ausgesetztheit gegenüber Verkehrslärm, sowie das Belästigungsempfinden.

Sehr deutlich treten die Unterschiede im Verhalten der befragten Innsbruckerinnen und Innsbrucker auch in Abhängigkeit von baulichem Lärmschutz in deren Wohnumfeld zutage:

Abbildung 222: Instrumentelles Lärmcoping und baulicher Schutz vor Verkehrslärm (gruppiert)

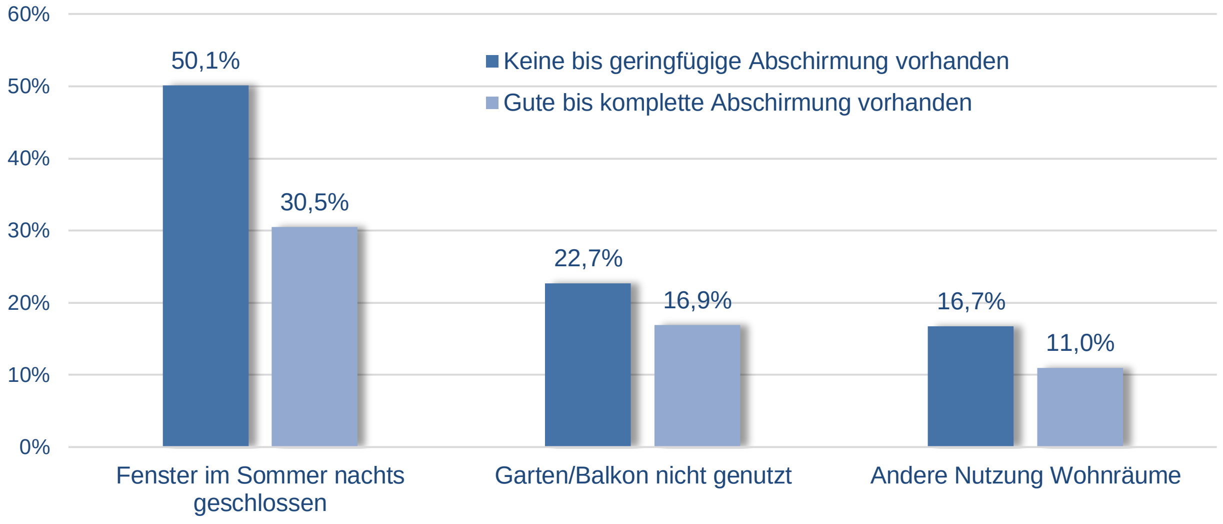

3. Emotionales Lärmcoping

Eine Mehrheit der Befragten (55,1%) hat sich schon zumindest einmal über den Verkehrslärm geärgert, knapp ein Viertel sich hilflos gefühlt und über ein Drittel mit Nachbarn über die Lärmbelastungen gesprochen:

Abbildung 223: Emotionales Lärmcoping in Prozent

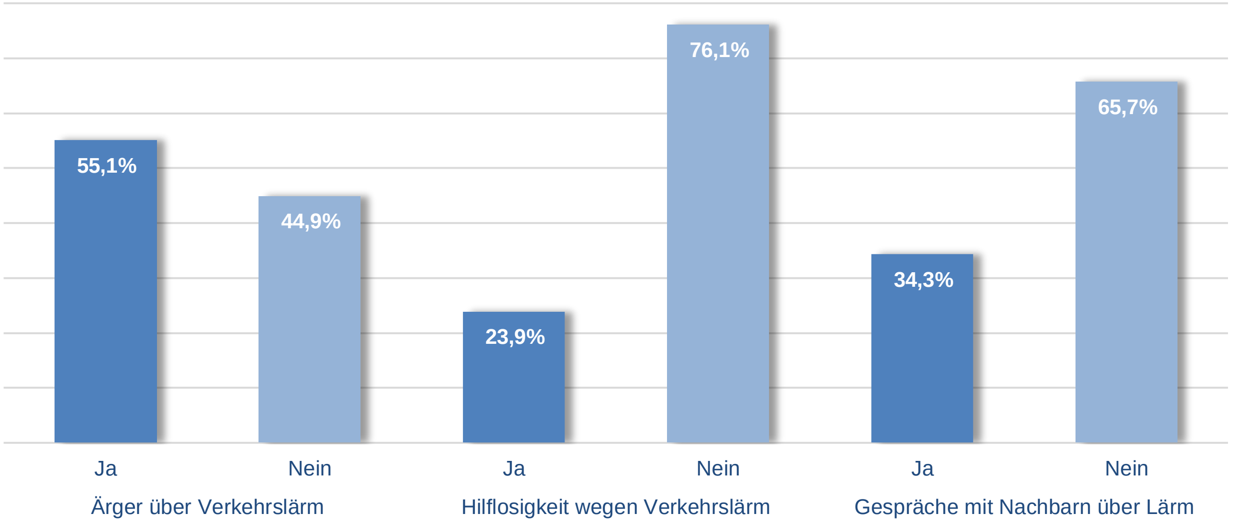

3.1. Emotionales Lärmcoping und Lärmbelastung

In der Zusammenschau mit den gegebenen Verkehrslärmpegeln werden sehr deutliche Unterschiede zwischen den einzelnen Belastungsgruppen sichtbar: Knapp ein Drittel der vom 24h-Straßenlärm stark Belasteten hat sich schon zumindest einmal hilflos wegen dem Verkehrslärm gefühlt. In der Gruppe der vom Straßenlärm gering belasteten Befragten sind es etwas weniger als 9%.

Beim Flug- und Schienenverkehrslärm fällt auf, dass die Gruppe der gering Belasteten deutlich stärker reagiert, als auf die Lärmbelastung durch den Straßenverkehr und beim Fluglärm die mittelgradig belasteten Befragten sogar häufiger emotionales Coping zeigen, als die Starkbelasteten. Die Unterschiede zwischen den einzelnen Belastungsgruppen fallen beim Flug- und Schienenverkehrslärm insgesamt geringer aus:

Tabelle 166: Emotionales Lärmcoping und Lärmbelastung nach Quelle (gruppiert)

		Ärger über Verkehrslärm empfunden		Hilflosigkeit wegen Verkehrslärm gefühlt		Gespräche mit Nachbarn über Lärm geführt	
		Anzahl	Zeilen (%)	Anzahl	Zeilen (%)	Anzahl	Zeilen (%)
**Straßenlärm 24h**	Stark belastet >55dB	338	65,5%	170	32,9%	201	39,0%
	Mittelgradig belastet 45-55dB	200	49,6%	66	16,4%	137	34,0%
	Gering belastet <45dB	30	26,8%	10	8,9%	16	14,3%
**Fluglärm 24h**	Stark belastet >55dB	59	52,7%	25	22,3%	40	35,7%
	Mittelgradig belastet 45-55dB	233	64,7%	102	28,3%	139	38,6%
	Gering belastet <45dB	276	49,4%	119	21,3%	175	31,3%
**Schienenlärm 24h**	Stark belastet >55dB	78	67,2%	41	35,3%	45	38,8%
	Mittelgradig belastet 45-55dB	181	56,6%	79	24,7%	120	37,5%
	Gering belastet <45dB	309	51,9%	126	21,2%	189	31,8%

In der Detailbetrachtung wird ersichtlich, dass beim Flug- und Schienenverkehrslärm bereits ab Lärmpegeln über 40dB die Häufigkeit des Ärgers über die Lärmbelastung deutlich höher liegt als beim Straßenverkehrslärm. Bei starken Dauerlärmbelastungen mit Beurteilungspegeln über 50dB sind die Unterschiede zwischen den Verkehrslärmquellen hingegen sehr gering ausgeprägt:

Abbildung 224: Ärger über Verkehrslärm und 24h-Lärmbelastung L_{den} nach Quelle in 10dB-Schritten im Mittelwertvergleich

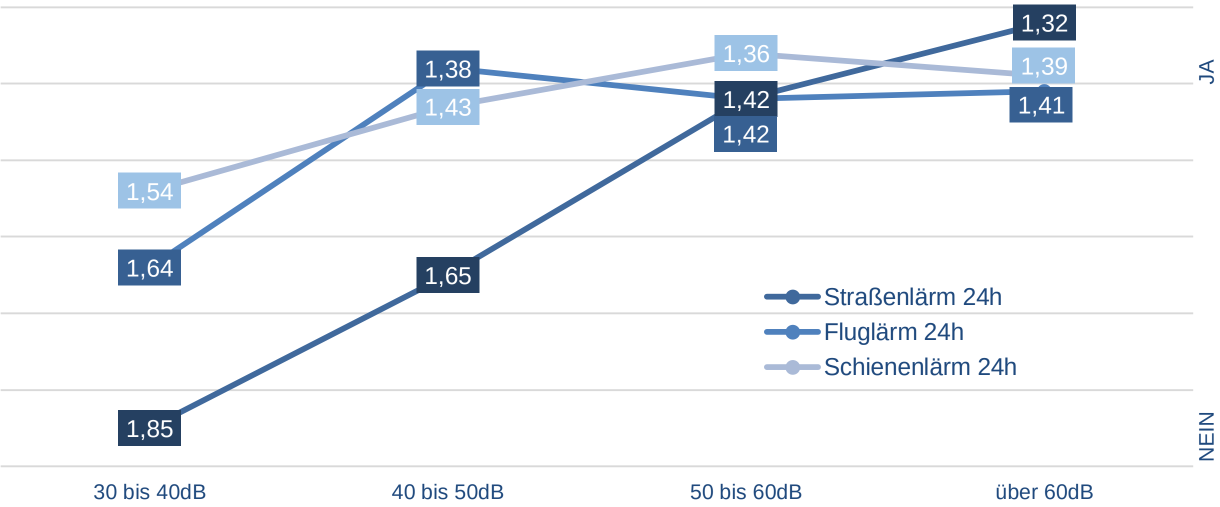

3.2. Emotionales Lärmcoping und ausgewählte Parameter

Die jüngste Altersgruppe ärgert sich häufiger über den Verkehrslärm als die beiden anderen Altersgruppen, fühlt sich jedoch seltener hilflos und führt auch seltener Gespräche mit den Nachbarn darüber.

Frauen copen etwas häufiger emotional gegen den Verkehrslärm an als Männer.

Eine sehr ausgeprägte Beziehung besteht zu den Faktoren Beurteilung der eigenen Wohnsituation, Einschätzung der eigenen Lebensqualität und Lärmempfindlichkeit, sowie besonders stark zur empfundenen Lärmexposition und –Belästigung.

Abbildung 225: Emotionales Lärmcoping und Lärmempfindlichkeit (gruppiert)

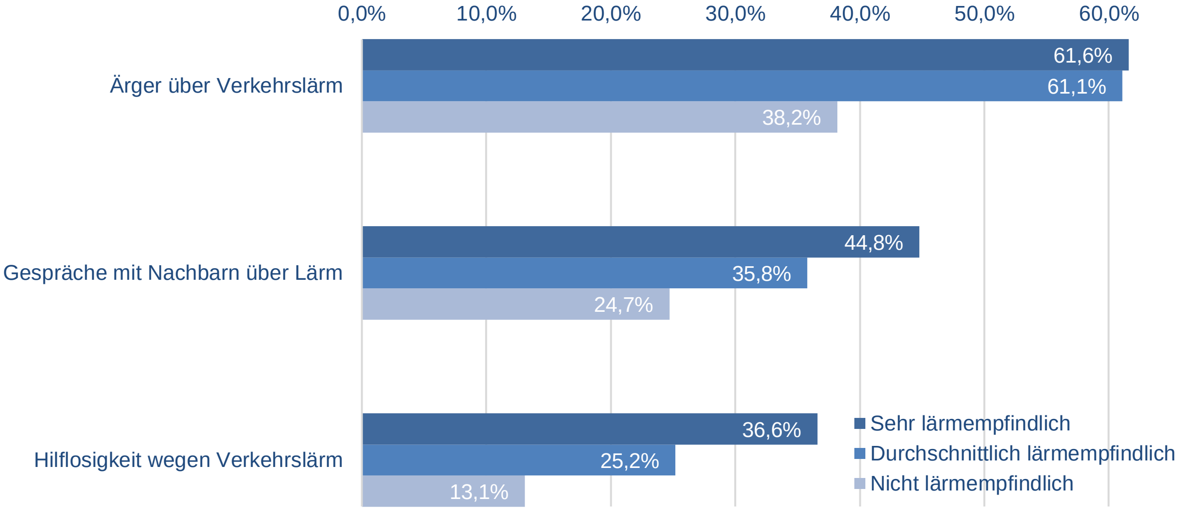

Den Spitzenwert zeigt die Gruppe der sich stark vom Lärm insgesamt belästigt fühlenden Befragten, welche sich zu knapp 93% schon zumindest einmal über den Verkehrslärm geärgert, zu über 60% schon zumindest einmal hilflos gefühlt und zu über 65% Gespräche mit Nachbarn über die Lärmbelastungen geführt haben:

Tabelle 167: Emotionales Lärmcoping und ausgewählte Parameter (gruppiert)

		Ärger über Verkehrslärm empfunden		Hilflosigkeit wegen Verkehrslärm gefühlt		Gespräche mit Nachbarn über Lärm geführt	
		Anzahl	Zeilen (%)	Anzahl	Zeilen (%)	Anzahl	Zeilen (%)
**Geschlecht**	weiblich	307	56,7%	145	26,8%	206	38,1%
	männlich	261	53,3%	101	20,6%	148	30,2%
**Altersgruppen**	18 bis 40 Jahre	265	60,2%	94	21,4%	122	27,7%
	41 bis 60 Jahre	173	54,7%	82	25,9%	131	41,5%
	über 60 Jahre	130	47,3%	70	25,5%	101	36,7%
**Wohnsituation**	Nicht zufrieden	23	82,1%	16	57,1%	17	60,7%
	Durchschnittlich	189	73,3%	94	36,4%	117	45,3%
	Sehr zufrieden	355	47,7%	136	18,3%	219	29,4%
**Lärmempfindlichkeit**	Nicht lärmempfindlich	105	38,2%	36	13,1%	68	24,7%
	Durchschnittlich	357	61,1%	147	25,2%	209	35,8%

	Sehr lärmempfindlich	106	61,6%	63	36,6%	77	44,8%
**Lebensqualität**	Nicht gut	8	80,0%	6	60,0%	5	50,0%
	Durchschnittlich	188	64,8%	94	32,4%	128	44,1%
	Sehr gut	369	50,9%	146	20,1%	220	30,3%
**Ausgesetztheit Lärm gesamt**	Gering exponiert	68	26,1%	13	5,0%	45	17,2%
	Mittelgradig exponiert	382	60,6%	152	24,1%	223	35,4%
	Stark exponiert	118	84,3%	81	57,9%	86	61,4%
**Belästigung Lärm gesamt**	Gering belästigt	98	25,9%	24	6,3%	53	14,0%
	Mittelgradig belästigt	354	67,0%	146	27,7%	219	41,5%
	Stark belästigt	116	92,8%	76	60,8%	82	65,6%

4. Politisches Lärmcoping

13% aller befragten Probandinnen und Probanden haben schon zumindest einmal mit Vertreterinnen und Vertretern der Stadt oder anderen Behörden über die Lärmbelastungen gesprochen:

Abbildung 226: Politisches Lärmcoping in Prozent

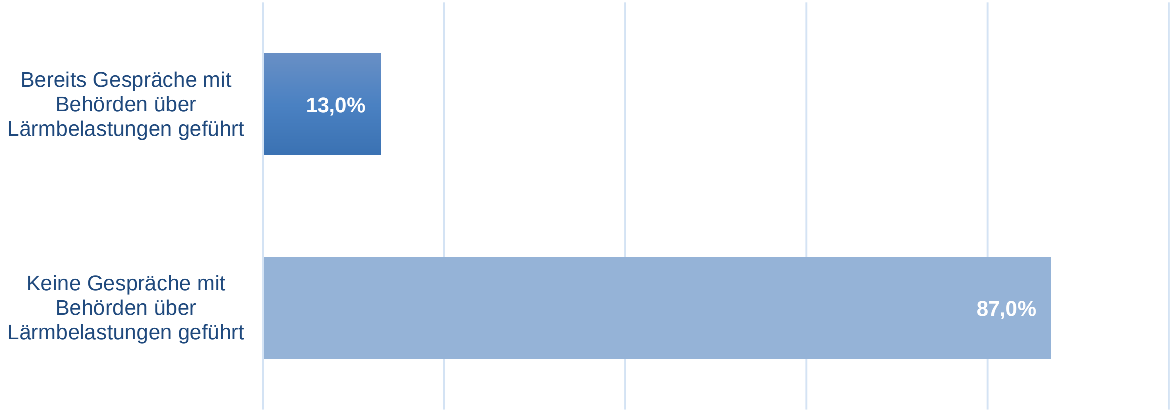

4.1. Politisches Lärmcoping und ausgewählte Parameter

Der Einfluss des Ausmaßes der gegebenen Lärmbelastungen auf das Eintreten von politischem Lärmcoping ist deutlich weniger einheitlich als die persönliche Disposition und Einschätzung der Lärmbelastungen. So zeigen vor allem sich stärker dem Lärm ausgesetzt empfindende und stärker lärmbelästigte Befragte ein aktives politisches Copingverhalten. Dieses ist in etwa doppelt so ausgeprägt wie im Durchschnitt aller Befragten und um ein Vielfaches höher als bei gering exponierten / belästigten Befragten.

Auch die Einschätzung der eigenen Wohn- und Lebensbedingungen, der Wohnsituation an sich, der eigenen Lärmempfindlichkeit und der Güte der eigenen Lebensqualität zeigen sehr ausgeprägte Bezüge zur Bereitschaft mit Behördenvertreterinnen und –Vertretern über den Lärm zu sprechen.

Zwischen den Geschlechtern besteht (bereinigt) kein Unterschied in der Häufigkeit der Befragten, welche bereits Gespräche mit Behörden über die Lärmbelastungen geführt haben.

Jüngere haben etwas weniger häufig als ältere Befragte schon zumindest einmal mit Behörden über den Lärm gesprochen:

Tabelle 168: Politisches Lärmcoping und ausgewählte Parameter (gruppiert)

		Gespräche mit Behörden über Lärm			
		Ja		Nein	
		Anzahl	Zeilen (%)	Anzahl	Zeilen (%)
**Geschlecht**	weiblich	72	13,3%	469	86,7%
	männlich	62	12,7%	428	87,3%
**Altersgruppen**	18 bis 40 Jahre	39	8,9%	401	91,1%
	41 bis 60 Jahre	49	15,5%	267	84,5%
	über 60 Jahre	46	16,7%	229	83,3%
**Wohnsituation**	Nicht zufrieden	9	32,1%	19	67,9%
	Durchschnittlich	54	20,9%	204	79,1%
	Sehr zufrieden	71	9,5%	673	90,5%
**Lärmempfindlichkeit**	Nicht lärmempfindlich	20	7,3%	255	92,7%
	Durchschnittlich	80	13,7%	504	86,3%
	Sehr lärmempfindlich	34	19,8%	138	80,2%
**Lebensqualität**	Nicht gut	6	60,0%	4	40,0%
	Durchschnittlich	57	19,7%	233	80,3%
	Sehr gut	71	9,8%	654	90,2%
**Ausgesetztheit Lärm gesamt**	Gering exponiert	9	3,4%	252	96,6%
	Mittelgradig exponiert	85	13,5%	545	86,5%
	Stark exponiert	40	28,6%	100	71,4%
**Belästigung Lärm gesamt**	Gering belästigt	17	4,5%	361	95,5%
	Mittelgradig belästigt	73	13,8%	455	86,2%
	Stark belästigt	44	35,2%	81	64,8%

5. Summenscore Lärmcoping und ausgewählte Parameter

Insgesamt kann zu den einzelnen Lärmcopingfragen zusammenfassend ausgesagt werden:

- Rund ein Viertel der Befragten zeigt Lärmbewältigungsmechanismen, zwei Drittel hingegen nicht.
- Das Belästigungsempfinden durch Verkehrslärm zeigt die stärksten Bezüge zum Lärmcopingverhalten der befragten Innsbruckerinnen und Innsbrucker.
- Ebenso besteht ein starker Zusammenhang zwischen dem Ausmaß der empfundenen eigenen Lärmexposition, der Einschätzung der eigenen Lärmempfindlichkeit, der Beurteilung der Wohn- und Lebensbedingungen in Innsbruck und in der eigenen Wohnung zur Häufigkeit der angewandten Verhaltensweisen gegenüber dem (Verkehrs-)Lärm.
- Höhere gegebene Verkehrslärmbelastungen führen zu deutlich steigendem Lärmcopingverhalten:

Abbildung 227: Summenscore Lärmcoping und 24h-Lärmbelastung L_{den} nach Quelle in 10dB-Schritten im Mittelwertvergleich

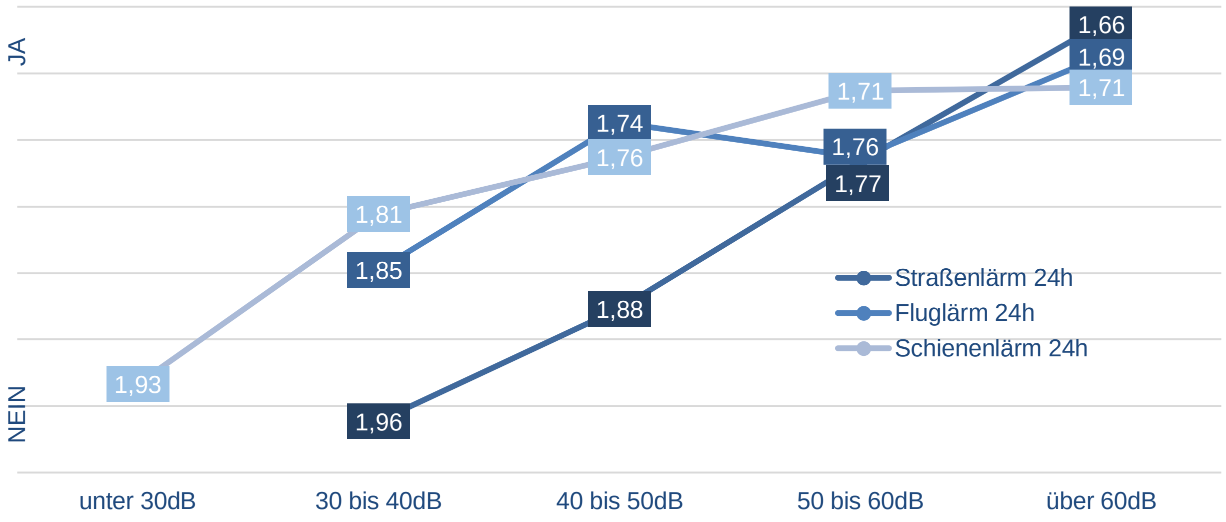

Anm.: Die Belastungsklasse unter 30dB L_{den} umfasst für den Straßenverkehrslärm nur 2 Befragte und ist für den Flugverkehrslärm nicht vorhanden.

- Weibliche Befragte reagieren geringfügig häufiger auf Lärm als Männer.
- Die jüngsten Befragten zeigen das geringste Lärmbewältigungsverhalten, die mittlere Altersklasse der 41- bis 60-Jährigen das am deutlichsten ausgeprägte.
- Je höher der erreichte höchste Schulabschluss, desto häufiger geben Befragte an, Lärmbewältigungsmechanismen einzusetzen.
- Befragte, welche durch bauliche Abschirmungen (Gebäude, Mauern, Innenhöfe …) vor Verkehrslärm (zum Teil) geschützt wohnen, haben weniger Lärmcopingbedarf.
- Befragte, welche der Meinung sind, in Innsbruck sei es lauter oder gleich laut wie in anderen vergleichbaren Städten Österreichs, zeigen ein stärkeres Läermadaptationsverhalten, als Befragte, die glauben, dass es in Innsbruck ruhiger als in anderen Städten sei.

Tabelle 169: Summenscore Lärmcoping und ausgewählte Parameter (gruppiert)

		Summenscore Lärmcoping			
		Lärmcoping ausgeprägt		Lärmcoping nicht ausgeprägt	
		Anzahl	Zeilen (%)	Anzahl	Zeilen (%)
**Geschlecht**	weiblich	139	25,7%	401	74,3%
	männlich	103	21,0%	387	79,0%
**Altersgruppen**	18 bis 40 Jahre	90	20,5%	350	79,5%
	41 bis 60 Jahre	88	27,9%	227	72,1%
	über 60 Jahre	64	23,3%	211	76,7%
**Bildungsschicht**	Primärer Schulabschluss	16	16,3%	82	83,7%
	Sekundärer Schulabschluss	114	21,0%	428	79,0%
	Tertiärer Schulabschluss	111	28,7%	276	71,3%
**Baulicher Schutz vor Verkehrslärm**	Nicht bis kaum vorhanden	199	26,0%	566	74,0%
	Vorhanden	38	16,1%	198	83,9%

**Straßenlärm 24h**	Stark belastet >55dB	158	30,7%	357	69,3%
	Mittelgradig belastet 45-55dB	75	18,6%	328	81,4%
	Gering belastet <45dB	9	8,0%	103	92,0%
**Schienenlärm 24h**	Stark belastet >55dB	36	31,0%	80	69,0%
	Mittelgradig belastet 45-55dB	81	25,3%	239	74,7%
	Gering belastet <45dB	125	21,0%	469	79,0%
**Fluglärm 24h**	Stark belastet >55dB	27	24,1%	85	75,9%
	Mittelgradig belastet 45-55dB	100	27,9%	259	72,1%
	Gering belastet <45dB	115	20,6%	444	79,4%
**Lautheit Innsbruck im Vergleich zu anderen Städten Österreichs**	Ruhiger	56	19,2%	236	80,8%
	Gleich	120	21,8%	431	78,2%
	Lauter	66	38,2%	107	61,8%
**Lärmempfindlichkeit**	Nicht lärmempfindlich	34	12,4%	241	87,6%
	Durchschnittlich	145	24,9%	438	75,1%
	Sehr lärmempfindlich	63	36,6%	109	63,4%
**Ausgesetztheit Lärm gesamt**	Gering exponiert	15	5,7%	246	94,3%
	Mittelgradig exponiert	151	24,0%	479	76,0%
	Stark exponiert	76	54,7%	63	45,3%

6. Summenscore Lärmcoping und Lärmbelästigung

Noch stärker als in Zusammenschau mit der gegebenen Lärmbelastung steigt die Häufigkeit der eingesetzten Lärmadaptationsmechanismen mit zunehmender empfundener Lärmbelästigung:

Abbildung 228: Summenscore Lärmcoping und Belästigung durch Lärm gesamt im Mittelwertvergleich

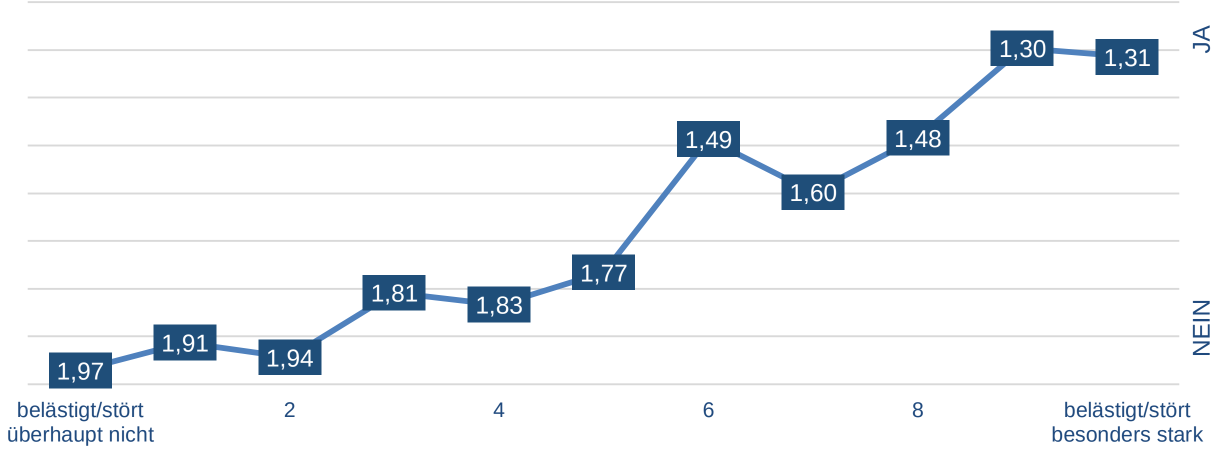

Die einzelnen Quellen der Verkehrslärmbelästigung (Straßen-, Flug- oder Schienenverkehrslärm) zeigen im Summenscore keine ausgeprägten Abweichungen voneinander.

Ähnliches gilt für die nicht verkehrsverursachte Lärmbelästigung. Im Vergleich der Extremgruppen fällt bei der Belästigung durch Erschütterungen, Nachbarschaftslärm und Lärm aus Gewerbe und Industrie ein stärker ausgeprägtes Lärmcopingverhalten als bei den anderen Belästigungsquellen auf:

Tabelle 170: Summenscore Lärmcoping und Lärmbelästigung (gruppiert)

		Summenscore Lärmcoping			
		Lärmcoping ausgeprägt		Lärmcoping nicht ausgeprägt	
		Anzahl	Zeilen (%)	Anzahl	Zeilen (%)
**Belästigung im Schlaf Lärm gesamt**	Gering belästigt	75	10,8%	620	89,2%
	Mittelgradig belästigt	112	43,8%	144	56,3%
	Stark belästigt	55	69,6%	24	30,4%
**Belästigung Lärm gesamt**	Gering belästigt	20	5,3%	358	94,7%
	Mittelgradig belästigt	147	27,8%	381	72,2%
	Stark belästigt	75	60,5%	49	39,5%
**Belästigung Fluglärm**	Gering belästigt	82	16,1%	428	83,9%
	Mittelgradig belästigt	98	24,6%	301	75,4%
	Stark belästigt	62	51,2%	59	48,8%
**Belästigung Straßenverkehrslärm**	Gering belästigt	73	13,5%	466	86,5%
	Mittelgradig belästigt	101	26,9%	274	73,1%
	Stark belästigt	68	58,6%	48	41,4%
**Belästigung Schienenverkehrslärm**	Gering belästigt	166	19,8%	673	80,2%
	Mittelgradig belästigt	60	37,3%	101	62,7%
	Stark belästigt	16	53,3%	14	46,7%
**Belästigung Baustellenlärm**	Gering belästigt	112	16,7%	558	83,3%
	Mittelgradig belästigt	87	33,0%	177	67,0%
	Stark belästigt	43	45,3%	52	54,7%
	w.n. / k.A.	0	0,0%	1	100,0%
**Belästigung Nachbarschaftslärm**	Gering belästigt	125	17,0%	611	83,0%
	Mittelgradig belästigt	83	34,2%	160	65,8%
	Stark belästigt	34	66,7%	17	33,3%
**Belästigung Lärm von Passanten und Gaststätten**	Gering belästigt	121	16,2%	627	83,8%
	Mittelgradig belästigt	92	39,7%	140	60,3%
	Stark belästigt	29	58,0%	21	42,0%
**Belästigung Erschütterungen**	Gering belästigt	156	18,1%	706	81,9%
	Mittelgradig belästigt	64	47,4%	71	52,6%
	Stark belästigt	22	66,7%	11	33,3%
**Belästigung Lärm aus Gewerbe u. Industrie**	Gering belästigt	189	20,2%	747	79,8%
	Mittelgradig belästigt	41	51,9%	38	48,1%
	Stark belästigt	12	80,0%	3	20,0%
**Belästigung Autobahnlärm**	Gering belästigt	209	22,1%	737	77,9%
	Mittelgradig belästigt	20	32,3%	42	67,7%
	Stark belästigt	13	59,1%	9	40,9%

Fühlen sich Befragte durch Lärm in der Nacht im Schlaf gestört, fallen die Lärmbewältigungsmechanismen stärker aus und werden bereits bei niedrigeren Belästigungsausmaßen gesetzt als in der Zusammenschau mit der Belästigung durch Lärm insgesamt:

Abbildung 229: Summenscore Lärmcoping und Belästigung durch Lärm gesamt im Schlaf im Mittelwertvergleich

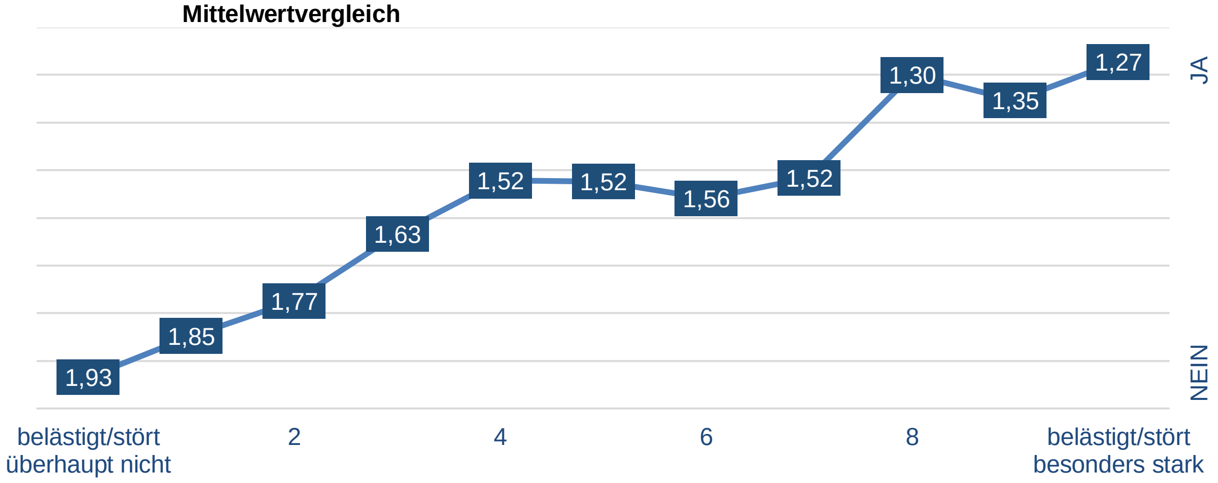

Empfundene Zu- oder Abnahme des Lärms in Innsbruck

1. Lärmbelastungsveränderung im Überblick

Eine deutliche Mehrheit der befragten Innsbruckerinnen und Innsbrucker ist der Meinung, dass die Lärmbelastungen in der Stadt in den letzten Jahren nicht zugenommen haben (57,7%), über 42% hingegen gehen davon aus, dass die Lärmbelastungen zugenommen haben.

Mit knapp 95% relativ einig sind sich die Befragten dahingehend, dass die Lärmbelastungen in den letzten Jahren nicht abgenommen haben:

Abbildung 230: Empfundene Zu- und Abnahme der Lärmbelastung in Prozent

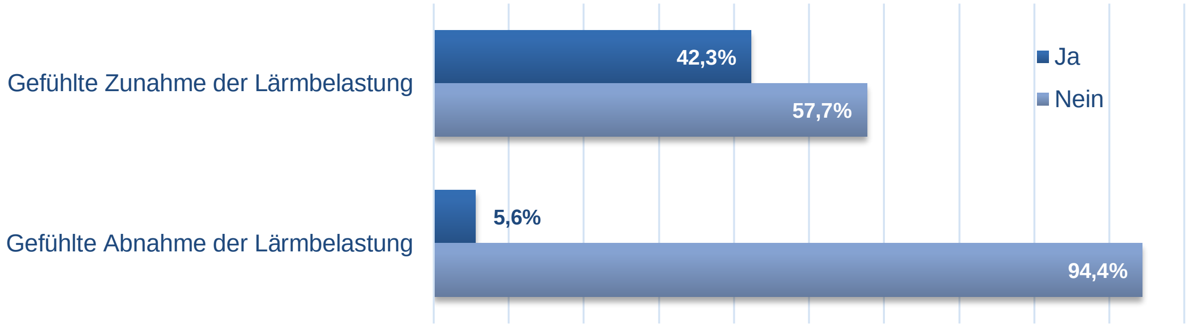

2. Lärmbelastungsveränderung und ausgewählte Parameter

Frauen sind deutlich häufiger als Männer der Meinung, die Lärmbelastung habe in den letzten Jahren zugenommen.

Die jüngste Gruppe der Befragten erkennt zu 70% keine Lärmzunahmen in Innsbruck.

Der höchste erreichte Schulabschluss spielt keine zentrale Rolle in der Einschätzung der Zu- oder Abnahme der Lärmbelastungen.

Befragte, die bei offenem oder gekippten Fenster schlafen, über einen Innenhof bzw. eine lärmabgewandte Seite verfügen, oder in ihrem Wohnumfeld durch bauliche Gegebenheiten vor Verkehrslärm geschützt sind, gehen seltener von einer Zunahme der Lärmbelastungen aus, als Innsbruckerinnen und Innsbrucker, deren Wohnung weniger lärmgeschützt sind und die bei geschlossenem Fenster schlafen:

Tabelle 171: Empfundene Zu- und Abnahme der Lärmbelastung und ausgewählte Parameter

		Gefühlte Zunahme Lärmbelastung				Gefühlte Abnahme Lärmbelastung			
		Ja		Nein		Ja		Nein	
		Anzahl	Prozent	Anzahl	Prozent	Anzahl	Prozent	Anzahl	Prozent
**Geschlecht**	weiblich	250	46,2%	291	53,8%	27	5,0%	514	95,0%
	männlich	186	38,0%	304	62,0%	31	6,3%	459	93,7%
**Altersgruppen**	18 bis 40 Jahre	132	30,0%	308	70,0%	23	5,2%	417	94,8%
	41 bis 60 Jahre	167	52,8%	149	47,2%	17	5,4%	299	94,6%

	über 60 Jahre	137	49,8%	138	50,2%	18	6,5%	257	93,5%
**Bildungsschicht**	Primär	40	40,8%	58	59,2%	11	11,2%	87	88,8%
	Sekundär	234	43,2%	308	56,8%	30	5,5%	512	94,5%
	Tertiär	162	41,8%	226	58,2%	17	4,4%	371	95,6%
**Baulicher Schutz vor Verkehrslärm**	Kaum vorhanden	344	44,9%	422	55,1%	38	5,0%	728	95,0%
	Vorhanden	81	34,3%	155	65,7%	20	8,5%	216	91,5%
**Innenhof bzw. lärm-abgewandte Seite**	Ja	280	39,9%	421	60,1%	46	6,6%	655	93,4%
	Nein	147	47,7%	161	52,3%	12	3,9%	296	96,1%
**Nachtschlaf**	Fenster geöffnet	334	40,7%	487	59,3%	46	5,6%	775	94,4%
	Fenster geschlossen	99	49,0%	103	51,0%	12	5,9%	190	94,1%
**Straßenlärm 24h**	Stark belastet >55dB	251	48,6%	265	51,4%	27	5,2%	489	94,8%
	Mittelgradig 45-55dB	156	38,7%	247	61,3%	29	7,2%	374	92,8%
	Geringbelastet <45dB	29	25,9%	83	74,1%	2	1,8%	110	98,2%
**Schienenlärm 24h**	Stark belastet >55dB	40	34,5%	76	65,5%	12	10,3%	104	89,7%
	Mittelgradig 45-55dB	153	47,8%	167	52,2%	22	6,9%	298	93,1%
	Geringbelastet <45dB	243	40,8%	352	59,2%	24	4,0%	571	96,0%
**Fluglärm 24h**	Stark belastet >55dB	44	39,3%	68	60,7%	5	4,5%	107	95,5%
	Mittelgradig 45-55dB	165	45,8%	195	54,2%	17	4,7%	343	95,3%
	Geringbelastet <45dB	227	40,6%	332	59,4%	36	6,4%	523	93,6%
**Lebensbedingungen (Summenscore)**	Nicht gut	5	100,0%	0	0,0%	0	0,0%	5	100,0%
	Durchschnittlich	306	45,7%	364	54,3%	42	6,3%	628	93,7%
	Sehr gut	125	35,1%	231	64,9%	16	4,5%	340	95,5%
**Lebensqualität**	Nicht gut	8	80,0%	2	20,0%	0	0,0%	10	100,0%
	Durchschnittlich	152	52,4%	138	47,6%	18	6,2%	272	93,8%
	Sehr gut	276	38,1%	449	61,9%	39	5,4%	686	94,6%
**Lautheit Innsbruck im Vergleich zu anderen Städten**	Ruhiger	95	32,5%	197	67,5%	23	7,9%	269	92,1%
	Gleich	247	44,8%	304	55,2%	25	4,5%	526	95,5%
	Lauter	91	52,3%	83	47,7%	10	5,7%	164	94,3%
**Notwendigkeit (weite-rer) Maßnahmen zur Lärmreduktion**	Ja	356	51,5%	335	48,5%	38	5,5%	653	94,5%
	Nein	44	22,9%	148	77,1%	13	6,8%	179	93,2%
**Lärmempfindlichkeit**	Nicht lärmempfindlich	90	32,7%	185	67,3%	20	7,3%	255	92,7%
	Durchschnittlich	257	44,0%	327	56,0%	33	5,7%	551	94,3%
	Sehr lärmempfindlich	89	51,7%	83	48,3%	5	2,9%	167	97,1%
**Ausgesetztheit Lärm gesamt**	Gering exponiert	56	21,5%	205	78,5%	17	6,5%	244	93,5%
	Mittelgradig exponiert	285	45,2%	345	54,8%	37	5,9%	593	94,1%
	Stark exponiert	95	67,9%	45	32,1%	4	2,9%	136	97,1%

Anm.: Die Fallzahlen in der Gruppe von Befragten, die von einer Abnahme der Lärmbelastung in den letzten Jahren ausgehen, sind in einigen der obenstehenden Subgruppen zu gering für solide Auswertungen.

Die gegebene Lärmbelastung durch die Verkehrsträger zeigt keine ausgeprägten Beziehungen zur Einschätzung der Befragten, ob sich die Lärmbelastung in Innsbruck in den vergangenen Jahren verändert hat. Deutlich stärker korrespondieren Faktoren wie die Einschätzung der eigenen Lebensquali-

tät, die Lebens- und Wohnbedingungen, die eigene Lärmempfindlichkeit und das Gefühl der Ausgesetztheit gegenüber Lärm.

Personen, die davon ausgehen, dass es in Innsbruck ruhiger als in anderen vergleichbaren Städten Österreichs ist und die keine (weiteren) Maßnahmen zur Lärmreduktion für angemessen halten, gehen zu mehr als zwei Dritteln davon aus, dass es in den vergangenen Jahren nicht lauter geworden ist.

3. Lärmbelastungsveränderung und Lärmbelästigung

Die empfundene Störung durch Lärm zeigt mit steigendem Ausmaß unabhängig von der Quelle der Lärmbelästigung in Zusammenschau mit der Einschätzung der Lärmbelastungsveränderung in Innsbruck sehr einheitlich eine deutlich steigende Tendenz der Befragten, von einer gefühlten Zunahme der Lärmbelastung auszugehen:

Abbildung 231: Empfundene Zunahme der Lärmbelastung und Belästigung durch Lärm insgesamt im Mittelwertvergleich

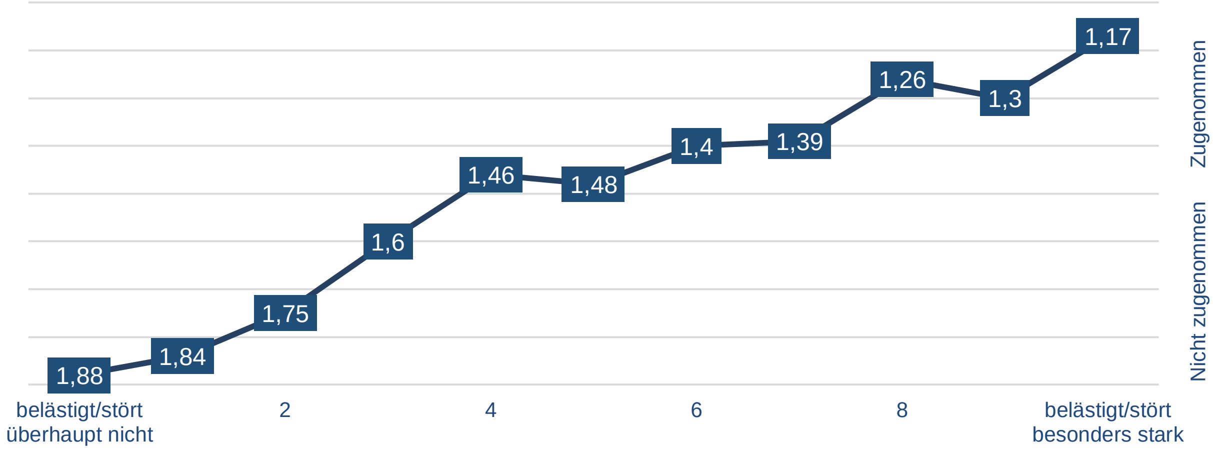

Die stark ansteigende Einschätzung der Zunahme der Lärmbelastungen in Innsbruck in den letzten Jahren ist bei steigender Belästigung durch Schienenverkehrslärm am geringsten, in allen anderen Belästigungskategorien nach Quelle relativ stark ausgeprägt. Rund 70% der Starkbelästigten gehen relativ unabhängig von der Quelle der Belästigung (Ausnahme Schienenverkehrslärmbelästigung) von Lärmzunahmen aus, während es bei den geringbelästigten Befragten im Mittel nur ein Drittel ist:

Tabelle 172: Empfundene Zu- und Abnahme der Lärmbelastung und Belästigung nach Quelle

		Gefühlte Zunahme Lärmbelastung				Gefühlte Abnahme Lärmbelastung			
		Ja		Nein		Ja		Nein	
		Anzahl	Prozent	Anzahl	Prozent	Anzahl	Prozent	Anzahl	Prozent
**Belästigung im Schlaf Lärm gesamt**	Gering belästigt	220	31,7%	475	68,3%	44	6,3%	651	93,7%
	Mittelgradig belästigt	160	62,5%	96	37,5%	13	5,1%	243	94,9%
	Stark belästigt	56	70,0%	24	30,0%	1	1,3%	79	98,8%
**Belästigung Lärm gesamt**	Gering belästigt	69	18,3%	309	81,7%	32	8,5%	346	91,5%
	Mittelgradig belästigt	272	51,5%	256	48,5%	24	4,5%	504	95,5%

	Stark belästigt	95	76,0%	30	24,0%	2	1,6%	123	98,4%
**Belästigung Fluglärm**	Gering belästigt	162	31,8%	348	68,2%	36	7,1%	474	92,9%
	Mittelgradig belästigt	191	47,8%	209	52,3%	20	5,0%	380	95,0%
	Stark belästigt	83	68,6%	38	31,4%	2	1,7%	119	98,3%
**Belästigung Strassenverkehrslärm**	Gering belästigt	155	28,8%	384	71,2%	37	6,9%	502	93,1%
	Mittelgradig belästigt	197	52,5%	178	47,5%	17	4,5%	358	95,5%
	Stark belästigt	84	71,8%	33	28,2%	4	3,4%	113	96,6%
**Belästigung Baustellenlärm**	Gering belästigt	212	31,6%	458	68,4%	40	6,0%	630	94,0%
	Mittelgradig belästigt	148	56,1%	116	43,9%	16	6,1%	248	93,9%
	Stark belästigt	76	79,2%	20	20,8%	2	2,1%	94	97,9%
**Belästigung Nachbarschaftslärm**	Gering belästigt	262	35,6%	474	64,4%	42	5,7%	694	94,3%
	Mittelgradig belästigt	138	56,8%	105	43,2%	14	5,8%	229	94,2%
	Stark belästigt	36	69,2%	16	30,8%	2	3,8%	50	96,2%
**Belästigung Lärm von Passanten und Gaststätten**	Gering belästigt	271	36,2%	477	63,8%	40	5,3%	708	94,7%
	Mittelgradig belästigt	133	57,1%	100	42,9%	15	6,4%	218	93,6%
	Stark belästigt	32	64,0%	18	36,0%	3	6,0%	47	94,0%
**Belästigung Schienenverkehrslärm**	Gering belästigt	338	40,2%	502	59,8%	45	5,4%	795	94,6%
	Mittelgradig belästigt	81	50,3%	80	49,7%	11	6,8%	150	93,2%
	Stark belästigt	17	56,7%	13	43,3%	2	6,7%	28	93,3%
**Belästigung Erschütterungen**	Gering belästigt	335	38,9%	527	61,1%	51	5,9%	811	94,1%
	Mittelgradig belästigt	74	54,8%	61	45,2%	7	5,2%	128	94,8%
	Stark belästigt	27	79,4%	7	20,6%	0	0,0%	34	100,0%
**Belästigung Lärm aus Gewerbe u. Industrie**	Gering belästigt	376	40,1%	561	59,9%	57	6,1%	880	93,9%
	Mittelgradig belästigt	48	60,8%	31	39,2%	1	1,3%	78	98,7%
	Stark belästigt	12	80,0%	3	20,0%	0	0,0%	15	100,0%
**Belästigung Autobahnlärm**	Gering belästigt	389	41,1%	558	58,9%	55	5,8%	892	94,2%
	Mittelgradig belästigt	32	51,6%	30	48,4%	2	3,2%	60	96,8%
	Stark belästigt	15	68,2%	7	31,8%	1	4,5%	21	95,5%

Anm.: Die Fallzahlen in der Gruppe von Befragten, die von einer Abnahme der Lärmbelastung in den letzten Jahren ausgehen, sind in einigen der obenstehenden Subgruppen zu gering für solide Auswertungen.

Einschätzung der Auswirkungen von Lärm auf die Gesundheit

1. Auswirkungen von Lärm im Überblick

Mehr als drei Viertel (75,8%) der befragten Innsbruckerinnen und Innsbrucker gehen davon aus, dass sich Lärm *eher stark* bis *sehr stark* negativ auf die Gesundheit auswirkt, 21% nehmen an, dass Lärmbelastungen nur geringe Gesundheitswirkungen aufweisen:

Abbildung 232: Einschätzung der negativen Wirkung von Lärm auf die Gesundheit in Prozent

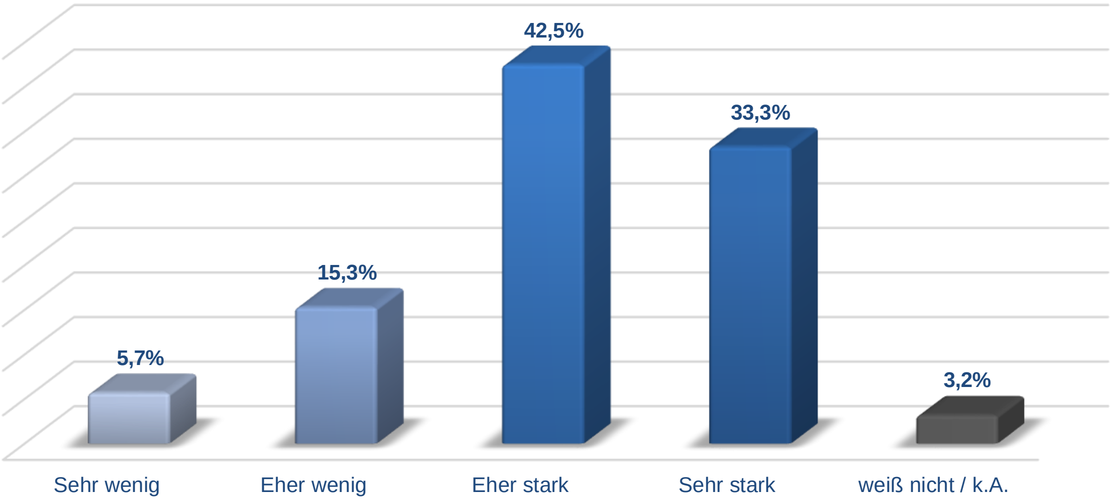

Tabelle 173: Einschätzung der negativen Wirkung von Lärm auf die Gesundheit gruppiert

		Häufigkeit	Gültige Prozente	Kumulierte Prozente
Gültig	Wenig	217	21,0	21,0
	Stark	781	75,8	96,8
	w.n. / k.A.	33	3,2	100,0
	Gesamt	1031	100,0	

Im Mittel gehen die Befragten von einer *eher starken* negativen Gesundheitswirkung von Lärm aus (Mittelwert 3,1 von maximal 4,0):

Tabelle 174: Mittelwert der Einschätzung der negativen Wirkung von Lärm auf die Gesundheit

Mittelwert	N	Standardabweichung	Standardfehler des Mittelwertes
3,07	998	,857	,027

* ohne Berücksichtigung der 33 Probandinnen und Probanden, die auf diese Frage mit weiß nicht / keine Angabe antworteten.

2. Auswirkungen von Lärm und ausgewählte Parameter

Männer gehen von einer deutlich geringeren negativen Wirkung von Lärm auf die Gesundheit aus als Frauen, während das Alter der Befragten kaum Einflüsse zeigt.

Stark unterschiedlich antworten Befragte in Abhängigkeit von ihrem höchsten bisher erreichten höchsten Schulabschluss: Von den Akademikerinnen und Akademikern gehen über 86% davon aus, dass Lärm starke negative Auswirkungen auf die Gesundheit hat, während bei den Pflichtschulabgängerinnen und –Abgängern etwas mehr als 61% dieser Meinung sind.

Abbildung 233: Einschätzung der negativen Wirkung von Lärm auf die Gesundheit und höchster erreichter Schulabschluss (gruppiert, ohne Antwortkategorie w.n. / k.A.)

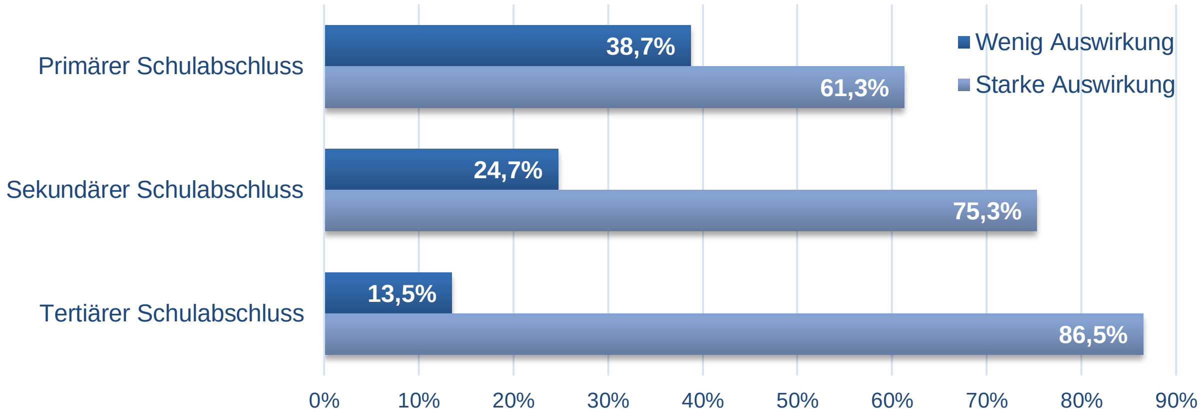

Die vorhandene Lärmbelastung zeigt nur beim Straßenverkehrslärm Beziehungen zur Einschätzung der negativen Wirkung von Lärm auf die Gesundheit auf. Diese sind vergleichsweise gering ausgeprägt.

Deutlich stärker fallen die Korrelationen zum Ausmaß der Lärmempfindlichkeit, dem Gefühl der Ausgesetztheit gegenüber Lärm, sowie den Belästigungsauswertungen aus:

Tabelle 175: Einschätzung der negativen Wirkung von Lärm auf die Gesundheit und ausgewählte Parameter (gruppiert)

		Einschätzung des Ausmaßes negativer Auswirkungen von Lärm auf die Gesundheit (gruppiert)			
		Wenig		Stark	
		Anzahl	Zeilen (%)	Anzahl	Zeilen (%)
**Geschlecht**	weiblich	94	17,9%	431	82,1%
	männlich	123	26,0%	350	74,0%
**Altersgruppen**	18 bis 40 Jahre	97	22,8%	328	77,2%
	41 bis 60 Jahre	60	19,5%	248	80,5%
	über 60 Jahre	60	22,6%	205	77,4%
**Bildungsschicht**	Primärer Schulabschluss	36	38,7%	57	61,3%
	Sekundärer Schulabschluss	129	24,7%	394	75,3%
	Tertiärer Schulabschluss	51	13,5%	328	86,5%
**Straßenlärm 24h**	Stark belastet >55dB	125	25,2%	372	74,8%

	Mittelgradig belastet 45-55dB	78	19,9%	313	80,1%
	Gering belastet <45dB	14	12,7%	96	87,3%
**Schienenlärm 24h**	Stark belastet >55dB	29	25,2%	86	74,8%
	Mittelgradig belastet 45-55dB	78	25,3%	230	74,7%
	Gering belastet <45dB	110	19,1%	465	80,9%
**Fluglärm 24h**	Stark belastet >55dB	25	23,6%	81	76,4%
	Mittelgradig belastet 45-55dB	64	18,2%	287	81,8%
	Gering belastet <45dB	128	23,7%	413	76,3%
**Notwendigkeit (weiterer) Maßnahmen zur Lärmreduktion**	Ja	102	15,1%	575	84,9%
	Nein	73	40,1%	109	59,9%
**Lärmempfindlichkeit**	Nicht lärmempfindlich	73	28,0%	188	72,0%
	Durchschnittlich	123	21,6%	447	78,4%
	Sehr lärmempfindlich	21	12,6%	146	87,4%
**Belästigung im Schlaf Lärm gesamt**	Gering belästigt	164	24,3%	511	75,7%
	Mittelgradig belästigt	46	18,9%	197	81,1%
	Stark belästigt	7	8,8%	73	91,3%
**Belästigung Lärm gesamt**	Gering belästigt	100	27,7%	261	72,3%
	Mittelgradig belästigt	102	19,9%	411	80,1%
	Stark belästigt	15	12,1%	109	87,9%

Befragte, welche davon ausgehen, dass die negativen Wirkungen von Lärm auf die Gesundheit stark ausgeprägt sind, sind auch wesentlich häufiger der Ansicht, dass (weitere) Maßnahmen zur Lärmreduktion in Innsbruck getroffen werden sollten und umgekehrt:

Abbildung 234: Einschätzung der negativen Wirkung von Lärm auf die Gesundheit und Notwendigkeit (weiterer Maßnahmen) zur Lärmreduktion (gruppiert, ohne Antwortkategorie w.n. / k.A.)

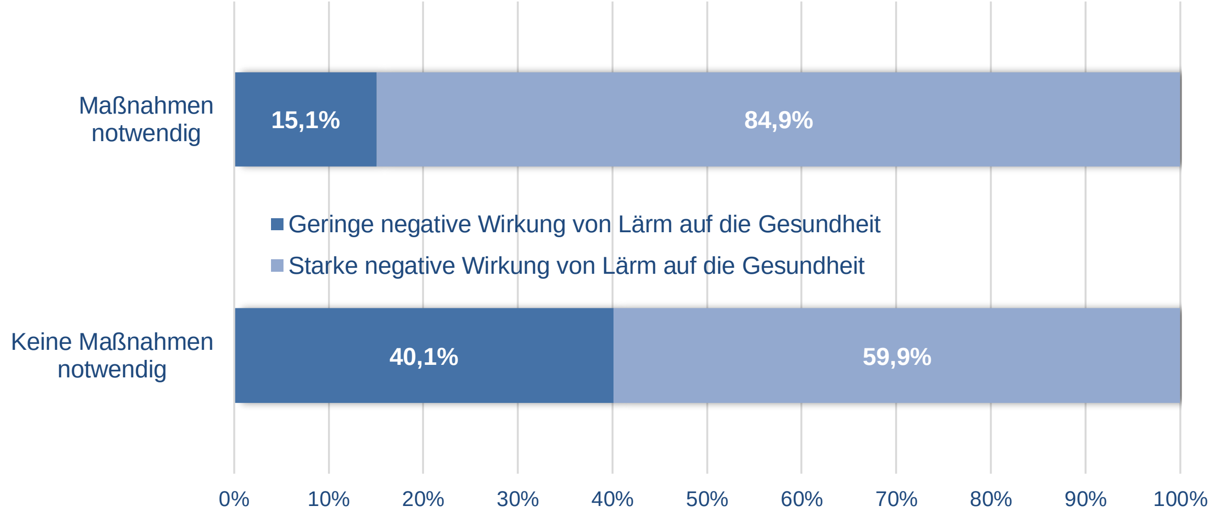

Bekanntheit der Umgebungslärmkarten

Mittels einer einfachen Ja/Nein-Frage wurde die Bekanntheit der Umgebungslärmkarten (http://www.laerminfo.at/) abgefragt. Etwas mehr als 21% aller Befragten haben schon einmal von Umgebungslärmkarten gehört, oder sich diese sogar angesehen. Mehr als drei Viertel der Probandinnen und Probanden sind diese unbekannt:

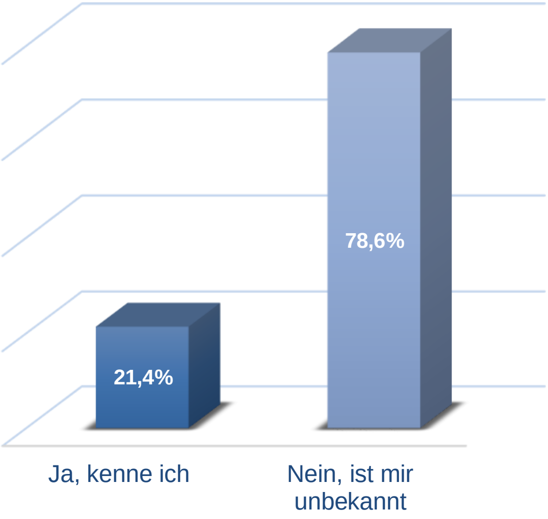

Abbildung 235: Bekanntheit der Umgebungslärmkarten in Prozent

- Die Bekanntheit der Umgebungslärmkarten nimmt mit zunehmendem Alter leicht ab.
- Männer geben doppelt so häufig wie Frauen an, dass ihnen Umgebungslärmkarten bekannt sind.
- Je höher der höchste bisher erreichte Schulabschluss, desto eher haben Befragte schon von Umgebungslärmkarten gehört oder sich diese angesehen.
- Es lassen sich keine eindeutigen Zusammenhänge zwischen der Bekanntheit der Umgebungslärmkarten und der vorhandenen Lärmbelastung, dem Gefühl der Ausgesetztheit gegenüber Lärm, der Lärmempfindlichkeit, oder der Belästigung durch Lärm nach Quelle herstellen.
- Befragte Innsbruckerinnen und Innsbruckern, die angeben, schon zumindest einmal mit Behörden über die Lärmbelastung gesprochen zu haben, kennen die Umgebungslärmkarten nicht häufiger, als jene, die das noch nie getan haben. Befragte, die (weitere) Maßnahmen zur Lärmreduktion in Innsbruck für sinnvoll halten, geben deutlich häufiger an, von Umgebungslärmkarten schon gehört oder sich diese angesehen zu haben.

Tabelle 176: Bekanntheit der Umgebungslärmkarten und ausgewählte Parameter (gruppiert)

		Bekanntheitsgrad Umgebungslärmkarten			
		Ja, kenne ich		Nein, ist mir unbekannt	
		Anzahl	Zeilen (%)	Anzahl	Zeilen (%)
**Geschlecht**	weiblich	80	14,8%	461	85,2%
	männlich	141	28,8%	349	71,2%
**Altersgruppen**	18 bis 40 Jahre	102	23,2%	338	76,8%
	41 bis 60 Jahre	69	21,8%	247	78,2%
	über 60 Jahre	50	18,2%	225	81,8%
**Bildungsschicht**	Primärer Schulabschluss	10	10,2%	88	89,8%
	Sekundärer Schulabschluss	99	18,3%	443	81,7%
	Tertiärer Schulabschluss	112	28,9%	276	71,1%
**Notwendigkeit (weiterer) Maßnahmen zur Lärmreduktion**	Ja	168	24,3%	523	75,7%
	Nein	26	13,5%	166	86,5%

Einschätzung des Lärmniveaus im Städtevergleich

1. Lärmniveau im Städtevergleich im Überblick

Bei der Frage, wie laut es in Innsbruck im Vergleich zu anderen Städten in Österreich ist, gehen die Meinungen der Befragten auseinander. Während etwas mehr als 28% der Meinung sind, es wäre in Innsbruck *ruhiger* bis *viel ruhiger*, gehen knapp 17% davon aus, dass es *lauter* bis *viel lauter* sei. Etwas mehr als die Hälfte der Befragten glaubt, dass das Lärmniveau in Innsbruck in etwa dem anderer österreichischer Städte entspricht:

Abbildung 236: Einschätzung des Lärmniveaus im Städtevergleich in Prozent

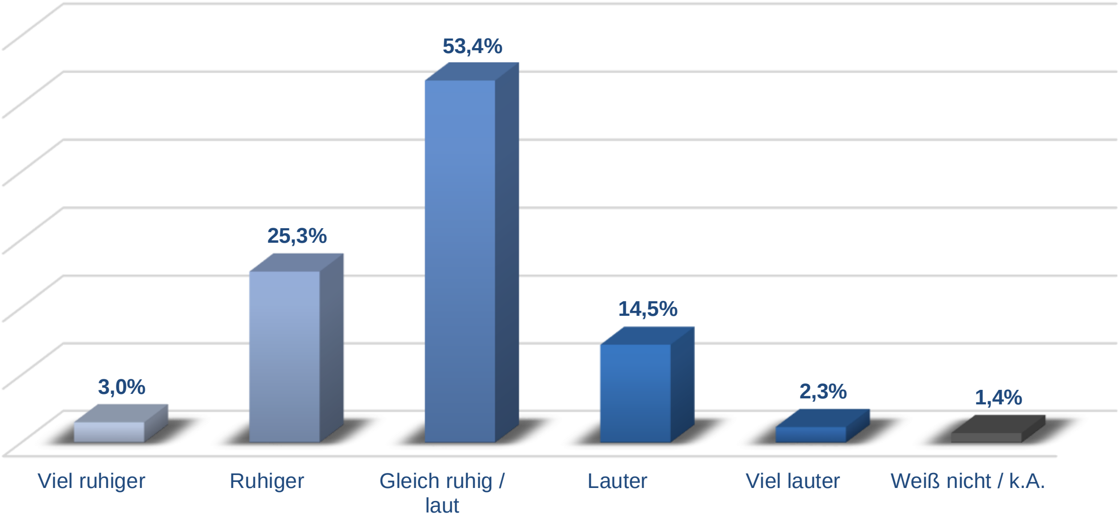

Tabelle 177: Einschätzung des Lärmniveaus im Städtevergleich gruppiert

		Häufigkeit	Gültige Prozente	Kumulierte Prozente
Gültig	Ruhiger	292	28,3	28,3
	Gleich	551	53,4	81,8
	Lauter	174	16,9	98,6
	Keine Angabe	14	1,4	100,0
	Gesamt	1031	100,0	

Im Mittel gehen die Befragten davon aus, dass es Innsbruck in etwa *gleich ruhig oder laut* im Vergleich zu anderen österreichischen Städten ist, mit leichter Tendenz, dass es etwas ruhiger sei:

Tabelle 178: Mittelwert der Einschätzung des Lärmniveaus im Städtevergleich

Mittelwert	N	Standardabweichung	Standardfehler des Mittelwertes
2,88	1017	,778	,024

* ohne Berücksichtigung der 24 Probandinnen und Probanden, die auf diese Frage mit weiß nicht / keine Angabe antworteten.

2. Lärmniveau im Städtevergleich und einzelne Parameter

Vor allem jüngere Befragte und Männer streuen in ihrer Meinung stärker als Frauen und ältere Befragte und halten Innsbruck häufiger einerseits für weniger, andererseits für stärker lärmbelastet als andere Städte ÖsterreichS. Ältere Befragte und Frauen sind hingegen mit größerer Mehrheit der Meinung, es sei in Innsbruck gleich laut.

Je höher der höchste bisher erreichte Schulabschluss, desto eher glauben Befragte es sei in Innsbruck lauter als in vergleichbaren Städten.

Die Bezüge zwischen der Einschätzung, wie laut es in Innsbruck im Vergleich zu anderen Städten in Österreich ist und der vorhandenen Lärmbelastung nach Quelle in der Wohnumgebung der Befragten sind nicht eindeutig. Stärker Lärmbelastete sind nicht per se der Meinung, es sei in Innsbruck lauter.

Befragte, die in ihrem Wohnumfeld über eine lärmabgewandte Seite, einen Innenhof oder baulichen Schutz vor Verkehrslärm verfügen, schätzen Innsbruck eher als ruhiger ein, als Probandinnen und Probanden, deren Wohnung stärker lärmexponiert ist.

Weniger lärmempfindliche Befragte schätzen Innsbruck im Städtevergleich eher als ruhiger ein, als sich selbst als stärker lärmempfindlich wahrnehmende Personen:

Abbildung 237: Einschätzung des Lärmniveaus und Lärmempfindlichkeit im Mittelwertvergleich

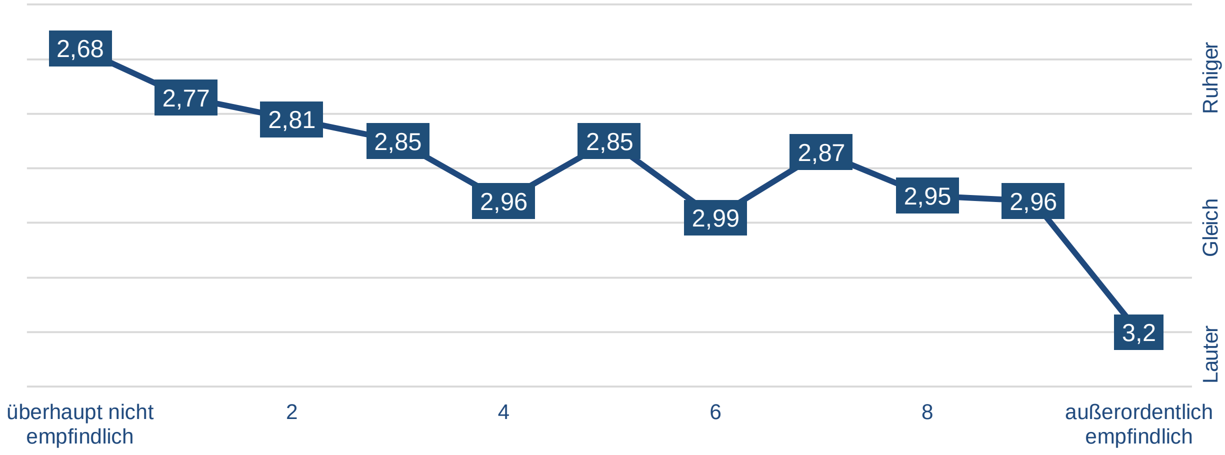

Vergleichbares gilt für diejenigen, die ihre persönliche Lebensqualität und ihre Wohn- und Lebensbedingungen höher einstufen und sich weniger lärmexponiert empfinden.

Befragte, die (weitere) Maßnahmen zur Lärmreduktion in Innsbruck für sinnvoll erachten, halten Innsbruck deutlich häufiger für lauter als andere vergleichbare Städte Österreichs.

Aus den Auswertungen in Zusammenschau mit den Belästigungsreaktionen ist ersichtlich, dass die Frage nach dem Lärmniveau von Innsbruck im Städtevergleich von den Befragten stark auf den Verkehrslärm und dabei vor allem auf den Straßenverkehrslärm fokussiert wird. Insgesamt sind die Unterschiede zwischen den sich stark gestört / belästigt fühlenden Befragten und den eher geringfügig Belästigten deutlich ausgeprägt:

Abbildung 238: Einschätzung des Lärmniveaus und Belästigung durch Lärm gesamt und Lärm gesamt im Schlaf (gruppiert)

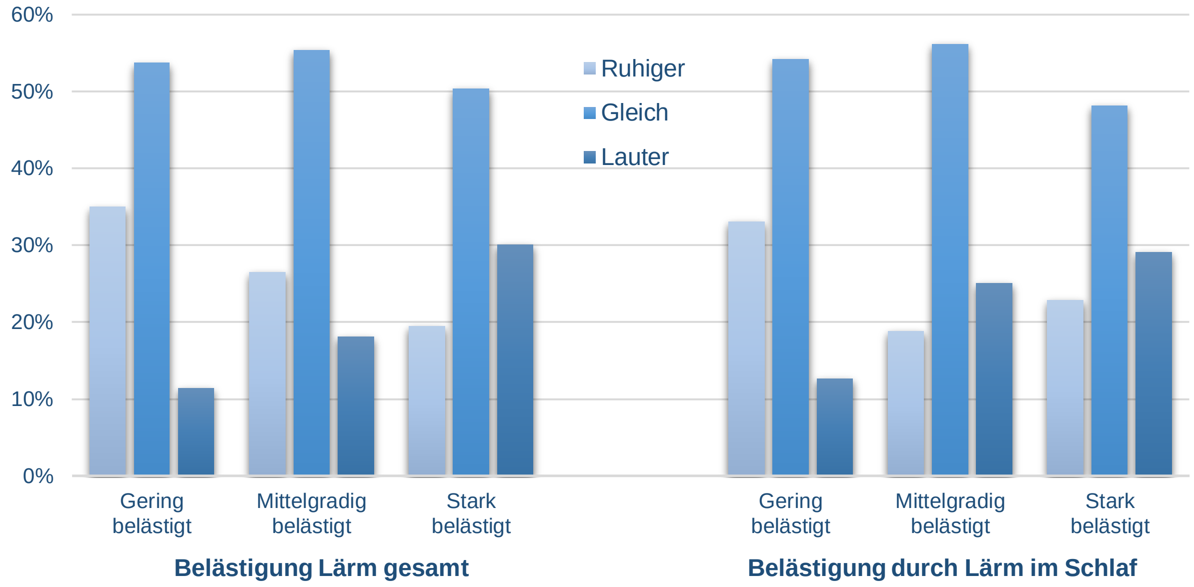

Tabelle 179: Einschätzung des Lärmniveaus im Städtevergleich und ausgewählte Parameter

		Lautheit Gesamtlärm in Innsbruck im Vergleich zu anderen Städten Österreichs					
		Ruhiger		Gleich		Lauter	
		Anzahl	Prozent	Anzahl	Prozent	Anzahl	Prozent
**Geschlecht**	weiblich	144	27,0%	309	57,9%	81	15,2%
	männlich	148	30,6%	242	50,1%	93	19,3%
**Altersgruppen**	18 bis 40 Jahre	149	34,4%	204	47,1%	80	18,5%
	41 bis 60 Jahre	84	26,7%	176	55,9%	55	17,5%
	über 60 Jahre	59	21,9%	171	63,6%	39	14,5%
**Bildungsschicht**	Primärer Schulabschluss	33	34,0%	50	51,5%	14	14,4%
	Sekundärer Schulabschluss	160	29,9%	290	54,2%	85	15,9%
	Tertiärer Schulabschluss	98	25,7%	209	54,7%	75	19,6%
**Baulicher Schutz vor Verkehrslärm**	Nicht bis kaum vorhanden	202	26,7%	417	55,2%	137	18,1%
	Vorhanden	80	34,5%	120	51,7%	32	13,8%
**Innenhof bzw. lärmabgewandte Seite**	Ja	214	31,0%	362	52,4%	115	16,6%
	Nein	72	23,7%	178	58,6%	54	17,8%
**Straßenlärm 24h**	Stark belastet >55dB	138	26,9%	269	52,4%	106	20,7%
	Mittelgradig belastet 45-55dB	130	32,7%	212	53,4%	55	13,9%
	Gering belastet <45dB	24	22,4%	70	65,4%	13	12,1%
**Schienenlärm 24h**	Stark belastet >55dB	29	25,2%	70	60,9%	16	13,9%
	Mittelgradig belastet 45-55dB	92	29,0%	159	50,2%	66	20,8%
	Gering belastet <45dB	171	29,2%	322	55,0%	92	15,7%
**Fluglärm 24h**	Stark belastet >55dB	33	29,5%	66	58,9%	13	11,6%
	Mittelgradig belastet 45-55dB	108	30,3%	172	48,3%	76	21,3%

	Gering belastet <45dB	151	27,5%	313	57,0%	85	15,5%
**Lebensbedingungen (Summenscore)**	Nicht gut	0(*)	0,0%	3(*)	60,0%	2(*)	40,0%
	Durchschnittlich	169	25,6%	354	53,6%	137	20,8%
	Sehr gut	123	34,9%	194	55,1%	35	9,9%
**Lebensqualität**	Nicht gut	2(*)	20,0%	6	60,0%	2(*)	20,0%
	Durchschnittlich	73	25,6%	139	48,8%	73	25,6%
	Sehr gut	217	30,3%	401	55,9%	99	13,8%
**Notwendigkeit (weiterer) Maßnahmen Lärmreduktion**	Ja	172	25,2%	367	53,7%	144	21,1%
	Nein	72	38,3%	94	50,0%	22	11,7%
**Lärmempfindlichkeit**	Nicht lärmempfindlich	100	36,6%	135	49,5%	38	13,9%
	Durchschnittlich	157	27,4%	315	54,9%	102	17,8%
	Sehr lärmempfindlich	35	20,6%	101	59,4%	34	20,0%
**Ausgesetztheit Lärm gesamt**	Gering exponiert	85	33,5%	139	54,7%	30	11,8%
	Mittelgradig exponiert	180	28,8%	342	54,8%	102	16,3%
	Stark exponiert	27	19,4%	70	50,4%	42	30,2%
**Belästigung im Schlaf Lärm gesamt**	Gering belästigt	226	33,1%	370	54,2%	87	12,7%
	Mittelgradig belästigt	48	18,8%	143	56,1%	64	25,1%
	Stark belästigt	18	22,8%	38	48,1%	23	29,1%
**Belästigung Lärm gesamt**	Gering belästigt	129	35,0%	198	53,7%	42	11,4%
	Mittelgradig belästigt	139	26,5%	291	55,4%	95	18,1%
	Stark belästigt	24	19,5%	62	50,4%	37	30,1%
**Belästigung Straßenverkehrslärm**	Gering belästigt	187	35,3%	280	52,8%	63	11,9%
	Mittelgradig belästigt	83	22,4%	208	56,2%	79	21,4%
	Stark belästigt	22	18,8%	63	53,8%	32	27,4%

(*) zu kleine Fallzahlen für solide Auswertungen

Ohne Antwortkategorie w.n. / k.A.

Einschätzung des eigenen Lärmbeitrags

1. Eigener Lärmbeitrag im Überblick

Der eigene Beitrag zur Gesamtlärmbelastung in Innsbruck wird von den Befragten größtenteils als *sehr gering* bis *gering* eingeschätzt (75,2%). Nur 2,8% sind der Meinung, Sie selber tragen *stark* oder *sehr stark* zur Lärmbelastung bei. 22% gehen davon aus, ihr eigener Lärmbeitrag sei *durchschnittlich*:

Abbildung 239: Einschätzung des eigenen Lärmbeitrags in Prozent

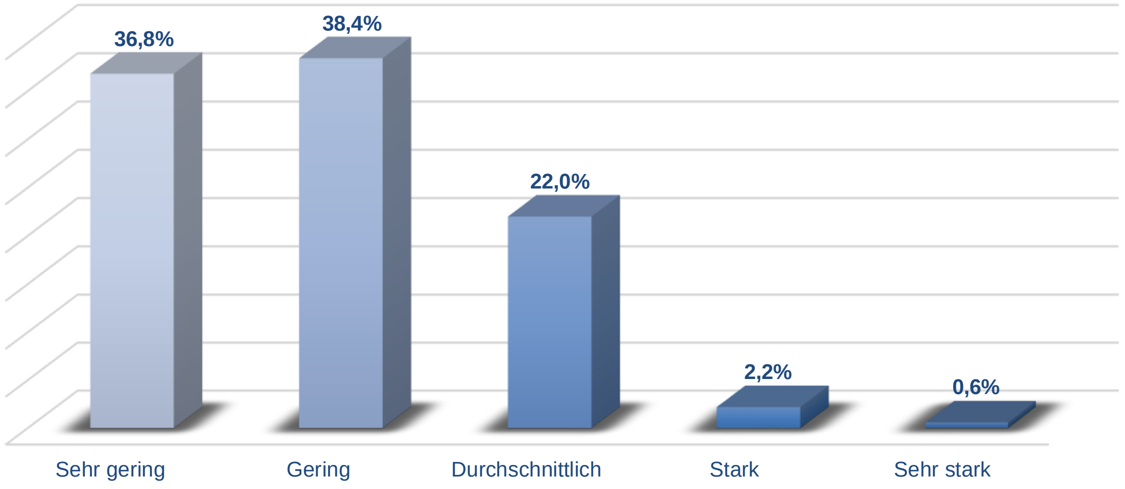

Tabelle 180: Einschätzung des eigenen Lärmbeitrags gruppiert

		Häufigkeit	Gültige Prozente	Kumulierte Prozente
Gültig	Gering	775	75,2	75,2
	Durchschnittlich	227	22,0	97,2
	Stark	29	2,8	100,0
	Gesamt	1031	100,0	

Im Mittel gehen die befragten Innsbruckerinnen und Innsbrucker davon aus, ihr eigener Beitrag zum Lärm in Innsbruck sei *gering*:

Tabelle 181: Mittelwert Einschätzung des eigenen Lärmbeitrags

Einschätzung eigener Beitrag zur Gesamtlärmbelastung in Innsbruck

Mittelwert	N	Standardabweichung	Standardfehler des Mittelwertes
1,2764	1031	,50648	,01577

2. Eigener Lärmbeitrag und demographische Parameter

Männer gehen von einem etwas größeren eigenen Lärmbeitrag zur Gesamtlärmsituation in Innsbruck aus als Frauen.

Befragte über 60 Jahren glauben zu über 82%, dass sie selbst nur wenig zur Gesamtlärmbelastung in Innsbruck beitragen.

Befragte, welche in Haushalten ohne minderjährige Kinder leben, halten ihren eigenen Lärmbeitrag für geringer als Befragte in Haushalten mit minderjährigen Kindern.

Je höher der bisher erreichte höchste Bildungsabschluss, desto geringer schätzen Befragte ihren eigenen Lärmbeitrag ein:

Tabelle 182: Einschätzung des eigenen Lärmbeitrags und demographische Parameter (gruppiert)

		Einschätzung eigener Beitrag zur Gesamtlärmbelastung					
		Gering		Durchschnittlich		Groß	
		Anzahl	Prozent	Anzahl	Prozent	Anzahl	Prozent
**Geschlecht**	weiblich	421	77,8%	109	20,1%	11	2,0%
	männlich	354	72,2%	118	24,1%	18	3,7%
**Altersgruppen**	18 bis 40 Jahre	323	73,4%	104	23,6%	13	3,0%
	41 bis 60 Jahre	225	71,2%	80	25,3%	11	3,5%
	über 60 Jahre	227	82,5%	43	15,6%	5	1,8%
**Haushalt mit/ohne minderjährige Kinder**	Haushalt ohne Kinder	586	76,9%	154	20,2%	22	2,9%
	Haushalt mit Kindern	189	70,3%	73	27,1%	7	2,6%
**Bildungsschicht**	Primärer Schulabschluss	69	70,4%	27	27,6%	2	2,0%
	Sekundärer Schulabschluss	405	74,7%	118	21,8%	19	3,5%
	Tertiärer Schulabschluss	298	76,8%	82	21,1%	8	2,1%

3. Eigener Lärmbeitrag und Mobilitätsverhalten

Pendlerinnen und Pendler schätzen ihren eigenen Lärmbeitrag zur Gesamtlärmbelastung in Innsbruck deutlich höher ein als Befragte, welche nicht zu Arbeits- oder Ausbildungszwecken pendeln.

Unter den Pendlerinnen und Pendlern glauben diejenigen, welche ein Kraftfahrzeug als vorrangiges Verkehrsmittel auf dem Weg zum Ausbildungs- oder Arbeitsplatz benutzen, einen deutlich größeren Beitrag zur Gesamtlärmbelastung in Innsbruck beizusteuern, als alle anderen. Zwischen den Benutzerinnen und Benutzern von Eisenbahn, Linienbussen und öffentlichen Verkehrsmitteln, Fußgängern und Fahrradfahrerinnen und –Fahrern sind die Unterschiede hingegen gering. Den geringsten Lärmbeitrag erkennen (ohne Adjustierung anderer Prädiktoren und Kovariablen) diejenigen für sich, die mit dem Fahrrad unterwegs sind:

Abbildung 240: Einschätzung des eigenen Lärmbeitrags und vorrangiges Verkehrsmittel im Mittelwertvergleich

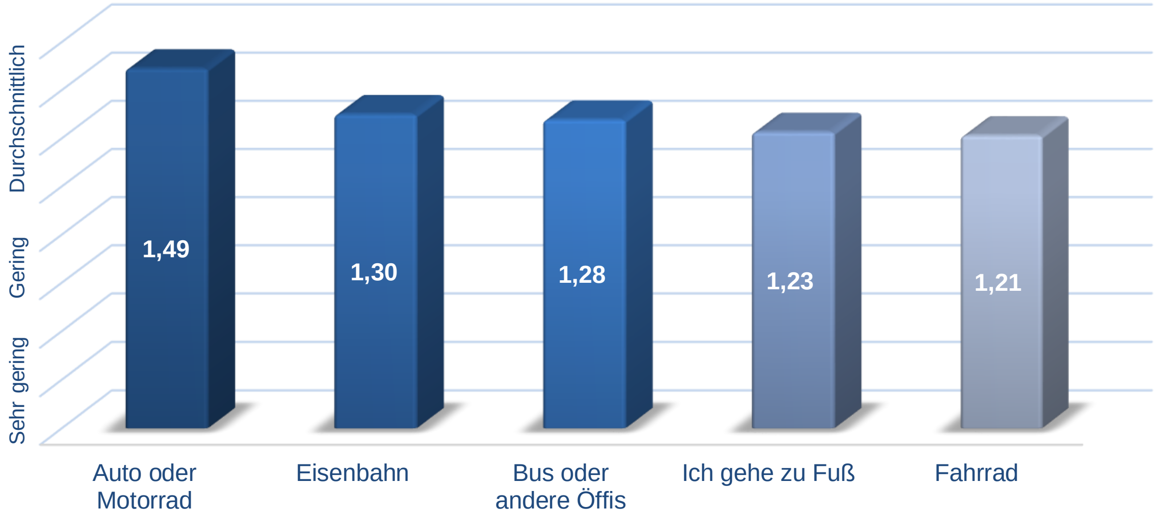

Ohne Nichtzutreffende (in Pension ...)

Wird das Mobilitätsverhalten der Befragten nach der Häufigkeit der Nutzung einzelner Verkehrsmittel unabhängig vom berufs- oder ausbildungsabhängigen Pendeln abgefragt und aufgeschlüsselt, können die Unterschiede in der Empfindung des eigenen Lärmbeitrages zur Gesamtlärmsituation in Innsbruck verfeinert werden: Diejenigen befragten Innsbruckerinnen und Innsbrucker, die sich regelmäßig mit öffentlichen Verkehrsmitteln bewegen, glauben (ohne Adjustierung anderer Prädiktoren und Kovariablen) den geringsten Beitrag zum Gesamtlärm beizusteuern, (fast) tägliche Autofahrerinnen und Autofahrer den größten. Die Unterschiede zwischen diesen beiden Extremgruppen fallen sehr deutlich aus.

Zwischen regelmäßigen Fußgängerinnen und Fußgängern und den (fast) täglichen Nutzerinnen und Nutzern von Fahrädern bestehen kaum Unterschiede. Auch die gelegentlichen Nutzerinnen und Nutzer von Flugzeugen (Antwortkategorie *Mehrmals pro Jahr*[16]) erkennen für sich keinen per se größeren Lärmbeitrag:

Abbildung 241: Eigener Lärmbeitrag durchschnittlich bis groß und Mobilitätsverhalten im Extremgruppenvergleich inProzent

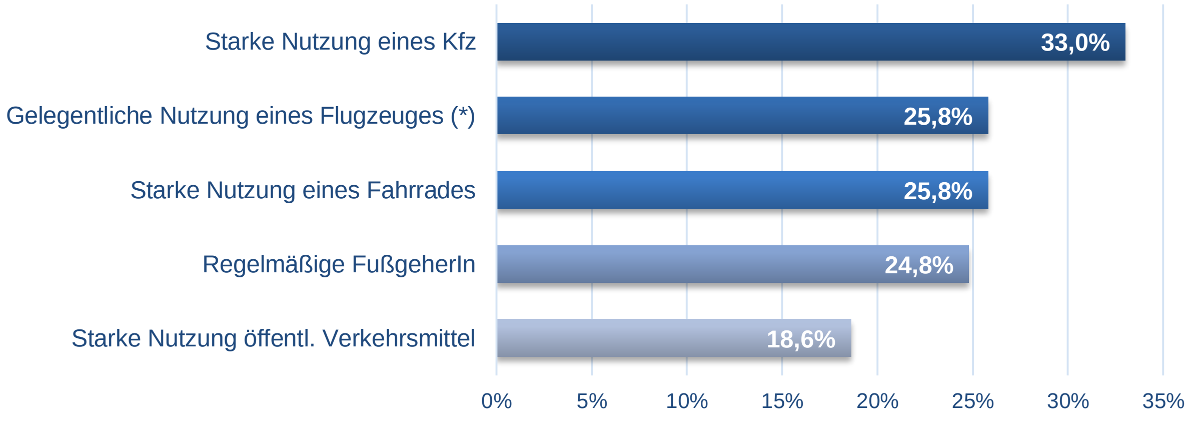

(*) Aufgrund der geringen Fallzahlen von starken Nutzerinnen bzw. Nutzern von Flugzeugen wurde für diesen Vergleich die Gruppierung *Gelegentliche Nutzung* (entspricht der Antwortkategorie „Mehrmals pro Jahr“) gewählt.

[16] Anm.: Umfasst auch einen einmal im Jahr stattfindenden Urlaub, der mit dem Flugzeug als Transportmittel erreicht wird und bei dem im Regelfall zumindest zwei Flüge – ein Hin- und ein Rückflug – absolviert werden.

Einschätzung der störendsten Lärmquelle in Innsbruck

1. Störendste Lärmquelle im Überblick

Fast 40% im befragten Kollektiv nehmen an, dass der Straßenverkehrslärm die Innsbruckerinnen und Innsbrucker insgesamt am meisten belästigt oder stört. Mit über 15 Prozentpunkten Abstand folgt der Flugverkehrslärm an zweiter Stelle, dicht gefolgt vom Baustellenlärm. Über 6% der Befragten machen keine Angaben zu dieser Frage:

Abbildung 242: Einschätzung der störendsten Lärmquelle in Innsbruck in Prozent

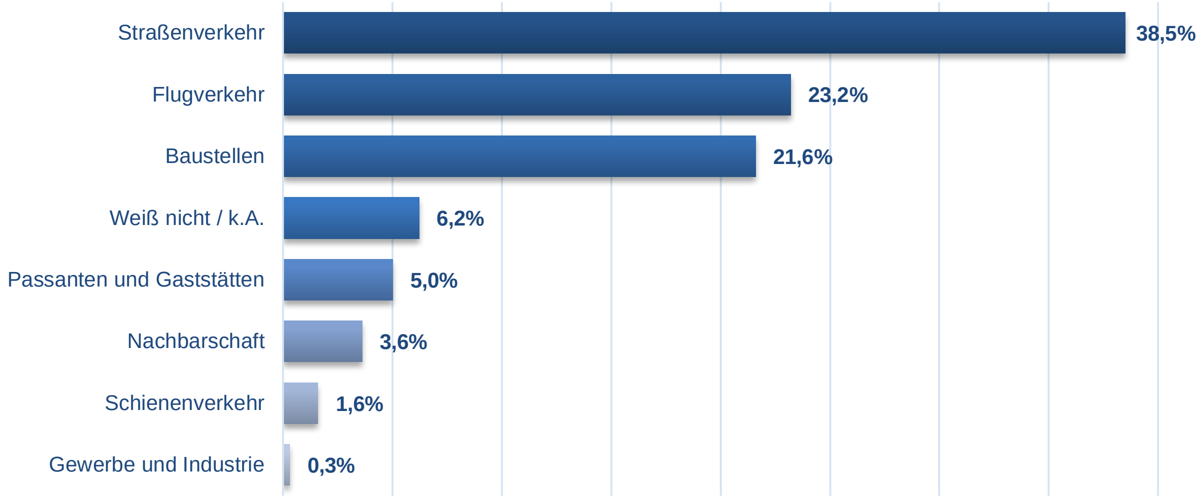

Die Lärmwirkungen ausgehend von Passanten und Gaststätten, Nachbarschaft, Schienenverkehr und Gewerbe und Industrie folgen mit sehr großem Abstand und werden nur von vergleichsweise wenigen bis sehr wenigen Befragten als störendste Lärmquelle in Innsbruck angenommen.

2. Störendste Lärmquelle und demographische Parameter

Männer, die Altersgruppe 60+ und Akademikerinnen und Akademiker sehen den Straßenverkehrslärm deutlicher als störendste Lärmquelle an als andere Subgruppen. Werden diese Attribute kombiniert, verstärkt sich dieser Effekt:

Tabelle 183: Einschätzung der störendsten Lärmquelle in Innsbruck und demographische Parameter

		Einschätzung störendste Lärmquelle in Innsbruck (*)						
		Straßen-verkehr	Schienen-verkehr	Flug-verkehr	Gewerbe und In-dustrie	Passanten und Gast-stätten	Bau-stellen	Nach-barschaft
		Prozent	Prozent	Prozent	Prozent	Prozent	Prozent	Prozent
Geschlecht	weiblich	40,2%	1,2%	23,3%	0,2%	5,9%	26,0%	3,2%
	männlich	42,0%	2,2%	26,3%	0,4%	4,8%	19,8%	4,6%
Altersgruppen	18 bis 40 Jahre	36,1%	0,2%	26,8%	0,2%	5,4%	27,3%	4,0%
	41 bis 60 Jahre	43,7%	3,1%	23,1%	0,0%	6,1%	19,3%	4,7%

	über 60 Jahre	46,5%	2,5%	23,0%	0,8%	4,5%	20,2%	2,5%
**Bildungsschicht**	Primär	37,9%	0,0%	19,5%	0,0%	6,9%	32,2%	3,4%
	Sekundär	40,1%	1,6%	21,1%	0,4%	6,1%	27,0%	3,7%
	Tertiär	43,4%	2,2%	30,6%	0,3%	4,1%	15,3%	4,1%

(*) ohne Antwortkategorie w.n. / k.A.

Vor allem bei Angehörigen der tertiären Bildungsschicht und Männern steht der Flugverkehrslärm klar an der zweiten Stelle der störendsten Lärmquellen, während Frauen, die jüngste Altersgruppe der 18 bis 40-Jährigen und Befragte mit Pflichtschulabschluss den Lärm ausgehend von Baustellen am zweithäufigsten und damit noch vor dem Flugverkehrslärm angeben:

Abbildung 243: Einschätzung der störendsten Lärmquelle und Bildungsschicht (gruppiert)

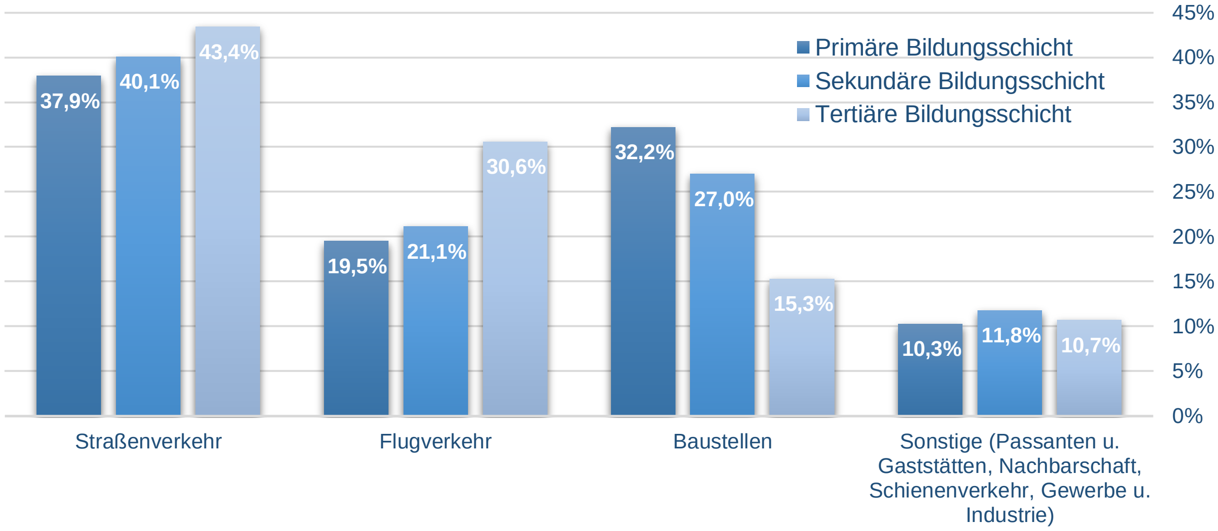

3. Störendste Lärmquelle und ausgewählte Parameter

Auch in der Zusammenschau mit diversen Subgruppen und Variablen wird der Straßenverkehr von den befragten Innsbruckerinnen und Innsbruckern durchgehend am häufigsten als störendste Lärmquelle genannt (eine Ausnahme bilden lediglich die Beziehungen zu den Belästigungsauswertungen – siehe dazu das Unterkapitel *5: Störendste Lärmquelle und Lärmbelästigung* und die Gruppe der vom Flugverkehrslärm stark Belasteten – siehe das Unterkapitel 4: *Störendste Lärmquelle und Lärmbelastung*). Allerdings werden in bi- und multivariaten Analysen Fokussierungseffekte sichtbar:

Befragte, deren Wohnräume durch einen Innenhof oder eine lärmabgewandte Seite zum Teil verkehrslärmgeschützt sind, streuen in ihren Antworten auf die Frage nach der störendsten Lärmquelle in Innsbruck stärker und fokussieren etwas weniger auf Straßen- und Flugverkehrslärm.

Befragte, die gewöhnlich bei geöffnetem oder gekipptem Fenster schlafen, sehen häufiger Baustellen als störendste Lärmquelle an, als Personen, die bei geschlossenem Fenster schlafen.

Probandinnen und Probanden, die davon ausgehen, dass es in Innsbruck gleich laut / ruhig wie in anderen vergleichbaren Städten Österreichs ist, sehen mit über 45% im Straßenverkehr die störendste Lärmquelle, während Befragte, die Innsbruck für lauter oder ruhiger halten, den Flugverkehr häufi-

ger als störendste Lärmquelle ansehen, welcher bei diesen Gruppen rund 5 Prozentpunkte hinter dem Straßenverkehr zu liegen kommt.

Ähnliches gilt für die Gruppe derjenigen die (weitere) Maßnahmen zur Lärmreduktion in Innsbruck für notwendig halten: Diese Befragten fokussieren stärker auf den Straßenverkehrslärm als auf Flugverkehrs- und Baulärm:

Tabelle 184: Einschätzung der störendsten Lärmquelle in Innsbruck und ausgewählte Parameter

		Einschätzung störendste Lärmquelle in Innsbruck (*)						
		**Straßen-verkehr**	**Schienen-verkehr**	**Flug-verkehr**	**Gewerbe und In-dustrie**	**Passanten und Gast-stätten**	**Bau-stellen**	**Nach-barschaft**
		Prozent	Prozent	Prozent	Prozent	Prozent	Prozent	Prozent
**Innenhof / lärm-abgew. Seite**	Ja	40,5%	1,4%	22,5%	0,3%	6,6%	24,3%	4,4%
	Nein	42,9%	2,1%	30,5%	0,4%	2,8%	18,8%	2,5%
**Nachtschlaf**	Fenster geöffnet	40,6%	1,5%	24,8%	0,1%	5,2%	24,4%	3,4%
	Fenster geschlossen	42,5%	2,2%	23,7%	1,1%	6,5%	18,3%	5,9%
**Lautheit Innsbruck im Vergleich**	Ruhiger	34,7%	1,1%	29,2%	0,4%	4,0%	26,7%	4,0%
	Gleich	45,8%	2,1%	20,5%	0,4%	6,2%	21,8%	3,1%
	Lauter	35,5%	1,2%	30,1%	0,0%	5,4%	21,7%	6,0%
**Maßnahmen zur Lärmreduktion**	Ja	42,5%	1,5%	24,4%	0,4%	4,9%	22,7%	3,6%
	Nein	32,4%	2,4%	29,4%	0,0%	7,1%	25,9%	2,9%
**Lärmempfind-lichkeit**	Nicht lärmempfindlich	39,0%	1,2%	22,5%	0,4%	6,0%	25,7%	5,2%
	Durchschnittlich	42,2%	1,1%	25,0%	0,0%	4,5%	23,5%	3,8%
	Sehr lärmempfindlich	40,4%	4,3%	27,3%	1,2%	7,5%	17,4%	1,9%
**Ausgesetztheit Lärm gesamt**	Gering exponiert	43,8%	1,3%	24,0%	0,4%	3,4%	25,3%	1,7%
	Mittelgradig exponiert	40,2%	1,7%	25,4%	0,2%	6,5%	22,0%	4,0%
	Stark exponiert	40,0%	2,2%	23,0%	0,7%	3,7%	23,7%	6,7%

(*) ohne Antwortkategorie w.n. / k.A.

Die Variable Ausgesetztheit gegenüber Verkehrslärm insgesamt zeigt keine deutlichen Bezüge. Werden jedoch die einzelnen Lärmquellen der empfundenen Exponiertheit korreliert, korrespondieren die Antworten der Befragten stark mit den für sie persönlich dominantesten Quellen (siehe dazu das Unterkapitel *5. Störendste Lärmquelle und Lärmbelästigung*).

4. Störendste Lärmquelle und Lärmbelastung

Von den einzelnen Lärmquellen geringbelastete Befragte glauben durchgehend am häufigsten, dass der Straßenverkehr die störendste Lärmquelle in Innsbruck ist. Stärker lärmbelastete Personen nähern sich den Mittelwerten an – mit einer Ausnahme: Stark fluglärmbelastete Innsbruckerinnen und Innsbrucker antworten entgegen dem Trend und sehen mit großem Abstand den Fluglärm als störendste Lärmquelle für Innsbruck insgesamt an:

Abbildung 244: Einschätzung der störendsten Lärmquelle und Lärmbelastung nach Quelle im Extremgruppenvergleich der Starkbelasteten

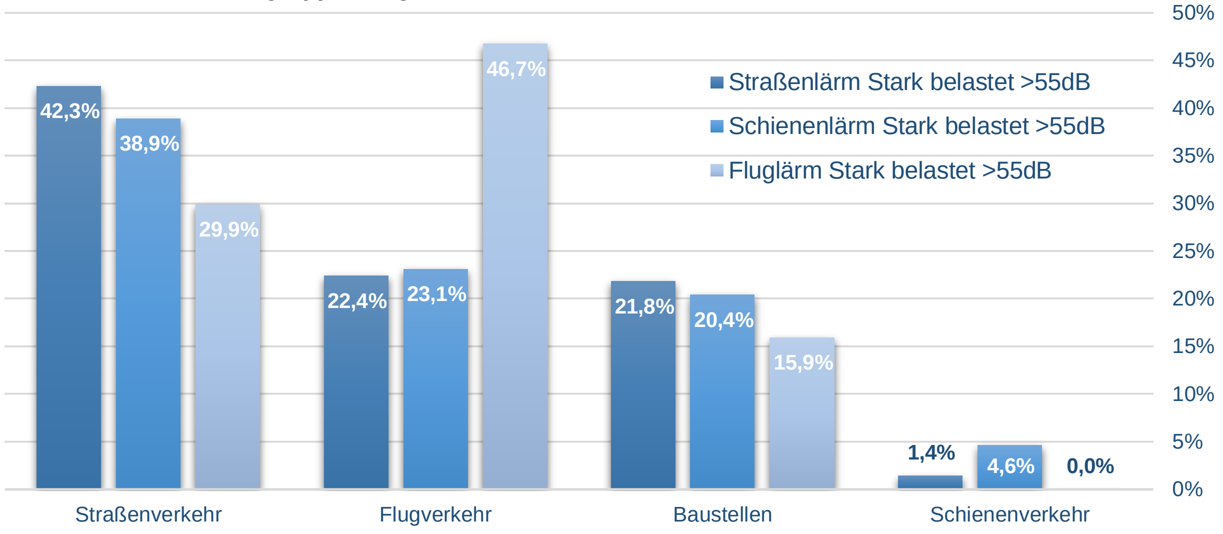

Eine weitere Auffälligkeit zeigt sich in diesem Extremgruppenvergleich der vom gegebenen Verkehrslärm Starkbelasteten: Während die stark Fluglärmbelasteten von der eigenen Situation in ihrem Wohnumfeld auf die Gesamtheit Innsbrucks schließen, tun dies die stark vom Schienenverkehrslärm Belasteten nicht. Zwar nehmen die stark Schienenverkehrslärmbelasteten zu einem größeren Teil als die Gesamtstichprobe an, dass der Schienenverkehr die störendste Lärmquelle sei (4,6%), der weitaus überwiegende Teil der stark Schienenverkehrslärmbelasteten liegt mit seinem Antwortverhalten jedoch ziemlich exakt im Mittel der Antworten der insgesamt Befragten. In Zusammenschau mit den Auswertungen zu den Belästigungsreaktionen (siehe das Kapitel *Belästigung / Störung durch Lärm*) lässt sich die Aussage erhärten, dass ein Großteil der befragten Innsbruckerinnen und Innsbrucker, darunter auch die vom Schienenverkehrslärm stark belasteten Befragten, den Schienenverkehrslärm im Vergleich zum Straßen- und Flugverkehrslärm, sowie dem Lärm ausgehend von Baustellen als weniger störend / belästigend einstuft.

Tabelle 185: Einschätzung der störendsten Lärmquelle in Innsbruck und Lärmbelastung nach Quelle

		Einschätzung störendste Lärmquelle in Innsbruck (*)						
		Straßenverkehr	Schienenverkehr	Flugverkehr	Gewerbe und Industrie	Passanten und Gaststätten	Baustellen	Nachbarschaft
		Prozent	Prozent	Prozent	Prozent	Prozent	Prozent	Prozent
**Straßenlärm 24h**	Stark belastet >55dB	42,3%	1,4%	22,4%	0,4%	7,4%	21,8%	4,3%
	Mittelgradig 45-55dB	36,2%	2,1%	28,5%	0,3%	4,0%	25,3%	3,7%
	Geringbelastet <45dB	52,9%	1,0%	22,1%	0,0%	1,0%	21,2%	1,9%
**Schienenlärm 24h**	Stark belastet >55dB	38,9%	4,6%	23,1%	0,9%	6,5%	20,4%	5,6%
	Mittelgradig 45-55dB	39,9%	1,0%	25,5%	0,3%	6,0%	24,2%	3,0%
	Geringbelastet <45dB	42,1%	1,4%	24,6%	0,2%	4,8%	23,0%	3,9%
**Fluglärm 24h**	Stark belastet >55dB	29,9%	0,0%	46,7%	0,9%	0,9%	15,9%	5,6%
	Mittelgradig 45-55dB	37,5%	1,7%	26,9%	0,3%	8,6%	22,1%	2,9%
	Geringbelastet <45dB	45,8%	2,0%	18,6%	0,2%	4,1%	25,2%	4,1%

(*) ohne Antwortkategorie w.n. / k.A.

5. Störendste Lärmquelle und Lärmbelästigung

Aus den Auswertungen ist ersichtlich, dass von einer bestimmten Lärmquelle stark belästigte Befragte dazu neigen, dieses Gefühl der persönlichen Störung auf die Gesamtheit Innsbrucks zu übertragen. Eine Abstraktion von der persönlichen Belästigung auf die insgesamt störendste Lärmquelle gelingt nicht (gänzlich), oder wird bewusst nicht angestrebt.

Die Extremgruppen der Starkbelästigten schätzen die sie selbst stark belästigende Quelle überproportional häufig als störendste Lärmquelle für Gesamtinnsbruck ein. Das gilt nicht nur für die Verkehrslärmquellen, sondern besonders ausgeprägt auch für die nicht-verkehrsinduzierten Lärmquellen:

Abbildung 245: Einschätzung der störendsten Lärmquelle in Innsbruck und Lärmbelästigung nach Quelle im Extremgruppenvergleich der Starkbelästigten

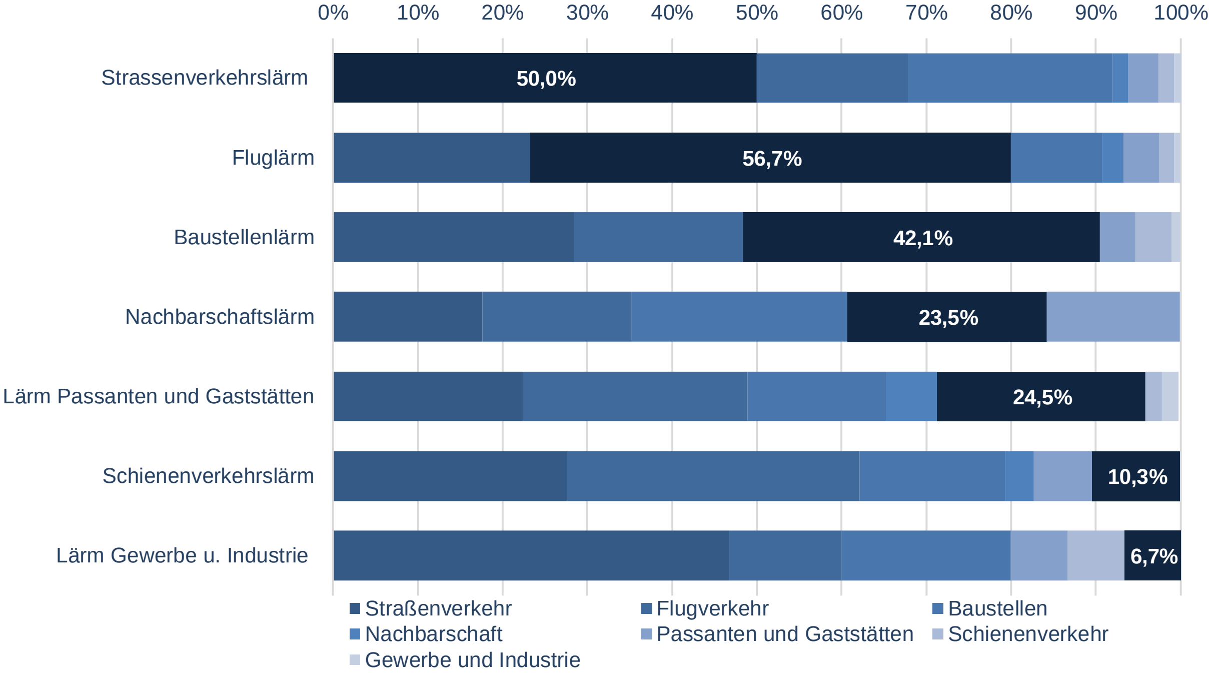

- Der Straßenverkehr als störendste Lärmquelle wird von den vom Straßenverkehr stark belästigten Befragten um rund 30% im Vergleich zum Gesamtkollektiv überschätzt.
- Der Fluglärm von den stark Fluglärmbelästigten um das 1,5-Fache.
- Der Baulärm von stark Baulärmbelästigten um das Doppelte.
- Der Lärm von Passanten und Gaststätten von stark von demselben Belästigten um das Vierfache.
- Der Schienenverkehrslärm von den stark Schienenverkehrslärmbelästigten um das 5,5-Fache.
- Der Nachbarschaftslärm von stark Nachbarschaftslärmbelästigten ebenfalls um das 5,5-Fache.
- Und der Lärm von Gewerbe und Industrie von stark von demselben Belästigten um das 22-Fache.

Tabelle 186: Einschätzung der störendsten Lärmquelle in Innsbruck u. Lärmbelästigung nach Quelle

		Einschätzung störendste Lärmquelle in Innsbruck (*)						
		**Straßen-verkehr**	**Schienen-verkehr**	**Flug-verkehr**	**Gewerbe und In-dustrie**	**Passanten und Gast-stätten**	**Bau-stellen**	**Nach-barschaft**
		Prozent	Prozent	Prozent	Prozent	Prozent	Prozent	Prozent
**Belästigung im Schlaf Lärm gesamt**	Gering belästigt	41,3%	0,9%	25,3%	0,2%	4,2%	24,7%	3,4%
	Mittelgradig belästigt	41,8%	3,6%	23,3%	0,4%	5,6%	21,7%	3,6%
	Stark belästigt	37,2%	1,3%	24,4%	1,3%	14,1%	14,1%	7,7%
**Belästigung Lärm gesamt**	Gering belästigt	43,0%	1,2%	23,7%	0,3%	4,4%	24,9%	2,6%
	Mittelgradig belästigt	40,4%	1,8%	25,5%	0,2%	6,1%	21,8%	4,2%
	Stark belästigt	38,3%	2,5%	24,2%	0,8%	5,0%	23,3%	5,8%
**Belästigung Fluglärm**	Gering belästigt	45,4%	1,7%	16,1%	0,2%	5,4%	26,9%	4,3%
	Mittelgradig belästigt	41,4%	1,6%	25,1%	0,3%	5,8%	22,3%	3,7%
	Stark belästigt	23,3%	1,7%	56,7%	0,8%	4,2%	10,8%	2,5%
**Belästigung Strassenver-kehrslärm**	Gering belästigt	36,6%	1,0%	28,1%	0,2%	5,3%	24,5%	4,3%
	Mittelgradig belästigt	44,3%	2,5%	22,2%	0,3%	6,1%	20,8%	3,9%
	Stark belästigt	50,0%	1,8%	17,9%	0,9%	3,6%	24,1%	1,8%
**Belästigung Baustellenlärm**	Gering belästigt	42,8%	1,5%	27,1%	0,3%	4,4%	19,4%	4,5%
	Mittelgradig belästigt	41,7%	1,2%	20,2%	0,0%	8,3%	25,0%	3,6%
	Stark belästigt	28,4%	4,2%	20,0%	1,1%	4,2%	42,1%	0,0%
**Belästigung Nachbarschafts-lärm**	Gering belästigt	42,7%	1,6%	25,0%	0,4%	4,4%	23,9%	1,9%
	Mittelgradig belästigt	41,3%	2,1%	25,5%	0,0%	6,0%	20,0%	5,1%
	Stark belästigt	17,6%	0,0%	17,6%	0,0%	15,7%	25,5%	23,5%
**Belästigung Lärm Passanten und Gaststätten**	Gering belästigt	42,4%	1,6%	26,0%	0,1%	3,2%	23,6%	3,0%
	Mittelgradig belästigt	41,0%	1,8%	20,3%	0,4%	7,9%	22,9%	5,7%
	Stark belästigt	22,4%	2,0%	26,5%	2,0%	24,5%	16,3%	6,1%
**Belästigung Schienenver-kehrslärm**	Gering belästigt	41,7%	0,9%	24,2%	0,3%	5,0%	24,1%	3,8%
	Mittelgradig belästigt	40,3%	3,9%	25,3%	0,6%	7,1%	18,8%	3,9%
	Stark belästigt	27,6%	10,3%	34,5%	0,0%	6,9%	17,2%	3,4%
**Belästigung Lärm Gewerbe u. Industrie**	Gering belästigt	41,0%	1,5%	24,9%	0,1%	5,3%	23,5%	3,8%
	Mittelgradig belästigt	40,8%	2,6%	25,0%	1,3%	6,6%	18,4%	5,3%
	Stark belästigt	46,7%	6,7%	13,3%	6,7%	6,7%	20,0%	0,0%

(*) ohne Antwortkategorie w.n. / k.A.

Sinnhaftigkeit von Maßnahmen zur Lärmreduktion in Innsbruck

1. Überblick Sinnhaftigkeit von Lärmreduktionsmaßnahmen

Mehr als zwei Drittel aller Befragten sind der Meinung, in Innsbruck sollten (weitere) Maßnahmen zur Lärmreduktion getroffen werden. Über 14% machen keine Angaben und knapp 19% halten (weitere) Maßnahmen zur Lärmreduktion nicht für zielführend:

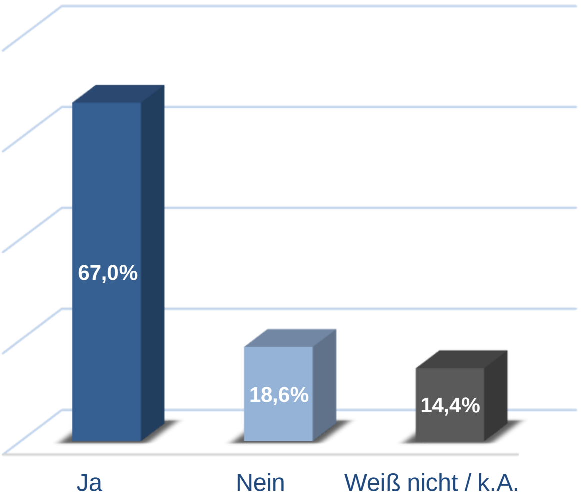

Abbildung 246: Sinnhaftigkeit (weiterer) Maßnahmen zur Lärmreduktion in Prozent

Werden nur diejenigen Befragten herangezogen, die diese Frage mit einem Ja oder einem Nein beantwortet haben, sprechen sich 78% für (weitere) Lärmreduktionsmaßnahmen aus, knapp 22% dagegen:

Tabelle 187: Sinnhaftigkeit (weiterer) Maßnahmen zur Lärmreduktion in Innsbruck (ohne w.n. / k.A.)

		Häufigkeit	Gültige Prozente	Kumulierte Prozente
Gültig	Ja	691	78,3	78,3
	Nein	192	21,7	100,0
	Gesamt	883	100,0	
Fehlend	Weiß nicht/ k.A.	148		
Gesamt		1031		

2. Sinnhaftigkeit von Lärmreduktionsmaßnahmen und ausgewählte Parameter

Frauen halten (weitere) Maßnahmen eher für sinnvoll als Männer.

Die jüngste Altersgruppe der Befragten sieht eine geringere Notwendigkeit für Lärmreduktionsmaßnahmen als die beiden anderen Altersgruppen.

Je höher der höchste erreichte Bildungsabschluss, desto eher werden Maßnahmen zur Lärmreduktion von den befragten Innsbruckerinnen und Innsbruckern befürwortet. Die Unterschiede im Antwortver-

halten sind wiederum vor allem zwischen Personen mit Pflichtschulabschluss und Akademikerinnen und Akademikern sehr ausgeprägt.

Probandinnen und Probanden, die Innsbruck im Vergleich mit anderen österreichischen Städten für lauter halten, sprechen sich häufiger für (weitere) Maßnahmen zur Lärmbekämpfung aus, als Befragte die Innsbruck insgesamt für ruhiger als andere vergleichbare Städte ansehen:

Abbildung 247: Sinnhaftigkeit von Maßnahmen zur Lärmreduktion und Lärmniveau von Innsbruck im Städtevergleich

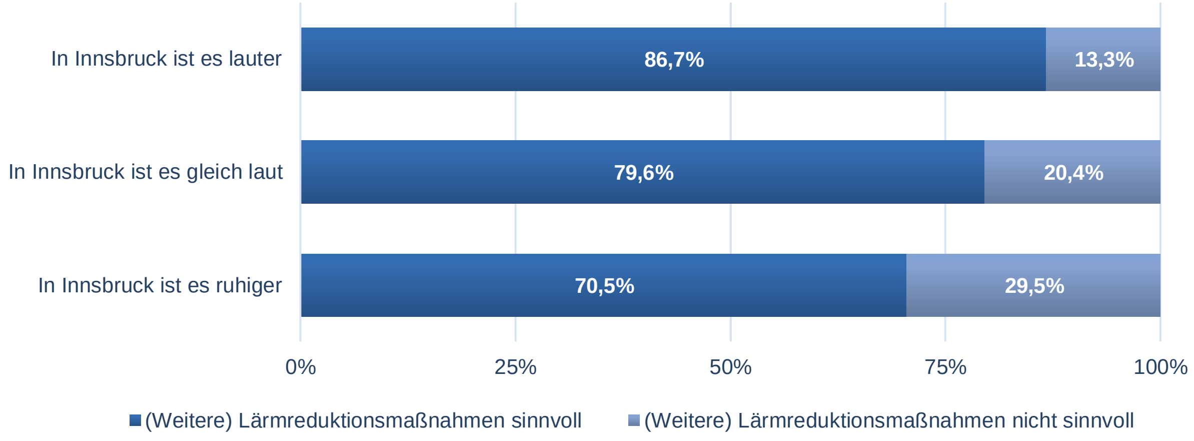

Befragte, welche ihre Lebensbedingungen als weniger gut oder durchschnittlich einstufen, sind weit häufiger für zusätzliche Maßnahmen als Personen, die ihre Wohn- und Lebensbedingungen als sehr gut beurteilen.

In Zusammenschau mit dem Gefühl der eigenen Ausgesetztheit gegenüber Lärm und der Einschätzung der persönlichen Lärmempfindlichkeit zeigt sich sowohl in den Extremgruppen- als auch in den Mittelwertvergleichen ein gewisser Schwellenwert: Eher geringfügig lärmempfindliche Befragte in den ersten 4 Antwortkategorien zeigen nur einen sehr moderaten Anstieg in der Zustimmung zur Notwendigkeit (weiterer) Lärmreduktionsmaßnahmen. Ab einem mittleren Niveau der eigenen Lärmempfindlichkeit, respektive dem Gefühl der Ausgesetztheit gegenüber Lärm, steigt die Befürwortung weiterer Maßnahmen stark an und bleibt auch bei den sich als stark lärmempfindlich / lärmexponiert einstufenden Befragten nahezu gleich auf demselben hohen Niveau:

Abbildung 248: Sinnhaftigkeit von Maßnahmen zur Lärmreduktion und Lärmempfindlichkeit im Mittelwertvergleich

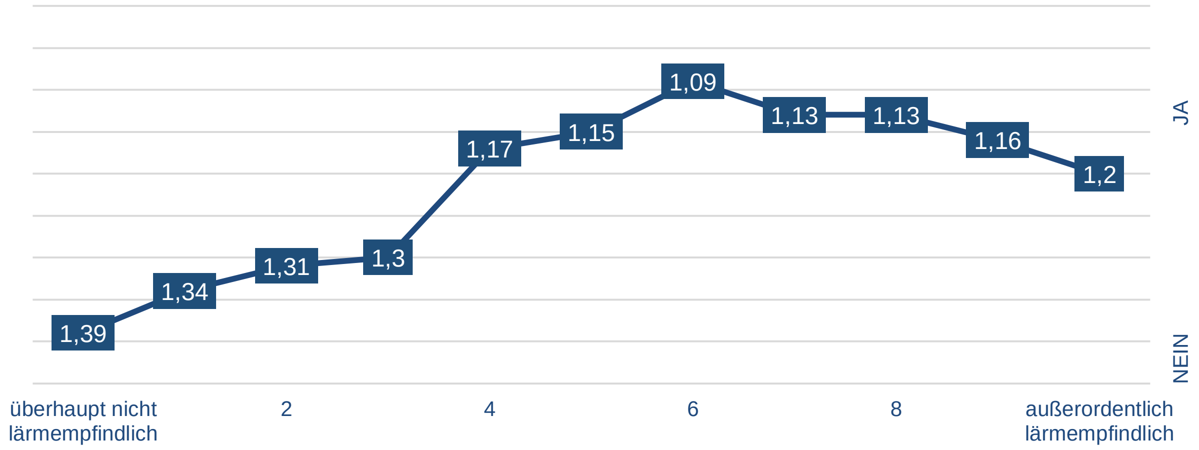

Tabelle 188: Sinnhaftigkeit von Maßnahmen zur Lärmreduktion und ausgewählte Parameter

		Notwendigkeit (weiterer) Maßnahmen zur Lärmreduktion (*)			
		Ja		Nein	
		Anzahl	Zeilen (%)	Anzahl	Zeilen (%)
**Geschlecht**	weiblich	372	81,0%	87	19,0%
	männlich	319	75,2%	105	24,8%
**Altersgruppen**	18 bis 40 Jahre	270	74,2%	94	25,8%
	41 bis 60 Jahre	228	82,9%	47	17,1%
	über 60 Jahre	193	79,1%	51	20,9%
**Bildungsschicht**	Primärer Schulabschluss	48	64,0%	27	36,0%
	Sekundärer Schulabschluss	353	76,4%	109	23,6%
	Tertiärer Schulabschluss	289	84,3%	54	15,7%
**Nachtschlaf**	Fenster geöffnet/gekippt	542	77,2%	160	22,8%
	Fenster geschlossen	145	83,3%	29	16,7%
**Lebensbedingungen (Summenscore)**	Nicht gut	5	100,0%	0	0,0%
	Durchschnittlich	483	82,4%	103	17,6%
	Sehr gut	203	69,5%	89	30,5%
**Lautheit Innsbruck im Vergleich zu anderen Städten**	Ruhiger	172	70,5%	72	29,5%
	Gleich	367	79,6%	94	20,4%
	Lauter	144	86,7%	22	13,3%
**Lärmempfindlichkeit**	Nicht lärmempfindlich	152	66,1%	78	33,9%
	Durchschnittlich	405	82,0%	89	18,0%
	Sehr lärmempfindlich	134	84,3%	25	15,7%
**Ausgesetztheit Lärm gesamt**	Gering exponiert	141	66,2%	72	33,8%
	Mittelgradig exponiert	431	80,3%	106	19,7%
	Stark exponiert	119	89,5%	14	10,5%

(*) ohne Antwortkategorie w.n. / k.A.

3. Sinnhaftigkeit von Lärmreduktionsmaßnahmen und Lärmbelastung

Die gegebene Lärmbelastung im Wohnumfeld der Befragten ist nicht bzw. nur in sehr geringfügigem Ausmaß ausschlaggebend für den Grad der Zustimmung zu (weiteren) Lärmreduktionsmaßnahmen:

Tabelle 189: Sinnhaftigkeit von Maßnahmen zur Lärmreduktion und Lärmbelastung nach Quelle

		Notwendigkeit (weiterer) Maßnahmen zur Lärmreduktion (*)			
		Ja		Nein	
		Anzahl	Zeilen (%)	Anzahl	Zeilen (%)
**Straßenlärm 24h**	Stark belastet >55dB	364	79,8%	92	20,2%
	Mittelgradig belastet 45-55dB	258	76,8%	78	23,2%

	Gering belastet <45dB	69	75,8%	22	24,2%
	Stark belastet >55dB	85	82,5%	18	17,5%
**Schienenlärm 24h**	Mittelgradig belastet 45-55dB	221	79,8%	56	20,2%
	Gering belastet <45dB	385	76,5%	118	23,5%
	Stark belastet >55dB	79	78,2%	22	21,8%
**Fluglärm 24h**	Mittelgradig belastet 45-55dB	259	81,4%	59	18,6%
	Gering belastet <45dB	353	76,1%	111	23,9%

(*) ohne Antwortkategorie w.n. / k.A.

4. Sinnhaftigkeit von Lärmreduktionsmaßnahmen und Lärmbelästigung

Im Gegensatz zur vergleichsweise geringen Beziehung zwischen der gegebenen Lärmbelastung nach Quelle im Wohnumfeld der Befragten und dem Ausmaß der Zustimmung zur (weiteren) Maßnahmen zur Lärmreduktion in Innsbruck, zeigt sich ein ausgeprägter Bezug zur Einschätzung der eigenen Belästigung durch Lärm.

Die Befürwortung (weiterer) Maßnahmen zur Lärmreduktion in Innsbruck unterscheidet sich deutlich je nach Belästigungsgrad der Befragten. Die vom Lärm insgesamt nur gering belästigten Befragten, erachten zu 65% weitere Maßnahmen für notwendig, dieser Grad der Zustimmung steigt bei den Starkbelästigten auf 90%.

Dabei zeigt sich auch erneut eine erhöhte Sensibilität auf Lärmbelästigung im Schlaf:

Abbildung 249: Sinnhaftigkeit von Maßnahmen zur Lärmreduktion und Lärmbelästigung gesamt und im Schlaf (gruppiert) im Vergleich

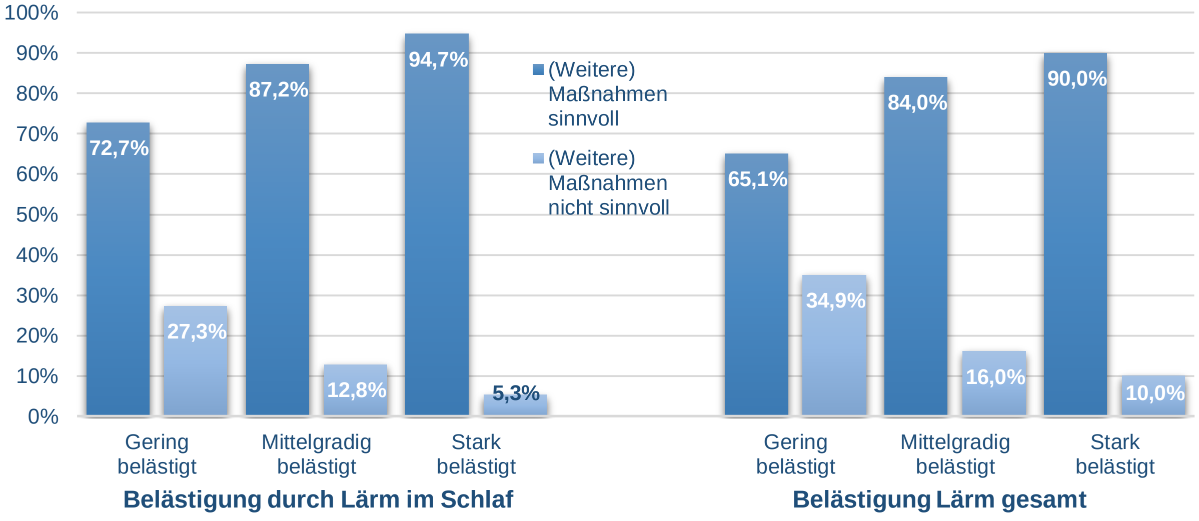

(*) ohne Antwortkategorie w.n. / k.A.

Die deutlich erhöhte Zustimmung der Starkbelästigten zu Lärmreduktionsmaßnahmen gestaltet sich unabhängig von der Art und Quelle der Lärmbelästigung. Drei Viertel der Befragten, die sich von den einzelnen Lärmquellen nur gering belästigt / gestört fühlen, sprechen sich im Mittel für (weitere) Lärm-

reduktionsmaßnahmen in Innsbruck aus. Dieser Anteil steigt bei sich stark belästigt Fühlenden auf im Durchschnitt 91%:

Abbildung 250: Sinnhaftigkeit von Maßnahmen zur Lärmreduktion und Mittelwerte der Lärmbelästigung (alle Quellen) in Prozent

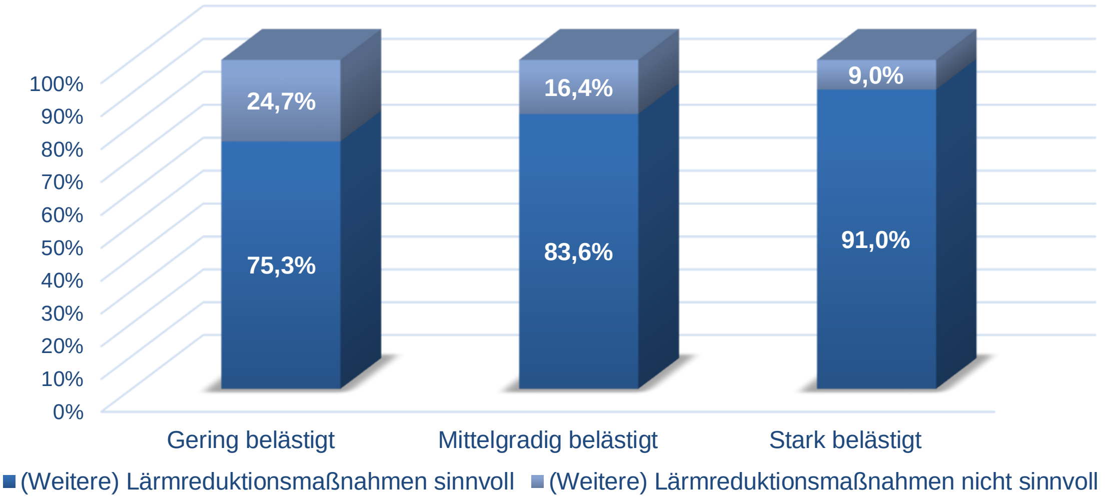

(*) ohne Antwortkategorie w.n. / k.A.

Tabelle 190: Sinnhaftigkeit von Maßnahmen zur Lärmreduktion und Lärmbelästigung nach Quelle

		Notwendigkeit (weiterer) Maßnahmen zur Lärmreduktion (*)			
		Ja		Nein	
		Anzahl	Zeilen (%)	Anzahl	Zeilen (%)
**Belästigung im Schlaf Lärm gesamt**	Gering belästigt	423	72,7%	159	27,3%
	Mittelgradig belästigt	197	87,2%	29	12,8%
	Stark belästigt	71	94,7%	4	5,3%
**Belästigung Lärm gesamt**	Gering belästigt	200	65,1%	107	34,9%
	Mittelgradig belästigt	383	84,0%	73	16,0%
	Stark belästigt	108	90,0%	12	10,0%
**Belästigung Fluglärm**	Gering belästigt	291	70,3%	123	29,7%
	Mittelgradig belästigt	294	83,1%	60	16,9%
	Stark belästigt	106	92,2%	9	7,8%
**Belästigung Straßenverkehrslärm**	Gering belästigt	322	70,9%	132	29,1%
	Mittelgradig belästigt	268	84,3%	50	15,7%
	Stark belästigt	101	91,0%	10	9,0%
**Belästigung Baustellenlärm**	Gering belästigt	435	77,1%	129	22,9%
	Mittelgradig belästigt	179	79,2%	47	20,8%
	Stark belästigt	76	82,6%	16	17,4%
**Belästigung Nachbarschaftslärm**	Gering belästigt	467	75,4%	152	24,6%
	Mittelgradig belästigt	181	83,4%	36	16,6%
	Stark belästigt	43	91,5%	4	8,5%
**Belästigung**	Gering belästigt	476	75,8%	152	24,2%

**Lärm von Passanten und Gaststätten**	Mittelgradig belästigt	171	82,6%	36	17,4%
	Stark belästigt	44	91,7%	4	8,3%
**Belästigung Schienenverkehrslärm**	Gering belästigt	544	76,5%	167	23,5%
	Mittelgradig belästigt	121	83,4%	24	16,6%
	Stark belästigt	26	96,3%	1	3,7%
**Belästigung Erschütterungen**	Gering belästigt	554	76,7%	168	23,3%
	Mittelgradig belästigt	109	84,5%	20	15,5%
	Stark belästigt	28	87,5%	4	12,5%
**Belästigung Lärm aus Gewerbe u. Industrie**	Gering belästigt	615	77,2%	182	22,8%
	Mittelgradig belästigt	62	86,1%	10	13,9%
	Stark belästigt	14	100,0%	0	0,0%
**Belästigung Autobahnlärm**	Gering belästigt	623	77,5%	181	22,5%
	Mittelgradig belästigt	49	86,0%	8	14,0%
	Stark belästigt	19	86,4%	3	13,6%

(*) ohne Antwortkategorie w.n. / k.A.

5. Einzelmaßnahmen zur Lärmreduktion in Innsbruck

Die 691 befragten Innsbruckerinnen und Innsbrucker (67% des befragten Kollektivs), welche (weitere) Maßnahmen zur Lärmreduktion in der Stadt für sinnvoll halten, wurden weitergehend nach ihrer Meinung zu einzelnen Maßnahmenbereichen befragt:

Die Schaffung, der Schutz und der Erhalt lärmarmer Bereiche in der Stadt Innsbruck wird von den Befragten am häufigsten als sinnvolle Maßnahme befürwortet (siehe dazu vor allem auch das Unterkapitel *Ruhige Gebiete* im Kapitel *Gesamtlärmbetrachtung Innsbruck*). Generell werden vor allem Förderungsmaßnahmen für sinnvoll erachtet, die von deutlich über 80% derjenigen Befragten, die Maßnahmen zur Lärmreduktion für zweckmäßig halten, angeregt werden:

Abbildung 251: Befürwortung von Einzelmaßnahmen zur Lärmreduktion in Innsbruck in Prozent

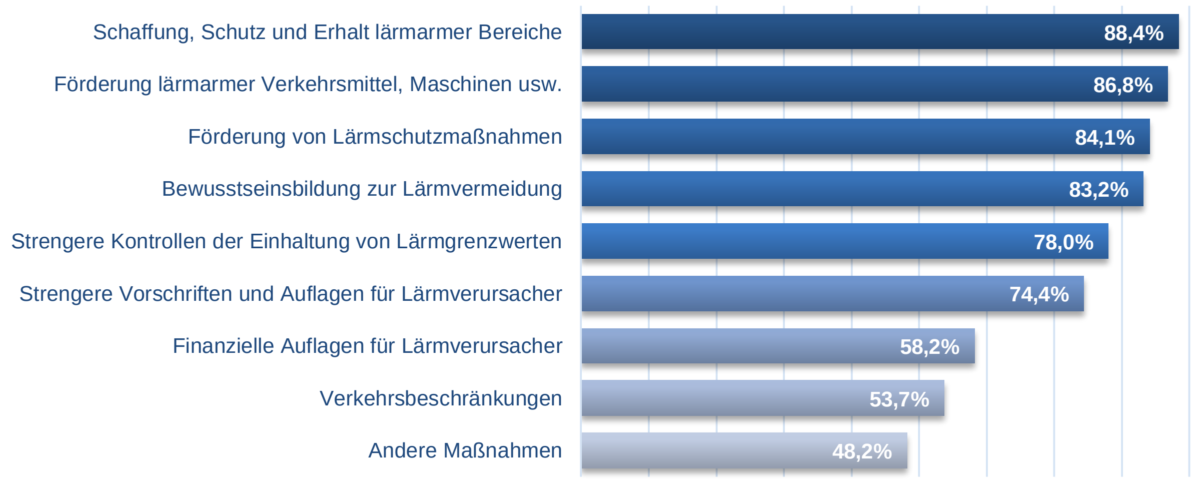

Die Bewusstseinsbildung zur Lärmvermeidung wird ebenfalls von über 80% aus dieser Subgruppe für wichtig erachtet, erhält aber geringere Zustimmungswerte als die Förderungsmaßnahmen auf den ersten drei Plätzen. Strengere Kontrollen der Einhaltung von Lärmgrenzwerten, sowie strengere Vorschriften und Auflagen für Lärmverursacherinnen und –Verursacher werden von rund drei Vierteln gutgeheißen. Finanzielle Auflagen für Lärmverursacherinnen und –Verursacher, sowie Verkehrsbeschränkungen hält knapp mehr als die Hälfte der Maßnahmenbefürworterinnen und –Befürworter für sinnvoll. Bezogen auf das Gesamtkollektiv aller 1031 befragten Innsbruckerinnen und Innsbrucker befürwortet über ein Drittel auch Verkehrsbeschränkungen und finanzielle Auflagen:

Tabelle 191: Sinnhaftigkeit Einzelmaßnahmen zur Lärmreduktion in Innsbruck

		Anzahl N (*)	Prozent N (*)	Prozent Gesamtanzahl
**Förderung lärmarmer Verkehrsmittel, Maschinen usw.**	Ja	600	86,8%	58,2%
	Nein	81	11,7%	7,9%
	Weiß nicht / k.A.	10	1,4%	1,0%
**Strengere Vorschriften und Auflagen für Lärmverursacher**	Ja	514	74,4%	49,9%
	Nein	170	24,6%	16,5%
	Weiß nicht/ k.A.	7	1,0%	0,7%
**Förderung von Lärmschutzmaßnahmen**	Ja	581	84,1%	56,4%
	Nein	97	14,0%	9,4%
	Weiß nicht/ k.A.	13	1,9%	1,3%
**Strengere Kontrollen der Einhaltung von Lärmgrenzwerten**	Ja	539	78,0%	52,3%
	Nein	135	19,5%	13,1%
	Weiß nicht/ k.A.	17	2,5%	1,6%
**Bewusstseinsbildung zur Lärmvermeidung**	Ja	575	83,2%	55,8%
	Nein	107	15,5%	10,4%
	Weiß nicht/ k.A.	9	1,3%	0,9%
**Verkehrsbeschränkungen**	Ja	371	53,7%	36,0%
	Nein	303	43,8%	29,4%
	Weiß nicht/ k.A.	17	2,5%	1,6%
**Finanzielle Auflagen für Lärmverursacher**	Ja	402	58,2%	39,0%
	Nein	277	40,1%	26,9%
	Weiß nicht/ k.A.	12	1,7%	1,2%
**Schaffung, Schutz und Erhalt lärmarmer Bereiche**	Ja	611	88,4%	59,3%
	Nein	65	9,4%	6,3%
	Weiß nicht/ k.A.	15	2,2%	1,5%
**Andere Maßnahmen**	Ja	333	48,2%	32,3%
	Nein	319	46,2%	30,9%
	Weiß nicht/ k.A.	39	5,6%	3,8%

(*) N entspricht der Subgruppe der 883 Befragten, die (weitere) Lärmreduktionsmaßnahmen für sinnvoll halten.

VERZEICHNISSE

Abkürzungsverzeichnis

AdTLR	Amt der Tiroler Landesregierung
Anm.	Anmerkung
Bezugszeit	Zeitraum, auf den der Beurteilungspegel bezogen wird:<br>Tag: die 13 Tagstunden von 6:00 bis 19:00 Uhr<br>Abend: die drei Abendstunden von 19:00 bis 22:00 Uhr<br>Nacht: die acht Nachtstunde von 22:00 bis 6:00 Uhr
Bf	Bahnhof
BGBl.	Bundesgesetzblatt
BMVIT	Bundesministerium für Verkehr, Innovation und Technologie
ca.	circa
dB	Dezibel
dB(A)	Dezibel, A-bewerteter Schalldruckpegel
DIN	Deutsches Institut für Normung e.V.
EEA	European Environment Agency
EFRE	Europäischer Fonds für regionale Entwicklung
EG	Erdgeschoß
END	Environmental noise directive
etc.	etcetera
EU	Europäische Union
EU SILC	EU Community Statistics on Income and Living Conditions
h	Stunde
HFA	Health for All
ICBEN	International Commission on Biological Effects of Noise
idgF	in der geltenden/gültigen Fassung
ILO	International Labour Organisation
JMW	Jahresmittelwert
Kfz	Kraftfahrzeug
kN	Kilonewton

$L_{A,eq}$ Energieäquivalenter A-bewerteter Dauerschallpegel

$L_{A,ES}$ Effektbezogener Substitutionspegel nach VDI 3722 Blatt 2, beschreibt die energetische Summe der einzelnen, nach den Belästigungsäquivalenten renormierten Ersatzpegeln

L_{day} A-bewerteter äquivalenter Dauerschallpegel, Beurteilungszeitraum 1 Jahr, Bestimmung an allen Kalendertagen am Tag (06:00 bis 19:00 Uhr)

L_{den} Lärmindex für die allgemeine Lärmbelastung

LEF-K Kurzfragebogen zur Erfassung der Lärmempfindlichkeit

L_{eq} Energieäquivalenter Dauerschallpegel. Einzahlenangabe, die zur Beschreibung von Schallereignissen mit schwankendem Schalldruckpegel dient. Der energieäquivalente Dauerschallpegel wird als jener Schalldruckpegel errechnet, der bei dauernder Einwirkung dem unterbrochenen Geräusch oder Geräusch mit schwankendem Schalldruckpegel energieäquivalent ist

$L_{evening}$: A-bewerteter äquivalenter Dauerschallpegel, Beurteilungszeitraum 1 Jahr, Bestimmung an allen Kalendertagen am Abend (19:00 bis 22:00 Uhr)

Lkw Lastkraftwagen

L_{night} / L_n A-bewerteter äquivalenter Dauerschallpegel, Beurteilungszeitraum 1 Jahr, Bestimmung an allen Kalendertagen in der Nacht (22:00 bis 6:00 Uhr)

L_P Schalldruckpegel: zehnfacher dekadischer Logarithmus des Verhältnisses der Quadrate des Effektivwertes des Schalldrucks p und des Bezugsschalldrucks p_0
$L_P = 10 \log (p^2 / p_0^2)$ in Dezibel (dB) mit $p_0 = 20\ \mu Pa$
Sofern eine Verwechslung mit dem Schallleistungspegel nicht zu erwarten ist, wird vielfach statt Schalldruckpegel die vereinfachte Bezeichnung Schallpegel verwendet

$L_{p,A}$ A-bewerteter Schalldruckpegel der mit A-Bewertung, festgelegt in der Verordnung des Bundesministers für Eich- und Vermessungswesen vom 29.Juni 1979, ermittelte Schalldruckpegel. Die A-Bewertung stellt eine gewisse Annäherung an die Lautheitsempfindung des Menschen dar. Der A-bewertete Schalldruckpegel wird in der Regel für die Beschreibung der Schallimmissionen verwendet

L_r Beurteilungspegel: der auf die Bezugszeit bezogene A-bewertete energieäquivalente Dauerschallpegel des zu beurteilenden Geräusches – wenn nötig – mit Anpassungswerten versehen

LSW Lärmschutzwand

LW Schallleistungspegel

$LW,_A$ Schallleistungspegel, A-bewertet

L_Z Anpassungswert: Pegelzu- oder Abschlag für bestimmte Geräuschcharakteristika oder Geräuschquellen auf Grund der Lästigkeit

m Meter

Max. Maximum

Messzeit Zeitraum, innerhalb dessen Schalldruckpegel messtechnisch ermittelt werden

Min. Minimum

MW Mittelwert

N	Stichprobenumfang
N_B:	Beeinträchtigungskenngröße nach VDI 3722 Blatt 2 zur Aussage über die Anzahl der durch Verkehrsgeräusche im Untersuchungsgebiet beeinträchtigten Personen
Nr.	Nummer
ÖAL	Österreichischer Arbeitsring für Lärmbekämpfung
ÖBB	Österreichische Bundesbahnen
OG	Obergeschoß
ÖNORM	Österreichische Norm
PKW	Personenkraftwagen
SA	Standardabweichung
S.	Seite
s	Sekunde
s.	siehe
SchIV	Schienenverkehrslärm-Immissionsschutzverordnung
SV	Sachverständige(r)
TB	Technischer Bericht
TBO	Tiroler Bauordnung
TIRIS	Tiroler Raumordnungsinformationssystem
TROG	Tiroler Raumordnungsgesetz
usw.	und so weiter
u.v.a.m.	und viele andere mehr
UVE	Umweltverträglichkeitserklärung
UVP	Umweltverträglichkeitsprüfung
UVP-G	Umweltverträglichkeitsprüfungsgesetz
V	Geschwindigkeit
VDI	Verein Deutscher Ingenieure
vgl.	vergleiche
V_{max}	(maximale) Betriebsgeschwindigkeit
VO	Verordnung
WHO	World Health Organisation

Tabellenverzeichnis

Tabelle 1: Messergebnisse temporäre Dauermessstelle Kranebitten (alle Quellen) ... 45
Tabelle 2: Teillärmpegel temporäre Dauermessstelle Kranebitten ... 46
Tabelle 3: Vergleich der Mess- mit den Berechnungsergebnissen ... 46
Tabelle 4: Anzahl der belasteten Gebäude, Einwohner und Wohnungen nach Quelle ... 48
Tabelle 5: Anteile der belasteten Gebäude, Einwohnerinnen und Einwohner und Wohnungen bezogen auf ganz Innsbruck nach Quelle in Prozent ... 49
Tabelle 6: Mittelwerte der 24h-Lärmbelastung L_{den} nach Quelle in dB ... 54
Tabelle 7: Mittelwerte der 24h-Lärmbelastung L_{den} der Innsbrucker Katastralgemeinden nach Quelle in dB (Innsbruck gesamt und Befragte gegenübergestellt) ... 56
Tabelle 8: Straßenverkehrslärmbelastung L_{den} in 3 Belastungskategorien nach Katastralgemeinde ... 58
Tabelle 9: Fluglärmbelastung L_{den} in 3 Belastungskategorien nach Katastralgemeinde ... 58
Tabelle 10: Schienenverkehrslärmbelastung L_{den} in 3 Belastungskategorien nach Katastralgemeinde ... 58
Tabelle 11: Autobahnlärmbelastung L_{den} in 3 Belastungskategorien nach Katastralgemeinde ... 59
Tabelle 12: Mittelwerte der nächtlichen Lärmbelastung L_n nach Quelle in dB ... 60
Tabelle 13: Mittelwerte der nächtlichen Lärmbelastung L_n der Innsbrucker Katastralgemeinden nach Quelle in dB (Innsbruck gesamt und Befragte gegenübergestellt) ... 62
Tabelle 14: Nächtliche Straßenlärmbelastung L_n in 3 Belastungskategorien nach Katastralgemeinde ... 64
Tabelle 15: Nächtliche Schienenlärmbelastung L_n in 3 Belastungskategorien nach Katastralgemeinde ... 64
Tabelle 16: Nächtliche Autobahnlärmbelastung L_n in 3 Belastungskategorien n. Katastralgemeinde ... 64
Tabelle 17: Nächtliche Fluglärmbelastung L_n in 3 Belastungskategorien nach Katastralgemeinde ... 65
Tabelle 18: Soll-Ist-Vergleich der Altersgruppen Stichprobe ↔ Interviews ... 83
Tabelle 19: Soll-Ist-Vergleich der Geschlechterverteilung Stichprobe ↔ Interviews ... 83
Tabelle 20: Schlüssel Katastralgemeinden Innsbruck (nach Einwohneranzahl geordnet) ... 84
Tabelle 21: Soll-Ist-Vergleich der Katastralgemeinden Stichprobe ↔ Interviews ... 85
Tabelle 22: Matrix Lärmkorridore ... 86
Tabelle 23: Rücklauf Befragungen Gesamtlärmbetrachtung Innsbruck ... 88
Tabelle 24: Mittelwert Alter ... 93
Tabelle 25: Höchster bisher erreichter Bildungsabschluss ... 94
Tabelle 26: Kreuztabelle Geschlecht und derzeitige berufliche Stellung in Prozent ... 96
Tabelle 27: Wohndauer im selben Haus / derselben Wohnung ... 98
Tabelle 28: Art des Wohnhauses ... 99
Tabelle 29: Anzahl an Kindern unter 18 Jahren im selben Haushalt ... 100
Tabelle 30: Stockwerk des Schlafzimmers ... 100
Tabelle 31: Schutz vor Verkehrslärm durch Innenhof bzw. lärmabgewandte Seite ... 101
Tabelle 32: Verbaute Lärmschutzfenster in den letzten 10 Jahren ... 102
Tabelle 33: Auspendleranteil an den berufstätigen bzw. sich in Ausbildung befindlichen befragten Innsbruckerinnen und Innsbruckern (ohne Nichtzutreffende) ... 103
Tabelle 34: Häufigkeit der Nutzung von Kraftfahrzeugen als Fahrerin / Fahrer gruppiert ... 104
Tabelle 35: Nutzung von Kfz als Fahrerin / Fahrer und ausgewählte Variablen (gruppiert) ... 105
Tabelle 36: Häufigkeit der Nutzung öffentlicher Verkehrsmittel innerhalb der Stadt gruppiert ... 106
Tabelle 37: Nutzung öffentl. Verkehrsmittel in der Stadt und ausgewählte Variablen (gruppiert) ... 106
Tabelle 38: Häufigkeit der Nutzung öffentl. Verkehrsmittel außerhalb von Innsbruck gruppiert ... 107
Tabelle 39: Nutzung öffentl. Verkehrsmittel außerhalb der Stadt u. ausgew. Variablen (gruppiert) ... 107

Tabelle 40: Häufigkeit der Nutzung eines Fahrrads gruppiert ... 109
Tabelle 41: Nutzung eines Fahrrads und ausgewählte Variablen (gruppiert) ... 109
Tabelle 42: Häufigkeit fußläufiger Wege gruppiert ... 110
Tabelle 43: Fußläufige Wege und ausgewählte Variablen (gruppiert) ... 110
Tabelle 44: Häufigkeit der Nutzung eines Flugzeuges gruppiert ... 111
Tabelle 45: Nutzung eines Flugzeugs und ausgewählte Variablen (gruppiert) ... 112
Tabelle 46: Beurteilung der Wohnbedingungen gruppiert ... 114
Tabelle 47: Beurteilung der Nachbarschaftsbeziehungen gruppiert ... 115
Tabelle 48: Beurteilung der Sicherheit gruppiert ... 115
Tabelle 49: Beurteilung der Landschaft, Natur, Parks und Grünflächen gruppiert ... 116
Tabelle 50: Beurteilung der Luftqualität gruppiert ... 117
Tabelle 51: Beurteilung der öffentlichen Verkehrsverbindungen gruppiert ... 118
Tabelle 52: Beurteilung der Bedingungen für das Aufwachsen von Kinder gruppiert ... 118
Tabelle 53: Beurteilung der Verbundenheit zur Stadt Innsbruck gruppiert ... 119
Tabelle 54: Lebensbedingungen und Katastralgemeinde im Mittelwertvergleich ... 120
Tabelle 55: Lebensbedingungen und Geschlecht im Mittelwertvergleich ... 121
Tabelle 56: Lebensbedingungen und Lärmempfindlichkeit im Mittelwertvergleich ... 123
Tabelle 57: Lebensbedingungen und 24h-Schienenlärmbelastung L_{den} im Mittelwertvergleich ... 125
Tabelle 58: Lebensbedingungen und 24h-Fluglärmbelastung L_{den} im Mittelwertvergleich ... 126
Tabelle 59: Lebensbedingungen und Straßenlärmexpositionsempfinden im Mittelwertvergleich ... 127
Tabelle 60: Lebensbedingungen und Belästigung durch Nachbarschaftslärm im Mittelwertvergleich ... 131
Tabelle 61: Lebensbedingungen und Belästigung durch Baustellenlärm im Mittelwertvergleich ... 131
Tabelle 62: Lebensbedingungen und Belästigung durch Lärm von Passanten und Gaststätten im Mittelwertvergleich ... 132
Tabelle 63: Lebensbedingungen und Belästigung durch Erschütterungen im Mittelwertvergleich ... 132
Tabelle 64: Lebensbedingungen und Belästigung durch Lärm aus Gewerbe und Industrie im Mittelwertvergleich ... 133
Tabelle 65: Zufriedenheit mit der eigenen Wohnsituation gruppiert ... 134
Tabelle 66: Zufriedenheit mit der eigenen Wohnsituation u. Katastralgemeinde im Mittelwertvergleich ... 135
Tabelle 67: Zufriedenheit mit der Wohnsituation und ausgewählte Variablen (gruppiert) ... 136
Tabelle 68: Zufriedenheit mit der Wohnsituation und 24h-Straßenverkehrslärmbelastung L_{den} in 10-dB Schritten im Mittelwertvergleich ... 138
Tabelle 69: Zufriedenheit mit der Wohnsituation und 24h-Flugverkehrslärmbelastung L_{den} in 10-dB Schritten im Mittelwertvergleich ... 139
Tabelle 70: Zufriedenheit mit der Wohnsituation und 24h-Schienenverkehrslärmbelastung L_{den} in 10-dB Schritten im Mittelwertvergleich ... 139
Tabelle 71: Zufriedenheit mit der Wohnsituation und nächtliche Flugverkehrslärmbelastung L_n in 10-dB Schritten im Mittelwertvergleich ... 140
Tabelle 72: Zufriedenheit mit der Wohnsituation und nächtliche Schienenverkehrslärmbelastung L_n in 10-dB Schritten im Mittelwertvergleich ... 141
Tabelle 73: Zufriedenheit mit der Wohnsituation und Lärmbelästigung gesamt im Mittelwertvergleich ... 143
Tabelle 74: Einschätzung des eigenen Gesundheitszustandes gruppiert ... 145
Tabelle 75: Einschätzung des eigenen Gesundheitszustandes (gruppiert) und ausgewählte Variablen ... 146
Tabelle 76: Bivariate Korrelation zu Gesundheitszustand und 24h-Lärmbelastung L_{den} nach Quelle ... 147
Tabelle 77: Gesundheitszustand und Lärmexpositionsempfinden nach Quelle (gruppier) ... 147
Tabelle 78: Einschätzung der persönlichen Lebensqualität gruppiert ... 150

Tabelle 79: Einschätzung der eigenen Lebensqualität (gruppiert) und ausgewählte Variablen 151
Tabelle 80: Bivariate Korrelation zu Lebensqualität und 24h-Lärmbelastung L_{den} nach Quelle 152
Tabelle 81: Lebensqualität und Lärmexpositionsempfinden nach Quelle (gruppier) 153
Tabelle 82: Einschätzung der eigenen Lärmempfindlichkeit gruppiert 156
Tabelle 83: Einschätzung der eigenen Lärmempfindlichkeit (gruppiert) und ausgewählte Variablen 157
Tabelle 84: Bivariate Korrelation zu Lärmempfindlichkeit und 24h-Lärmbelastung L_{den} nach Quelle 158
Tabelle 85: Lärmempfindlichkeit und 24h-Straßenlärm L_{den} in 10-dB Schritten im Mittelwertvergleich 158
Tabelle 86: Lärmempfindlichkeit und 24h-Fluglärm L_{den} in 10-dB Schritten im Mittelwertvergleich............ 158
Tabelle 87: Lärmempfindlichkeit und 24h-Schienenlärm L_{den} in 10-dB Schritten im Mittelwertvergleich 159
Tabelle 88: Lärmempfindlichkeit und Lärmexpositionsempfinden nach Quelle (gruppier) 159
Tabelle 89: Lärmempfindlichkeit und Belästigung durch Verkehrslärm nach Quelle gruppiert 161
Tabelle 90: Einfluss von Wind- und Wetterverhältnissen auf die Lärmbelastung gruppiert 163
Tabelle 91: Einfluss von Wind- und Wetterverhältnissen auf die Lärmbelastung und ausgewählte Parameter (gruppiert, ohne Antwortkategorie k.A./w.n.) 164
Tabelle 92: Nachtschlaf bei geöffnetem oder gekipptem Fenster gruppiert 166
Tabelle 93: Nachtschlaf bei geöffnetem oder gekipptem Fenster und ausgewählte Parameter (gruppiert, ohne Antwortkategorie k.A./w.n.) 167
Tabelle 94: Nachtschlaf bei geöffnetem oder gekipptem Fenster und nächtliche Lärmbelastung L_n nach Quelle (gruppiert) 168
Tabelle 95: Mittelwerte der empfundenen Exposition nach Quelle 170
Tabelle 96: Empfundene Lärmexposition insgesamt gruppiert 174
Tabelle 97: Empfundene Lärmexposition insgesamt und ausgewählte Parameter (gruppiert) 175
Tabelle 98: Empfundene Flugverkehrslärmexposition gruppiert 177
Tabelle 99: Empfundene Fluglärmexposition und ausgewählte Parameter (gruppiert) 179
Tabelle 100: Empfundene Straßenverkehrslärmexposition gruppiert 181
Tabelle 101: Empfundene Straßenlärmexposition und ausgewählte Parameter (gruppiert) 183
Tabelle 102: Empfundene Baustellenlärmexposition gruppiert 185
Tabelle 103: Empfundene Baustellenlärmexposition und ausgewählte Parameter (gruppiert).................... 186
Tabelle 104: Empfundene Nachbarschaftslärmexposition gruppiert 187
Tabelle 105: Empfundene Nachbarschaftslärmexposition und ausgewählte Parameter (gruppiert)............ 188
Tabelle 106: Empfundene Lärmexposition durch Passanten und Gaststätten gruppiert 190
Tabelle 107: Empfundene Lärmexposition durch Passanten und Gaststätten und ausgewählte Parameter (gruppiert) 191
Tabelle 108: Empfundene Exposition gegenüber Erschütterungen gruppiert 193
Tabelle 109: Empfundene Erschütterungsexposition und ausgewählte Parameter (gruppiert) 194
Tabelle 110: Empfundene Schienenverkehrslärmexposition gruppiert 196
Tabelle 111: Empfundene Schienenlärmexposition und ausgewählte Parameter (gruppiert) 198
Tabelle 112: Empfundene Autobahnlärmexposition gruppiert.. 199
Tabelle 113: Empfundene Autobahnlärmexposition und ausgewählte Parameter (gruppiert)..................... 200
Tabelle 114: Empfundene Lärmexposition durch Gewerbe und Industrie gruppiert 202
Tabelle 115: Empfundene Lärmexposition durch Gewerbe und Industrie und ausgewählte Parameter 202
Tabelle 116: Mittelwerte der Belästigung nach Quelle 204
Tabelle 117: Belästigung nach Lärmquelle und Katastralgemeinde im Mittelwertvergleich 207
Tabelle 118: Belästigung durch Lärm insgesamt gruppiert 210
Tabelle 119: Bedingungen für das Aufwachsen von Kindern und Belästigung durch Lärm insgesamt........ 212
Tabelle 120: Belästigung durch Lärm insgesamt und ausgewählte Parameter (gruppiert) 213

Tabelle 121: Belästigung durch Fluglärm gruppiert ... 215
Tabelle 122: Belästigung durch Fluglärm und ausgewählte Parameter (gruppiert) ... 217
Tabelle 123: Belästigung durch Straßenverkehrslärm gruppiert ... 219
Tabelle 124: Geschlecht und Belästigung durch Straßenverkehrslärm gruppiert ... 221
Tabelle 125: Belästigung durch Straßenverkehrslärm und ausgewählte Parameter (gruppiert) ... 222
Tabelle 126: Belästigung durch Baustellenlärm gruppiert ... 224
Tabelle 127: Belästigung durch Baustellenlärm und ausgewählte Parameter (gruppiert) ... 225
Tabelle 128: Belästigung durch Nachbarschaftslärm gruppiert ... 227
Tabelle 129: Belästigung durch Nachbarschaftslärm und ausgewählte Parameter (gruppiert) ... 228
Tabelle 130: Belästigung durch Lärm von Passanten und Gaststätten gruppiert ... 230
Tabelle 131: Belästigung durch Lärm von Passanten und Gaststätten und ausgewählte Parameter ... 230
Tabelle 132: Belästigung durch Schienenverkehrslärm gruppiert ... 232
Tabelle 133: Schienenverkehrslärmbelästigung und Bildungsschicht im Mittelwertvergleich ... 234
Tabelle 134: Belästigung durch Schienenverkehrslärm und ausgewählte Parameter (gruppiert) ... 234
Tabelle 135: Belästigung durch Erschütterungen gruppiert ... 236
Tabelle 136: Belästigung durch Erschütterungen und ausgewählte Parameter (gruppiert) ... 237
Tabelle 137: Belästigung durch Lärm aus Gewerbe u. Industrie gruppiert ... 239
Tabelle 138: Belästigung durch Lärm aus Gewerbe und Industrie und ausgewählte Parameter ... 240
Tabelle 139: Belästigung durch Autobahnlärm gruppiert ... 242
Tabelle 140: Belästigung durch Autobahnlärm und ausgewählte Parameter (gruppiert) ... 243
Tabelle 141: Mittelwerte der Belästigung im Schlaf nach Quelle ... 245
Tabelle 142: Belästigung im Schlaf nach Lärmquelle und Katastralgemeinde im Mittelwertvergleich ... 248
Tabelle 143: Belästigung im Schlaf durch Lärm gesamt gruppiert ... 251
Tabelle 144: Belästigung im Schlaf durch Lärm insgesamt und Nachtschlaf im Mittelwertvergleich ... 252
Tabelle 145: Belästigung im Schlaf durch Lärm insgesamt und ausgewählte Parameter ... 254
Tabelle 146: Belästigung im Schlaf durch Straßenverkehrslärm gruppiert ... 256
Tabelle 147: Belästigung im Schlaf durch Straßenverkehrslärm und ausgewählte Parameter ... 259
Tabelle 148: Belästigung im Schlaf durch Lärm von Passanten und Gaststätten gruppiert ... 261
Tabelle 149: Belästigung im Schlaf durch Passanten und Gaststätten und ausgewählte Parameter ... 262
Tabelle 150: Belästigung im Schlaf durch Nachbarschaftslärm gruppiert ... 264
Tabelle 151: Belästigung im Schlaf durch Nachbarschaftslärm und ausgewählte Parameter ... 265
Tabelle 152: Belästigung im Schlaf durch Fluglärm gruppiert ... 267
Tabelle 153: Belästigung im Schlaf durch Flugverkehrslärm und höchster erreichter Bildungsabschluss im Mittelwertvergleich ... 269
Tabelle 154: Belästigung im Schlaf durch Flugverkehrslärm und ausgewählte Parameter ... 269
Tabelle 155: Belästigung im Schlaf durch Lärm von Baustellen gruppiert ... 271
Tabelle 156: Belästigung im Schlaf durch Baustellenlärm und ausgewählte Parameter ... 272
Tabelle 157: Belästigung im Schlaf durch Schienenverkehrslärm gruppiert ... 274
Tabelle 158: Belästigung im Schlaf durch Schienenverkehrslärm und ausgewählte Parameter ... 275
Tabelle 159: Belästigung im Schlaf durch Erschütterungen gruppiert ... 277
Tabelle 160: Belästigung im Schlaf durch Erschütterungen und ausgewählte Parameter ... 278
Tabelle 161: Belästigung im Schlaf durch Lärm von Gewerbe und Industrie gruppiert ... 280
Tabelle 162: Belästigung im Schlaf durch Lärm von Gewerbe / Industrie und ausgewählte Parameter ... 281
Tabelle 163: Belästigung im Schlaf durch Autobahnlärm gruppiert ... 283
Tabelle 164: Belästigung im Schlaf durch Autobahnlärm und ausgewählte Parameter ... 284
Tabelle 165: Lärmcopingreaktionen im Mittelwertvergleich (absteigend sortiert) ... 287

Tabelle 166: Emotionales Lärmcoping und Lärmbelastung nach Quelle (gruppiert) 290
Tabelle 167: Emotionales Lärmcoping und ausgewählte Parameter (gruppiert) 291
Tabelle 168: Politisches Lärmcoping und ausgewählte Parameter (gruppiert) 293
Tabelle 169: Summenscore Lärmcoping und ausgewählte Parameter (gruppiert) 294
Tabelle 170: Summenscore Lärmcoping und Lärmbelästigung (gruppiert) 296
Tabelle 171: Empfundene Zu- und Abnahme der Lärmbelastung und ausgewählte Parameter 298
Tabelle 172: Empfundene Zu- und Abnahme der Lärmbelastung und Belästigung nach Quelle 300
Tabelle 173: Einschätzung der negativen Wirkung von Lärm auf die Gesundheit gruppiert 302
Tabelle 174: Mittelwert der Einschätzung der negativen Wirkung von Lärm auf die Gesundheit 302
Tabelle 175: Einschätzung der negativen Wirkung von Lärm auf die Gesundheit und ausgewählte Parameter (gruppiert) 303
Tabelle 176: Bekanntheit der Umgebungslärmkarten und ausgewählte Parameter (gruppiert) 305
Tabelle 177: Einschätzung des Lärmniveaus im Städtevergleich gruppiert 306
Tabelle 178: Mittelwert der Einschätzung des Lärmniveaus im Städtevergleich 306
Tabelle 179: Einschätzung des Lärmniveaus im Städtevergleich und ausgewählte Parameter 308
Tabelle 180: Einschätzung des eigenen Lärmbeitrags gruppiert 310
Tabelle 181: Mittelwert Einschätzung des eigenen Lärmbeitrags 310
Tabelle 182: Einschätzung des eigenen Lärmbeitrags und demographische Parameter (gruppiert) 311
Tabelle 183: Einschätzung der störendsten Lärmquelle in Innsbruck und demographische Parameter 313
Tabelle 184: Einschätzung der störendsten Lärmquelle in Innsbruck und ausgewählte Parameter 315
Tabelle 185: Einschätzung der störendsten Lärmquelle in Innsbruck und Lärmbelastung nach Quelle 316
Tabelle 186: Einschätzung der störendsten Lärmquelle in Innsbruck u. Lärmbelästigung nach Quelle 318
Tabelle 187: Sinnhaftigkeit (weiterer) Maßnahmen zur Lärmreduktion in Innsbruck (ohne w.n. / k.A.) 319
Tabelle 188: Sinnhaftigkeit von Maßnahmen zur Lärmreduktion und ausgewählte Parameter 321
Tabelle 189: Sinnhaftigkeit von Maßnahmen zur Lärmreduktion und Lärmbelastung nach Quelle 321
Tabelle 190: Sinnhaftigkeit von Maßnahmen zur Lärmreduktion und Lärmbelästigung nach Quelle 323
Tabelle 191: Sinnhaftigkeit Einzelmaßnahmen zur Lärmreduktion in Innsbruck 325

Abbildungsverzeichnis

Abbildung 1: Katastralgemeinden Innsbruck 35
Abbildung 2: Einwohnerschaft Innsbrucks mit Hauptwohnsitz nach Katastralgemeinden (2016) 36
Abbildung 3: Demographische Strukturen in Innsbruck auf einen Blick 36
Abbildung 4: Alterspyramide der Bevölkerung von Innsbruck nach Geschlecht 2016 37
Abbildung 5: Bevölkerung von Innsbruck nach höchstem erreichten Schulabschluss (2014) 37
Abbildung 6: Überblick der Verkehrslärmbelastung nach Quelle 48
Abbildung 7: Prozentuelle Verteilung der Belastung der Gebäude und Wohnungen in Innsbruck nach Quelle 49
Abbildung 8: Rasterlärmkarte 24h-Fluglärm L_{den} in Innsbruck gruppiert nach Belastungskategorien 50
Abbildung 9: Rasterlärmkarte 24h-Schienenverkehrslärm L_{den} in Innsbruck gruppiert nach Belastungskategorien 51
Abbildung 10: Rasterlärmkarte 24h-Straßenverkehrslärm L_{den} in Innsbruck gruppiert nach Belastungskategorien 52
Abbildung 11: Übersicht der ausgewerteten Katastralgemeinden in Innsbruck 53
Abbildung 12: Mittelwertvergleich der 24h-Lärmbelastung L_{den} nach Quelle in dB 54
Abbildung 13: Vergleich gruppierte Häufigkeit der 24h-Lärmbelastung L_{den} nach Quelle in Prozent 55
Abbildung 14: Mittelwertvergleich der 24h-Lärmbelastung L_{den} bewohnter Gebäude der Innsbrucker Katastralgemeinden nach Quelle in dB 55
Abbildung 15: Stark verkehrslärmbelastete Adresspunkte in Innsbruck nach Quelle und Katastralgemeinde in Prozent 57
Abbildung 16: Mittelwertvergleich der nächtlichen Lärmbelastung L_n nach Quelle in dB 59
Abbildung 17: Vergleich gruppierte Häufigkeit der nächtlichen Lärmbelastung L_n nach Quelle in Prozent 61
Abbildung 18: Mittelwertvergleich der nächtlichen Lärmbelastung L_n bewohnter Gebäude der Innsbrucker Katastralgemeinden nach Quelle in dB 61
Abbildung 19: In der Nacht stark verkehrslärmbelastete Adresspunkte in Innsbruck nach Quelle und Katastralgemeinde in Prozent 63
Abbildung 20: Rasterlärmkarte für den Autobahnverkehr als L_{den} in 4 m Höhe 67
Abbildung 21: Rasterlärmkarte für den Straßenverkehr als L_{den} in 4 m Höhe 68
Abbildung 22: Rasterlärmkarte für den Flugverkehr als L_{den} in 4 m Höhe 70
Abbildung 23: Rasterlärmkarte für den Flugverkehr als $L_{A,ES}$ in 4 m Höhe 71
Abbildung 24: Rasterlärmkarte für den Schienenverkehr als L_{den} in 4 m Höhe 72
Abbildung 25: Rasterlärmkarte für den Schienenverkehr als $L_{A,ES}$ in 4 m Höhe 73
Abbildung 26: Rasterlärmkarte für den Gesamtlärm als effektbezogener Substitutionspegel $L_{A,ES}$ in 4 m Höhe 75
Abbildung 27: Hotsptanalyse in 200 m Rasterzellen auf Basis der Lärmkennziffer 76
Abbildung 28: Ausweisung „ruhiger Gebiete“ mit einem effektbezogener Substitutionspegel $L_{A,ES}$ in 4 m Höhe kleiner 55 dB 78
Abbildung 29: Angepasster Befragungsschlüssel Katastralgemeinden Innsbruck 85
Abbildung 30: Bevölkerungspyramide der befragten Personen nach Altersgruppe und Geschlecht 93
Abbildung 31: Bildungsschichten kategorisiert nach höchstem erreichten Schulabschluss 94
Abbildung 32: Höchster bisher erreichter Bildungsabschluss nach Geschlecht 95
Abbildung 33: Höchster Bildungsabschluss innerhalb kategorisierter Altersgruppen 95
Abbildung 34: Berufliche Stellung der Befragten in Prozent 96

Abbildung 35: Wohndauer in derselben Wohnung nach Alter 98
Abbildung 36: Eigentumsverhältnisse der Wohnung / des Hauses 99
Abbildung 37: Haushaltsgröße in Prozent 99
Abbildung 38: Schutz vor Verkehrslärm durch bauliche Abschirmungen in Prozent 101
Abbildung 39: Vorrangiges Schutzziel der Lärmschutzfenster nach Lärmquelle in Prozent 102
Abbildung 40: Auspendleranteil gesamt in Prozent 103
Abbildung 41: Vorrangiges Verkehrsmittel auf dem Weg zum Arbeits- oder Ausbildungsplatz in Prozent 104
Abbildung 42: Häufigkeit der Nutzung von Kraftfahrzeugen als Fahrerin / Fahrer in Prozent 104
Abbildung 43: Häufigkeit der Nutzung öffentlicher Verkehrsmittel in Innsbuck in Prozent 106
Abbildung 44: Häufigkeit der Nutzung öffentl. Verkehrsmittel außerhalb von Innsbruck in % 107
Abbildung 45: Häufigkeit der Nutzung von Fahrrädern in Prozent 108
Abbildung 46: Häufigkeit fußläufiger Wege in Prozent 110
Abbildung 47: Häufigkeit der Nutzung von Flugzeugen in Prozent 111
Abbildung 48: Mittelwertvergleich der Lebensbedingungen 113
Abbildung 49: Beurteilung der Wohnbedingungen in Prozent 114
Abbildung 50: Beurteilung der Nachbarschaftsbeziehungen in Prozent 114
Abbildung 51: Beurteilung der Sicherheit in Prozent 115
Abbildung 52: Beurteilung von Landschaft, Natur, Parks und Grünflächen in Prozent 116
Abbildung 53: Beurteilung der Luftqualität in Prozent 117
Abbildung 54: Beurteilung der öffentlichen Verkehrsmittel in Prozent 117
Abbildung 55: Beurteilung der Bedingungen für das Aufwachsen von Kinder in Prozent 118
Abbildung 56: Beurteilung der Verbundenheit zur Stadt Innsbruck in Prozent 119
Abbildung 57: Summenscore Lebensbedingungen und Katastralgemeinde im Mittelwertvergleich (aufsteigend sortiert) 120
Abbildung 58: Lebensbedingungen und Alter (gruppiert) im Mittelwertvergleich 122
Abbildung 59: Lebensbedingungen und höchster Schulabschluss (gruppiert) im Mittelwertvergleich 122
Abbildung 60: Lebensbedingungen und Gesundheitszustand (gruppiert) im Mittelwertvergleich 123
Abbildung 61: Lebensbedingungen und persönl. Lebensqualität (gruppiert) im Mittelwertvergleich 124
Abbildung 62: Lebensbedingungen und 24h-Straßenverkehrslärmbelastung L_{den} (gruppiert) im Mittelwertvergleich 125
Abbildung 63: Lebensbedingungen und Lärmexpositionsempfinden insgesamt (gruppiert) im Mittelwertvergleich 126
Abbildung 64: Lebensbedingungen und Fluglärmexpositionsempfinden (gruppiert) im Mittelwertvergleich 127
Abbildung 65: Lebensbedingungen und Schienenverkehrslärmexpositionsempfinden (gruppiert) im Mittelwertvergleich 128
Abbildung 66: Lebensbedingungen und Lärmbelästigung insgesamt (gruppiert) im Mittelwertvergleich 129
Abbildung 67: Lebensbedingungen und Straßenlärmbelästigung (gruppiert) im Mittelwertvergleich 129
Abbildung 68: Lebensbedingungen und Fluglärmbelästigung (gruppiert) im Mittelwertvergleich 130
Abbildung 69: Lebensbedingungen und Schienenlärmbelästigung (gruppiert) im Mittelwertvergleich 130
Abbildung 70: Lebensbedingungen nach Nachtschlaf bei offenem oder gekippten Fenster (gruppiert) im Mittelwertvergleich 133
Abbildung 71: Zufriedenheit mit der eigenen Wohnsituation in Prozent 134
Abbildung 72: Zufriedenheit mit der eigenen Wohnsituation und Katastralgemeinde im Mittelwertvergleich (aufsteigend sortiert) 135
Abbildung 73: Anteilige Zufriedenheit mit der Wohnsituation nach Lärmempfindlichkeit (gruppiert) 137
Abbildung 74: Zufriedenheit mit der Wohnsituation und 24h-Straßenlärmbelastung L_{den} (gruppiert) 137

Abbildung 75: Zufriedenheit mit der Wohnsituation und 24h-Straßenverkehrslärmbelastung L_{den} in 10-dB Schritten im Mittelwertvergleich ... 138
Abbildung 76: Zufriedenheit mit der Wohnsituation und 24h-Fluglärmbelastung L_{den} (gruppiert) ... 138
Abbildung 77: Zufriedenheit mit der Wohnsituation und 24h-Schienenlärmbelastung L_{den} (gruppiert) ... 139
Abbildung 78: Zufriedenheit mit der Wohnsituation und nächtliche Straßenverkehrslärmbelastung L_n in 10-dB Schritten im Mittelwertvergleich ... 140
Abbildung 79: Zufriedenheit mit der Wohnsituation und verkehrsverursachtes Lärmexpositionsempfinden im Mittelwertvergleich ... 141
Abbildung 80: Zufriedenheit mit der Wohnsituation und nichtverkehrsverursachtes Lärmexpositionsempfinden im Mittelwertvergleich ... 142
Abbildung 81: Zufriedenheit mit der Wohnsituation und Lärmbelästigung gesamt im Mittelwertvergleich ... 142
Abbildung 82: Zufriedenheit mit der Wohnsituation und Lärmbelästigung nach Quelle (gruppiert) im Mittelwertvergleich (aufsteigend sortiert nach Pearsons Rho) ... 143
Abbildung 83: Zufriedenheit mit der Wohnsituation und Lärmbelästigung in der Nacht nach Quelle (gruppiert) im Mittelwertvergleich (aufsteigend sortiert nach Pearsons Rho) ... 144
Abbildung 84: Einschätzung des eigenen Gesundheitszustandes in Prozent ... 145
Abbildung 85: Einschätzung des eigenen Gesundheitszustandes und höchster erreichter Schulabschluss nach Altersgruppen (gruppiert) im Mittelwertvergleich ... 146
Abbildung 86: Gesundheitszustand und Lärmbelästigung nach Quelle (gruppiert) im Mittelwertvergleich (aufsteigend sortiert nach Extremwertdifferenz) ... 148
Abbildung 87: Einschätzung der persönlichen Lebensqualität in Prozent ... 150
Abbildung 88: Einschätzung der Lebensqualität und höchster erreichter Schulabschluss (gruppiert) ... 151
Abbildung 89: Einschätzung der eigenen Lebensqualität und nächtliche Straßenverkehrslärmbelastung L_n in 10dB-Schritten im Mittelwertvergleich ... 152
Abbildung 90: Lebensqualität und Expositionsempfinden Lärm insgesamt im Mittelwertvergleich ... 153
Abbildung 91: Lebensqualität und Belästigung durch Lärm insgesamt im Mittelwertvergleich ... 154
Abbildung 92: Lebensqualität und Lärmbelästigung nach Quelle (gruppiert) im Mittelwertvergleich (aufsteigend sortiert nach Extremwertdifferenz) ... 155
Abbildung 93: Lebensqualität und Lärmbelästigung im Schlaf nach Quelle (gruppiert) im Mittelwertvergleich (aufsteigend sortiert nach Extremwertdifferenz) ... 155
Abbildung 94: Einschätzung der eigenen Lärmempfindlichkeit in Prozent ... 156
Abbildung 95: Lärmempfindlichkeit und Belästigung durch Lärm insgesamt im Mittelwertvergleich ... 160
Abbildung 96: Lärmempfindlichkeit und Belästigung durch Lärm nach Quelle (gruppiert) im Mittelwertvergleich (aufsteigend sortiert nach Extremwertdifferenz) ... 161
Abbildung 97: Lärmempfindlichkeit und Belästigung durch Verkehrslärm nach Quelle (gruppiert) im Mittelwertvergleich ... 162
Abbildung 98: Lärmempfindlichkeit und Belästigung durch Lärm im Schlaf nach Quelle (gruppiert) im Mittelwertvergleich (aufsteigend sortiert nach Extremwertdifferenz) ... 162
Abbildung 99: Ausmaß des Einflusses von Wind- und Wetterverhältnissen auf die Lärmbelastung ... 163
Abbildung 100: Nachtschlaf bei geöffnetem oder gekipptem Fenster in Prozent ... 166
Abbildung 101: Nachtschlaf bei geöffnetem oder gekipptem Fenster und nächtliche Straßenverkehrslärmbelastung L_n in 10-db Schritten im Mittelwertvergleich ... 168
Abbildung 102: Mittelwertvergleich der empfundenen Exposition nach Quelle ... 170
Abbildung 103: Empfundene Exposition nach Quelle kategorisiert in 3 Expositionsgruppen in Prozent ... 171
Abbildung 104: Extremgruppenvergleich *highly exposed* nach Quelle in Prozent ... 171

Abbildung 105: Empfundene Lärmexposition und Verkehrslärmbelastung nach Quelle 24h- (L_{den}) und Nachtzeitraum (L_n) gruppiert im Mittelwertvergleich (aufsteigend sortiert) 172
Abbildung 106: Empfundene Lärmexposition und Lärmbelastung durch Straßen- und Flugverkehr im 24h- (L_{den}) und Nachtzeitraum (L_n) in 10dB-Schritten im Mittelwertvergleich (absteigend sortiert) 172
Abbildung 107: Empfundene Lärmexposition und Schienenverkehrslärmbelastung im 24h- (L_{den}) und Nachtzeitraum (L_n) in 10dB-Schritten im Mittelwertvergleich 173
Abbildung 108: Empfundene Lärmexposition insgesamt in Prozent 173
Abbildung 109: Empfundene Lärmexposition insgesamt und Katastralgemeinden im Mittelwertvergleich 174
Abbildung 110: Empfundene Lärmexposition insgesamt und Belästigung durch Lärm insgesamt im Mittelwertvergleich 176
Abbildung 111: Empfundene Flugverkehrslärmexposition in Prozent 177
Abbildung 112: Empfundene Flugverkehrslärmexposition und 24h-Fluglärmbelastung L_{den} in 10dB-Schritten im Mittelwertvergleich 178
Abbildung 113: Empfundene Flugverkehrslärmexposition und nächtliche Fluglärmbelastung L_n in 10dB-Schritten im Mittelwertvergleich 178
Abbildung 114: Empfundene Fluglärmexposition und Katastralgemeinden im Mittelwertvergleich 178
Abbildung 115: Empfundene Straßenverkehrslärmexposition in Prozent 180
Abbildung 116: Empfundene Straßenverkehrslärmexposition und 24h-Straßenverkehrslärmbelastung L_{den} in 10dB-Schritten im Mittelwertvergleich 181
Abbildung 117: Empfundene Straßenverkehrslärmexposition und nächtliche Straßenverkehrslärmbelastung L_n in 10dB-Schritten im Mittelwertvergleich 182
Abbildung 118: Empfundene Straßenlärmexposition und Katastralgemeinden im Mittelwertvergleich 182
Abbildung 119: Empfundene Straßenverkehrslärmexposition und Belästigung durch Straßenverkehrslärm im Mittelwertvergleich 183
Abbildung 120: Empfundene Baustellenlärmexposition in Prozent 184
Abbildung 121: Empfundene Baustellenlärmexposition und Katastralgemeinden im Mittelwertvergleich 185
Abbildung 122: Empfundene Nachbarschaftslärmexposition in Prozent 187
Abbildung 123: Empfundene Nachbarschaftslärmexposition und Katastralgemeinden im Mittelwertvergleich 188
Abbildung 124: Empfundene Nachbarschaftslärmexposition und Güte der Nachbarschaftsbeziehungen im Mittelwertvergleich 190
Abbildung 125: Empfundene Lärmexposition durch Passanten und Gaststätten in Prozent 190
Abbildung 126: Empfundene Lärmexposition durch Passanten und Gaststätten und Katastralgemeinden im Mittelwertvergleich 191
Abbildung 127: Empfundene Exposition gegenüber Erschütterungen in Prozent 193
Abbildung 128: Empfundene Erschütterungsexposition und Katastralgemeinden im Mittelwertvergleich........ 194
Abbildung 129: Empfundene Schienenverkehrslärmexposition in Prozent 196
Abbildung 130: Empfundene Schienenverkehrslärmexposition und 24h-Schienenverkehrslärmbelastung L_{den} in 10dB-Schritten im Mittelwertvergleich 196
Abbildung 131: Empfundene Schienenverkehrslärmexposition und nächtliche Schienenverkehrslärmbelastung L_n in 10dB-Schritten im Mittelwertvergleich 197
Abbildung 132: Empfundene Schienenverkehrslärmexposition und Katastralgemeinden im Mittelwertvergleich 197
Abbildung 133: Empfundene Autobahnlärmexposition in Prozent 199
Abbildung 134: Empfundene Autobahnlärmexposition und Katastralgemeinden im Mittelwertvergleich 200
Abbildung 135: Empfundene Lärmexposition durch Gewerbe und Industrie in Prozent 201

Abbildung 136: Empfundene Lärmexposition durch Gewerbe und Industrie und Katastralgemeinden im Mittelwertvergleich 202
Abbildung 137: Mittelwertvergleich der Belästigung nach Quelle 204
Abbildung 138: Belästigung nach Quelle kategorisiert in 3 Expositionsgruppen in Prozent 205
Abbildung 139: Extremgruppenvergleich *highly annoyed* nach Quelle in Prozent 205
Abbildung 140: Belästigung und Verkehrslärmbelastung nach Quelle 24h-Pegel (L_{den}) gruppiert im Mittelwertvergleich 206
Abbildung 141: Belästigung und Verkehrslärmbelastung nach Quelle 24h-Pegel (L_{den}) in 10dB-Schritten im Mittelwertvergleich 206
Abbildung 142: Belästigung nach Lärmquelle und Katastralgemeinde im Mittelwertvergleich 207
Abbildung 143: Belästigung nach Verkehrslärmquelle und Katastralgemeinde im Mittelwertvergleich 209
Abbildung 144: Belästigung nach nicht verkehrsverursachten Lärmquellen und Katastralgemeinde im Mittelwertvergleich 209
Abbildung 145: Belästigung durch Lärm insgesamt in Prozent 210
Abbildung 146: Belästigung durch Lärm insgesamt und Katastralgemeinden im Mittelwertvergleich 211
Abbildung 147: Belästigung durch Lärm insgesamt und baulicher Schutz vor Verkehrslärm im Extremgruppenvergleich in Prozent 211
Abbildung 148: Zufriedenheit mit der eigenen Wohnsituation und Belästigung durch Lärm insgesamt im Mittelwertvergleich 212
Abbildung 149: Lärmempfindlichkeit und Belästigung durch Lärm insgesamt im Mittelwertvergleich 213
Abbildung 150: Belästigung durch Flugverkehrslärm in Prozent 215
Abbildung 151: Fluglärmbelästigung, –Expositionsempfinden und 24h-Fluglärmbelastung L_{den} in 10dB-Schritten im Mittelwertvergleich 216
Abbildung 152: Fluglärmbelästigung und Katastralgemeinden im Mittelwertvergleich 216
Abbildung 153: Wohnbedingungen und Belästigung durch Fluglärm im Extremgruppenvergleich 217
Abbildung 154: Lärmempfindlichkeit und Belästigung durch Fluglärm im Mittelwertvergleich 217
Abbildung 155: Belästigung durch Strassenverkehrslärm in Prozent 219
Abbildung 156: Straßenlärmbelästigung und –Expositionsempfinden und 24h-Straßenlärmbelastung L_{den} in 10dB-Schritten im Mittelwertvergleich 220
Abbildung 157: Straßenlärmbelästigung und Katastralgemeinden im Mittelwertvergleich 220
Abbildung 158: Belästigung durch Straßenverkehrslärm und baulicher Schutz vor Verkehrslärm im Extremgruppenvergleich in Prozent 221
Abbildung 159: Lebensbedingungen (Summenscore) und Belästigung durch Straßenverkehrslärm im Mittelwertvergleich 222
Abbildung 160: Belästigung durch Baustellenlärm in Prozent 224
Abbildung 161: Belästigung durch Baustellenlärm und Katastralgemeinden im Mittelwertvergleich 224
Abbildung 162: Belästigung durch Nachbarschaftslärm in Prozent 226
Abbildung 163: Nachbarschaftslärmbelästigung und Katastralgemeinden im Mittelwertvergleich 227
Abbildung 164: Zufriedenheit mit der eigenen Wohnsituation und Belästigung durch Nachbarschaftslärm im Mittelwertvergleich 228
Abbildung 165: Belästigung durch Lärm von Passanten und Gaststätten in Prozent 229
Abbildung 166: Lärmbelästigung Passanten / Gaststätten und Katastralgemeinden im Mittelwertvergleich 230
Abbildung 167: Belästigung durch Schienenverkehrslärm in Prozent 232
Abbildung 168: Schienenlärmbelästigung und –Expositionsempfinden und 24h-Schienenlärmbelastung L_{den} in 10dB-Schritten im Mittelwertvergleich 233
Abbildung 169: Schienenverkehrslärmbelästigung und Katastralgemeinden im Mittelwertvergleich 233

Abbildung 170: Güte der Wohnbedingungen und Belästigung durch Schienenverkehrslärm nach 24h-Schienenlärmbelastung L_{den} in 10dB-Schritten im Mittelwertvergleich.................................. 234
Abbildung 171: Belästigung durch Erschütterungen in Prozent 236
Abbildung 172: Belästigung durch Erschütterungen und Katastralgemeinden im Mittelwertvergleich 236
Abbildung 173: Belästigung durch Lärm aus Gewerbe und Industrie in Prozent.. 238
Abbildung 174: Lärmbelästigung Gewerbe und Industrie und Katastralgemeinden im Mittelwertvergleich...... 239
Abbildung 175: Belästigung durch Autobahnlärm in Prozent 241
Abbildung 176: Autobahnlärmbelästigung und –Expositionsempfinden und 24h-Autobahnlärmbelastung L_{den} in 10dB-Schritten im Mittelwertvergleich... 242
Abbildung 177: Belästigung durch Autobahnlärm und Katastralgemeinden im Mittelwertvergleich 243
Abbildung 178: Mittelwertvergleich der Belästigung im Schlaf nach Quelle 245
Abbildung 179: Belästigung im Schlaf nach Quelle kategorisiert in 3 Expositionsgruppen in Prozent 246
Abbildung 180: Extremgruppenvergleich *highly annoyed sleep* nach Quelle in Prozent................................ 246
Abbildung 181: Belästigung im Schlaf und Verkehrslärmbelastung nach Quelle Nachtlärmpegel (L_n) gruppiert im Mittelwertvergleich.. 247
Abbildung 182: Belästigung im Schlaf und Verkehrslärmbelastung nach Quelle Nachtlärmpegel (L_n) in 10dB-Schritten im Mittelwertvergleich.. 247
Abbildung 183: Belästigung im Schlaf nach Lärmquelle und Katastralgemeinde im Mittelwertvergleich 248
Abbildung 184: Belästigung im Schlaf nach Verkehrslärmquelle und Katastralgemeinde im Mittelwertvergleich.. 249
Abbildung 185: Belästigung im Schlaf nach nicht verkehrsverursachten Lärmquellen und Katastralgemeinde im Mittelwertvergleich.. 250
Abbildung 186: Belästigung durch Lärm im Schlaf insgesamt in Prozent 251
Abbildung 187: Belästigung im Schlaf durch Lärm insgesamt und Katastralgemeinden im Mittelwertvergleich251
Abbildung 188: Belästigung im Schlaf durch Lärm insgesamt nach Geschlecht (anteilig) 252
Abbildung 189: Belästigung im Schlaf durch Lärm insgesamt und baulicher Schutz vor Verkehrslärm im Extremgruppenvergleich in Prozent.. 253
Abbildung 190: Güte der eigenen Wohnbedingunegn und Belästigung im Schlaf durch Lärm insgesamt im Mittelwertvergleich.. 253
Abbildung 191: Lebensqualität und Belästigung im Schlaf durch Lärm insgesamt im Mittelwertvergleich....... 254
Abbildung 192: Belästigung durch Straßenverkehrslärm im Schlaf in Prozent... 256
Abbildung 193: Straßenlärmbelästigung im Schlaf und nächtliche Straßenlärmbelastung L_n in 10dB-Schritten im Mittelwertvergleich.. 257
Abbildung 194: Straßenlärmbelästigung im Schlaf und Katastralgemeinden im Mittelwertvergleich 257
Abbildung 195: Belästigung im Schlaf durch Straßenverkehrslärm und baulicher Schutz vor Verkehrslärm im Extremgruppenvergleich in Prozent.. 258
Abbildung 196: Einschätzung des Lärmniveaus in Innsbruck im Vergleich zu anderen Städten Öster-reichs und Belästigung im Schlaf durch Straßenverkehrslärm im Mittelwertvergleich 258
Abbildung 197: Einschätzung der Sicherheit und Belästigung im Schlaf durch Straßenverkehrslärm nach nächtlicher Straßenverkehrslärmbelastung L_n in 10dB-Schritten im Mittelwertvergleich......... 259
Abbildung 198: Belästigung durch Lärm von Passanten und Gaststätten im Schlaf in Prozent 261
Abbildung 199: Lärmbelästigung im Schlaf durch Passanten und Gaststätten und Katastralgemeinden im Mittelwertvergleich.. 261
Abbildung 200: Belästigung im Schlaf durch Lärm von Passanten und Gaststätten und Verbundenheit zur Stadt Innsbruck im Extremgruppenvergleich in Prozent 262
Abbildung 201: Belästigung durch Nachbarschaftslärm im Schlaf in Prozent 264

Abbildung 202: Nachbarschaftslärmbelästigung im Schlaf und Katastralgemeinden im Mittelwertvergleich.... 265
Abbildung 203: Zufriedenheit mit der eigenen Wohnsituation insgesamt und Belästigung im Schlaf durch Nachbarschaftslärm im Mittelwertvergleich ... 265
Abbildung 204: Belästigung im Schlaf durch Flugverkehrslärm in Prozent ... 267
Abbildung 205: Fluglärmbelästigung im Schlaf und nächtliche Fluglärmbelastung L_n in 10dB-Schritten im Mittelwertvergleich ... 268
Abbildung 206: Fluglärmbelästigung im Schlaf und Katastralgemeinden im Mittelwertvergleich ... 268
Abbildung 207: Belästigung durch Baustellenlärm im Schlaf in Prozent ... 271
Abbildung 208: Belästigung im Schlaf durch Baulärm und Katastralgemeinden im Mittelwertvergleich ... 271
Abbildung 209: Belästigung durch Schienenverkehrslärm im Schlaf in Prozent ... 273
Abbildung 210: Schienenlärmbelästigung im Schlaf und nächtliche Schienenlärmbelastung L_n in 10dB-Schritten im Mittelwertvergleich ... 274
Abbildung 211: Schienenlärmbelästigung im Schlaf und Katastralgemeinden im Mittelwertvergleich ... 275
Abbildung 212: Belästigung durch Erschütterungen im Schlaf in Prozent ... 277
Abbildung 213: Belästigung im Schlaf durch Erschütterungen und Katastralgemeinden im Mittelwertvergleich ... 277
Abbildung 214: Belästigung im Schlaf durch Lärm von Gewerbe und Industrie in Prozent ... 280
Abbildung 215: Belästigung im Schlaf durch Lärm von Gewerbe und Industrie und Katastralgemeinden im Mittelwertvergleich ... 280
Abbildung 216: Belästigung durch Autobahnlärm im Schlaf in Prozent ... 283
Abbildung 217: Autobahnlärmbelästigung und und nächtliche Autobahnlärmbelastung L_n in 10dB-Schritten im Mittelwertvergleich ... 283
Abbildung 218: Belästigung im Schlaf durch Autobahnlärm und Katastralgemeinden im Mittelwertvergleich .. 284
Abbildung 219: Lärmcopingreaktionen im Vergleich in Prozent (nach Häufigkeit absteigend sortiert) ... 287
Abbildung 220: Summenscore Lärmcoping in Prozent ... 288
Abbildung 221: Instrumentelles Lärmcoping in Prozent ... 288
Abbildung 222: Instrumentelles Lärmcoping und baulicher Schutz vor Verkehrslärm (gruppiert) ... 289
Abbildung 223: Emotionales Lärmcoping in Prozent ... 289
Abbildung 224: Ärger über Verkehrslärm und 24h-Lärmbelastung L_{den} nach Quelle in 10dB-Schritten im Mittelwertvergleich ... 290
Abbildung 225: Emotionales Lärmcoping und Lärmempfindlichkeit (gruppiert) ... 291
Abbildung 226: Politisches Lärmcoping in Prozent ... 292
Abbildung 227: Summenscore Lärmcoping und 24h-Lärmbelastung L_{den} nach Quelle in 10dB-Schritten im Mittelwertvergleich ... 294
Abbildung 228: Summenscore Lärmcoping und Belästigung durch Lärm gesamt im Mittelwertvergleich ... 295
Abbildung 229: Summenscore Lärmcoping und Belästigung durch Lärm gesamt im Schlaf im Mittelwertvergleich ... 297
Abbildung 230: Empfundene Zu- und Abnahme der Lärmbelastung in Prozent ... 298
Abbildung 231: Empfundene Zunahme der Lärmbelastung und Belästigung durch Lärm insgesamt im Mittelwertvergleich ... 300
Abbildung 232: Einschätzung der negativen Wirkung von Lärm auf die Gesundheit in Prozent ... 302
Abbildung 233: Einschätzung der negativen Wirkung von Lärm auf die Gesundheit und höchster erreichter Schulabschluss (gruppiert, ohne Antwortkategorie w.n. / k.A.) ... 303
Abbildung 234: Einschätzung der negativen Wirkung von Lärm auf die Gesundheit und Notwendigkeit (weiterer Maßnahmen) zur Lärmreduktion (gruppiert, ohne Antwortkategorie w.n. / k.A.) ... 304
Abbildung 235: Bekanntheit der Umgebungslärmkarten in Prozent ... 305

Abbildung 236: Einschätzung des Lärmniveaus im Städtevergleich in Prozent 306
Abbildung 237: Einschätzung des Lärmniveaus und Lärmempfindlichkeit im Mittelwertvergleich.................. 307
Abbildung 238: Einschätzung des Lärmniveaus und Belästigung durch Lärm gesamt und Lärm gesamt im Schlaf (gruppiert).. 308
Abbildung 239: Einschätzung des eigenen Lärmbeitrags in Prozent.. 310
Abbildung 240: Einschätzung des eigenen Lärmbeitrags und vorrangiges Verkehrsmittel im Mittelwertvergleich... 312
Abbildung 241: Eigener Lärmbeitrag durchschnittlich bis groß und Mobilitätsverhalten im Extremgruppenvergleich inProzent.. 312
Abbildung 242: Einschätzung der störendsten Lärmquelle in Innsbruck in Prozent 313
Abbildung 243: Einschätzung der störendsten Lärmquelle und Bildungsschicht (gruppiert)........................... 314
Abbildung 244: Einschätzung der störendsten Lärmquelle und Lärmbelastung nach Quelle im Extremgruppenvergleich der Starkbelasteten 316
Abbildung 245: Einschätzung der störendsten Lärmquelle in Innsbruck und Lärmbelästigung nach Quelle im Extremgruppenvergleich der Starkbelästigten.. 317
Abbildung 246: Sinnhaftigkeit (weiterer) Maßnahmen zur Lärmreduktion in Prozent 319
Abbildung 247: Sinnhaftigkeit von Maßnahmen zur Lärmreduktion und Lärmniveau von Innsbruck im Städtevergleich 320
Abbildung 248: Sinnhaftigkeit von Maßnahmen zur Lärmreduktion und Lärmempfindlichkeit im Mittelwertvergleich... 320
Abbildung 249: Sinnhaftigkeit von Maßnahmen zur Lärmreduktion und Lärmbelästigung gesamt und im Schlaf (gruppiert) im Vergleich 322
Abbildung 250: Sinnhaftigkeit von Maßnahmen zur Lärmreduktion und Mittelwerte der Lärmbelästigung (alle Quellen) in Prozent.. 323
Abbildung 251: Befürwortung von Einzelmaßnahmen zur Lärmreduktion in Innsbruck in Prozent................. 324

ANHANG

Anhang 1: Deskriptive Statistik der Befragungen

Deskriptive Statistik	N	Minimum	Maximum	Summe	Mittelwert	Standard-fehler	Standardab-weichung
Gebäude-ID	1031	1124403 5	11264636	11607588143	11258572,40	113,164	3633,608
Anzahl Einwohner laut ZMR	1031	1	216	18268	17,72	,651	20,895
Anzahl Wohnungen laut ZMR	1031	1	105	10174	9,87	,371	11,919
Lärmquellensamplezugehörigkeit	1031	1,00	9,00	5171,00	5,0155	,08022	2,57579
Straßenlärm 24h	1031	24,90	72,00	56833,60	55,1247	,23189	7,44587
Straßenlärm Nacht	1031	15,30	63,30	46996,60	45,5835	,23237	7,46123
Autobahnlärm 24h	1031	30,30	65,70	45751,20	44,3756	,18708	6,00688
Autobahnlärm Nacht	1031	21,20	56,70	36289,00	35,1979	,18599	5,97197
Schienenlärm 24h	1031	20,70	69,00	46163,70	44,7757	,23758	7,62842
Schienenlärm Nacht	1031	10,60	62,70	39207,90	38,0290	,23513	7,54968
Fluglärm 24h	1031	30,50	66,40	45984,70	44,6020	,21712	6,97139
Fluglärm Nacht	1031	7,50	47,70	28082,20	27,2378	,23247	7,46429
Geschlecht	1031	1	2	1521	1,48	,016	,500
Geburtsjahr	1031	1920	1999	2030252	1969,21	,587	18,838
Alter	1031	18,00	97,00	49272,00	47,7905	,58673	18,83950
Altersgruppen	1031	1,00	3,00	1897,00	1,8400	,02546	,81764
Wohnhaft seit	1031	1,0	86,0	18142,0	17,597	,5594	17,9618
Hauptwohnsitz (Kontrollfrage)	1031	1	1	1031	1,00	0,000	0,000
Aufenthalt in Innsbruck mehr als 200 Tage im Jahr	1031	1	1	1031	1,00	0,000	0,000
Art des Hauses	1031	1	6	3923	3,81	,042	1,352
Eigentumsverhältnisse	1031	1	3	1801	1,75	,022	,704
Haushaltsgröße	1031	1,0	10,0	2648,0	2,568	,0412	1,3230
Anzahl Kinder unter 18 Jahren im selben Haushalt	1031	0	5	485	,47	,028	,902
Stockwerk Schlafzimmer	1031	-2,0	11,0	1562,0	1,515	,0428	1,3751
Schutz vor Verkehrslärm durch bauliche Abschirmungen (Gebäude, Mauern, Innen-höfe,...)	1031	1	5	1943	1,88	,031	1,002
Schutz vor Verkehrslärm durch geschützten Innenhof bzw. lärmabgewandte Seite	1031	1	3	1383	1,34	,016	,517
Nachtschlaf bei geöffneten oder gekippten Fenstern	1031	1	6	2105	2,04	,044	1,415
Lärmschutzfenster in den letzten 10 Jahren eingebaut	1031	1	3	2047	1,99	,018	,590
Schutz Lärmschutzfenster vor welcher Lärmquelle	1031	1,00	6,00	5585,00	5,4171	,04166	1,33753
Ausgesetztheit Lärm gesamt	1031	0	10	4443	4,31	,080	2,571
Ausgesetztheit Autobahnlärm	1031	0	10	683	,66	,053	1,711
Ausgesetztheit Strassenverkehrslärm	1031	0	10	3834	3,72	,091	2,921
Ausgesetztheit Schienenverkehrslärm	1031	0	10	1157	1,12	,069	2,208
Ausgesetztheit Flugverkehrslärm	1031	0	10	3987	3,87	,092	2,943
Ausgesetztheit Lärm von Gewerbe und Industrie	1031	0	10	671	,65	,054	1,735
Ausgesetztheit Lärm von Passanten u. Gaststätten	1031	0	10	1974	1,91	,082	2,622
Ausgesetztheit Lärm von Baustellen	1031	0	10	2473	2,40	,096	3,074

Ausgesetztheit Nachbarschaftslärm	1031	0	10	2147	2,08	,080	2,581
Ausgesetztheit Erschütterungen gesamt	1031	0	10	1422	1,38	,074	2,380
Ausmass Windeinfluss auf Lärm	1031	1	5	2302	2,23	,032	1,030
Güte Wohnbedingungen	1031	0	99	6703	6,50	,149	4,779
Güte Nachbarschaftsbeziehungen	1031	0	99	7946	7,71	,331	10,623
Güte Sicherheit	1031	0	99	6532	6,34	,121	3,876
Güte Landschaft, Natur, öffentliche Parks und Grünflächen	1031	0	99	8010	7,77	,230	7,375
Güte Luftqualität	1031	0	99	6329	6,14	,118	3,788
Güte öffentliche Verkehrsverbindungen	1031	0	99	9028	8,76	,312	10,020
Güte Bedingungen für Kinder	1031	0	99	9089	8,82	,421	13,507
Ausmaß Verbundenheit zur Stadt Innsbruck	1031	0	99	7818	7,58	,146	4,693
Ausmaß Zufriedenheit eigene Wohnsituation insgesamt	1031	0	99	8453	8,20	,110	3,527
Güte eigene Lebensqualität	1031	0	99	8825	8,56	,222	7,141
Ausmass eigene Lärmempfindlichkeit	1031	0	10	4606	4,47	,084	2,700
Güte eigener Gesundheitszustand	1031	1	99	2290	2,22	,136	4,383
Belästigung Lärm gesamt	1031	0	10	4013	3,89	,086	2,771
Belästigung Autobahnlärm	1031	0	10	615	,60	,053	1,702
Belästigung Strassenverkehrslärm	1031	0	10	3195	3,10	,090	2,893
Belästigung Schienenverkehrslärm	1031	0	10	1158	1,12	,067	2,151
Belästigung Fluglärm	1031	0	10	3262	3,16	,089	2,868
Belästigung Lärm aus Gewerbe u. Industrie	1031	0	10	649	,63	,052	1,672
Belästigung Lärm von Passanten und Gaststätten	1031	0	10	1764	1,71	,077	2,473
Belästigung Baustellenlärm	1031	0	99	2450	2,38	,132	4,254
Belästigung Nachbarschaftslärm	1031	0	10	1868	1,81	,077	2,457
Belästigung Erschütterungen gesamt	1031	0	10	1121	1,09	,068	2,171
Belästigung im Schlaf Lärm gesamt	1031	0	10	2225	2,16	,086	2,755
Belästigung im Schlaf Autobahnlärm	1031	0	10	295	,29	,039	1,238
Belästigung im Schlaf Straßenverkehrslärm	1031	0	10	1633	1,58	,078	2,519
Belästigung im Schlaf Schienenverkehrslärm	1031	0	10	637	,62	,054	1,725
Belästigung im Schlaf Fluglärm	1031	0	10	1121	1,09	,070	2,232
Belästigung im Schlaf Lärm von Gewerbe und Industrie	1031	0	10	356	,35	,042	1,348
Belästigung im Schlaf Lärm von Passanten und Gaststätten	1031	0	10	1401	1,36	,077	2,475
Belästigung im Schlaf Lärm von Baustellen	1031	0	99	853	,83	,112	3,609
Belästigung im Schlaf Nachbarschaftslärm	1031	0	10	1300	1,26	,073	2,337
Belästigung im Schlaf Erschütterungen gesamt	1031	0	10	542	,53	,047	1,523
Lärmcoping: Fenster im Sommer nachts geschlossen	1031	1	2	1590	1,54	,016	,498
Lärmcoping: Garten/Balkon nicht genutzt	1031	1	99	1941	1,88	,095	3,055
Lärmcoping: Andere Nutzung Wohnräume	1031	1	2	1903	1,85	,011	,361
Lärmcoping: Ärger über Verkehrslärm	1031	1	2	1494	1,45	,015	,498
Lärmcoping: Hilflosigkeit wegen Verkehrslärm	1031	1	2	1816	1,76	,013	,426
Lärmcoping: Gefühlte Zunahme Lärmbelastung	1031	1	2	1626	1,58	,015	,494
Lärmcoping: Gefühlte Abnahme Lärmbelastung	1031	1	2	2004	1,94	,007	,231
Lärmcoping: Gespräche mit Nachbarn über Lärm	1031	1	2	1708	1,66	,015	,475
Lärmcoping: Gespräche mit Behörden über Lärm	1031	1	2	1928	1,87	,010	,336

Derzeitige berufliche Stellung	1031	1	11	5630	5,46	,114	3,655
Höchster bisher erreichter Bildungsabschluss	1031	1	99	4057	3,94	,166	5,317
Arbeits- oder Ausbildungsplatz in Innsbruck	1031	1	99	1945	1,89	,099	3,167
Vorrangiges Verkehrsmittel auf dem Weg zum Arbeits- oder Ausbildungsplatz	1031	1	6	4160	4,03	,058	1,864
Häufigkeit Nutzung eines Autos als Fahrer/in	1031	1	5	2968	2,88	,048	1,540
Häufigkeit Nutzung öffentl. Verkehrsmittel innerhalb der Stadt	1031	1	99	3291	3,19	,103	3,322
Häufigkeit Nutzung der Eisenbahn oder von Autobussen außerhalb der Stadt	1031	1	99	4392	4,26	,096	3,096
Häufigkeit Nutzung eines Fahrrads	1031	1	5	3088	3,00	,052	1,669
Häufigkeit länger als 10 Minuten zu Fuß gehen	1031	1	5	1654	1,60	,029	,941
Häufigkeit Nutzung eines Flugzeuges	1031	1	5	3969	3,85	,023	,727
Einschätzung des Ausmaßes negativer Auswirkungen von Lärm auf die Gesundheit	1031	1	5	3226	3,13	,028	,909
Bekanntheitsgrad Umgebungslärmkarten	1031	1	2	1841	1,79	,013	,411
Lautheit Gesamtlärm in Innsbruck im Vergleich zu anderen Städten Österreichs	1031	1	99	4312	4,18	,347	11,157
Einschätzung eigener Beitrag zur Gesamtlärmbelastung in Innsbruck	1031	1	5	1974	1,91	,026	,850
Einschätzung störendste Lärmquelle insgesamt	1031	1	8	3527	3,42	,074	2,379
Notwendigkeit (weiterer) Maßnahmen zur Lärmreduktion in Innsbruck	1031	1	3	1519	1,47	,023	,733
Förderung lärmarmer Verkehrsmittel, Maschinen usw.	691	1	99	1752	2,54	,445	11,702
Strengere Vorschriften und Auflagen für Lärmverursacher	691	1	99	1547	2,24	,373	9,805
Förderung von Lärmschutzmaßnahmen	691	1	99	2062	2,98	,506	13,310
Strengere Kontrollen der Einhaltung von Lärmgrenzwerten	691	1	99	2492	3,61	,577	15,166
Bewusstseinsbildung zur Lärmvermeidung	691	1	99	1680	2,43	,423	11,107
Verkehrsbeschränkungen	691	0	99	2659	3,85	,576	15,131
Finanzielle Auflagen für Lärmverursacher	691	0	99	2143	3,10	,486	12,767
Schaffung, Schutz und Erhalt lärmarmer Bereiche	691	1	99	2226	3,22	,543	14,281
Andere Maßnahmen	691	1	99	4832	6,99	,857	22,524

Anhang 2: Häufigkeitsstatistik der Befragungen

Katastralgemeinde

		Häufigkeit	Gültige Prozente	Kumulierte Prozente
Gültig	Amras	60	5,8	5,8
	Arzl	102	9,9	15,7
	Hötting	243	23,6	39,3
	Igls	35	3,4	42,7
	Innsbruck	176	17,1	59,7
	Mühlau	50	4,8	64,6
	Pradl	188	18,2	82,8
	Vill	21	2,0	84,9
	Wilten	156	15,1	100,0
	Gesamt	1031	100,0	

Lärmquellensamplezugehörigkeit

		Häufigkeit	Gültige Prozente	Kumulierte Prozente
Gültig	Flug stark	111	10,8	10,8
	Flug mittel	111	10,8	21,5
	Flug gering	118	11,4	33,0
	Straße stark	118	11,4	44,4
	Straße mittel	119	11,5	56,0
	Straße gering	111	10,8	66,7
	Schiene stark	111	10,8	77,5
	Schiene mittel	114	11,1	88,6
	Schiene gering	118	11,4	100,0
	Gesamt	1031	100,0	

Geschlecht

		Häufigkeit	Gültige Prozente	Kumulierte Prozente
Gültig	weiblich	541	52,5	52,5
	männlich	490	47,5	100,0
	Gesamt	1031	100,0	

Altersgruppen

		Häufigkeit	Gültige Prozente	Kumulierte Prozente
Gültig	18 bis 40 Jahre	440	42,7	42,7
	41 bis 60 Jahre	316	30,6	73,3
	über 60 Jahre	275	26,7	100,0
	Gesamt	1031	100,0	

Hauptwohnsitz (Kontrollfrage)

		Häufigkeit	Gültige Prozente	Kumulierte Prozente
Gültig	Ja	1031	100,0	100,0

Aufenthalt in Innsbruck mehr als 200 Tage im Jahr (Kontrollfrage)

		Häufigkeit	Gültige Prozente	Kumulierte Prozente
Gültig	Ja	1031	100,0	100,0

Art des Hauses

		Häufigkeit	Gültige Prozente	Kumulierte Prozente
Gültig	Alleinstehendes Haus	142	13,8	13,8
	Doppelhaushälfte	48	4,7	18,4
	Reihenhaus	58	5,6	24,1
	Mehrparteienhaus <10 Wohnungen	412	40,0	64,0
	Mehrparteienhaus 10+ Wohnungen	363	35,2	99,2
	Anderes	8	,8	100,0
	Gesamt	1031	100,0	

Eigentumsverhältnisse

		Häufigkeit	Gültige Prozente	Kumulierte Prozente
Gültig	Eigentümer/in	419	40,6	40,6
	Hauptmieter/in	454	44,0	84,7
	Mitbewohner/in, Untermieter/in	158	15,3	100,0
	Gesamt	1031	100,0	

Haushaltsgröße

		Häufigkeit	Gültige Prozente	Kumulierte Prozente
Gültig	Einpersonenhaushalt	220	21,3	21,3
	Zweipersonenhaushalt	377	36,6	57,9
	Dreipersonenhaushalt	186	18,0	75,9
	Vierpersonenhaushalt	162	15,7	91,7
	Fünfpersonenhaushalt	61	5,9	97,6
	Sechspersonenhaushalt	17	1,6	99,2
	Siebenpersonenhaushalt	5	,5	99,7
	Achtpersonenhaushalt	2	,2	99,9
	Zehnpersonenhaushalt	1	,1	100,0
	Gesamt	1031	100,0	

Anzahl Kinder unter 18 Jahren im selben Haushalt

		Häufigkeit	Gültige Prozente	Kumulierte Prozente
Gültig	Keine Kinder	762	73,9	73,9
	1 Kind	112	10,9	84,8
	2 Kinder	111	10,8	95,5
	3 Kinder	36	3,5	99,0
	4 Kinder	7	,7	99,7
	5 Kinder	3	,3	100,0
	Gesamt	1031	100,0	

Stockwerk Schlafzimmer

		Häufigkeit	Gültige Prozente	Kumulierte Prozente
Gültig	2. Untergeschoß	1	,1	,1
	1. Untergeschoß	3	,3	,4
	Erdgeschoß	230	22,3	22,7
	1. Stock	363	35,2	57,9
	2. Stock	230	22,3	80,2
	3. Stock	122	11,8	92,0
	4. Stock	63	6,1	98,2
	5. Stock	11	1,1	99,2
	7. Stock	1	,1	99,3
	8. Stock	3	,3	99,6
	9. Stock	1	,1	99,7
	10. Stock	2	,2	99,9
	11. Stock	1	,1	100,0
	Gesamt	1031	100,0	

Schutz vor Verkehrslärm durch bauliche Abschirmungen (Gebäude, Mauern, Innenhöfe,...)

		Häufigkeit	Gültige Prozente	Kumulierte Prozente
Gültig	Nein, gar nicht	469	45,5	45,5
	Ja, sehr geringfügig	297	28,8	74,3
	Ja, zum Großteil	209	20,3	94,6
	Ja, komplett abgeschirmt	27	2,6	97,2
	weiß nicht / k.A.	29	2,8	100,0
	Gesamt	1031	100,0	

Schutz vor Verkehrslärm durch geschützten Innenhof bzw. lärmabgewandte Seite

		Häufigkeit	Gültige Prozente	Kumulierte Prozente
Gültig	Ja	701	68,0	68,0
	Nein	308	29,9	97,9
	weiß nicht/ k.A.	22	2,1	100,0
	Gesamt	1031	100,0	

Nachtschlaf bei geöffneten oder gekippten Fenstern

		Häufigkeit	Gültige Prozente	Kumulierte Prozente
Gültig	Ja, häufig bis immer	513	49,8	49,8
	Ja, gelegentlich	308	29,9	79,6
	Nein, Wohnraumlüftung vorhanden	7	,7	80,3
	Nein, das möchte ich nicht	68	6,6	86,9
	Nein, das wäre zu laut	127	12,3	99,2
	keine Angabe	8	,8	100,0
	Gesamt	1031	100,0	

Lärmschutzfenster in den letzten 10 Jahren eingebaut

		Häufigkeit	Gültige Prozente	Kumulierte Prozente
Gültig	Ja	187	18,1	18,1
	Nein	672	65,2	83,3
	weiß nicht/ k.A.	172	16,7	100,0
	Gesamt	1031	100,0	

Schutz Lärmschutzfenster vor welcher Lärmquelle

		Häufigkeit	Gültige Prozente	Kumulierte Prozente
Gültig	Autobahnlärm	6	,6	,6
	Straßenverkehrslärm	110	10,7	11,3
	Schienenverkehrslärm	16	1,6	12,8
	Fluglärm	28	2,7	15,5
	Andere Lärmquellen	27	2,6	18,1
	Keine Lärmschutzfenster vorhanden / w.n.	844	81,9	100,0
	Gesamt	1031	100,0	

Ausgesetztheit Lärm gesamt

		Häufigkeit	Gültige Prozente	Kumulierte Prozente
Gültig	gar nicht	70	6,8	6,8
	1	62	6,0	12,8
	2	129	12,5	25,3
	3	186	18,0	43,4
	4	127	12,3	55,7
	5	159	15,4	71,1
	6	75	7,3	78,4
	7	83	8,1	86,4
	8	71	6,9	93,3
	9	27	2,6	95,9
	äußerst stark	42	4,1	100,0
	Gesamt	1031	100,0	

Ausgesetztheit Autobahnlärm

		Häufigkeit	Gültige Prozente	Kumulierte Prozente
Gültig	gar nicht	821	79,6	79,6
	1	56	5,4	85,1
	2	54	5,2	90,3
	3	34	3,3	93,6
	4	15	1,5	95,1
	5	13	1,3	96,3
	6	11	1,1	97,4
	7	8	,8	98,2
	8	8	,8	98,9
	9	4	,4	99,3
	äußerst stark	7	,7	100,0
	Gesamt	1031	100,0	

Ausgesetztheit Strassenverkehrslärm

		Häufigkeit	Gültige Prozente	Kumulierte Prozente
Gültig	gar nicht	158	15,3	15,3
	1	108	10,5	25,8
	2	164	15,9	41,7
	3	133	12,9	54,6
	4	96	9,3	63,9
	5	102	9,9	73,8
	6	70	6,8	80,6
	7	63	6,1	86,7
	8	44	4,3	91,0
	9	38	3,7	94,7
	äußerst stark	55	5,3	100,0
	Gesamt	1031	100,0	

Ausgesetztheit Schienenverkehrslärm

		Häufigkeit	Gültige Prozente	Kumulierte Prozente
Gültig	gar nicht	721	69,9	69,9
	1	71	6,9	76,8
	2	53	5,1	82,0
	3	51	4,9	86,9
	4	35	3,4	90,3
	5	30	2,9	93,2
	6	18	1,7	95,0
	7	23	2,2	97,2
	8	6	,6	97,8
	9	10	1,0	98,7
	äußerst stark	13	1,3	100,0
	Gesamt	1031	100,0	

Ausgesetztheit Flugverkehrslärm

		Häufigkeit	Gültige Prozente	Kumulierte Prozente
Gültig	gar nicht	149	14,5	14,5
	1	116	11,3	25,7
	2	134	13,0	38,7
	3	120	11,6	50,3
	4	114	11,1	61,4
	5	126	12,2	73,6
	6	59	5,7	79,3
	7	48	4,7	84,0
	8	67	6,5	90,5
	9	49	4,8	95,2
	äußerst stark	49	4,8	100,0
	Gesamt	1031	100,0	

Ausgesetztheit Lärm von Gewerbe und Industrie

		Häufigkeit	Gültige Prozente	Kumulierte Prozente
Gültig	gar nicht	831	80,6	80,6
	1	53	5,1	85,7
	2	48	4,7	90,4
	3	34	3,3	93,7
	4	14	1,4	95,1
	5	13	1,3	96,3
	6	9	,9	97,2
	7	9	,9	98,1
	8	7	,7	98,7
	9	4	,4	99,1
	äußerst stark	9	,9	100,0
	Gesamt	1031	100,0	

Ausgesetztheit Lärm von Passanten u. Gaststätten

		Häufigkeit	Gültige Prozente	Kumulierte Prozente
Gültig	gar nicht	509	49,4	49,4
	1	108	10,5	59,8
	2	96	9,3	69,2
	3	94	9,1	78,3
	4	58	5,6	83,9
	5	47	4,6	88,5
	6	26	2,5	91,0
	7	32	3,1	94,1
	8	25	2,4	96,5
	9	15	1,5	98,0
	äußerst stark	21	2,0	100,0
	Gesamt	1031	100,0	

Ausgesetztheit Lärm von Baustellen

		Häufigkeit	Gültige Prozente	Kumulierte Prozente
Gültig	gar nicht	458	44,4	44,4
	1	111	10,8	55,2
	2	104	10,1	65,3
	3	72	7,0	72,3
	4	53	5,1	77,4
	5	58	5,6	83,0
	6	32	3,1	86,1
	7	36	3,5	89,6
	8	29	2,8	92,4
	9	20	1,9	94,4
	äußerst stark	58	5,6	100,0
	Gesamt	1031	100,0	

Ausgesetztheit Nachbarschaftslärm

		Häufigkeit	Gültige Prozente	Kumulierte Prozente
Gültig	gar nicht	418	40,5	40,5
	1	140	13,6	54,1
	2	153	14,8	69,0
	3	91	8,8	77,8
	4	56	5,4	83,2
	5	46	4,5	87,7
	6	34	3,3	91,0
	7	30	2,9	93,9
	8	27	2,6	96,5
	9	16	1,6	98,1
	äußerst stark	20	1,9	100,0
	Gesamt	1031	100,0	

Ausgesetztheit Erschütterungen gesamt

		Häufigkeit	Gültige Prozente	Kumulierte Prozente
Gültig	gar nicht	652	63,2	63,2
	1	89	8,6	71,9
	2	65	6,3	78,2
	3	56	5,4	83,6
	4	47	4,6	88,2
	5	27	2,6	90,8
	6	30	2,9	93,7
	7	23	2,2	95,9
	8	20	1,9	97,9
	9	9	,9	98,7
	äußerst stark	13	1,3	100,0
	Gesamt	1031	100,0	

Ausmaß Windeinfluss auf Lärm

		Häufigkeit	Gültige Prozente	Kumulierte Prozente
Gültig	Gar nicht	262	25,4	25,4
	Eher wenig	421	40,8	66,2
	Eher stark	232	22,5	88,7
	Sehr stark	78	7,6	96,3
	weiß nicht/ k.A.	38	3,7	100,0
	Gesamt	1031	100,0	

Güte Wohnbedingungen

		Häufigkeit	Gültige Prozente	Kumulierte Prozente
Gültig	gar nicht gut	26	2,5	2,5
	1	11	1,1	3,6
	2	47	4,6	8,1
	3	69	6,7	14,8
	4	67	6,5	21,3
	5	174	16,9	38,2
	6	84	8,1	46,4
	7	161	15,6	62,0
	8	196	19,0	81,0
	9	84	8,1	89,1
	außerordentlich gut	110	10,7	99,8
	Keine Angabe	2	,2	100,0
	Gesamt	1031	100,0	

Güte Nachbarschaftsbeziehungen

		Häufigkeit	Gültige Prozente	Kumulierte Prozente
Gültig	gar nicht gut	23	2,2	2,2
	1	13	1,3	3,5
	2	32	3,1	6,6
	3	67	6,5	13,1
	4	55	5,3	18,4
	5	178	17,3	35,7
	6	94	9,1	44,8
	7	147	14,3	59,1
	8	162	15,7	74,8
	9	88	8,5	83,3
	außerordentlich gut	159	15,4	98,7
	Keine Angabe	13	1,3	100,0
	Gesamt	1031	100,0	

Güte Sicherheit

		Häufigkeit	Gültige Prozente	Kumulierte Prozente
Gültig	gar nicht gut	38	3,7	3,7
	1	22	2,1	5,8
	2	49	4,8	10,6
	3	58	5,6	16,2
	4	67	6,5	22,7
	5	140	13,6	36,3
	6	87	8,4	44,7
	7	176	17,1	61,8
	8	217	21,0	82,8
	9	79	7,7	90,5
	außerordentlich gut	97	9,4	99,9
	Keine Angabe	1	,1	100,0
	Gesamt	1031	100,0	

Güte Landschaft, Natur, öffentliche Parks und Grünflächen

		Häufigkeit	Gültige Prozente	Kumulierte Prozente
Gültig	gar nicht gut	17	1,6	1,6
	1	7	,7	2,3
	2	27	2,6	4,9
	3	42	4,1	9,0
	4	42	4,1	13,1
	5	103	10,0	23,1
	6	71	6,9	30,0
	7	145	14,1	44,0
	8	222	21,5	65,6
	9	161	15,6	81,2
	außerordentlich gut	188	18,2	99,4
	Keine Angabe	6	,6	100,0
	Gesamt	1031	100,0	

Güte Luftqualität

		Häufigkeit	Gültige Prozente	Kumulierte Prozente
Gültig	gar nicht gut	17	1,6	1,6
	1	15	1,5	3,1
	2	53	5,1	8,2
	3	93	9,0	17,3
	4	99	9,6	26,9
	5	163	15,8	42,7
	6	103	10,0	52,7
	7	142	13,8	66,4
	8	180	17,5	83,9
	9	83	8,1	91,9
	außerordentlich gut	82	8,0	99,9
	Keine Angabe	1	,1	100,0
	Gesamt	1031	100,0	

Güte öffentliche Verkehrsverbindungen

		Häufigkeit	Gültige Prozente	Kumulierte Prozente
Gültig	gar nicht gut	7	,7	,7
	1	6	,6	1,3
	2	14	1,4	2,6
	3	31	3,0	5,6
	4	34	3,3	8,9
	5	64	6,2	15,1
	6	74	7,2	22,3
	7	133	12,9	35,2
	8	239	23,2	58,4
	9	200	19,4	77,8
	außerordentlich gut	217	21,0	98,8
	Keine Angabe	12	1,2	100,0
	Gesamt	1031	100,0	

Güte Bedingungen für Kinder

		Häufigkeit	Gültige Prozente	Kumulierte Prozente
Gültig	gar nicht gut	13	1,3	1,3
	1	11	1,1	2,3
	2	23	2,2	4,6
	3	41	4,0	8,5
	4	46	4,5	13,0
	5	137	13,3	26,3
	6	107	10,4	36,7
	7	184	17,8	54,5
	8	220	21,3	75,8
	9	98	9,5	85,4
	außerordentlich gut	129	12,5	97,9
	Keine Angabe	22	2,1	100,0
	Gesamt	1031	100,0	

Ausmaß Verbundenheit zur Stadt Innsbruck

		Häufigkeit	Gültige Prozente	Kumulierte Prozente
Gültig	gar nicht	22	2,1	2,1
	1	6	,6	2,7
	2	27	2,6	5,3
	3	28	2,7	8,1
	4	31	3,0	11,1
	5	98	9,5	20,6
	6	76	7,4	27,9
	7	146	14,2	42,1
	8	210	20,4	62,5
	9	146	14,2	76,6
	außerordentlich	239	23,2	99,8
	Keine Angabe	2	,2	100,0
	Gesamt	1031	100,0	

Ausmaß Zufriedenheit eigene Wohnsituation insgesamt

		Häufigkeit	Gültige Prozente	Kumulierte Prozente
Gültig	gar nicht zufrieden	13	1,3	1,3
	1	7	,7	1,9
	2	8	,8	2,7
	3	22	2,1	4,8
	4	17	1,6	6,5
	5	48	4,7	11,2
	6	54	5,2	16,4
	7	117	11,3	27,7
	8	212	20,6	48,3
	9	202	19,6	67,9
	Außerordentlich zufrieden	330	32,0	99,9
	Keine Angabe	1	,1	100,0
	Gesamt	1031	100,0	

Güte eigene Lebensqualität

		Häufigkeit	Gültige Prozente	Kumulierte Prozente
Gültig	gar nicht gut	3	,3	,3
	1	3	,3	,6
	2	4	,4	1,0
	3	15	1,5	2,4
	4	19	1,8	4,3
	5	56	5,4	9,7
	6	53	5,1	14,8
	7	147	14,3	29,1
	8	284	27,5	56,6
	9	210	20,4	77,0
	außerordentlich gut	231	22,4	99,4
	Keine Angabe	6	,6	100,0
	Gesamt	1031	100,0	

Ausmaß eigene Lärmempfindlichkeit

		Häufigkeit	Gültige Prozente	Kumulierte Prozente
Gültig	überhaupt nicht empfindlich	72	7,0	7,0
	1	66	6,4	13,4
	2	137	13,3	26,7
	3	159	15,4	42,1
	4	85	8,2	50,3
	5	184	17,8	68,2
	6	75	7,3	75,5
	7	81	7,9	83,3
	8	81	7,9	91,2
	9	46	4,5	95,6
	außerordentlich empfindlich	45	4,4	100,0
	Gesamt	1031	100,0	

Güte eigener Gesundheitszustand

		Häufigkeit	Gültige Prozente	Kumulierte Prozente
Gültig	Sehr gut	354	34,3	34,3
	Gut	400	38,8	73,1
	Zufriedenstellend	186	18,0	91,2
	Weniger gut	65	6,3	97,5
	Schlecht	24	2,3	99,8
	keine Angabe	2	,2	100,0
	Gesamt	1031	100,0	

Belästigung Lärm gesamt

		Häufigkeit	Gültige Prozente	Kumulierte Prozente
Gültig	belästigt/stört überhaupt nicht	138	13,4	13,4
	1	80	7,8	21,1
	2	160	15,5	36,7
	3	146	14,2	50,8
	4	90	8,7	59,6
	5	128	12,4	72,0
	6	72	7,0	79,0
	7	92	8,9	87,9
	8	62	6,0	93,9
	9	27	2,6	96,5
	belästigt/stört besonders stark	36	3,5	100,0
	Gesamt	1031	100,0	

Belästigung Autobahnlärm

		Häufigkeit	Gültige Prozente	Kumulierte Prozente
Gültig	belästigt/stört überhaupt nicht	849	82,3	82,3
	1	49	4,8	87,1
	2	49	4,8	91,9
	3	26	2,5	94,4
	4	9	,9	95,2
	5	13	1,3	96,5
	6	8	,8	97,3
	7	6	,6	97,9
	8	9	,9	98,7
	9	3	,3	99,0
	belästigt/stört besonders stark	10	1,0	100,0
	Gesamt	1031	100,0	

Belästigung Strassenverkehrslärm

		Häufigkeit	Gültige Prozente	Kumulierte Prozente
Gültig	belästigt/stört überhaupt nicht	252	24,4	24,4
	1	127	12,3	36,8
	2	160	15,5	52,3
	3	111	10,8	63,0
	4	82	8,0	71,0
	5	82	8,0	79,0
	6	59	5,7	84,7
	7	41	4,0	88,7
	8	53	5,1	93,8
	9	28	2,7	96,5
	belästigt/stört besonders stark	36	3,5	100,0
	Gesamt	1031	100,0	

Belästigung Schienenverkehrslärm

		Häufigkeit	Gültige Prozente	Kumulierte Prozente
Gültig	belästigt/stört überhaupt nicht	712	69,1	69,1
	1	71	6,9	75,9
	2	57	5,5	81,5
	3	60	5,8	87,3
	4	33	3,2	90,5
	5	30	2,9	93,4
	6	17	1,6	95,1
	7	21	2,0	97,1
	8	15	1,5	98,5
	9	8	,8	99,3
	belästigt/stört besonders stark	7	,7	100,0
	Gesamt	1031	100,0	

Belästigung Fluglärm

		Häufigkeit	Gültige Prozente	Kumulierte Prozente
Gültig	belästigt/stört überhaupt nicht	249	24,2	24,2
	1	125	12,1	36,3
	2	136	13,2	49,5
	3	119	11,5	61,0
	4	83	8,1	69,1
	5	104	10,1	79,1
	6	52	5,0	84,2
	7	42	4,1	88,3
	8	68	6,6	94,9
	9	24	2,3	97,2
	belästigt/stört besonders stark	29	2,8	100,0
	Gesamt	1031	100,0	

Belästigung Lärm aus Gewerbe u. Industrie

		Häufigkeit	Gültige Prozente	Kumulierte Prozente
Gültig	belästigt/stört überhaupt nicht	836	81,1	81,1
	1	45	4,4	85,5
	2	56	5,4	90,9
	3	28	2,7	93,6
	4	13	1,3	94,9
	5	20	1,9	96,8
	6	8	,8	97,6
	7	10	1,0	98,5
	8	5	,5	99,0
	9	2	,2	99,2
	belästigt/stört besonders stark	8	,8	100,0
	Gesamt	1031	100,0	

Belästigung Lärm von Passanten und Gaststätten

		Häufigkeit	Gültige Prozente	Kumulierte Prozente
Gültig	belästigt/stört überhaupt nicht	545	52,9	52,9
	1	91	8,8	61,7
	2	112	10,9	72,6
	3	96	9,3	81,9
	4	41	4,0	85,8
	5	49	4,8	90,6
	6	26	2,5	93,1
	7	21	2,0	95,2
	8	19	1,8	97,0
	9	13	1,3	98,3
	belästigt/stört besonders stark	18	1,7	100,0
	Gesamt	1031	100,0	

Belästigung Baustellenlärm

		Häufigkeit	Gültige Prozente	Kumulierte Prozente
Gültig	belästigt/stört überhaupt nicht	500	48,5	48,5
	1	86	8,3	56,8
	2	84	8,1	65,0
	3	87	8,4	73,4
	4	42	4,1	77,5
	5	57	5,5	83,0
	6	35	3,4	86,4
	7	43	4,2	90,6
	8	33	3,2	93,8
	9	22	2,1	95,9
	belästigt/stört besonders stark	41	4,0	99,9
	Keine Angabe	1	,1	100,0
	Gesamt	1031	100,0	

Belästigung Nachbarschaftslärm

		Häufigkeit	Gültige Prozente	Kumulierte Prozente
Gültig	belästigt/stört überhaupt nicht	480	46,6	46,6
	1	147	14,3	60,8
	2	109	10,6	71,4
	3	92	8,9	80,3
	4	59	5,7	86,0
	5	46	4,5	90,5
	6	22	2,1	92,6
	7	24	2,3	95,0
	8	24	2,3	97,3
	9	11	1,1	98,4
	belästigt/stört besonders stark	17	1,6	100,0
	Gesamt	1031	100,0	

Belästigung Erschütterungen gesamt

		Häufigkeit	Gültige Prozente	Kumulierte Prozente
Gültig	belästigt/stört überhaupt nicht	723	70,1	70,1
	1	77	7,5	77,6
	2	62	6,0	83,6
	3	37	3,6	87,2
	4	31	3,0	90,2
	5	32	3,1	93,3
	6	18	1,7	95,1
	7	17	1,6	96,7
	8	19	1,8	98,5
	9	4	,4	98,9
	belästigt/stört besonders stark	11	1,1	100,0
	Gesamt	1031	100,0	

Belästigung im Schlaf Lärm gesamt

		Häufigkeit	Gültige Prozente	Kumulierte Prozente
Gültig	belästigt/stört überhaupt nicht	470	45,6	45,6
	1	92	8,9	54,5
	2	133	12,9	67,4
	3	82	8,0	75,4
	4	46	4,5	79,8
	5	63	6,1	85,9
	6	34	3,3	89,2
	7	31	3,0	92,2
	8	41	4,0	96,2
	9	17	1,6	97,9
	belästigt/stört besonders stark	22	2,1	100,0
	Gesamt	1031	100,0	

Belästigung im Schlaf Autobahnlärm

		Häufigkeit	Gültige Prozente	Kumulierte Prozente
Gültig	belästigt/stört überhaupt nicht	948	91,9	91,9
	1	25	2,4	94,4
	2	16	1,6	95,9
	3	11	1,1	97,0
	4	4	,4	97,4
	5	8	,8	98,2
	6	5	,5	98,6
	7	4	,4	99,0
	8	3	,3	99,3
	9	3	,3	99,6
	belästigt/stört besonders stark	4	,4	100,0
	Gesamt	1031	100,0	

Belästigung im Schlaf Straßenverkehrslärm

		Häufigkeit	Gültige Prozente	Kumulierte Prozente
Gültig	belästigt/stört überhaupt nicht	595	57,7	57,7
	1	103	10,0	67,7
	2	94	9,1	76,8
	3	49	4,8	81,6
	4	45	4,4	85,9
	5	35	3,4	89,3
	6	27	2,6	91,9
	7	27	2,6	94,6
	8	31	3,0	97,6
	9	9	,9	98,4
	belästigt/stört besonders stark	16	1,6	100,0
	Gesamt	1031	100,0	

Belästigung im Schlaf Schienenverkehrslärm

		Häufigkeit	Gültige Prozente	Kumulierte Prozente
Gültig	belästigt/stört überhaupt nicht	861	83,5	83,5
	1	36	3,5	87,0
	2	37	3,6	90,6
	3	18	1,7	92,3
	4	20	1,9	94,3
	5	23	2,2	96,5
	6	7	,7	97,2
	7	14	1,4	98,5
	8	3	,3	98,8
	9	6	,6	99,4
	belästigt/stört besonders stark	6	,6	100,0
	Gesamt	1031	100,0	

Belästigung im Schlaf Fluglärm

		Häufigkeit	Gültige Prozente	Kumulierte Prozente
Gültig	belästigt/stört überhaupt nicht	725	70,3	70,3
	1	73	7,1	77,4
	2	72	7,0	84,4
	3	40	3,9	88,3
	4	30	2,9	91,2
	5	24	2,3	93,5
	6	9	,9	94,4
	7	18	1,7	96,1
	8	13	1,3	97,4
	9	10	1,0	98,4
	belästigt/stört besonders stark	17	1,6	100,0
	Gesamt	1031	100,0	

Belästigung im Schlaf Lärm von Gewerbe und Industrie

		Häufigkeit	Gültige Prozente	Kumulierte Prozente
Gültig	belästigt/stört überhaupt nicht	931	90,3	90,3
	1	30	2,9	93,2
	2	22	2,1	95,3
	3	8	,8	96,1
	4	6	,6	96,7
	5	9	,9	97,6
	6	8	,8	98,4
	7	7	,7	99,0
	8	2	,2	99,2
	9	4	,4	99,6
	belästigt/stört besonders stark	4	,4	100,0
	Gesamt	1031	100,0	

Belästigung im Schlaf Lärm von Passanten und Gaststätten

		Häufigkeit	Gültige Prozente	Kumulierte Prozente
Gültig	belästigt/stört überhaupt nicht	668	64,8	64,8
	1	97	9,4	74,2
	2	58	5,6	79,8
	3	48	4,7	84,5
	4	33	3,2	87,7
	5	32	3,1	90,8
	6	17	1,6	92,4
	7	21	2,0	94,5
	8	27	2,6	97,1
	9	13	1,3	98,4
	belästigt/stört besonders stark	17	1,6	100,0
	Gesamt	1031	100,0	

Belästigung im Schlaf Lärm von Baustellen

		Häufigkeit	Gültige Prozente	Kumulierte Prozente
Gültig	belästigt/stört überhaupt nicht	837	81,2	81,2
	1	49	4,8	85,9
	2	30	2,9	88,8
	3	22	2,1	91,0
	4	21	2,0	93,0
	5	14	1,4	94,4
	6	15	1,5	95,8
	7	20	1,9	97,8
	8	11	1,1	98,8
	9	3	,3	99,1
	belästigt/stört besonders stark	8	,8	99,9
	Keine Angabe	1	,1	100,0
	Gesamt	1031	100,0	

Belästigung im Schlaf Nachbarschaftslärm

		Häufigkeit	Gültige Prozente	Kumulierte Prozente
Gültig	belästigt/stört überhaupt nicht	666	64,6	64,6
	1	108	10,5	75,1
	2	68	6,6	81,7
	3	51	4,9	86,6
	4	26	2,5	89,1
	5	27	2,6	91,8
	6	14	1,4	93,1
	7	27	2,6	95,7
	8	19	1,8	97,6
	9	11	1,1	98,6
	belästigt/stört besonders stark	14	1,4	100,0
	Gesamt	1031	100,0	

Belästigung im Schlaf Erschütterungen gesamt

		Häufigkeit	Gültige Prozente	Kumulierte Prozente
Gültig	belästigt/stört überhaupt nicht	861	83,5	83,5
	1	56	5,4	88,9
	2	29	2,8	91,8
	3	27	2,6	94,4
	4	12	1,2	95,5
	5	18	1,7	97,3
	6	10	1,0	98,3
	7	6	,6	98,8
	8	5	,5	99,3
	9	3	,3	99,6
	belästigt/stört besonders stark	4	,4	100,0
	Gesamt	1031	100,0	

Lärmcoping: Fenster im Sommer nachts geschlossen

		Häufigkeit	Gültige Prozente	Kumulierte Prozente
Gültig	Ja	472	45,8	45,8
	Nein	559	54,2	100,0
	Gesamt	1031	100,0	

Lärmcoping: Garten/Balkon nicht genutzt

		Häufigkeit	Gültige Prozente	Kumulierte Prozente
Gültig	Ja	218	21,1	21,1
	Nein	812	78,8	99,9
	Keine Angabe	1	,1	100,0
	Gesamt	1031	100,0	

Lärmcoping: Andere Nutzung Wohnräume

		Häufigkeit	Gültige Prozente	Kumulierte Prozente
Gültig	Ja	159	15,4	15,4
	Nein	872	84,6	100,0
	Gesamt	1031	100,0	

Lärmcoping: Ärger über Verkehrslärm

		Häufigkeit	Gültige Prozente	Kumulierte Prozente
Gültig	Ja	568	55,1	55,1
	Nein	463	44,9	100,0
	Gesamt	1031	100,0	

Lärmcoping: Hilflosigkeit wegen Verkehrslärm

		Häufigkeit	Gültige Prozente	Kumulierte Prozente
Gültig	Ja	246	23,9	23,9
	Nein	785	76,1	100,0
	Gesamt	1031	100,0	

Lärmcoping: Gefühlte Zunahme Lärmbelastung

		Häufigkeit	Gültige Prozente	Kumulierte Prozente
Gültig	Ja	436	42,3	42,3
	Nein	595	57,7	100,0
	Gesamt	1031	100,0	

Lärmcoping: Gefühlte Abnahme Lärmbelastung

		Häufigkeit	Gültige Prozente	Kumulierte Prozente
Gültig	Ja	58	5,6	5,6
	Nein	973	94,4	100,0
	Gesamt	1031	100,0	

Lärmcoping: Gespräche mit Nachbarn über Lärm

		Häufigkeit	Gültige Prozente	Kumulierte Prozente
Gültig	Ja	354	34,3	34,3
	Nein	677	65,7	100,0
	Gesamt	1031	100,0	

Lärmcoping: Gespräche mit Behörden über Lärm

		Häufigkeit	Gültige Prozente	Kumulierte Prozente
Gültig	Ja	134	13,0	13,0
	Nein	897	87,0	100,0
	Gesamt	1031	100,0	

Derzeitige berufliche Stellung

		Häufigkeit	Gültige Prozente	Kumulierte Prozente
Gültig	Selbständig	88	8,5	8,5
	Angestellte(r)	349	33,9	42,4
	Vertragsbedienstete/r oder Beamte/r	47	4,6	46,9
	Facharbeiter/in	21	2,0	49,0
	angelernte/r Arbeiter/in	19	1,8	50,8
	Hausfrau/-Mann	40	3,9	54,7
	in Ausbildung	144	14,0	68,7
	Präsenz- oder Zivildiener	3	,3	69,0
	mithelfend im Familienbetrieb	1	,1	69,1
	Pensionist/in	266	25,8	94,9
	derzeit nicht berufstätig	53	5,1	100,0
	Gesamt	1031	100,0	

Höchster bisher erreichter Bildungsabschluss

		Häufigkeit	Gültige Prozente	Kumulierte Prozente
Gültig	Pflichtschule	98	9,5	9,5
	Abgeschlossene Lehre	166	16,1	25,6
	Fachschule ohne Matura	114	11,1	36,7
	Matura	262	25,4	62,1
	Universität, FH oder Vergleichbares	388	37,6	99,7
	Keine Angabe	3	,3	100,0
	Gesamt	1031	100,0	

Arbeits- oder Ausbildungsplatz in Innsbruck

		Häufigkeit	Gültige Prozente	Kumulierte Prozente
Gültig	Ja	573	55,6	55,6
	Nein	98	9,5	65,1
	Nicht zutreffend (in Pension ...)	359	34,8	99,9
	Keine Angabe	1	,1	100,0
	Gesamt	1031	100,0	

Vorrangiges Verkehrsmittel auf dem Weg zum Arbeits- oder Ausbildungsplatz

		Häufigkeit	Gültige Prozente	Kumulierte Prozente
Gültig	Auto oder Motorrad	206	20,0	20,0
	Eisenbahn	10	1,0	21,0
	Bus oder andere Öffis	148	14,4	35,3
	Fahrrad	203	19,7	55,0
	Ich gehe zu Fuß	106	10,3	65,3
	Nicht zutreffend (in Pension ...)	358	34,7	100,0
	Gesamt	1031	100,0	

Häufigkeit Nutzung eines Autos als Fahrer/in

		Häufigkeit	Gültige Prozente	Kumulierte Prozente
Gültig	(Fast) täglich	243	23,6	23,6
	Mehrmals pro Woche	269	26,1	49,7
	Mehrmals pro Monat	182	17,7	67,3
	Mehrmals pro Jahr	44	4,3	71,6
	(Fast) nie	293	28,4	100,0
	Gesamt	1031	100,0	

Häufigkeit Nutzung öffentl. Verkehrsmittel innerhalb der Stadt

		Häufigkeit	Gültige Prozente	Kumulierte Prozente
Gültig	(Fast) täglich	193	18,7	18,7
	Mehrmals pro Woche	194	18,8	37,5
	Mehrmals pro Monat	231	22,4	59,9
	Mehrmals pro Jahr	142	13,8	73,7
	(Fast) nie	270	26,2	99,9
	Keine Angabe	1	,1	100,0
	Gesamt	1031	100,0	

Häufigkeit Nutzung der Eisenbahn oder von Autobussen außerhalb der Stadt

		Häufigkeit	Gültige Prozente	Kumulierte Prozente
Gültig	(Fast) täglich	12	1,2	1,2
	Mehrmals pro Woche	46	4,5	5,6
	Mehrmals pro Monat	162	15,7	21,3
	Mehrmals pro Jahr	347	33,7	55,0
	(Fast) nie	463	44,9	99,9
	Keine Angabe	1	,1	100,0
	Gesamt	1031	100,0	

Häufigkeit Nutzung eines Fahrrads

		Häufigkeit	Gültige Prozente	Kumulierte Prozente
Gültig	(Fast) täglich	310	30,1	30,1
	Mehrmals pro Woche	159	15,4	45,5
	Mehrmals pro Monat	136	13,2	58,7
	Mehrmals pro Jahr	78	7,6	66,2
	(Fast) nie	348	33,8	100,0
	Gesamt	1031	100,0	

Häufigkeit länger als 10 Minuten zu Fuß gehen

		Häufigkeit	Gültige Prozente	Kumulierte Prozente
Gültig	(Fast) täglich	622	60,3	60,3
	Mehrmals pro Woche	280	27,2	87,5
	Mehrmals pro Monat	78	7,6	95,1
	Mehrmals pro Jahr	17	1,6	96,7
	(Fast) nie	34	3,3	100,0
	Gesamt	1031	100,0	

Häufigkeit Nutzung eines Flugzeuges

		Häufigkeit	Gültige Prozente	Kumulierte Prozente
Gültig	Mehrmals pro Woche	3	,3	,3
	Mehrmals pro Monat	11	1,1	1,4
	Mehrmals pro Jahr	310	30,1	31,4
	Alle paar Jahre	521	50,5	82,0
	Nie	186	18,0	100,0
	Gesamt	1031	100,0	

Einschätzung des Ausmaßes negativer Auswirkungen von Lärm auf die Gesundheit

		Häufigkeit	Gültige Prozente	Kumulierte Prozente
Gültig	Sehr wenig	59	5,7	5,7
	Eher wenig	158	15,3	21,0
	Eher stark	438	42,5	63,5
	Sehr stark	343	33,3	96,8
	Weiß nicht/ k.A.	33	3,2	100,0
	Gesamt	1031	100,0	

Bekanntheitsgrad Umgebungslärmkarten

		Häufigkeit	Gültige Prozente	Kumulierte Prozente
Gültig	Ja, kenne ich	221	21,4	21,4
	Nein, ist mir unbekannt	810	78,6	100,0
	Gesamt	1031	100,0	

Lautheit Gesamtlärm in Innsbruck im Vergleich zu anderen Städten Österreichs

		Häufigkeit	Gültige Prozente	Kumulierte Prozente
Gültig	Viel ruhiger	31	3,0	3,0
	Ruhiger	261	25,3	28,3
	Gleich ruhig / laut	551	53,4	81,8
	Lauter	150	14,5	96,3
	Viel lauter	24	2,3	98,6
	Weiß nicht/ k.A.	14	1,4	100,0
	Gesamt	1031	100,0	

Einschätzung eigener Beitrag zur Gesamtlärmbelastung in Innsbruck

		Häufigkeit	Gültige Prozente	Kumulierte Prozente
Gültig	Sehr gering	379	36,8	36,8
	Gering	396	38,4	75,2
	Durchschnittlich	227	22,0	97,2
	Stark	23	2,2	99,4
	Sehr stark	6	,6	100,0
	Gesamt	1031	100,0	

Einschätzung störendste Lärmquelle für die Innsbruckerinnen und Innsbrucker insgesamt

		Häufigkeit	Gültige Prozente	Kumulierte Prozente
Gültig	Straßenverkehrslärm	397	38,5	38,5
	Schienenverkehrslärm	16	1,6	40,1
	Fluglärm	239	23,2	63,2
	Lärm von Gewerbe und Industrie	3	,3	63,5
	Lärm von Passanten und Gaststätten	52	5,0	68,6
	Baustellenlärm	223	21,6	90,2
	Nachbarschaftslärm	37	3,6	93,8
	Weiß nicht/ k.A.	64	6,2	100,0
	Gesamt	1031	100,0	

Notwendigkeit (weiterer) Maßnahmen zur Lärmreduktion in Innsbruck

		Häufigkeit	Gültige Prozente	Kumulierte Prozente
Gültig	Ja	691	67,0	67,0
	Nein	192	18,6	85,6
	Weiß nicht/ k.A.	148	14,4	100,0
	Gesamt	1031	100,0	

Subkategorie: Förderung lärmarmer Verkehrsmittel, Maschinen usw.

		Häufigkeit	Gültige Prozente	Kumulierte Prozente
Gültig	Ja	600	86,8	86,8
	Nein	81	11,7	98,6
	Weiß nicht/ k.A.	10	1,4	100,0
	Gesamt	691	100,0	
Fehlend	System	340		
Gesamt		1031		

Subkategorie: Strengere Vorschriften und Auflagen für Lärmverursacher

		Häufigkeit	Gültige Prozente	Kumulierte Prozente
Gültig	Ja	514	74,4	74,4
	Nein	170	24,6	99,0
	Weiß nicht/ k.A.	7	1,0	100,0
	Gesamt	691	100,0	
Fehlend	System	340		
Gesamt		1031		

Subkategorie: Förderung von Lärmschutzmaßnahmen

		Häufigkeit	Gültige Prozente	Kumulierte Prozente
Gültig	Ja	581	84,1	84,1
	Nein	97	14,0	98,1
	Weiß nicht/ k.A.	13	1,9	100,0
	Gesamt	691	100,0	
Fehlend	System	340		
Gesamt		1031		

Subkategorie: Strengere Kontrollen der Einhaltung von Lärmgrenzwerten

		Häufigkeit	Gültige Prozente	Kumulierte Prozente
Gültig	Ja	539	78,0	78,0
	Nein	135	19,5	97,5
	Weiß nicht/ k.A.	17	2,5	100,0
	Gesamt	691	100,0	
Fehlend	System	340		
Gesamt		1031		

Subkategorie: Bewusstseinsbildung zur Lärmvermeidung

		Häufigkeit	Gültige Prozente	Kumulierte Prozente
Gültig	Ja	575	83,2	83,2
	Nein	107	15,5	98,7
	Weiß nicht/ k.A.	9	1,3	100,0
	Gesamt	691	100,0	
Fehlend	System	340		
Gesamt		1031		

Subkategorie: Verkehrsbeschränkungen

		Häufigkeit	Gültige Prozente	Kumulierte Prozente
Gültig	Ja	371	53,7	53,7
	Nein	303	43,8	97,5
	Weiß nicht/ k.A.	17	2,5	100,0
	Gesamt	691	100,0	
Fehlend	System	340		
Gesamt		1031		

Subkategorie: Finanzielle Auflagen für Lärmverursacher

		Häufigkeit	Gültige Prozente	Kumulierte Prozente
Gültig	Ja	402	58,2	58,2
	Nein	277	40,1	98,3
	Weiß nicht/ k.A.	12	1,7	100,0
	Gesamt	691	100,0	
Fehlend	System	340		
Gesamt		1031		

Subkategorie: Schaffung, Schutz und Erhalt lärmarmer Bereiche

		Häufigkeit	Gültige Prozente	Kumulierte Prozente
Gültig	Ja	611	88,4	88,4
	Nein	65	9,4	97,8
	Weiß nicht/ k.A.	15	2,2	100,0
	Gesamt	691	100,0	
Fehlend	System	340		
Gesamt		1031		

Subkategorie: Andere Maßnahmen

		Häufigkeit	Gültige Prozente	Kumulierte Prozente
Gültig	Ja	333	48,2	48,2
	Nein	319	46,2	94,4
	Weiß nicht/ k.A.	39	5,6	100,0
	Gesamt	691	100,0	
Fehlend	System	340		
Gesamt		1031		

Abweichungsvariable für Geschlechterverteilung <0,5%

		Häufigkeit	Gültige Prozente	Kumulierte Prozente
Gültig	Nicht berücksichtigte Fragebögen	17	1,6	1,6
	Berücksichtigte Gesamtheit	1014	98,4	100,0
	Gesamt	1031	100,0	

Impressum

Medieninhaber: Dipl.-HTL-Ing. Mag. Christoph Lechner (Amt der Tiroler Landesregierung, Abteilung Emissionen Sicherheitstechnik Anlagen)
Mag. Dr. David Schnaiter

Herausgeber: Amt der Tiroler Landesregierung, Eduard-Wallnöfer-Platz 3,
6020 Innsbruck, Tel.: +43 512 508, Mail: post@tirol.gv.at
DVR-Nummer: 0059463

Copyright: – **Alle Rechte vorbehalten** –
