# Supplementary figures and images for: Combined Effects of Aircraft, Rail, and Road Traffic Noise on Total Noise Annoyance—A Cross-Sectional Study in Innsbruck
Source: Int J Environ Res Public Health. 2019 Sep 19;16(18):3504. doi: 10.3390/ijerph16183504 (PMC6766025; doi:10.3390/ijerph16183504)

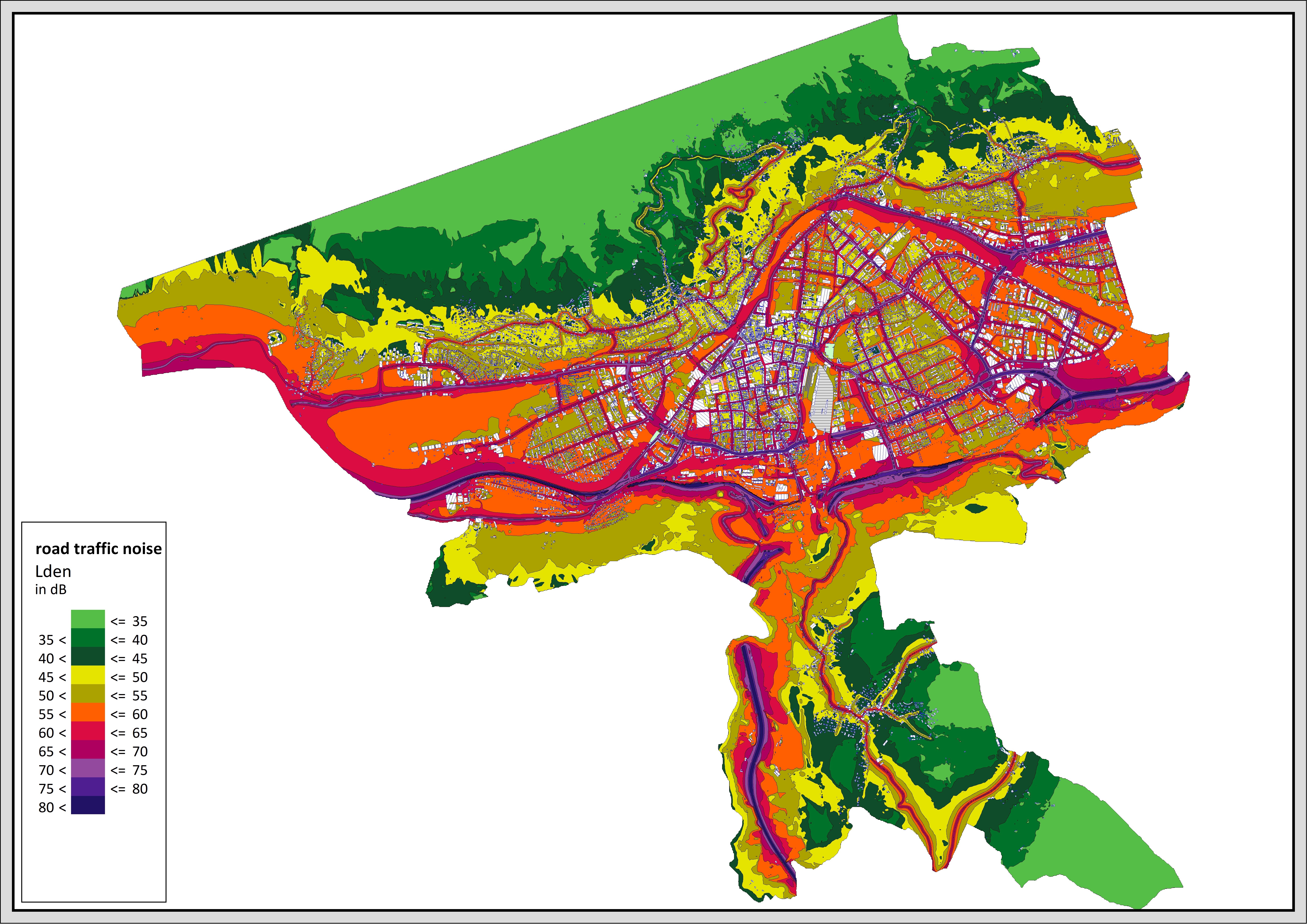

Supplement: Supplementary file 1 [file ijerph-16-03504-s001.zip › figure S 1 Lden_road.jpg]

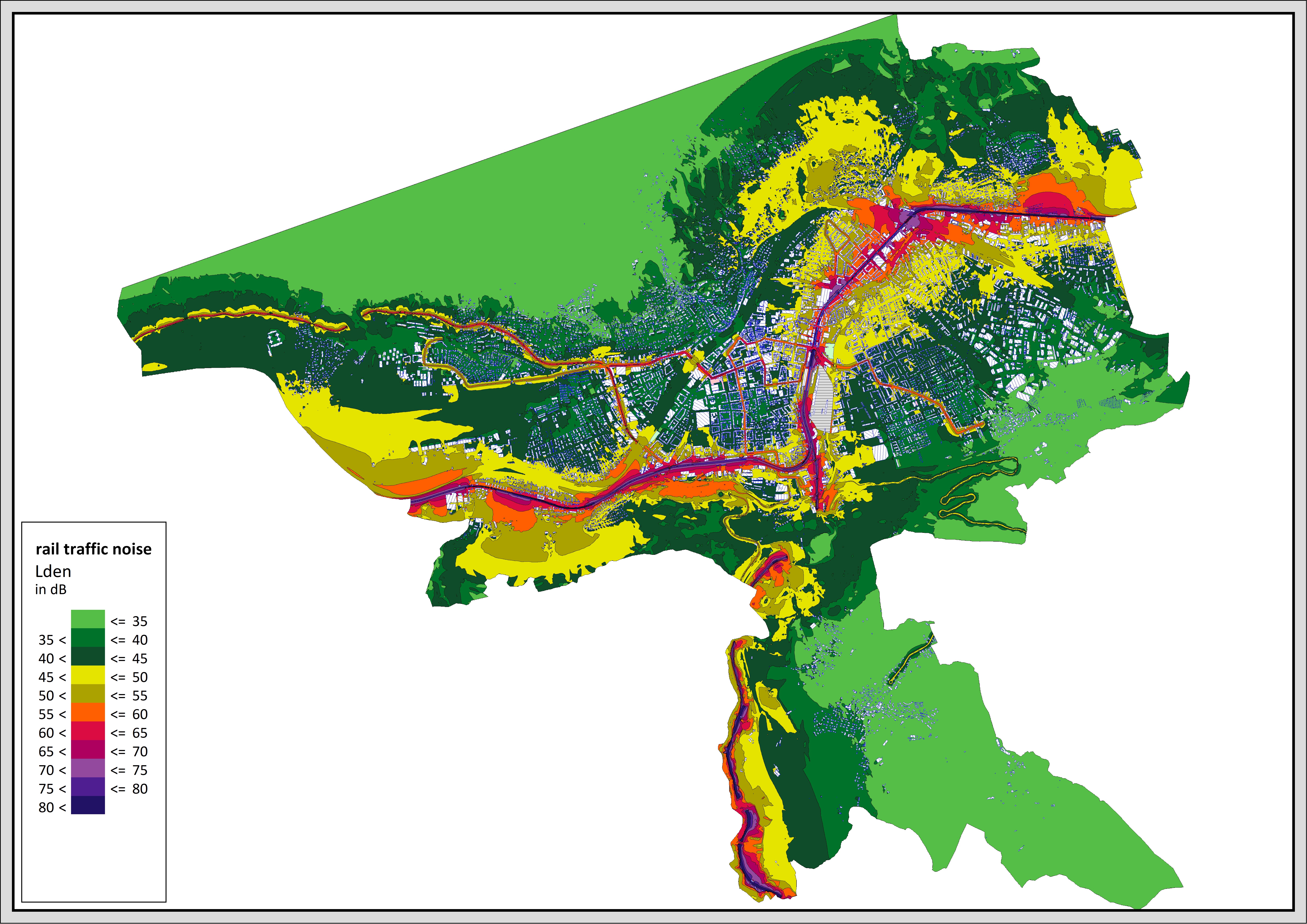

Supplement: Supplementary file 1 [file ijerph-16-03504-s001.zip › figure S 2 Lden_rail.jpg]

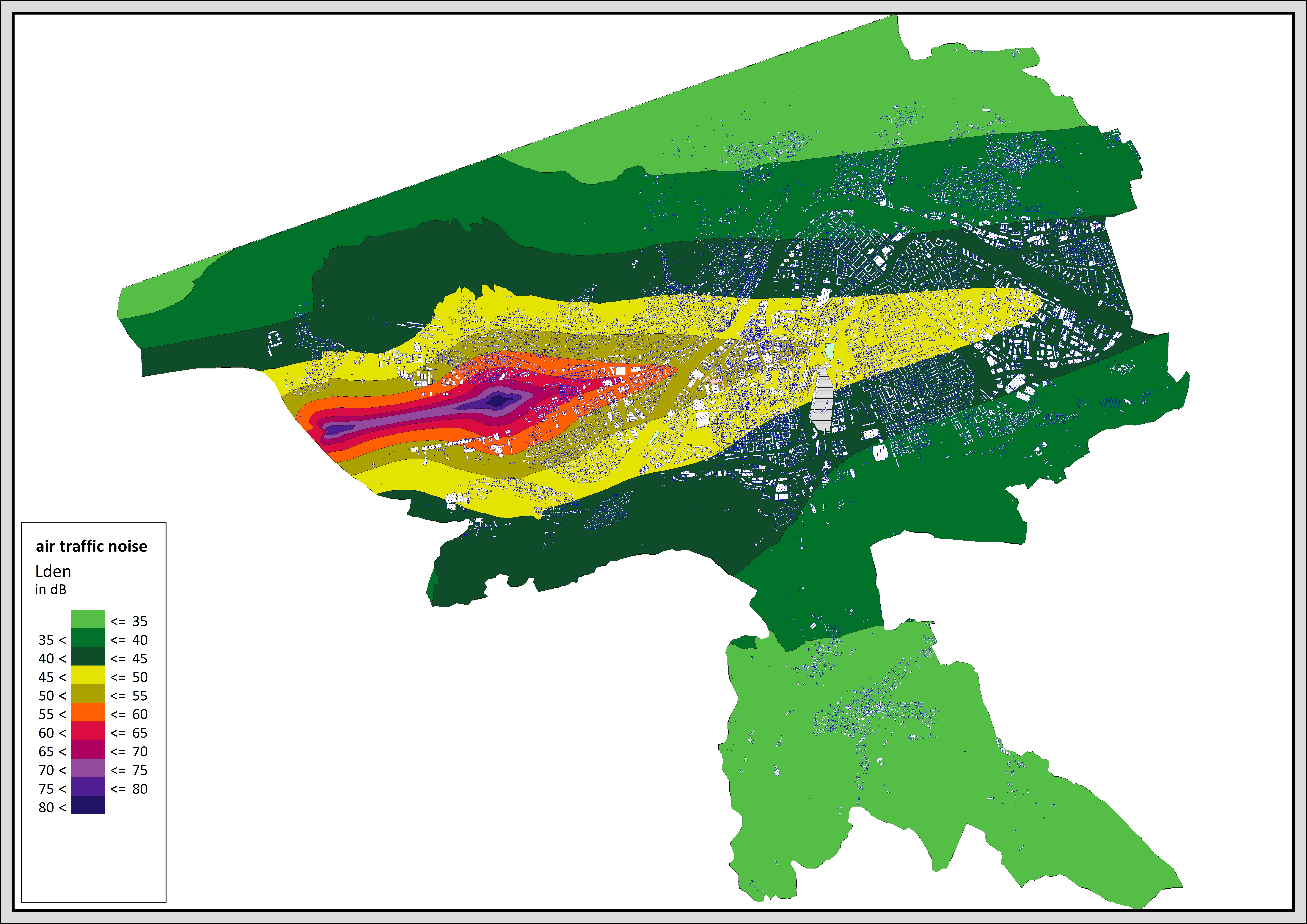

Supplement: Supplementary file 1 [file ijerph-16-03504-s001.zip › figure S 3 Lden_air.jpg]

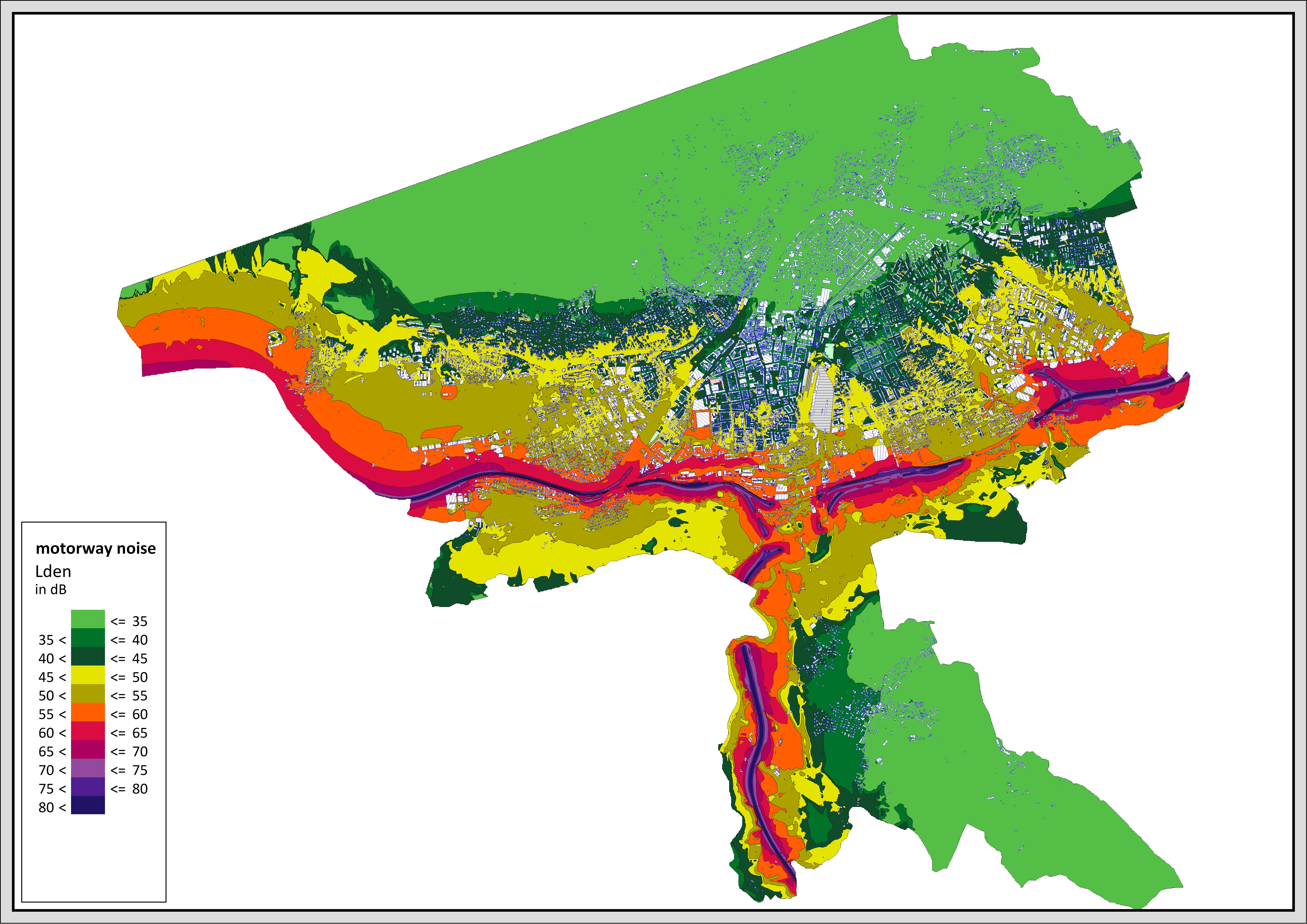

Supplement: Supplementary file 1 [file ijerph-16-03504-s001.zip › figure S 4 Lden_motorway.jpg]
